# Supplementary figures and images for: Efficacy and mechanism of high-purity HAMCC combined with CGF in promoting the repair of radiation-induced skin and soft tissue damage (part 1 of 3)
Source: PLoS One. 2025 Sep 9;20(9):e0330078. doi: 10.1371/journal.pone.0330078 (PMC12419615; doi:10.1371/journal.pone.0330078)

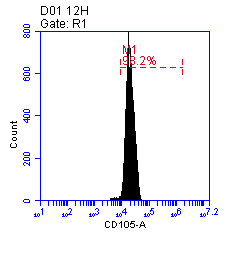

Supplement: S1 File — (ZIP) [file pone.0330078.s001.zip › Identificatioin of HAMCC/Flow cytometry/Figure/CD105.png]

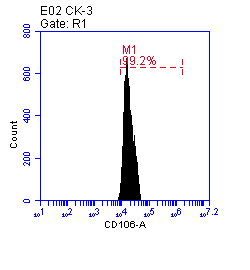

Supplement: S1 File — (ZIP) [file pone.0330078.s001.zip › Identificatioin of HAMCC/Flow cytometry/Figure/CD106.png]

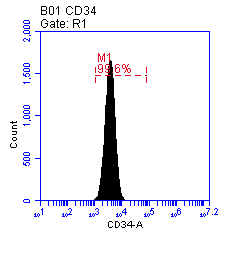

Supplement: S1 File — (ZIP) [file pone.0330078.s001.zip › Identificatioin of HAMCC/Flow cytometry/Figure/CD34.png]

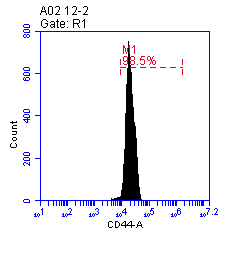

Supplement: S1 File — (ZIP) [file pone.0330078.s001.zip › Identificatioin of HAMCC/Flow cytometry/Figure/CD44.png]

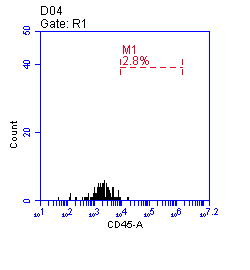

Supplement: S1 File — (ZIP) [file pone.0330078.s001.zip › Identificatioin of HAMCC/Flow cytometry/Figure/CD45.png]

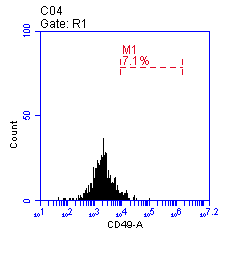

Supplement: S1 File — (ZIP) [file pone.0330078.s001.zip › Identificatioin of HAMCC/Flow cytometry/Figure/CD49.png]

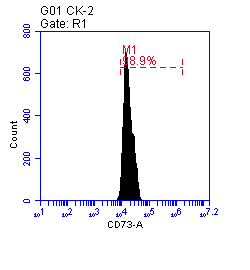

Supplement: S1 File — (ZIP) [file pone.0330078.s001.zip › Identificatioin of HAMCC/Flow cytometry/Figure/CD73.png]

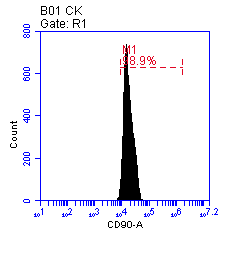

Supplement: S1 File — (ZIP) [file pone.0330078.s001.zip › Identificatioin of HAMCC/Flow cytometry/Figure/CD90.png]

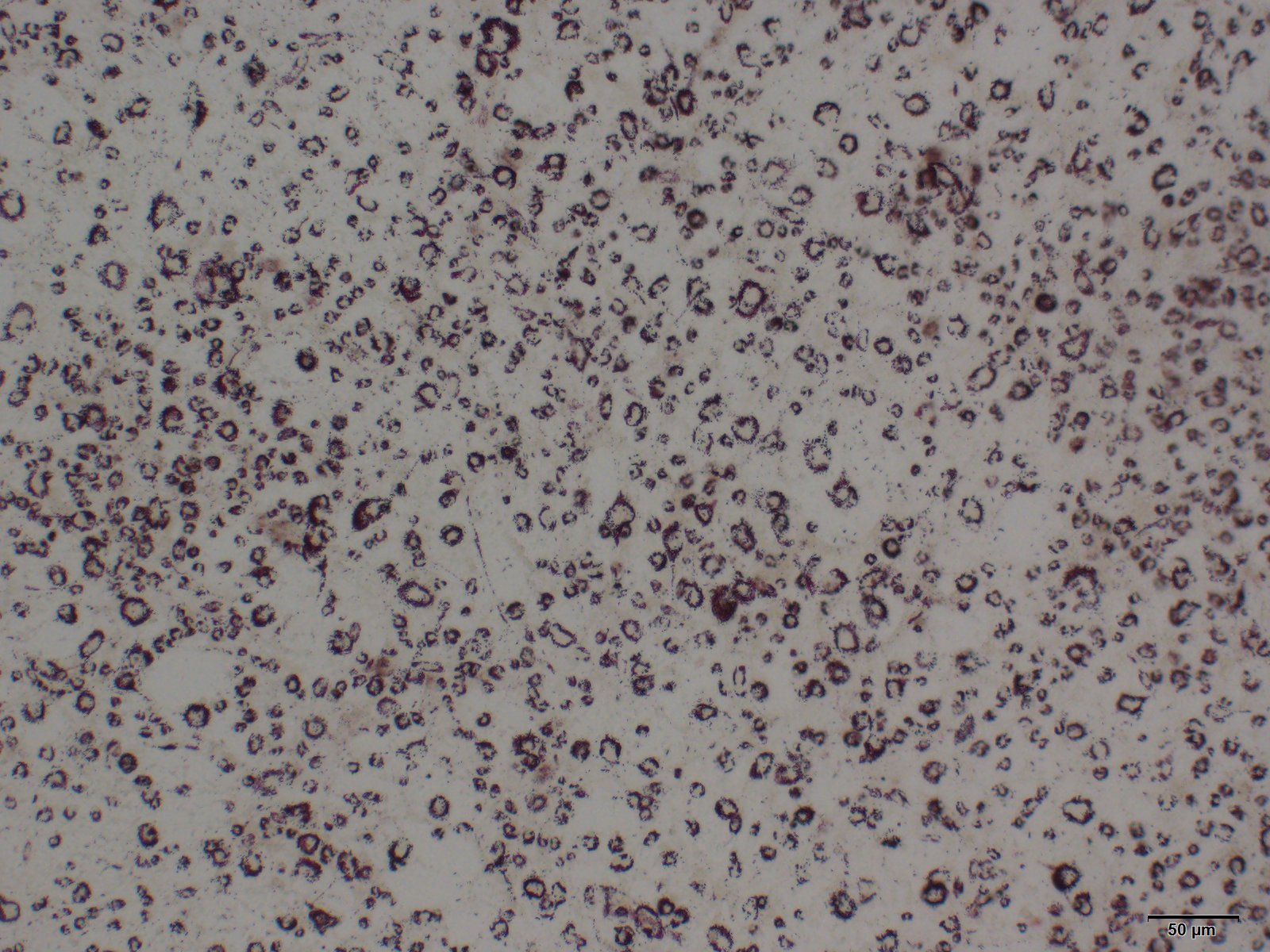

Supplement: S1 File — (ZIP) [file pone.0330078.s001.zip › Identificatioin of HAMCC/adipogenic induction/100X (2).jpg]

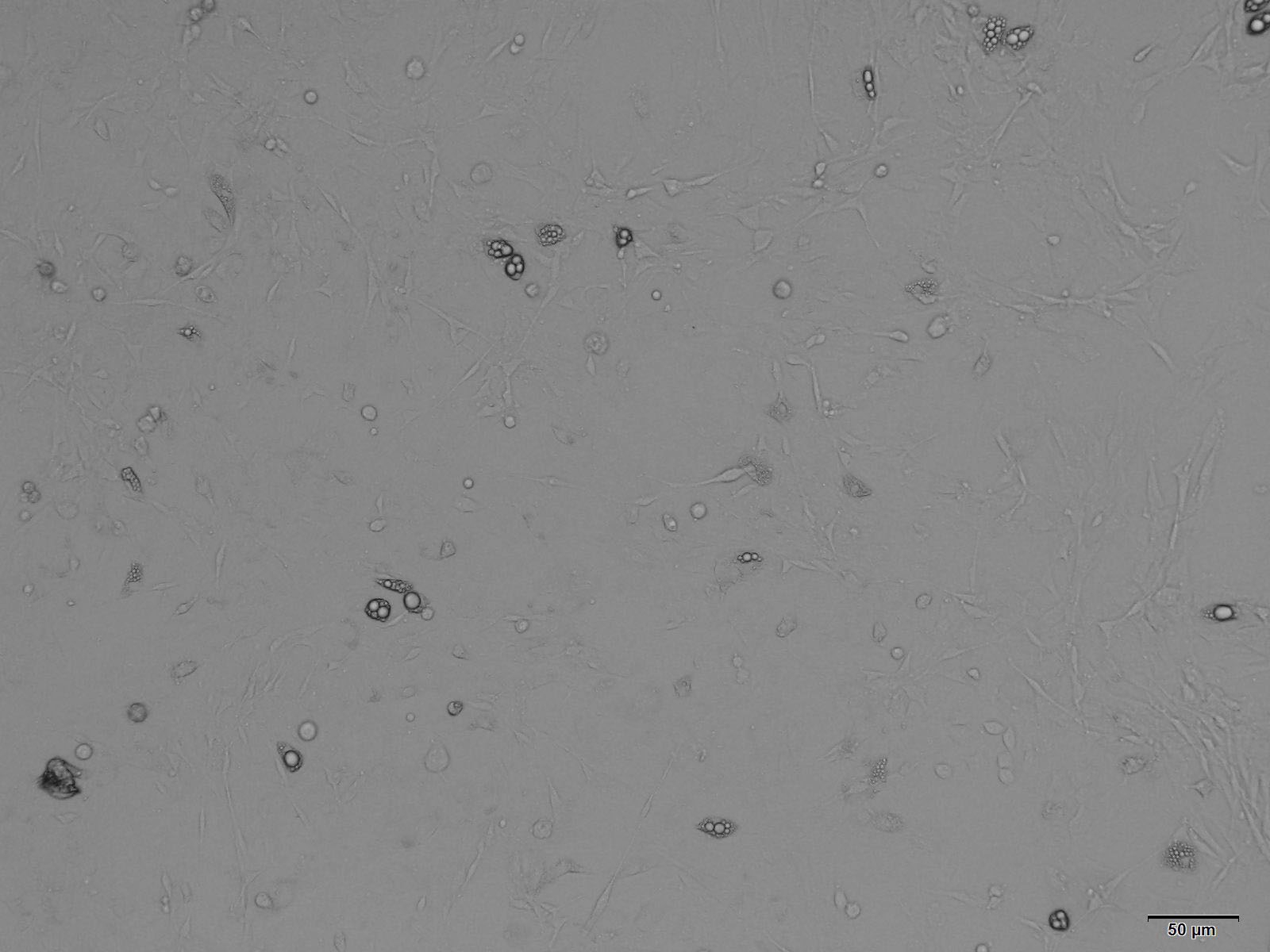

Supplement: S1 File — (ZIP) [file pone.0330078.s001.zip › Identificatioin of HAMCC/cell morphology observation/1d.jpg]

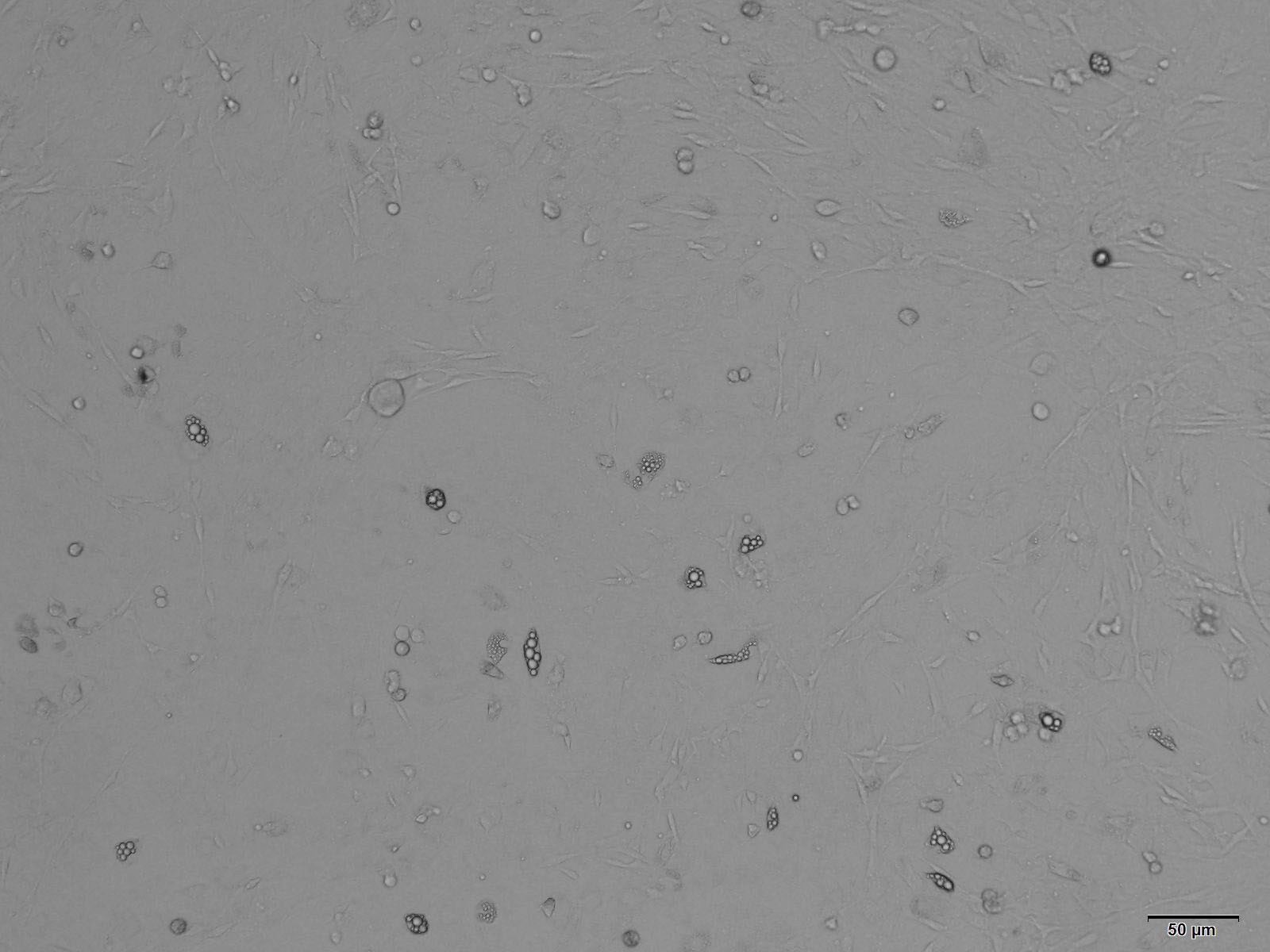

Supplement: S1 File — (ZIP) [file pone.0330078.s001.zip › Identificatioin of HAMCC/cell morphology observation/3d.jpg]

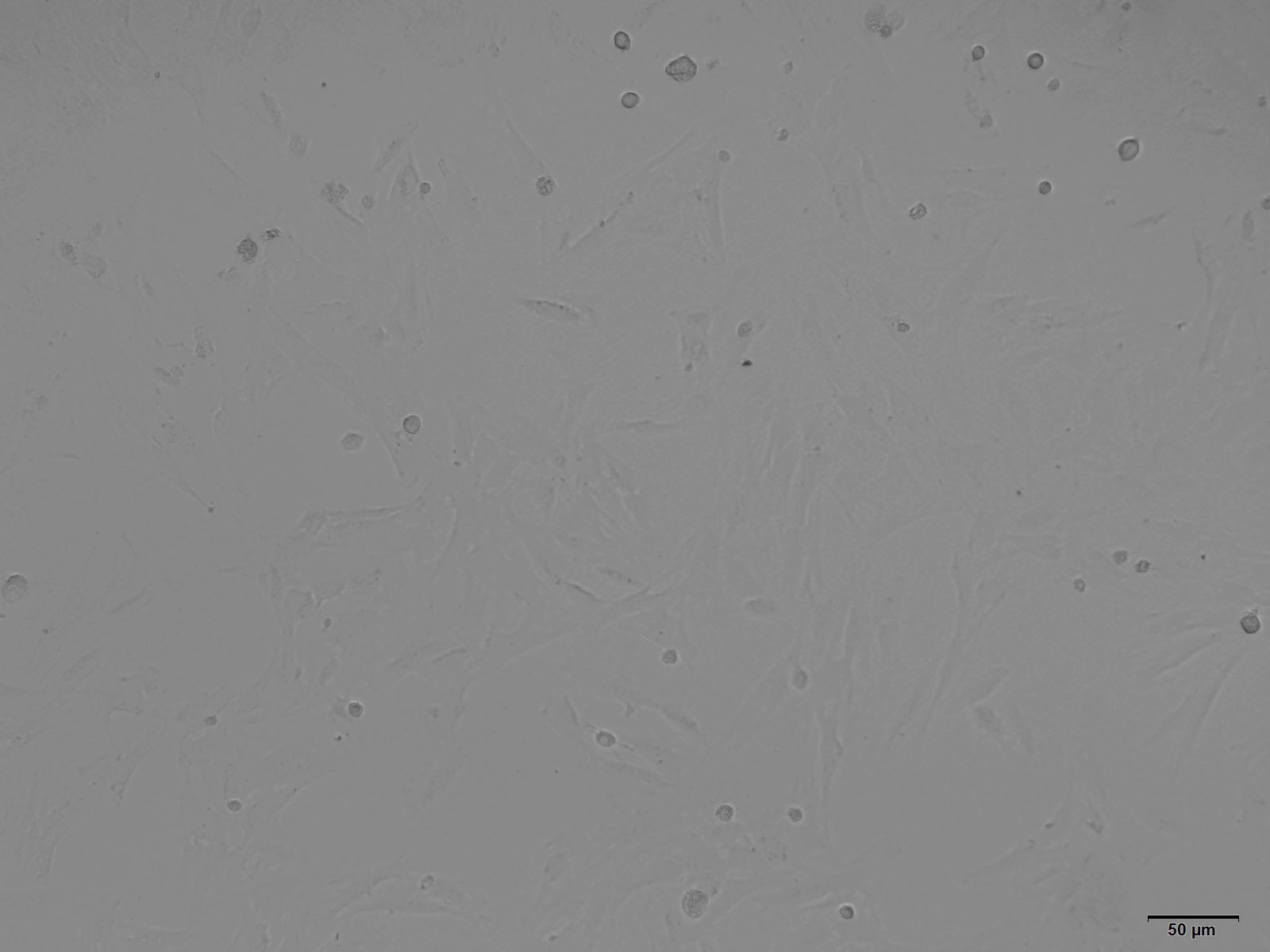

Supplement: S1 File — (ZIP) [file pone.0330078.s001.zip › Identificatioin of HAMCC/cell morphology observation/5d.jpg]

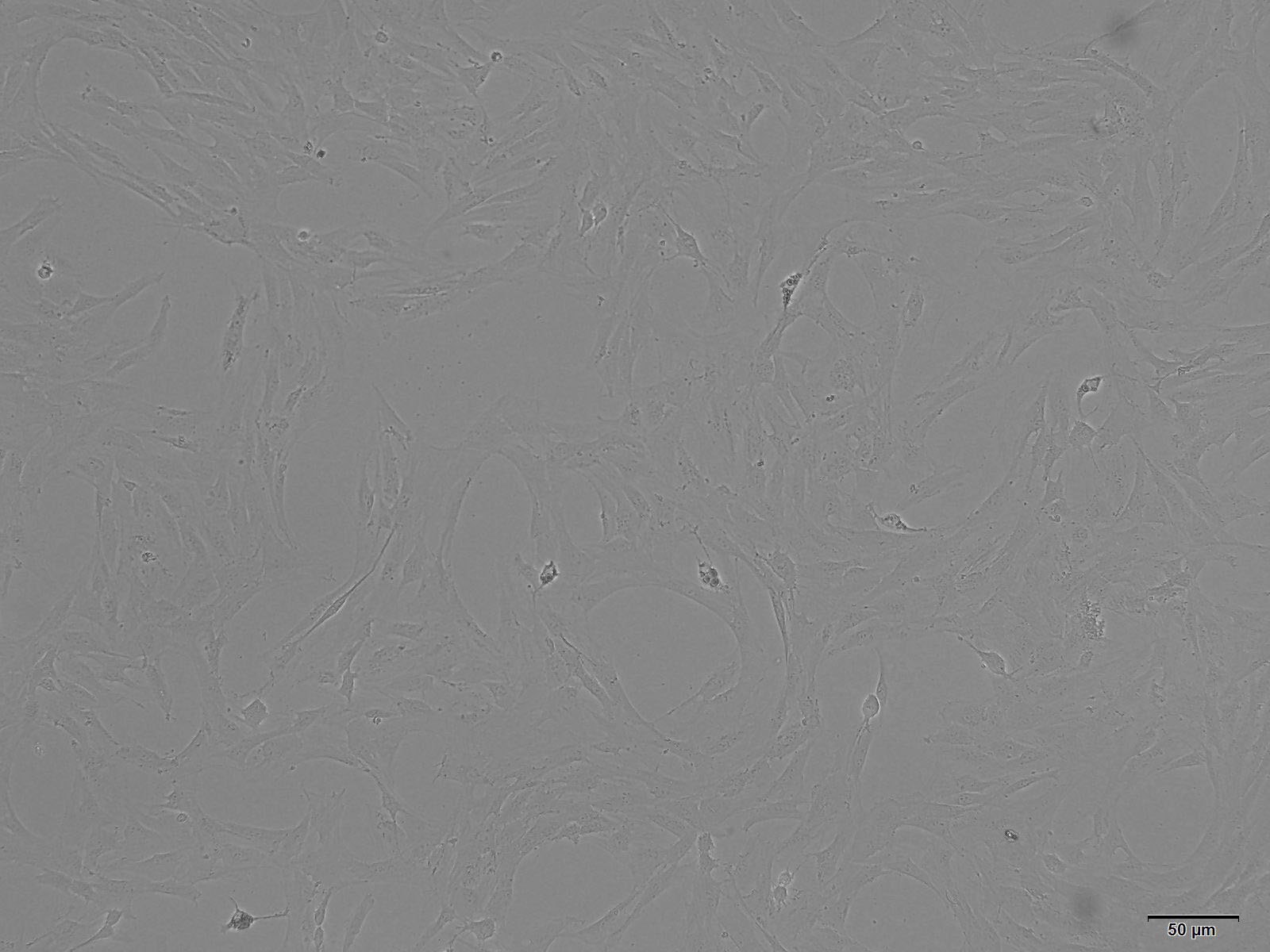

Supplement: S1 File — (ZIP) [file pone.0330078.s001.zip › Identificatioin of HAMCC/cell morphology observation/7d.jpg]

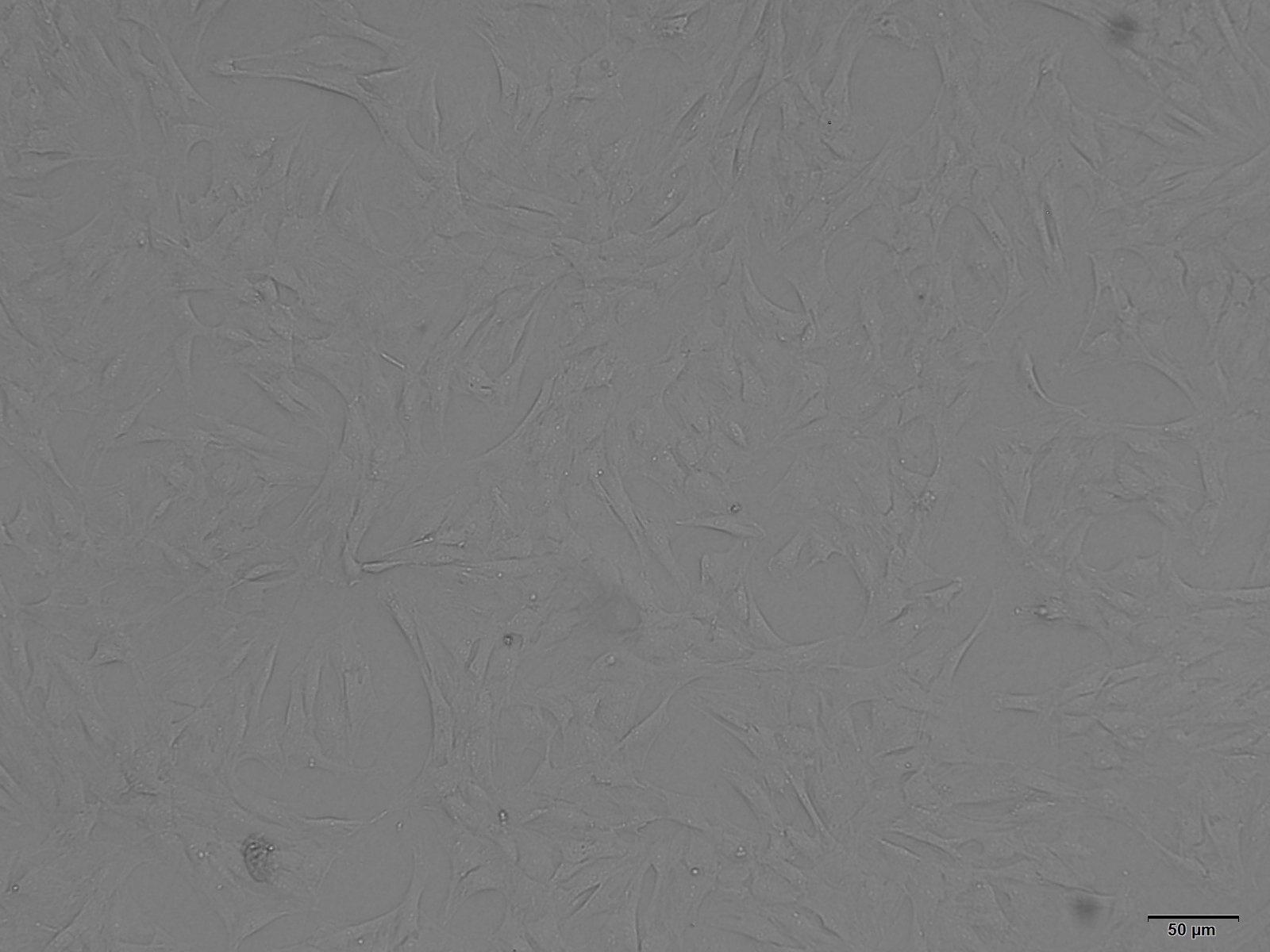

Supplement: S1 File — (ZIP) [file pone.0330078.s001.zip › Identificatioin of HAMCC/cell morphology observation/9d.jpg]

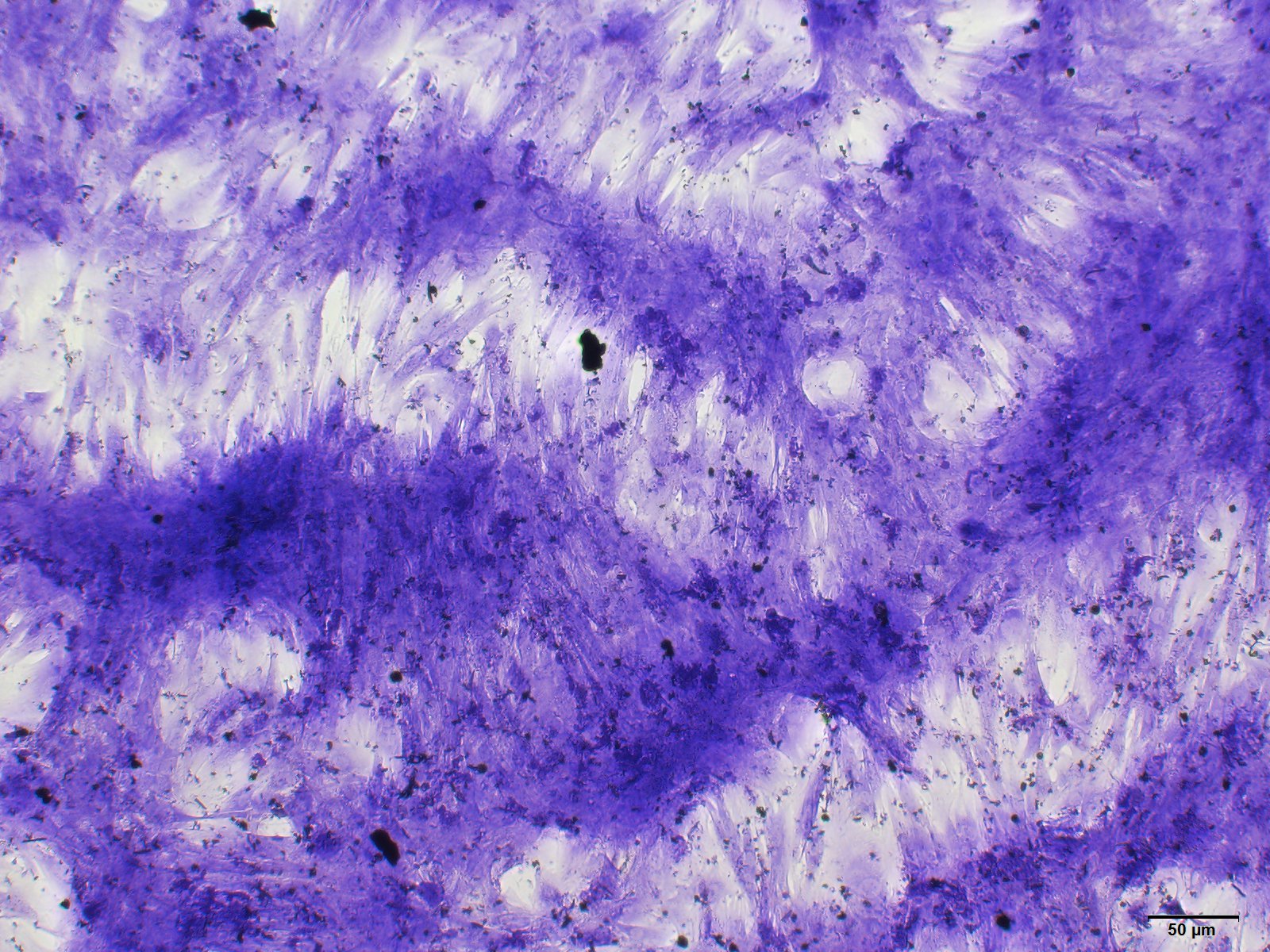

Supplement: S1 File — (ZIP) [file pone.0330078.s001.zip › Identificatioin of HAMCC/chondrogenic induction/100X (2).jpg]

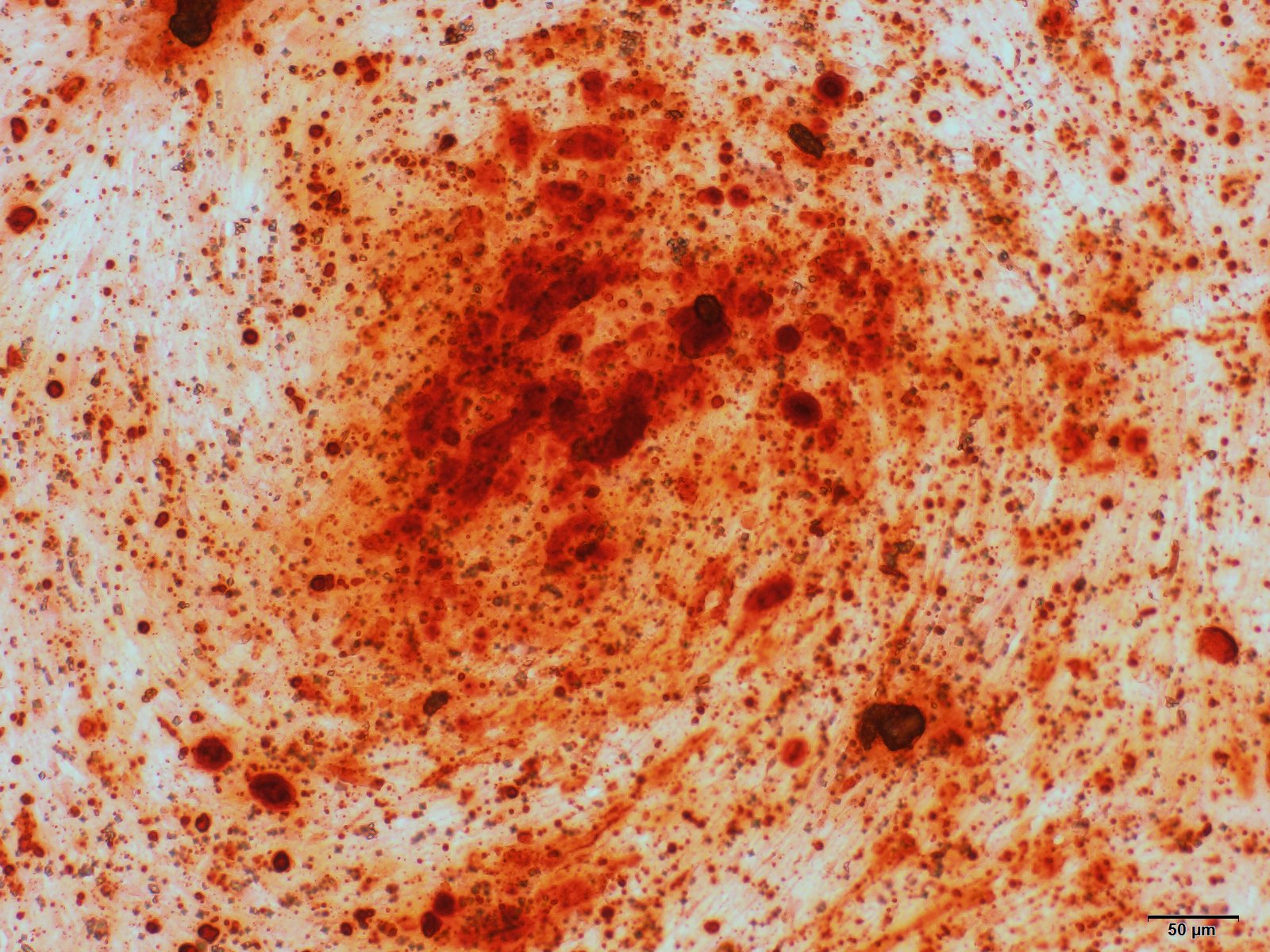

Supplement: S1 File — (ZIP) [file pone.0330078.s001.zip › Identificatioin of HAMCC/osteogenic induction/100X (2).jpg]

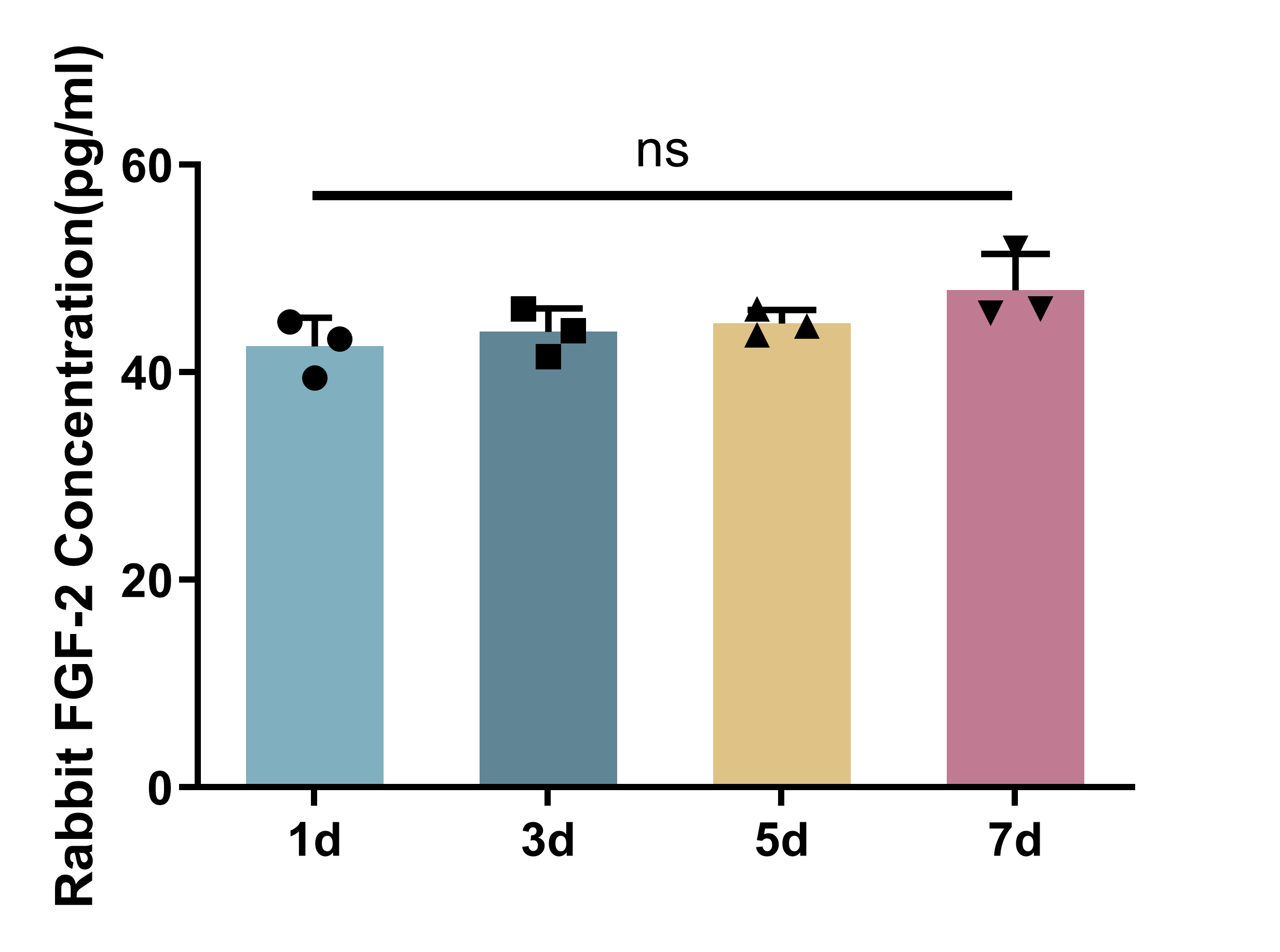

Supplement: S2 File — (ZIP) [file pone.0330078.s002.zip › Identification of the efficient component in CGF/2.ELISA/Figure/FGF-2.tif]

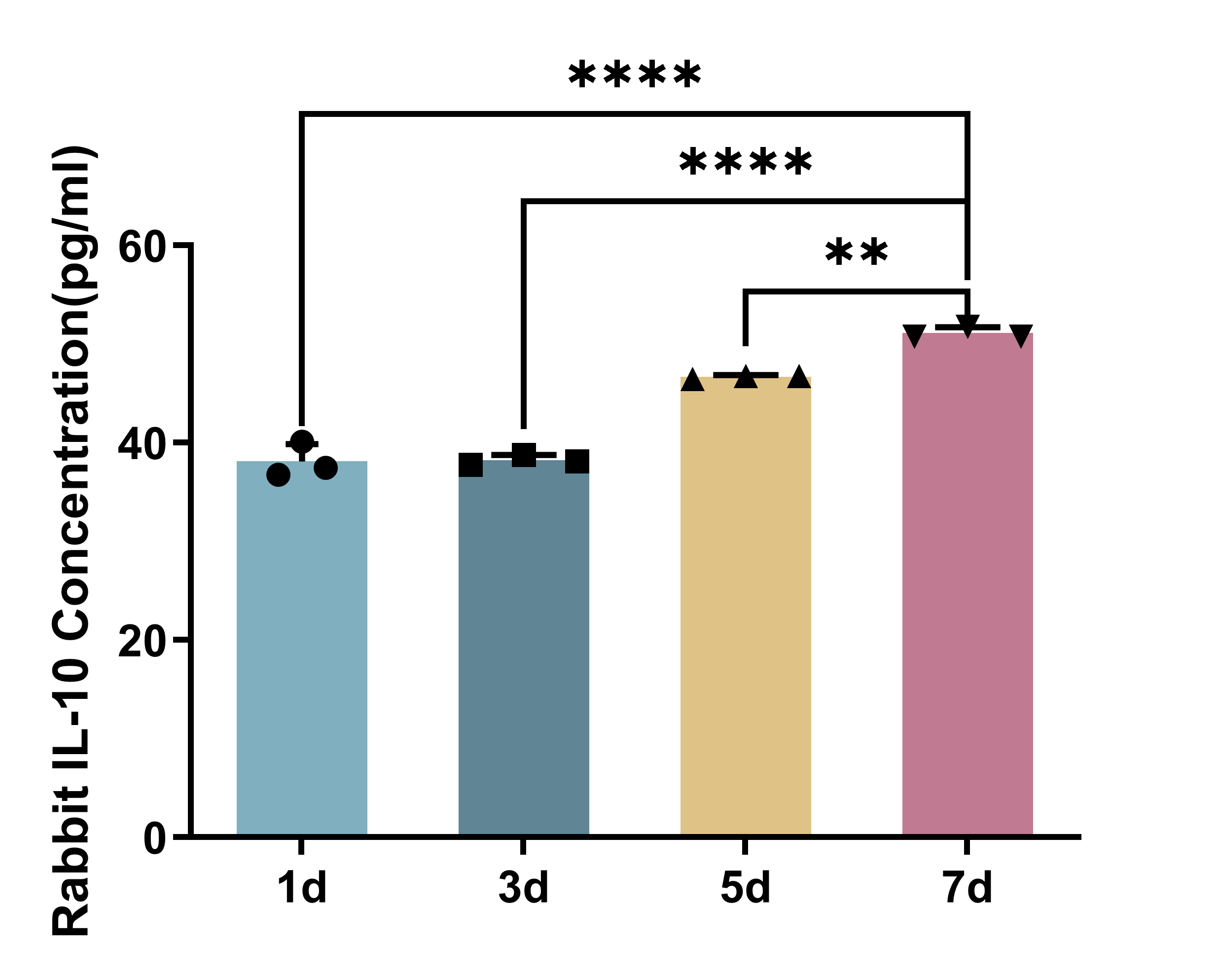

Supplement: S2 File — (ZIP) [file pone.0330078.s002.zip › Identification of the efficient component in CGF/2.ELISA/Figure/IL-10.tif]

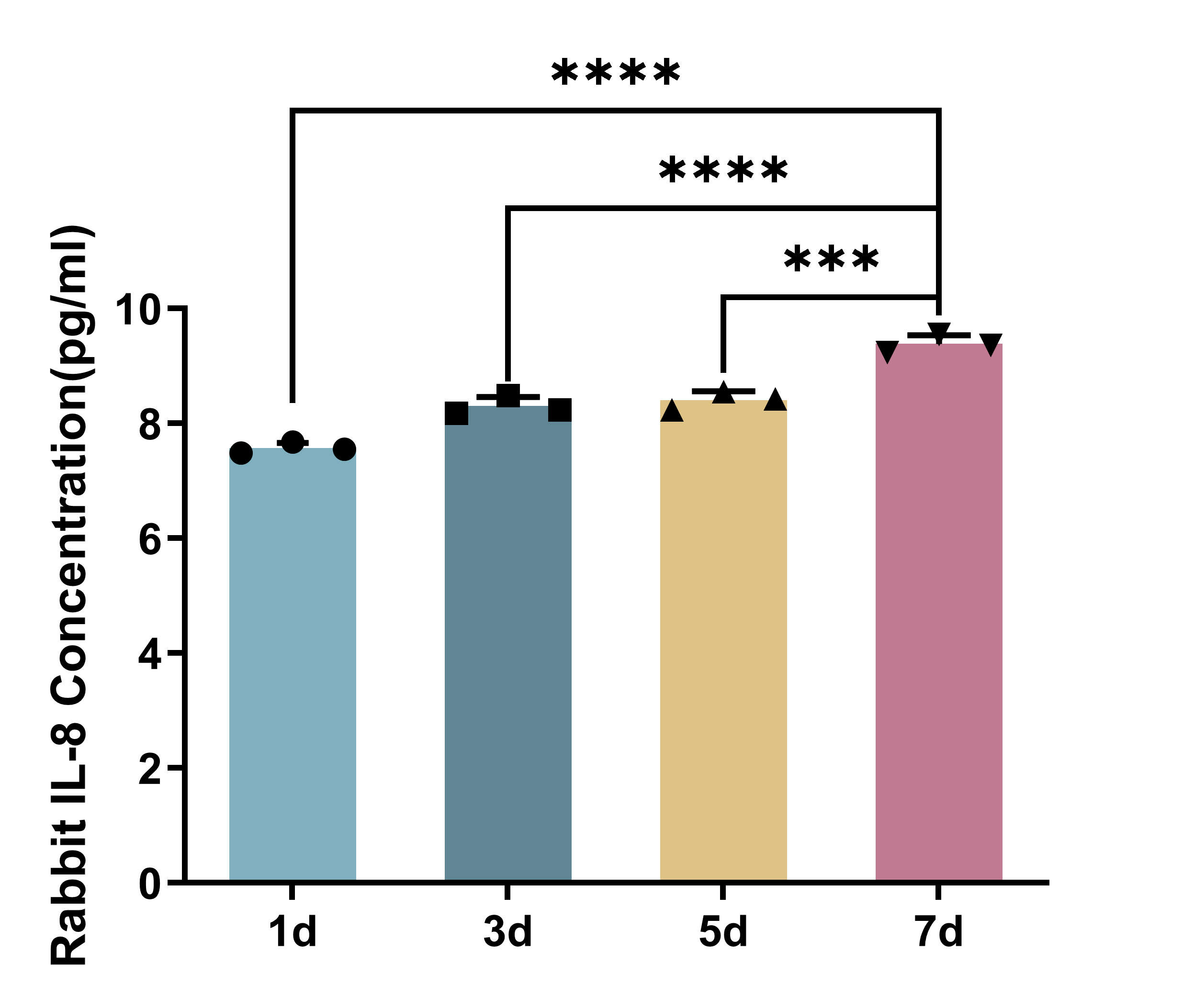

Supplement: S2 File — (ZIP) [file pone.0330078.s002.zip › Identification of the efficient component in CGF/2.ELISA/Figure/IL-8.tif]

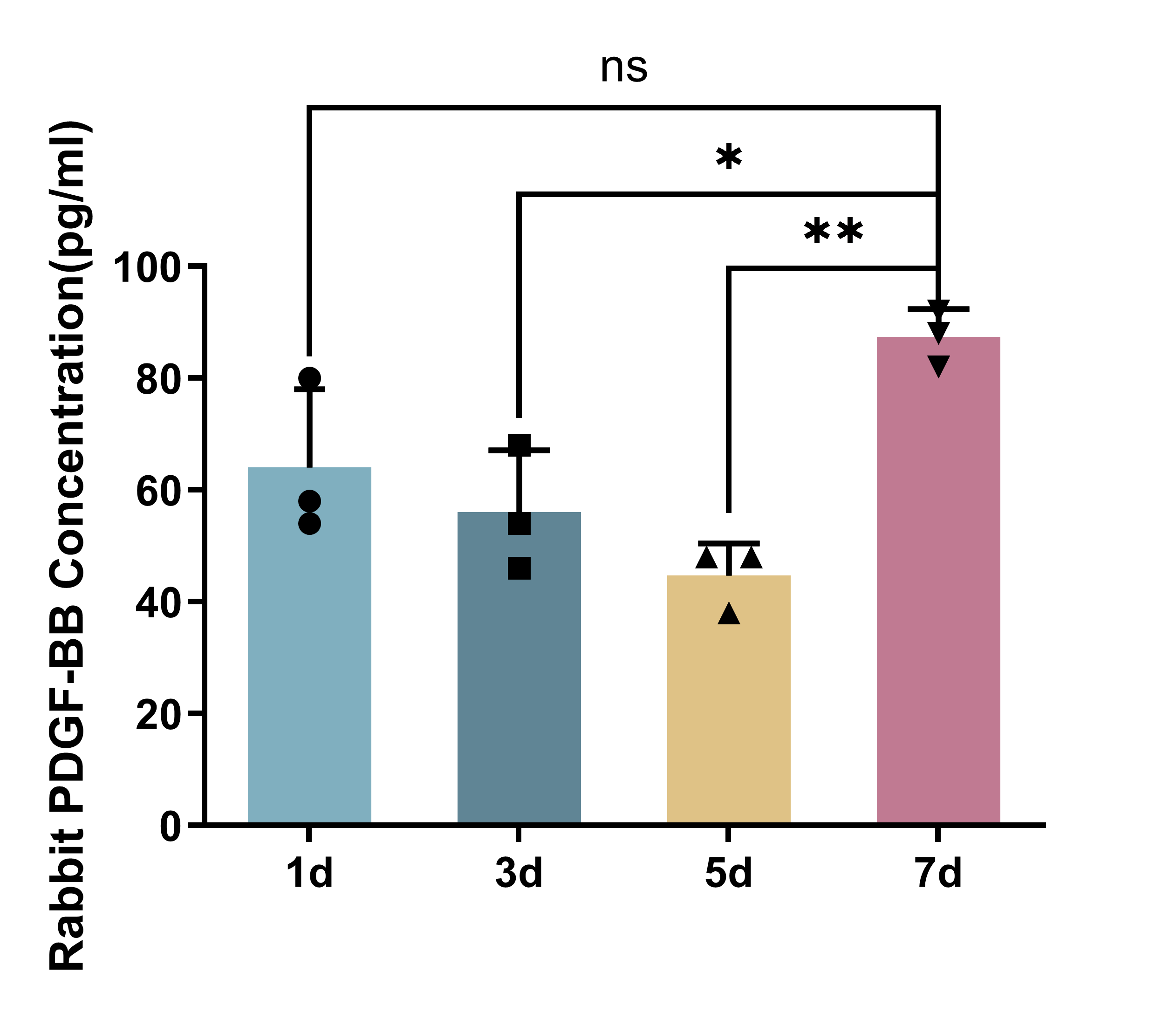

Supplement: S2 File — (ZIP) [file pone.0330078.s002.zip › Identification of the efficient component in CGF/2.ELISA/Figure/PDGF-BB.tif]

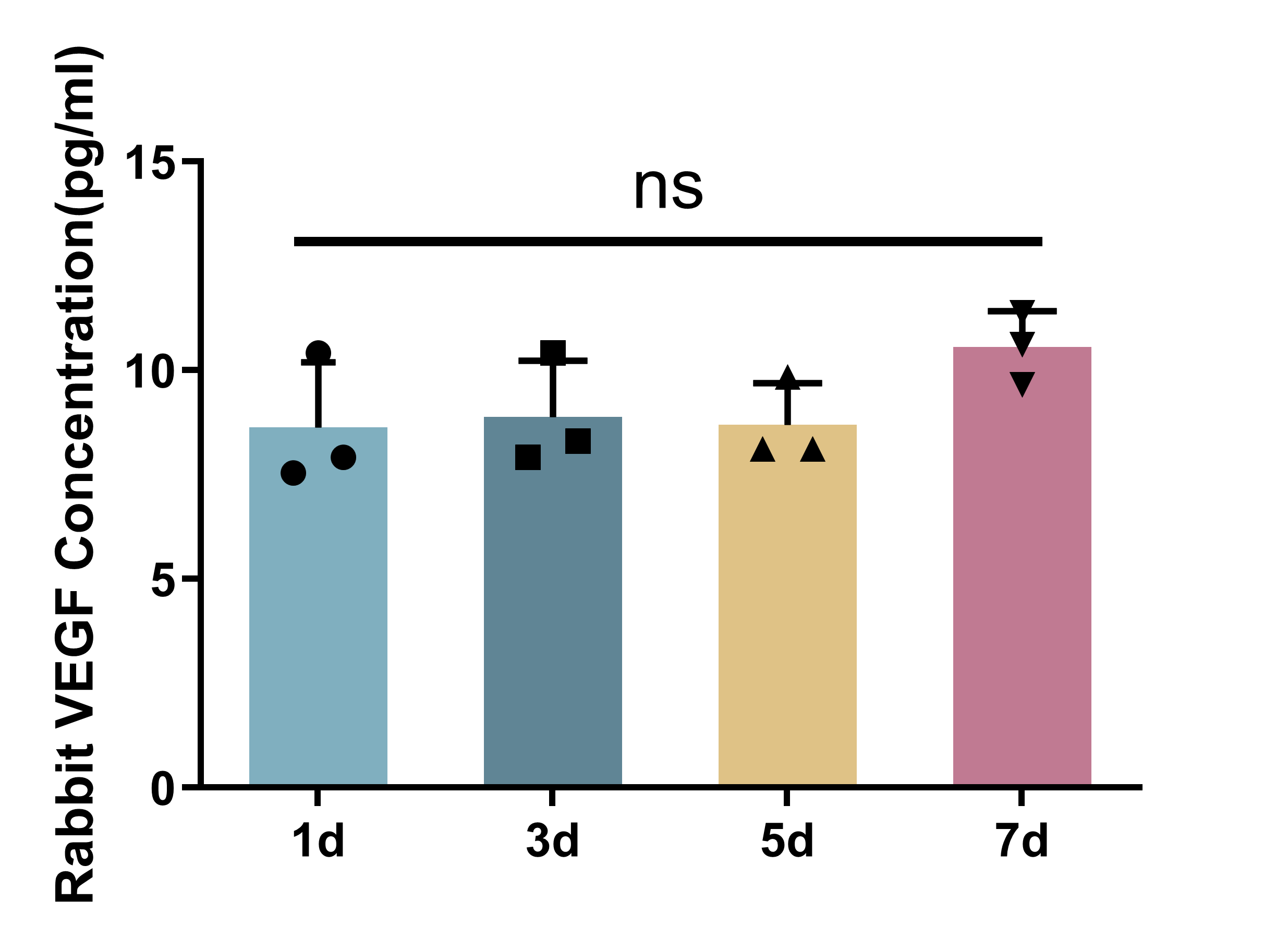

Supplement: S2 File — (ZIP) [file pone.0330078.s002.zip › Identification of the efficient component in CGF/2.ELISA/Figure/VEGF.tif]

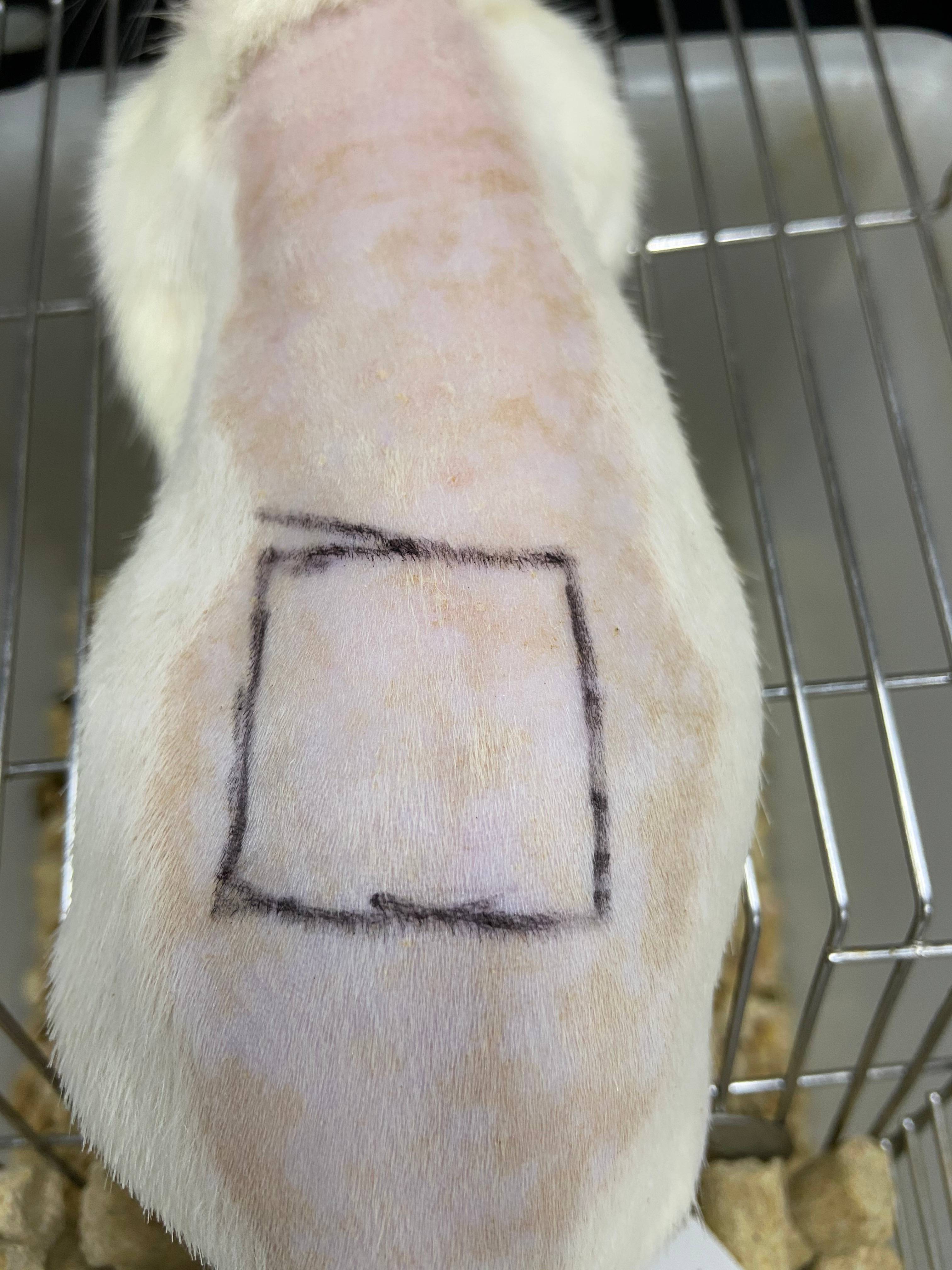

Supplement: S3 File — (ZIP) [file pone.0330078.s003.zip › Animal experiment/CGF/0d 1.jpg]

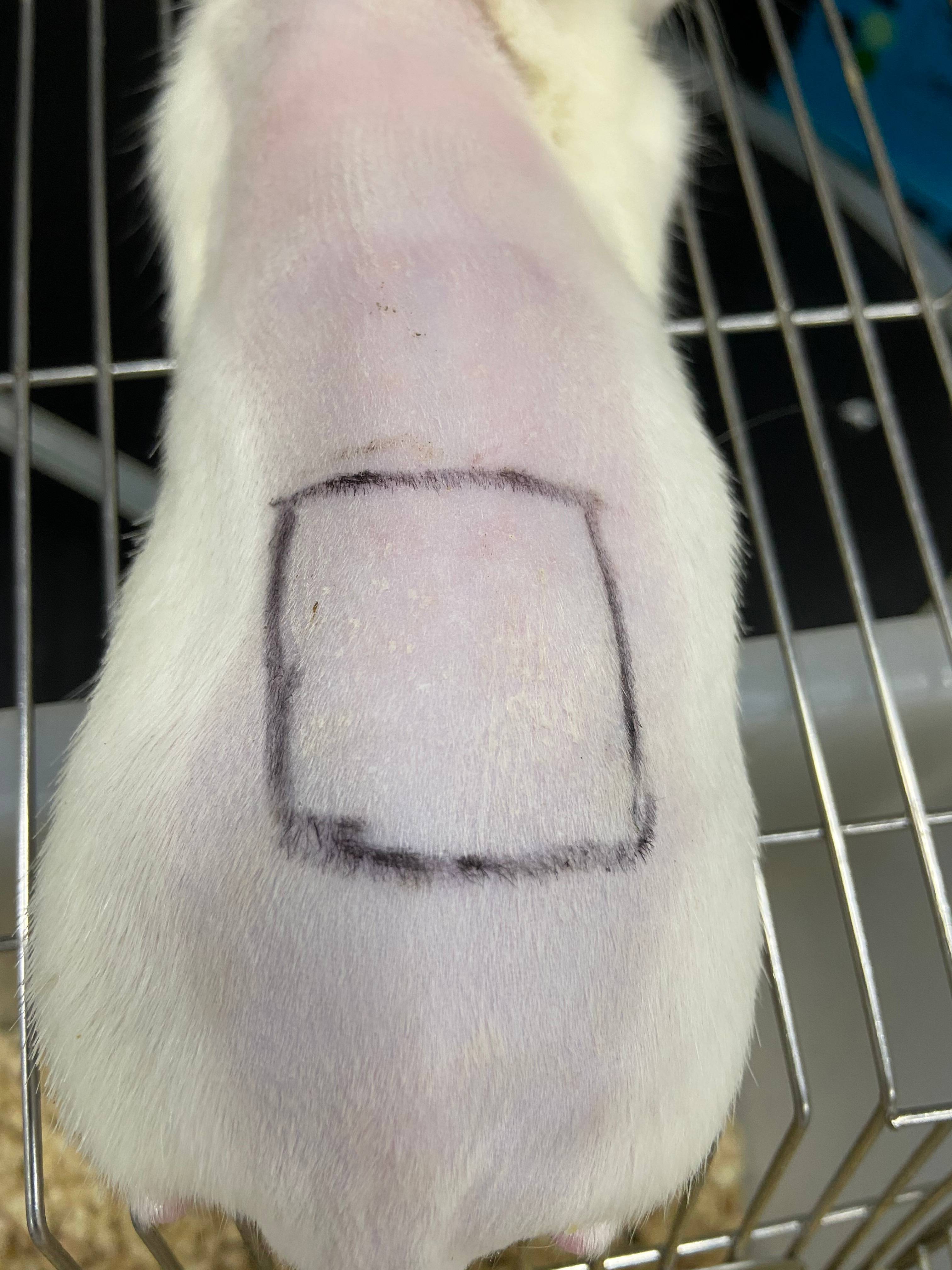

Supplement: S3 File — (ZIP) [file pone.0330078.s003.zip › Animal experiment/CGF/0d 2.jpg]

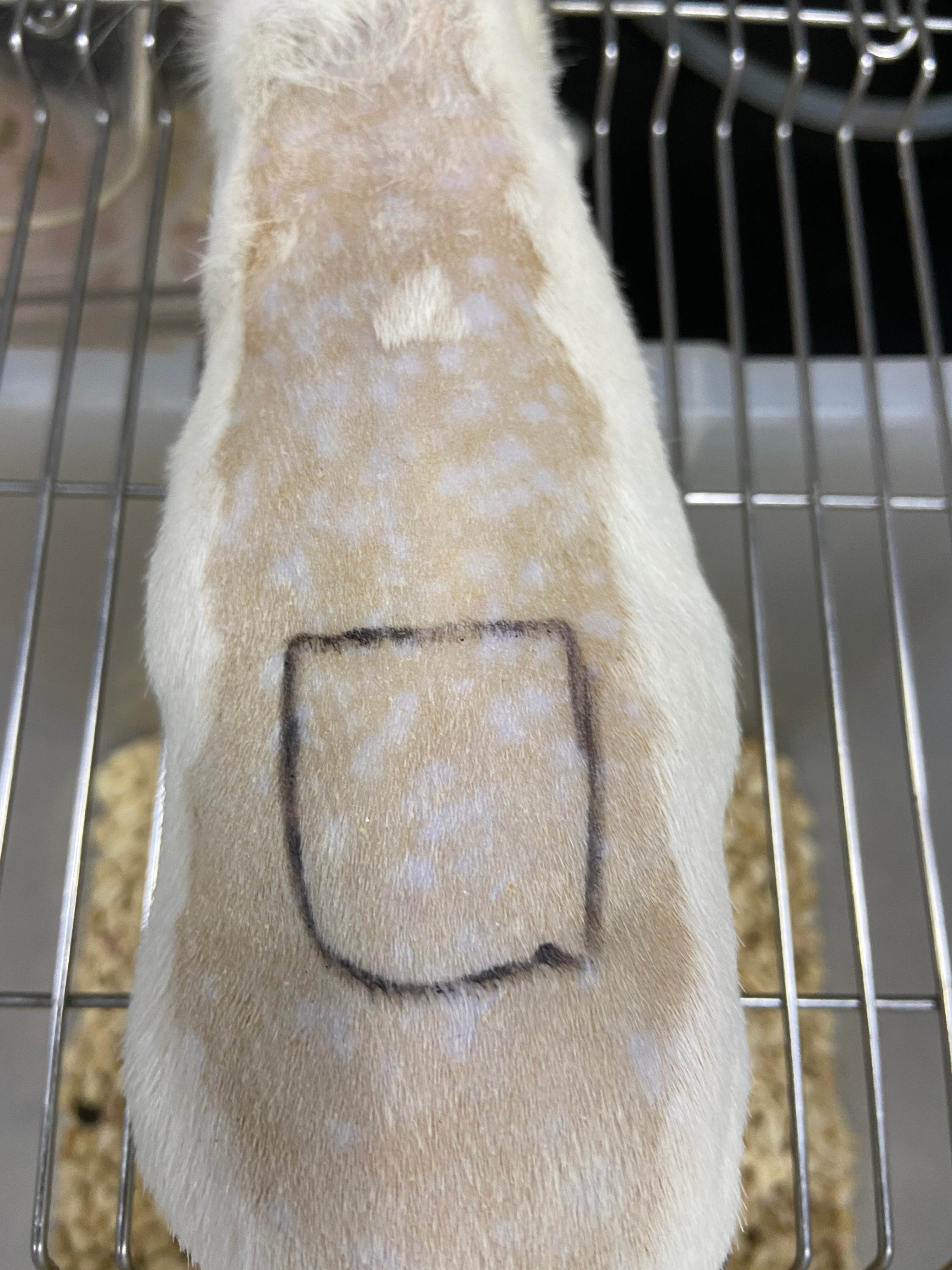

Supplement: S3 File — (ZIP) [file pone.0330078.s003.zip › Animal experiment/CGF/0d 3.jpg]

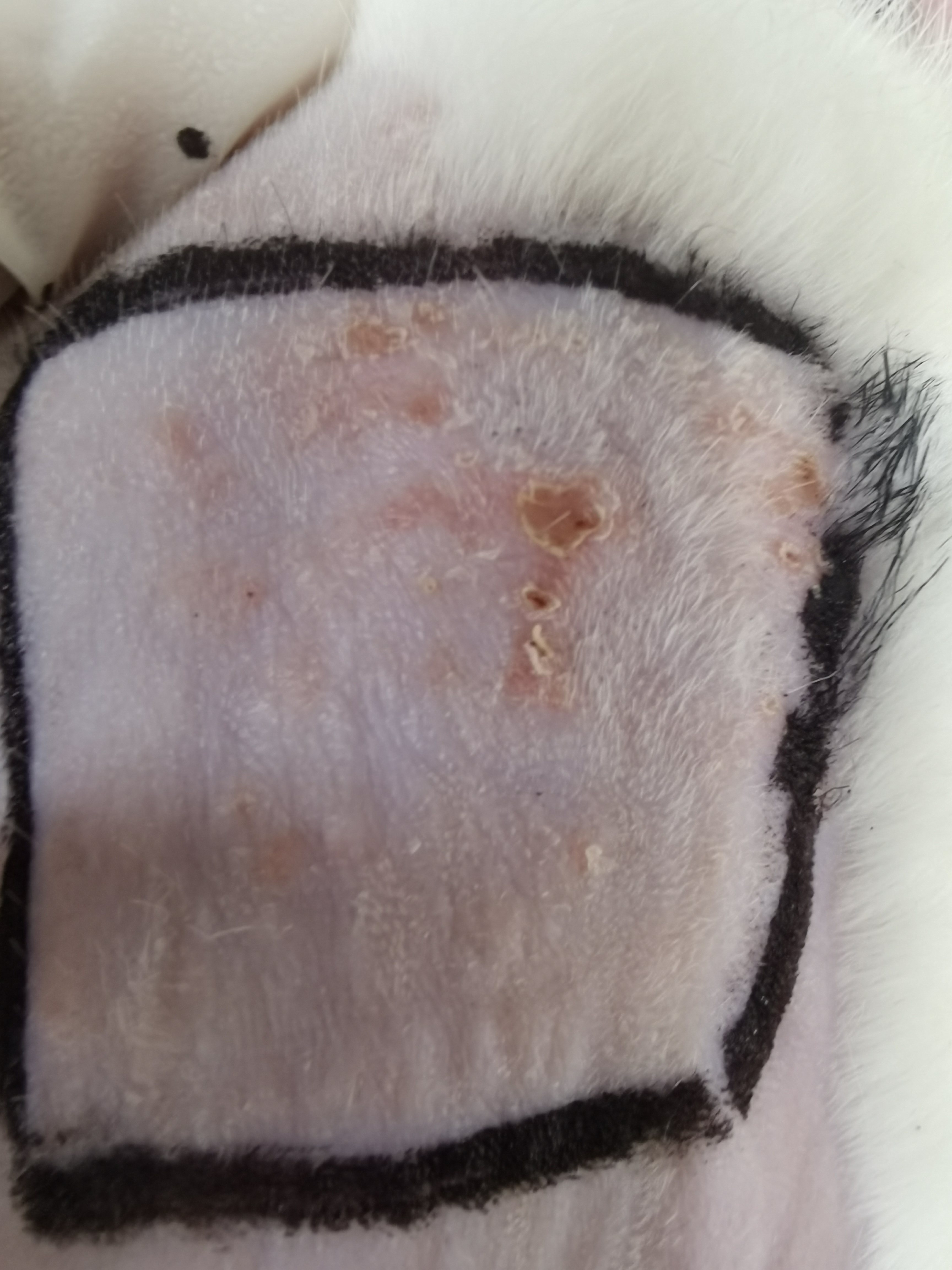

Supplement: S3 File — (ZIP) [file pone.0330078.s003.zip › Animal experiment/CGF/14d 1.jpg]

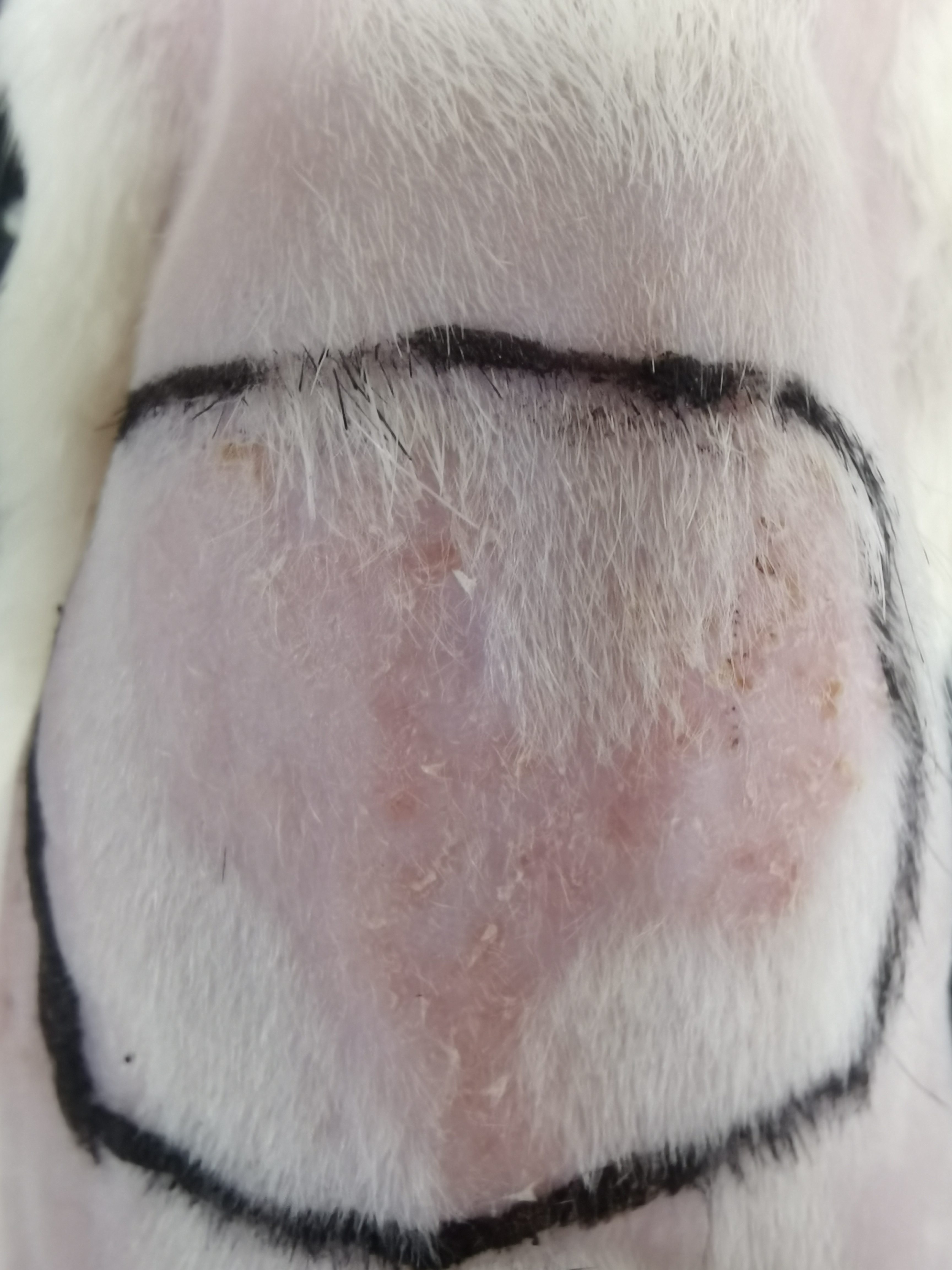

Supplement: S3 File — (ZIP) [file pone.0330078.s003.zip › Animal experiment/CGF/14d 2.jpg]

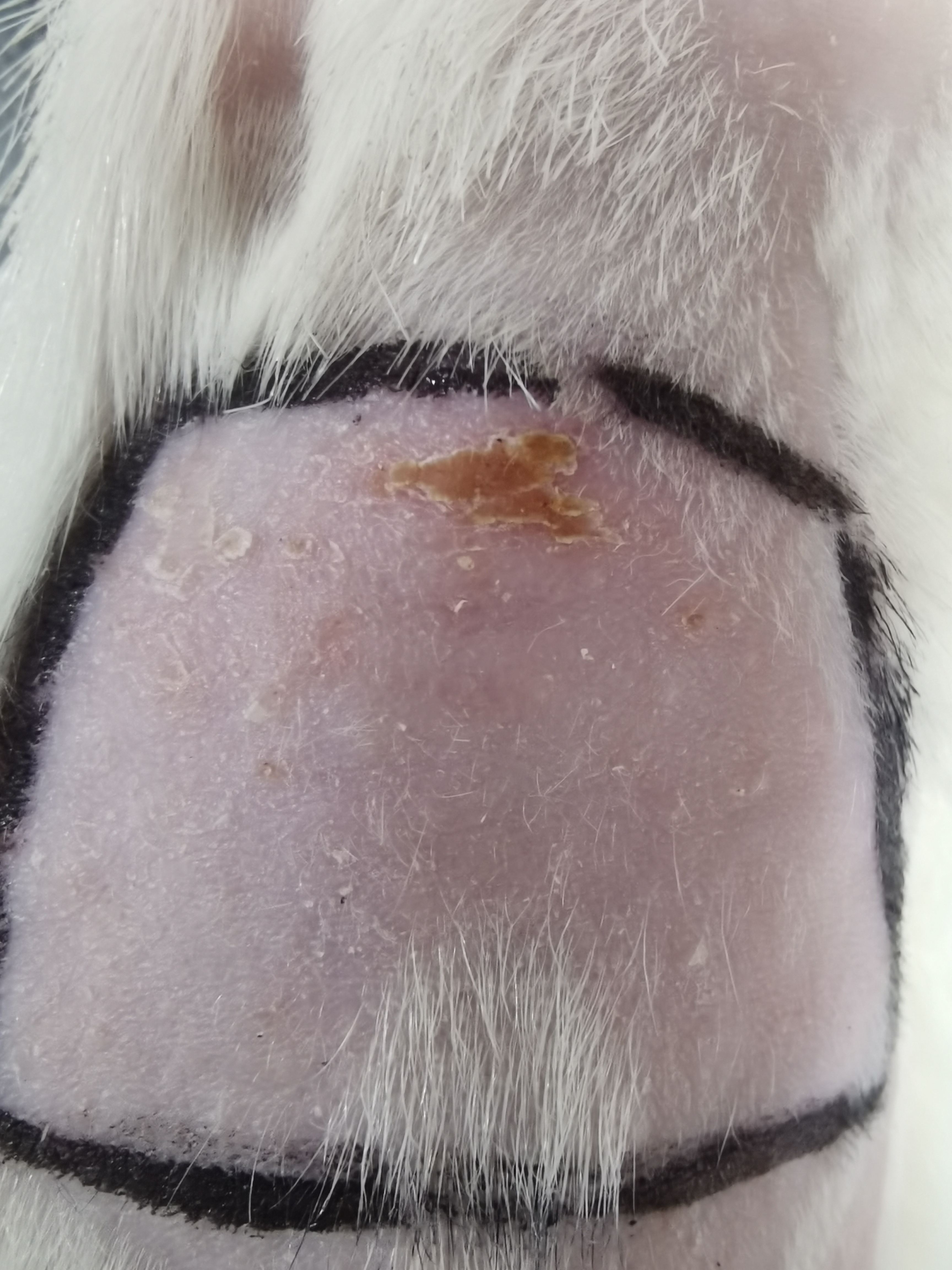

Supplement: S3 File — (ZIP) [file pone.0330078.s003.zip › Animal experiment/CGF/14d 3.jpg]

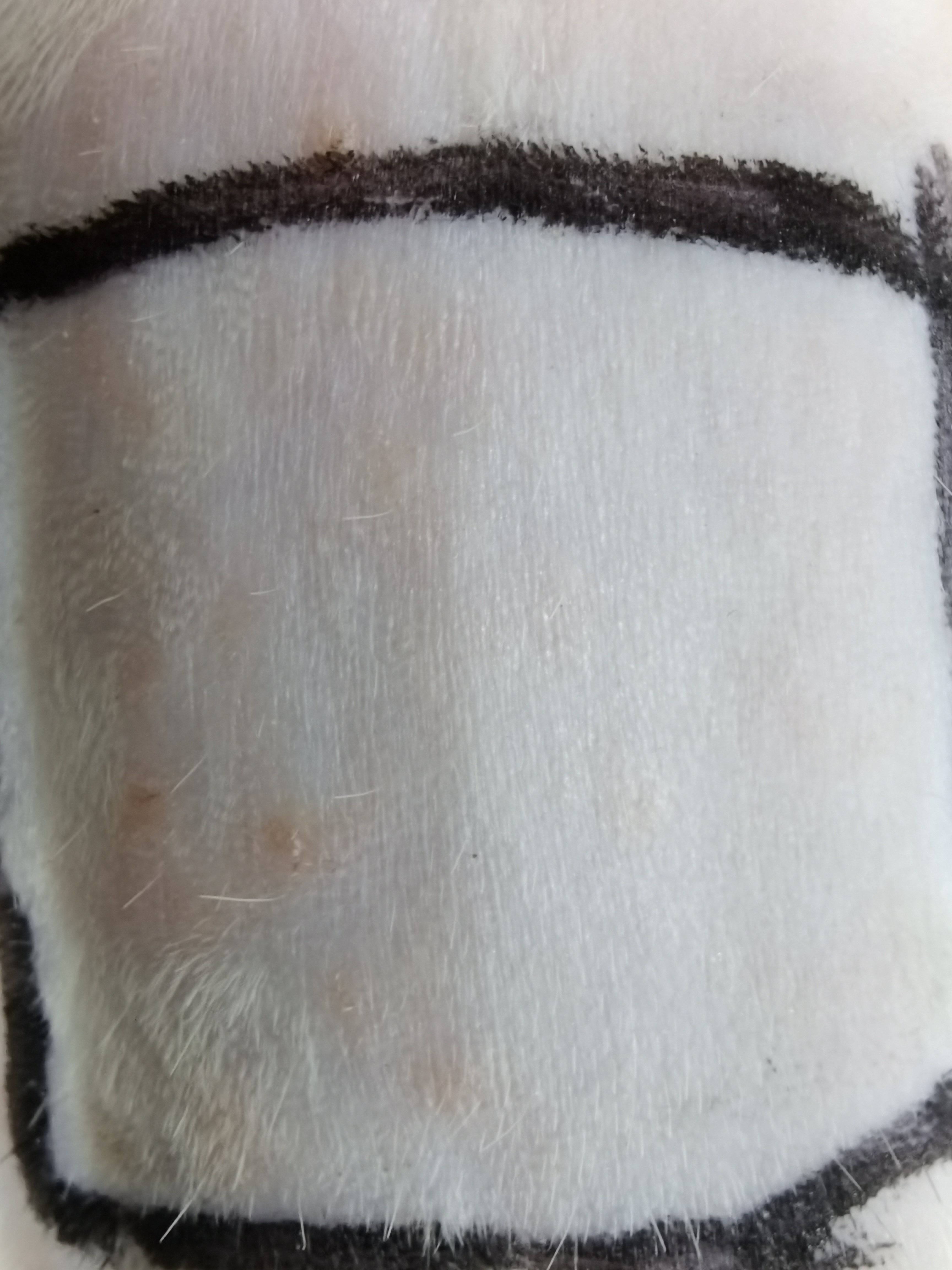

Supplement: S3 File — (ZIP) [file pone.0330078.s003.zip › Animal experiment/CGF/21d 1.jpg]

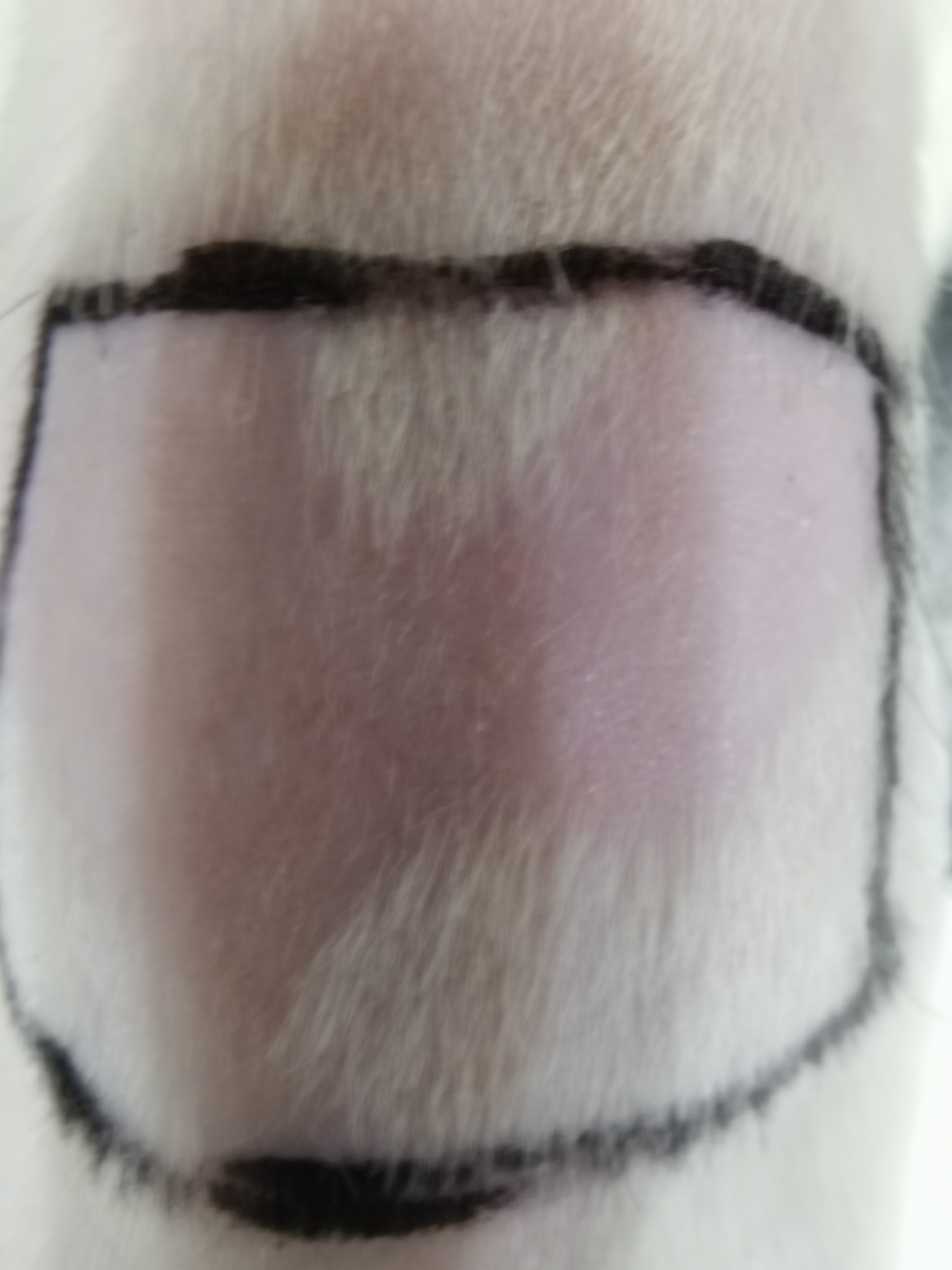

Supplement: S3 File — (ZIP) [file pone.0330078.s003.zip › Animal experiment/CGF/21d 2.jpg]

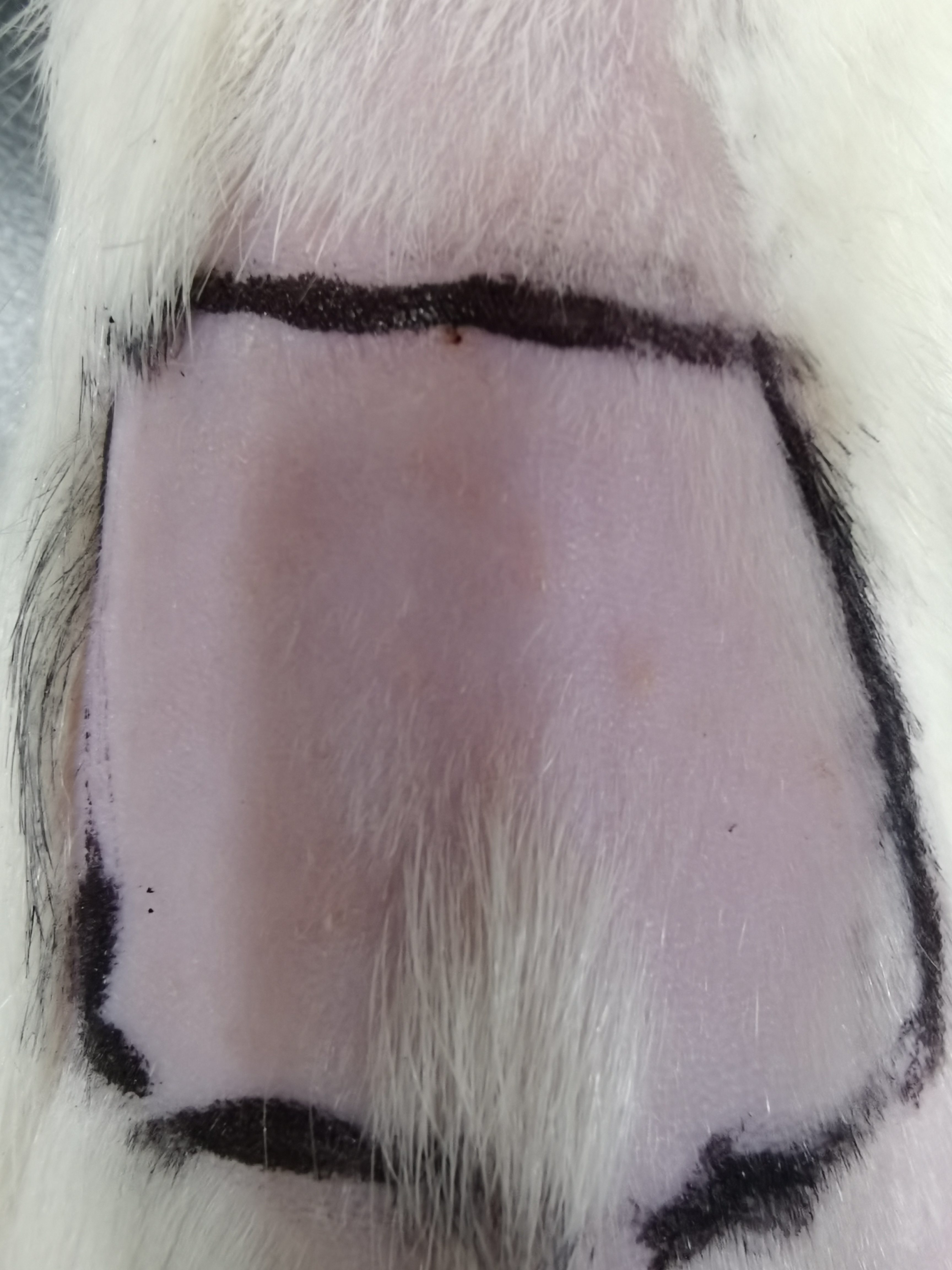

Supplement: S3 File — (ZIP) [file pone.0330078.s003.zip › Animal experiment/CGF/21d 3.jpg]

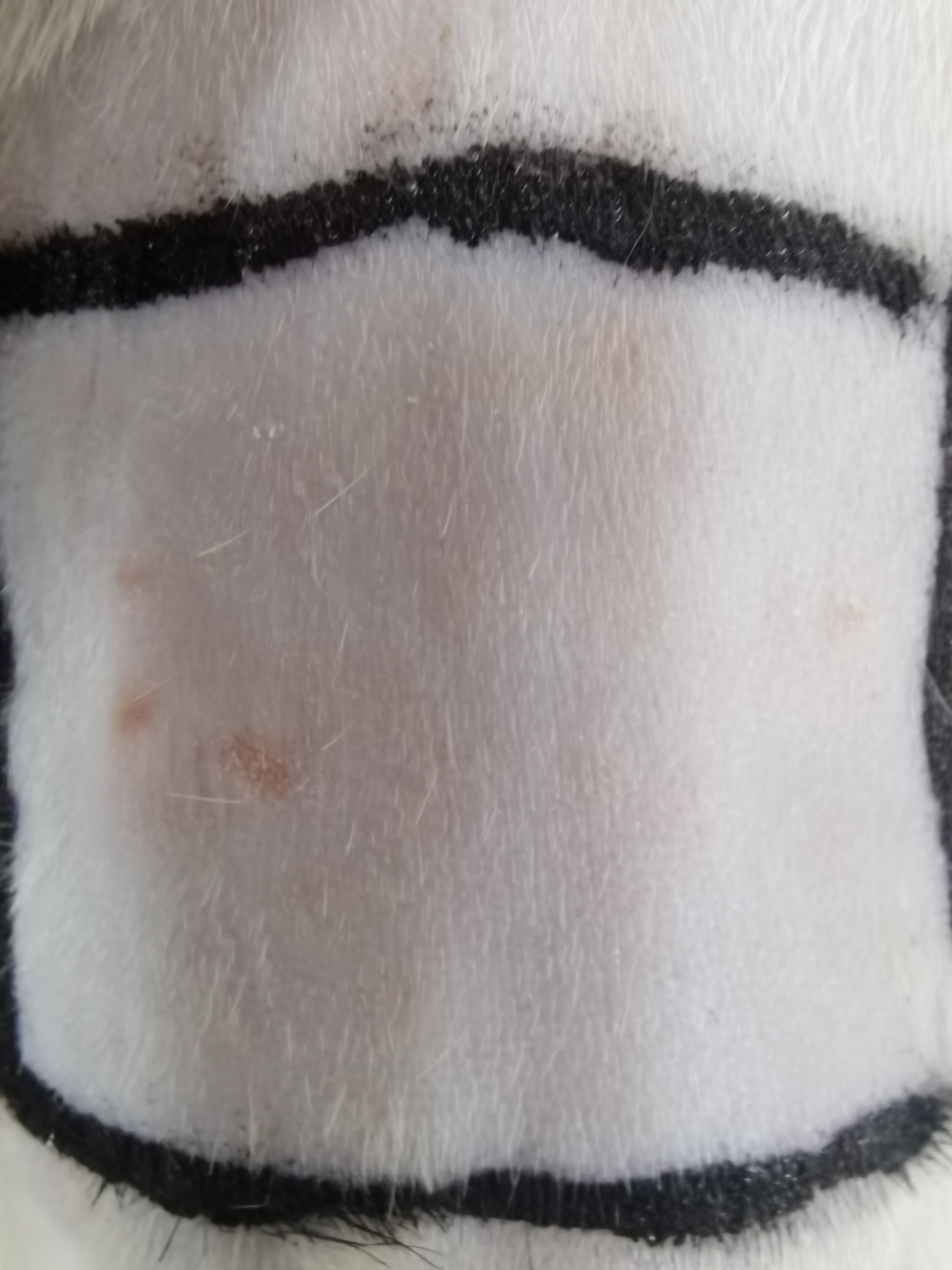

Supplement: S3 File — (ZIP) [file pone.0330078.s003.zip › Animal experiment/CGF/28d 1.jpg]

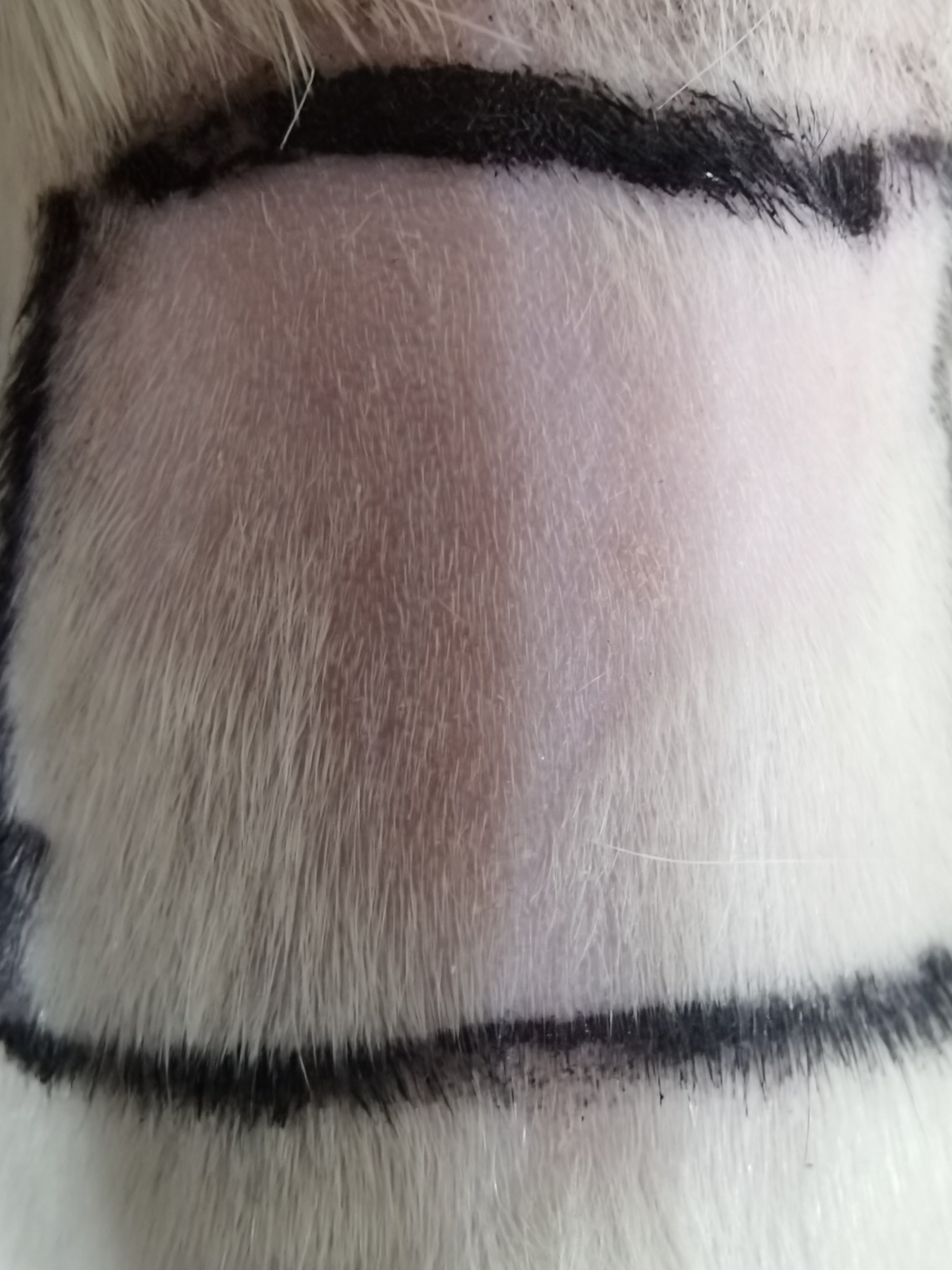

Supplement: S3 File — (ZIP) [file pone.0330078.s003.zip › Animal experiment/CGF/28d 2.jpg]

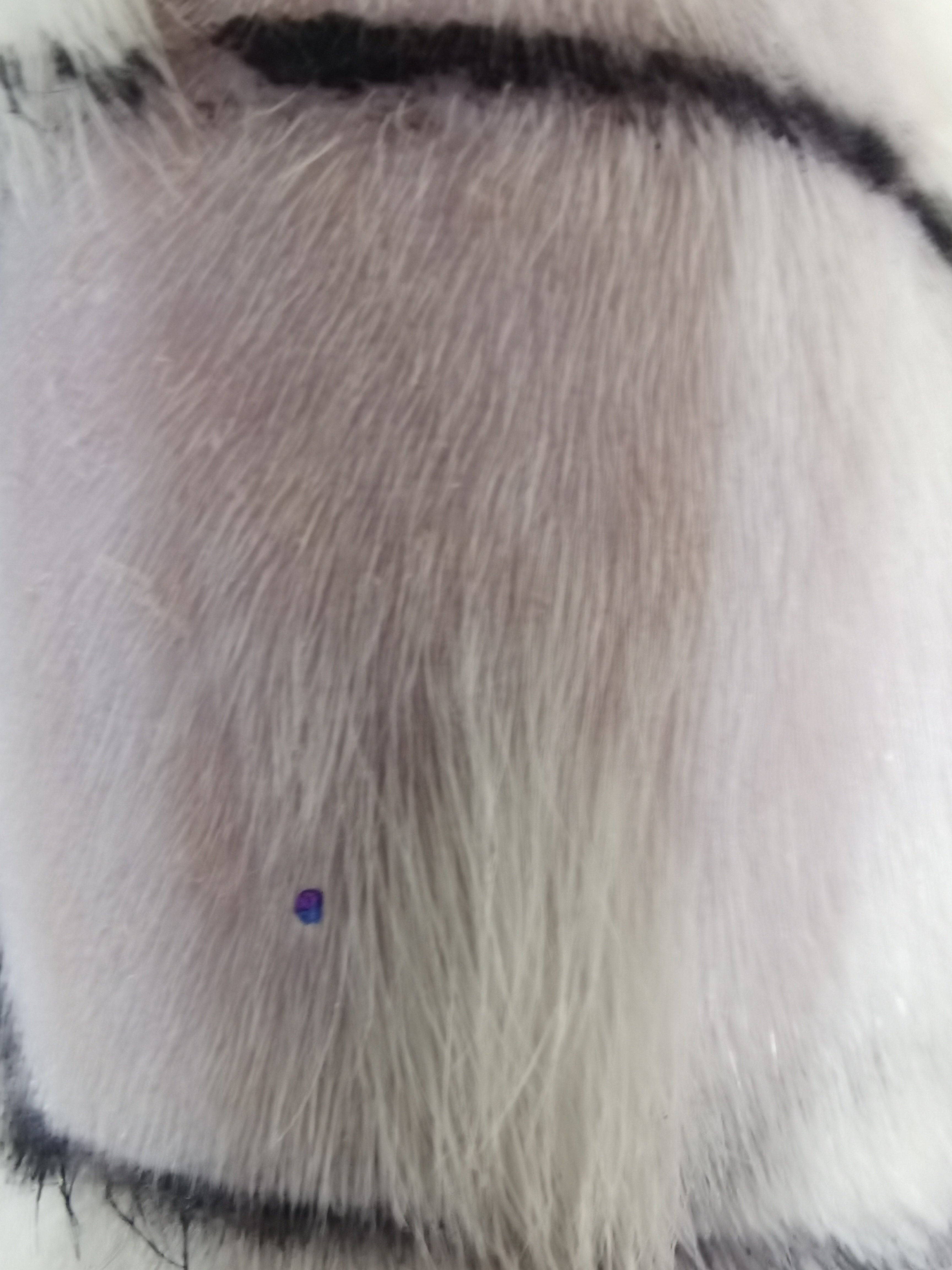

Supplement: S3 File — (ZIP) [file pone.0330078.s003.zip › Animal experiment/CGF/28d 3.jpg]

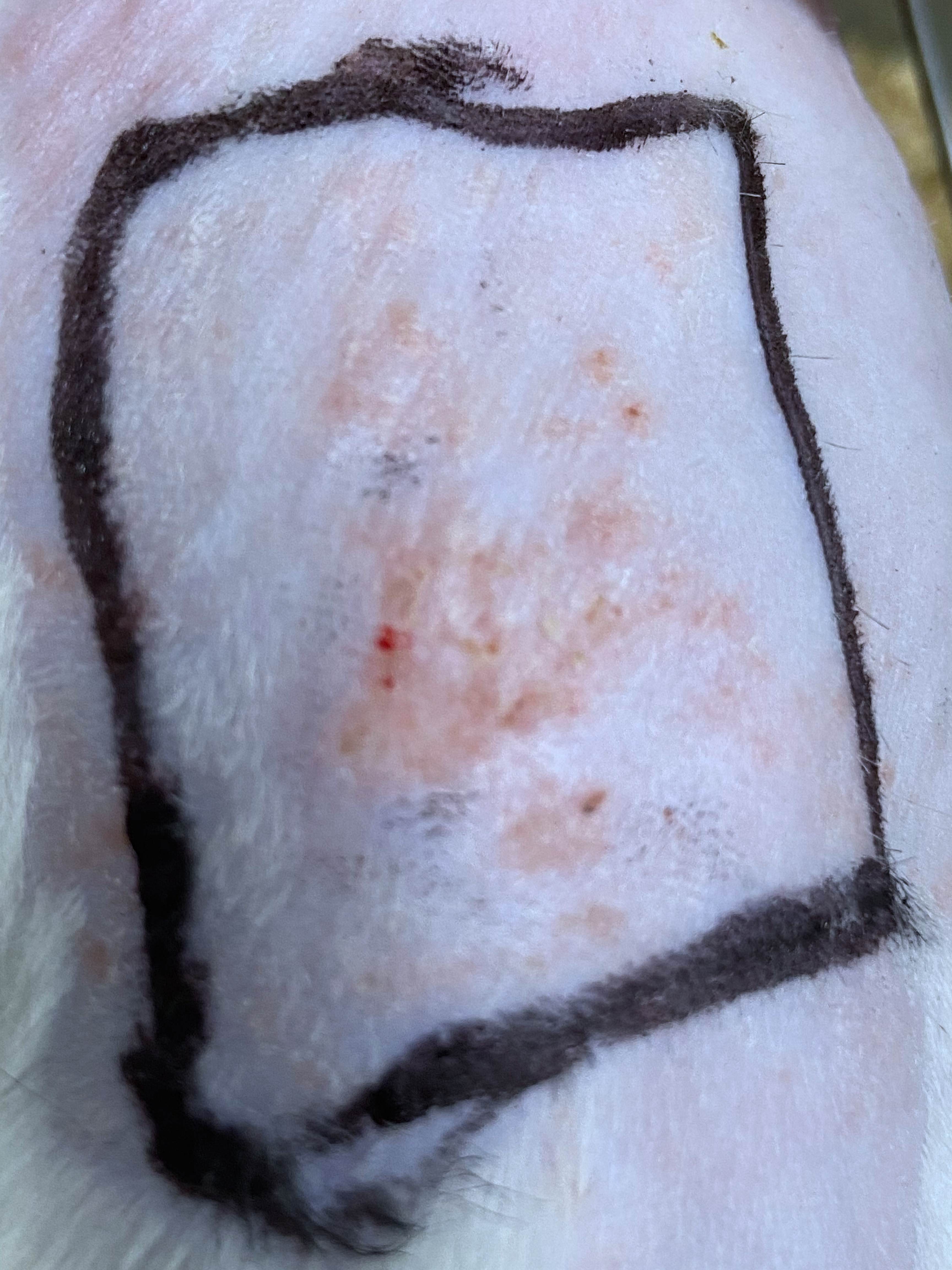

Supplement: S3 File — (ZIP) [file pone.0330078.s003.zip › Animal experiment/CGF/3d 1.jpg]

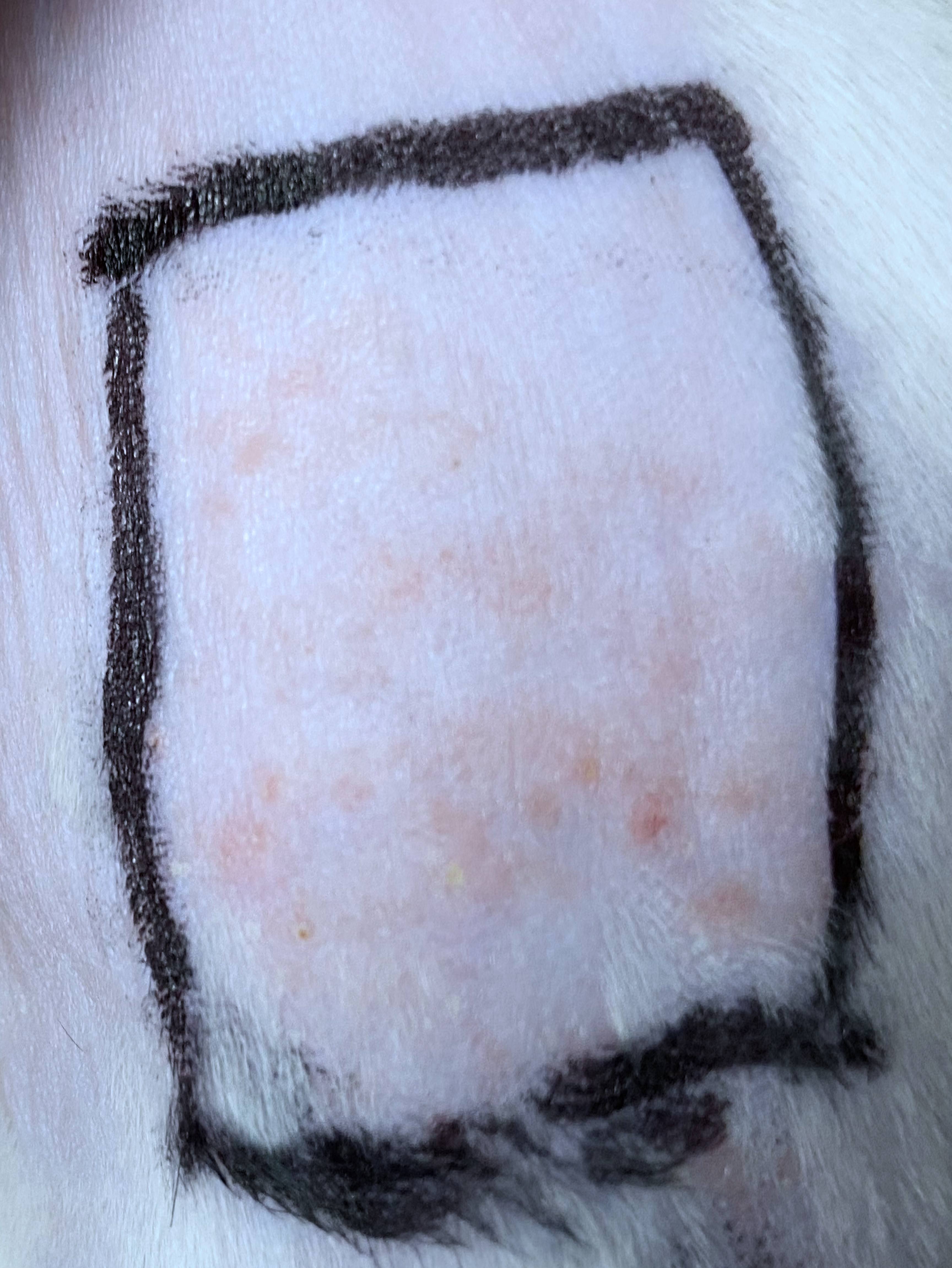

Supplement: S3 File — (ZIP) [file pone.0330078.s003.zip › Animal experiment/CGF/3d 2.jpg]

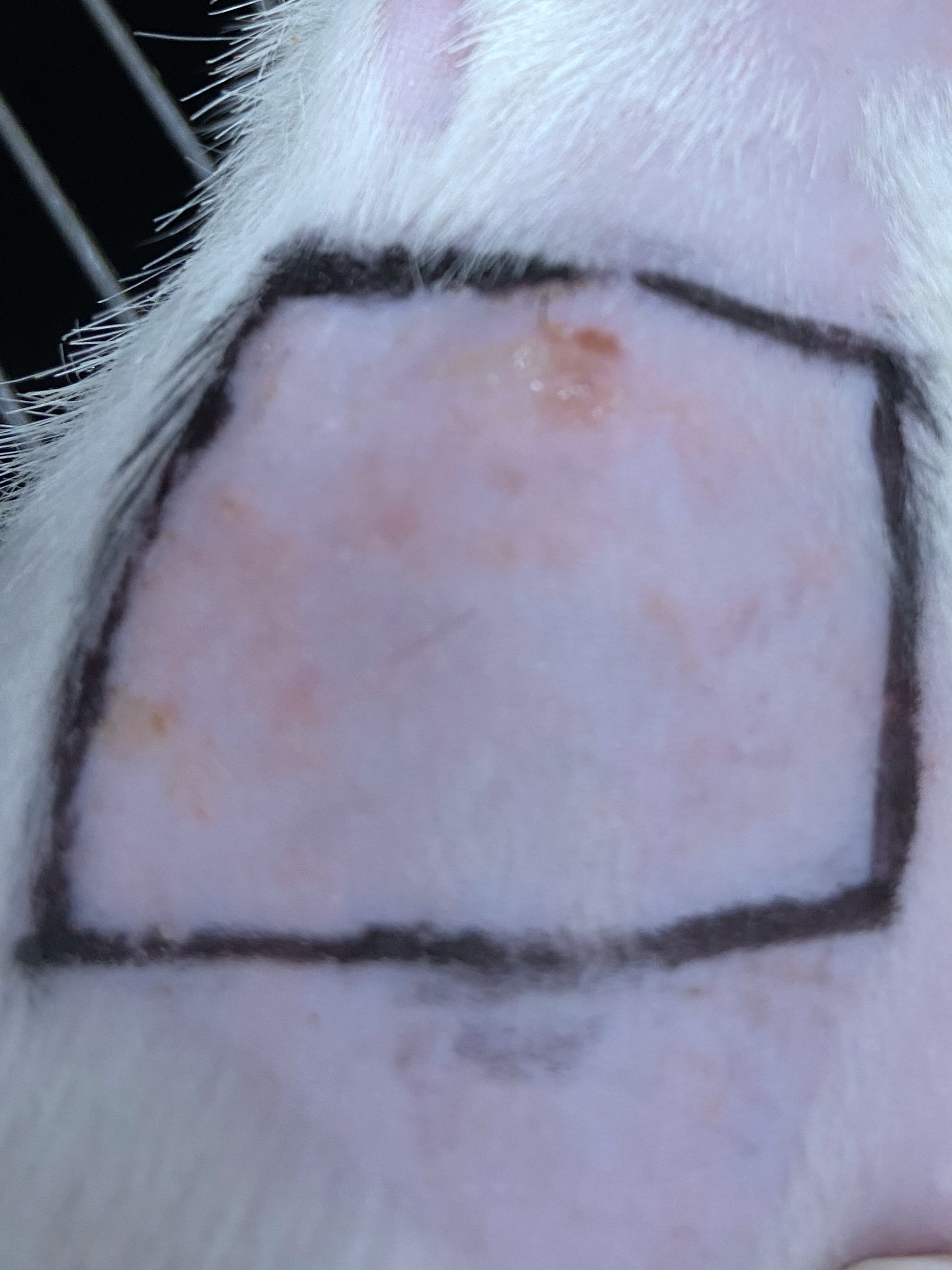

Supplement: S3 File — (ZIP) [file pone.0330078.s003.zip › Animal experiment/CGF/3d 3.jpg]

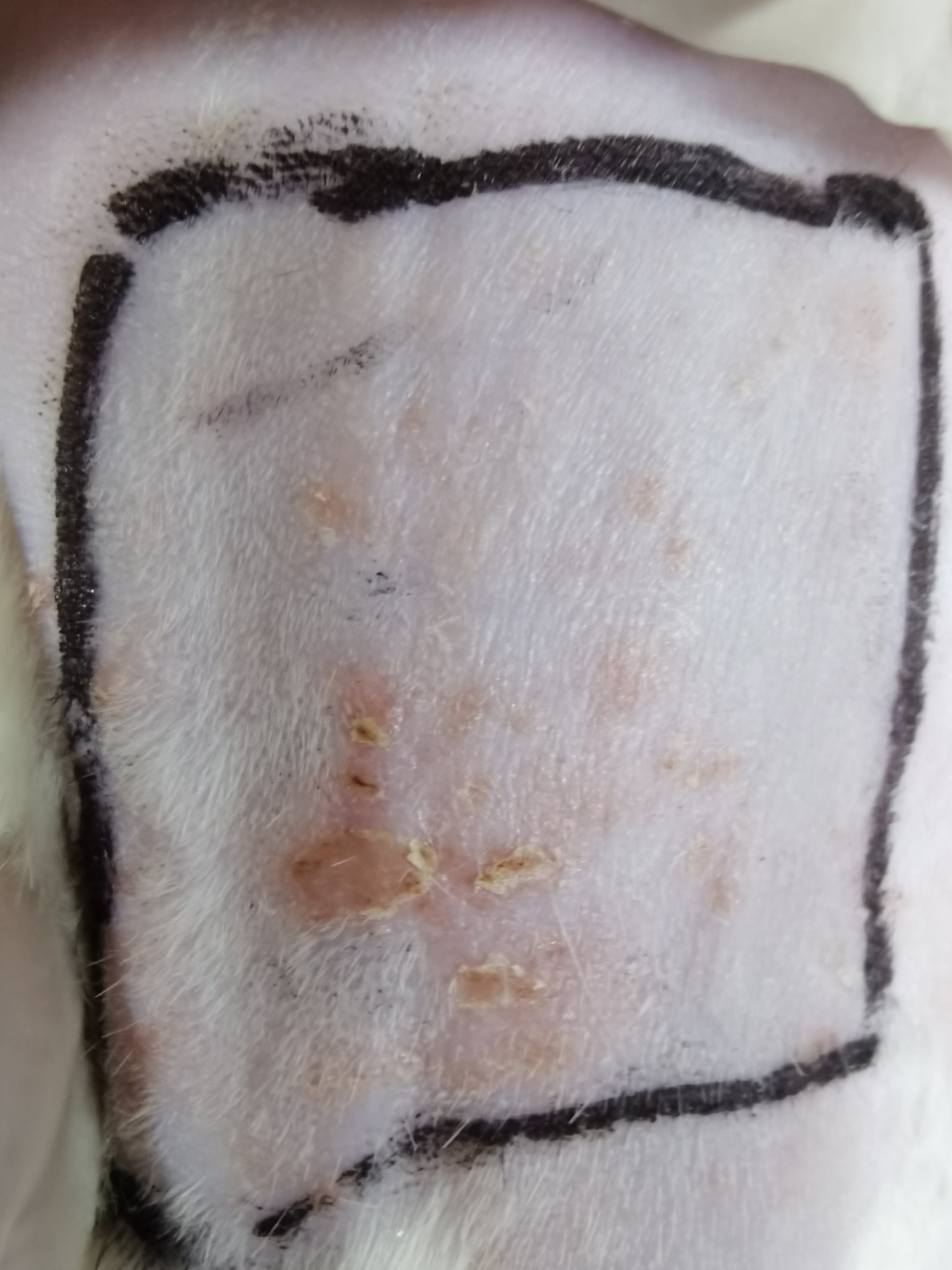

Supplement: S3 File — (ZIP) [file pone.0330078.s003.zip › Animal experiment/CGF/7d 1.jpg]

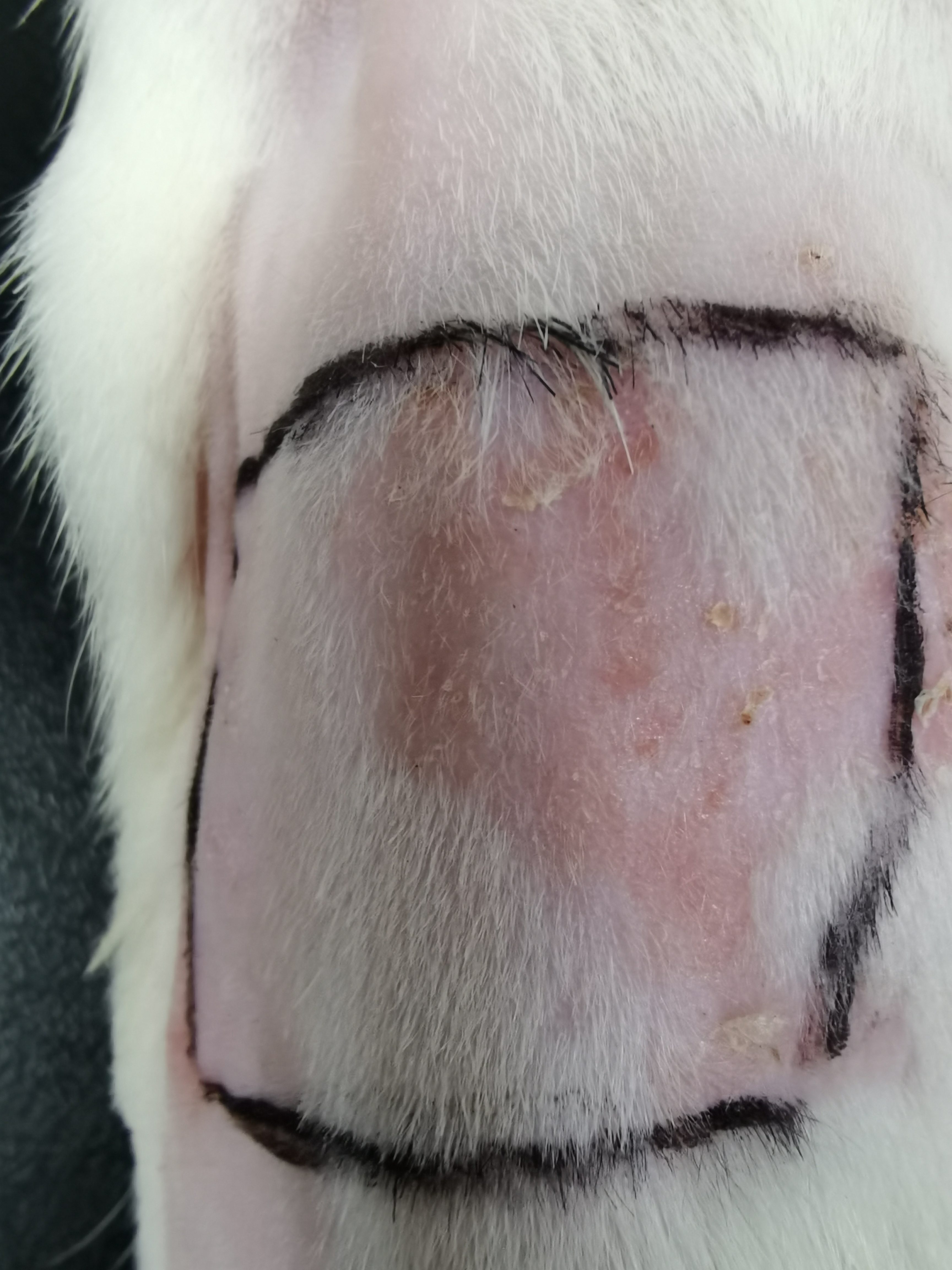

Supplement: S3 File — (ZIP) [file pone.0330078.s003.zip › Animal experiment/CGF/7d 2.jpg]

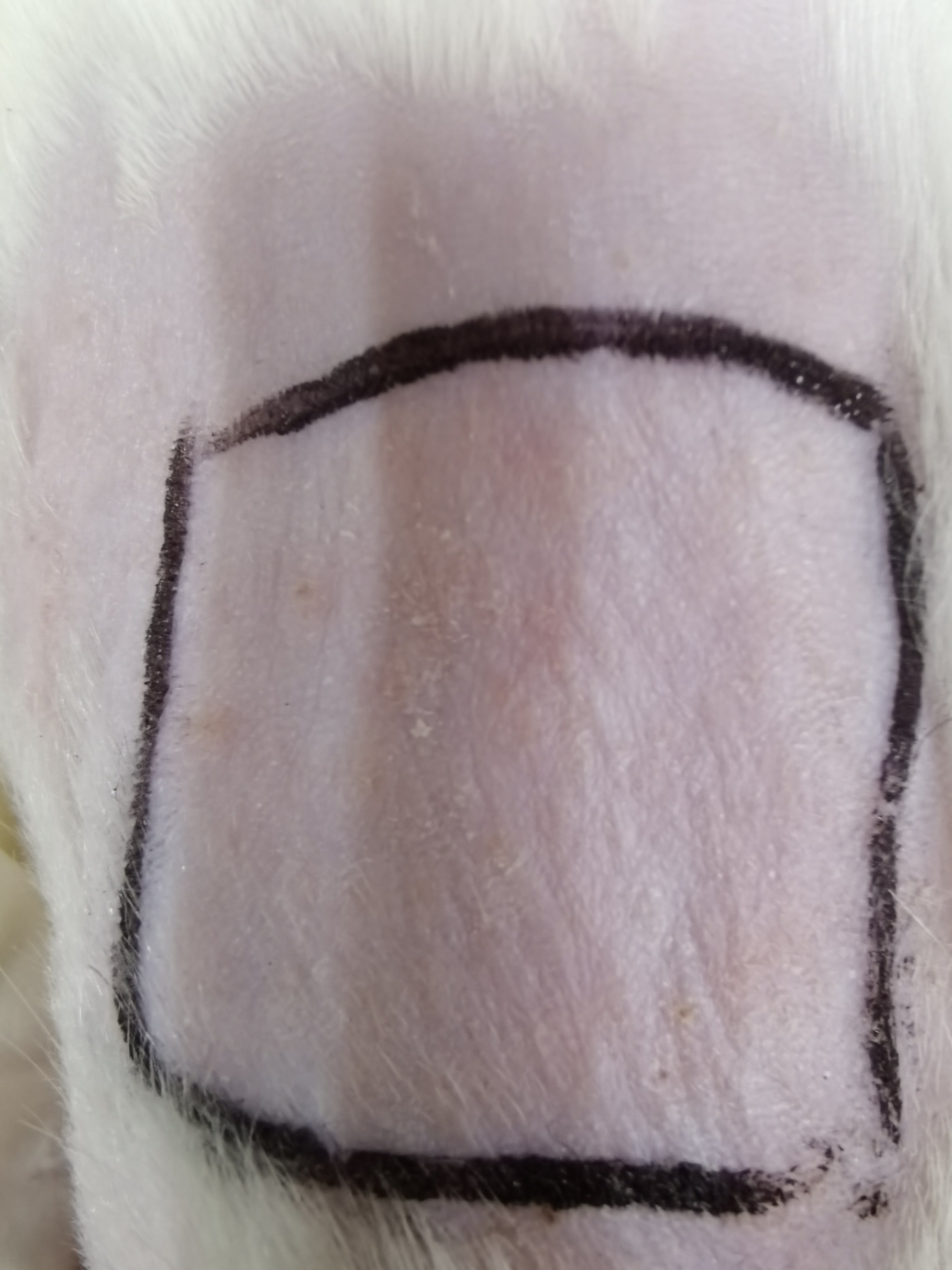

Supplement: S3 File — (ZIP) [file pone.0330078.s003.zip › Animal experiment/CGF/7d 3.jpg]

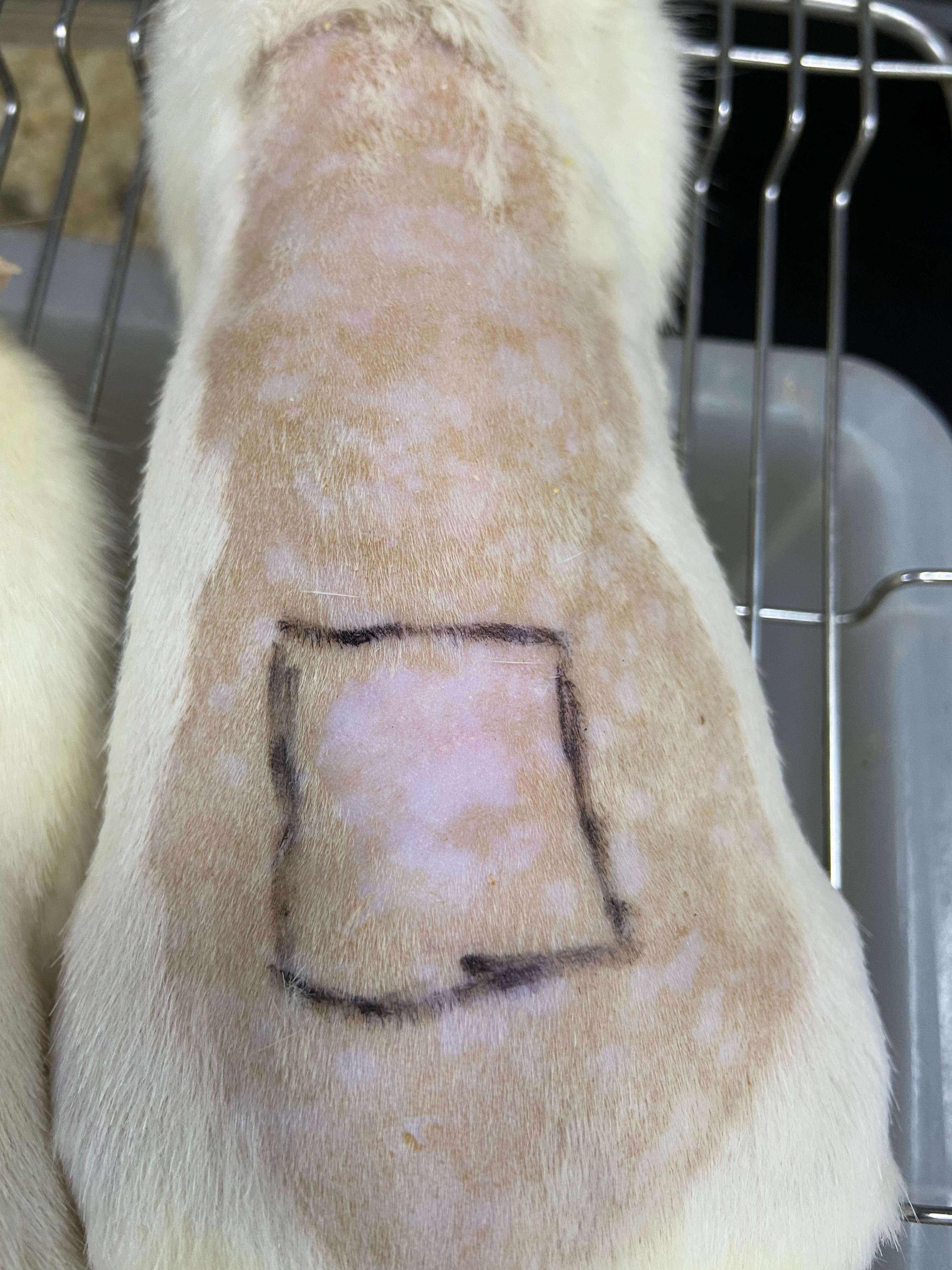

Supplement: S3 File — (ZIP) [file pone.0330078.s003.zip › Animal experiment/CGF+HAMCC/0d 1.jpg]

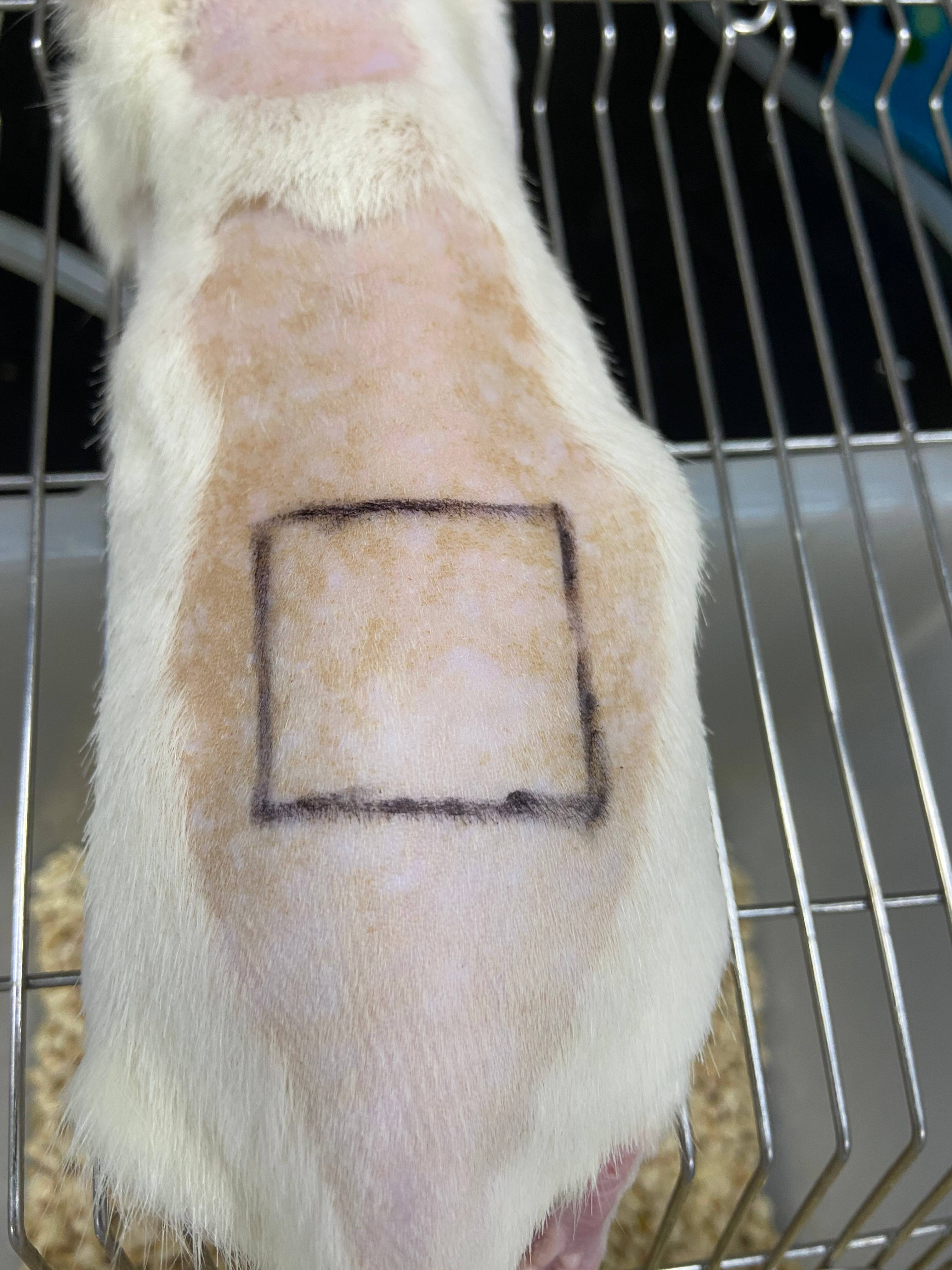

Supplement: S3 File — (ZIP) [file pone.0330078.s003.zip › Animal experiment/CGF+HAMCC/0d 2.jpg]

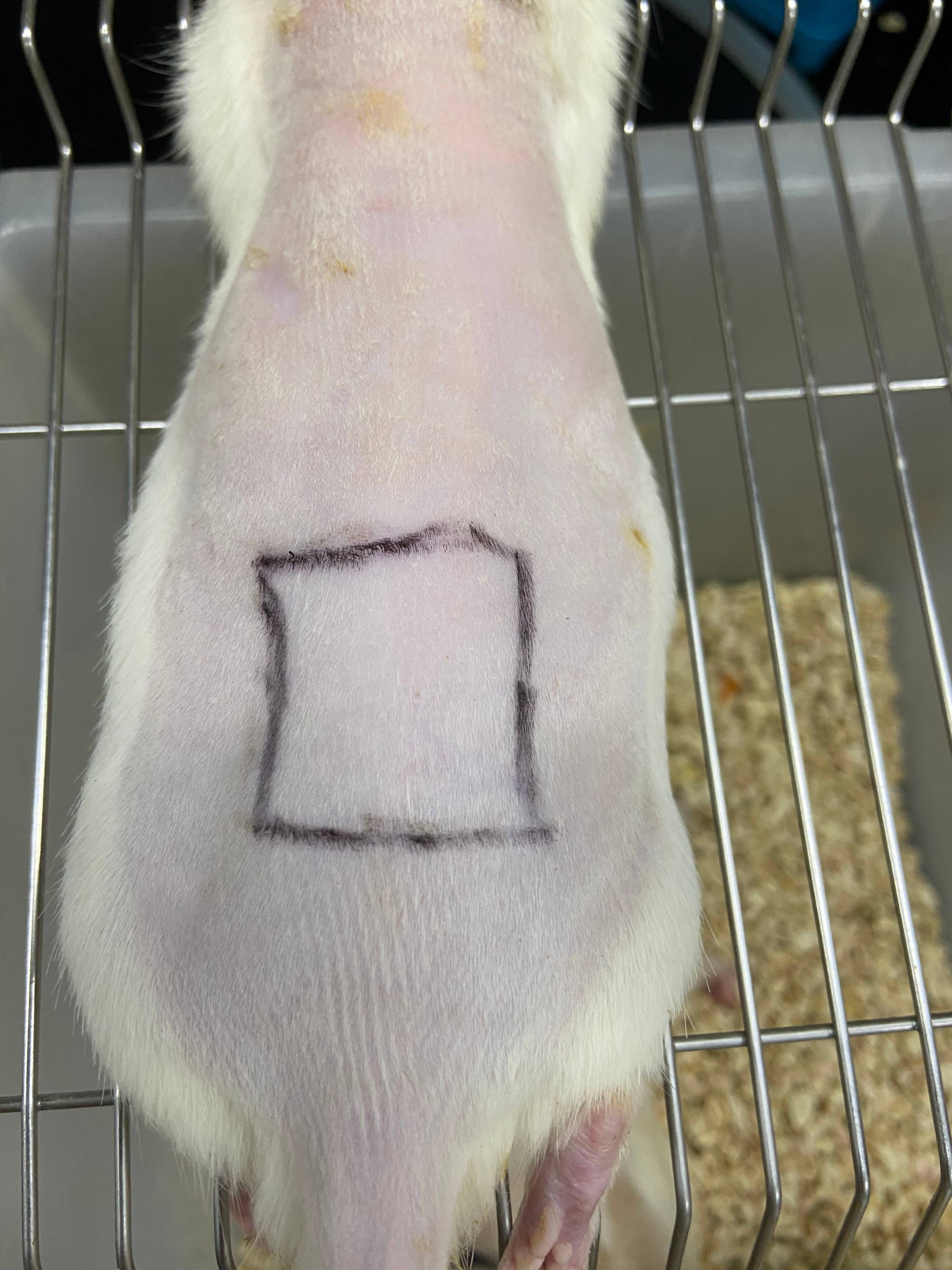

Supplement: S3 File — (ZIP) [file pone.0330078.s003.zip › Animal experiment/CGF+HAMCC/0d 3.jpg]

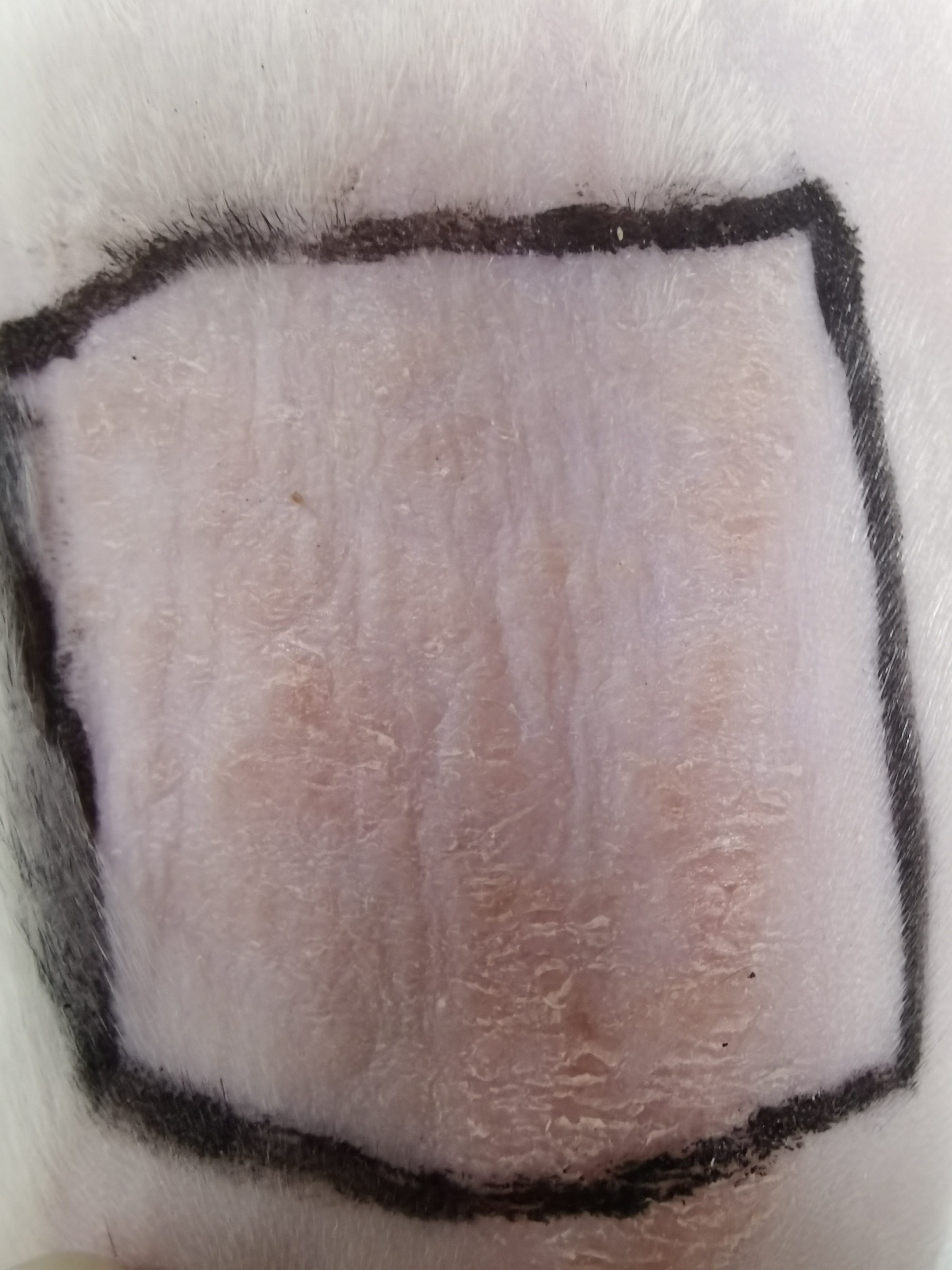

Supplement: S3 File — (ZIP) [file pone.0330078.s003.zip › Animal experiment/CGF+HAMCC/14d 1.jpg]

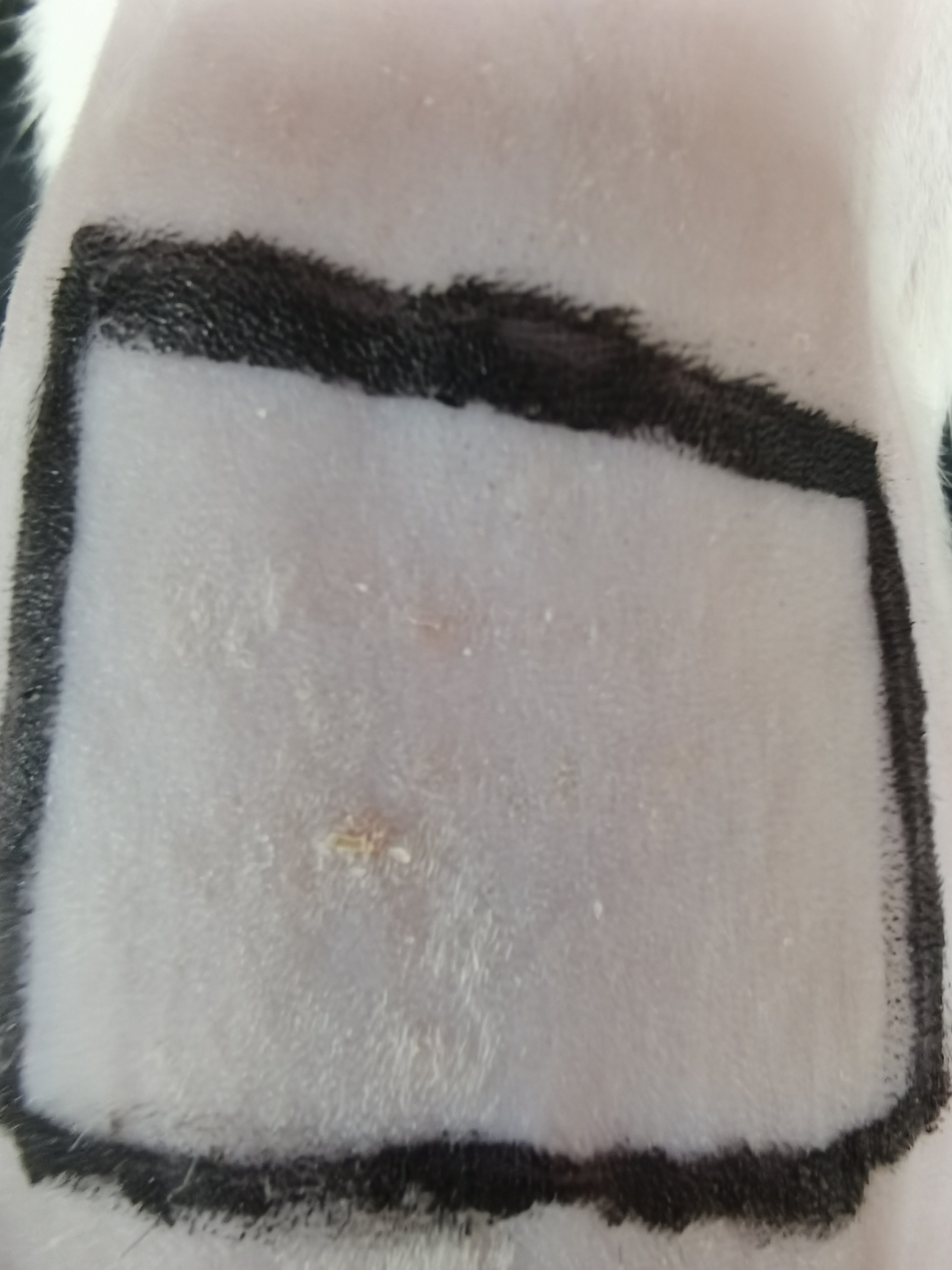

Supplement: S3 File — (ZIP) [file pone.0330078.s003.zip › Animal experiment/CGF+HAMCC/14d 2.jpg]

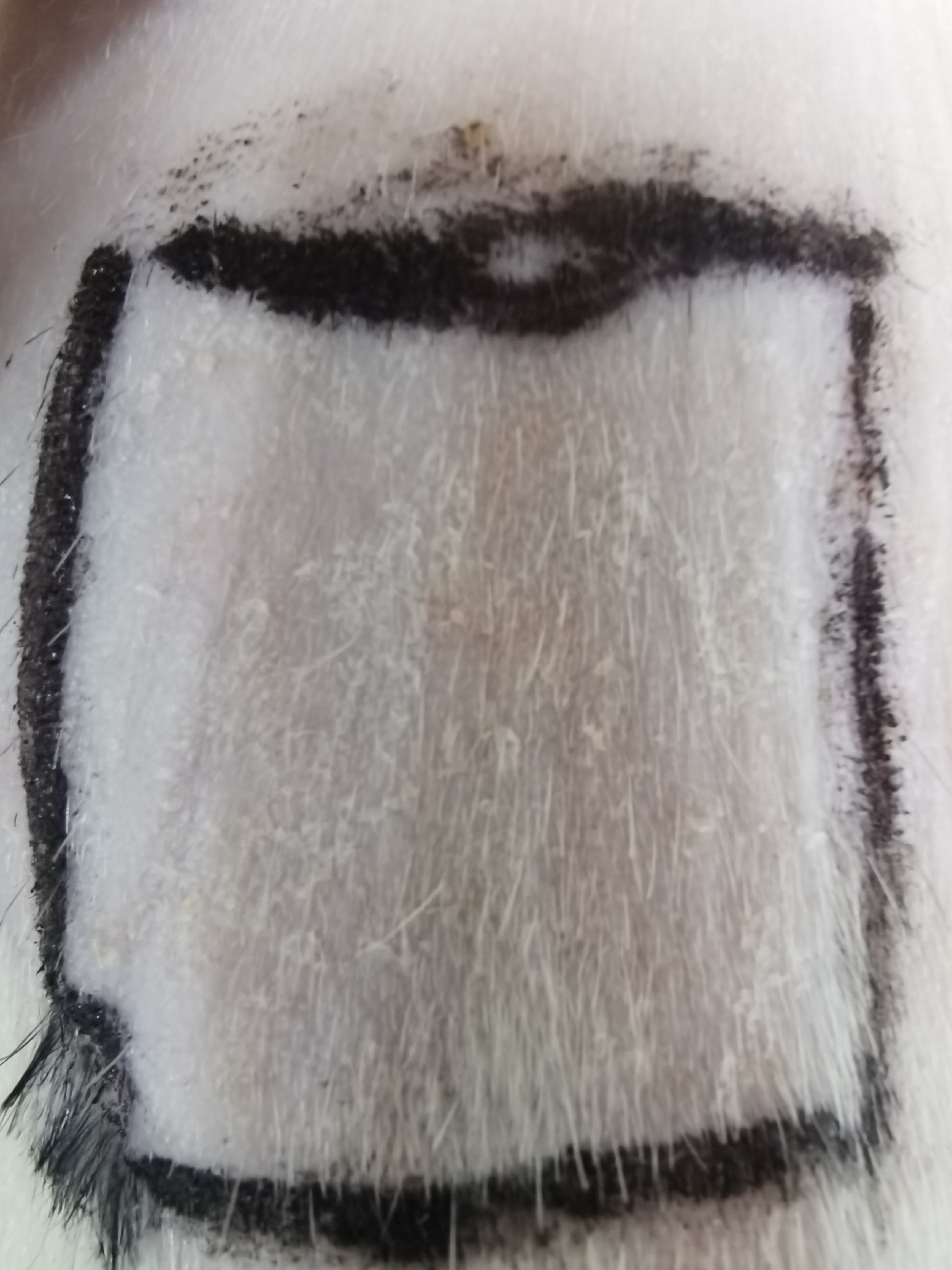

Supplement: S3 File — (ZIP) [file pone.0330078.s003.zip › Animal experiment/CGF+HAMCC/14d 3.jpg]

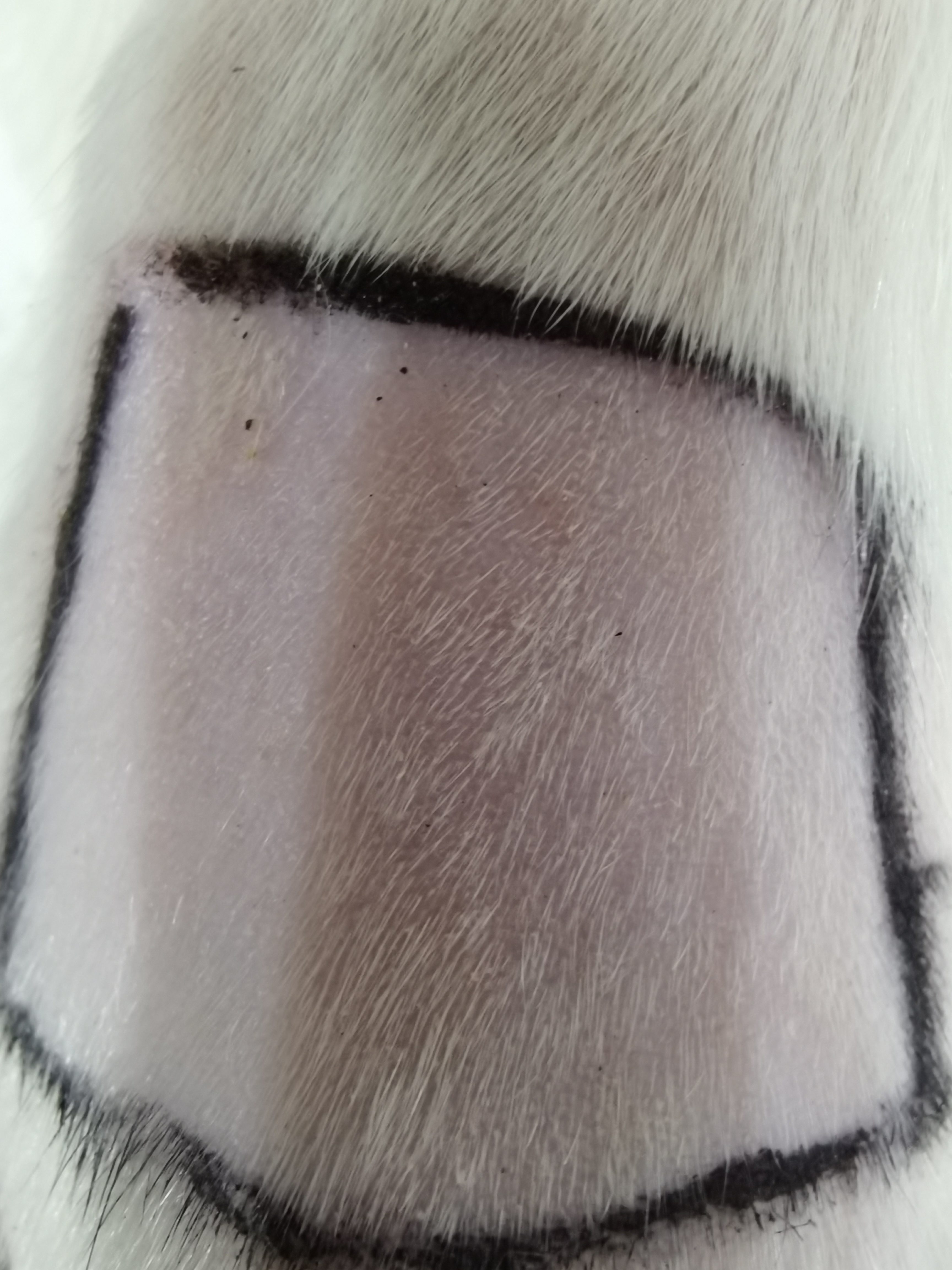

Supplement: S3 File — (ZIP) [file pone.0330078.s003.zip › Animal experiment/CGF+HAMCC/21d 1.jpg]

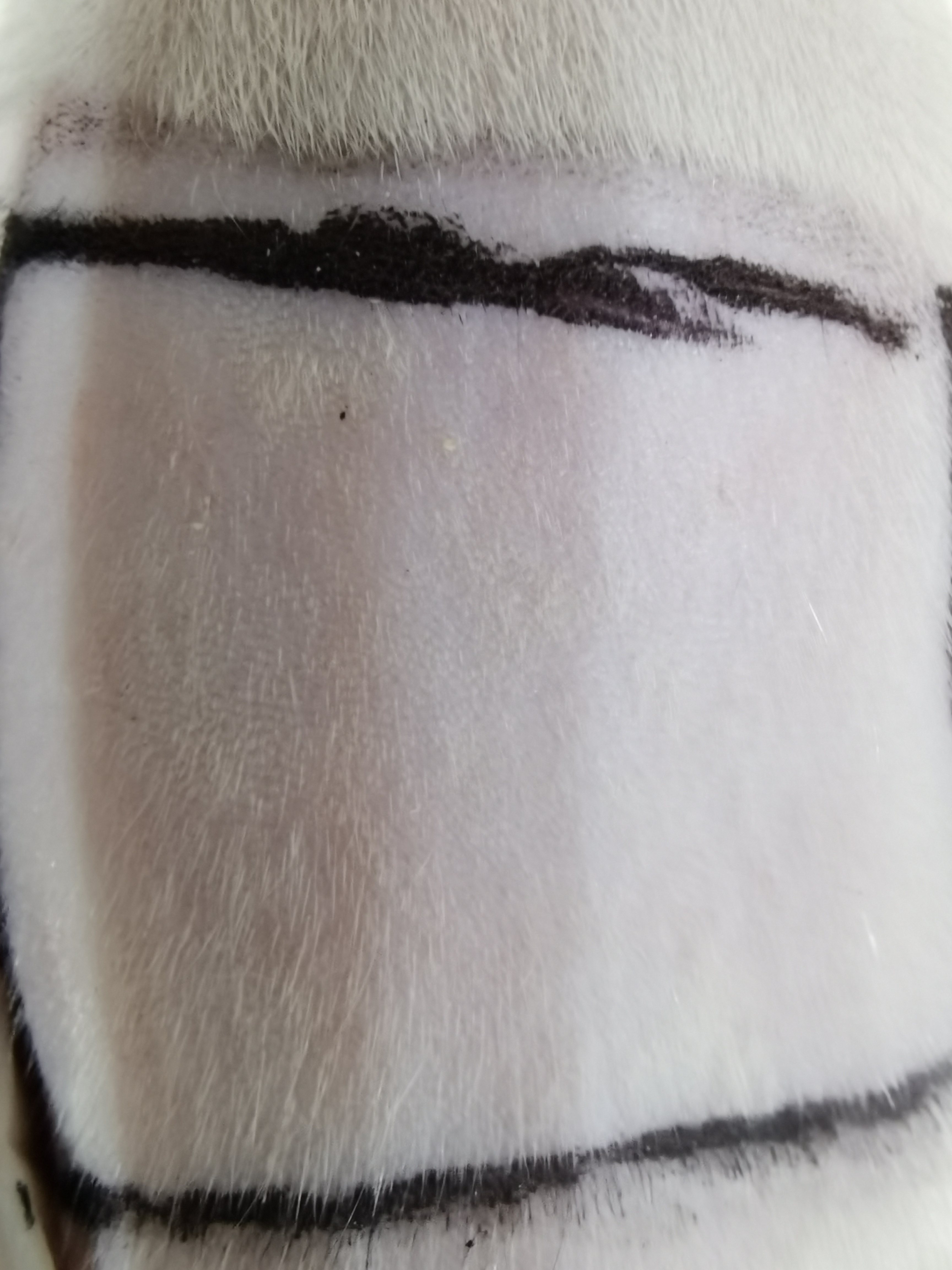

Supplement: S3 File — (ZIP) [file pone.0330078.s003.zip › Animal experiment/CGF+HAMCC/21d 2.jpg]

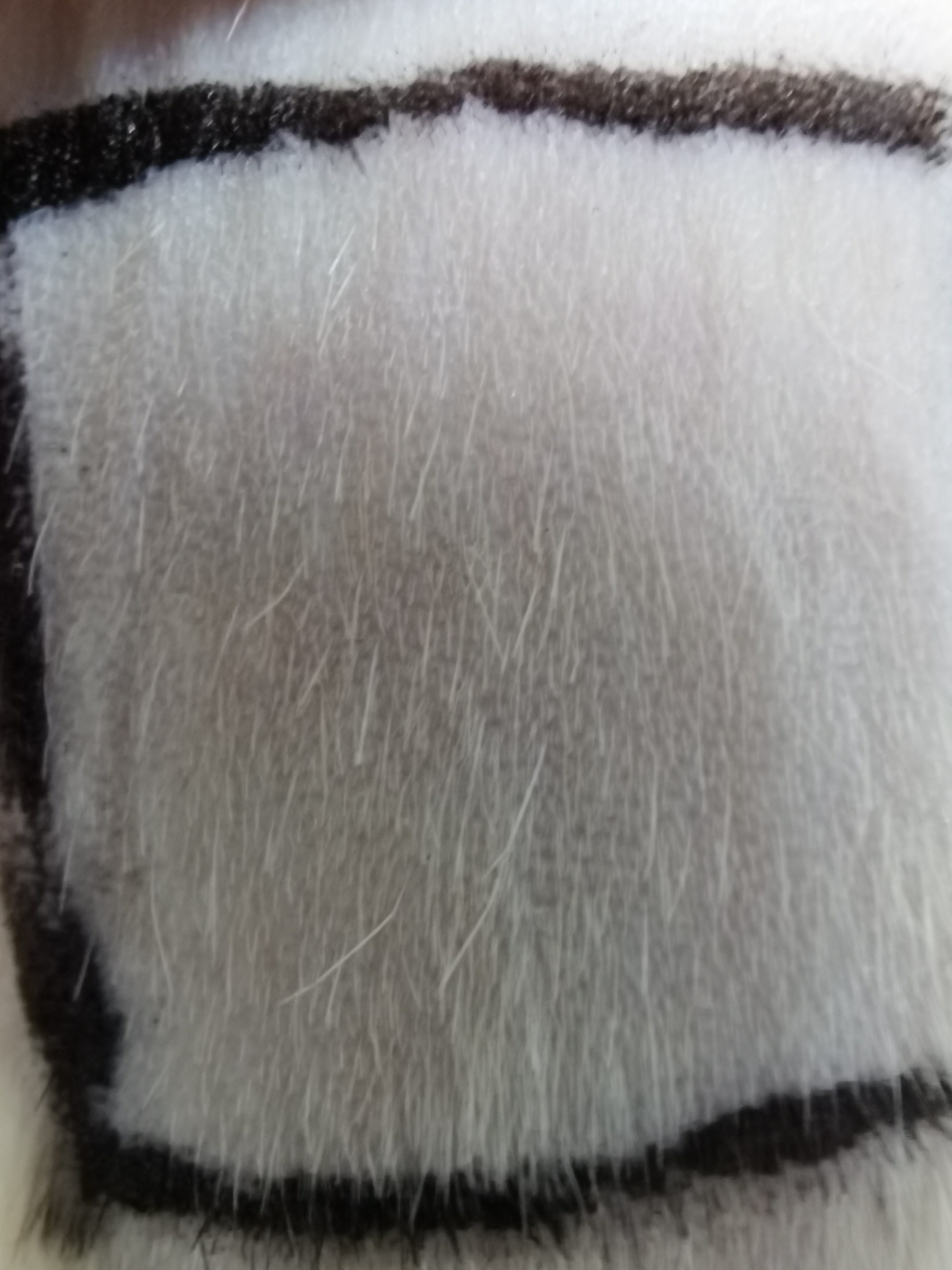

Supplement: S3 File — (ZIP) [file pone.0330078.s003.zip › Animal experiment/CGF+HAMCC/21d 3.jpg]

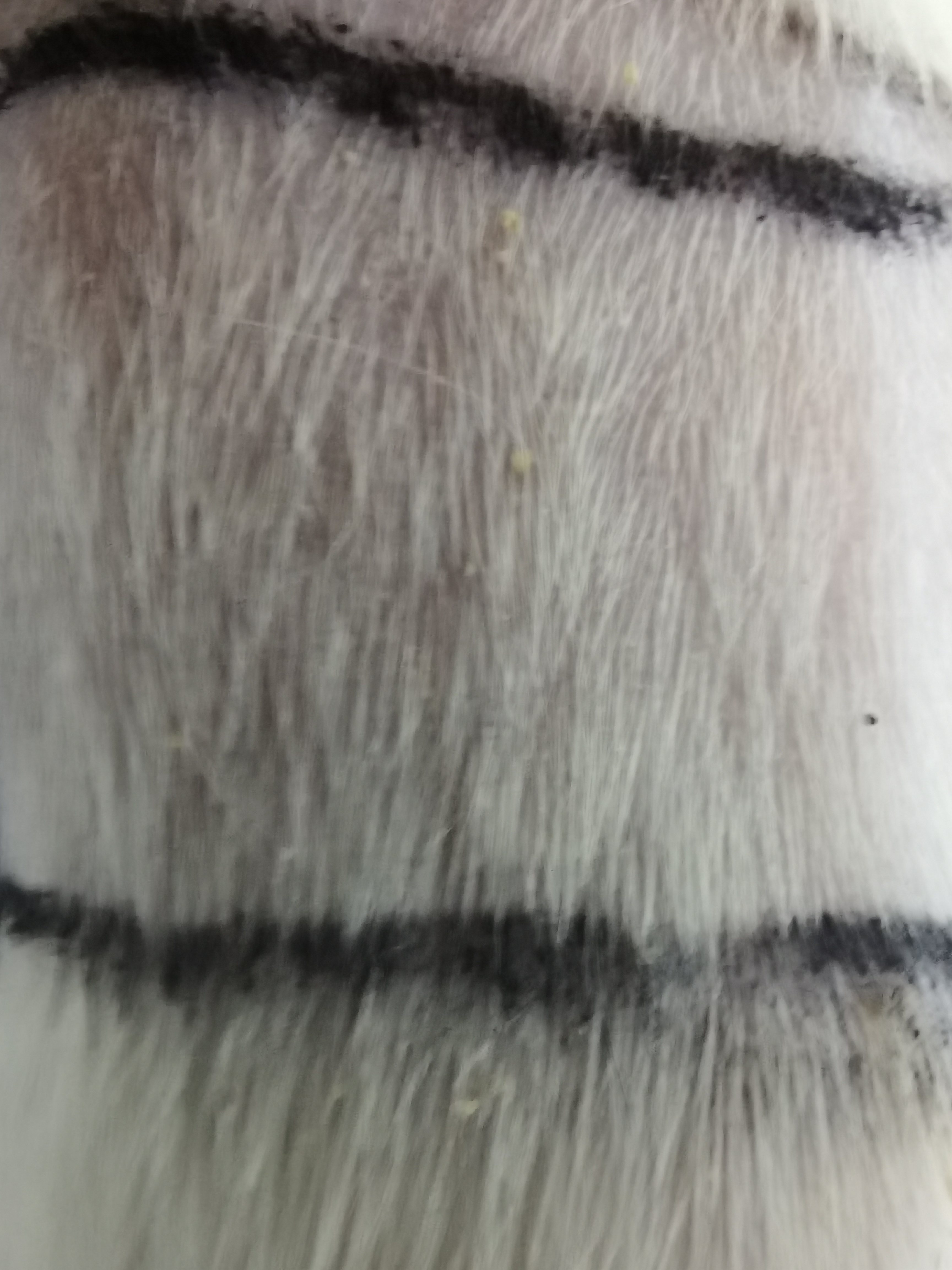

Supplement: S3 File — (ZIP) [file pone.0330078.s003.zip › Animal experiment/CGF+HAMCC/28d 1.jpg]

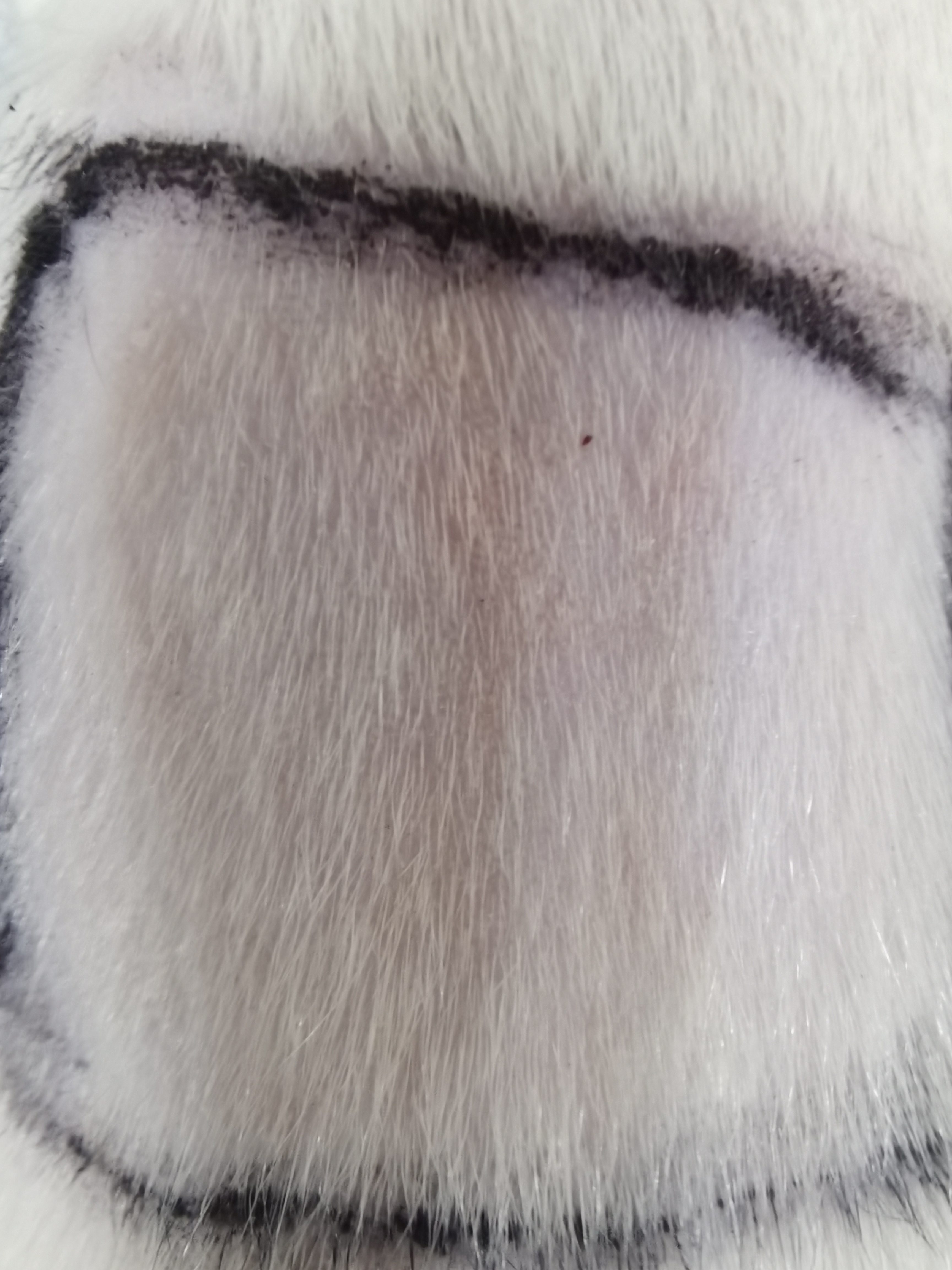

Supplement: S3 File — (ZIP) [file pone.0330078.s003.zip › Animal experiment/CGF+HAMCC/28d 2.jpg]

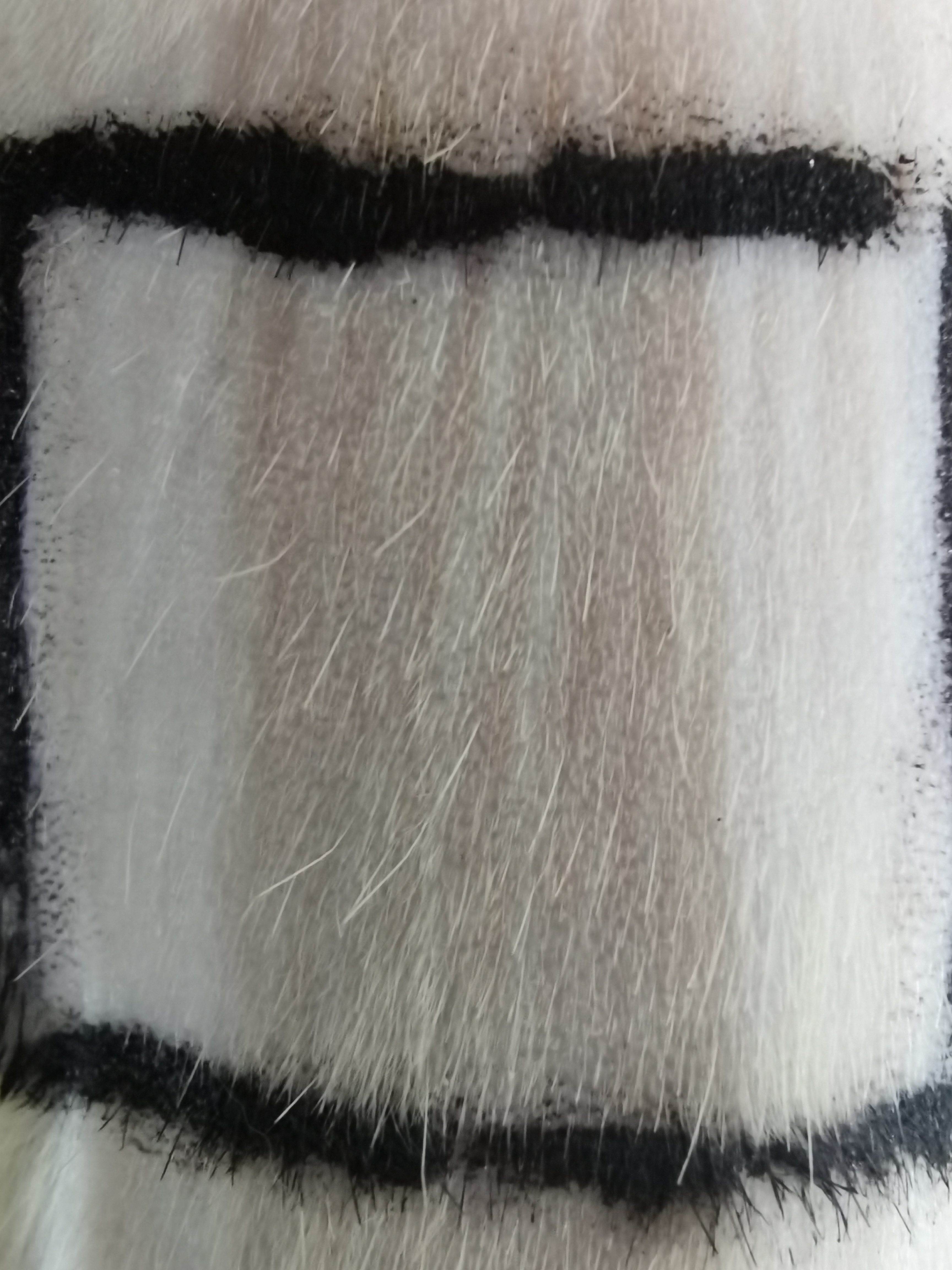

Supplement: S3 File — (ZIP) [file pone.0330078.s003.zip › Animal experiment/CGF+HAMCC/28d 3.jpg]

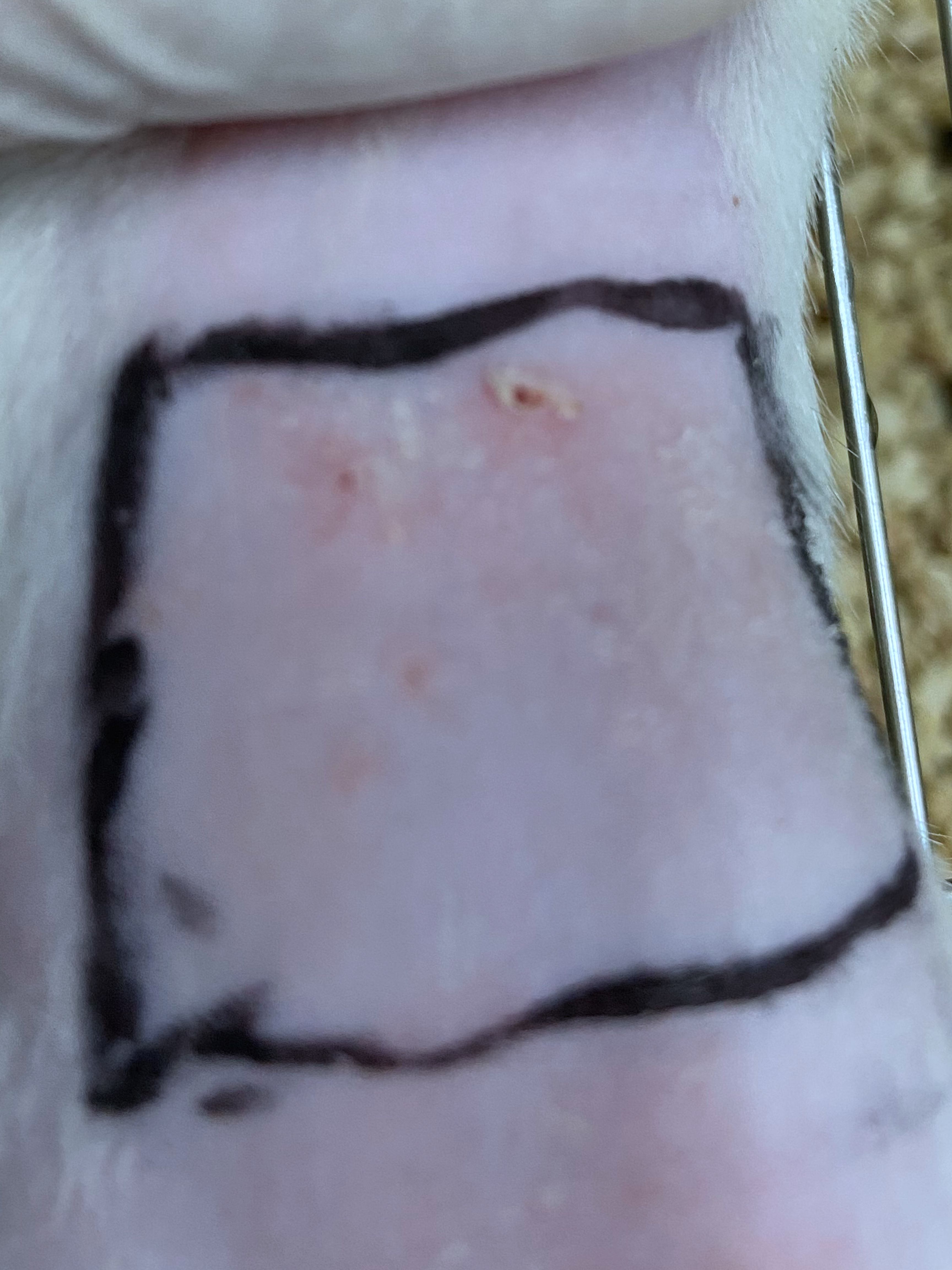

Supplement: S3 File — (ZIP) [file pone.0330078.s003.zip › Animal experiment/CGF+HAMCC/3d 1.jpg]

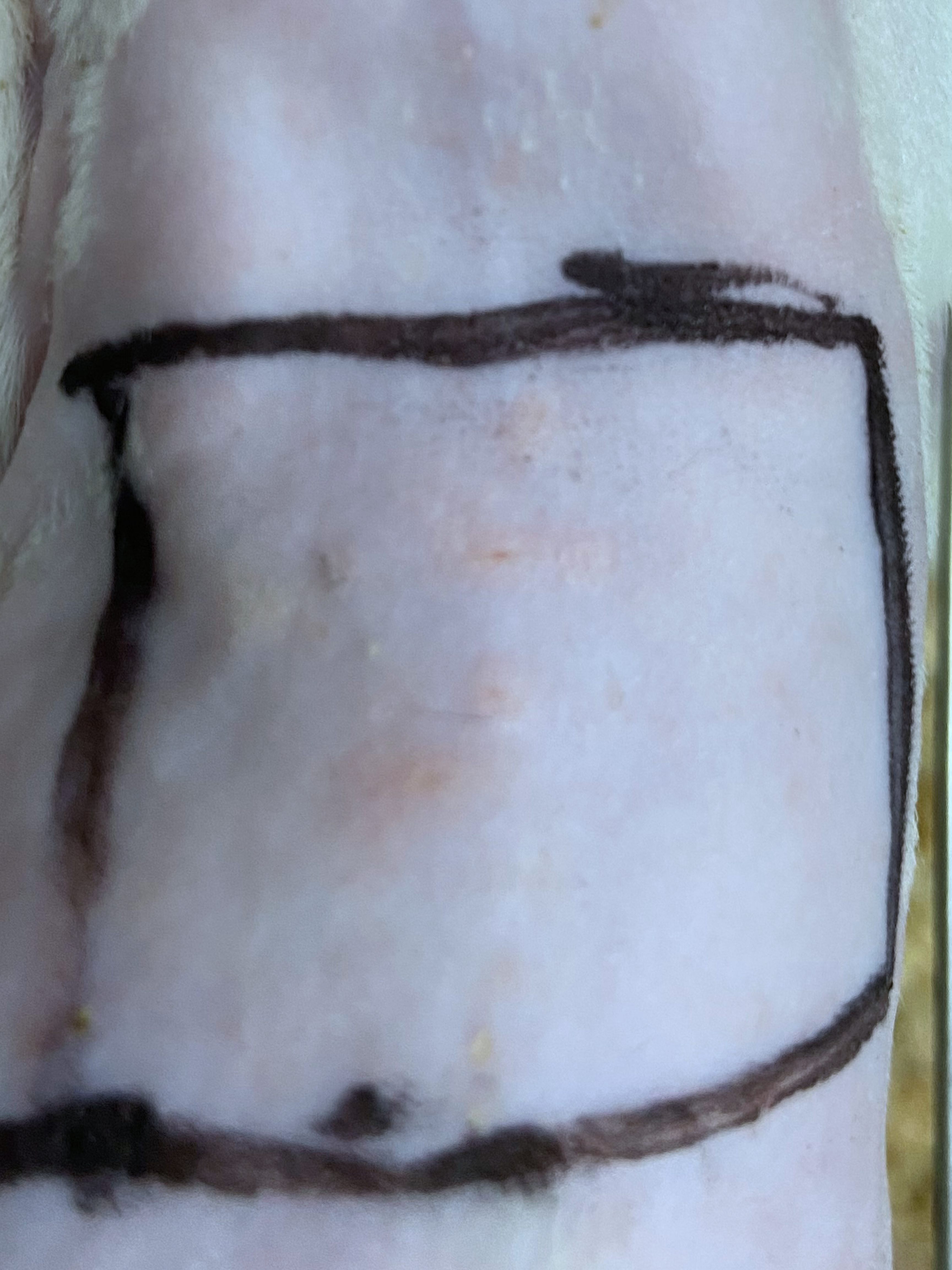

Supplement: S3 File — (ZIP) [file pone.0330078.s003.zip › Animal experiment/CGF+HAMCC/3d 2.jpg]

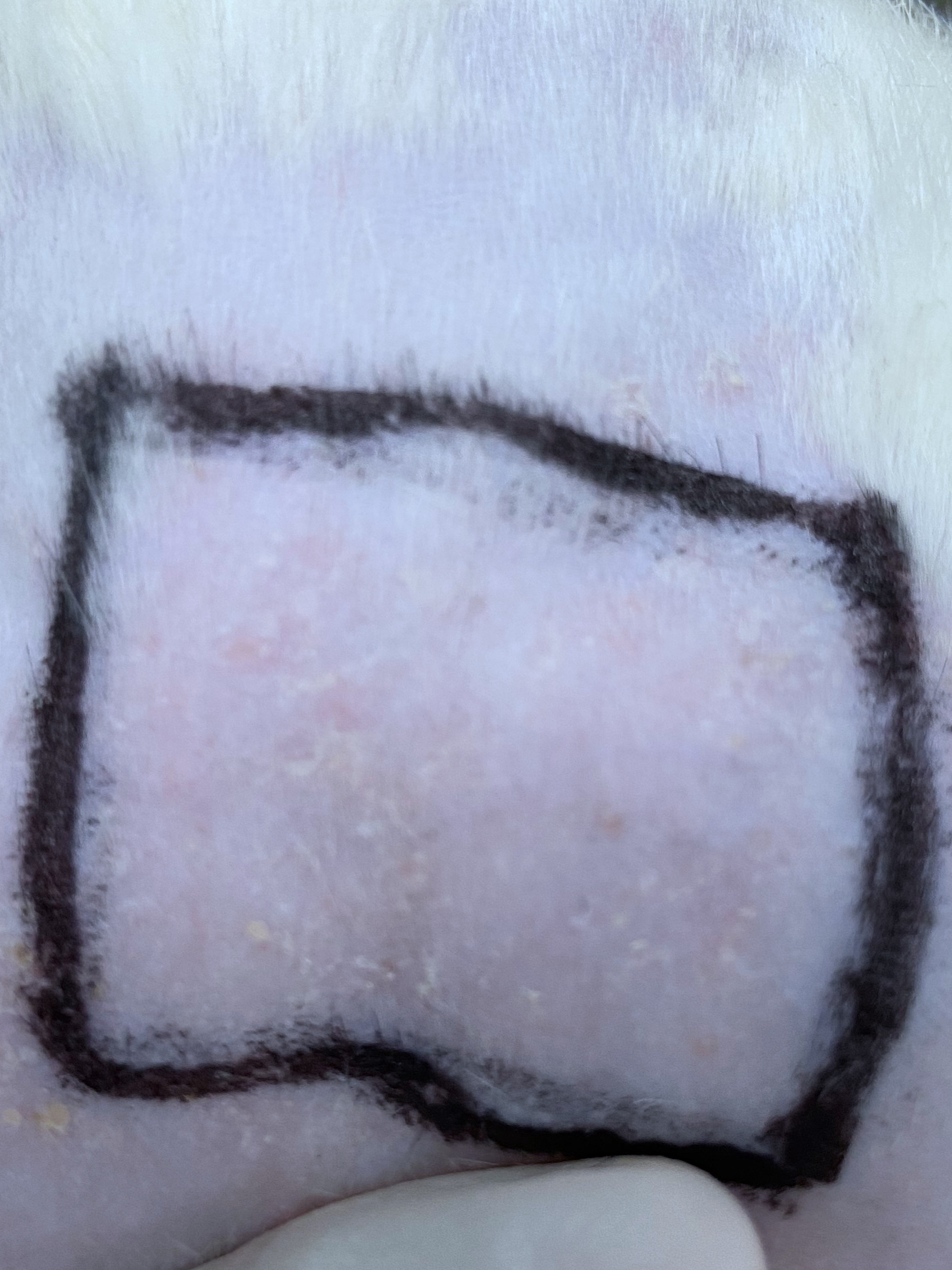

Supplement: S3 File — (ZIP) [file pone.0330078.s003.zip › Animal experiment/CGF+HAMCC/3d 3.jpg]

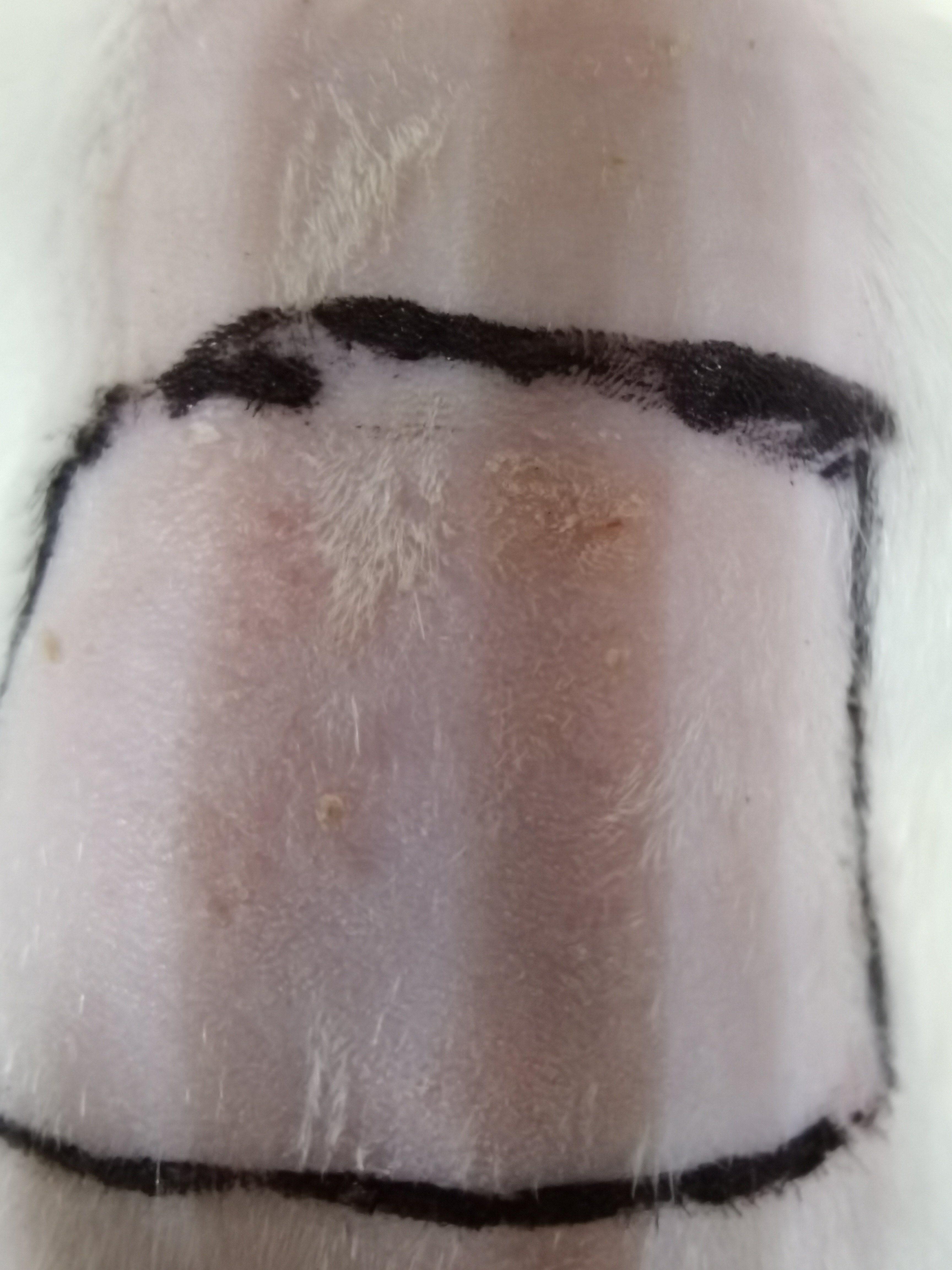

Supplement: S3 File — (ZIP) [file pone.0330078.s003.zip › Animal experiment/CGF+HAMCC/7d 1.jpg]

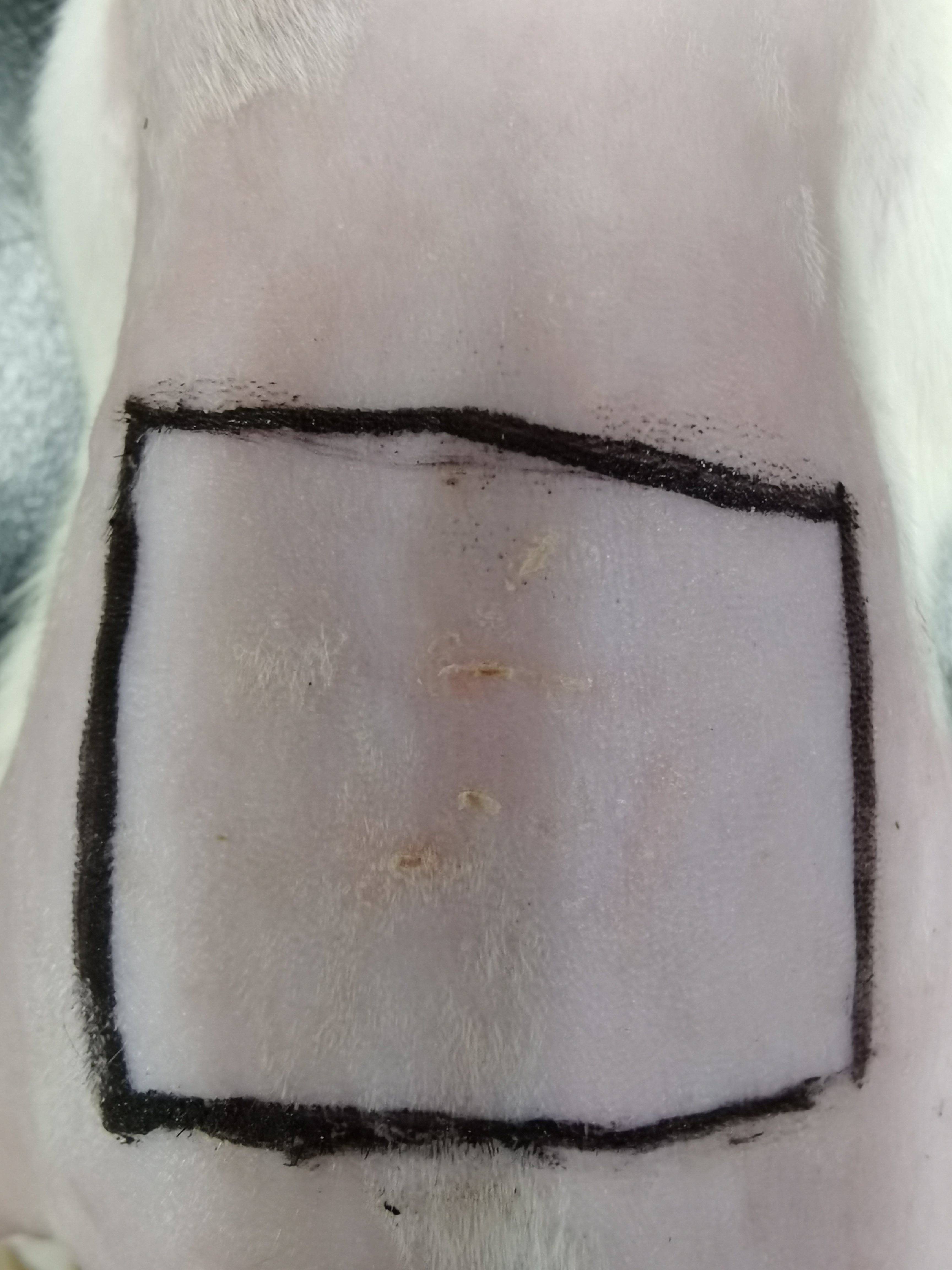

Supplement: S3 File — (ZIP) [file pone.0330078.s003.zip › Animal experiment/CGF+HAMCC/7d 2.jpg]

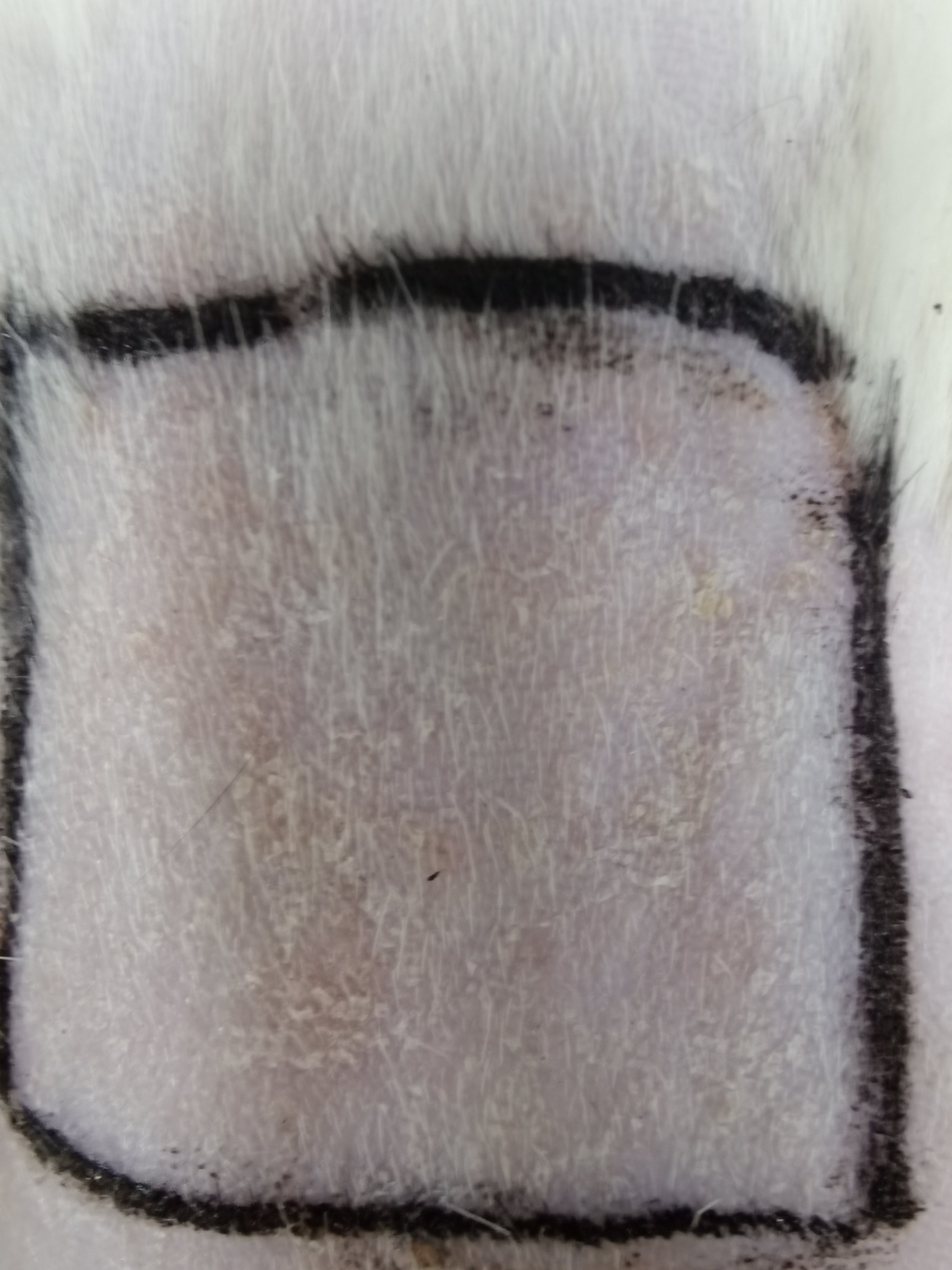

Supplement: S3 File — (ZIP) [file pone.0330078.s003.zip › Animal experiment/CGF+HAMCC/7d 3.jpg]

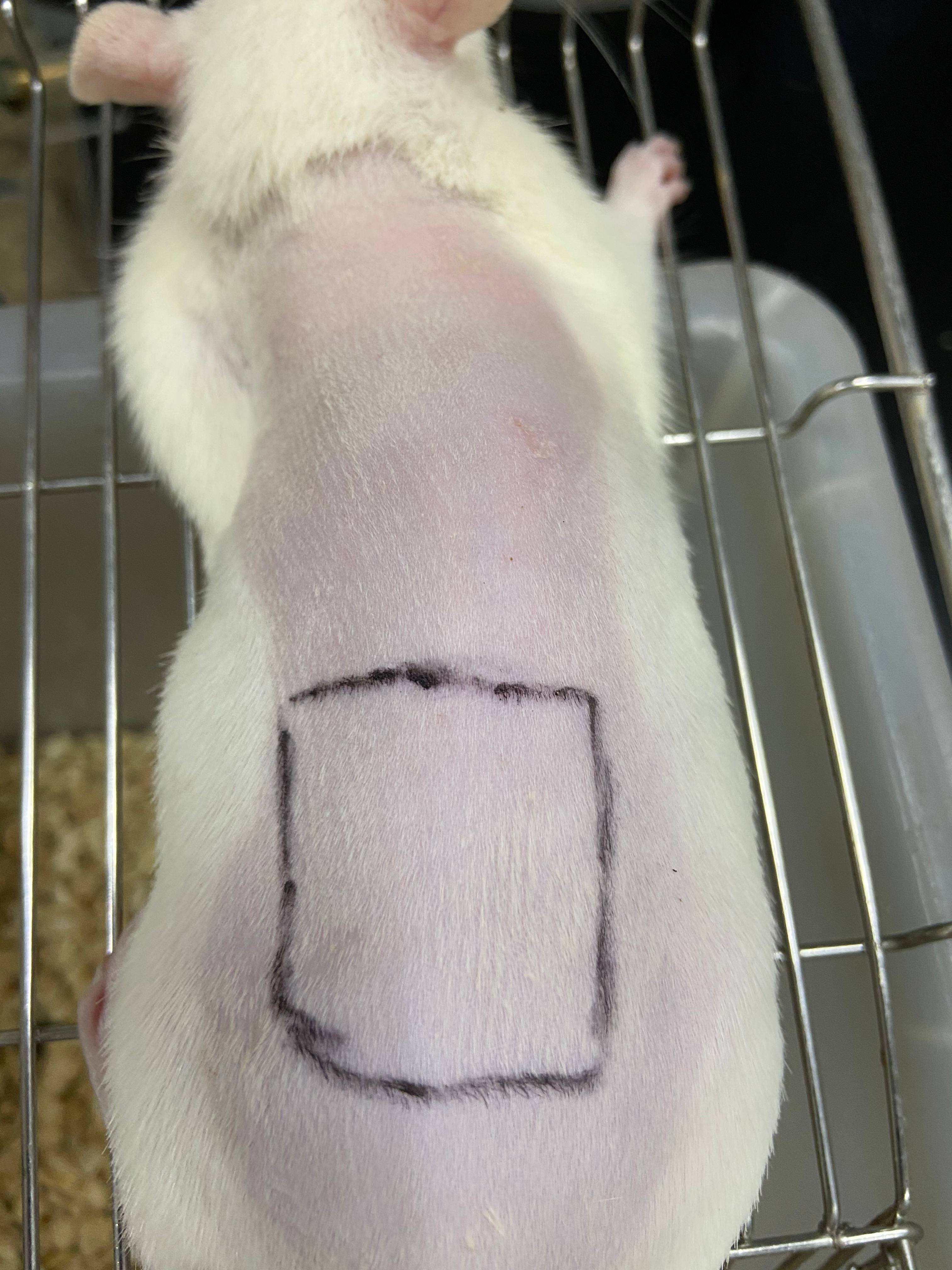

Supplement: S3 File — (ZIP) [file pone.0330078.s003.zip › Animal experiment/Control/0d 1.jpg]

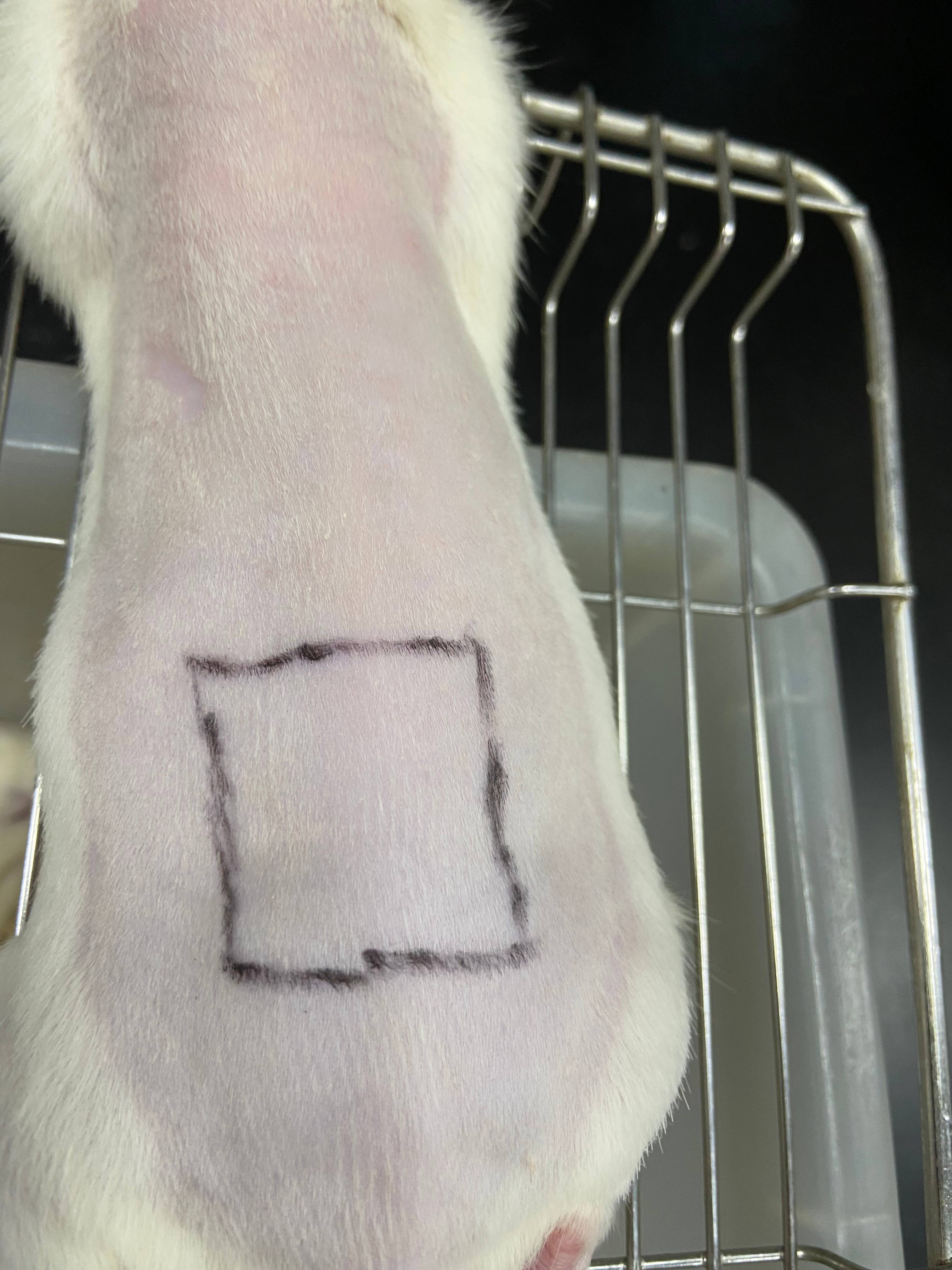

Supplement: S3 File — (ZIP) [file pone.0330078.s003.zip › Animal experiment/Control/0d 2.jpg]

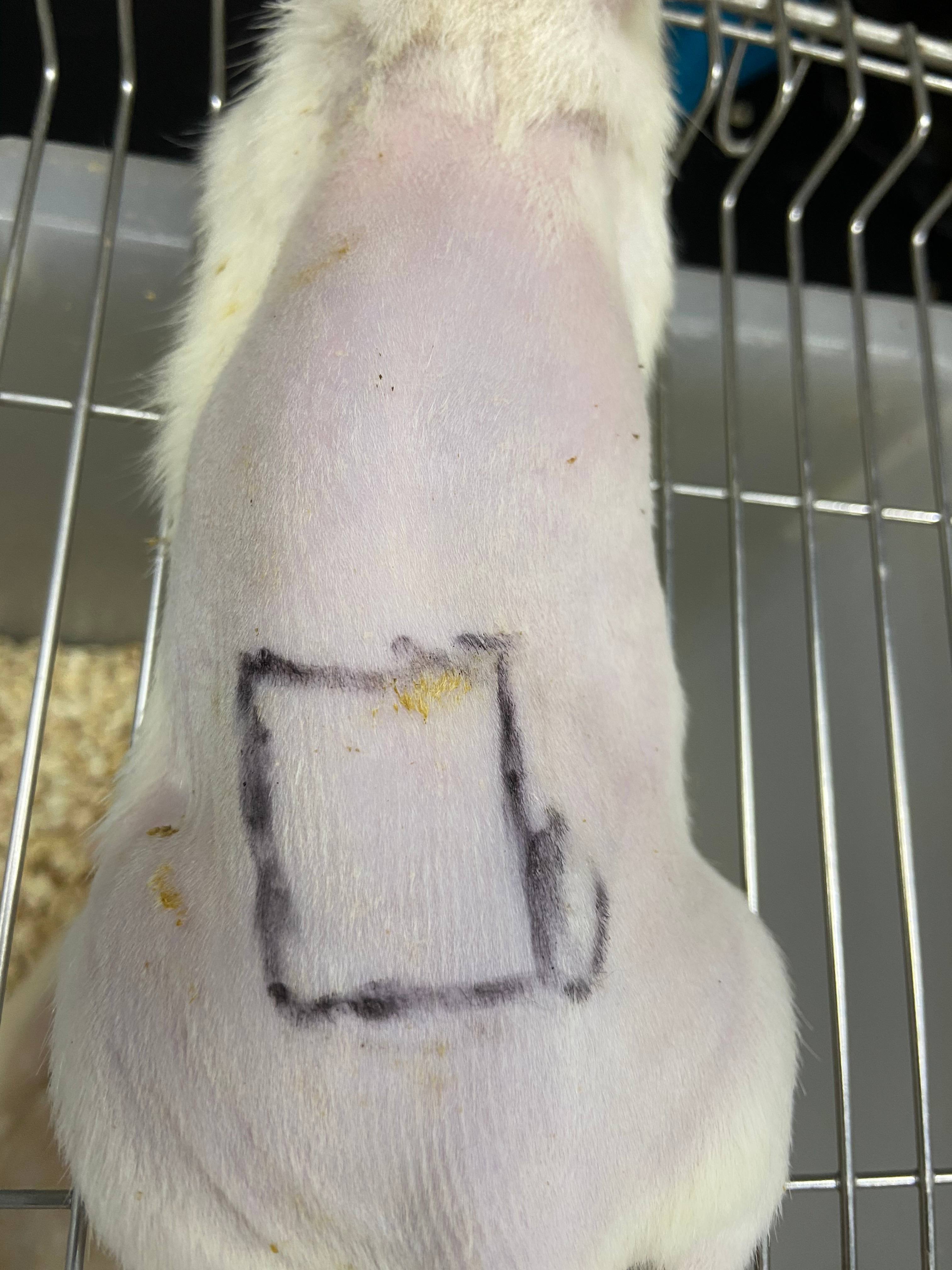

Supplement: S3 File — (ZIP) [file pone.0330078.s003.zip › Animal experiment/Control/0d 3.jpg]

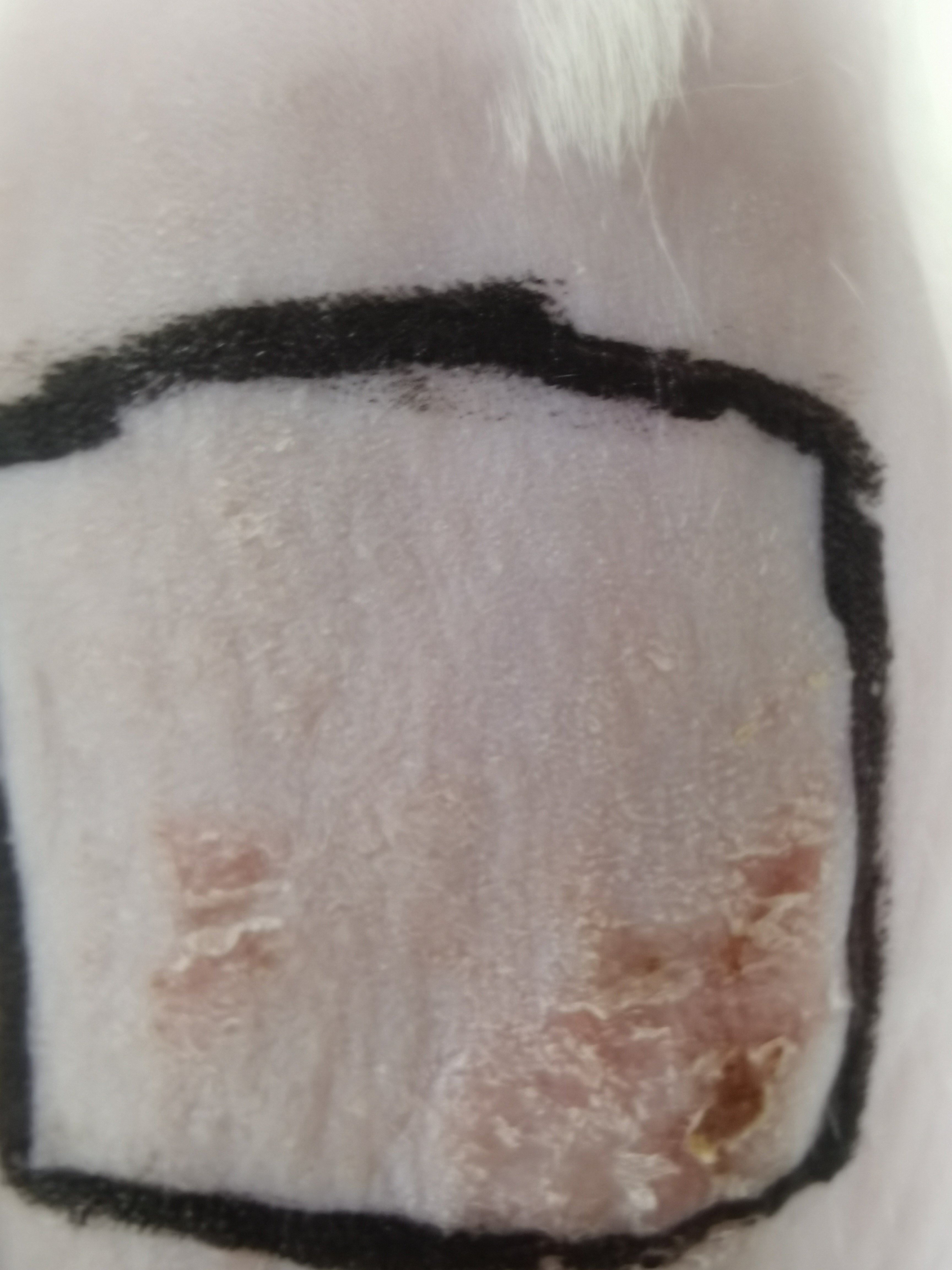

Supplement: S3 File — (ZIP) [file pone.0330078.s003.zip › Animal experiment/Control/14d 1.jpg]

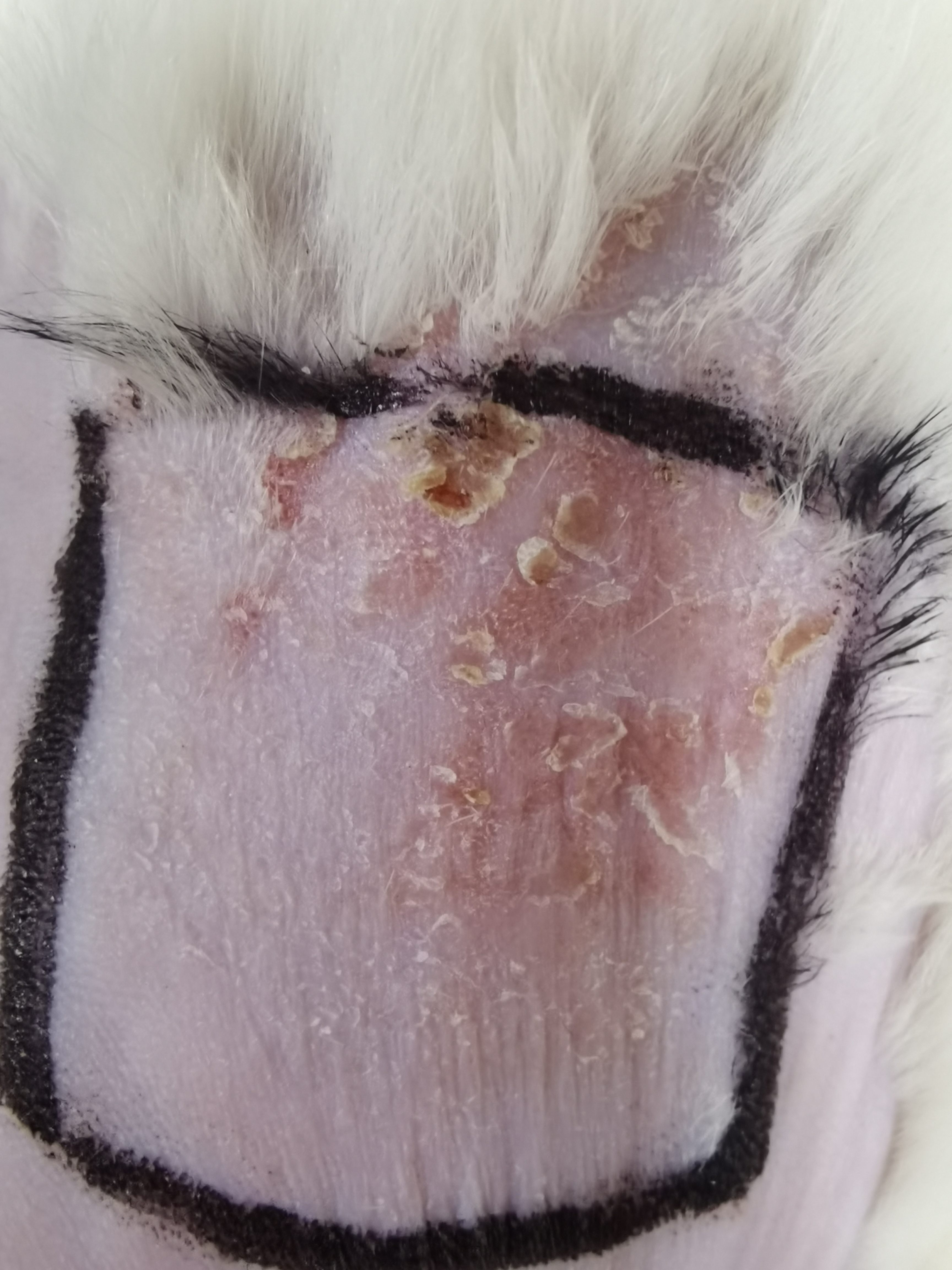

Supplement: S3 File — (ZIP) [file pone.0330078.s003.zip › Animal experiment/Control/14d 2.jpg]

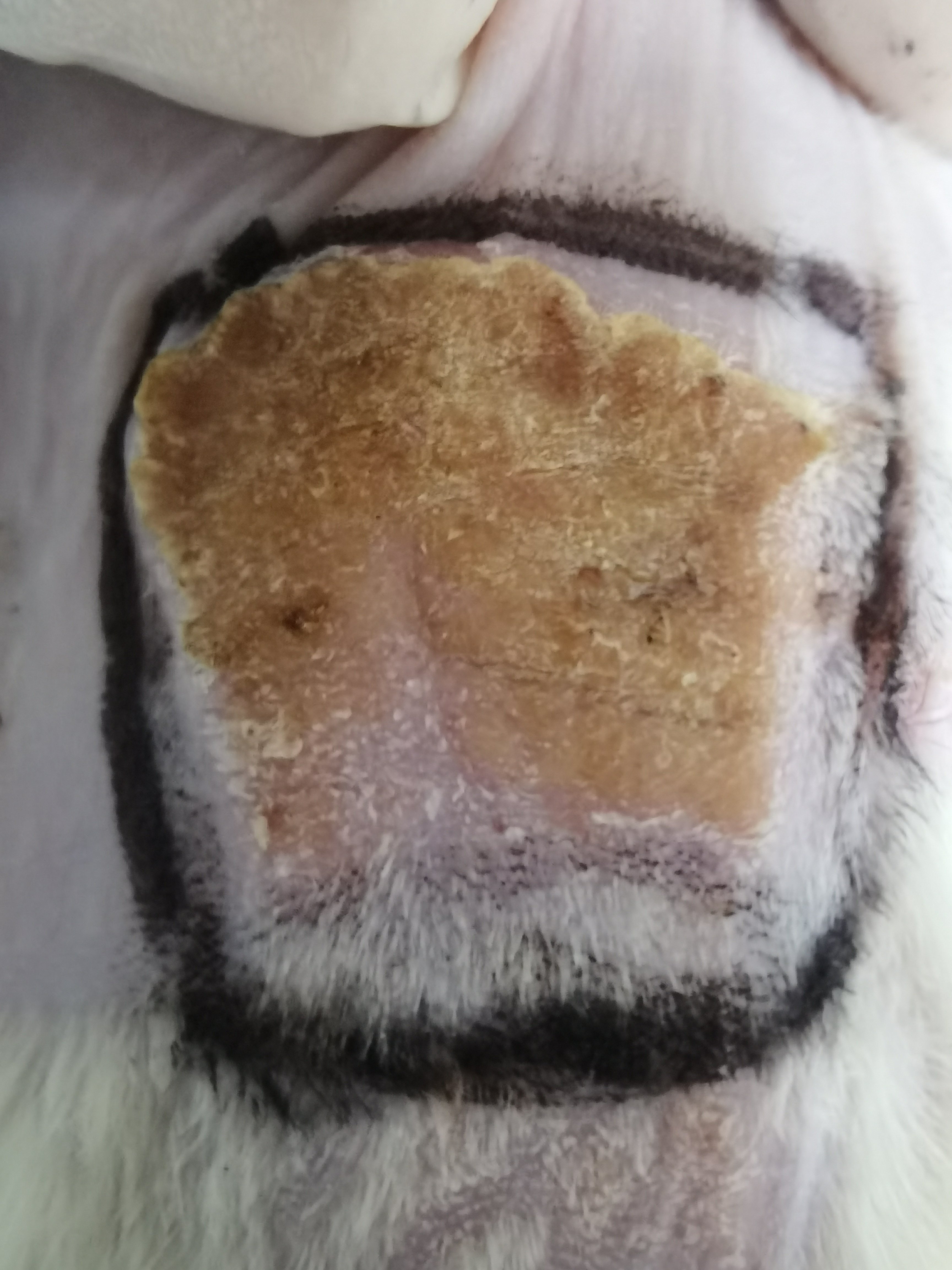

Supplement: S3 File — (ZIP) [file pone.0330078.s003.zip › Animal experiment/Control/14d 3.jpg]

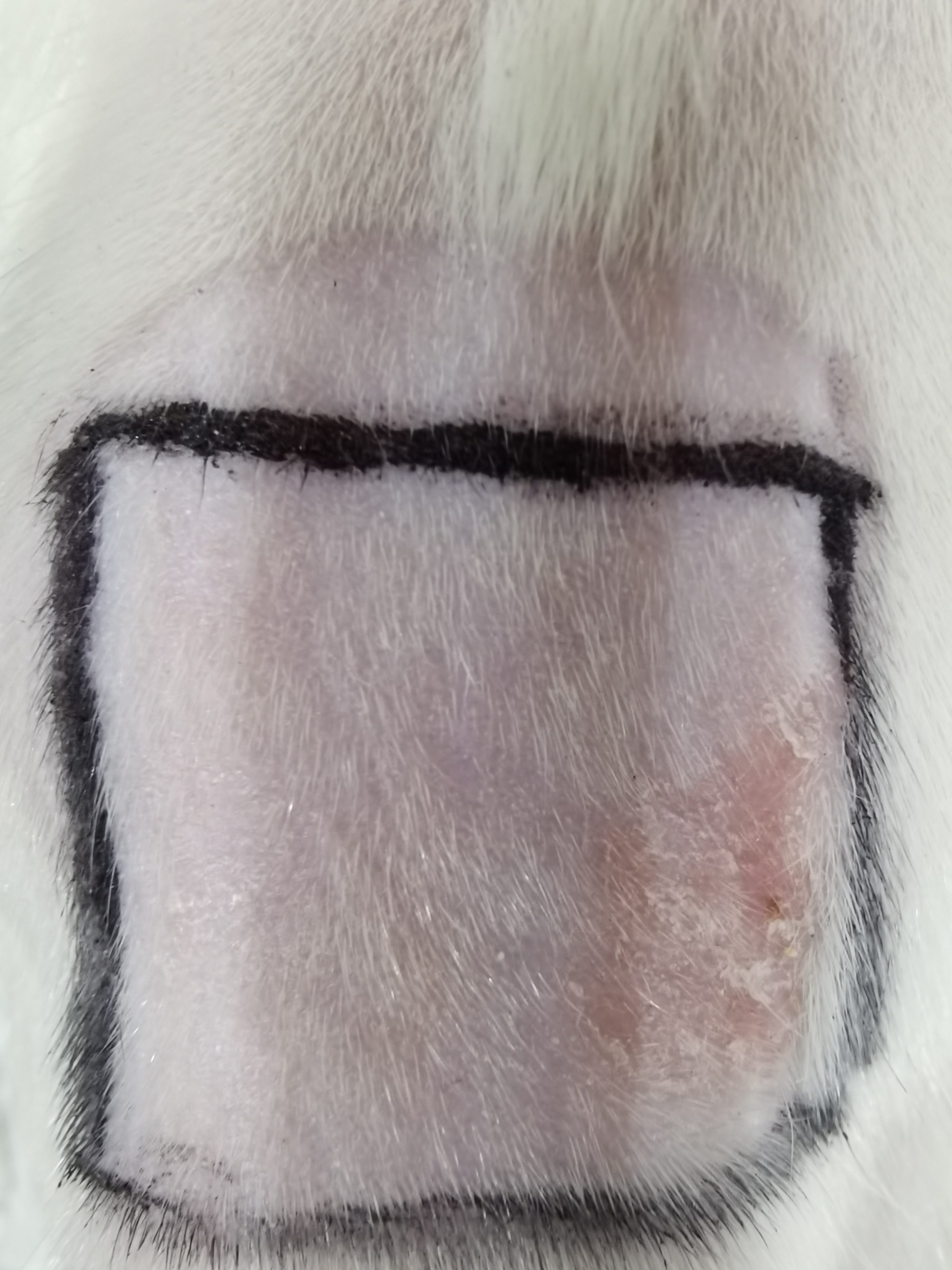

Supplement: S3 File — (ZIP) [file pone.0330078.s003.zip › Animal experiment/Control/21d 1.jpg]

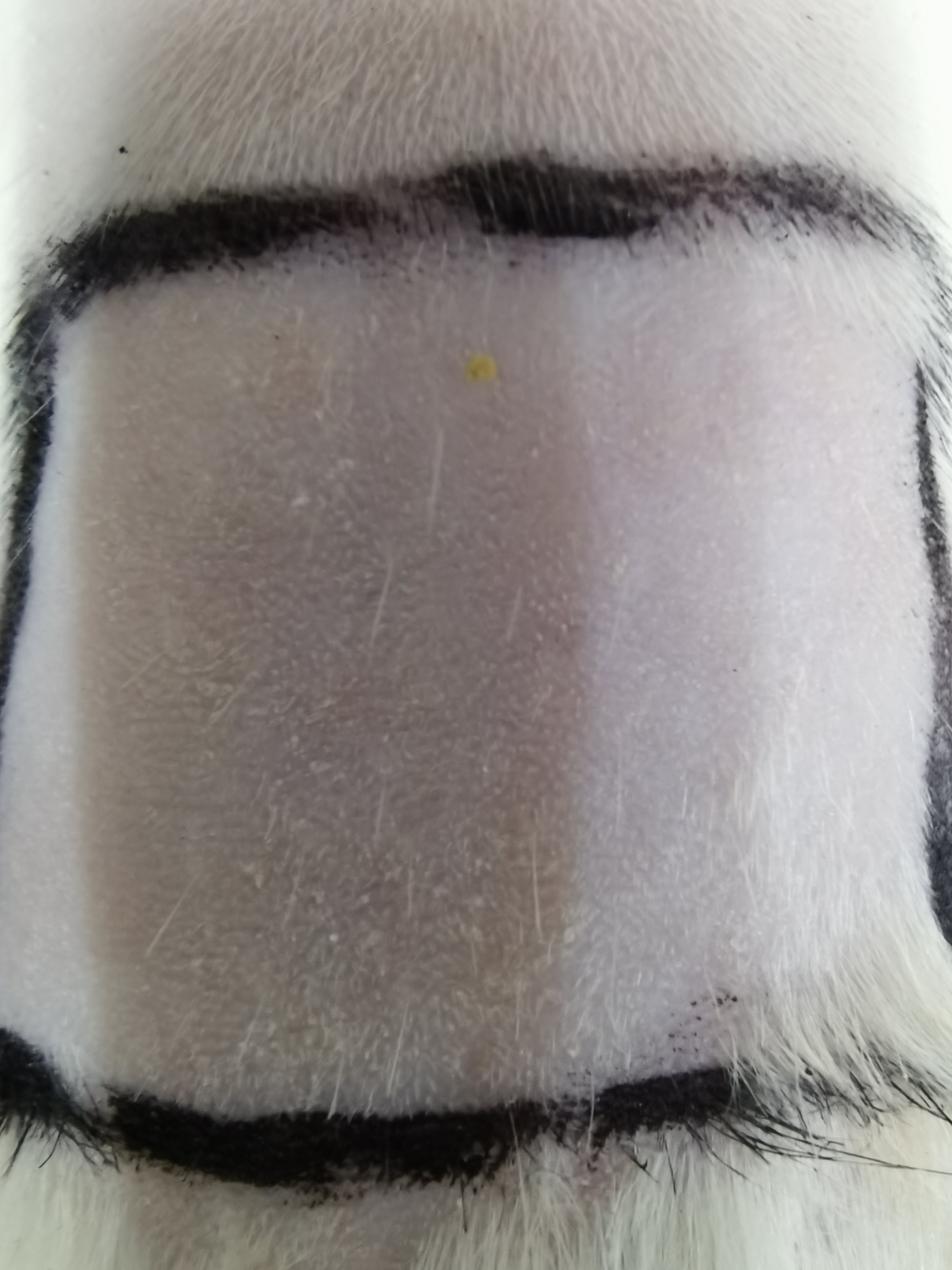

Supplement: S3 File — (ZIP) [file pone.0330078.s003.zip › Animal experiment/Control/21d 2.jpg]

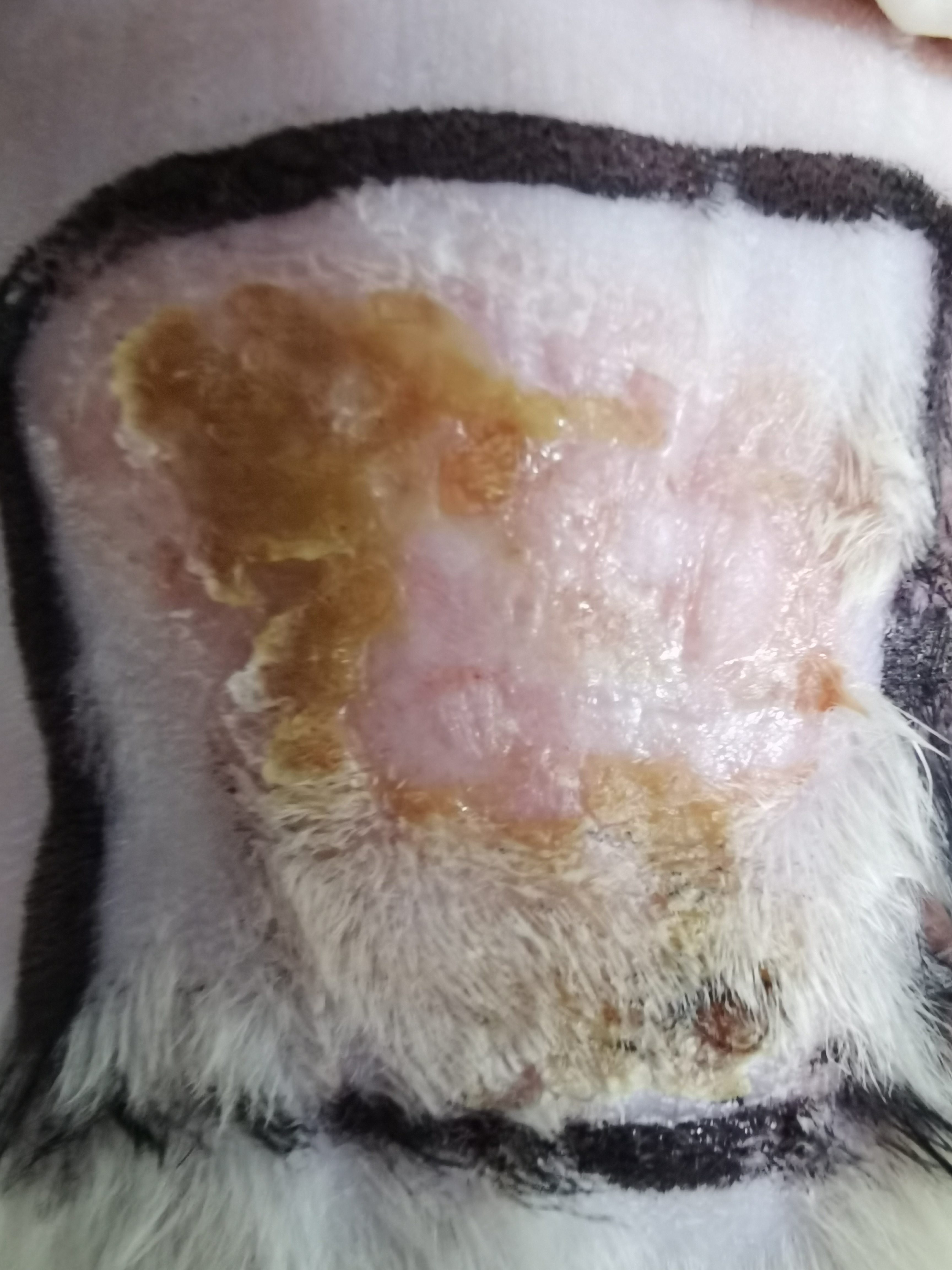

Supplement: S3 File — (ZIP) [file pone.0330078.s003.zip › Animal experiment/Control/21d 3.jpg]

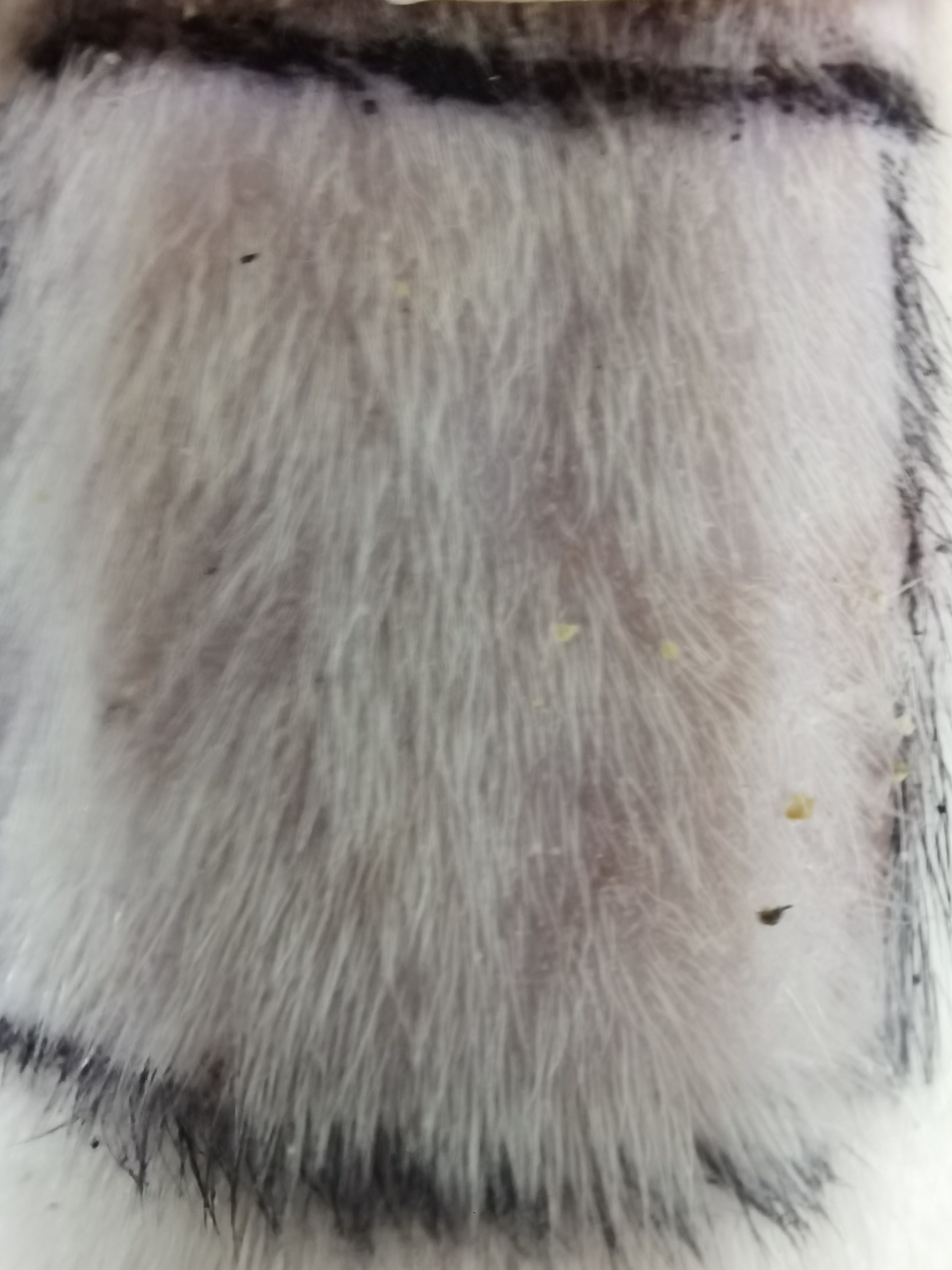

Supplement: S3 File — (ZIP) [file pone.0330078.s003.zip › Animal experiment/Control/28d 1.jpg]

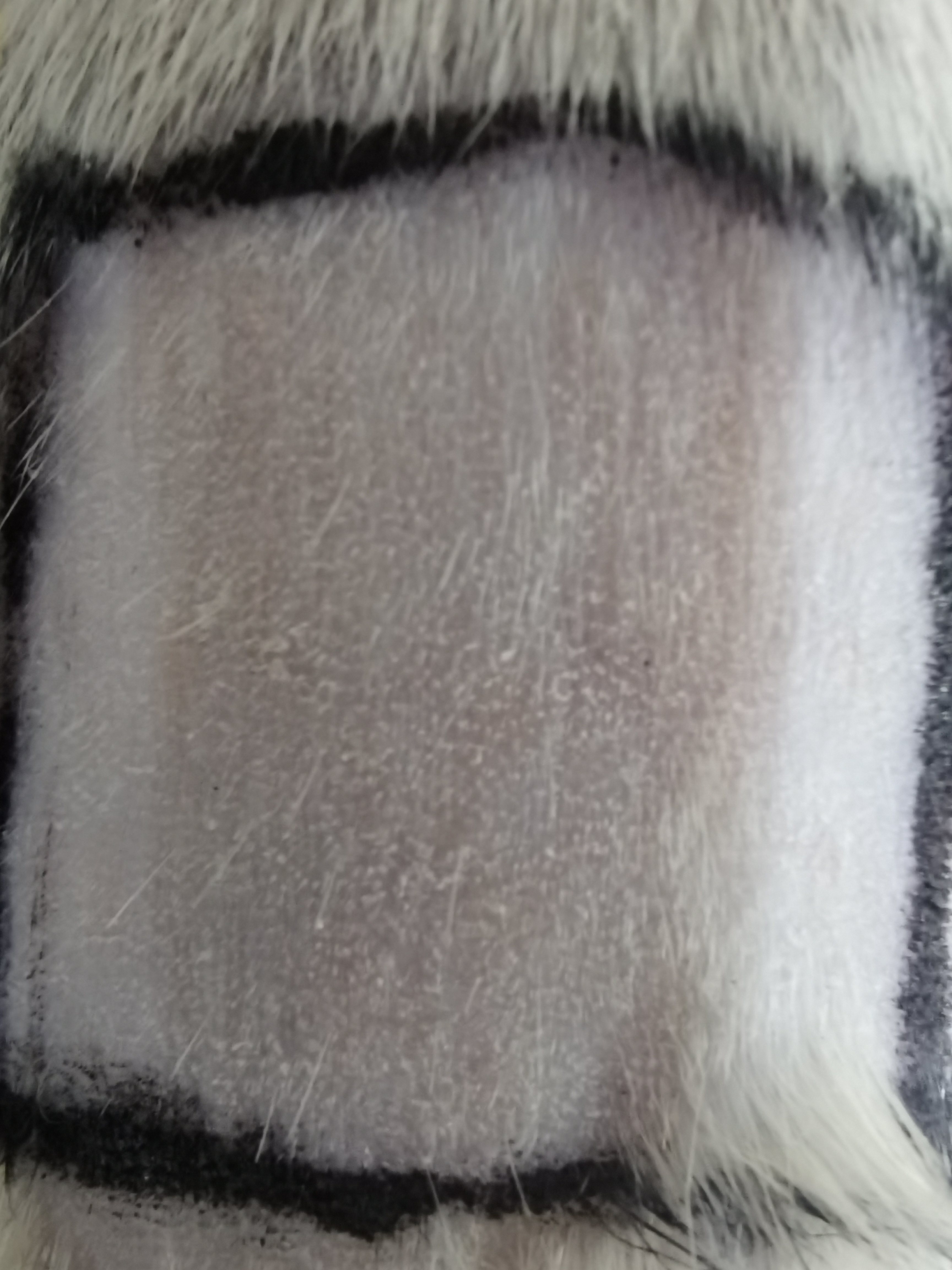

Supplement: S3 File — (ZIP) [file pone.0330078.s003.zip › Animal experiment/Control/28d 2.jpg]

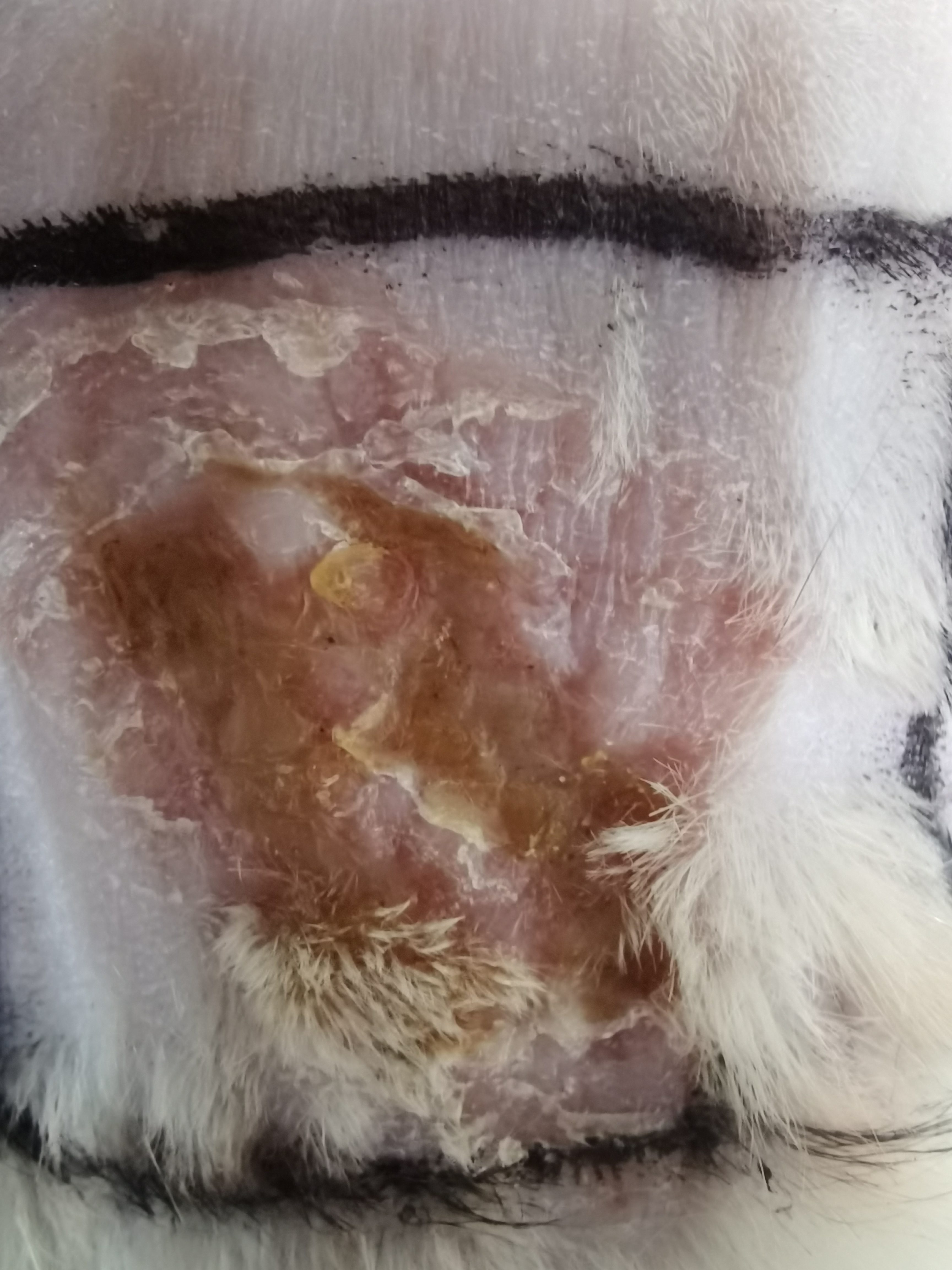

Supplement: S3 File — (ZIP) [file pone.0330078.s003.zip › Animal experiment/Control/28d 3.jpg]

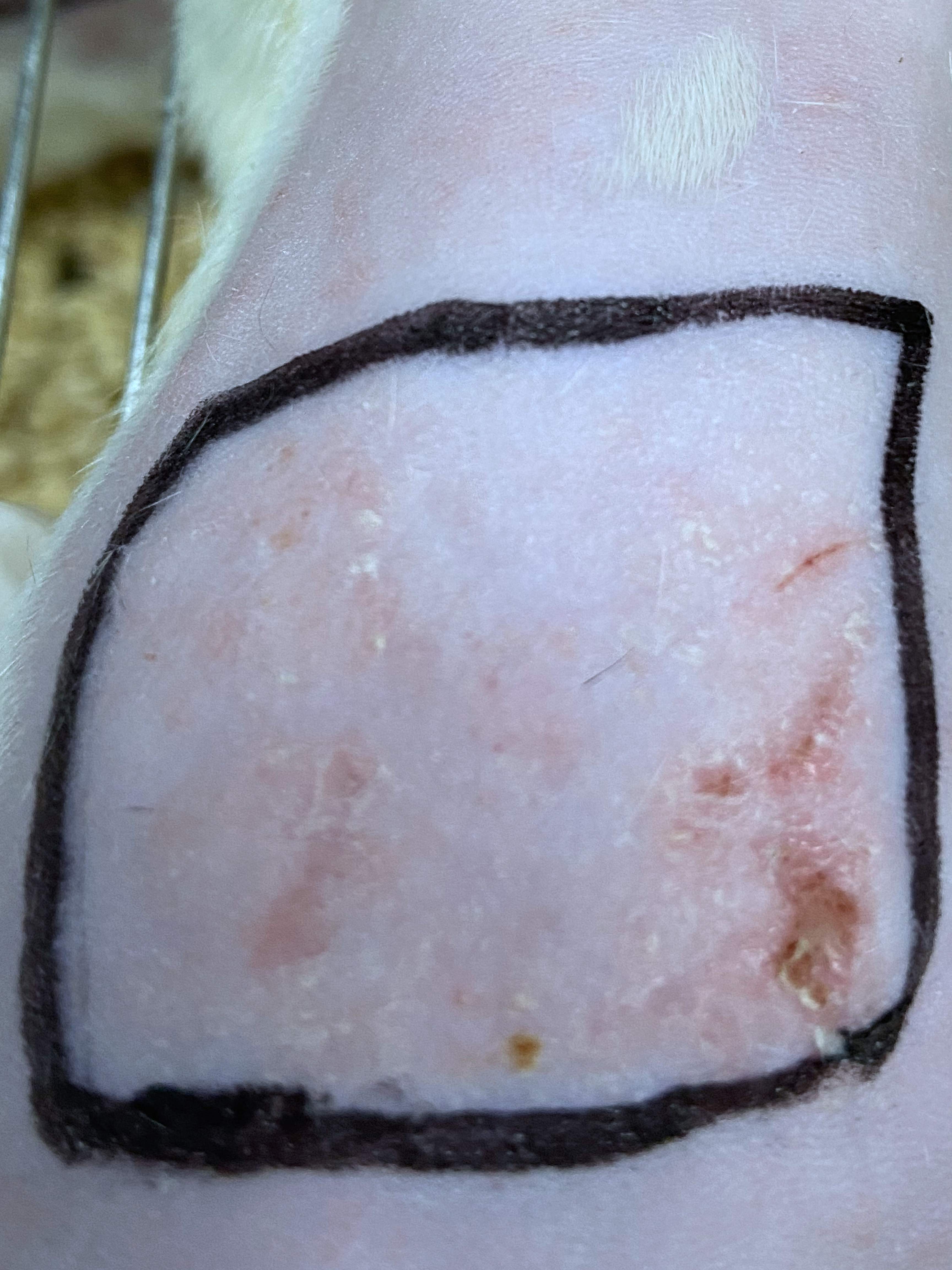

Supplement: S3 File — (ZIP) [file pone.0330078.s003.zip › Animal experiment/Control/3d 1.jpg]

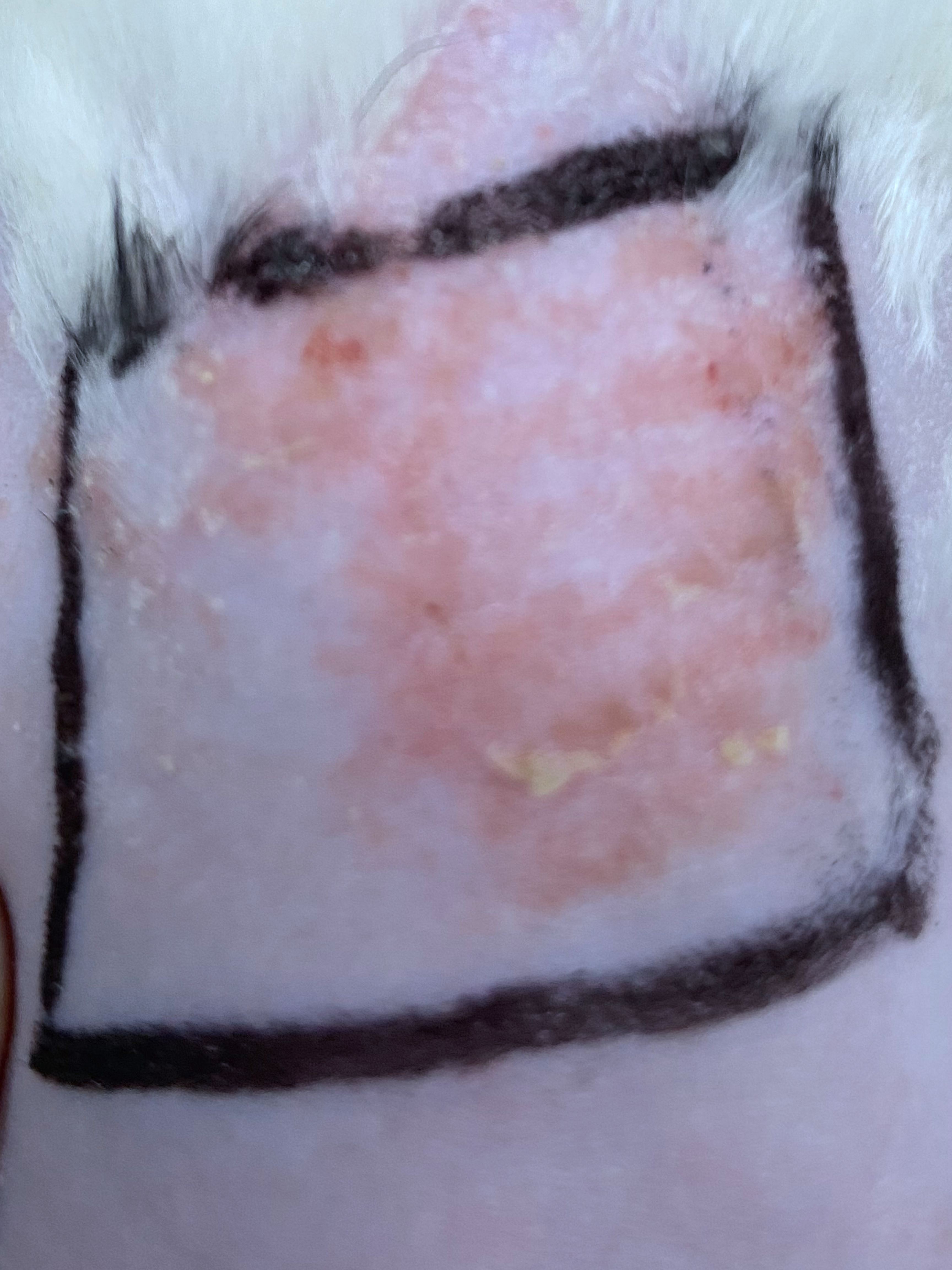

Supplement: S3 File — (ZIP) [file pone.0330078.s003.zip › Animal experiment/Control/3d 2.jpg]

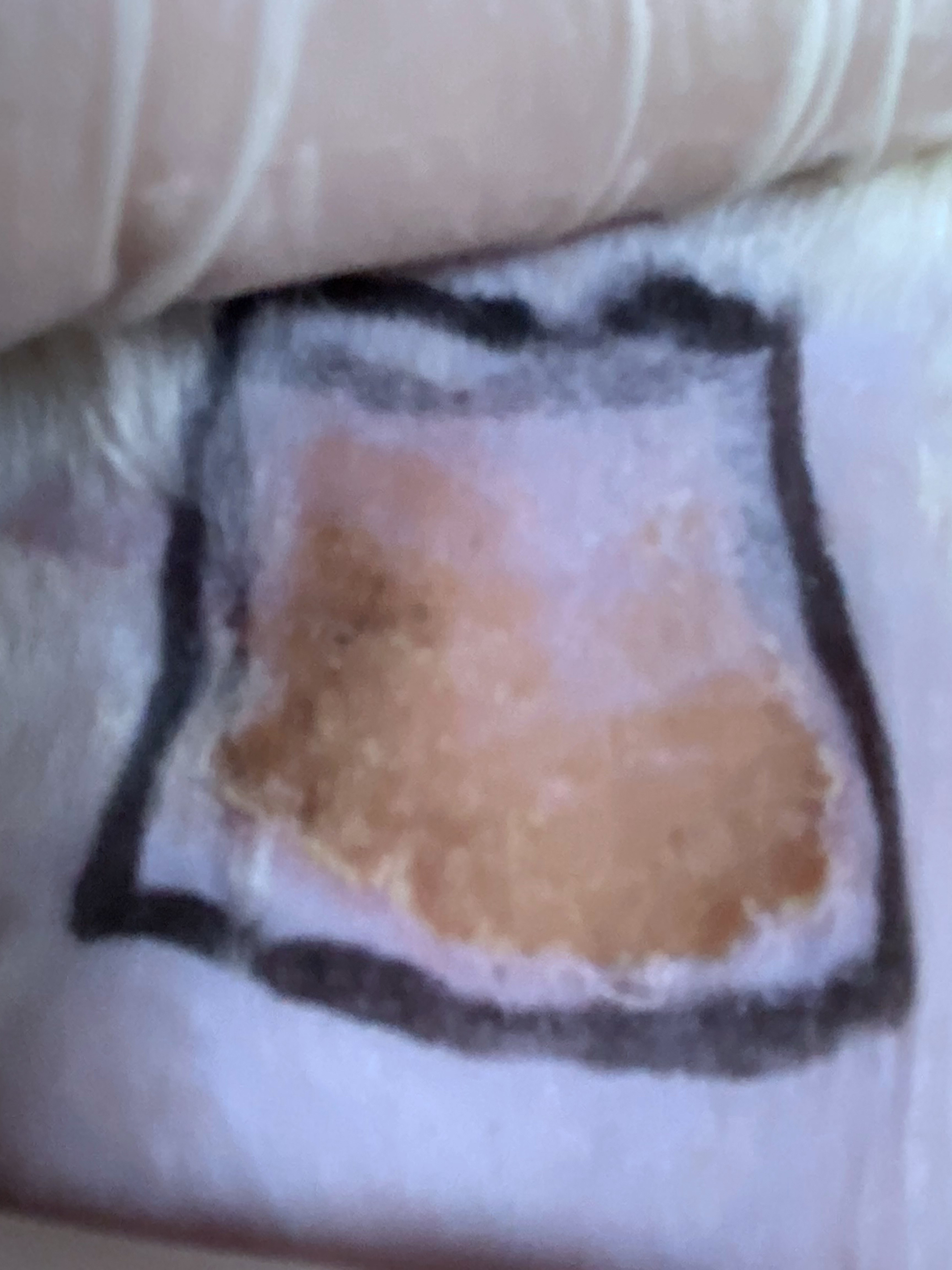

Supplement: S3 File — (ZIP) [file pone.0330078.s003.zip › Animal experiment/Control/3d 3.jpg]

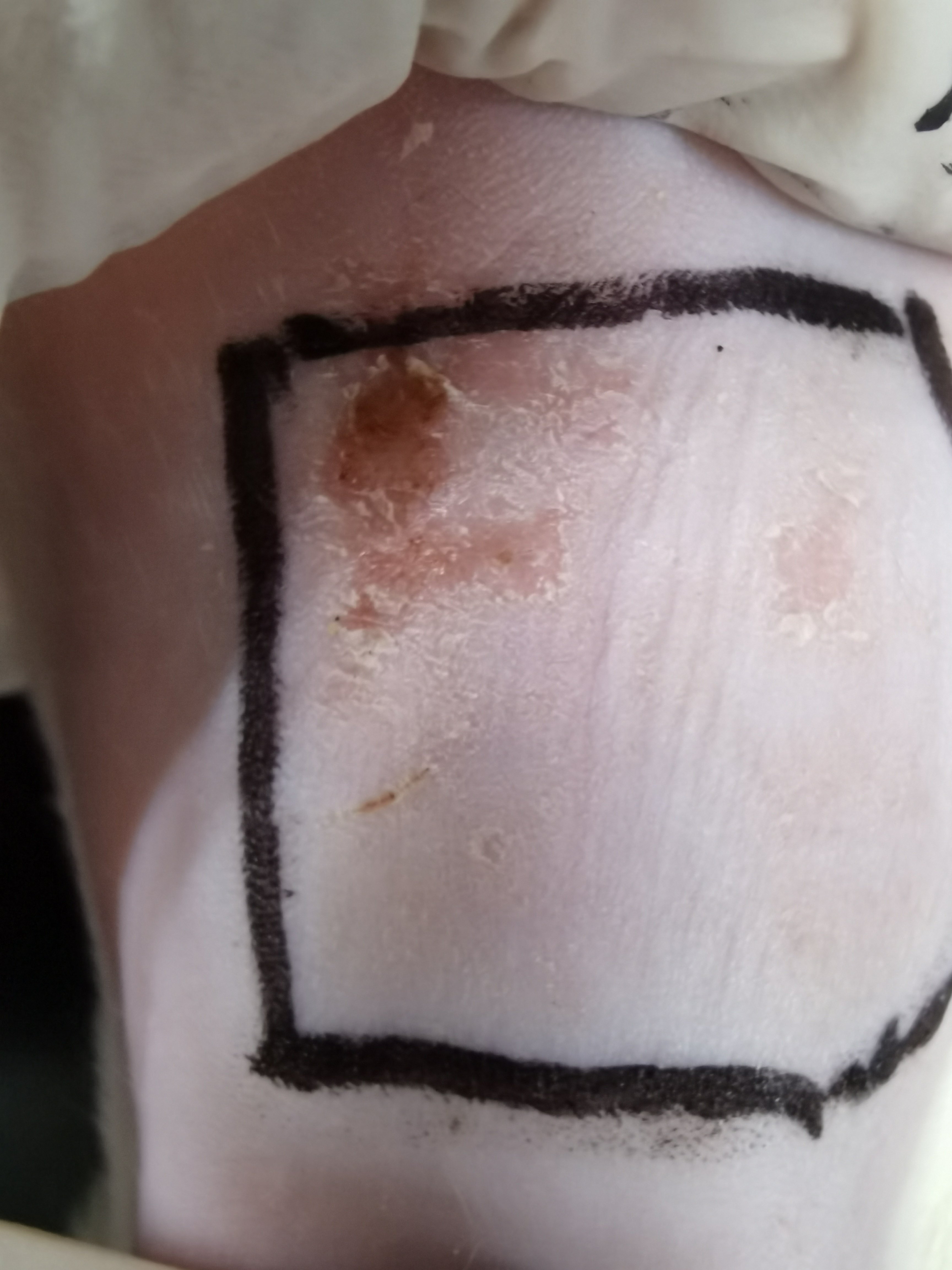

Supplement: S3 File — (ZIP) [file pone.0330078.s003.zip › Animal experiment/Control/7d 1.jpg]

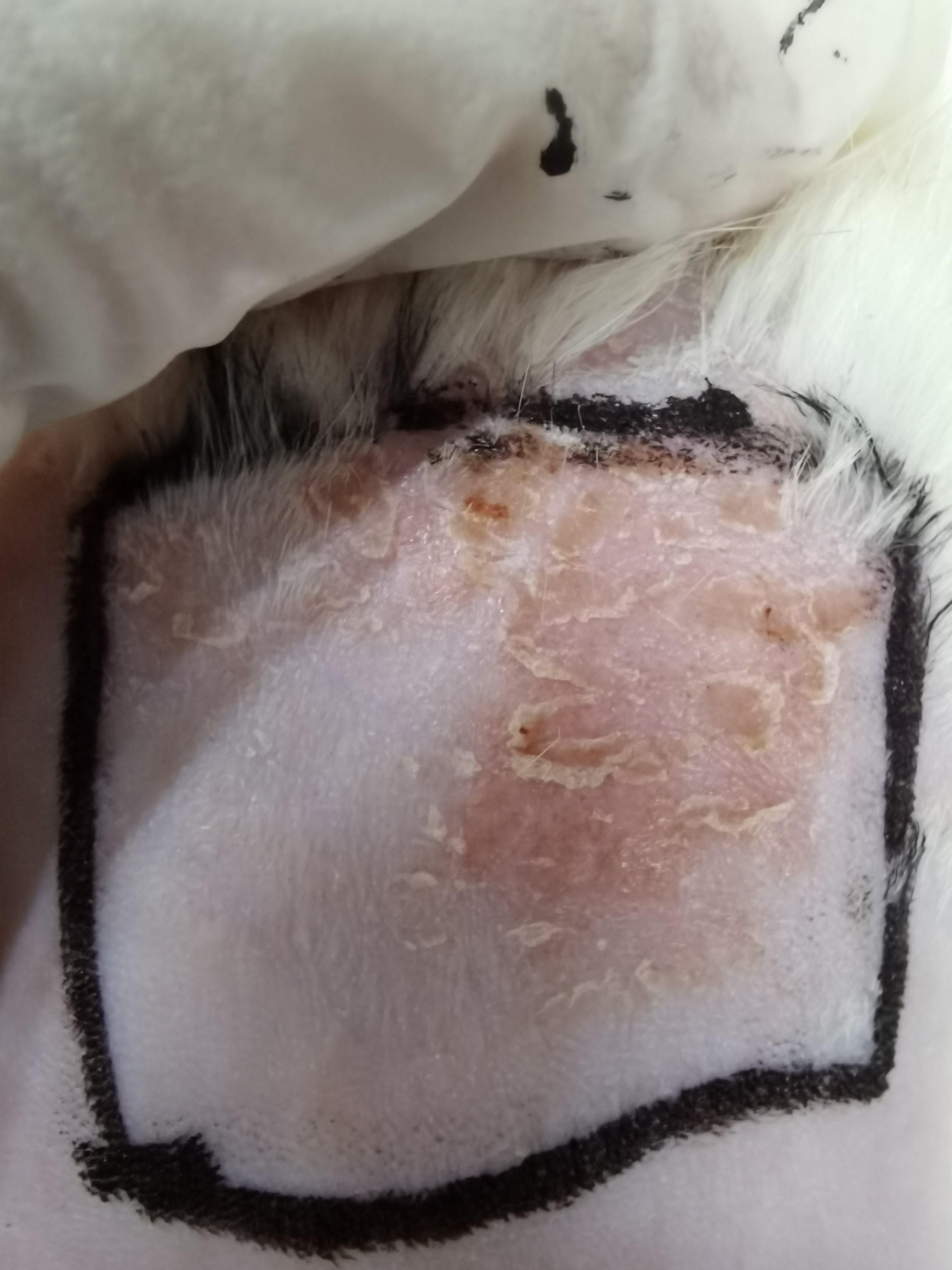

Supplement: S3 File — (ZIP) [file pone.0330078.s003.zip › Animal experiment/Control/7d 2.jpg]

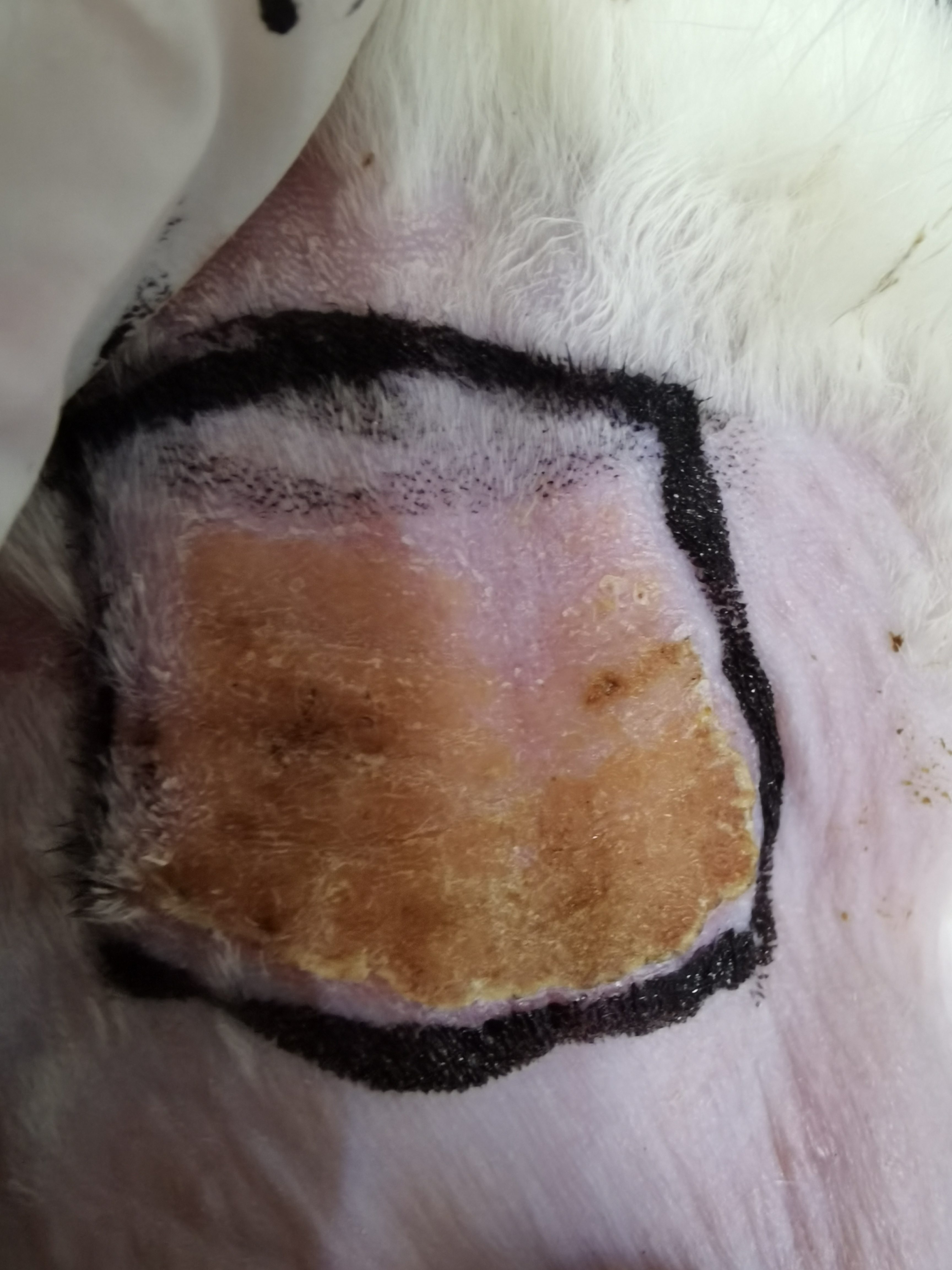

Supplement: S3 File — (ZIP) [file pone.0330078.s003.zip › Animal experiment/Control/7d 3.jpg]

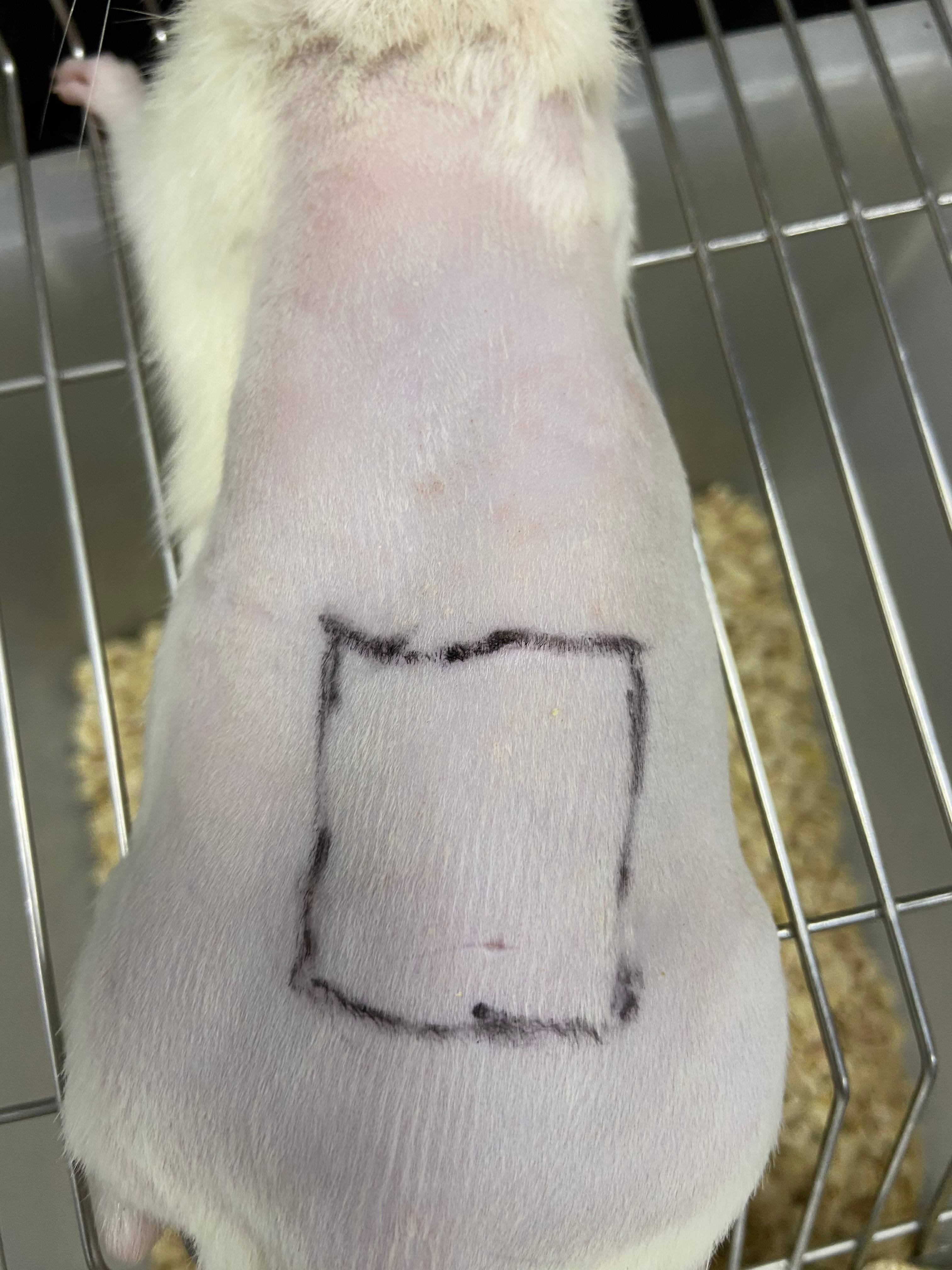

Supplement: S3 File — (ZIP) [file pone.0330078.s003.zip › Animal experiment/HAMCC/0d 1.jpg]

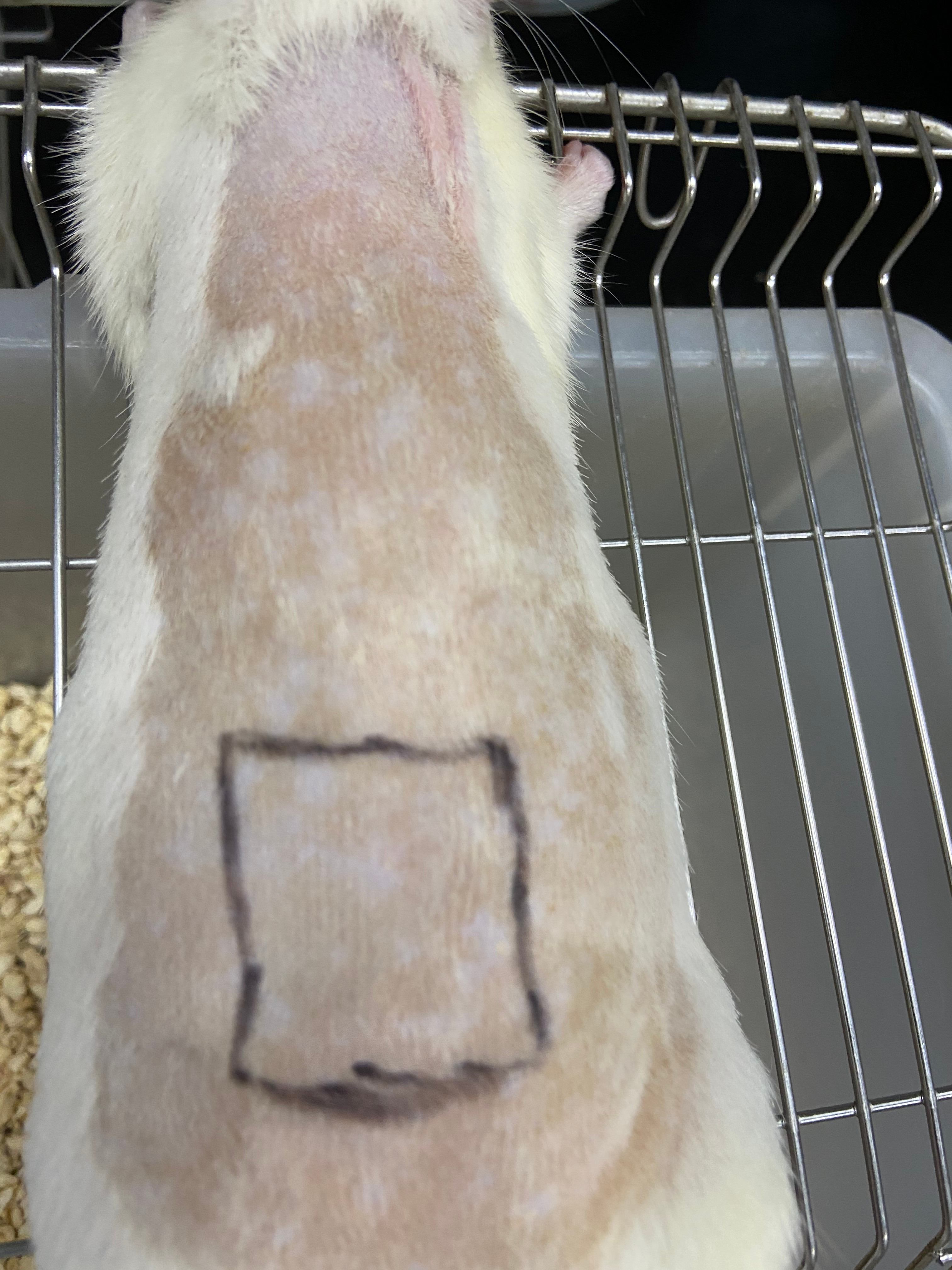

Supplement: S3 File — (ZIP) [file pone.0330078.s003.zip › Animal experiment/HAMCC/0d 2.jpg]

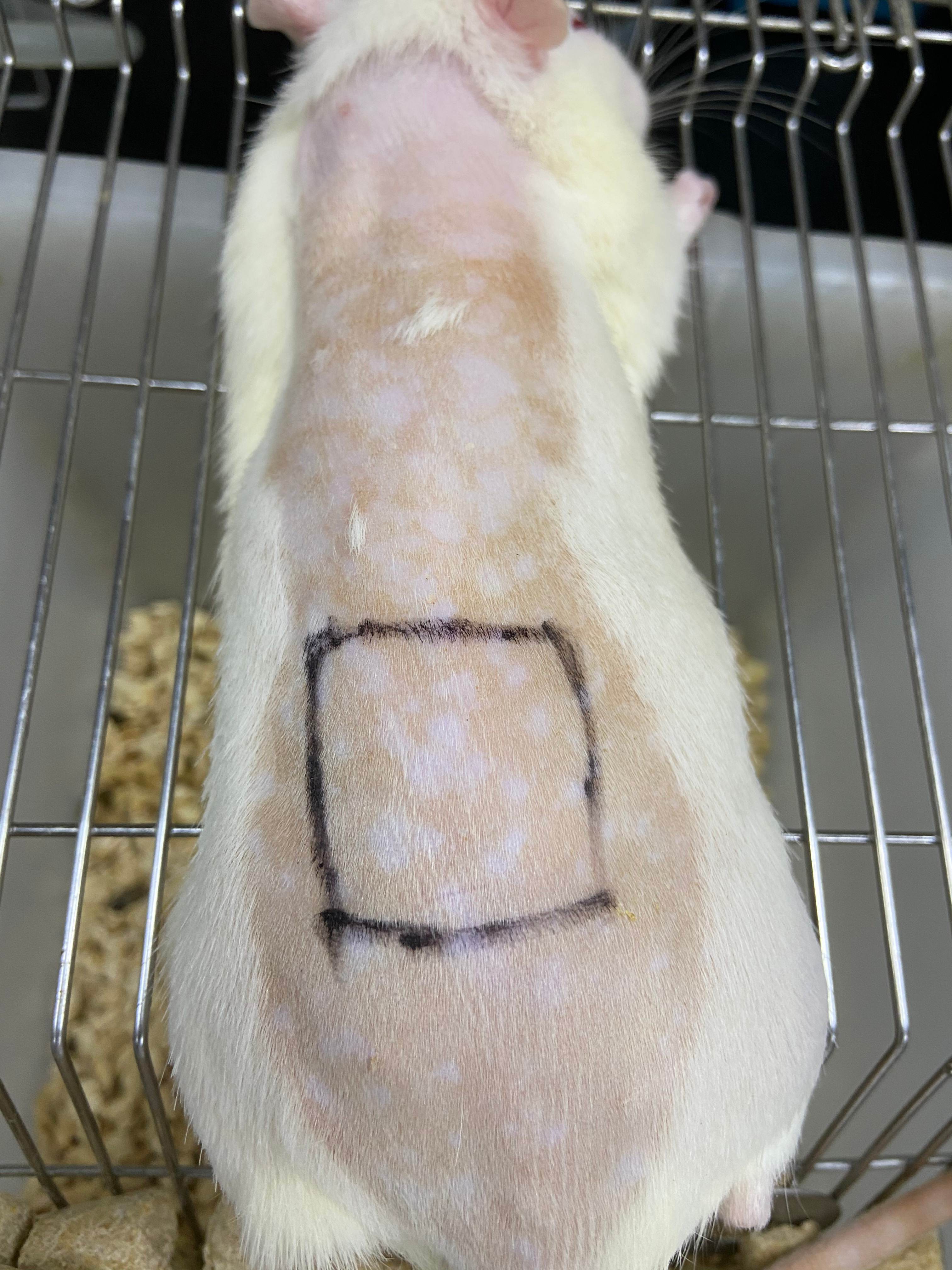

Supplement: S3 File — (ZIP) [file pone.0330078.s003.zip › Animal experiment/HAMCC/0d 3.jpg]

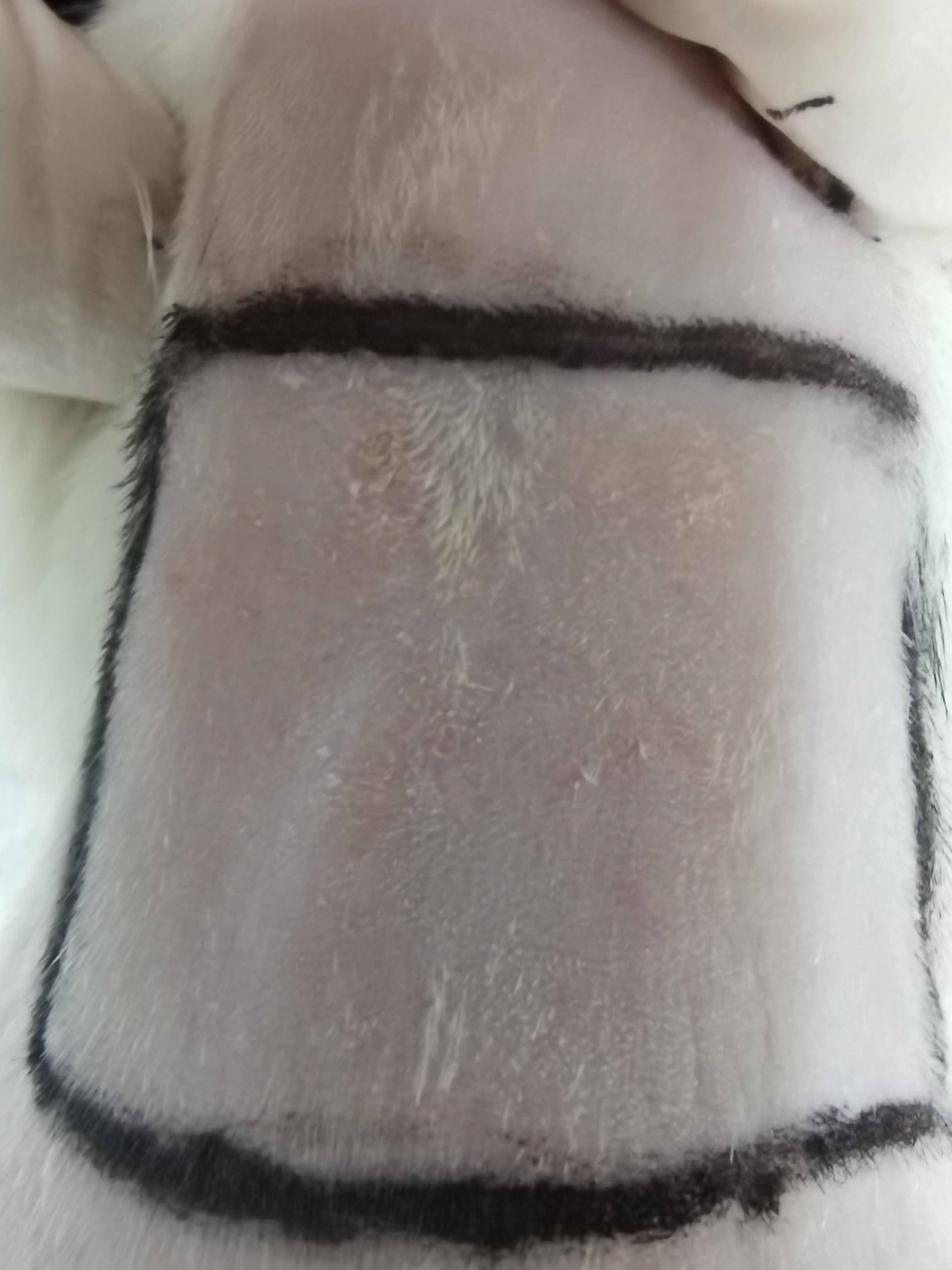

Supplement: S3 File — (ZIP) [file pone.0330078.s003.zip › Animal experiment/HAMCC/14d 1.jpg]

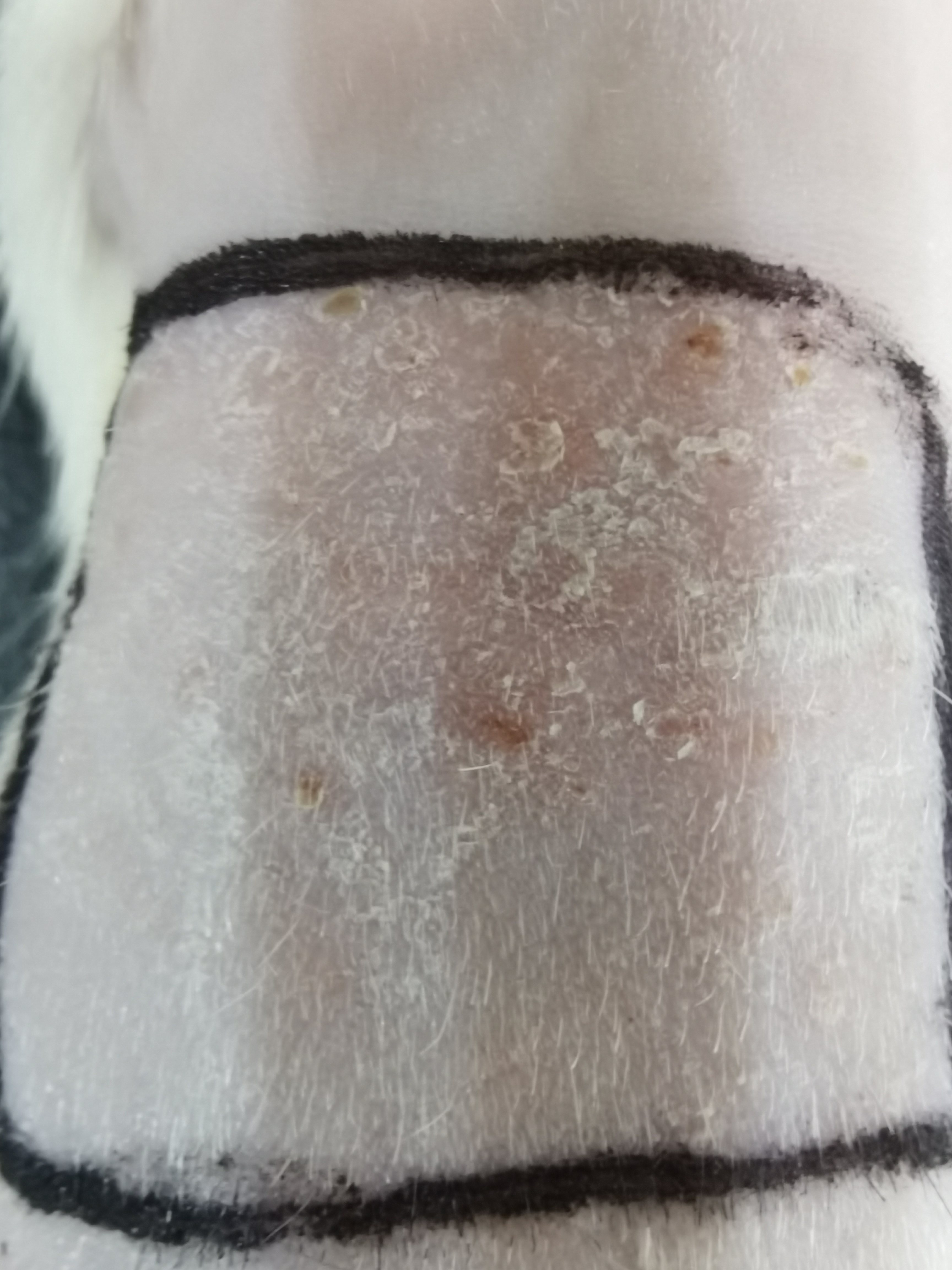

Supplement: S3 File — (ZIP) [file pone.0330078.s003.zip › Animal experiment/HAMCC/14d 2.jpg]

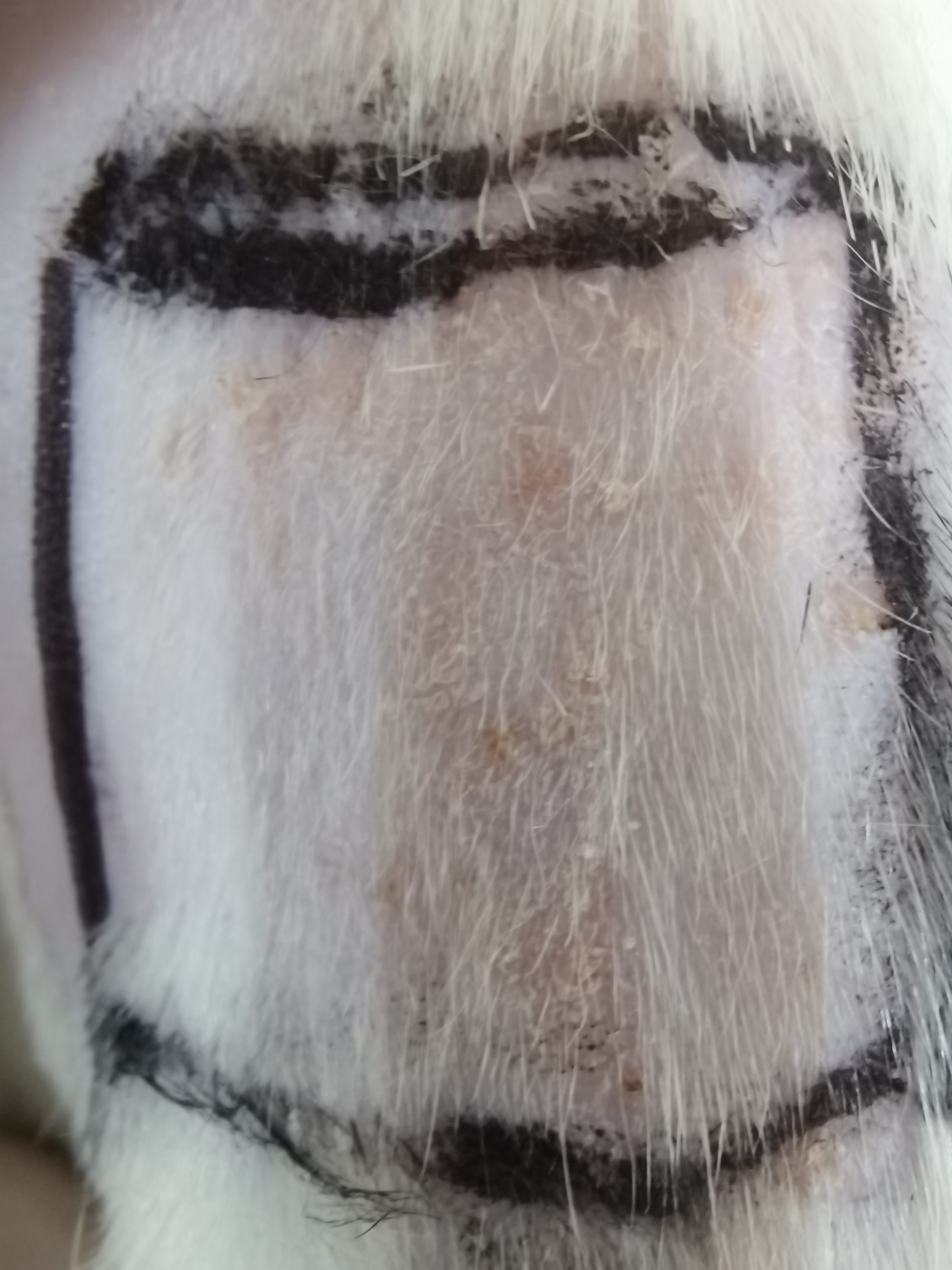

Supplement: S3 File — (ZIP) [file pone.0330078.s003.zip › Animal experiment/HAMCC/14d 3.jpg]

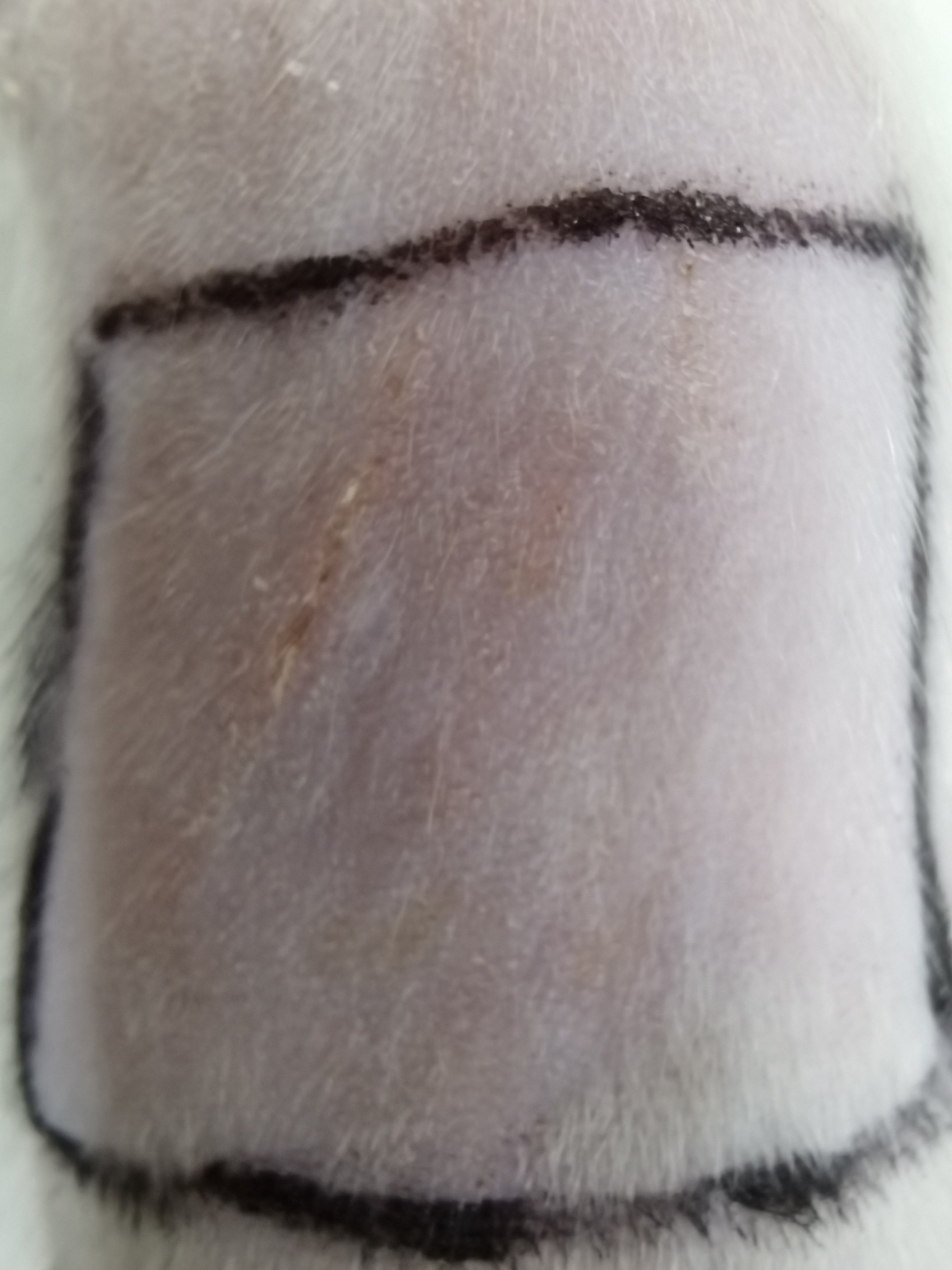

Supplement: S3 File — (ZIP) [file pone.0330078.s003.zip › Animal experiment/HAMCC/21d 1.jpg]

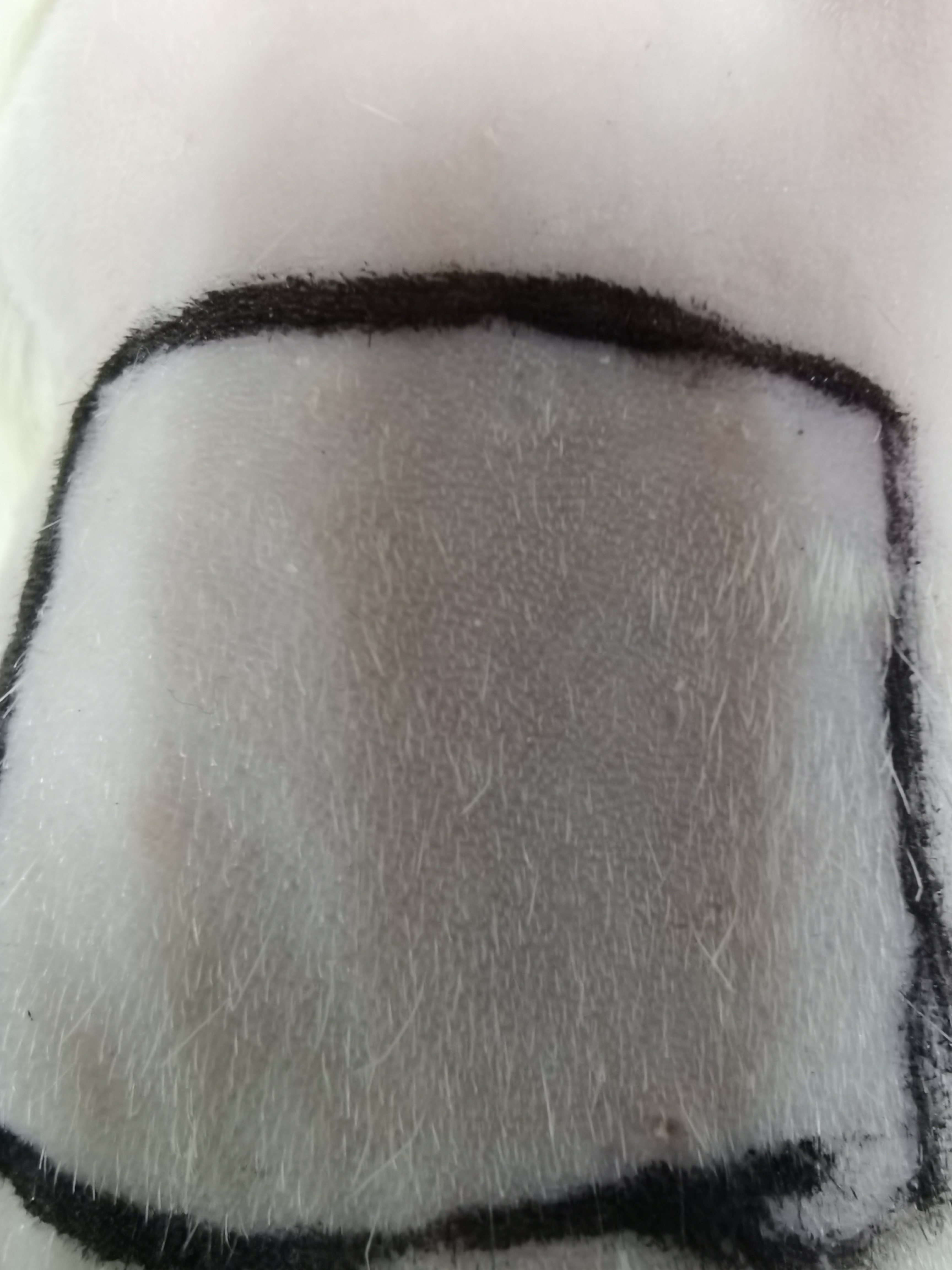

Supplement: S3 File — (ZIP) [file pone.0330078.s003.zip › Animal experiment/HAMCC/21d 2.jpg]

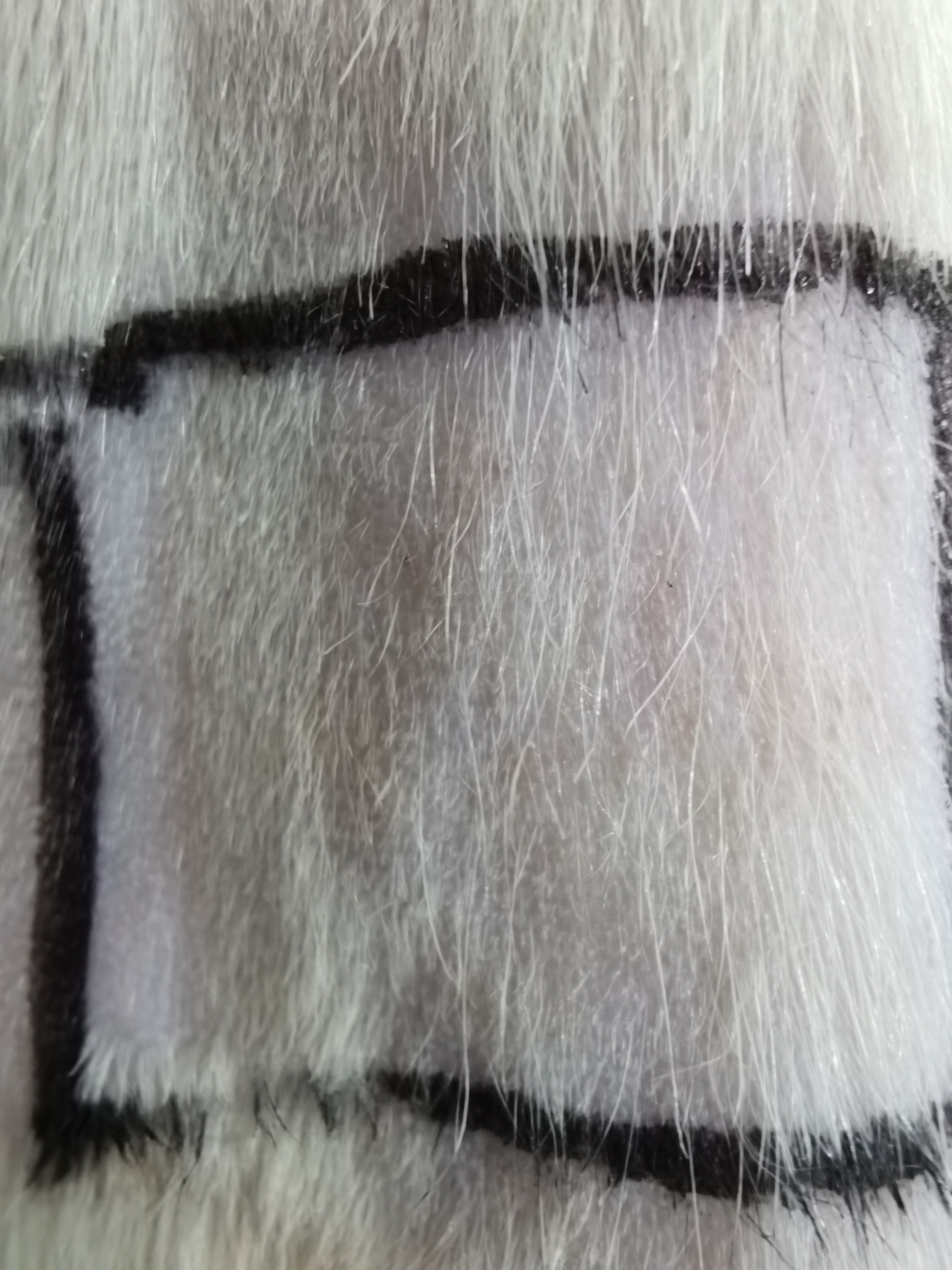

Supplement: S3 File — (ZIP) [file pone.0330078.s003.zip › Animal experiment/HAMCC/21d 3.jpg]

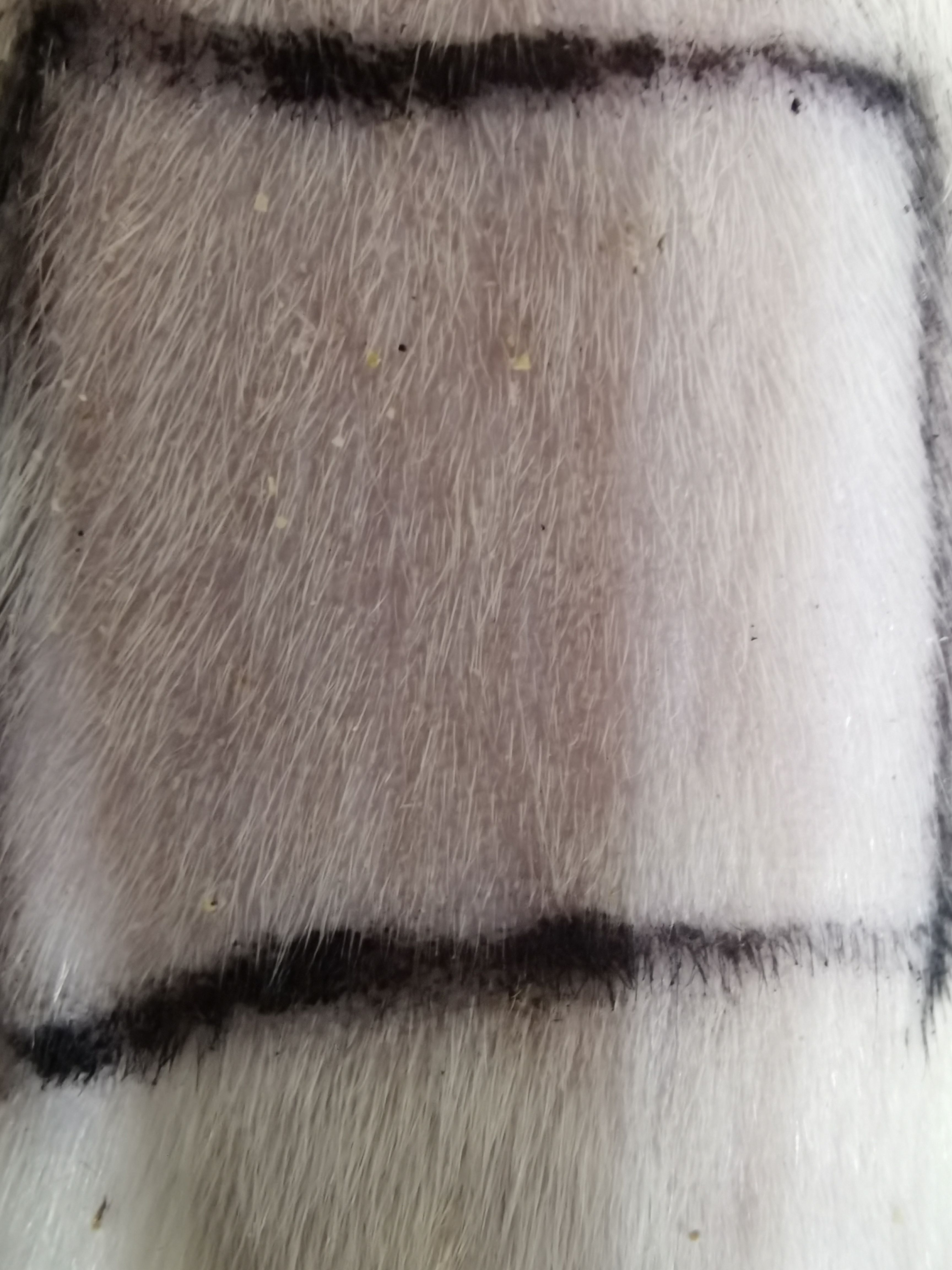

Supplement: S3 File — (ZIP) [file pone.0330078.s003.zip › Animal experiment/HAMCC/28d 1.jpg]

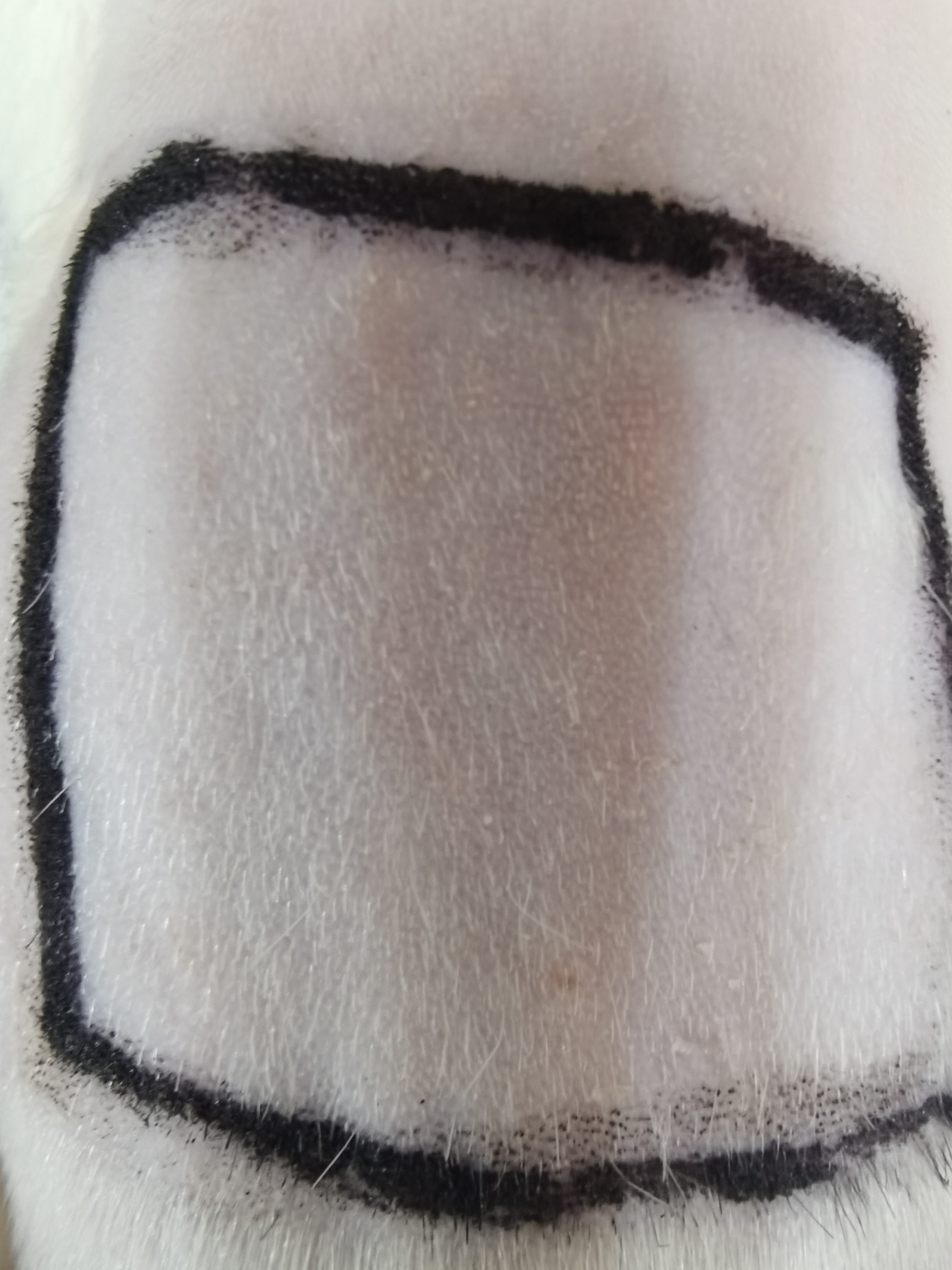

Supplement: S3 File — (ZIP) [file pone.0330078.s003.zip › Animal experiment/HAMCC/28d 2.jpg]

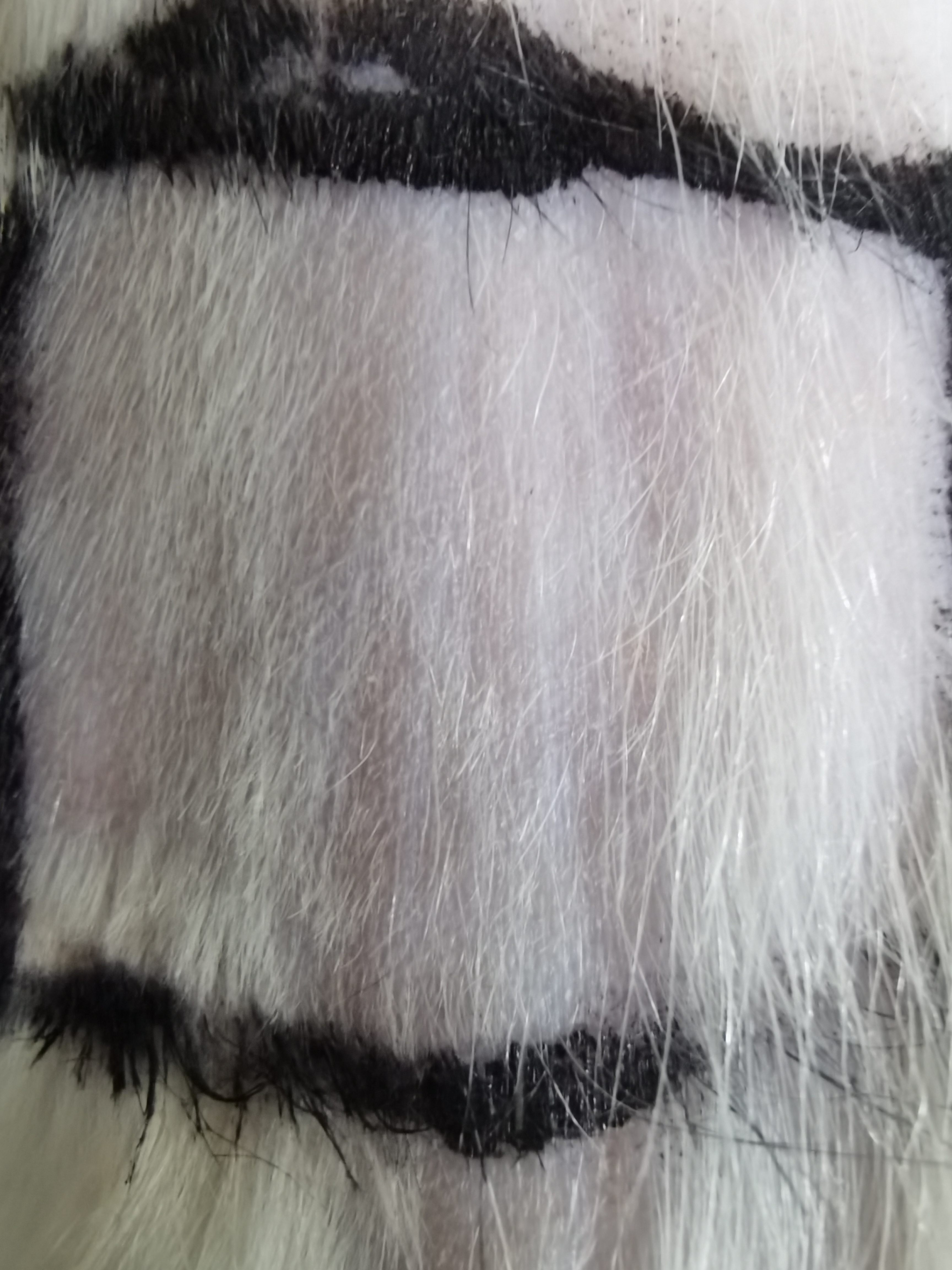

Supplement: S3 File — (ZIP) [file pone.0330078.s003.zip › Animal experiment/HAMCC/2d 3.jpg]

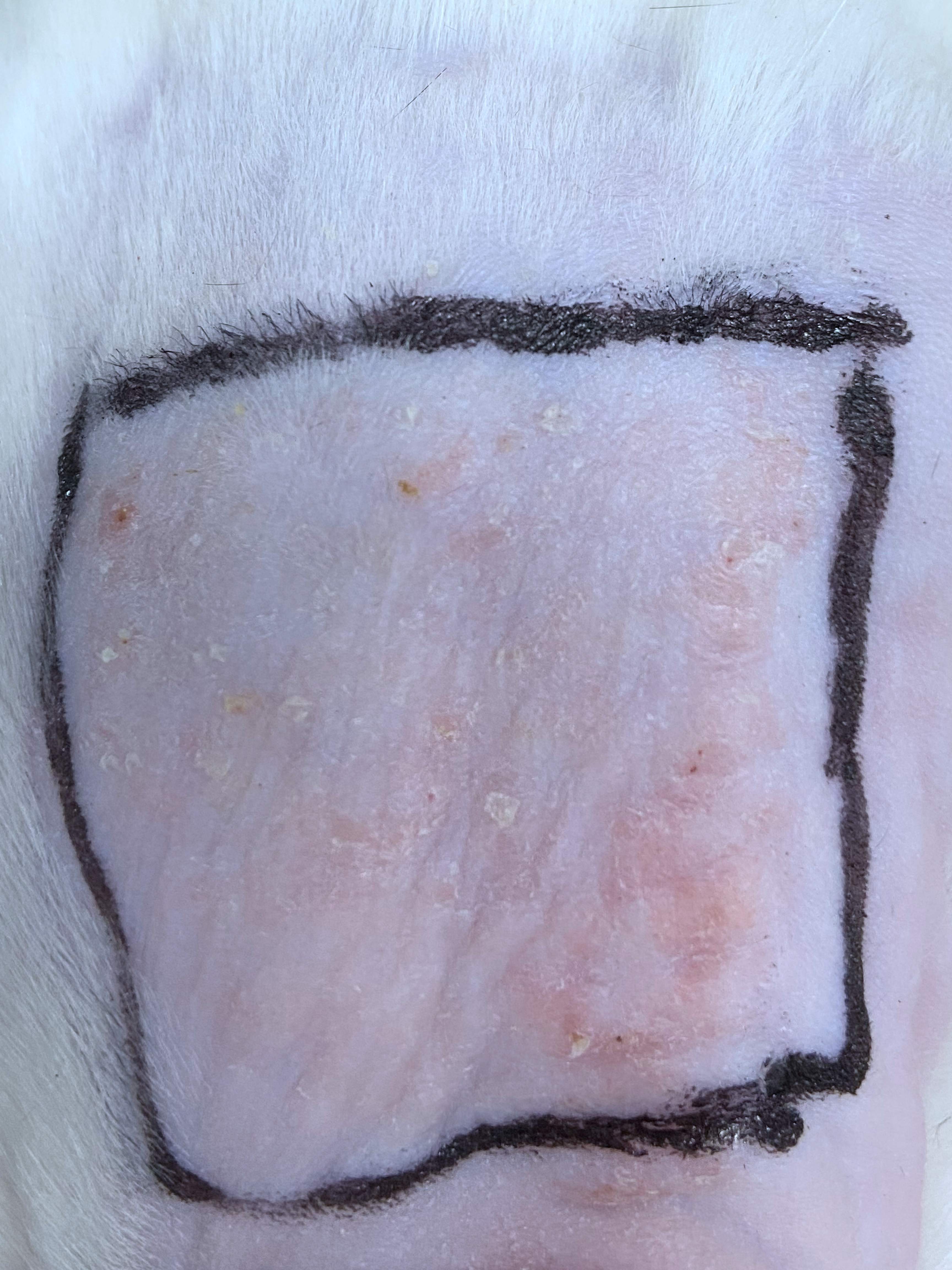

Supplement: S3 File — (ZIP) [file pone.0330078.s003.zip › Animal experiment/HAMCC/3d 1.jpg]

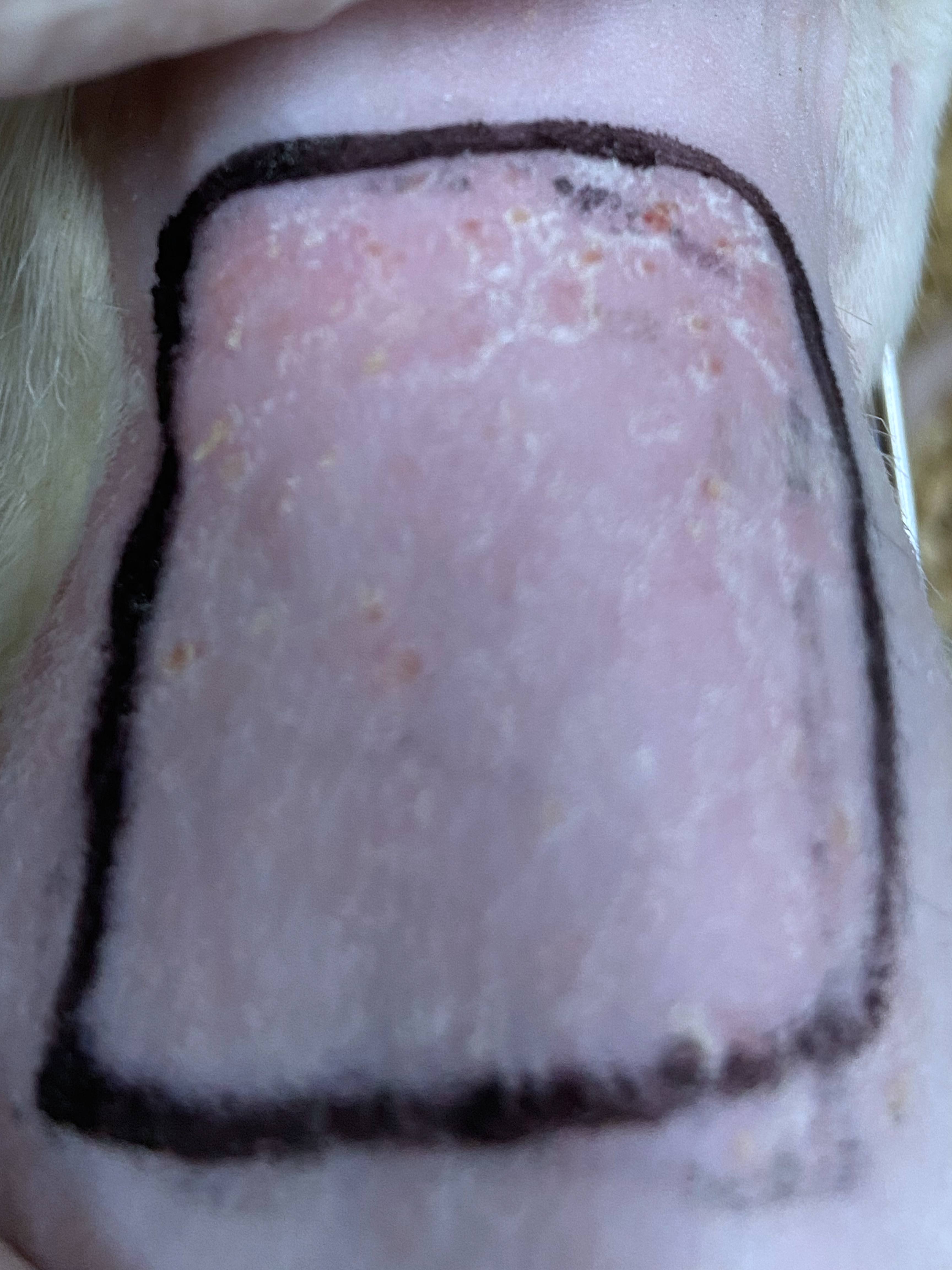

Supplement: S3 File — (ZIP) [file pone.0330078.s003.zip › Animal experiment/HAMCC/3d 2.jpg]

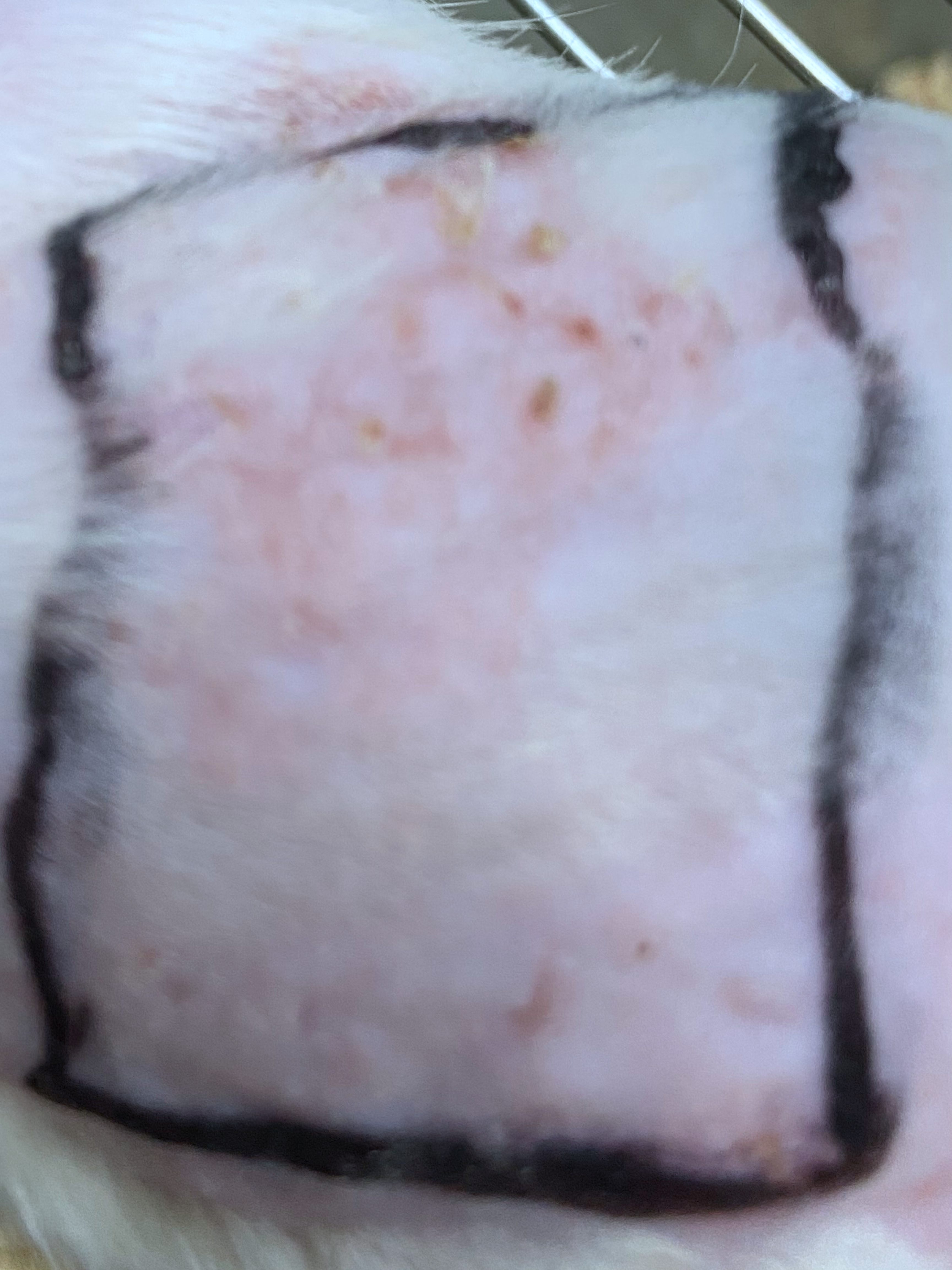

Supplement: S3 File — (ZIP) [file pone.0330078.s003.zip › Animal experiment/HAMCC/3d 3.jpg]

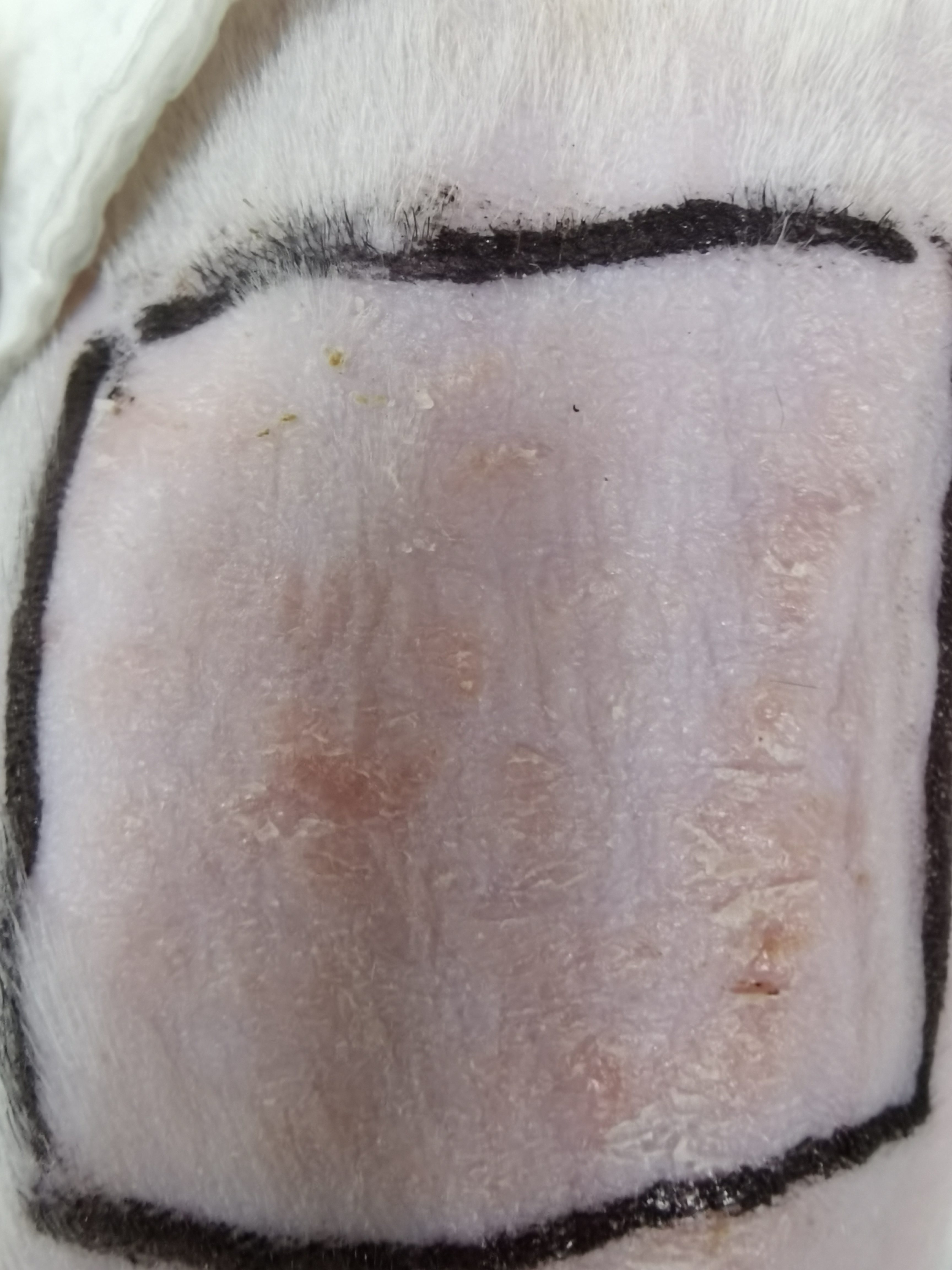

Supplement: S3 File — (ZIP) [file pone.0330078.s003.zip › Animal experiment/HAMCC/7d 1.jpg]

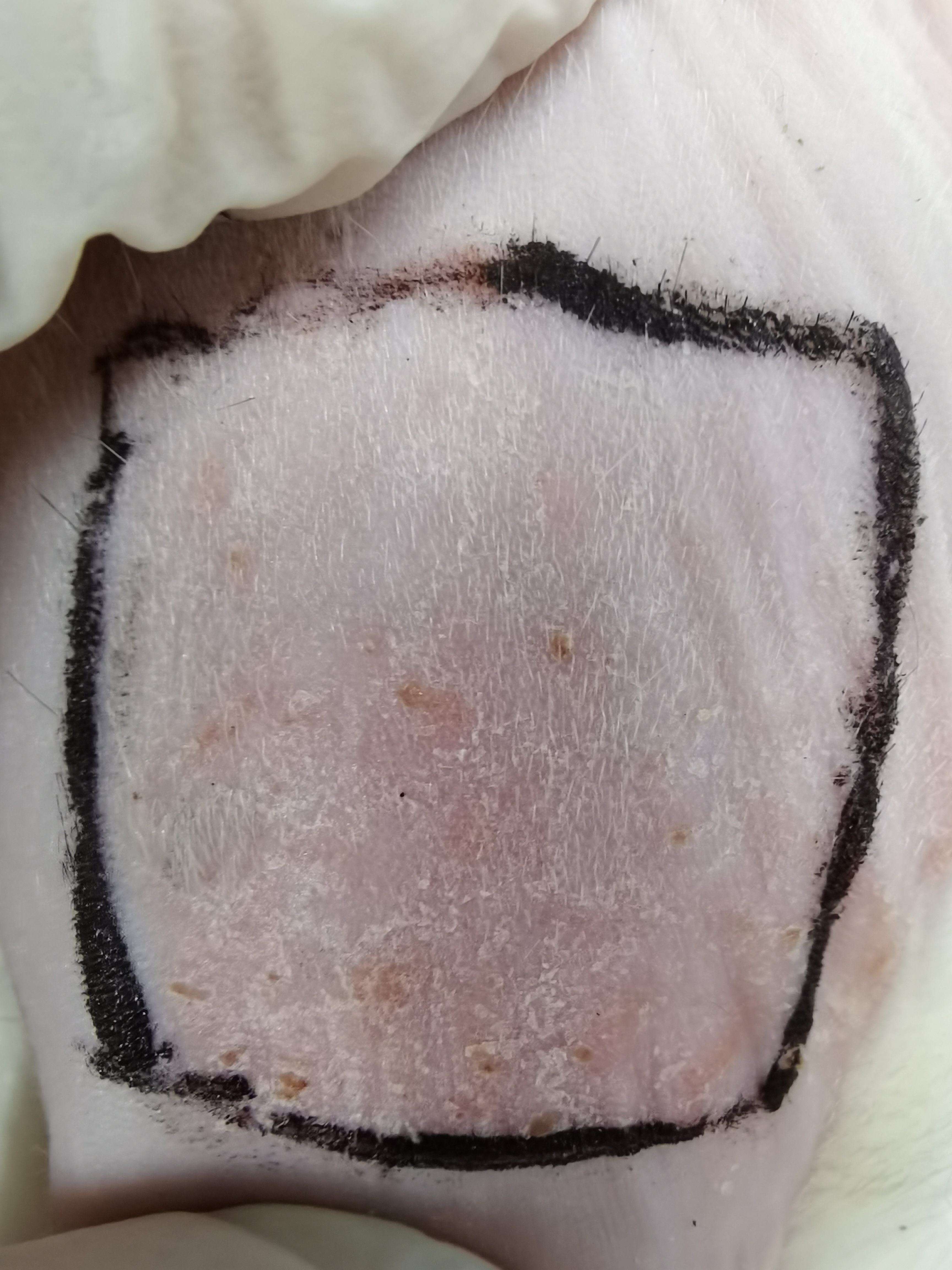

Supplement: S3 File — (ZIP) [file pone.0330078.s003.zip › Animal experiment/HAMCC/7d 2.jpg]

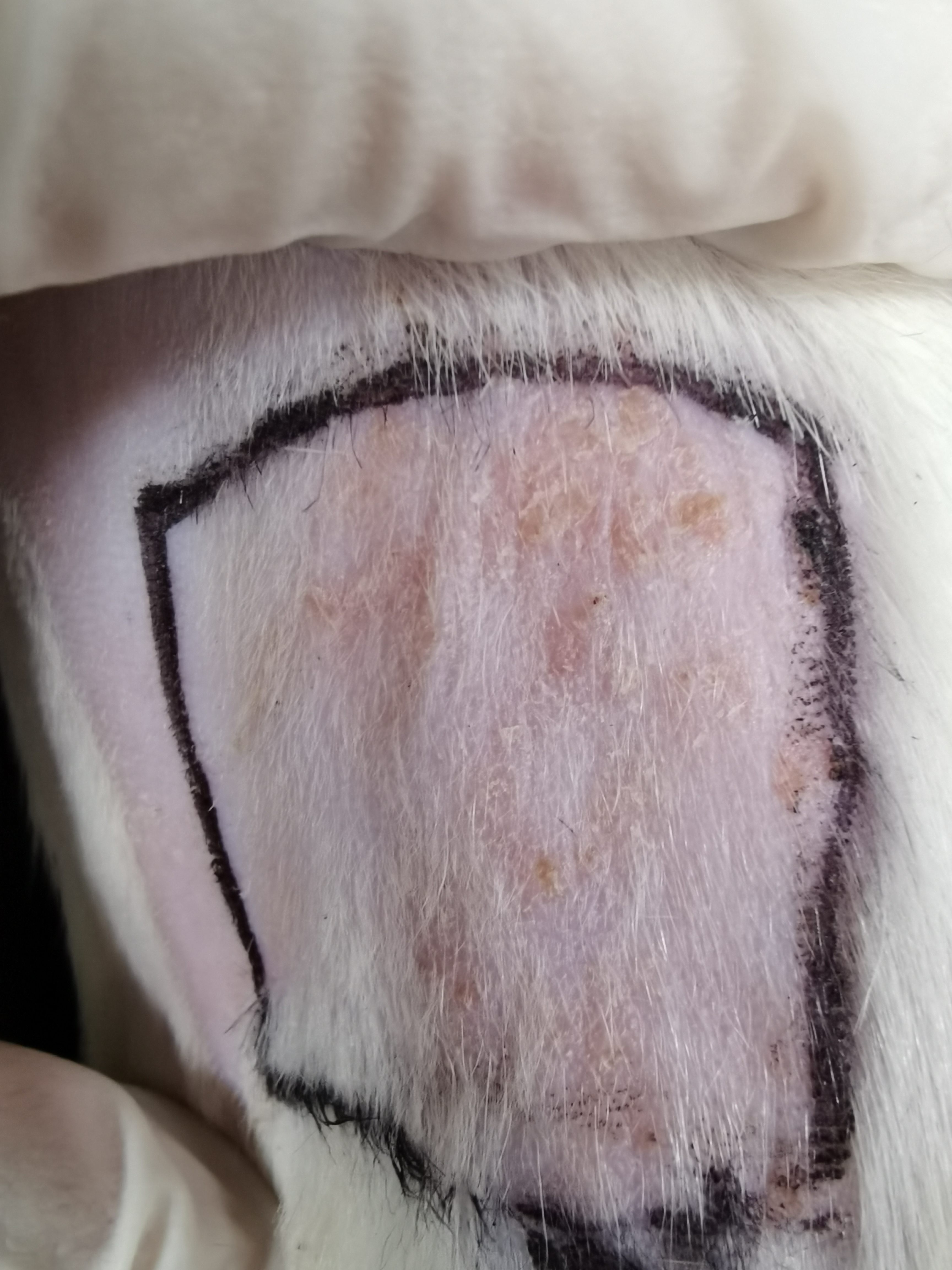

Supplement: S3 File — (ZIP) [file pone.0330078.s003.zip › Animal experiment/HAMCC/7d 3.jpg]

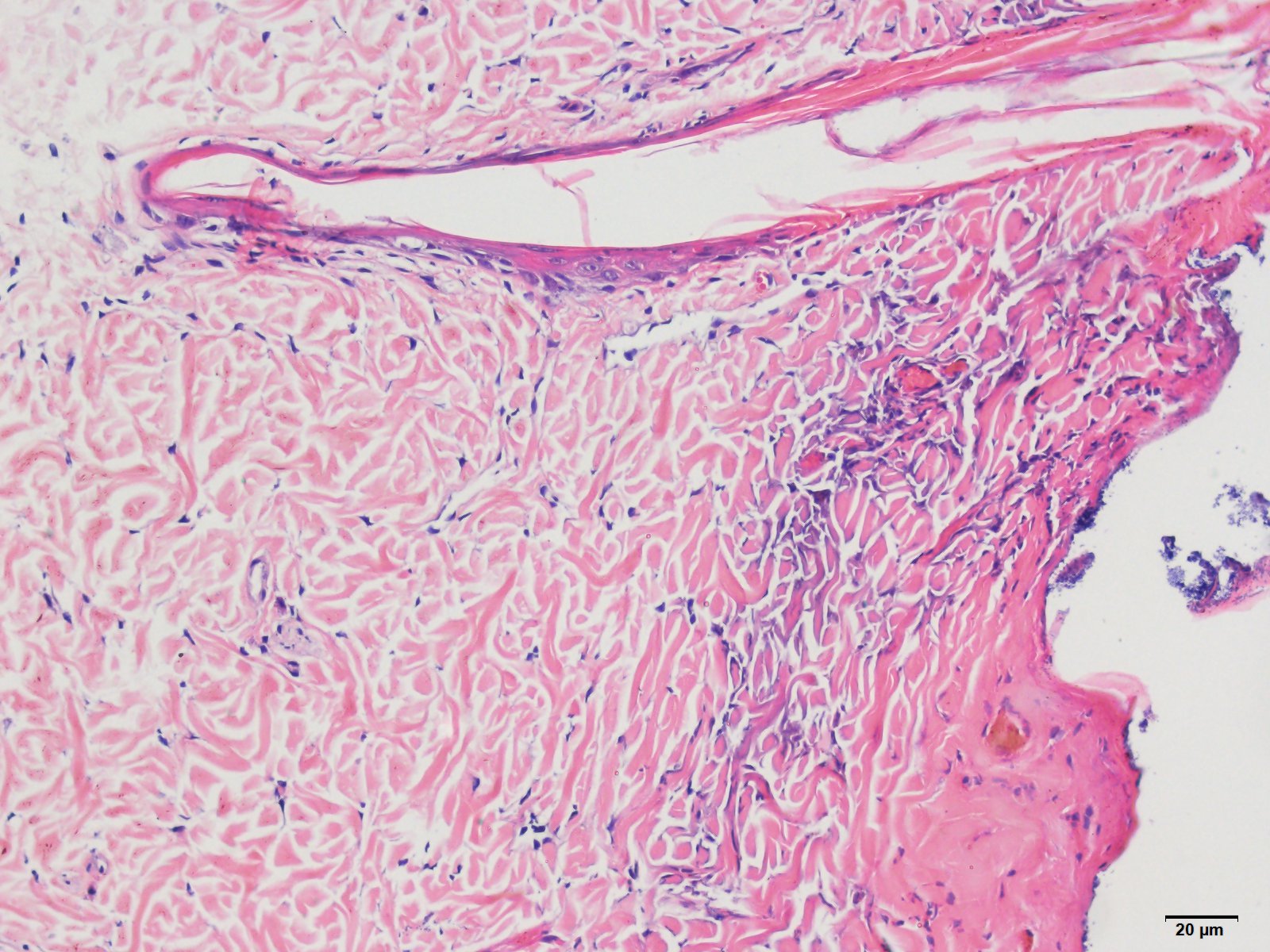

Supplement: S4 File — (ZIP) [file pone.0330078.s004.zip › HE staining/14d CGF 1.jpg]

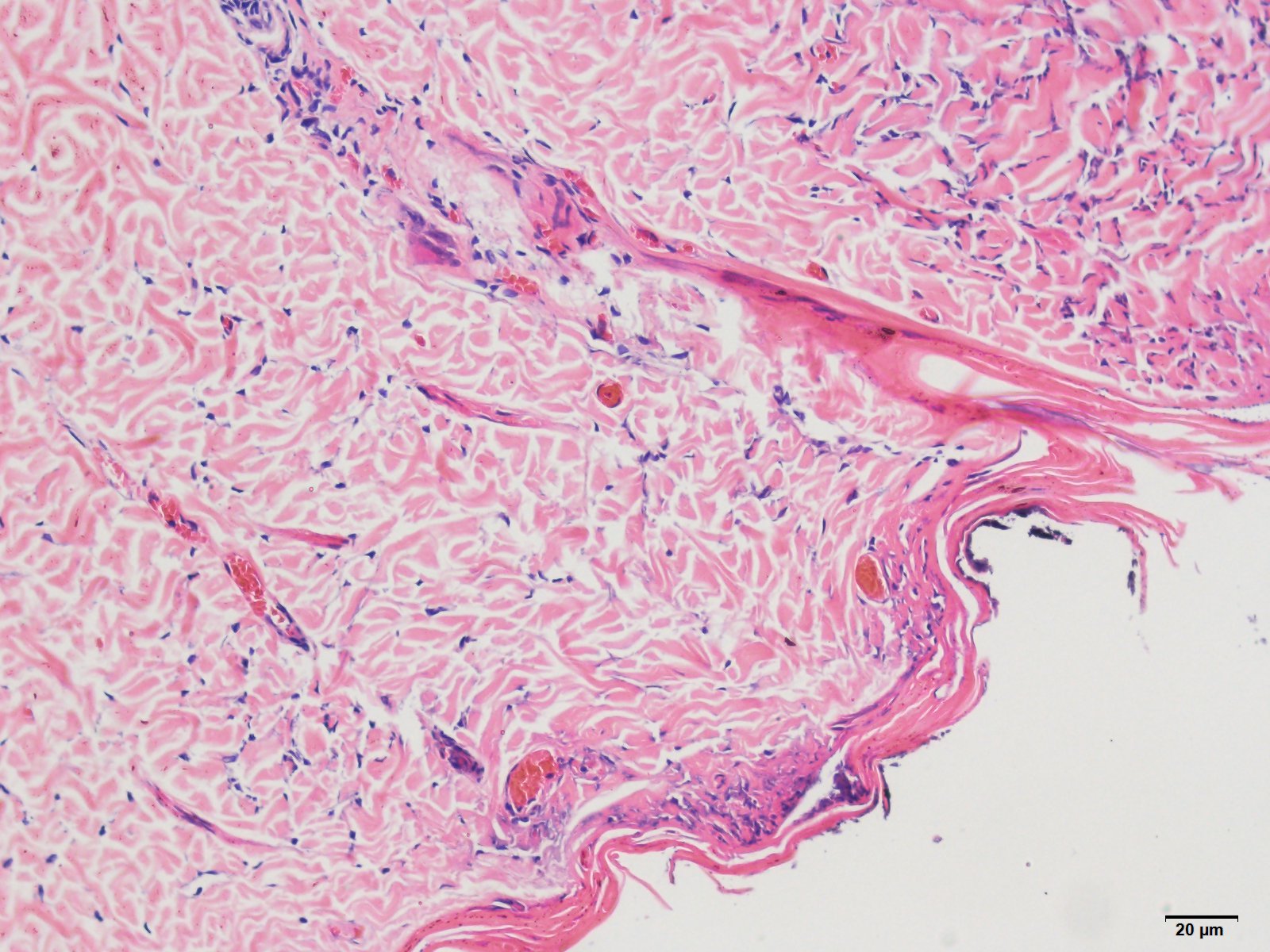

Supplement: S4 File — (ZIP) [file pone.0330078.s004.zip › HE staining/14d CGF 2.jpg]

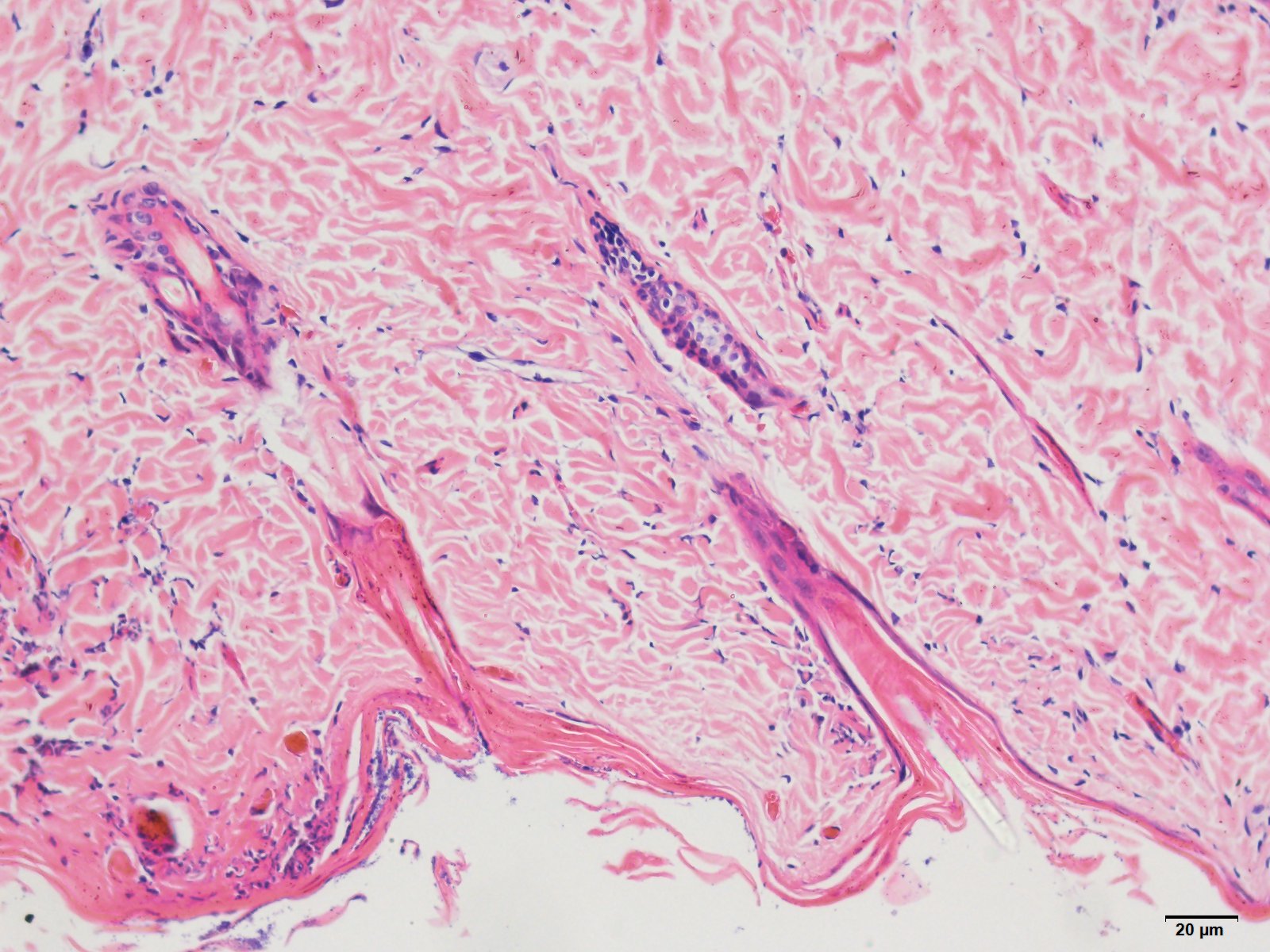

Supplement: S4 File — (ZIP) [file pone.0330078.s004.zip › HE staining/14d CGF 3.jpg]

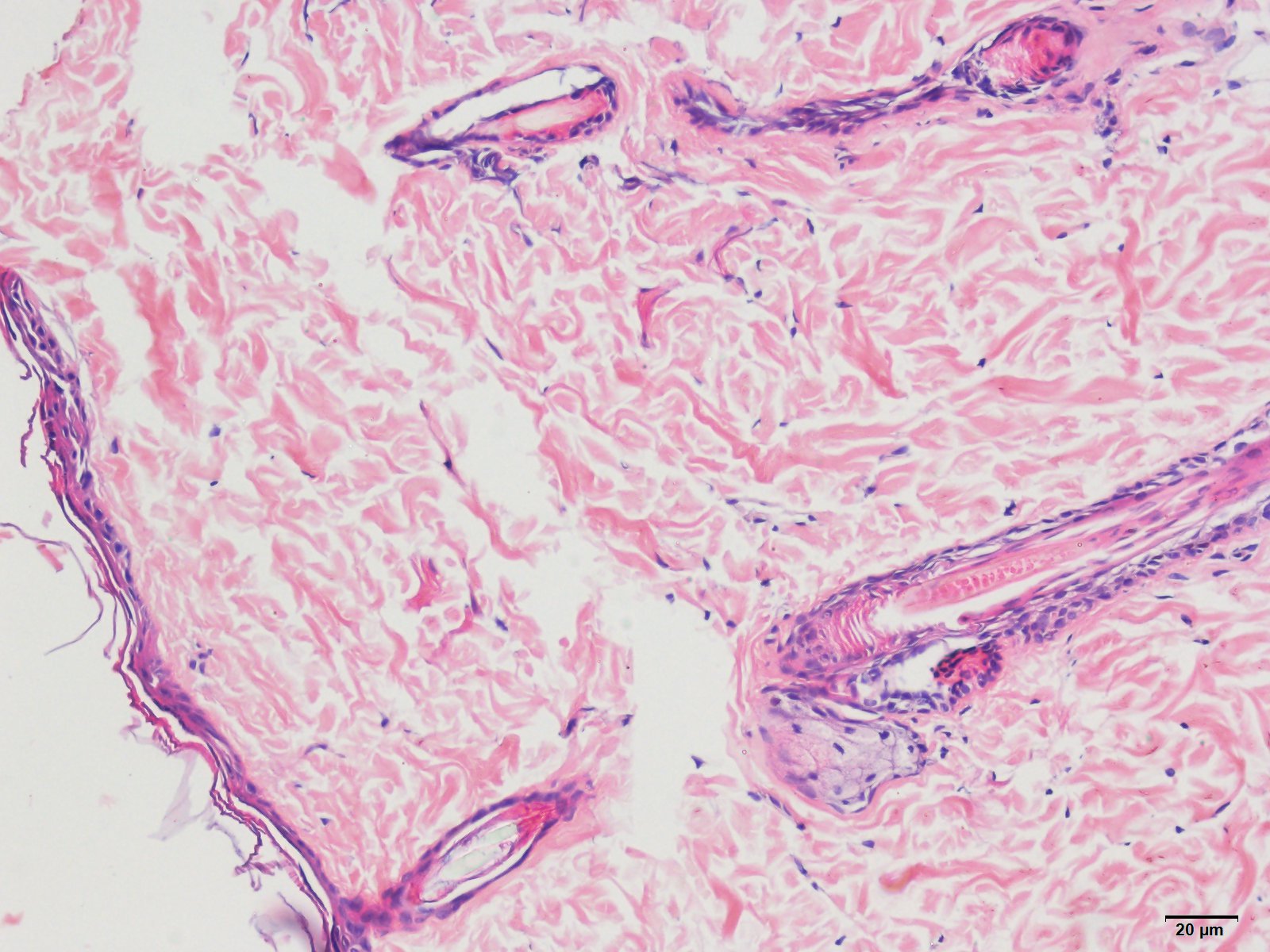

Supplement: S4 File — (ZIP) [file pone.0330078.s004.zip › HE staining/14d CGF+HAMCC 1.jpg]

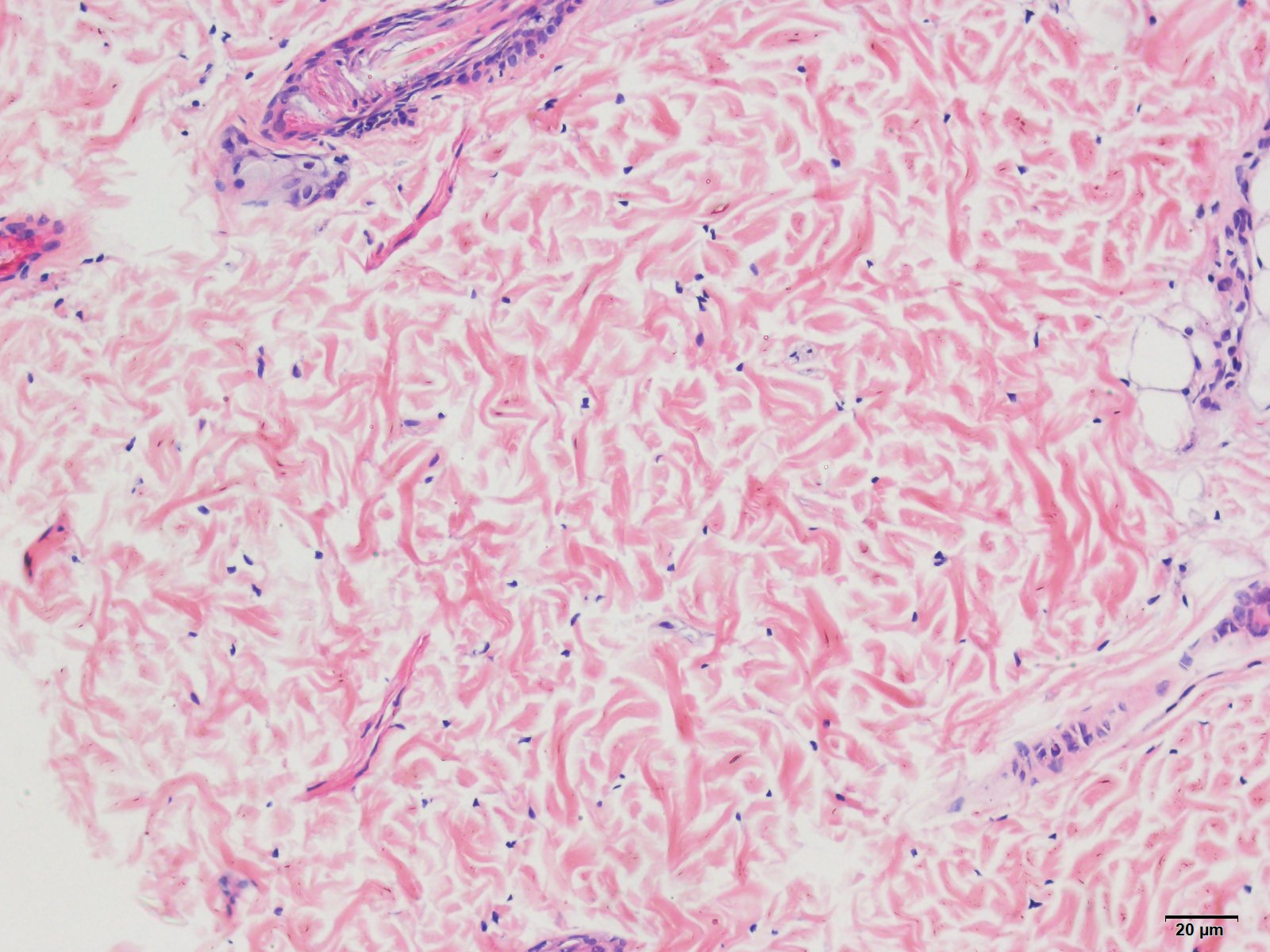

Supplement: S4 File — (ZIP) [file pone.0330078.s004.zip › HE staining/14d CGF+HAMCC 2.jpg]

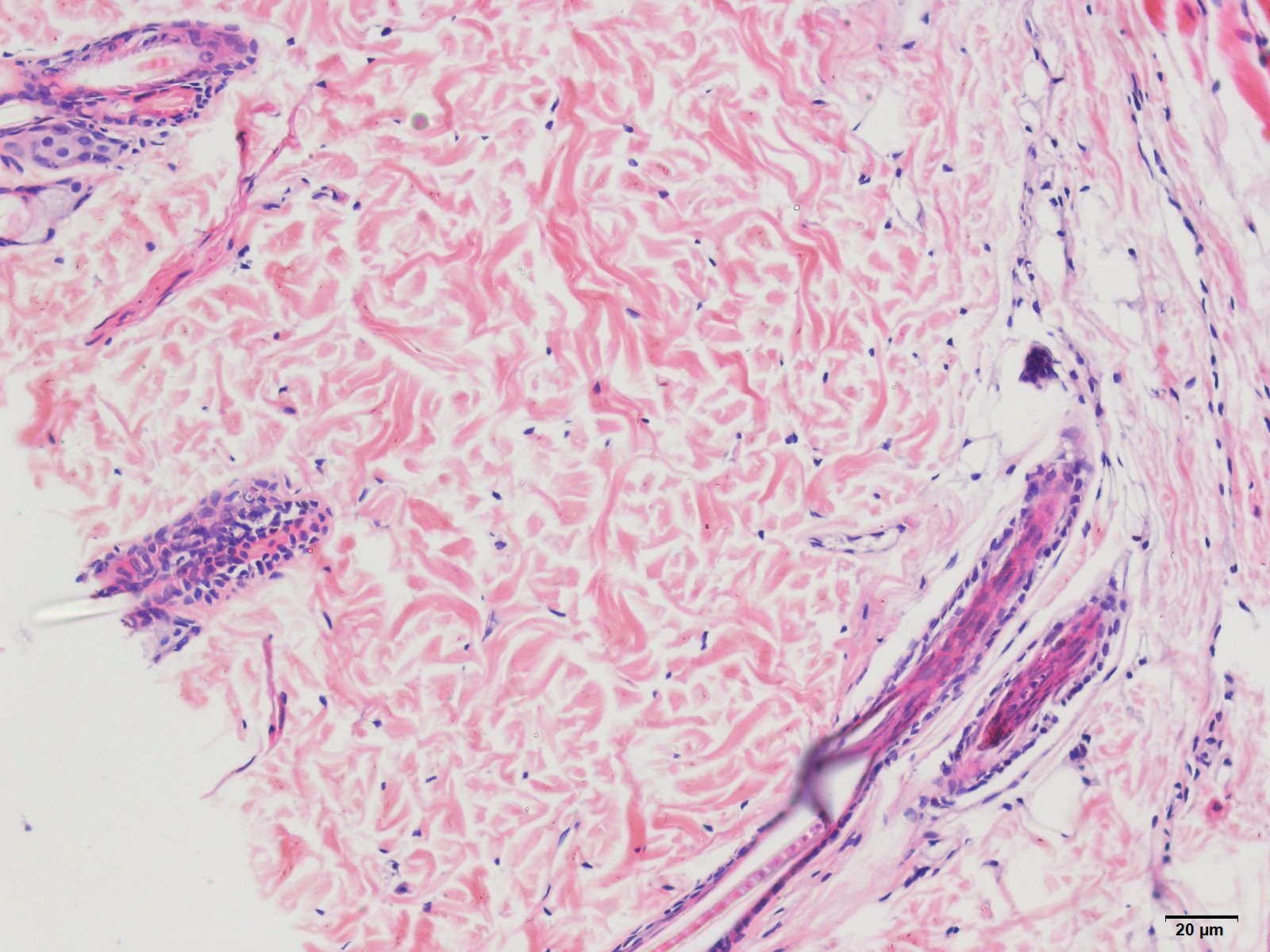

Supplement: S4 File — (ZIP) [file pone.0330078.s004.zip › HE staining/14d CGF+HAMCC 3.jpg]

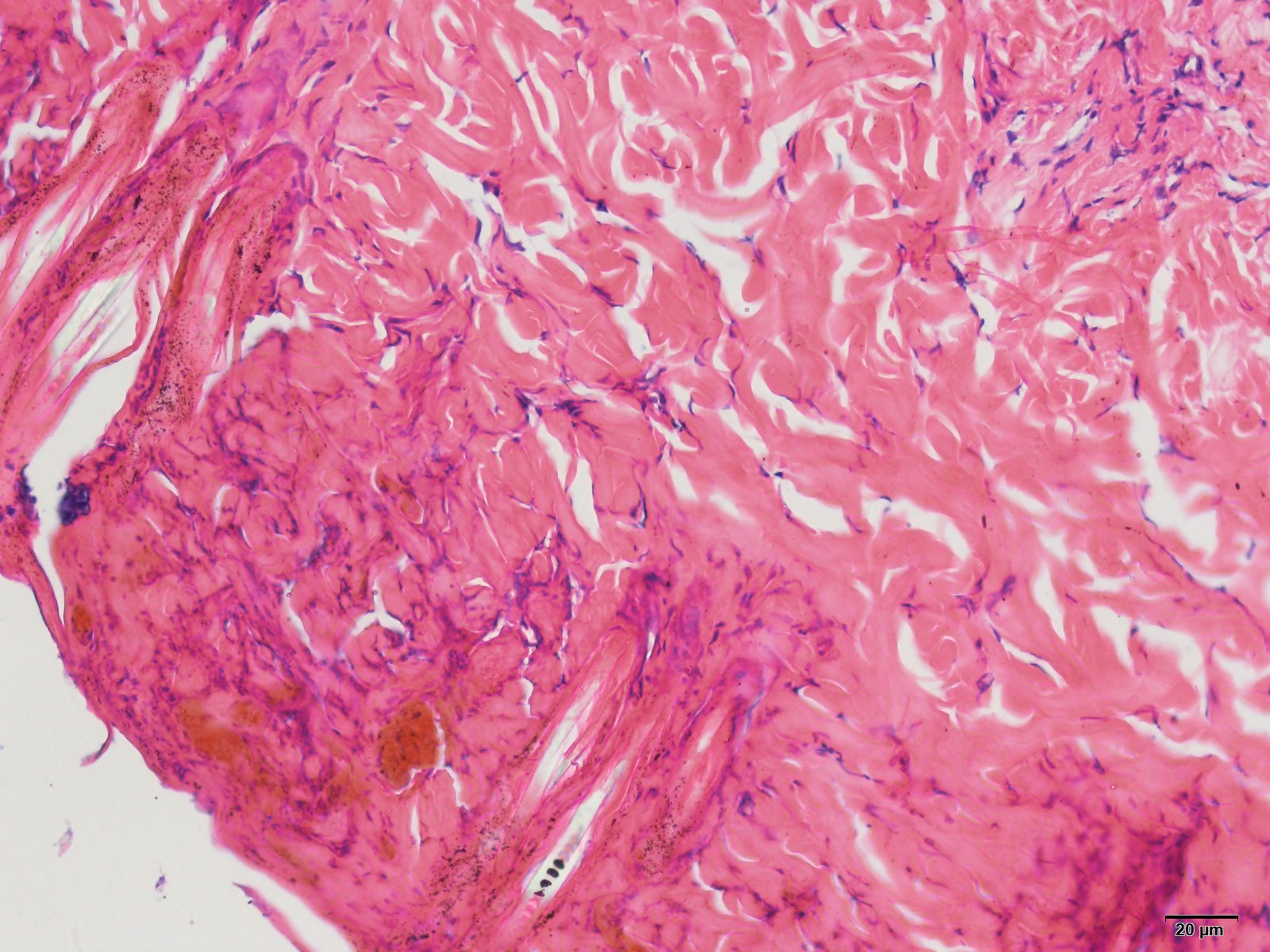

Supplement: S4 File — (ZIP) [file pone.0330078.s004.zip › HE staining/14d Control 1.jpg]
