# Supplementary figures and images for: Efficacy and mechanism of high-purity HAMCC combined with CGF in promoting the repair of radiation-induced skin and soft tissue damage (part 2 of 3)
Source: PLoS One. 2025 Sep 9;20(9):e0330078. doi: 10.1371/journal.pone.0330078 (PMC12419615; doi:10.1371/journal.pone.0330078)

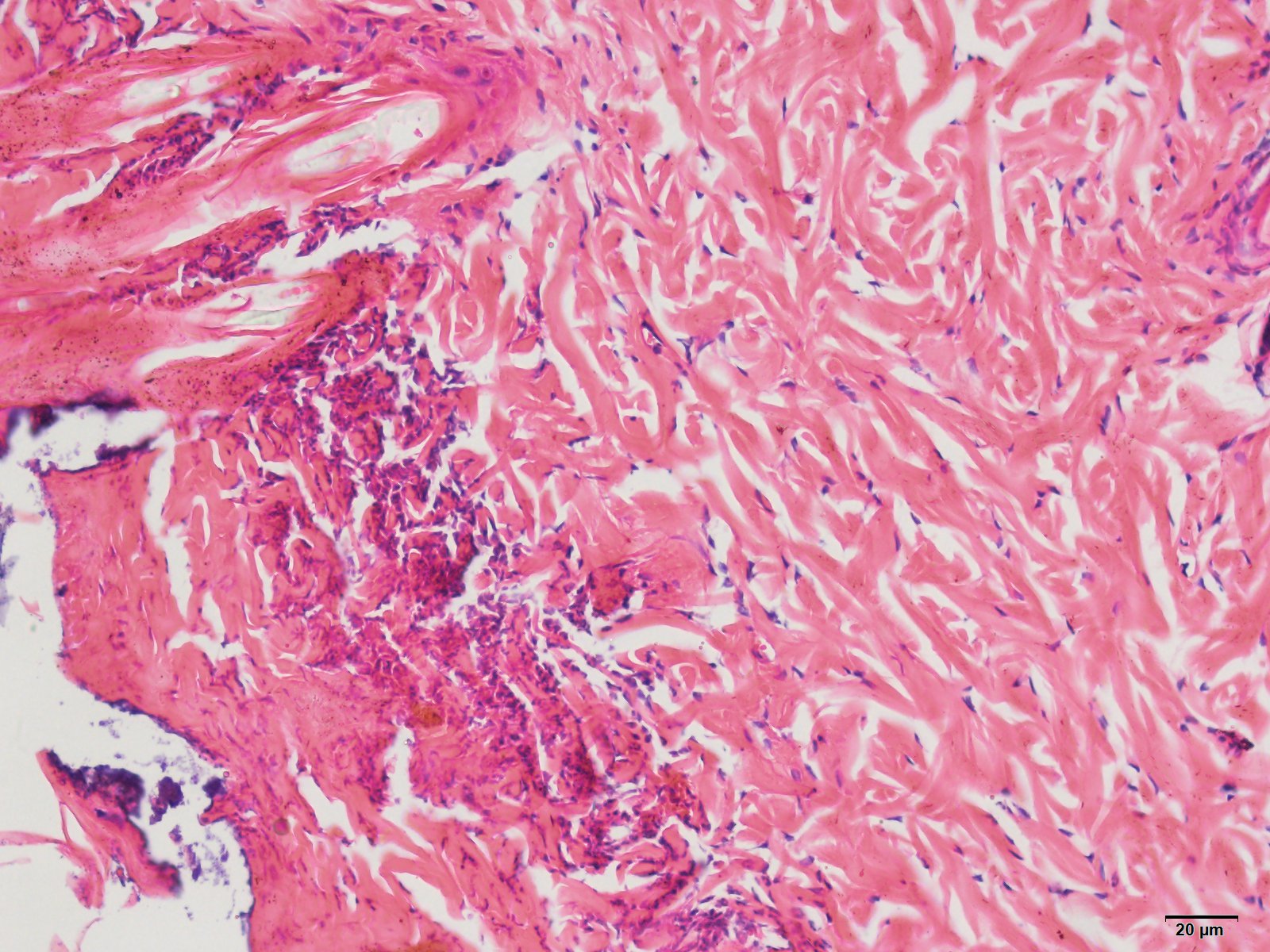

Supplement: S4 File — (ZIP) [file pone.0330078.s004.zip › HE staining/14d Control 2.jpg]

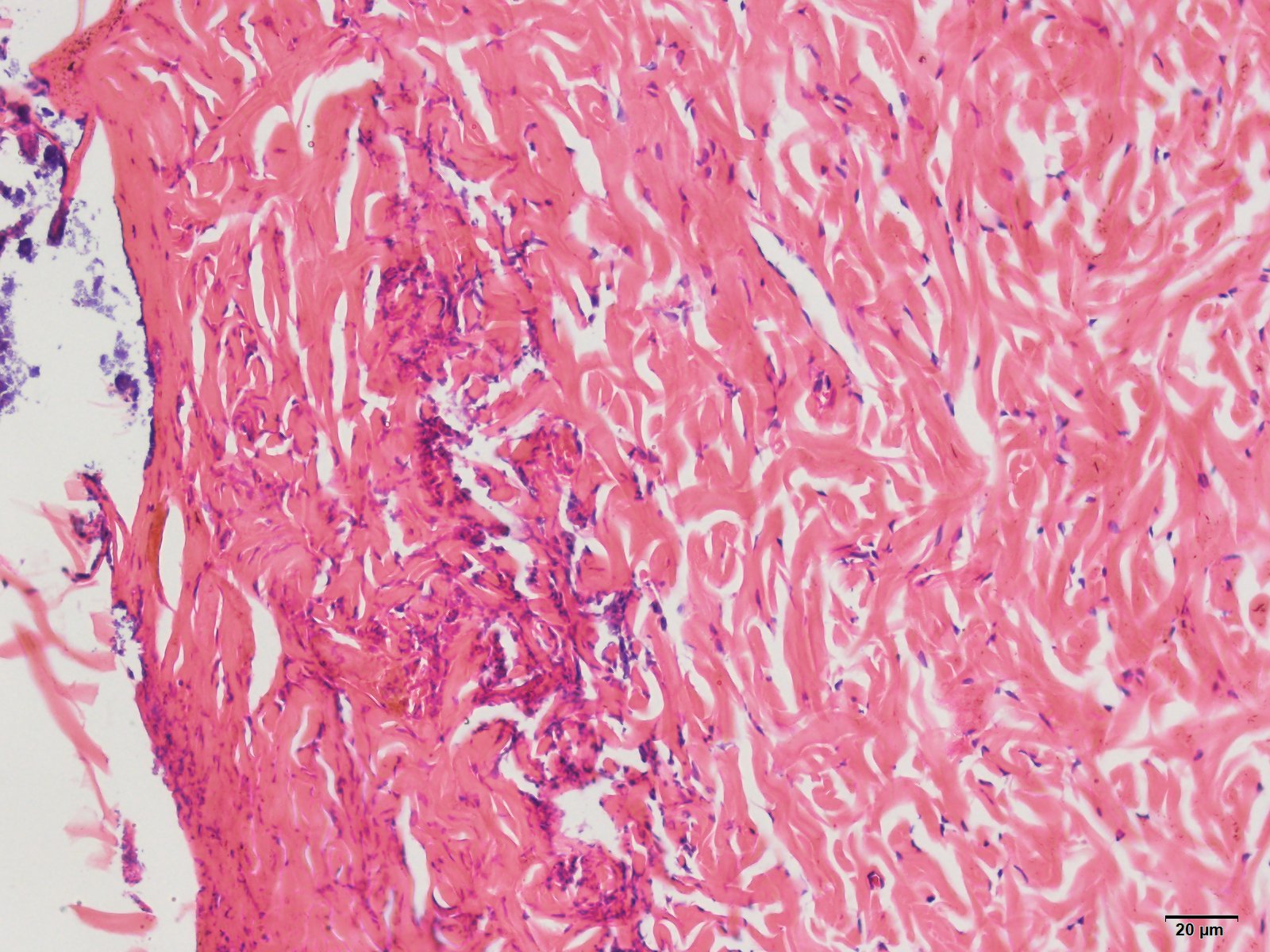

Supplement: S4 File — (ZIP) [file pone.0330078.s004.zip › HE staining/14d Control 3.jpg]

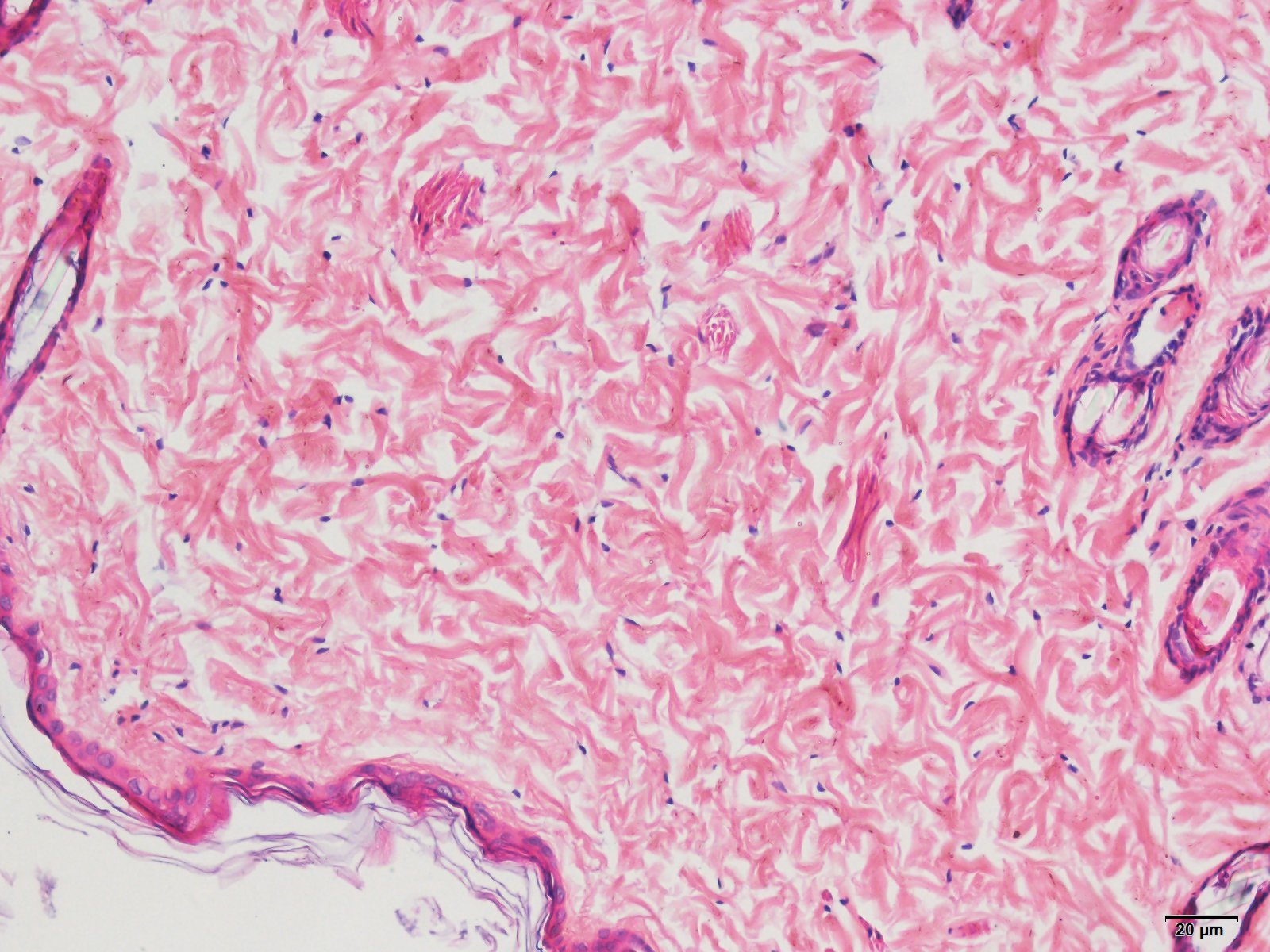

Supplement: S4 File — (ZIP) [file pone.0330078.s004.zip › HE staining/14d HAMCC 1.jpg]

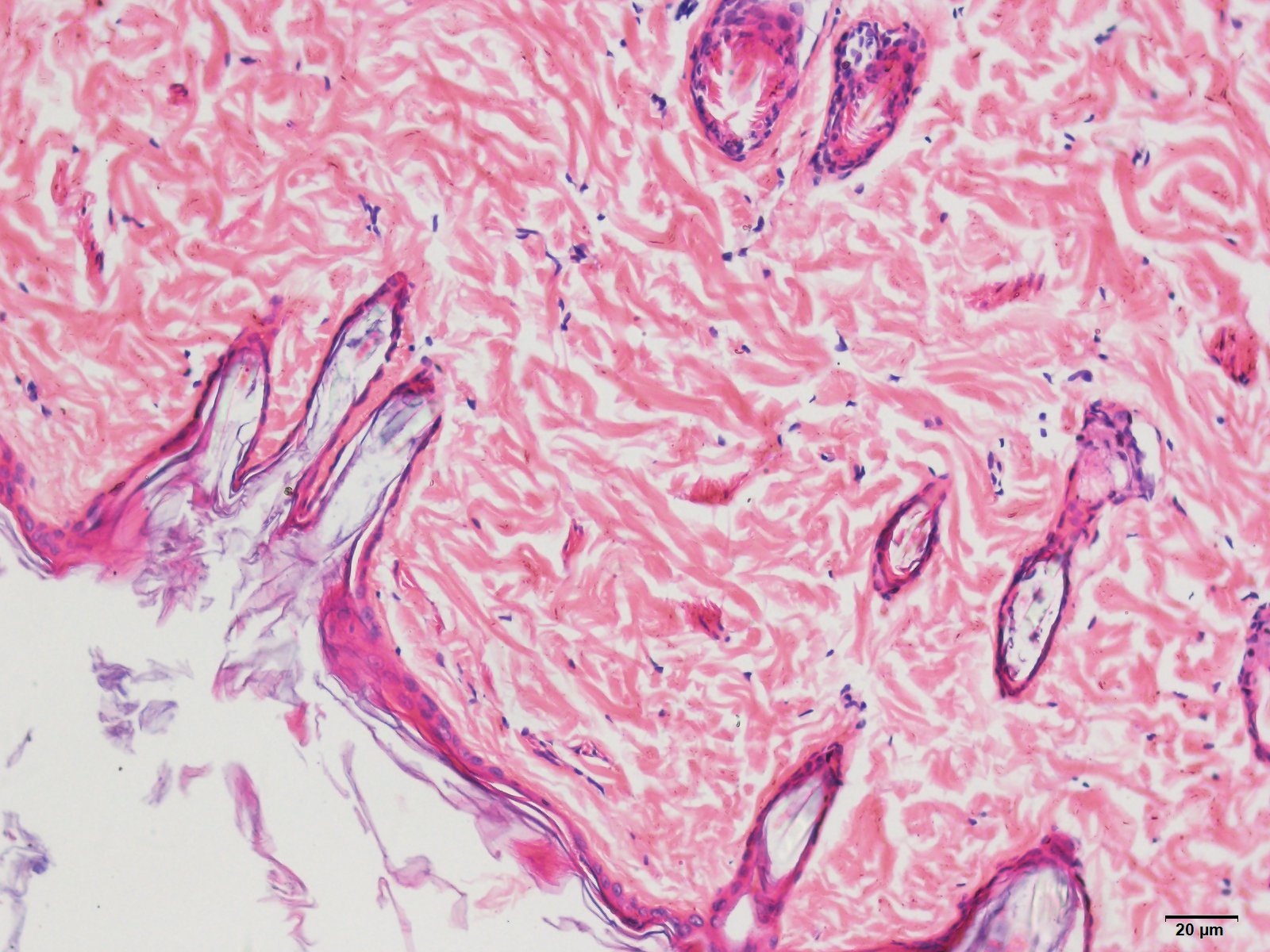

Supplement: S4 File — (ZIP) [file pone.0330078.s004.zip › HE staining/14d HAMCC 2.jpg]

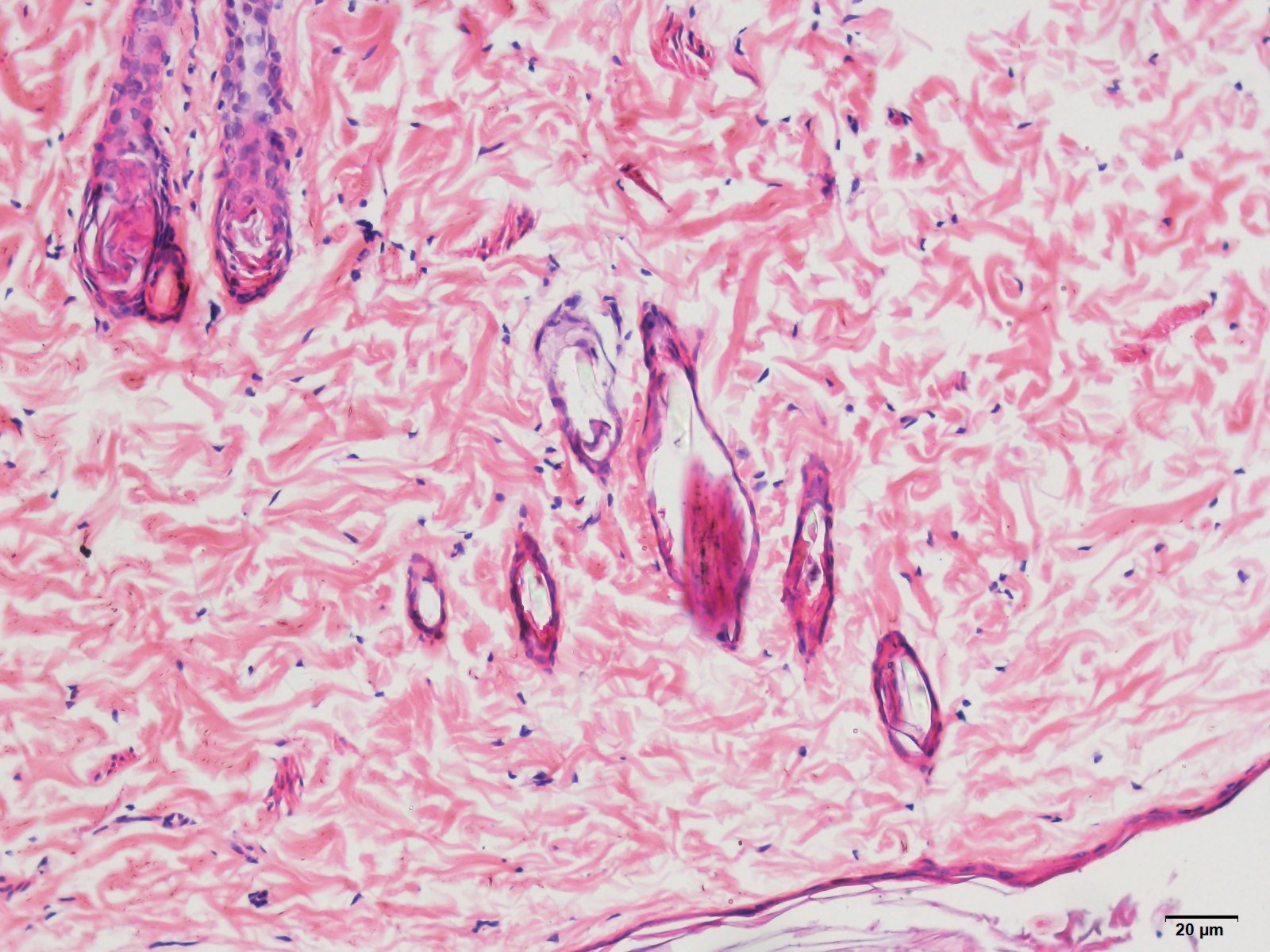

Supplement: S4 File — (ZIP) [file pone.0330078.s004.zip › HE staining/14d HAMCC 3.jpg]

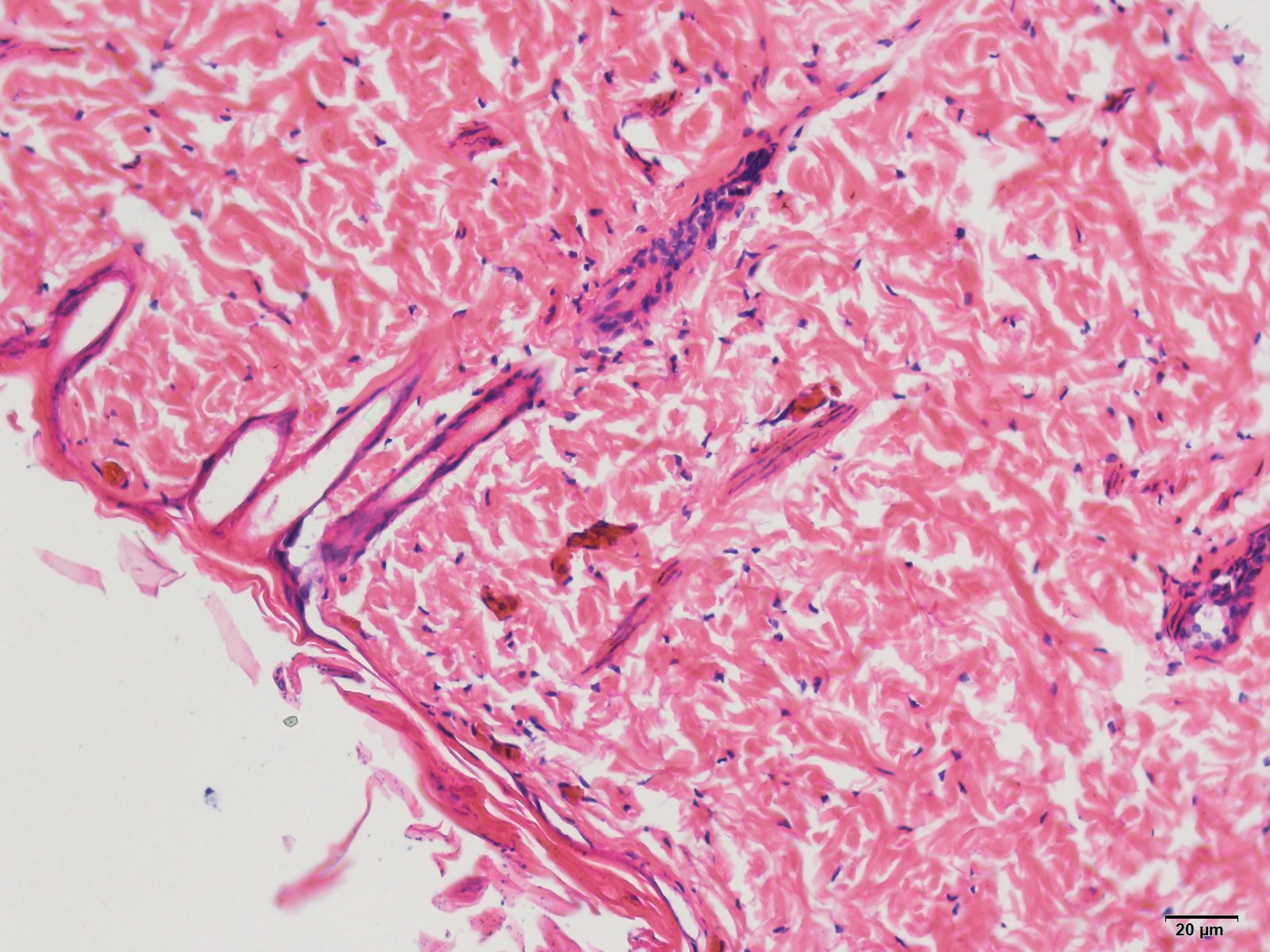

Supplement: S4 File — (ZIP) [file pone.0330078.s004.zip › HE staining/28d CGF 1.jpg]

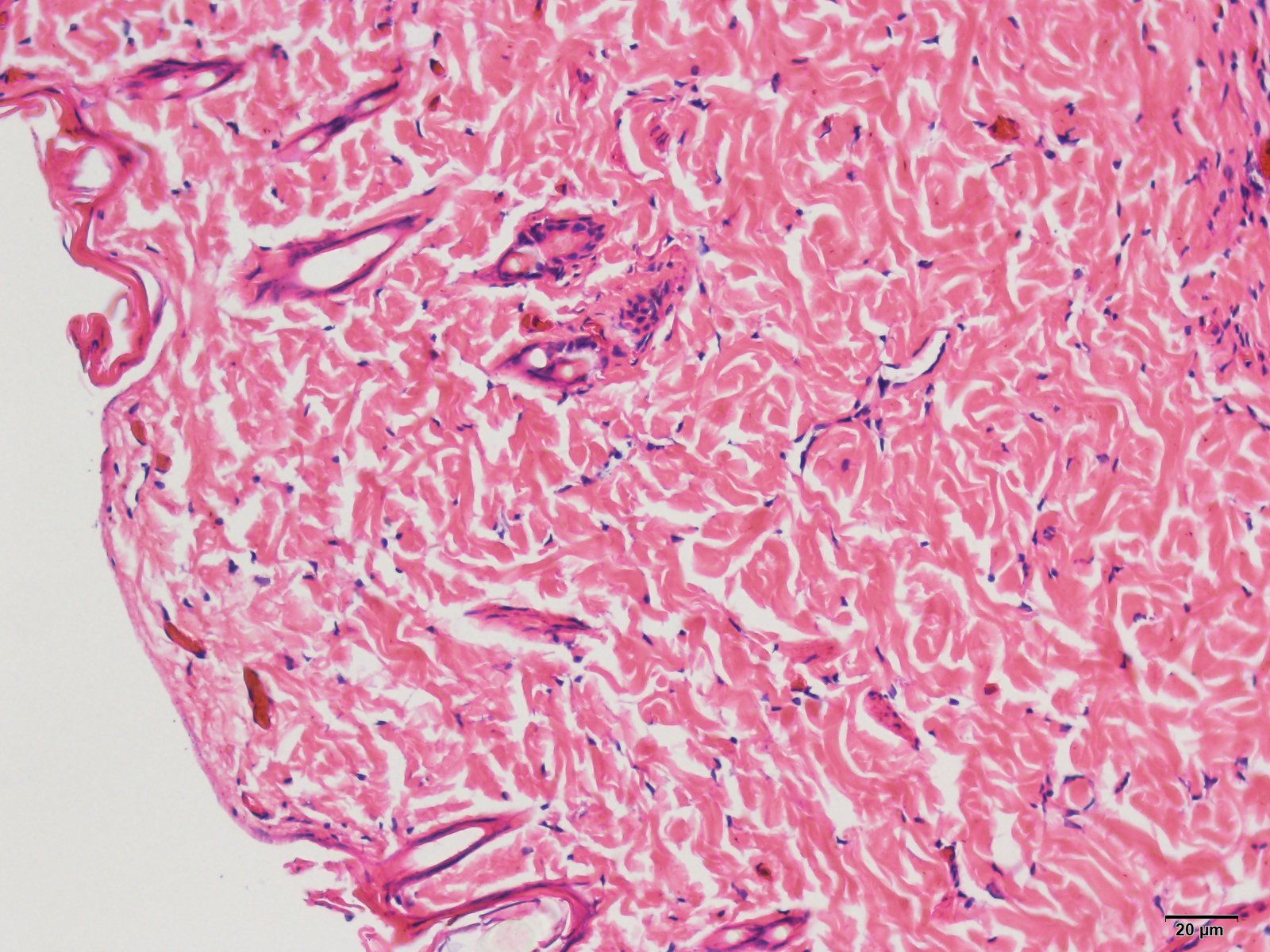

Supplement: S4 File — (ZIP) [file pone.0330078.s004.zip › HE staining/28d CGF 2.jpg]

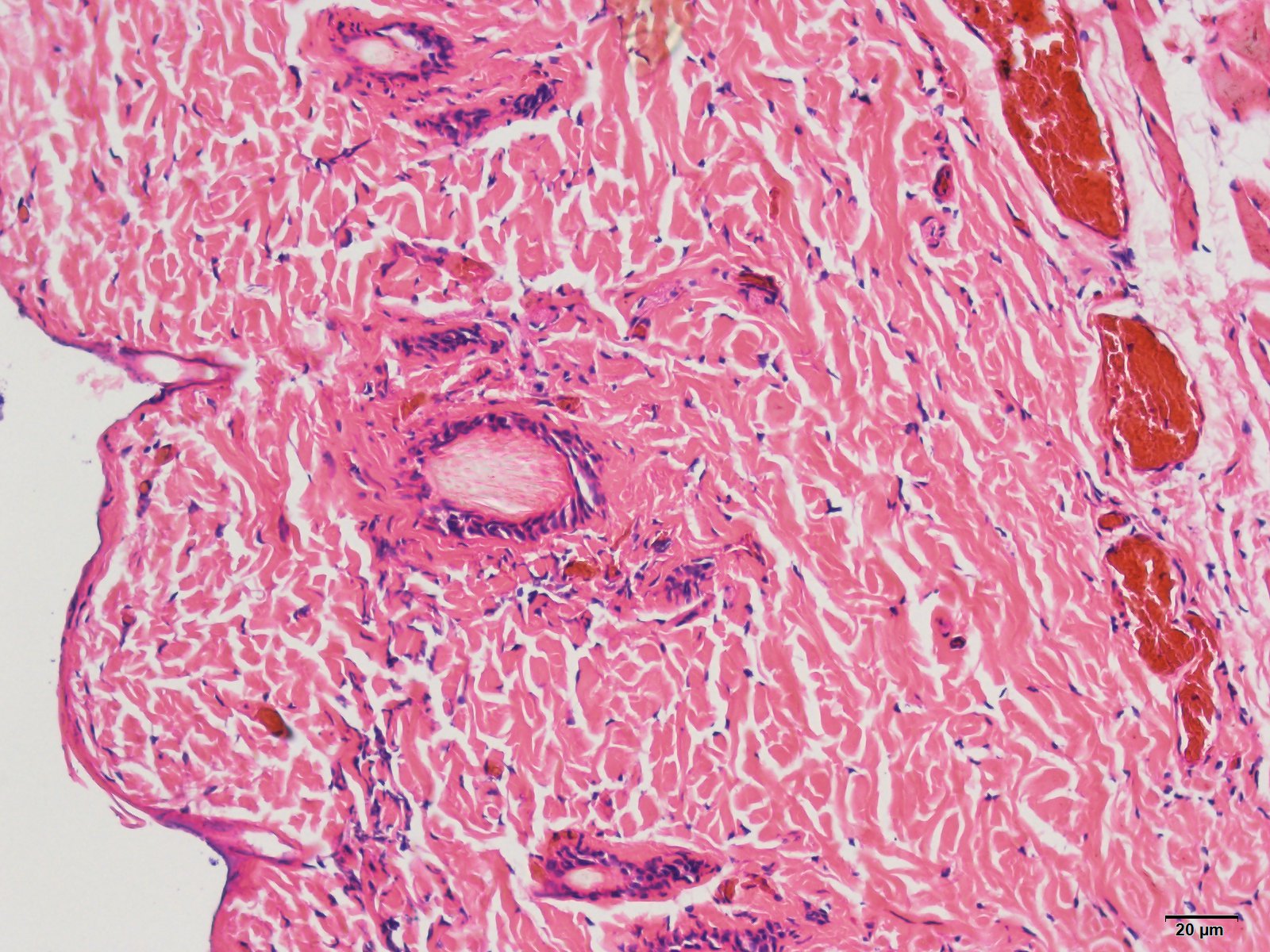

Supplement: S4 File — (ZIP) [file pone.0330078.s004.zip › HE staining/28d CGF 3.jpg]

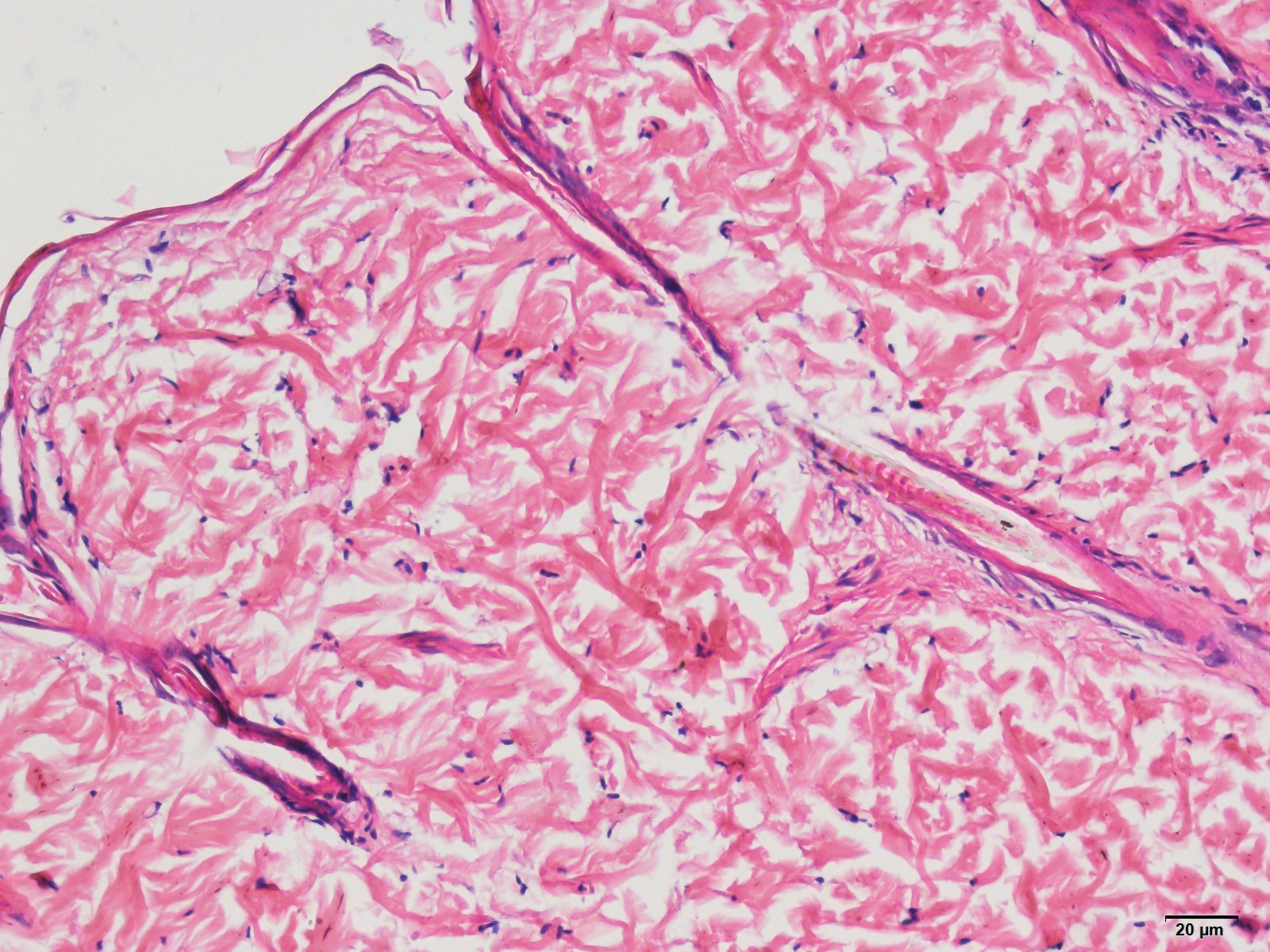

Supplement: S4 File — (ZIP) [file pone.0330078.s004.zip › HE staining/28d CGF+HAMCC 1.jpg]

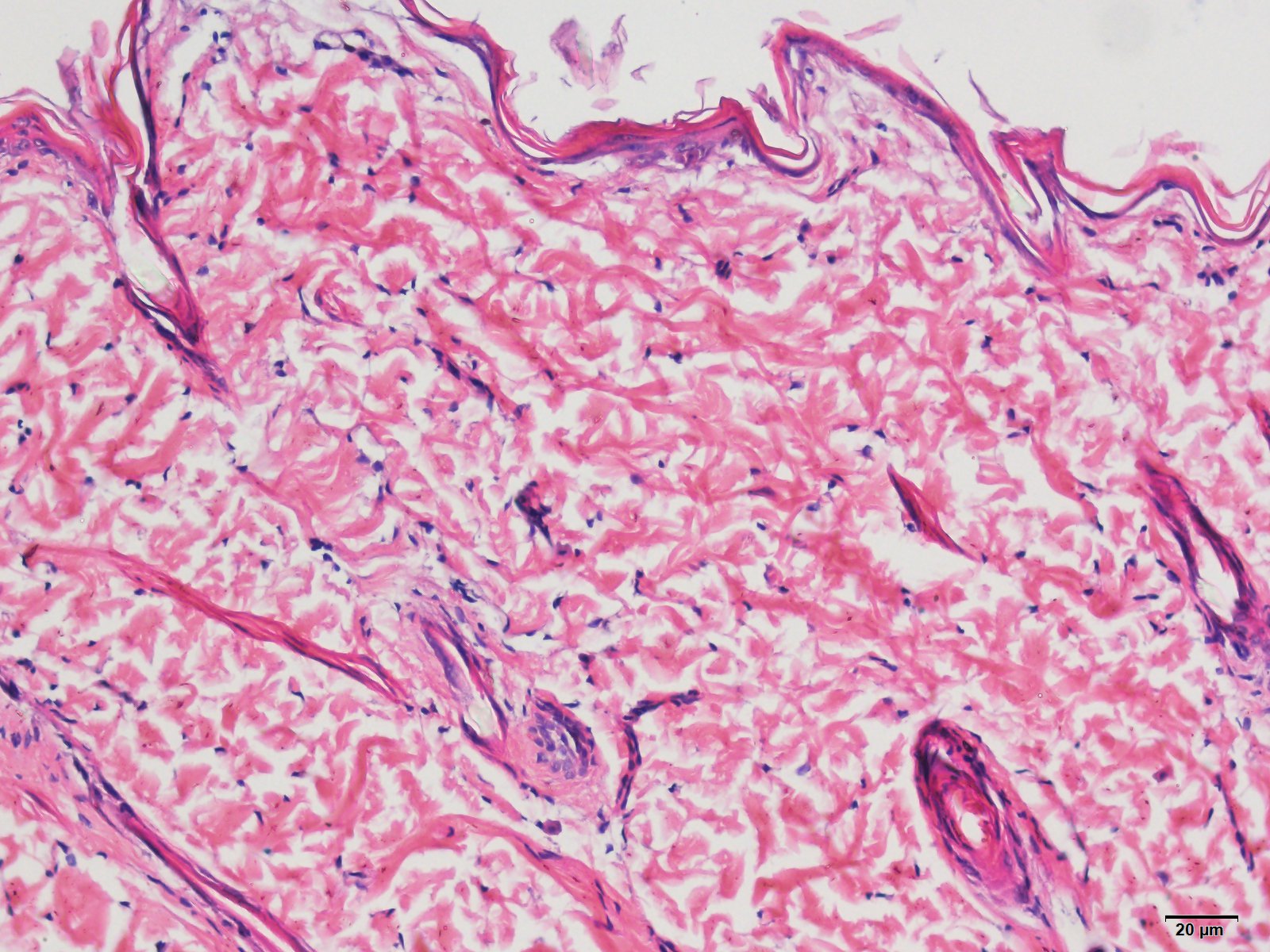

Supplement: S4 File — (ZIP) [file pone.0330078.s004.zip › HE staining/28d CGF+HAMCC 2.jpg]

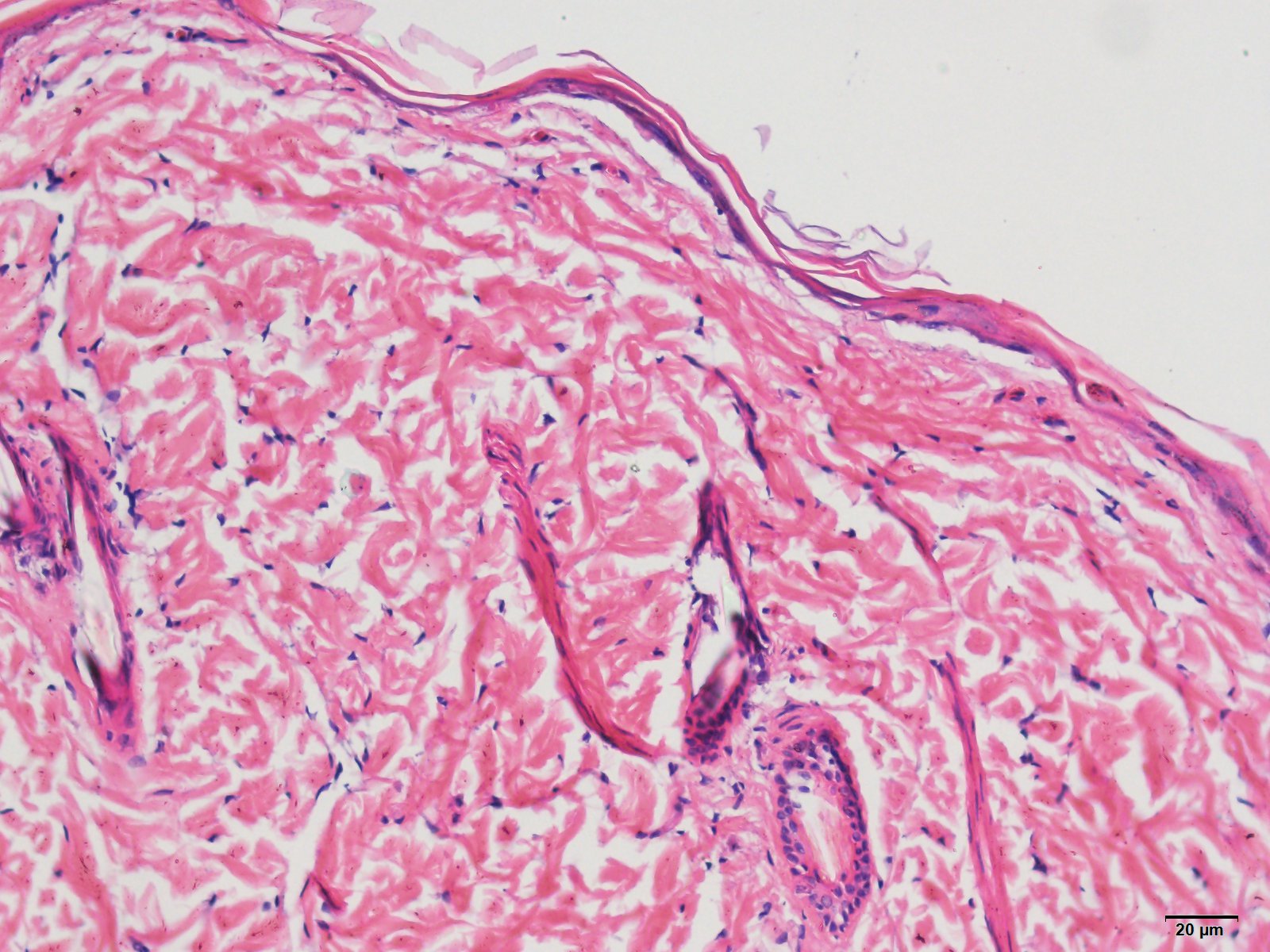

Supplement: S4 File — (ZIP) [file pone.0330078.s004.zip › HE staining/28d CGF+HAMCC 3.jpg]

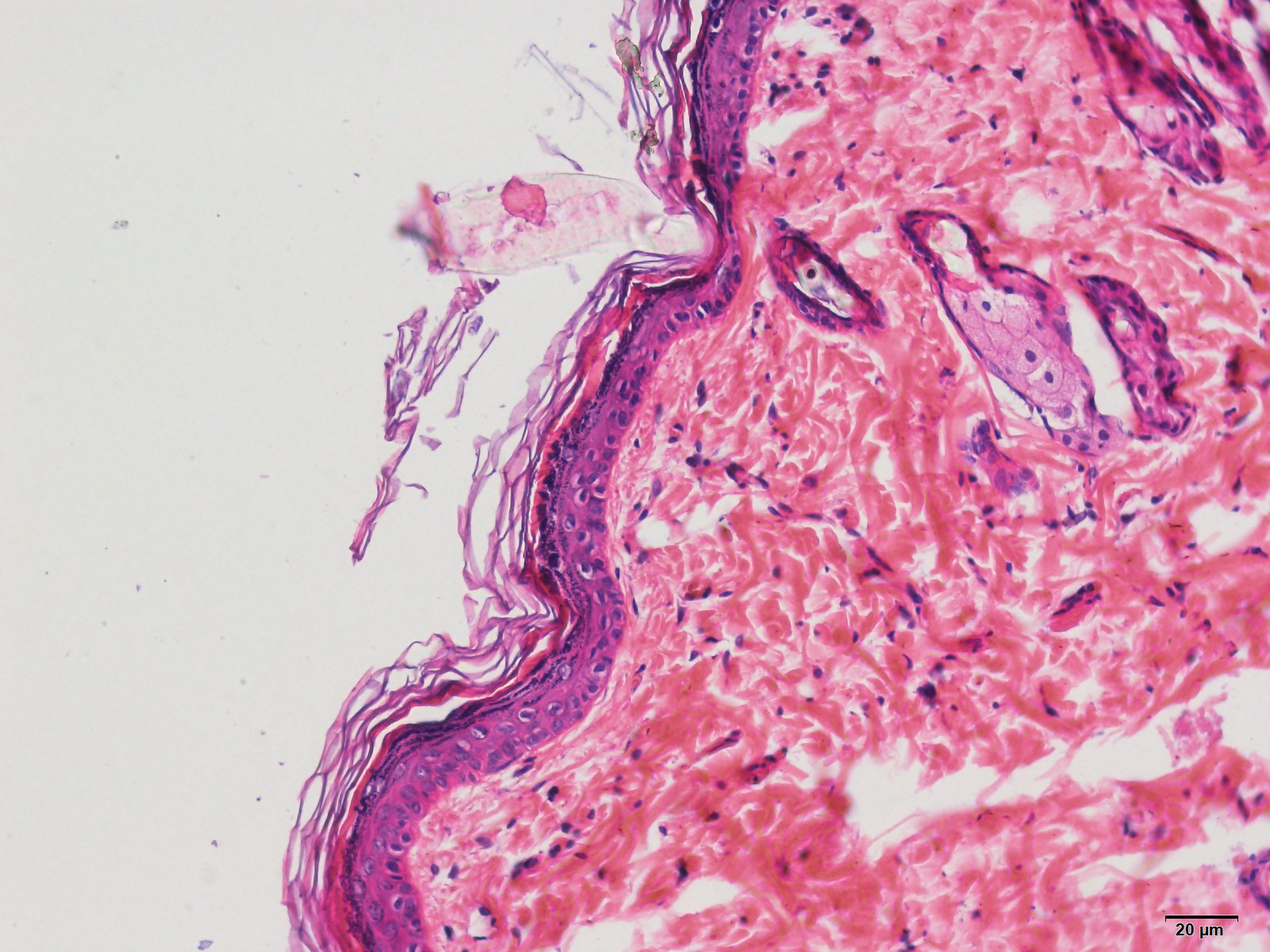

Supplement: S4 File — (ZIP) [file pone.0330078.s004.zip › HE staining/28d Control 1.jpg]

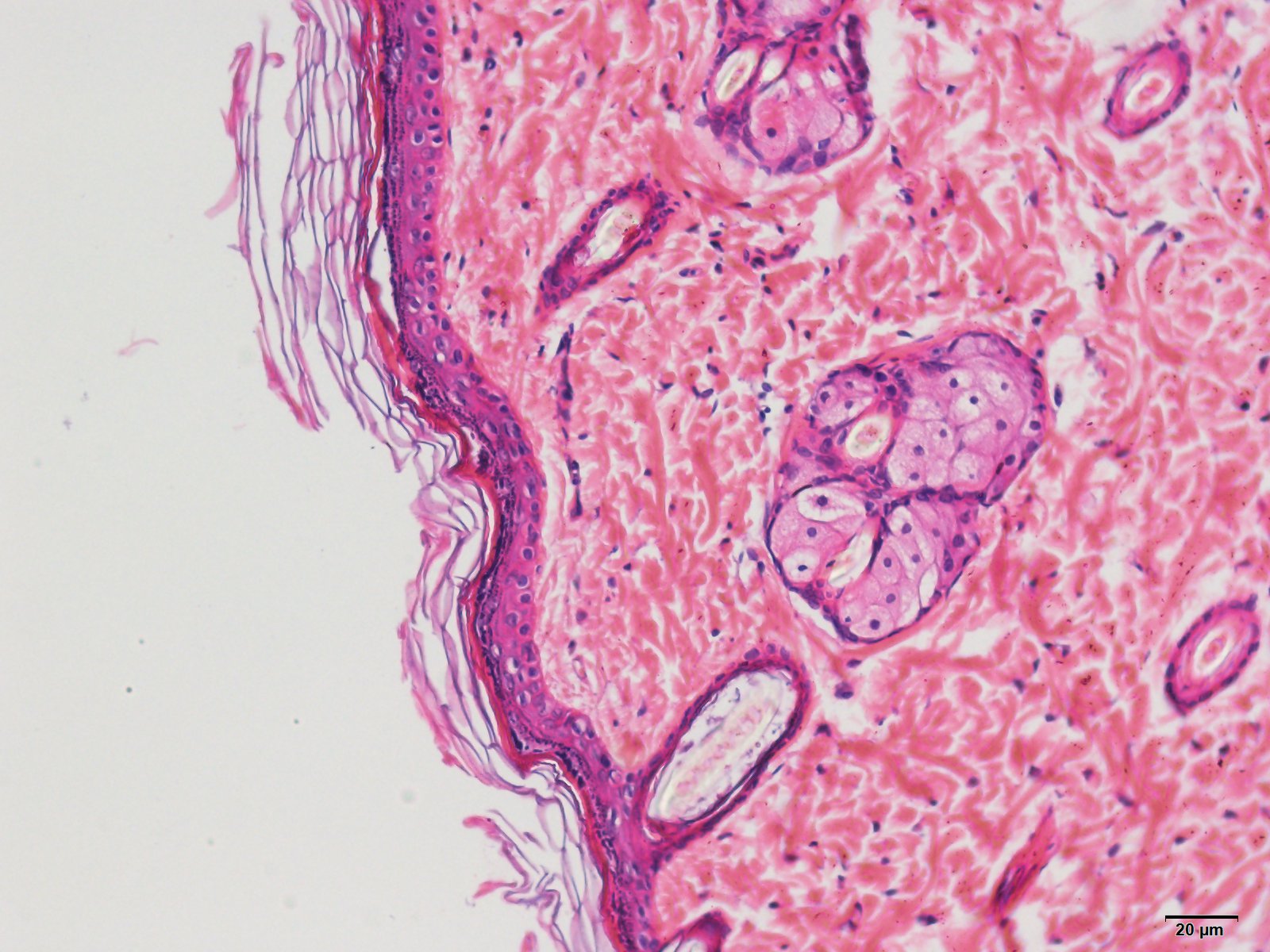

Supplement: S4 File — (ZIP) [file pone.0330078.s004.zip › HE staining/28d Control 2.jpg]

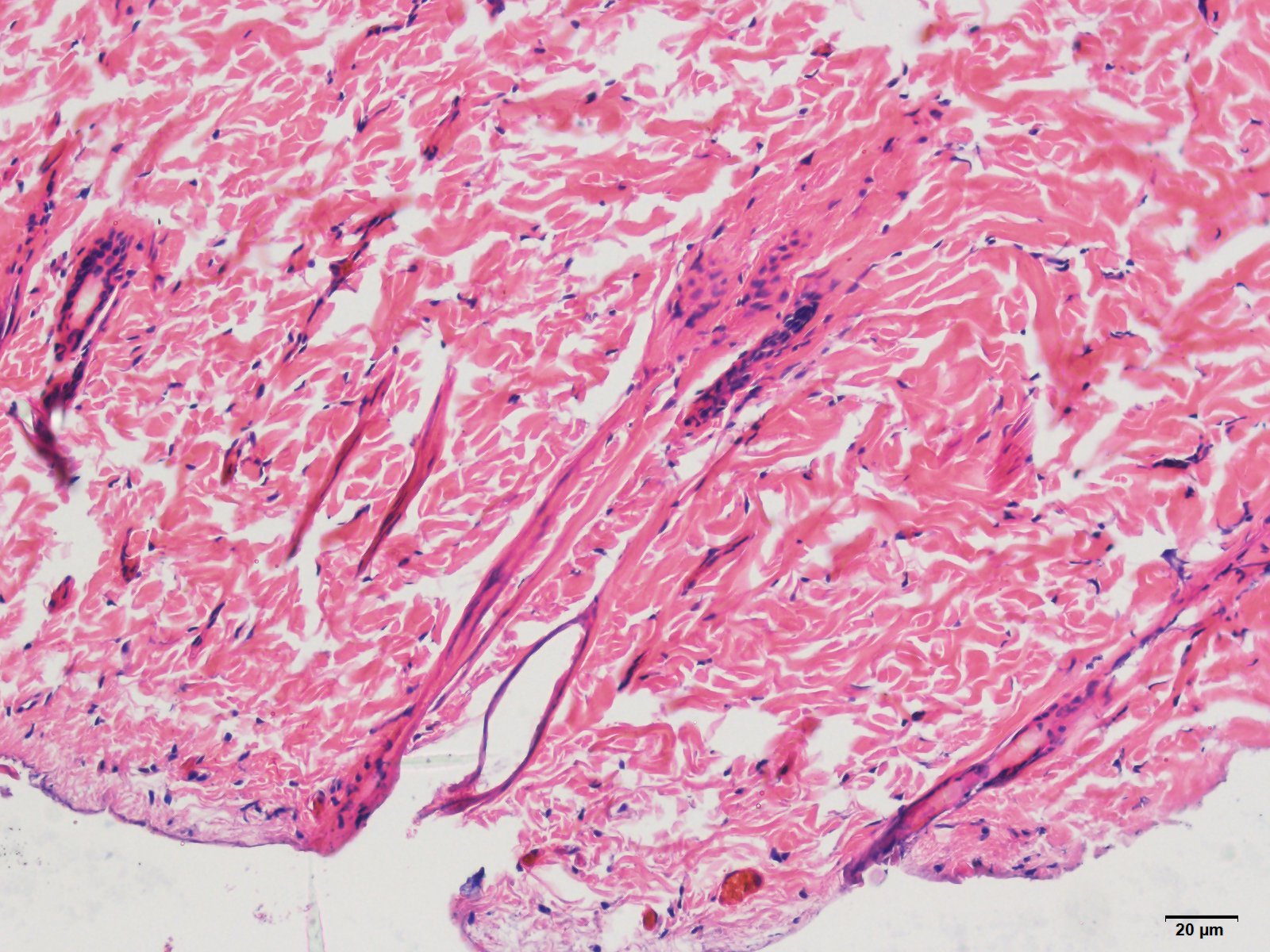

Supplement: S4 File — (ZIP) [file pone.0330078.s004.zip › HE staining/28d HAMCC 1.jpg]

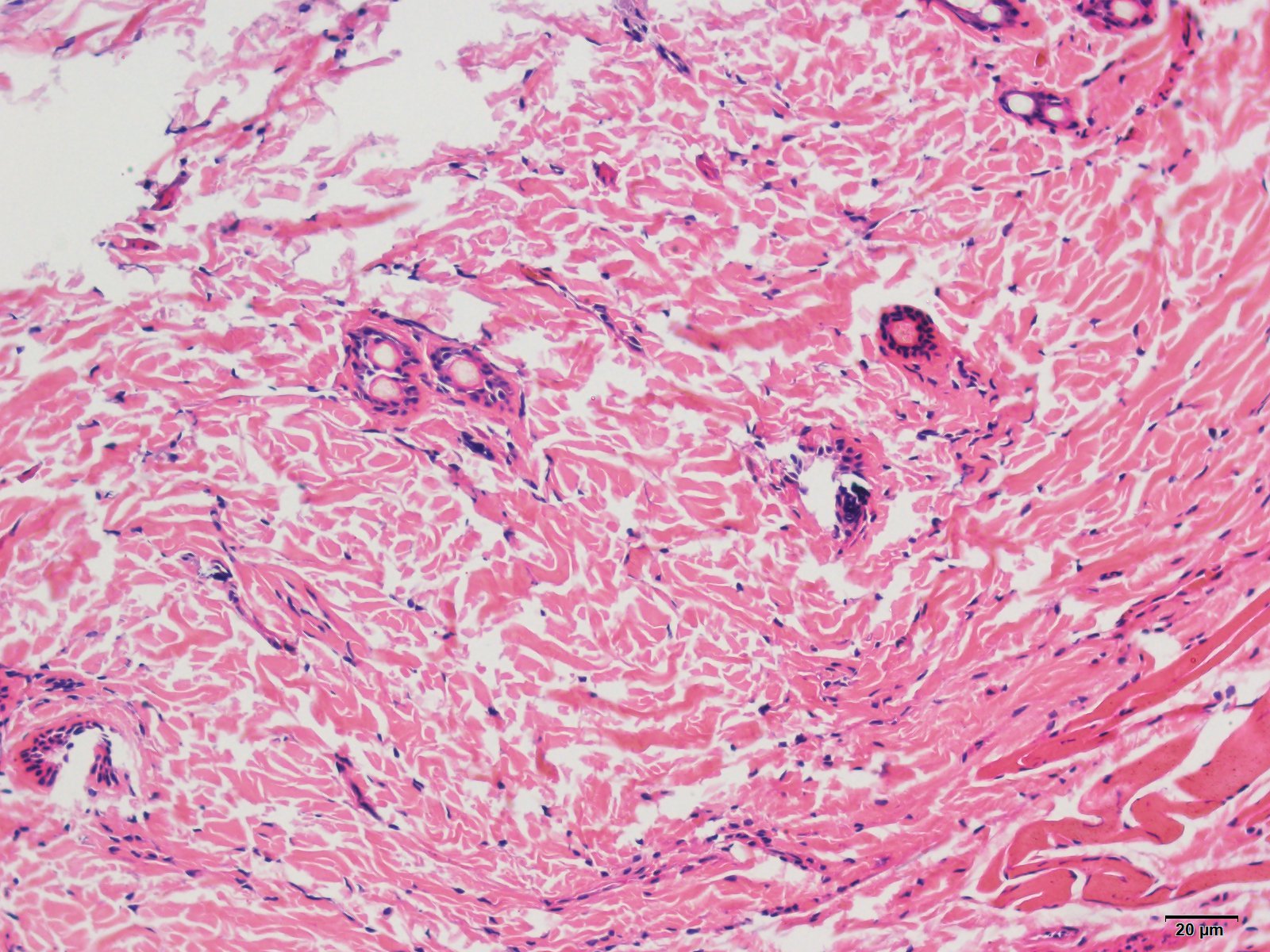

Supplement: S4 File — (ZIP) [file pone.0330078.s004.zip › HE staining/28d HAMCC 2.jpg]

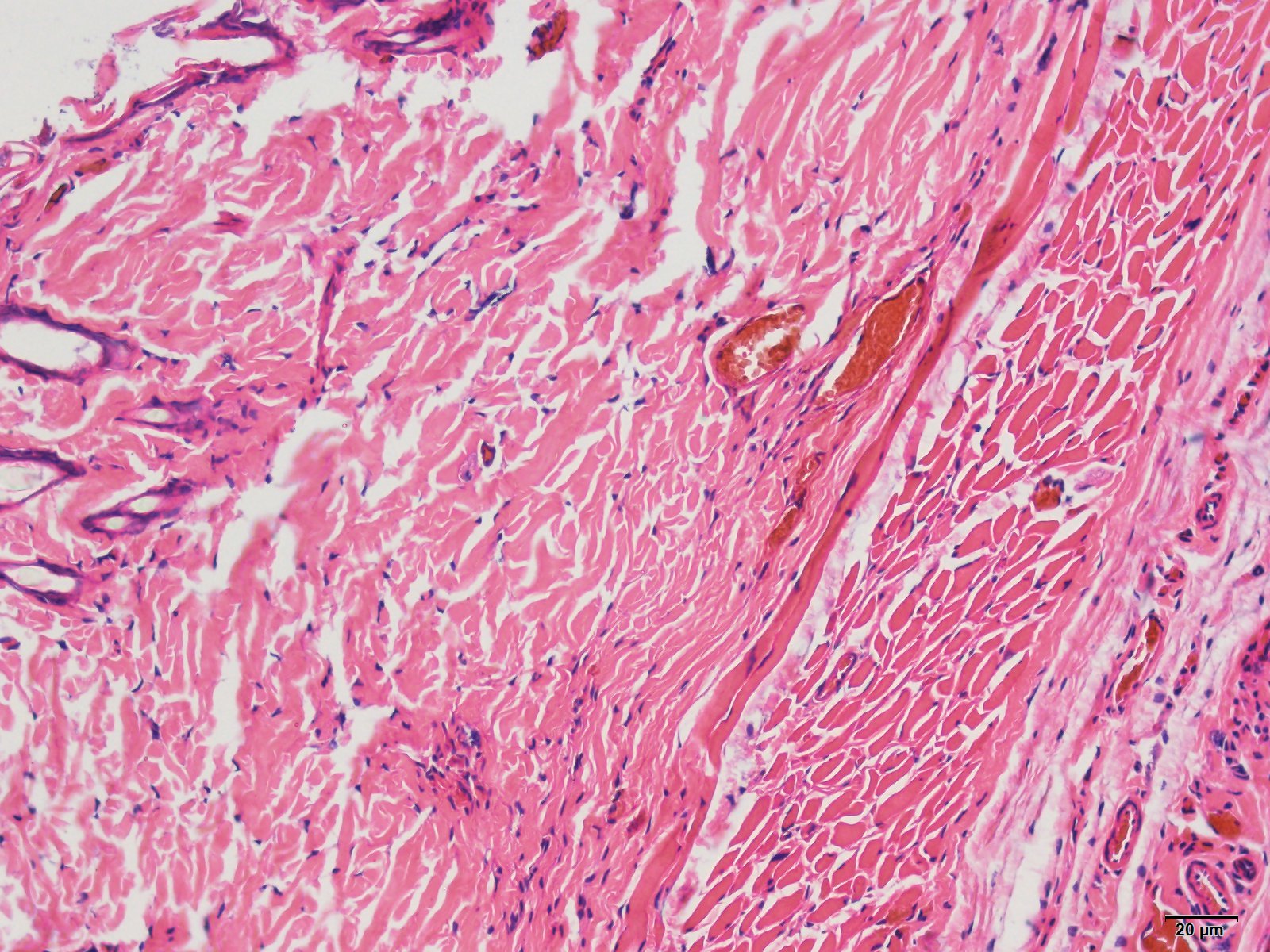

Supplement: S4 File — (ZIP) [file pone.0330078.s004.zip › HE staining/28d HAMCC 3.jpg]

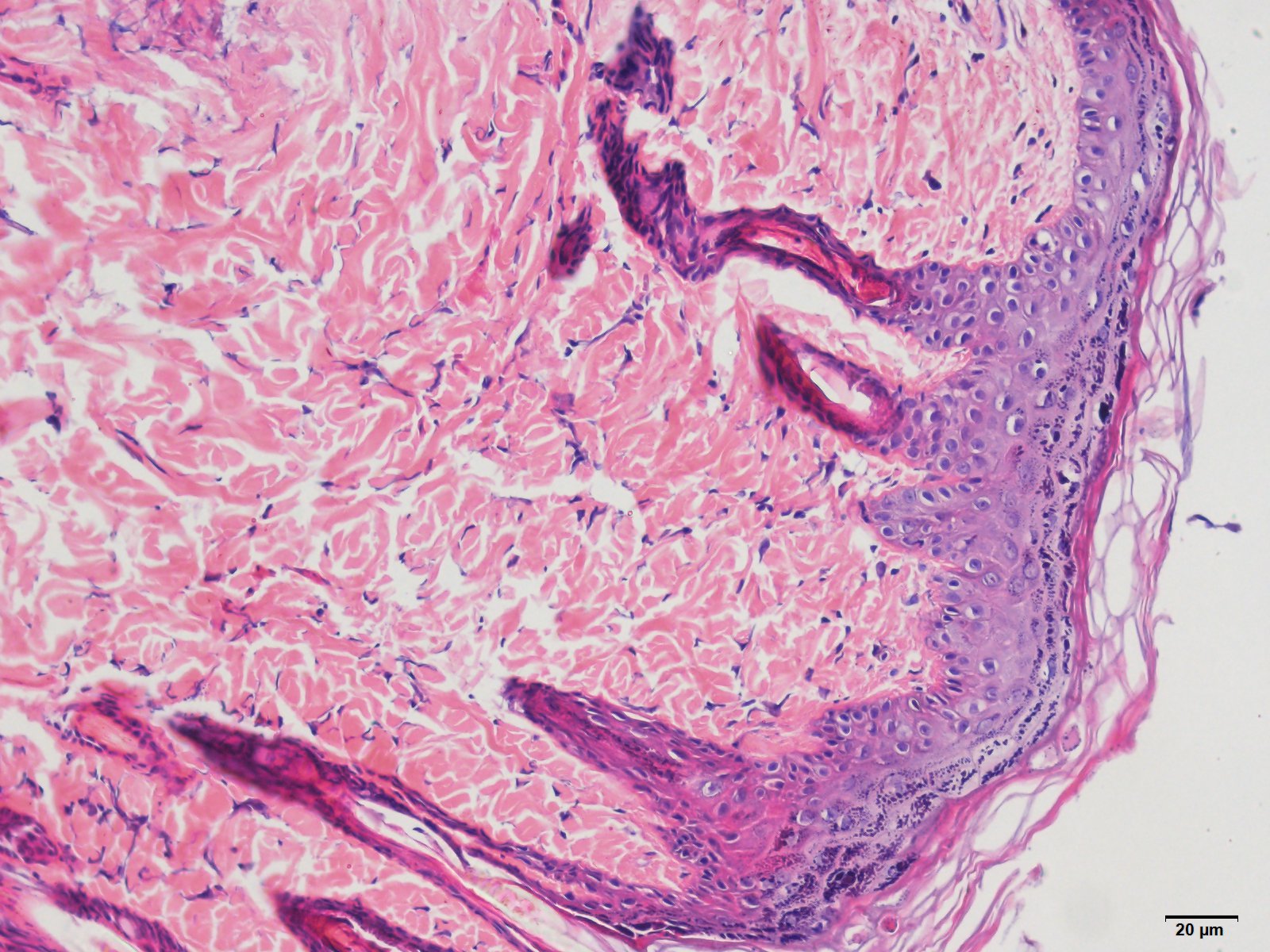

Supplement: S4 File — (ZIP) [file pone.0330078.s004.zip › HE staining/7d CGF 1.jpg]

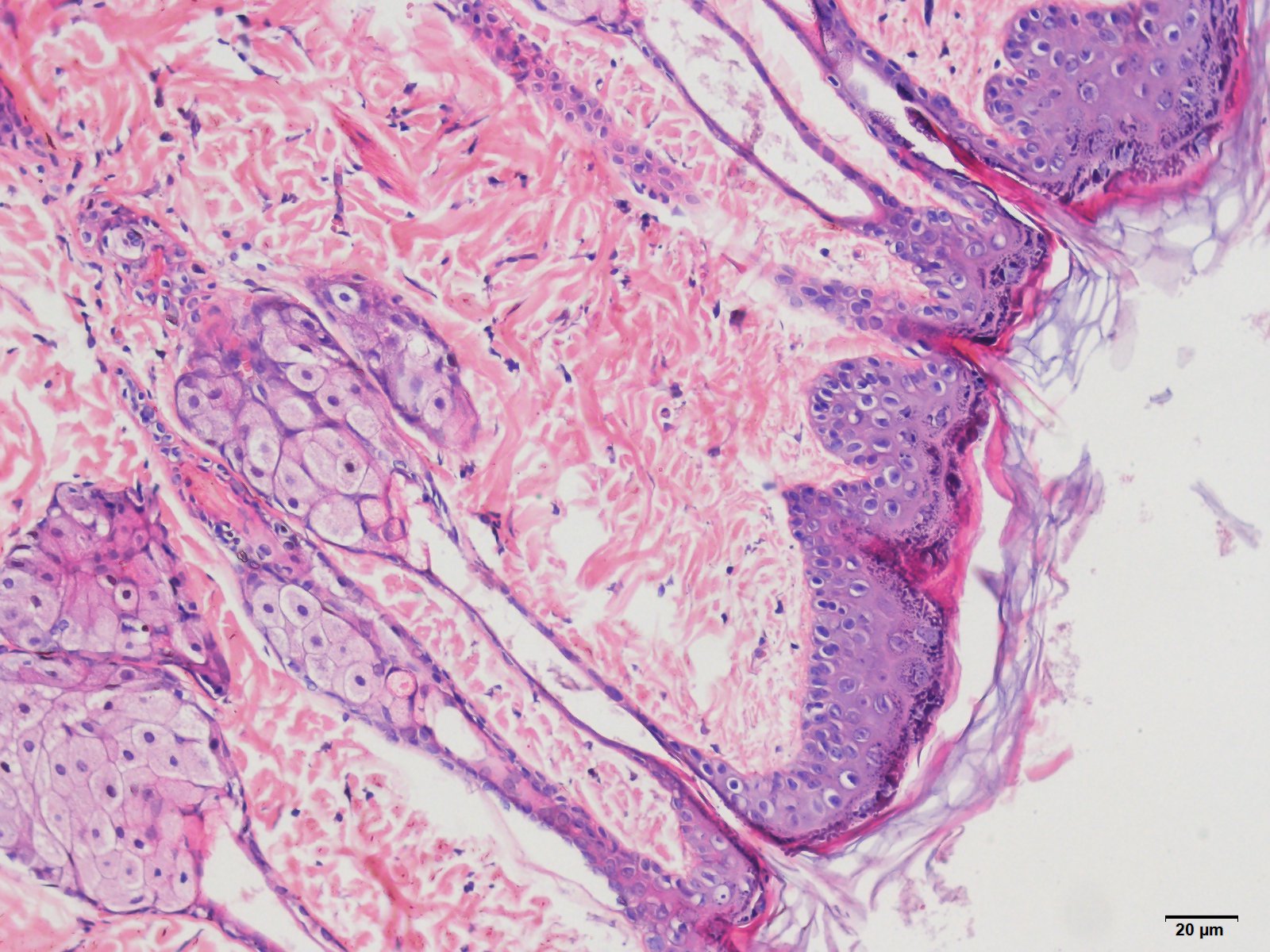

Supplement: S4 File — (ZIP) [file pone.0330078.s004.zip › HE staining/7d CGF 2.jpg]

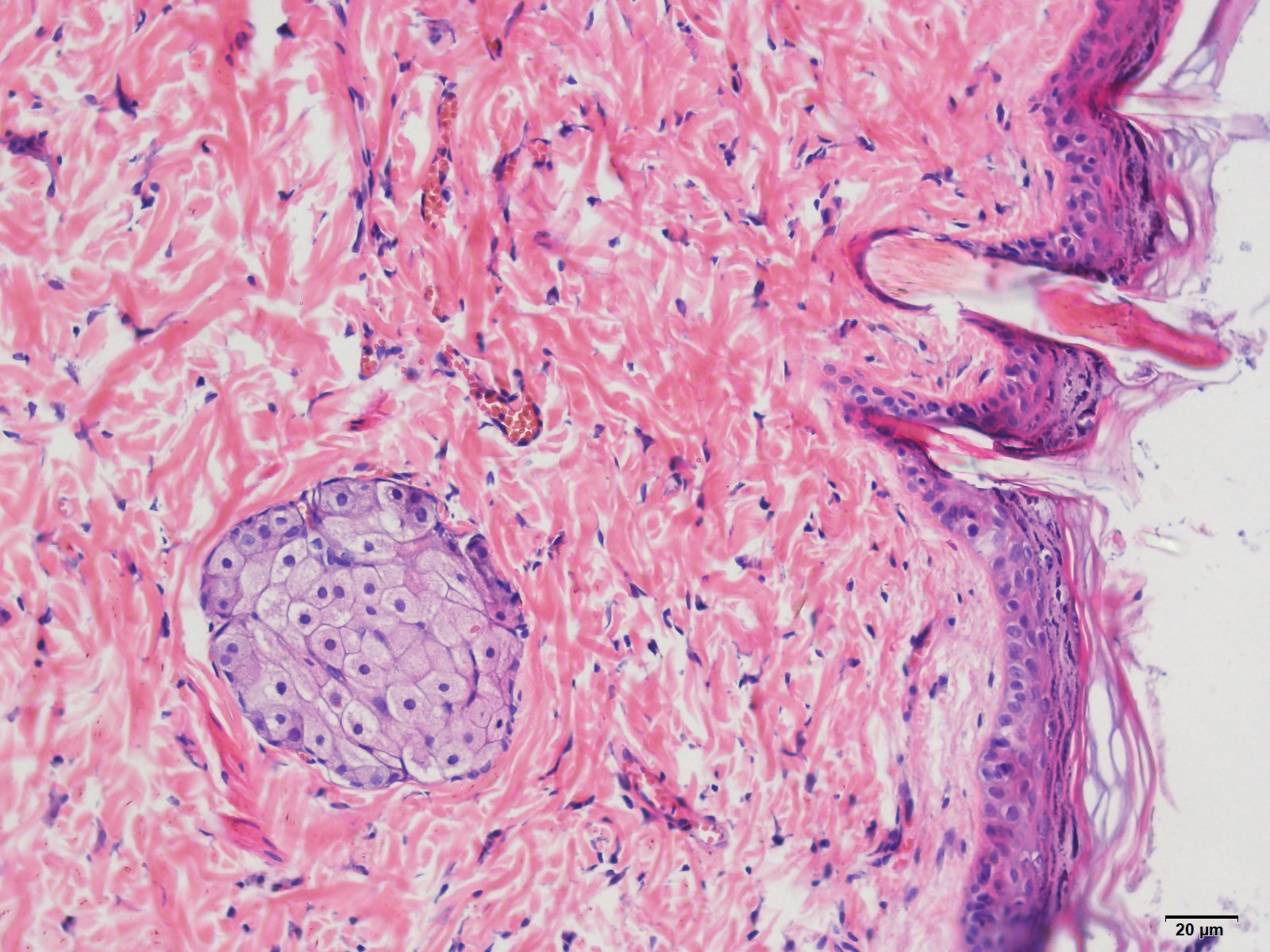

Supplement: S4 File — (ZIP) [file pone.0330078.s004.zip › HE staining/7d CGF 3.jpg]

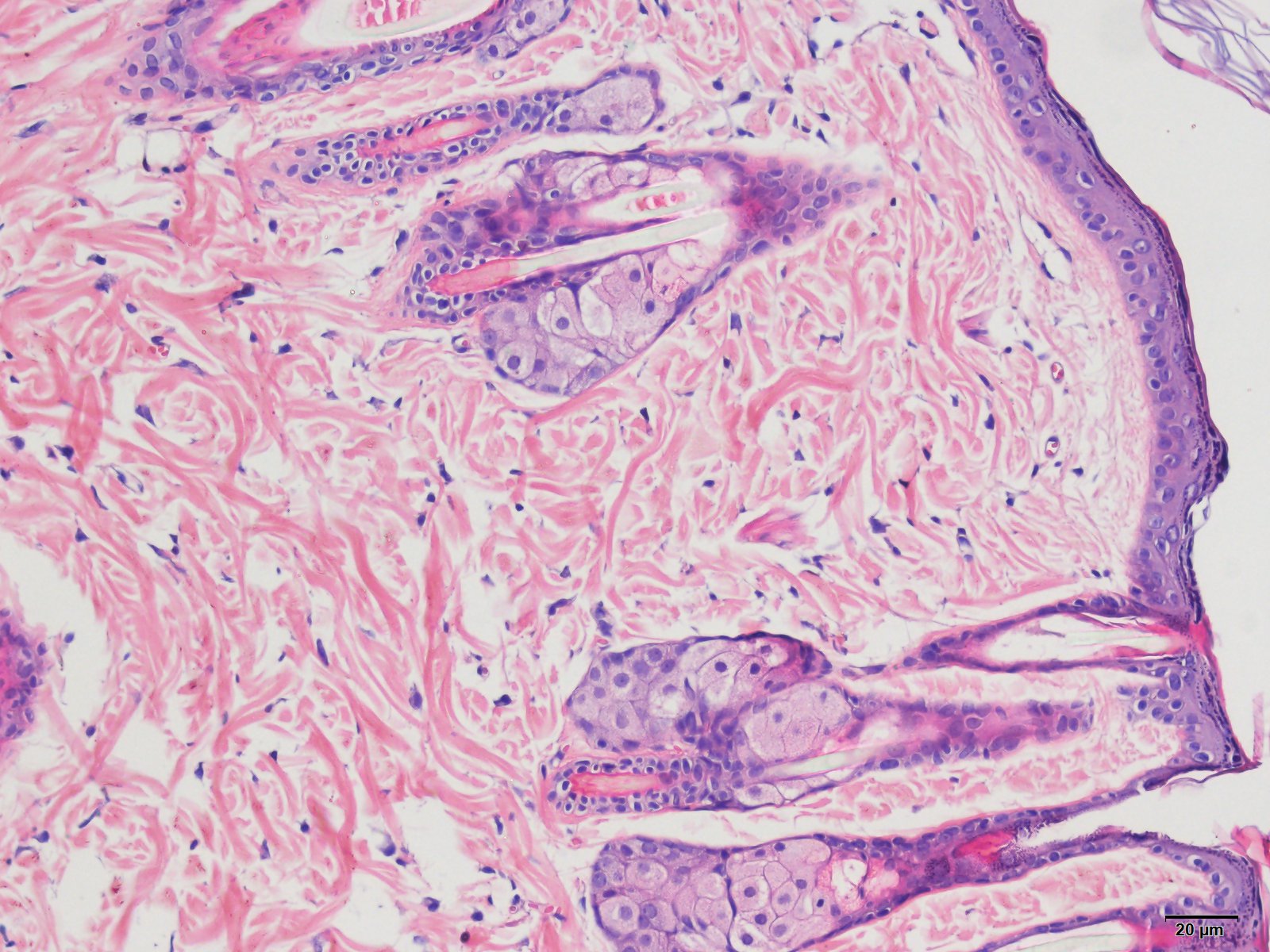

Supplement: S4 File — (ZIP) [file pone.0330078.s004.zip › HE staining/7d CGF+HAMCC 1.jpg]

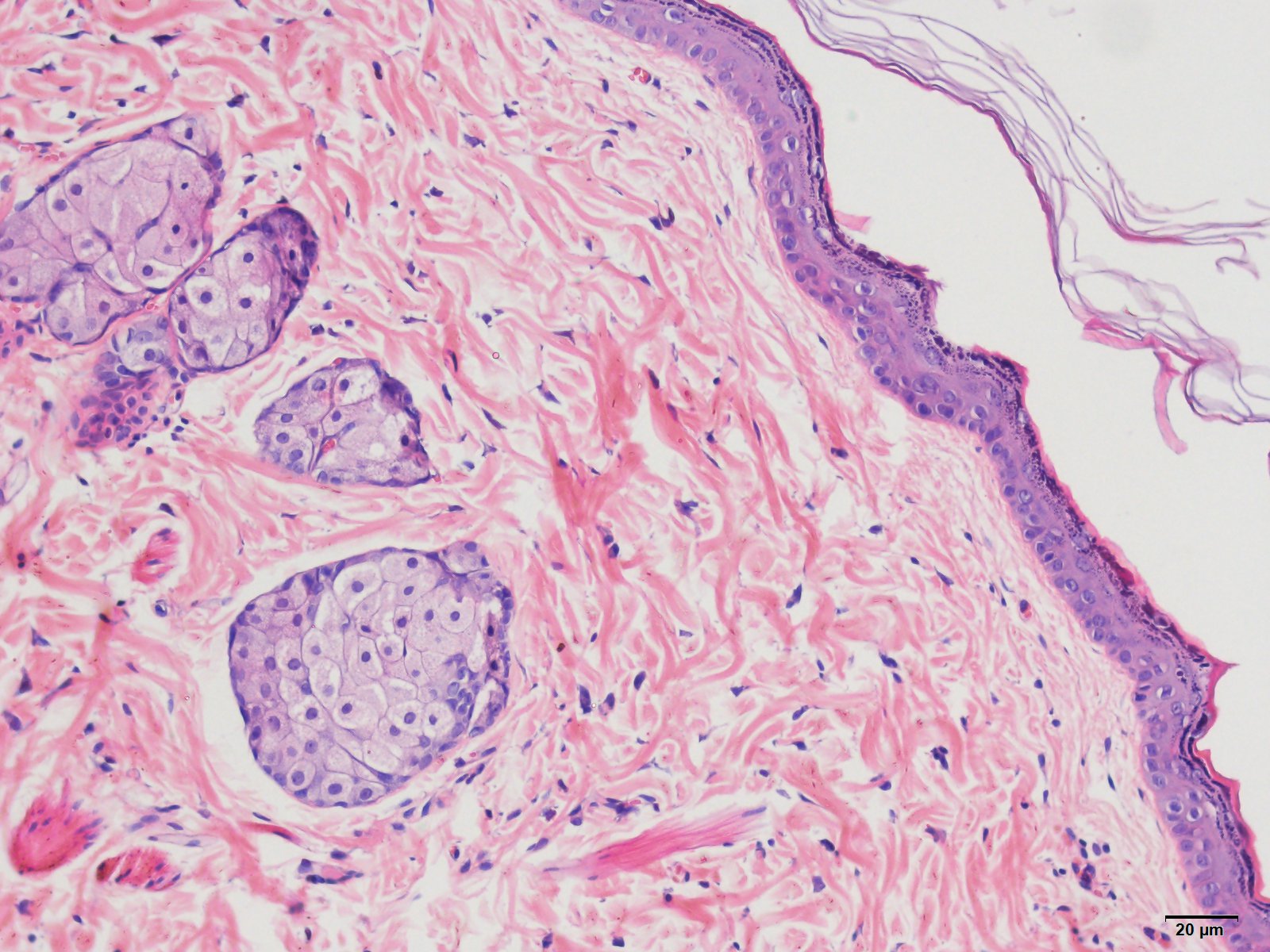

Supplement: S4 File — (ZIP) [file pone.0330078.s004.zip › HE staining/7d CGF+HAMCC 2.jpg]

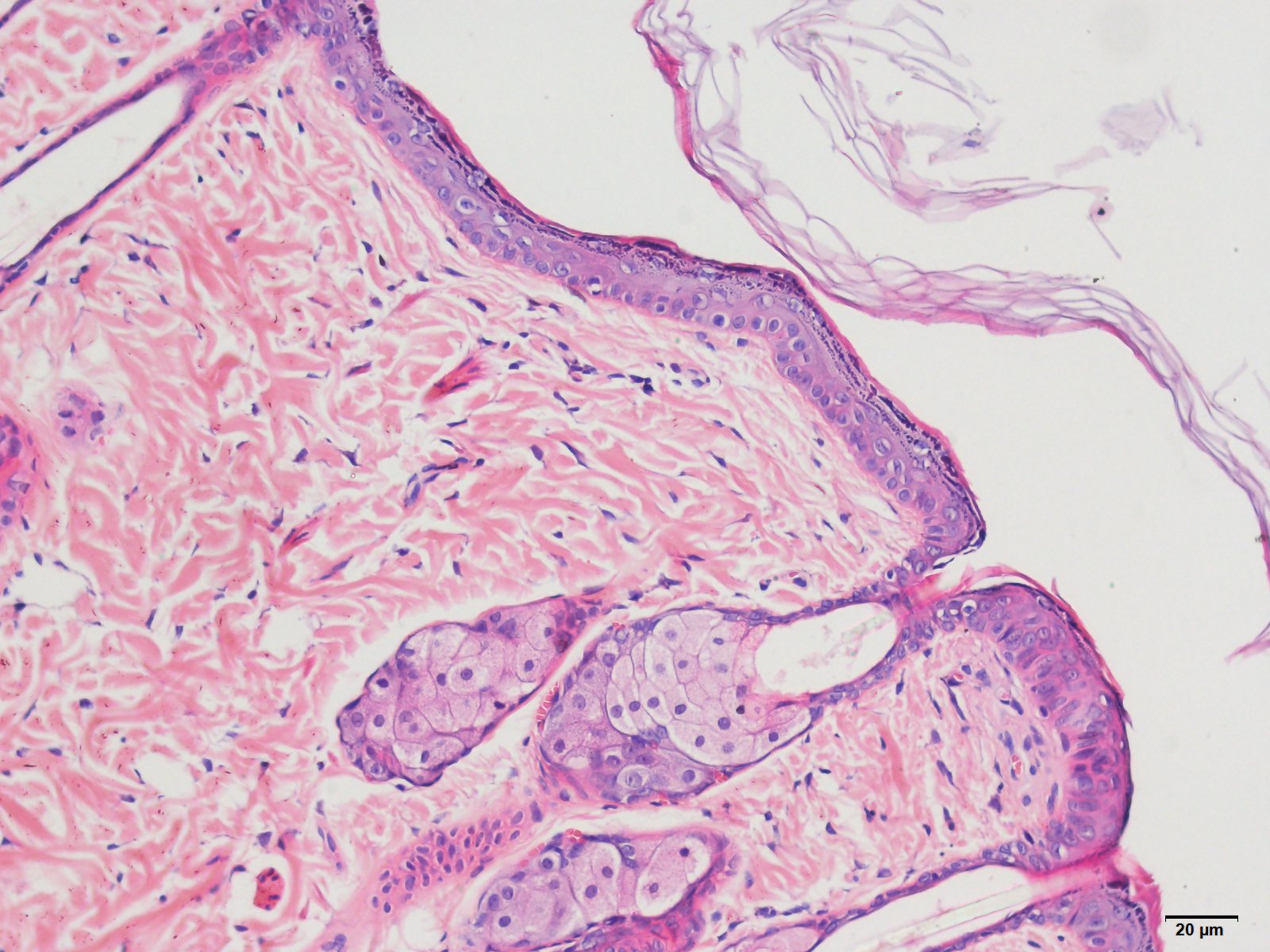

Supplement: S4 File — (ZIP) [file pone.0330078.s004.zip › HE staining/7d CGF+HAMCC 3.jpg]

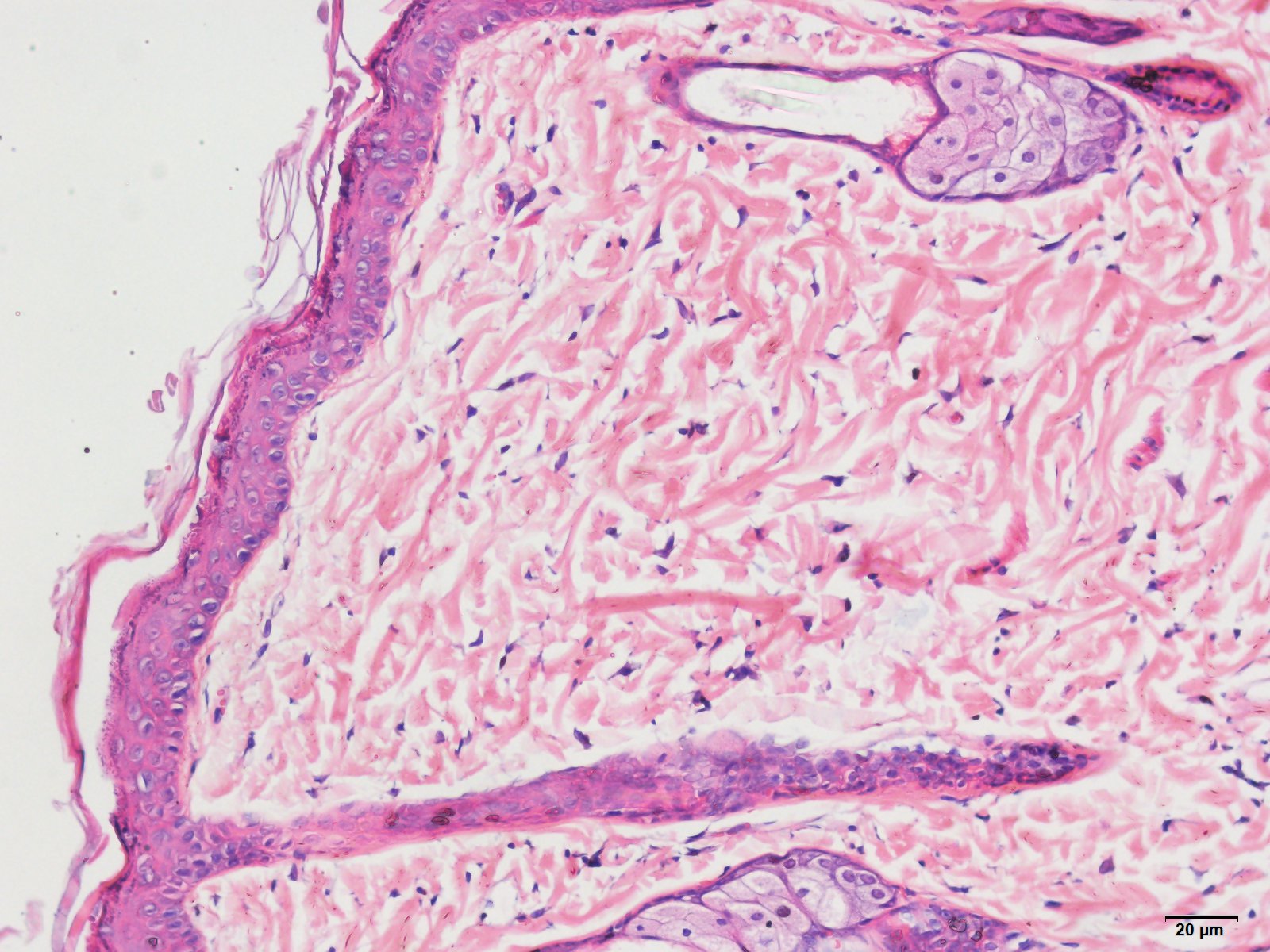

Supplement: S4 File — (ZIP) [file pone.0330078.s004.zip › HE staining/7d Control 1.jpg]

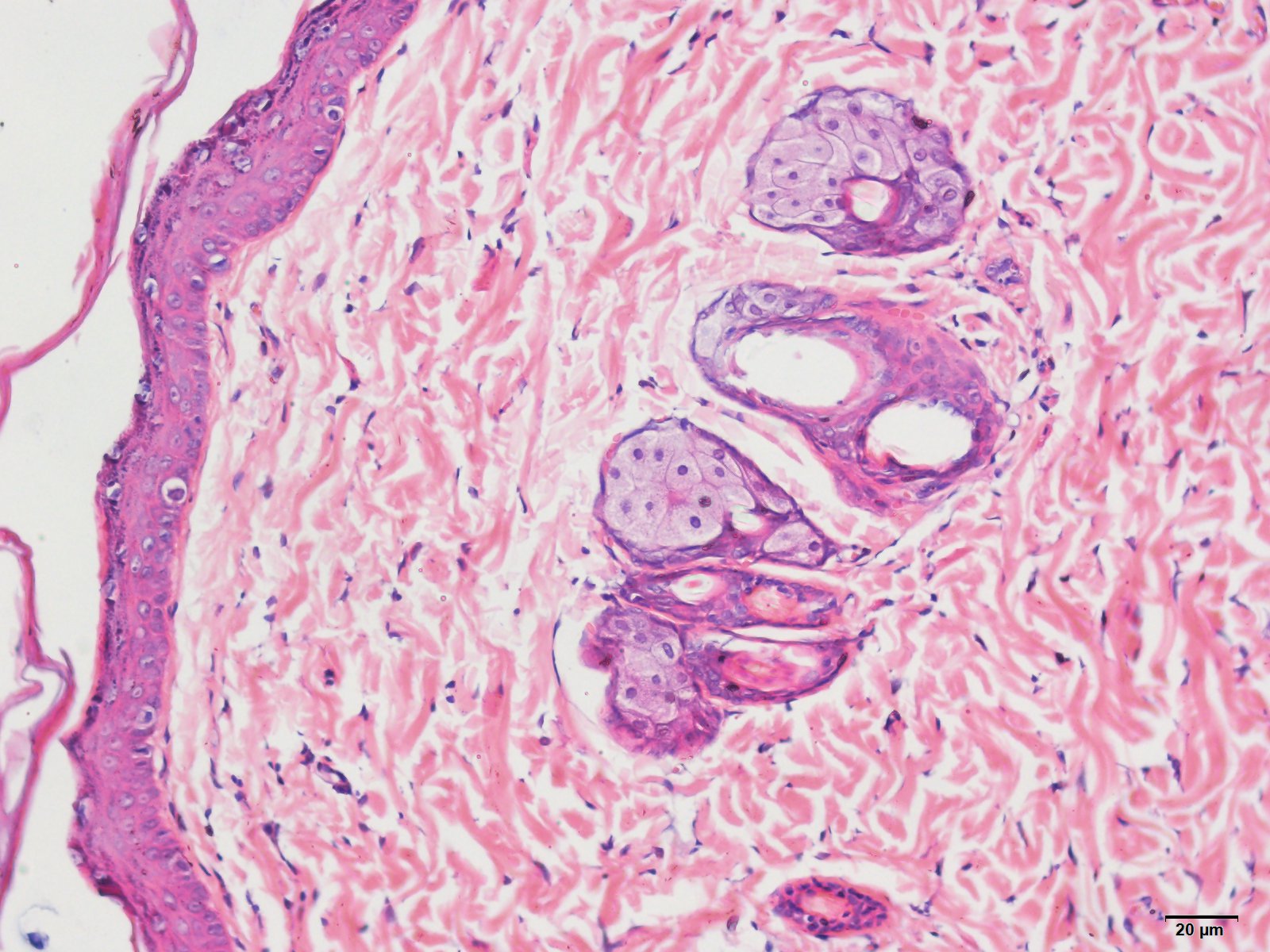

Supplement: S4 File — (ZIP) [file pone.0330078.s004.zip › HE staining/7d Control 2.jpg]

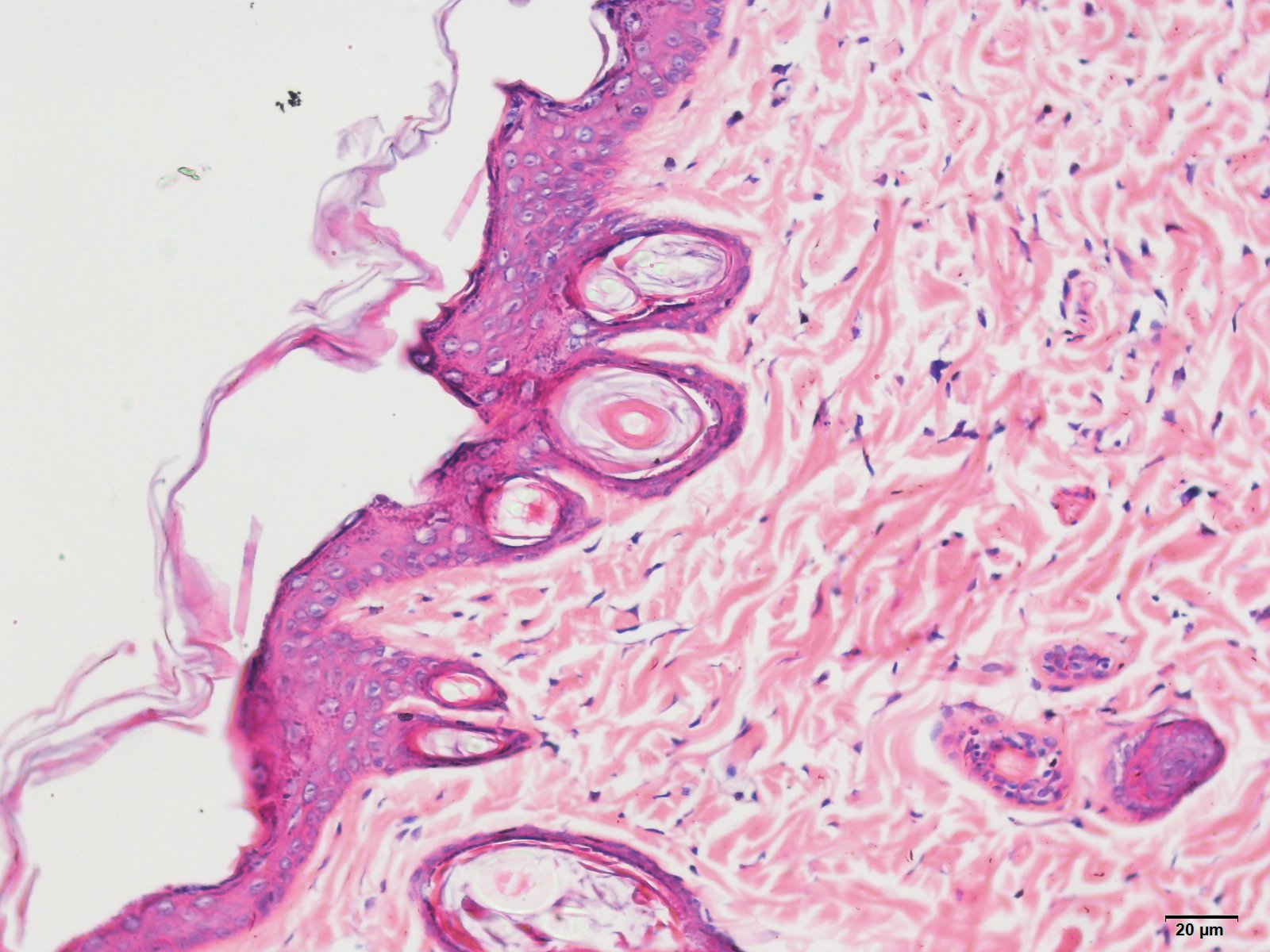

Supplement: S4 File — (ZIP) [file pone.0330078.s004.zip › HE staining/7d Control 3.jpg]

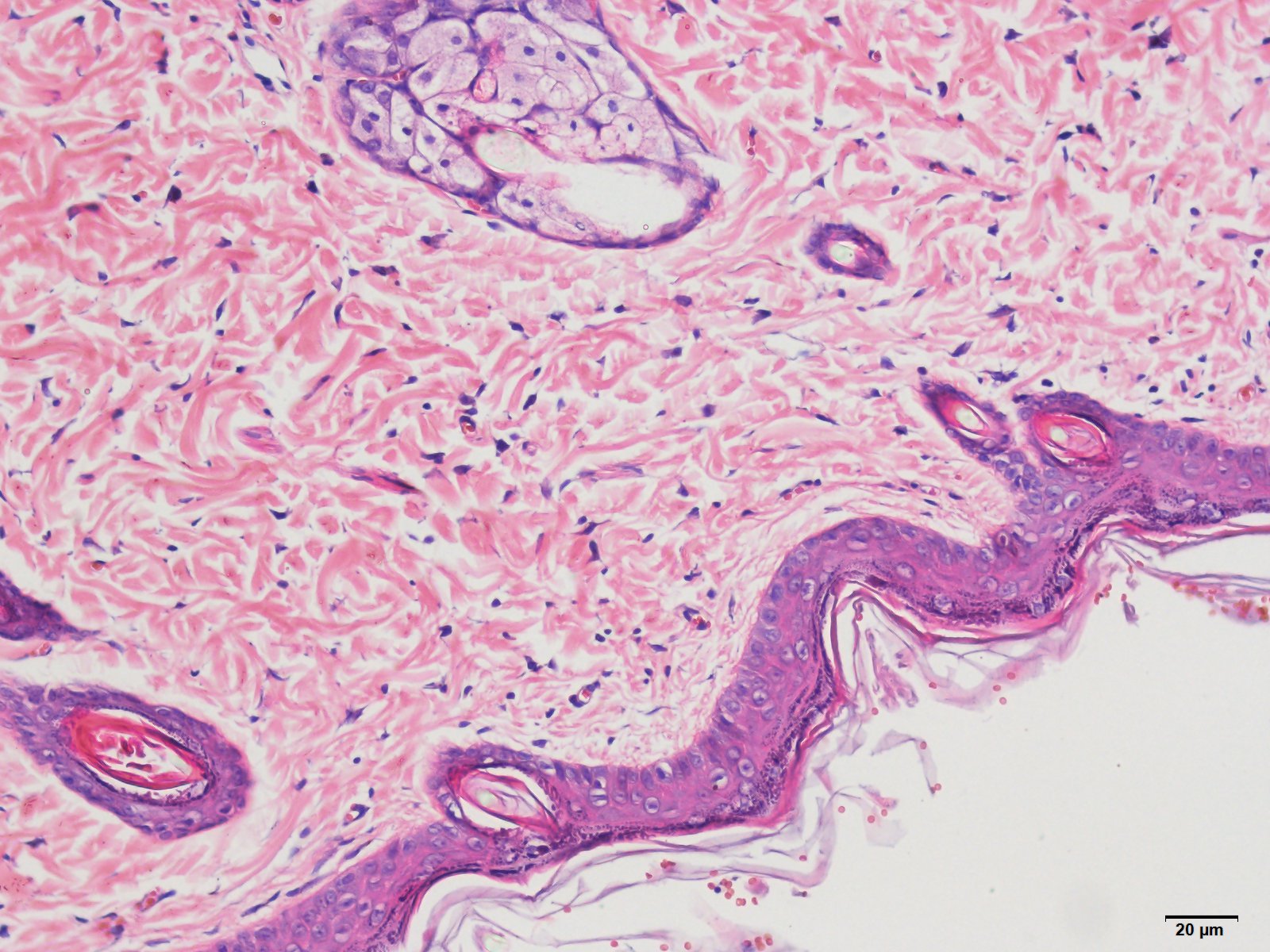

Supplement: S4 File — (ZIP) [file pone.0330078.s004.zip › HE staining/7d HAMCC 1.jpg]

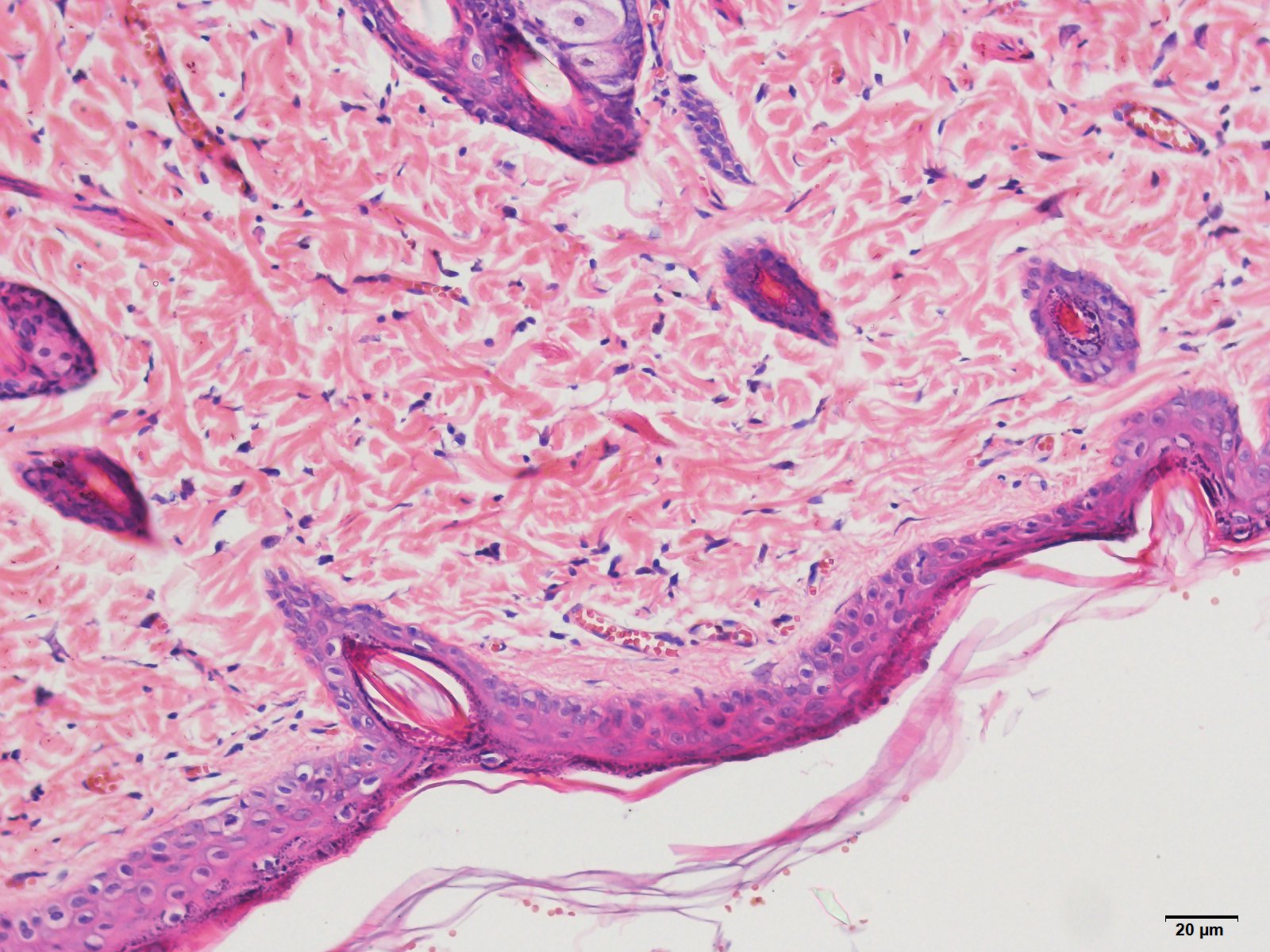

Supplement: S4 File — (ZIP) [file pone.0330078.s004.zip › HE staining/7d HAMCC 2.jpg]

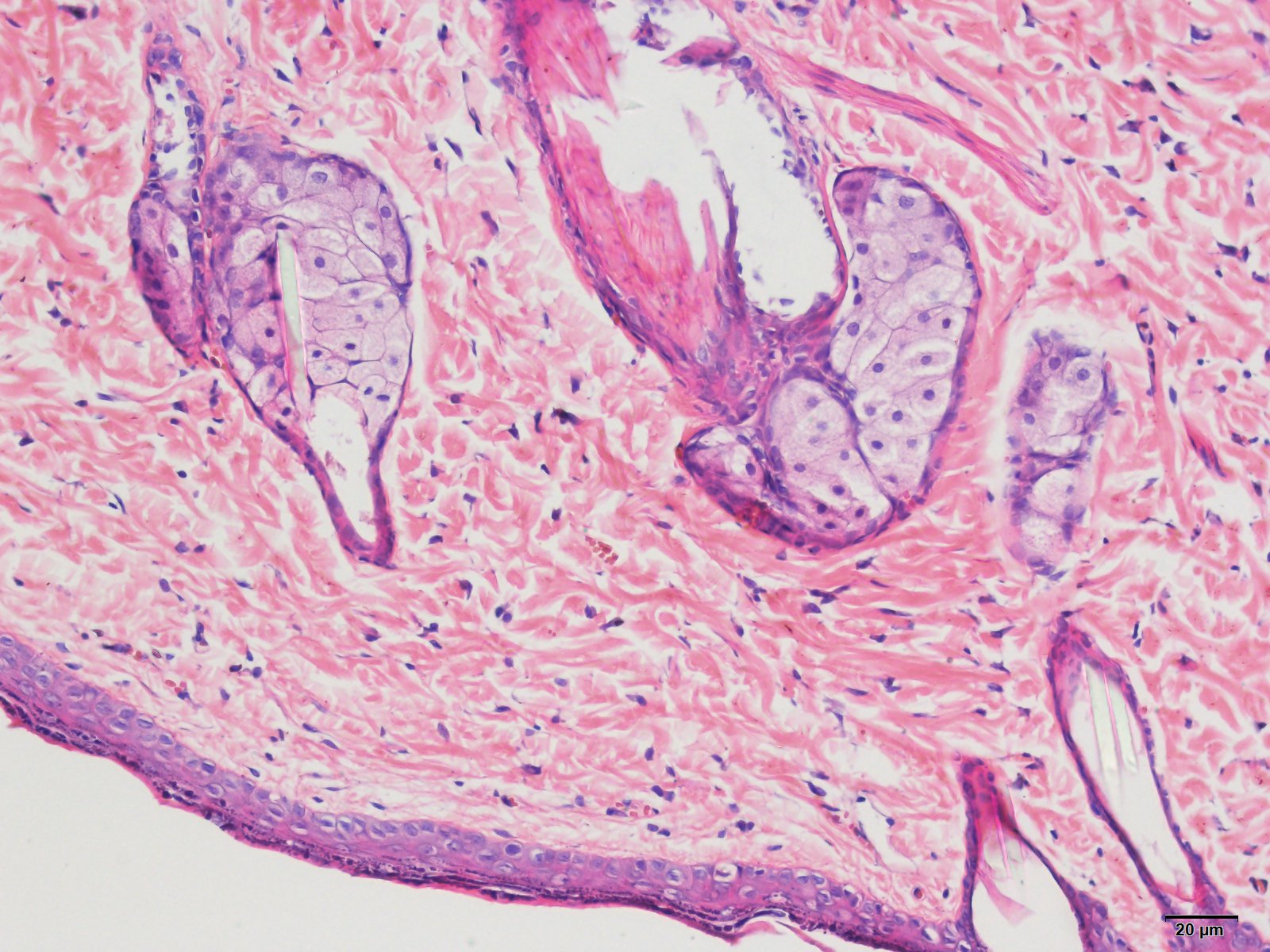

Supplement: S4 File — (ZIP) [file pone.0330078.s004.zip › HE staining/7d HAMCC 3.jpg]

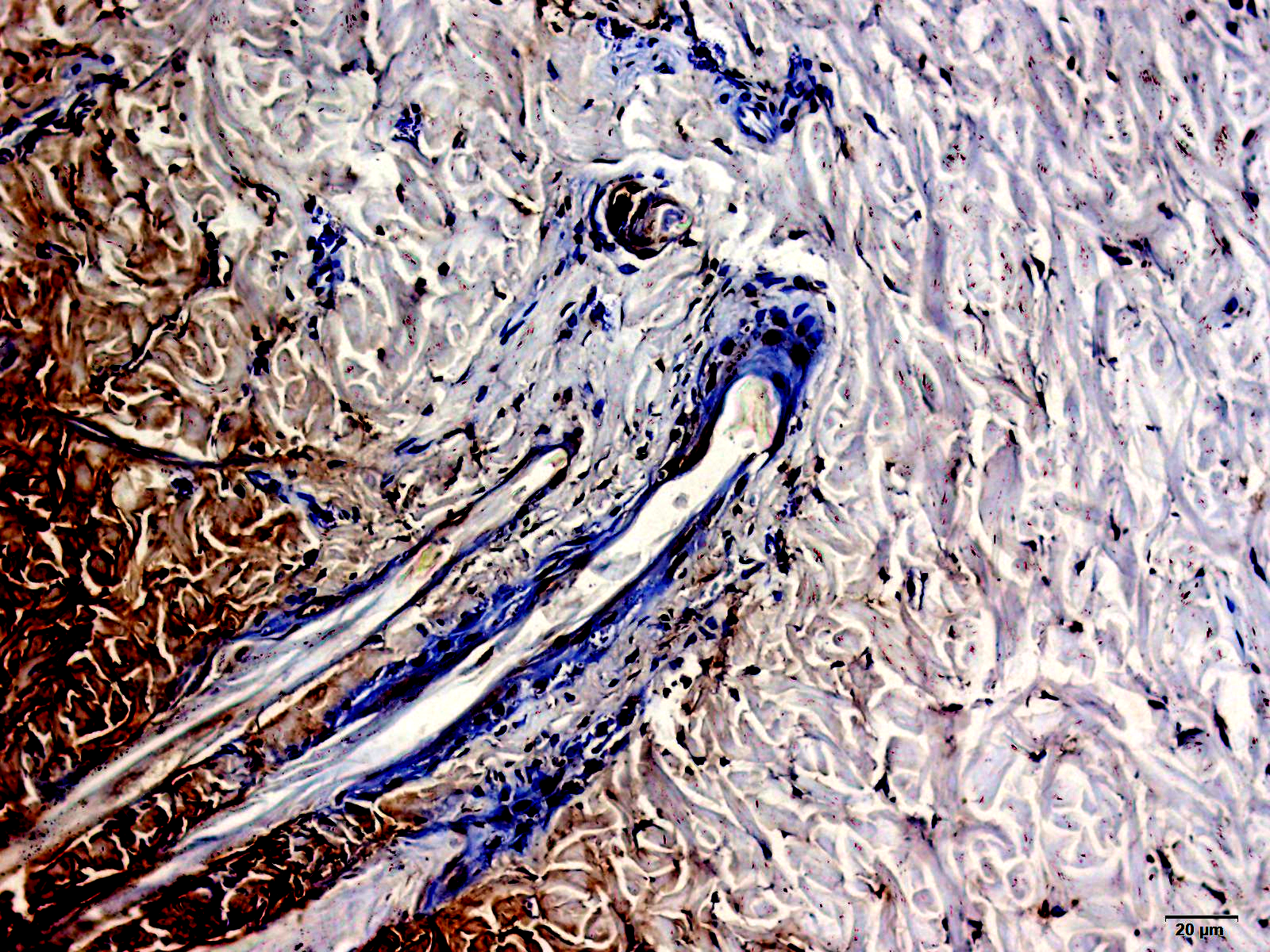

Supplement: S5 File — (ZIP) [file pone.0330078.s005.zip › Tunel/14D/CGF/CGF 1.tif]

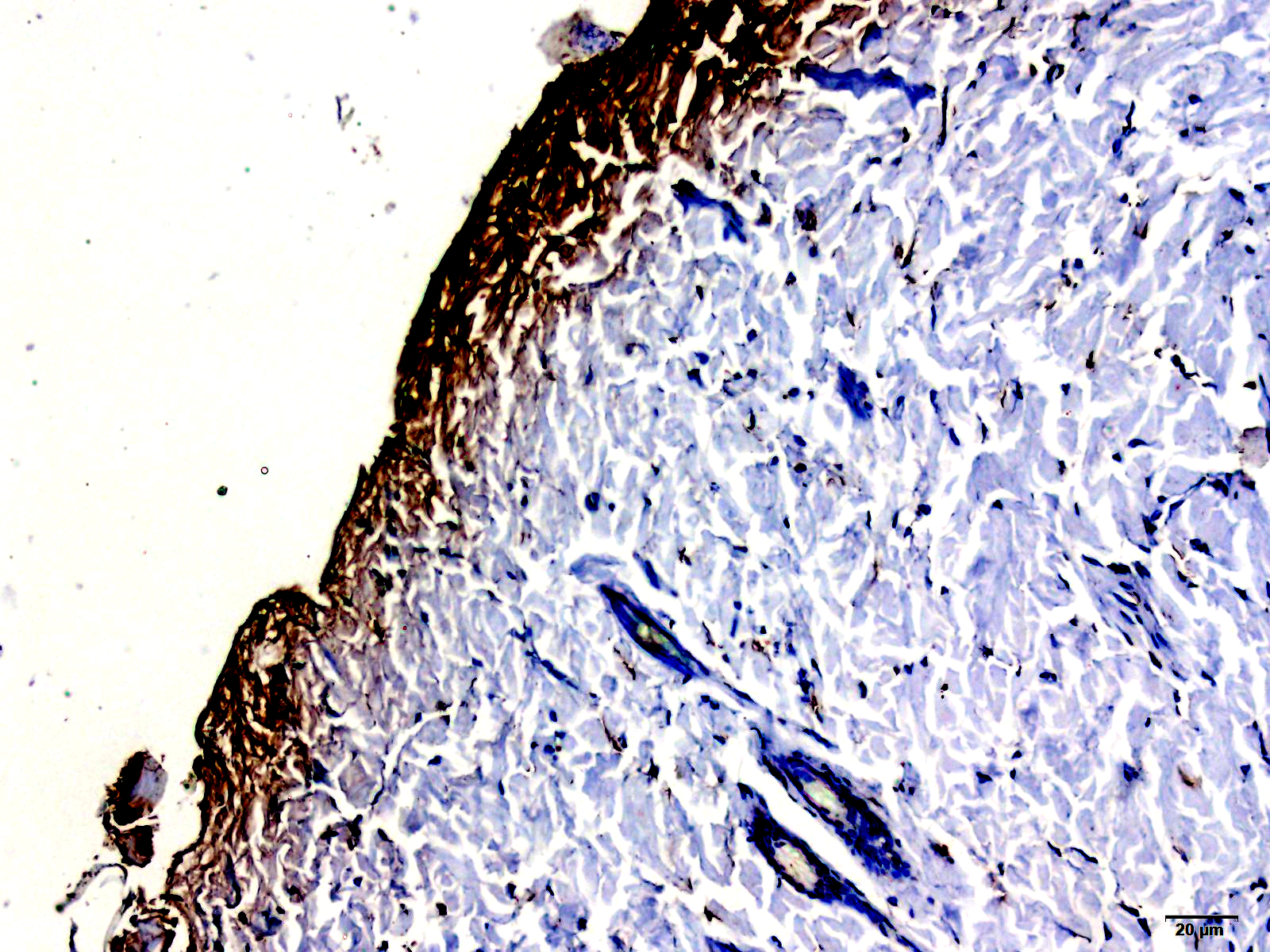

Supplement: S5 File — (ZIP) [file pone.0330078.s005.zip › Tunel/14D/CGF/CGF 2.tif]

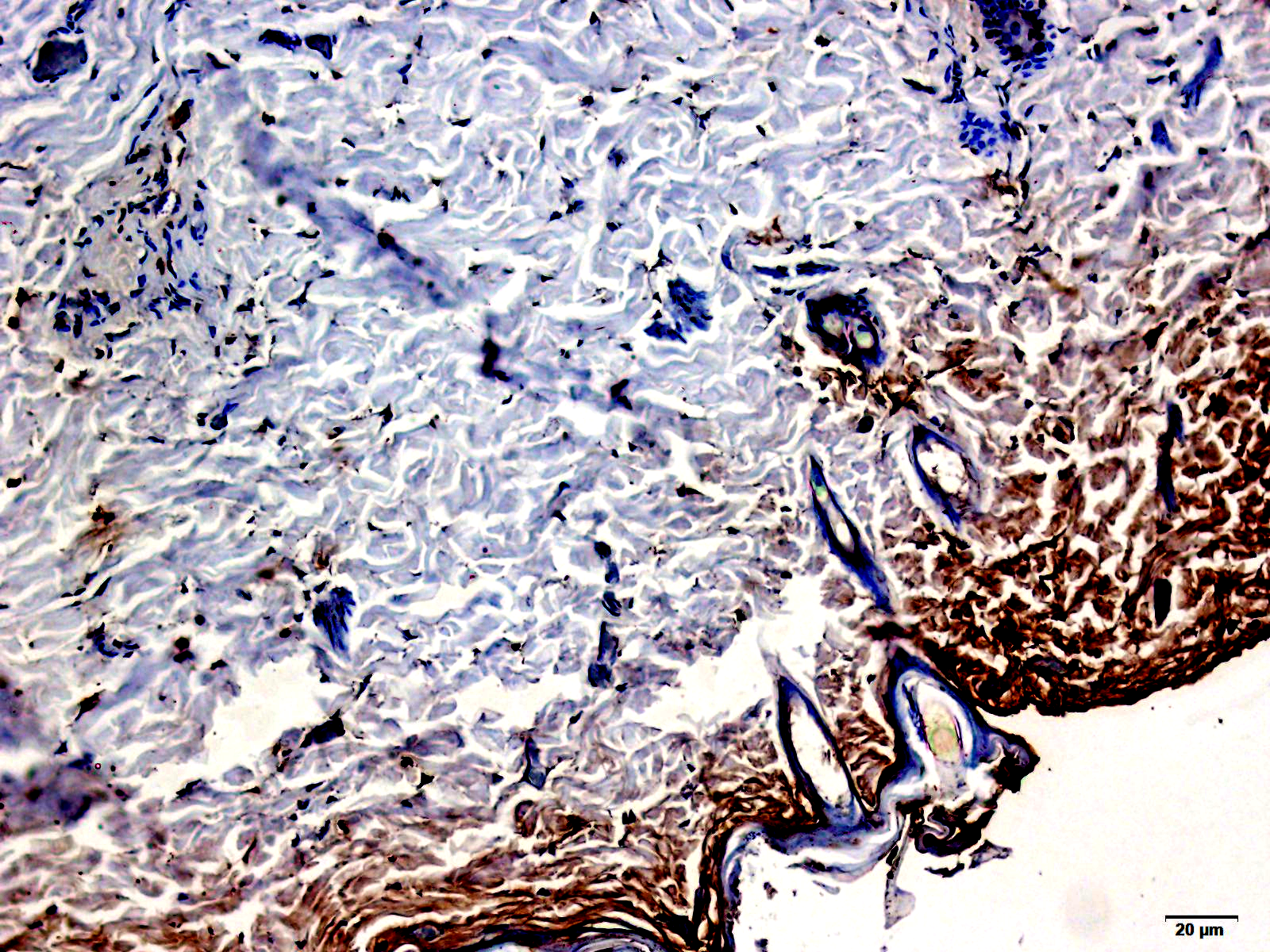

Supplement: S5 File — (ZIP) [file pone.0330078.s005.zip › Tunel/14D/CGF/CGF 3.tif]

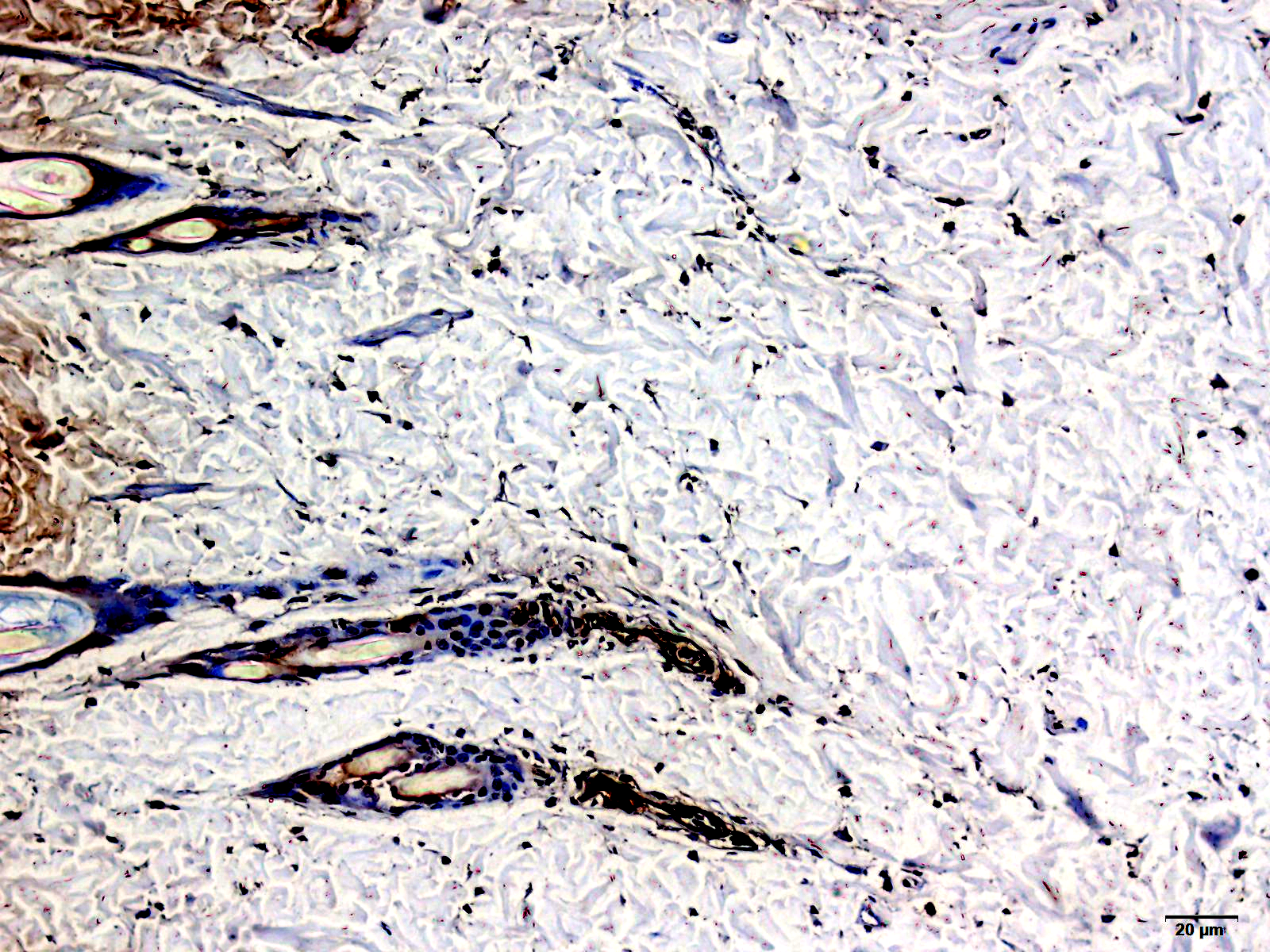

Supplement: S5 File — (ZIP) [file pone.0330078.s005.zip › Tunel/14D/CGF+HAMCC/CGF+HAMCC 1.tif]

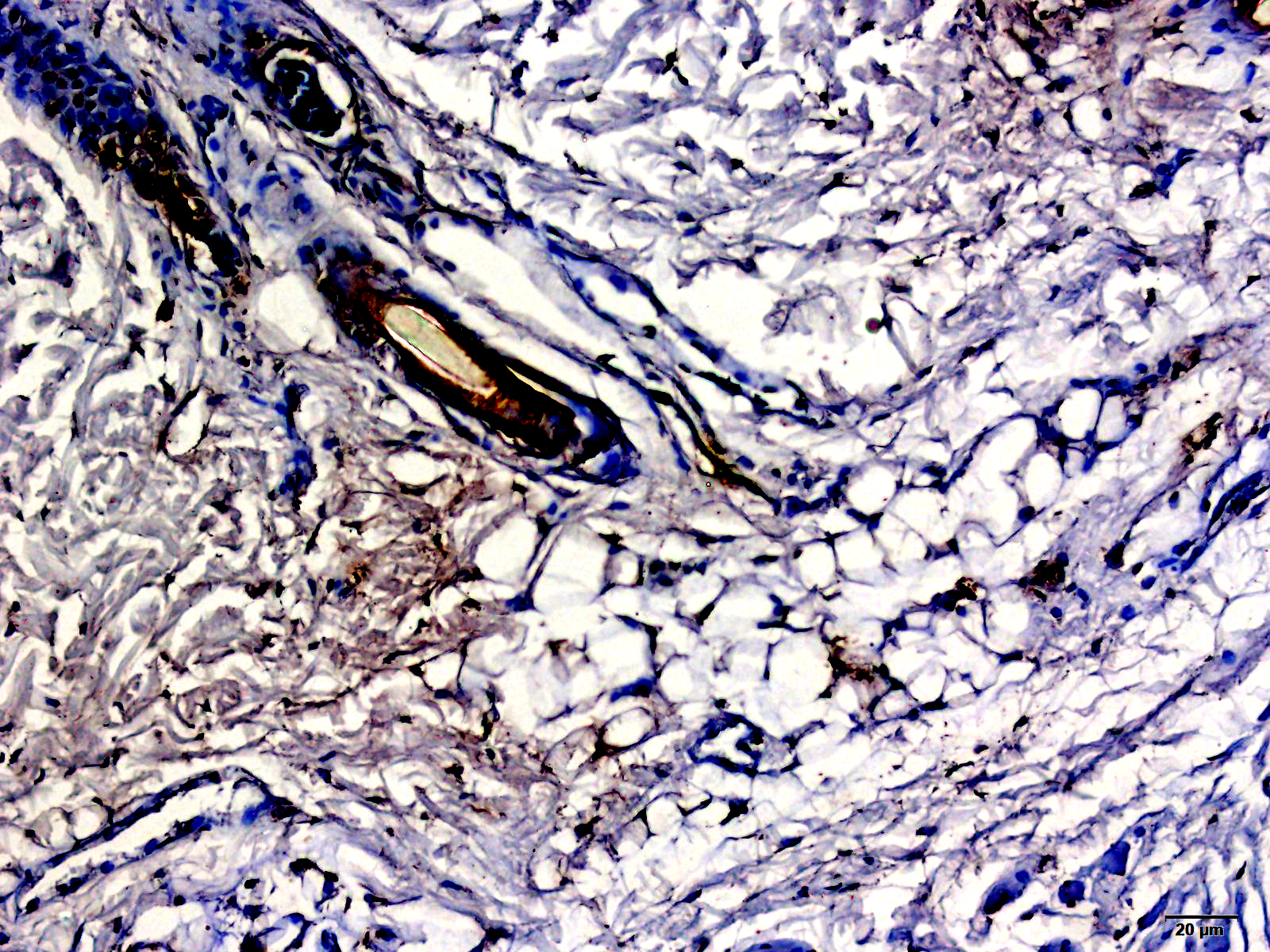

Supplement: S5 File — (ZIP) [file pone.0330078.s005.zip › Tunel/14D/CGF+HAMCC/CGF+HAMCC 2.tif]

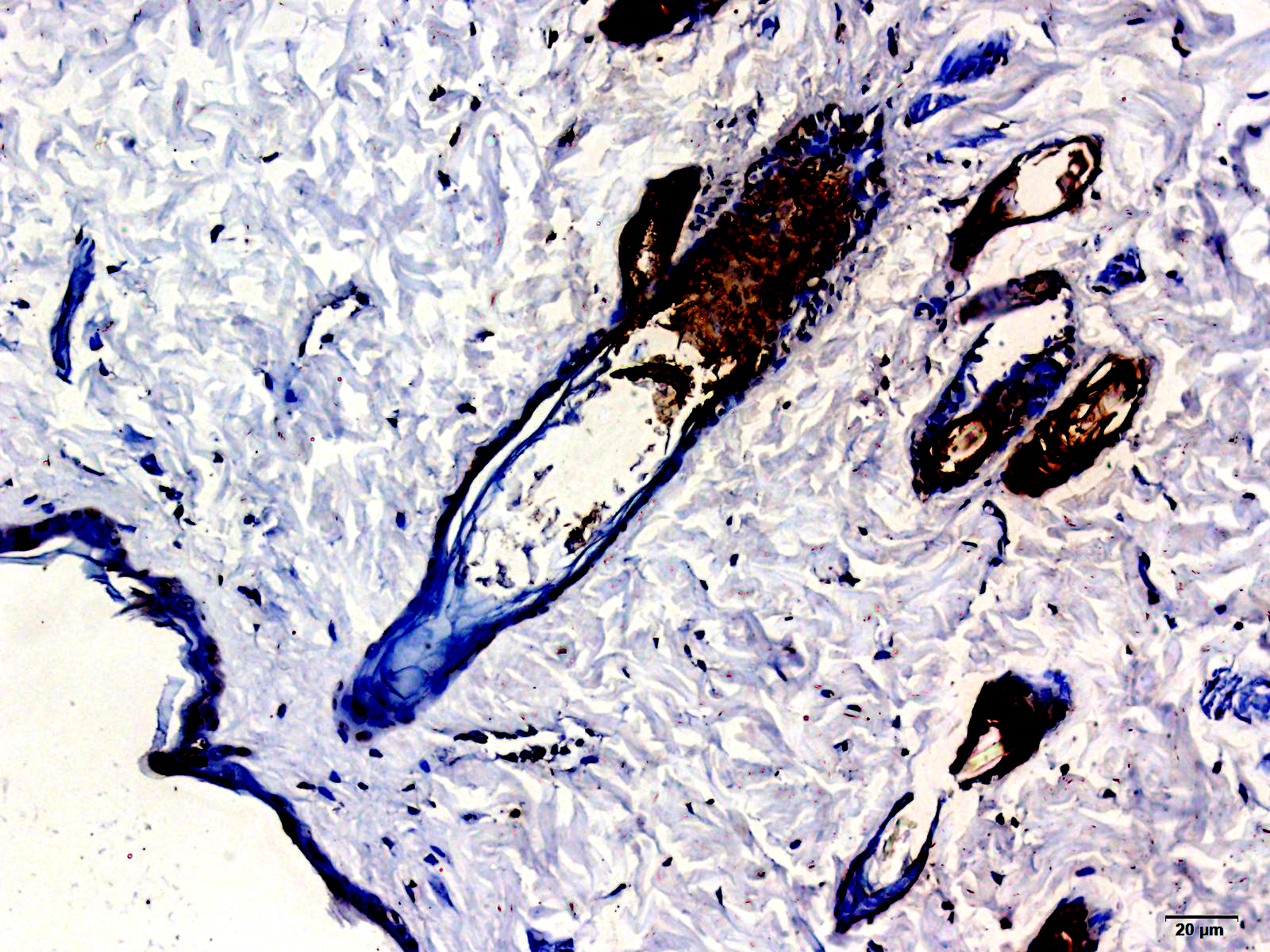

Supplement: S5 File — (ZIP) [file pone.0330078.s005.zip › Tunel/14D/CGF+HAMCC/CGF+HAMCC 3.tif]

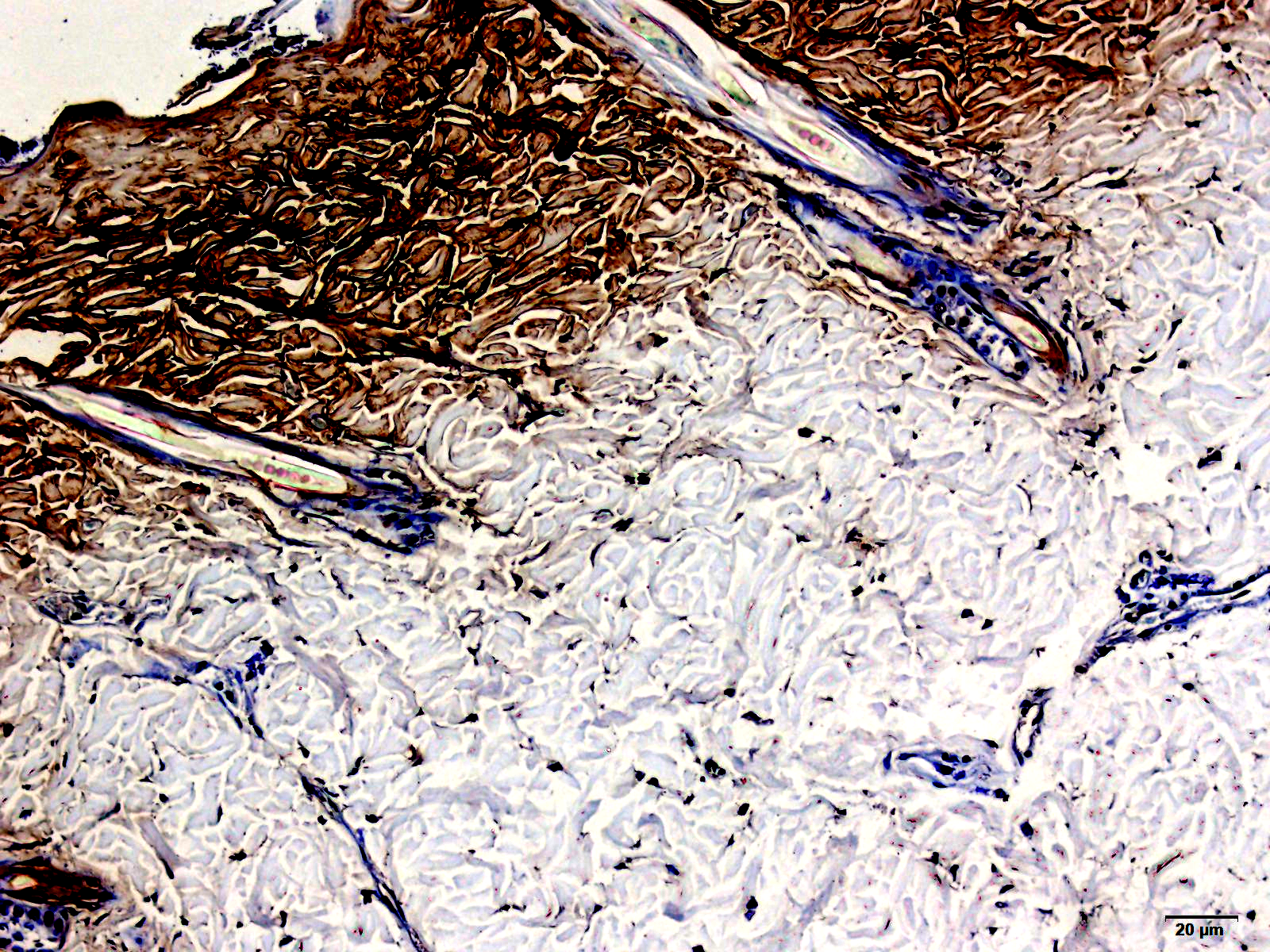

Supplement: S5 File — (ZIP) [file pone.0330078.s005.zip › Tunel/14D/Control/Control 1.tif]

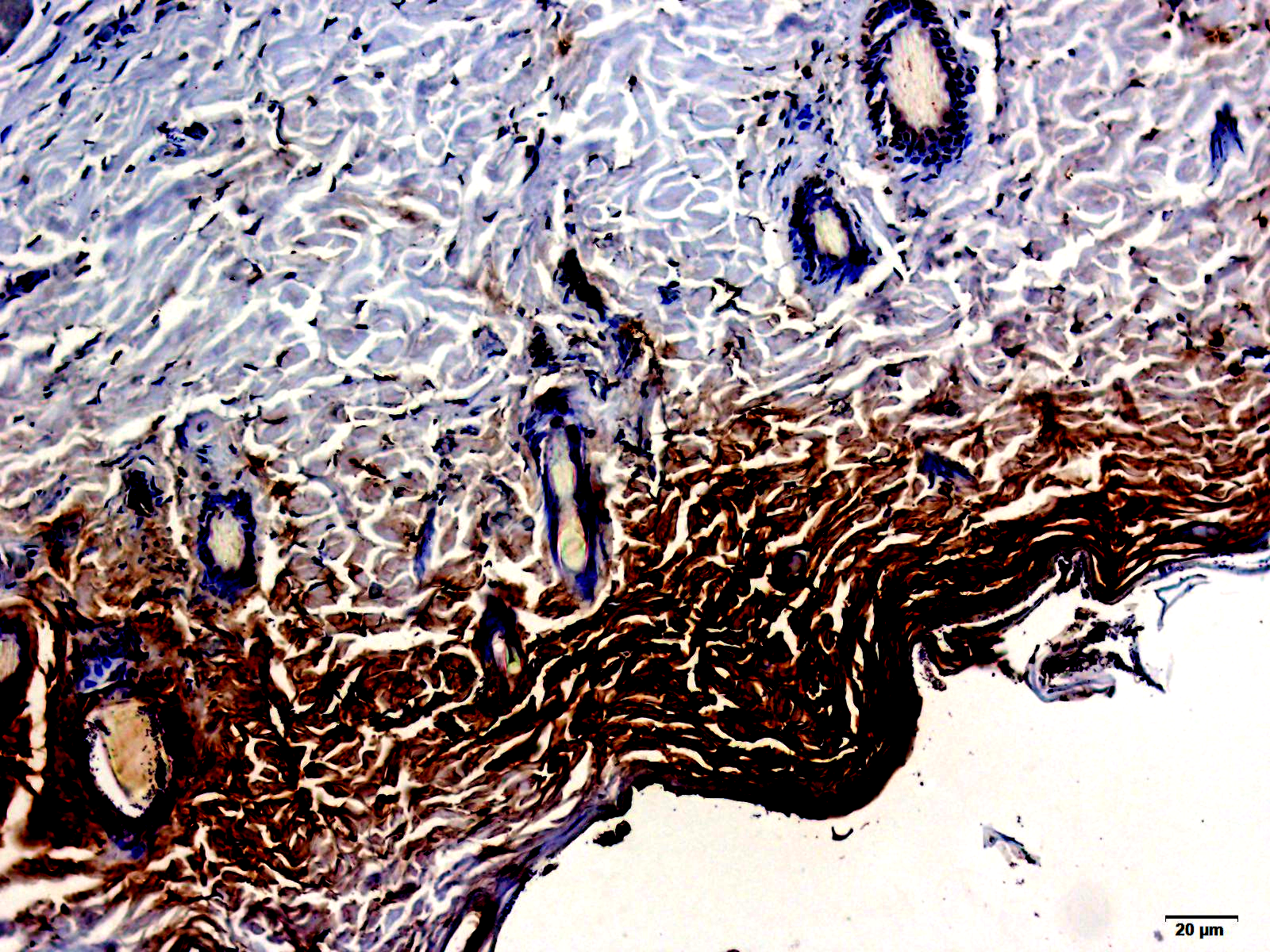

Supplement: S5 File — (ZIP) [file pone.0330078.s005.zip › Tunel/14D/Control/Control 2.tif]

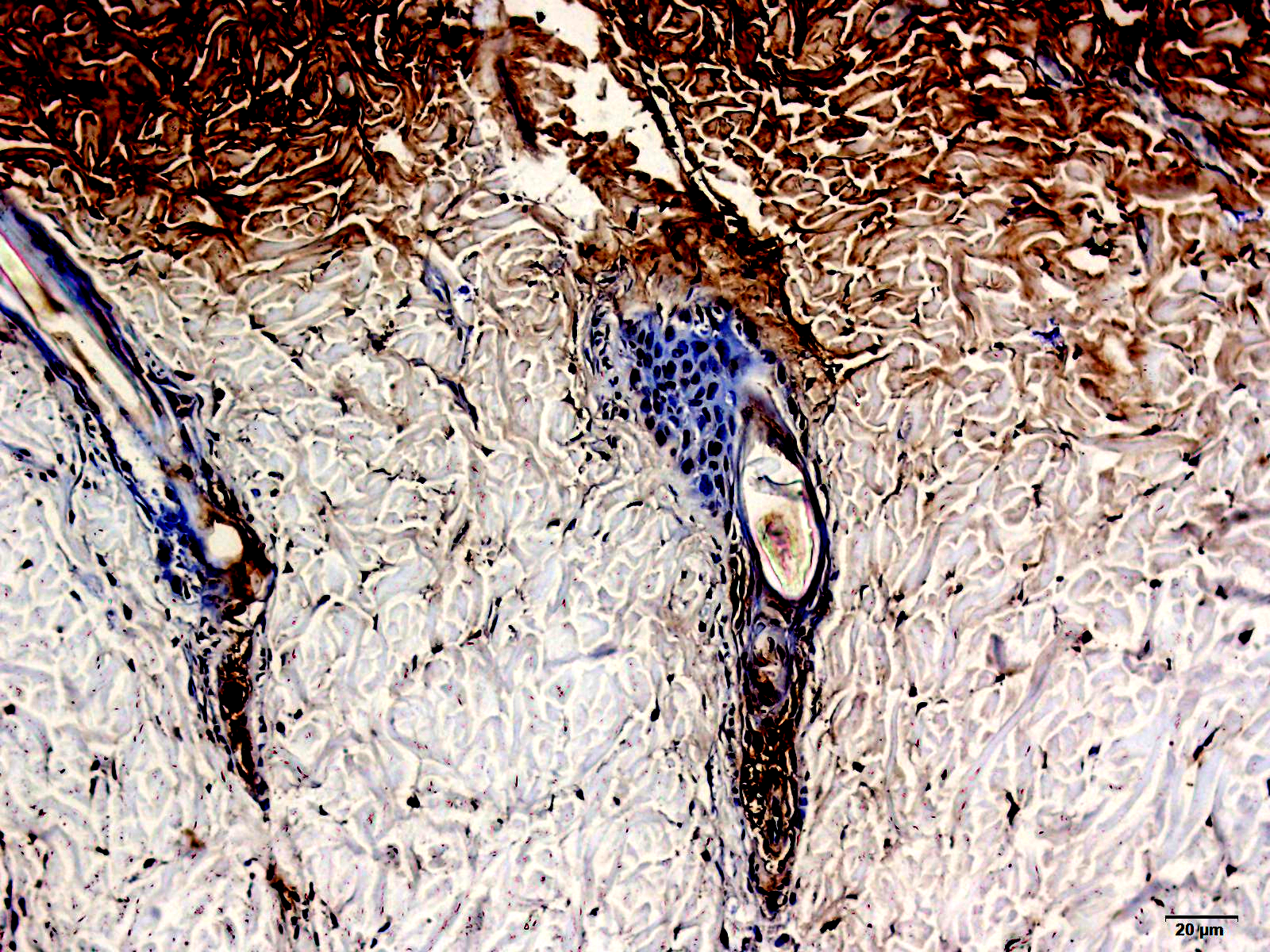

Supplement: S5 File — (ZIP) [file pone.0330078.s005.zip › Tunel/14D/Control/Control 3.tif]

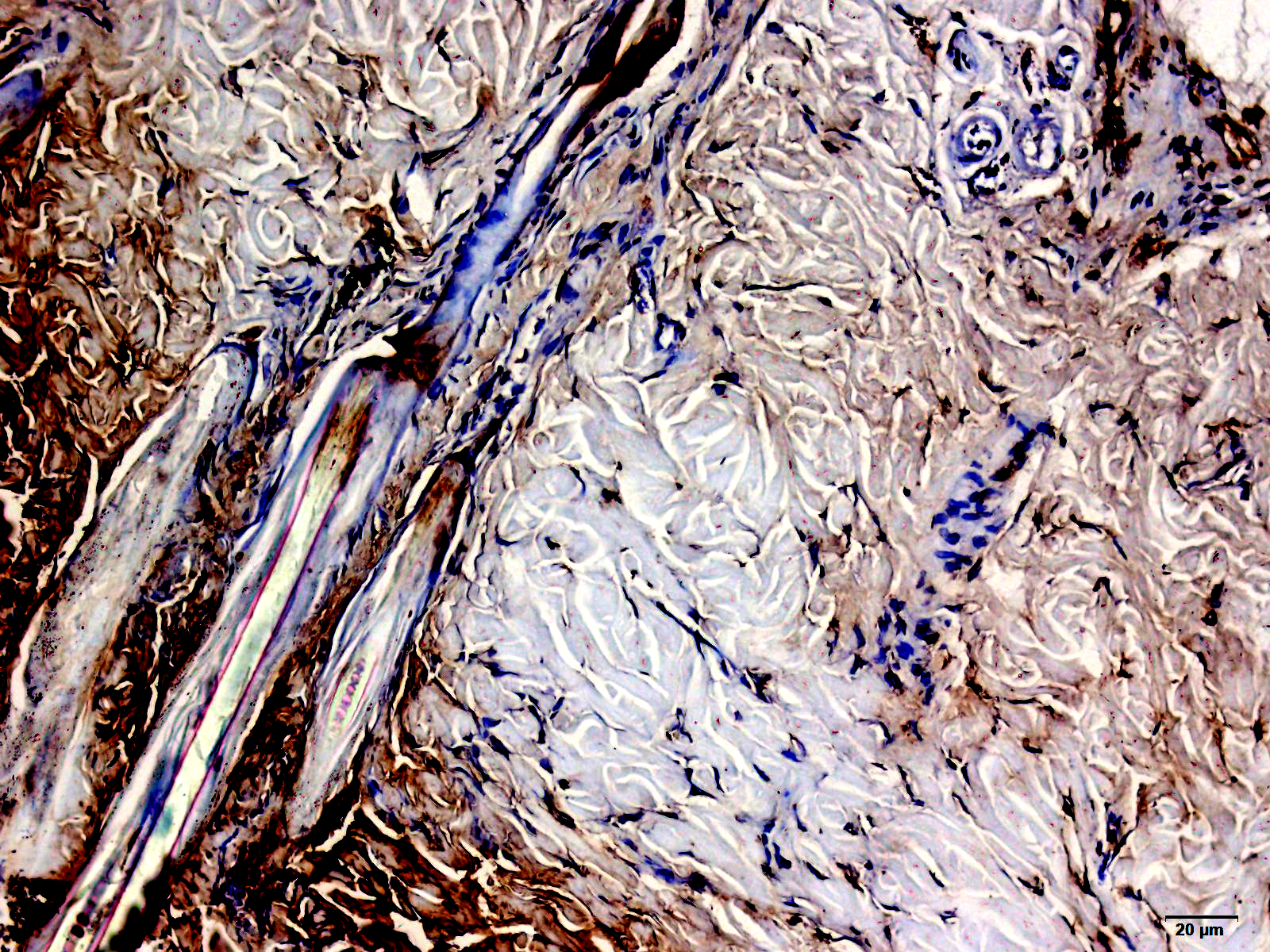

Supplement: S5 File — (ZIP) [file pone.0330078.s005.zip › Tunel/14D/HAMCC/HAMCC 1.tif]

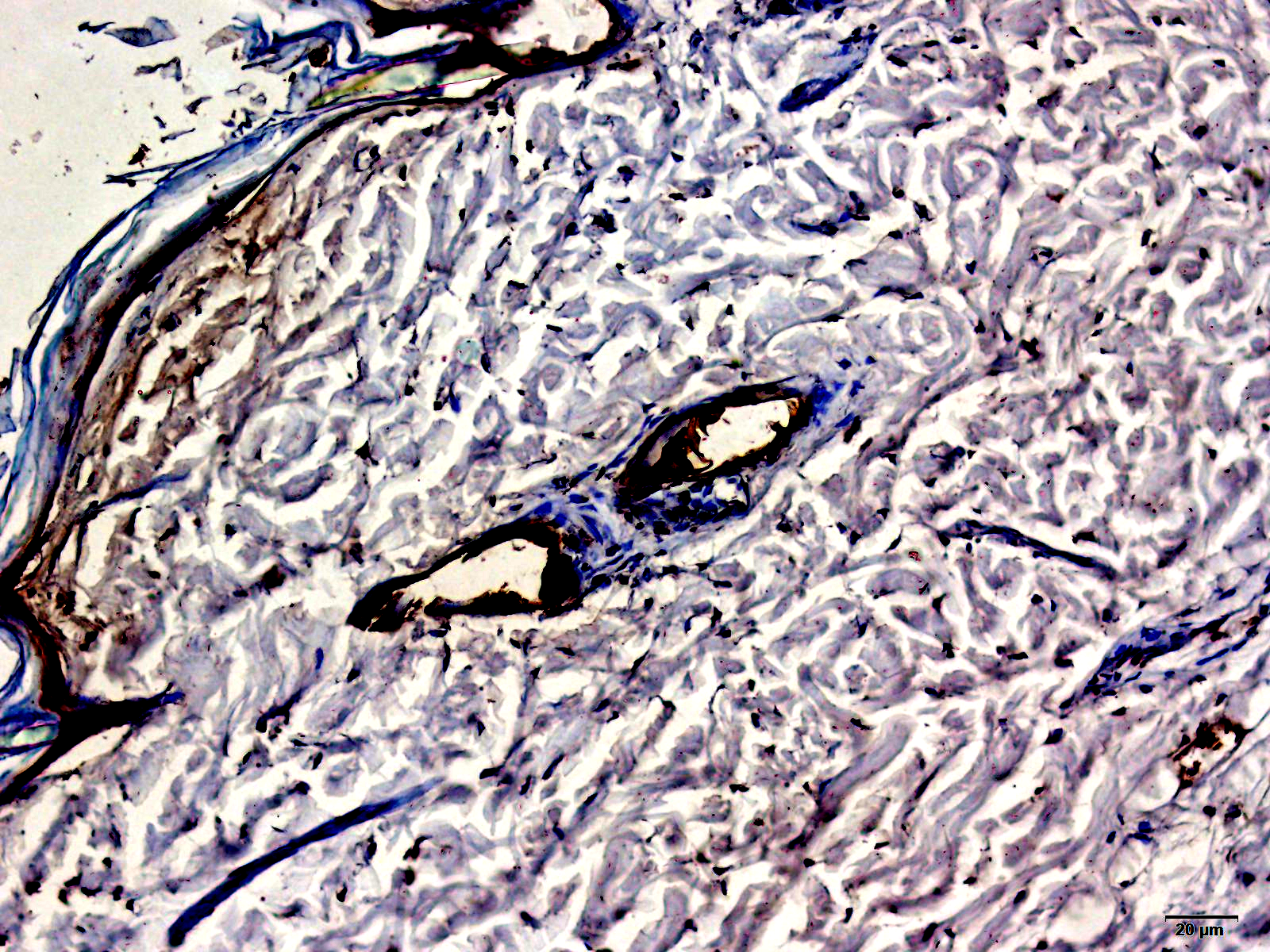

Supplement: S5 File — (ZIP) [file pone.0330078.s005.zip › Tunel/14D/HAMCC/HAMCC 2.tif]

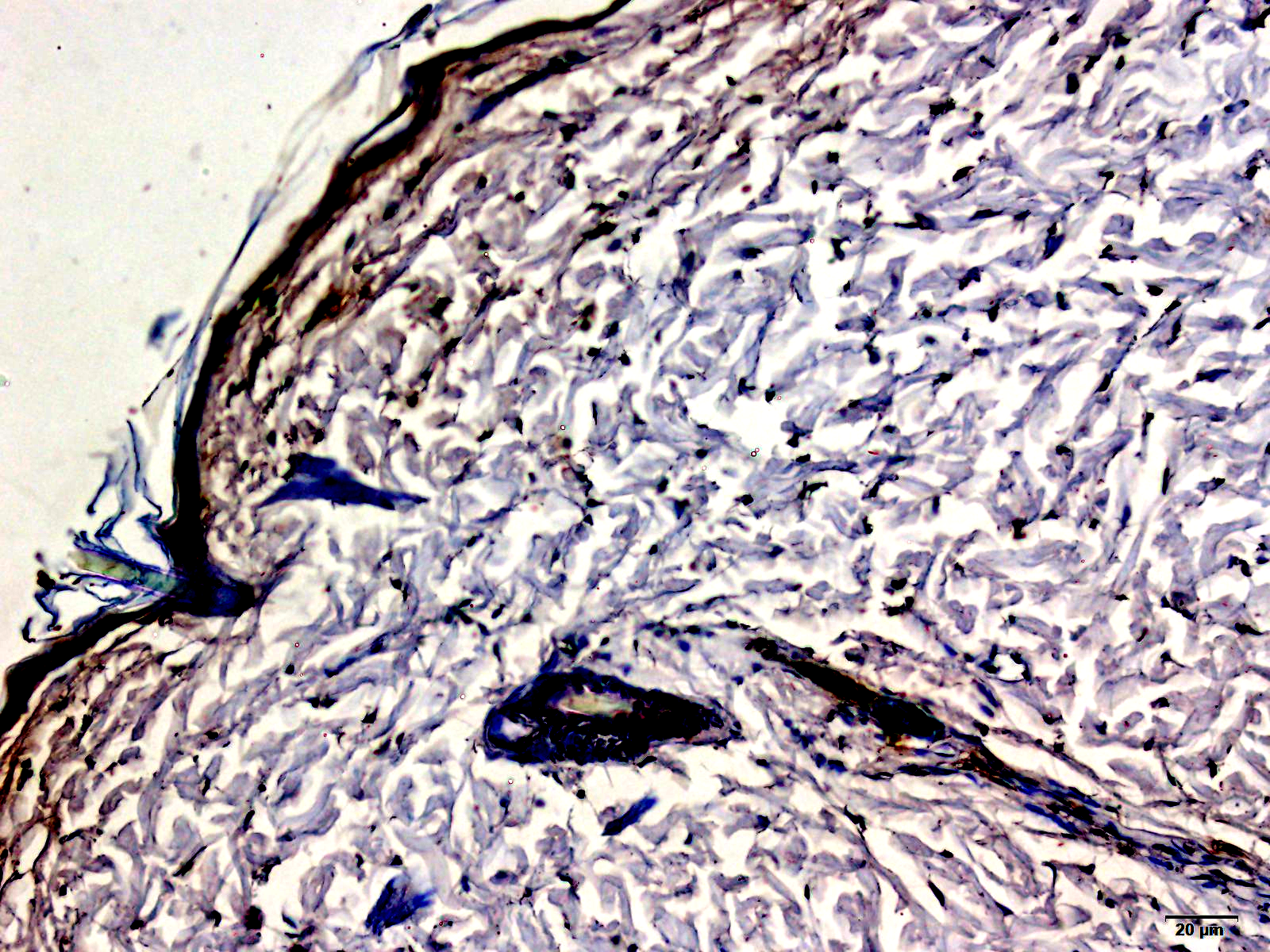

Supplement: S5 File — (ZIP) [file pone.0330078.s005.zip › Tunel/14D/HAMCC/HAMCC 3.tif]

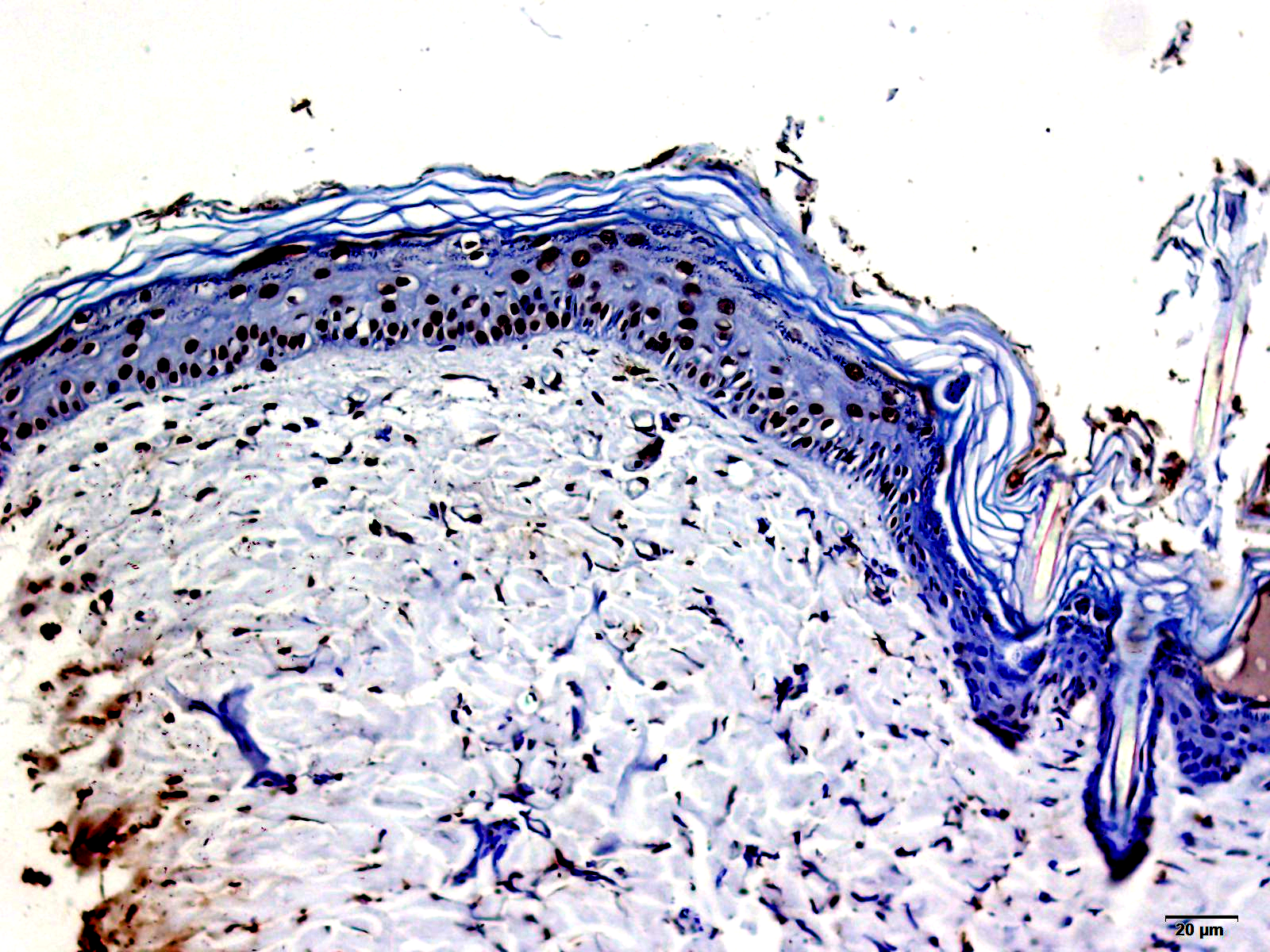

Supplement: S5 File — (ZIP) [file pone.0330078.s005.zip › Tunel/28D/CGF/CGF 1.tif]

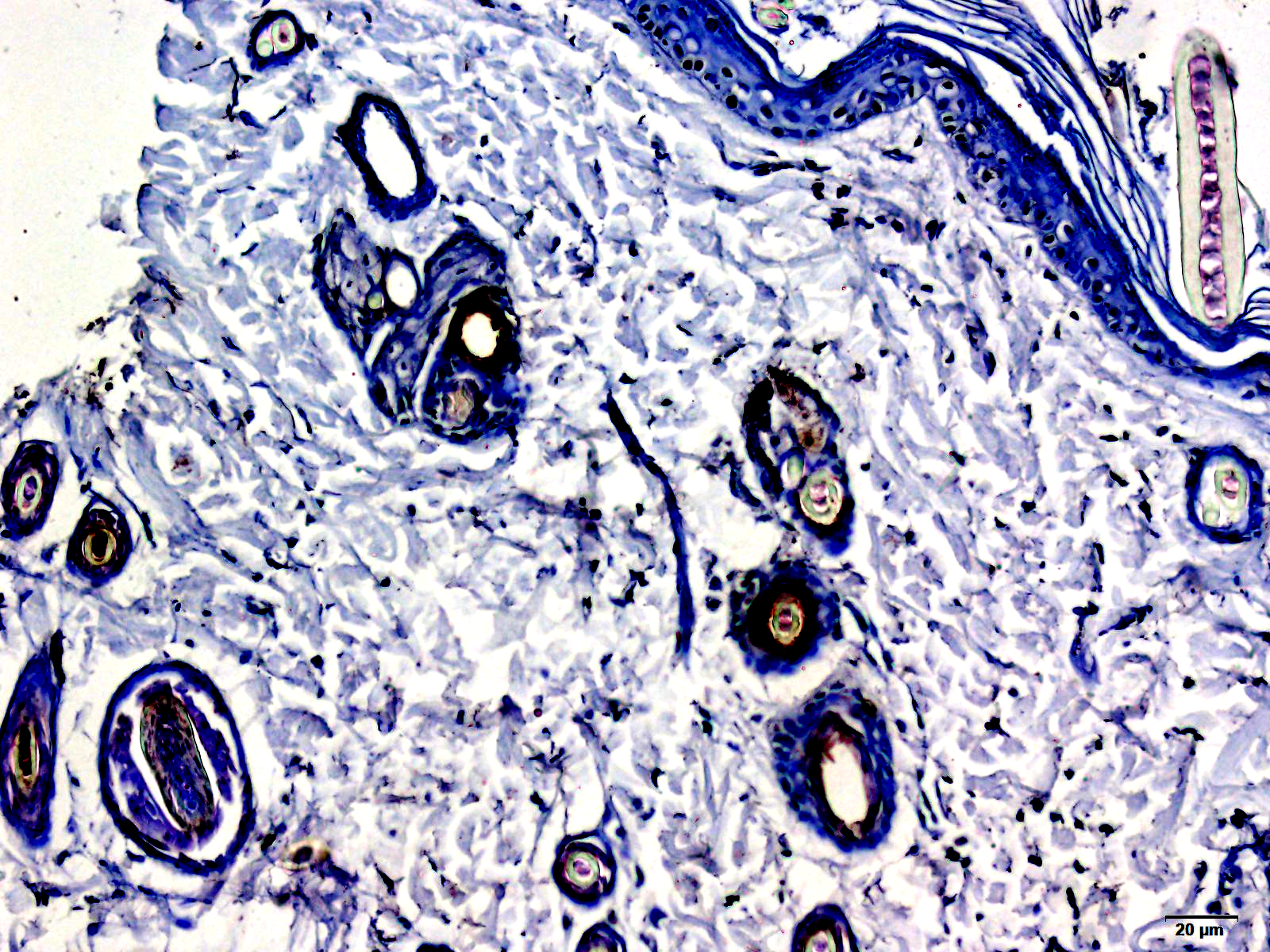

Supplement: S5 File — (ZIP) [file pone.0330078.s005.zip › Tunel/28D/CGF/CGF 2.tif]

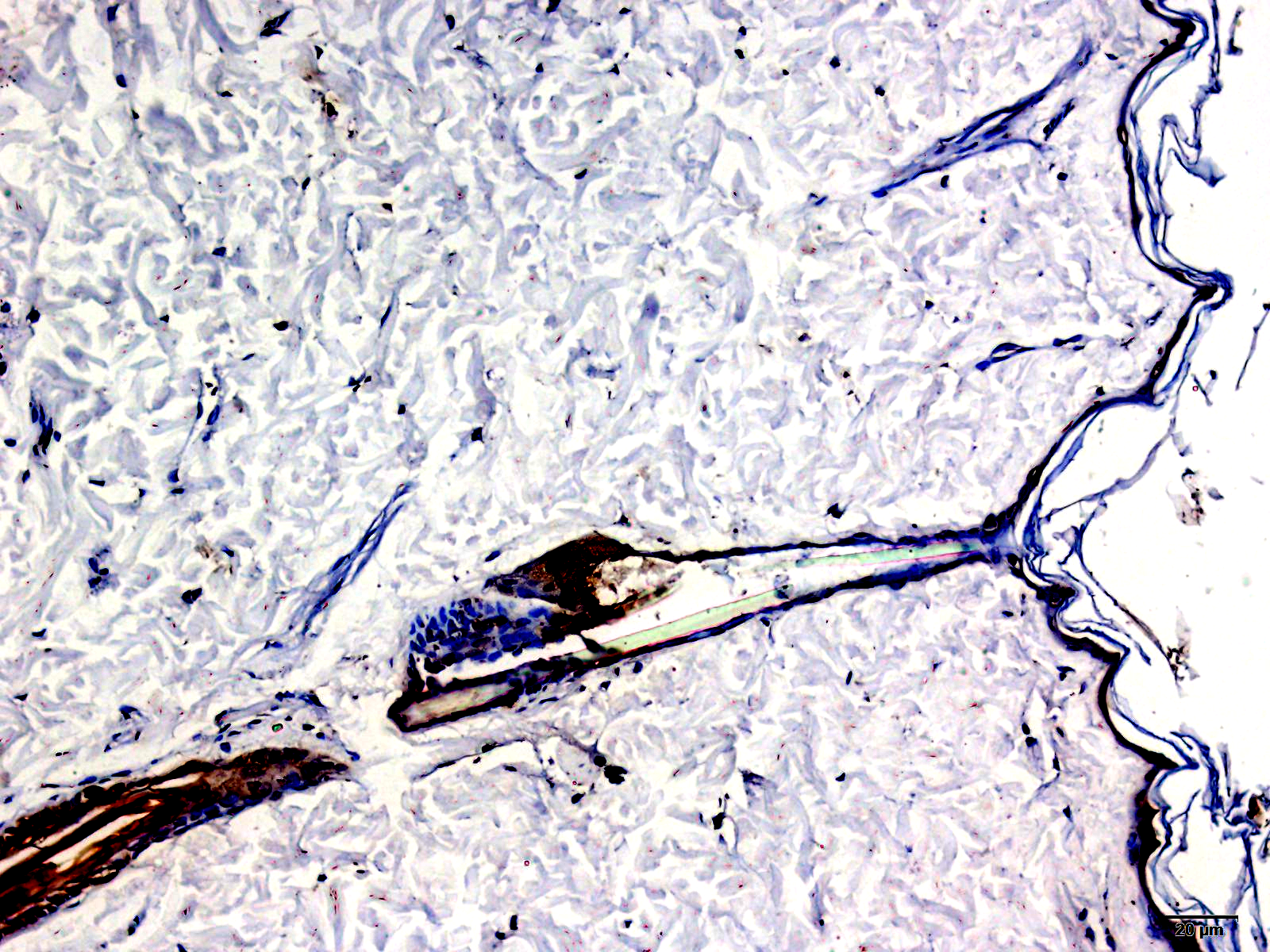

Supplement: S5 File — (ZIP) [file pone.0330078.s005.zip › Tunel/28D/CGF/CGF 3.tif]

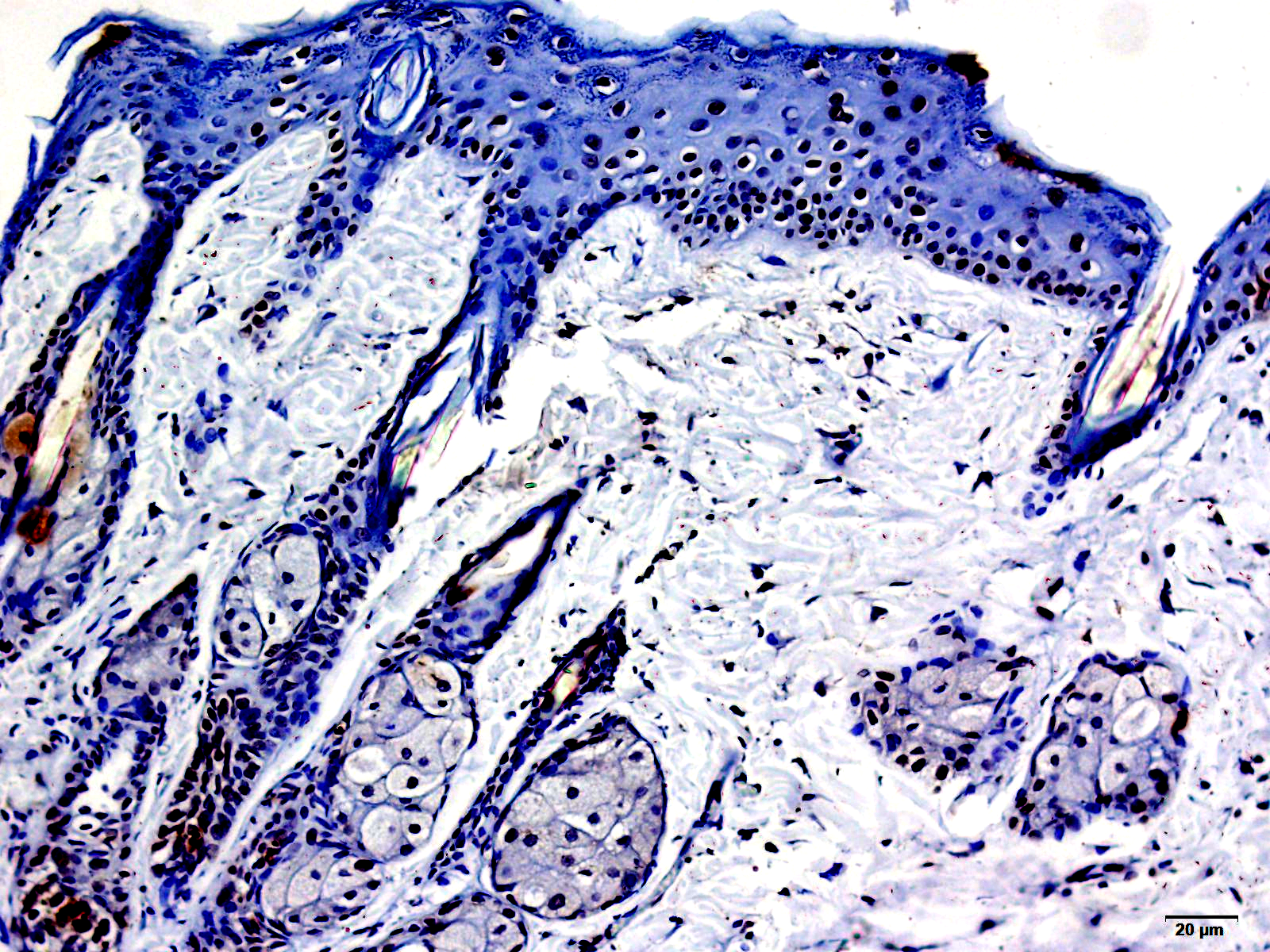

Supplement: S5 File — (ZIP) [file pone.0330078.s005.zip › Tunel/28D/CGF+HAMCC/CGF+ HAMCC 1.tif]

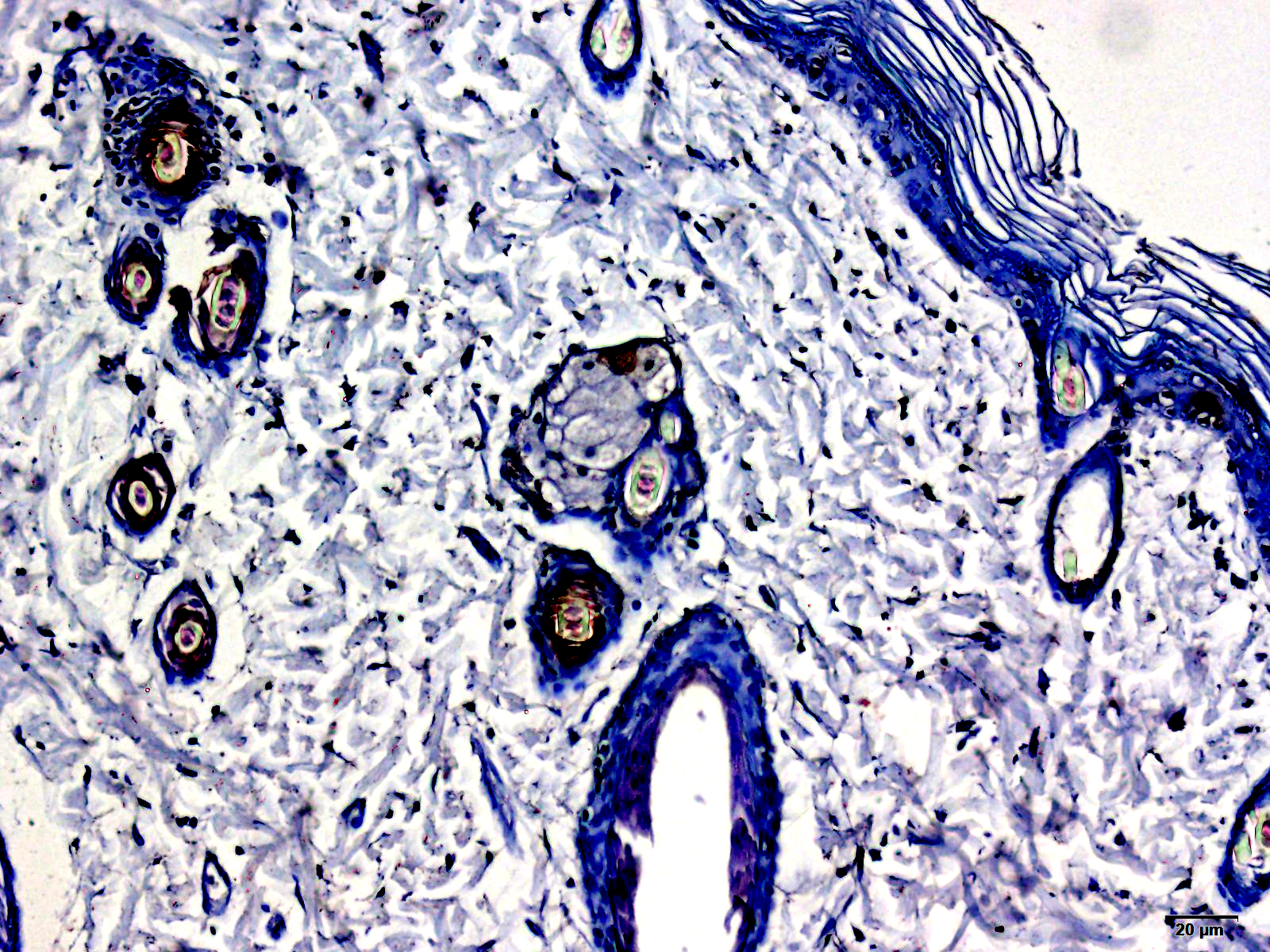

Supplement: S5 File — (ZIP) [file pone.0330078.s005.zip › Tunel/28D/CGF+HAMCC/CGF+ HAMCC 2.tif]

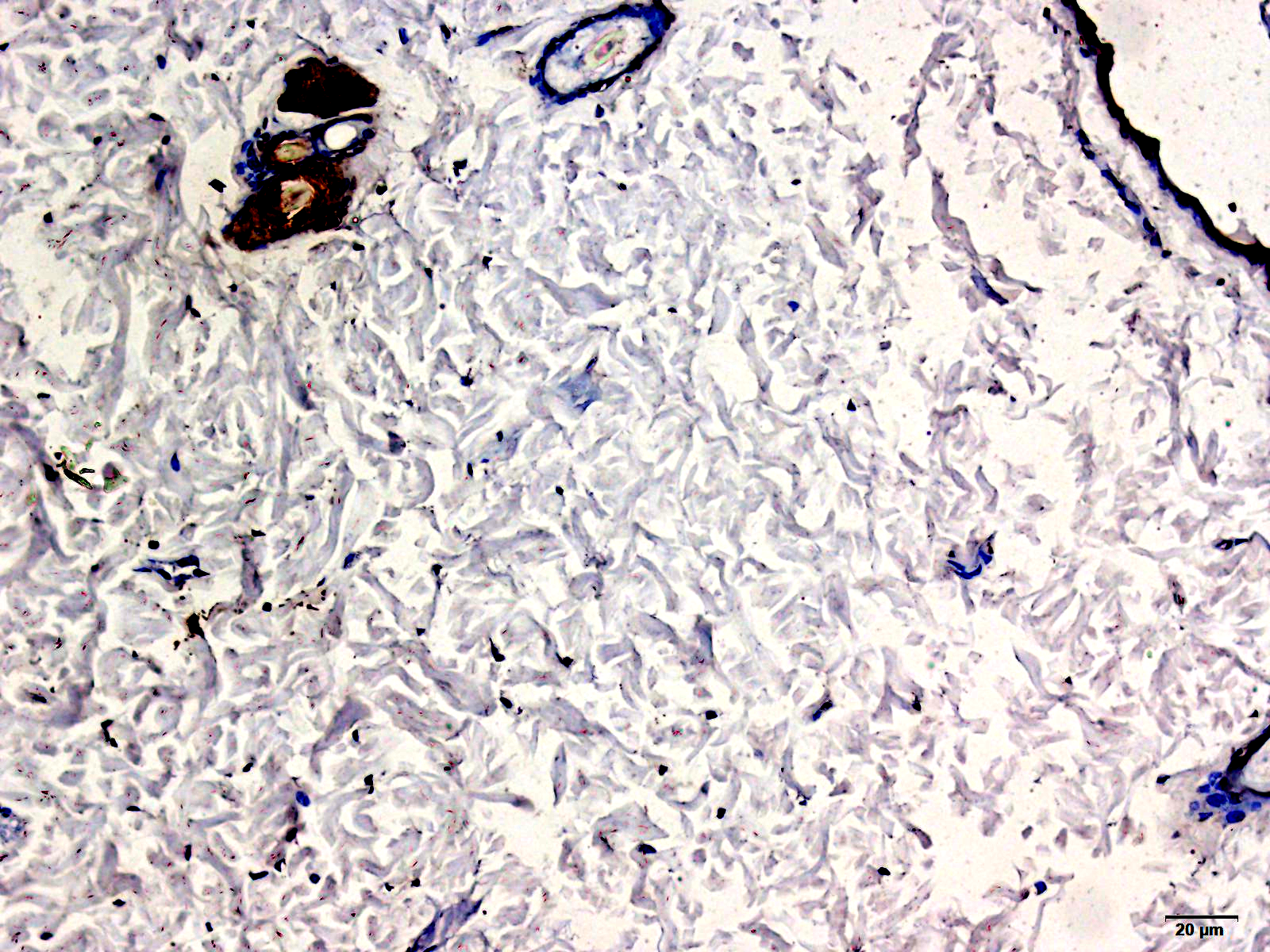

Supplement: S5 File — (ZIP) [file pone.0330078.s005.zip › Tunel/28D/CGF+HAMCC/CGF+ HAMCC 3.tif]

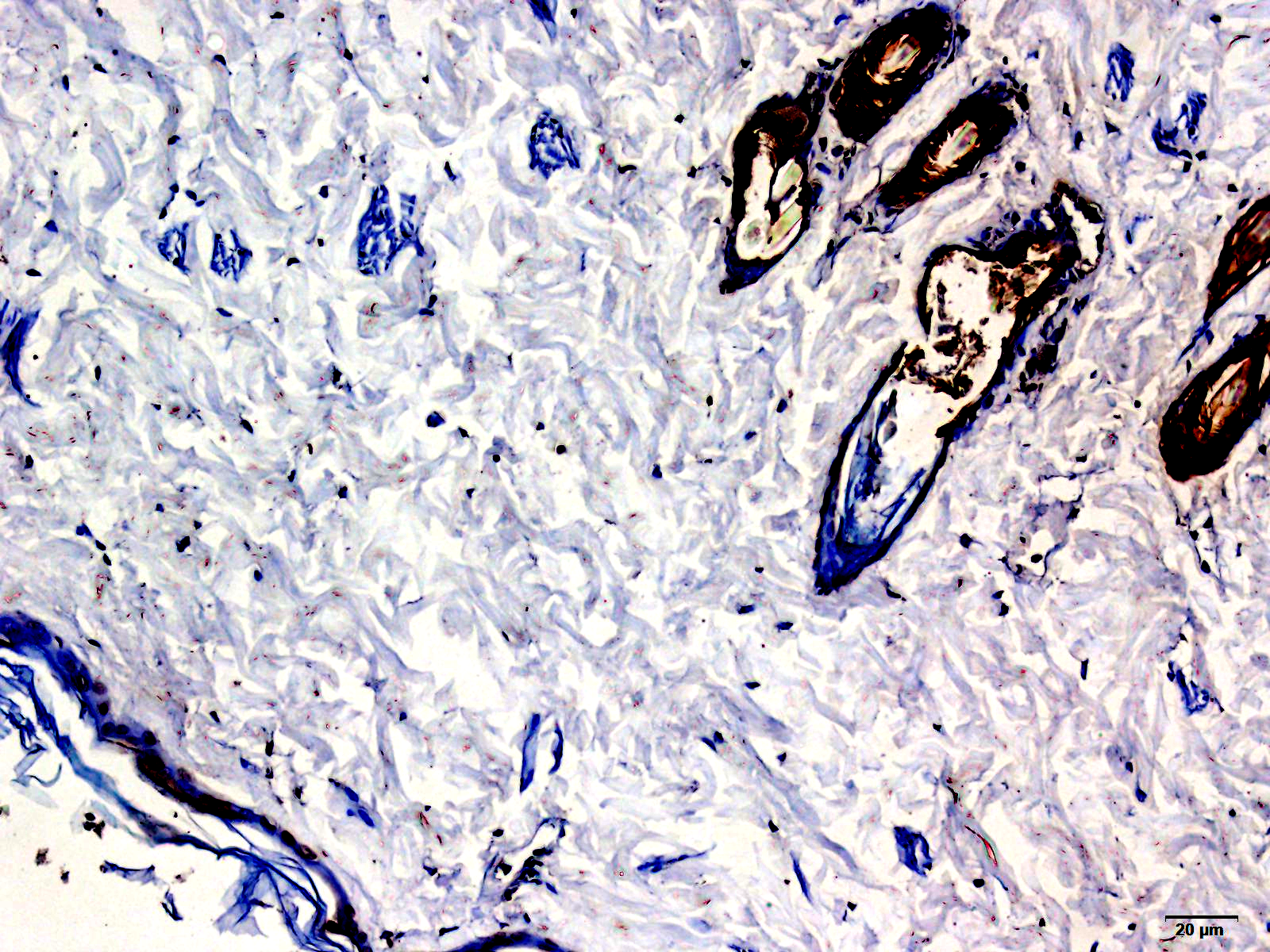

Supplement: S5 File — (ZIP) [file pone.0330078.s005.zip › Tunel/28D/Control/Control 1.tif]

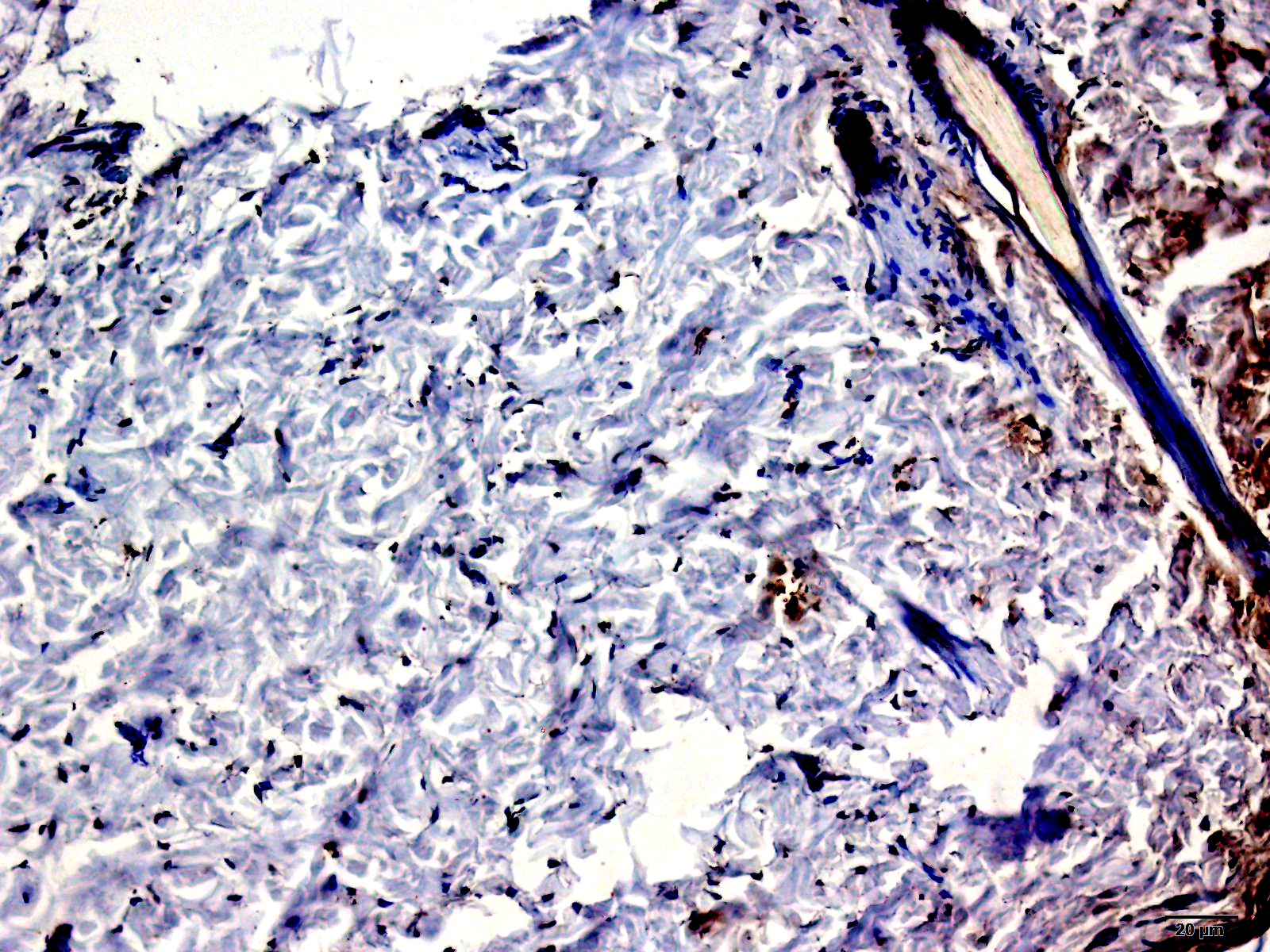

Supplement: S5 File — (ZIP) [file pone.0330078.s005.zip › Tunel/28D/Control/Control 2.tif]

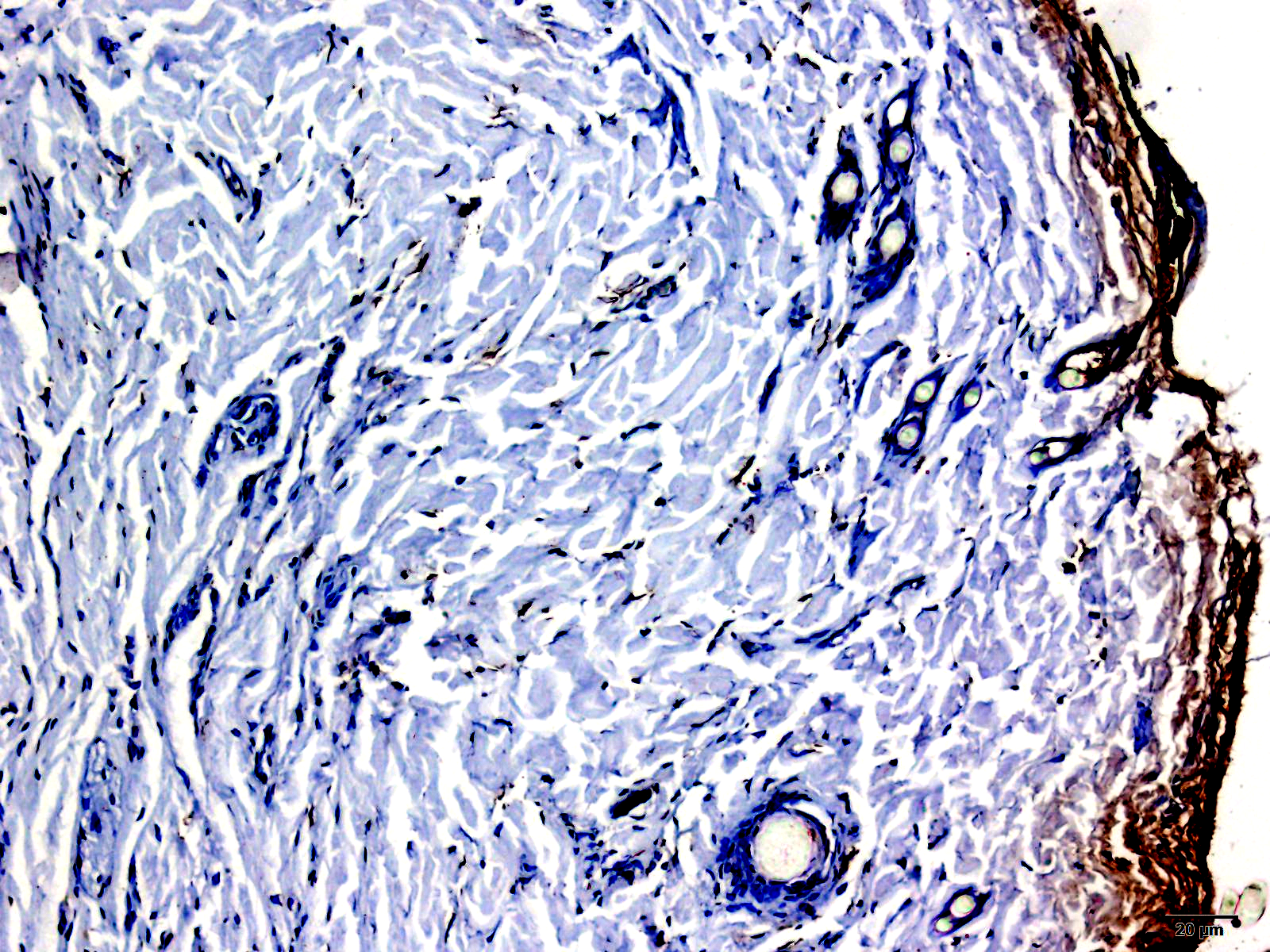

Supplement: S5 File — (ZIP) [file pone.0330078.s005.zip › Tunel/28D/Control/Control 3.tif]

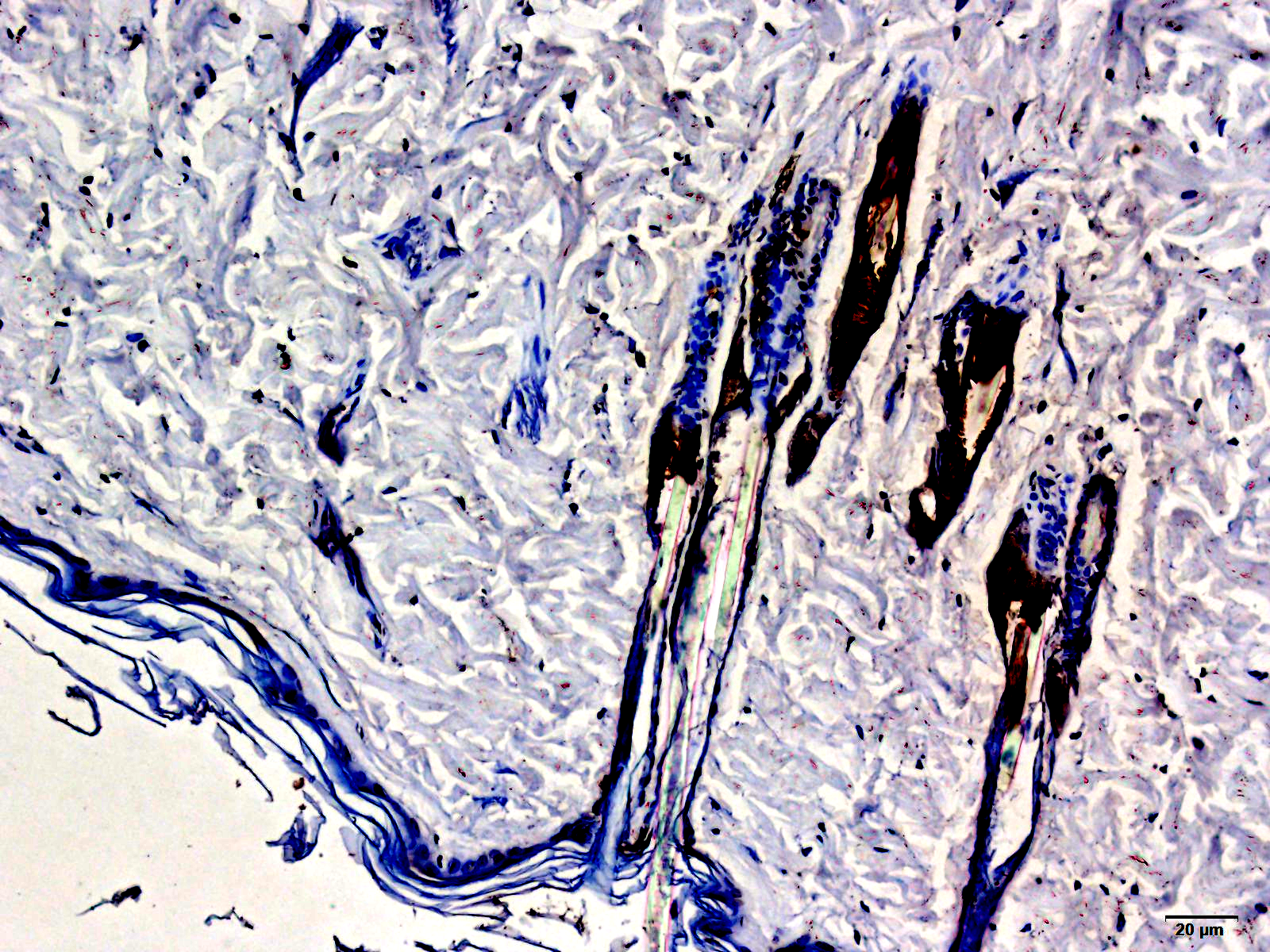

Supplement: S5 File — (ZIP) [file pone.0330078.s005.zip › Tunel/28D/HAMCC/HAMCC 1.tif]

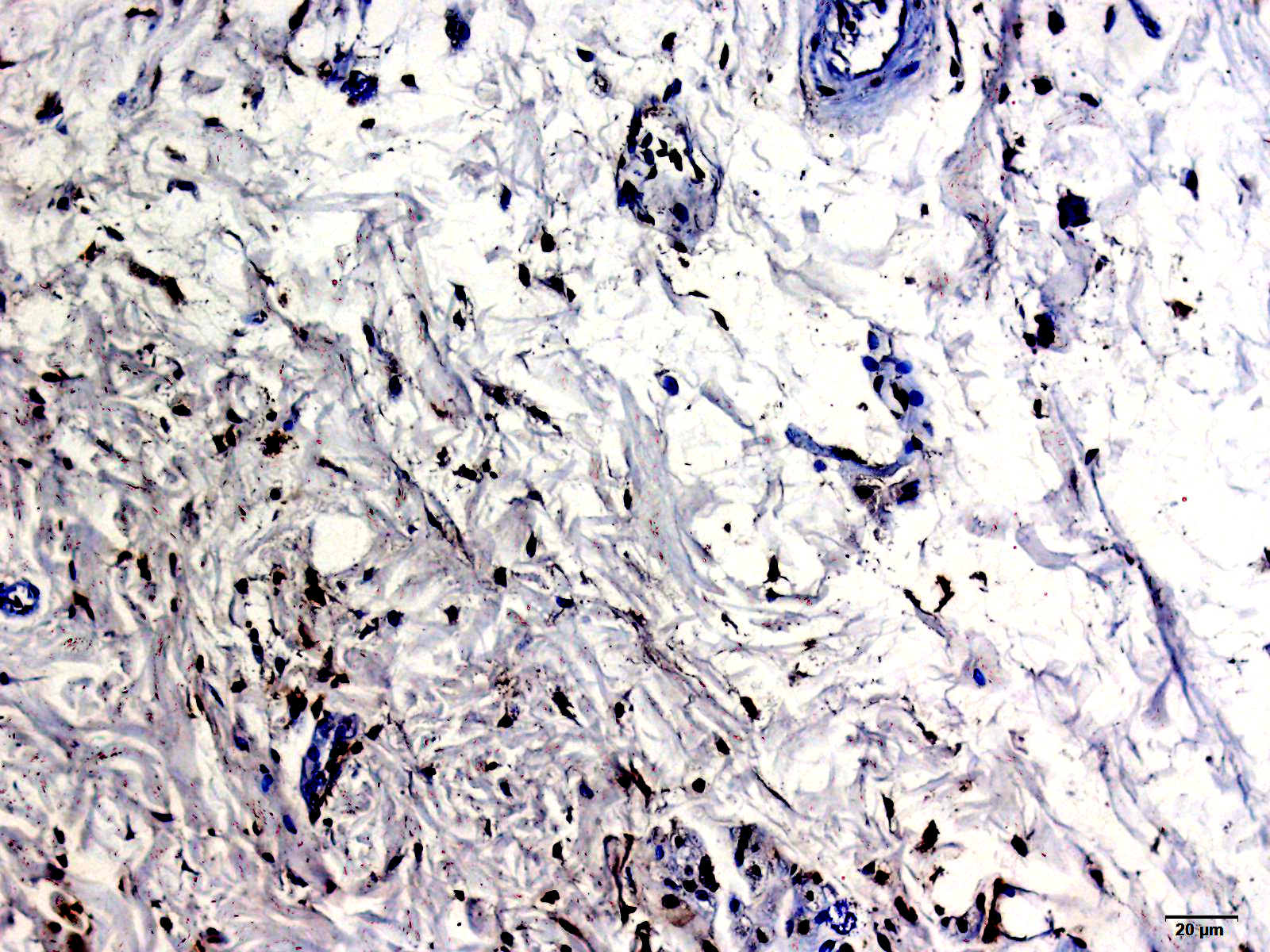

Supplement: S5 File — (ZIP) [file pone.0330078.s005.zip › Tunel/28D/HAMCC/HAMCC 2.tif]

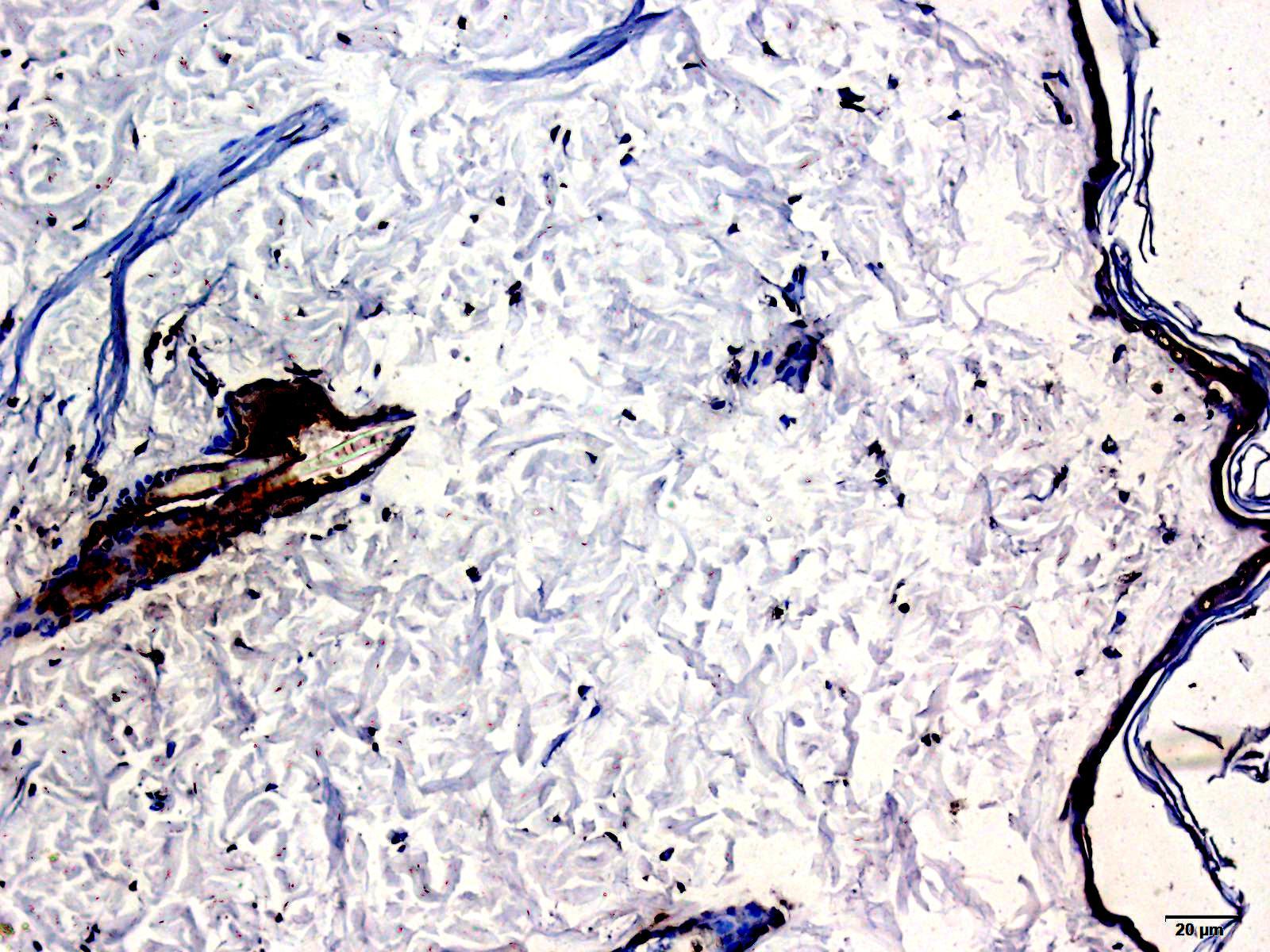

Supplement: S5 File — (ZIP) [file pone.0330078.s005.zip › Tunel/28D/HAMCC/HAMCC 3.tif]

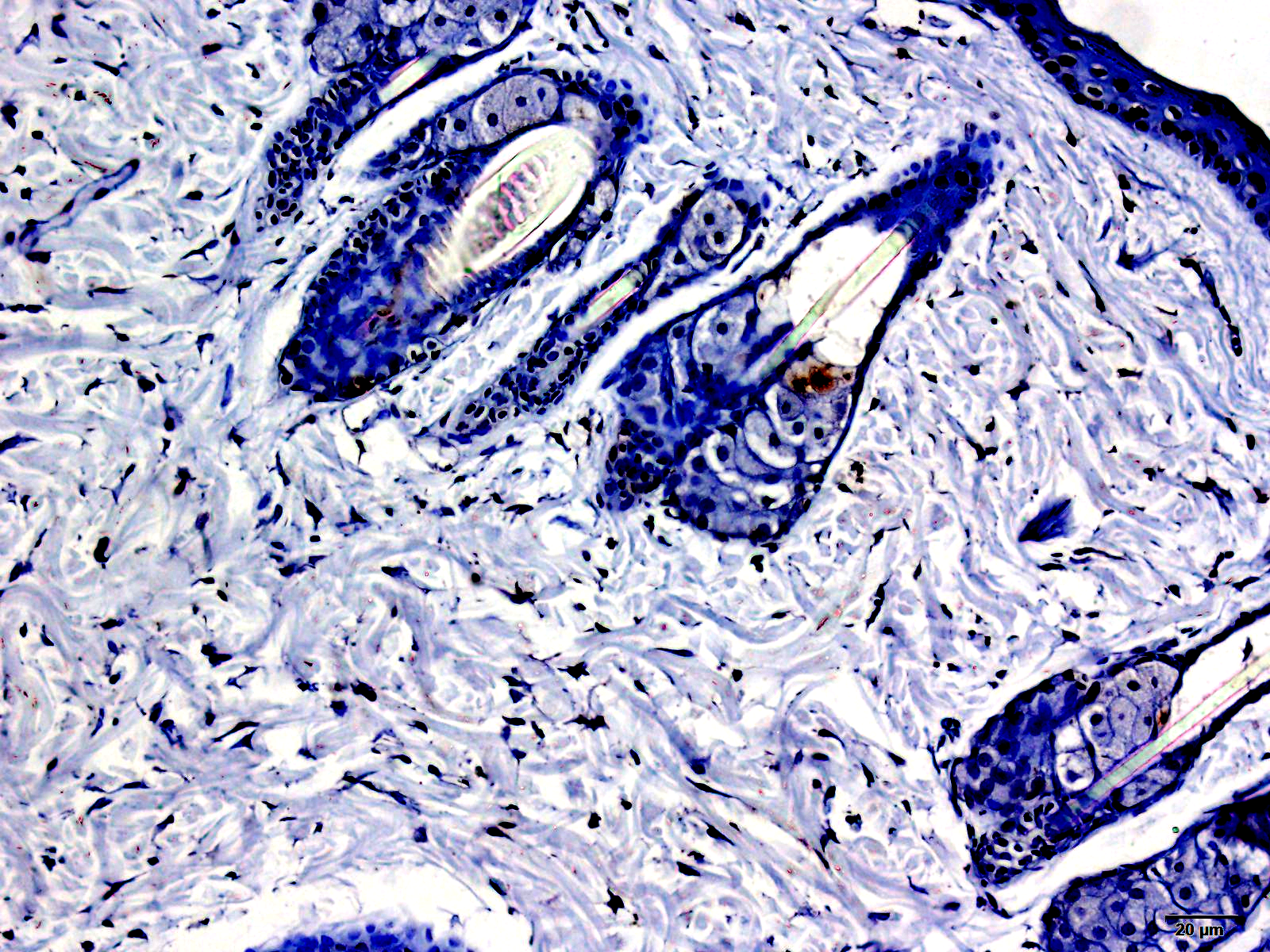

Supplement: S5 File — (ZIP) [file pone.0330078.s005.zip › Tunel/7D/CGF/CGF 1.tif]

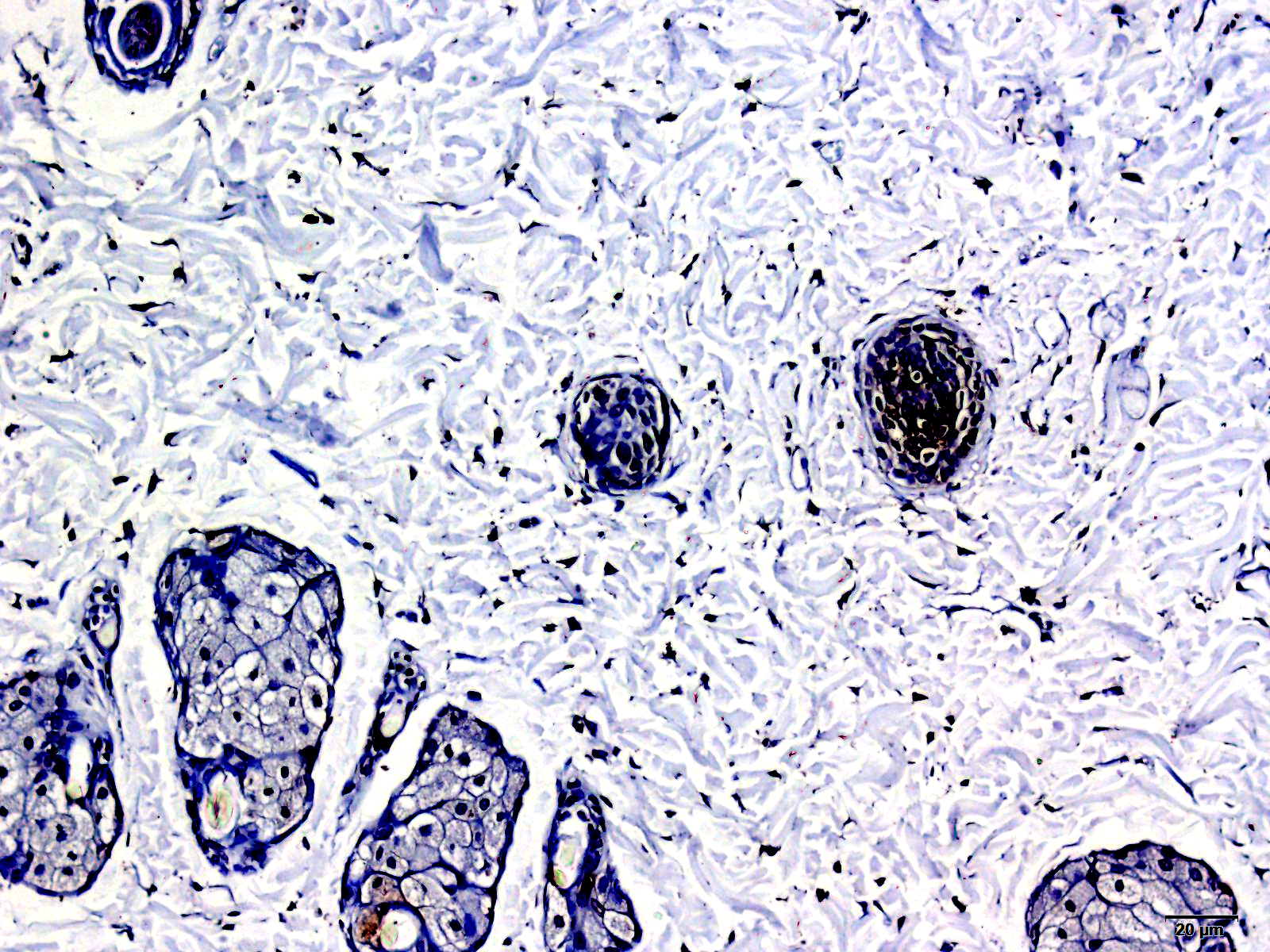

Supplement: S5 File — (ZIP) [file pone.0330078.s005.zip › Tunel/7D/CGF/CGF 2.tif]

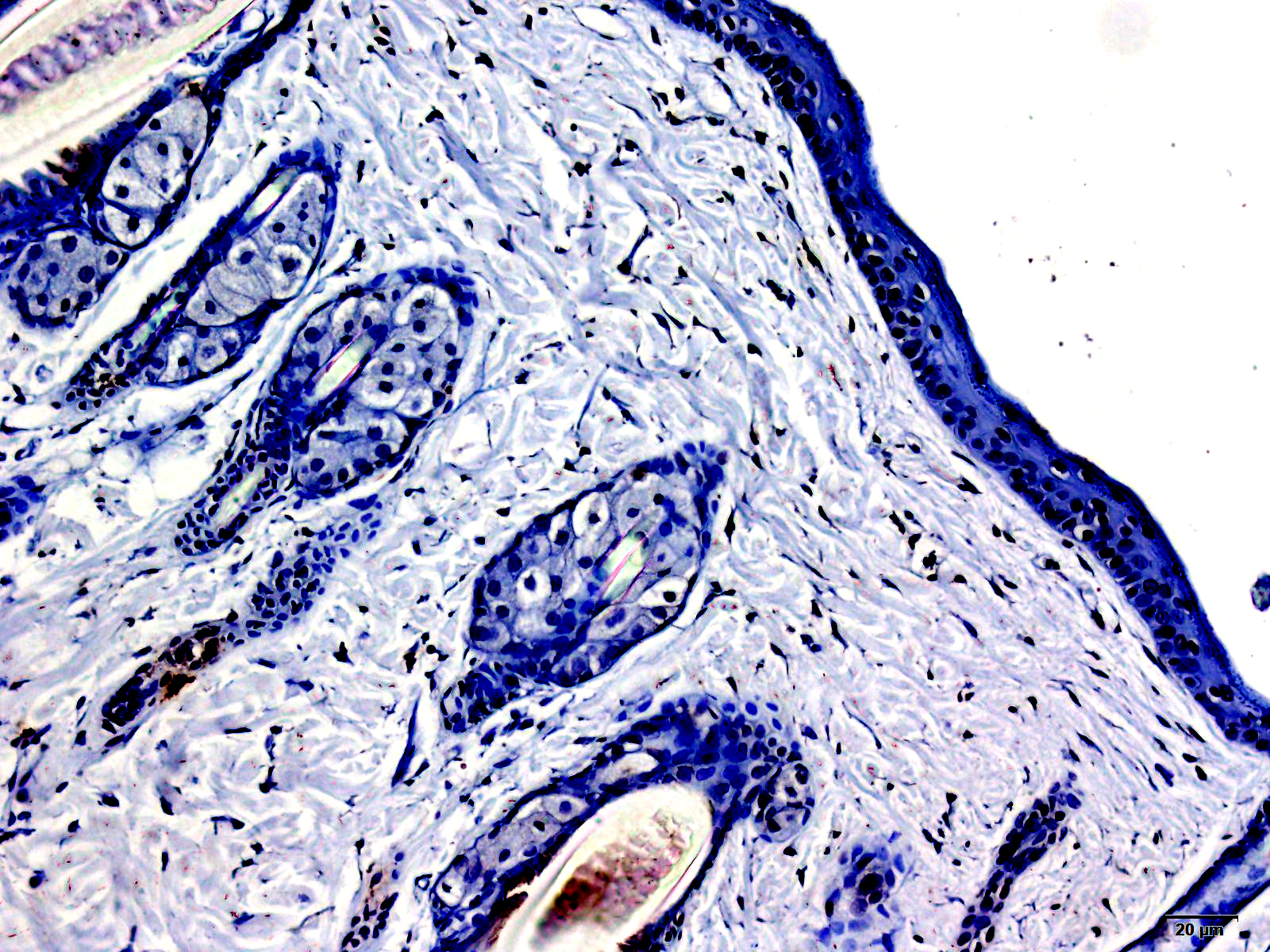

Supplement: S5 File — (ZIP) [file pone.0330078.s005.zip › Tunel/7D/CGF/CGF 3.tif]

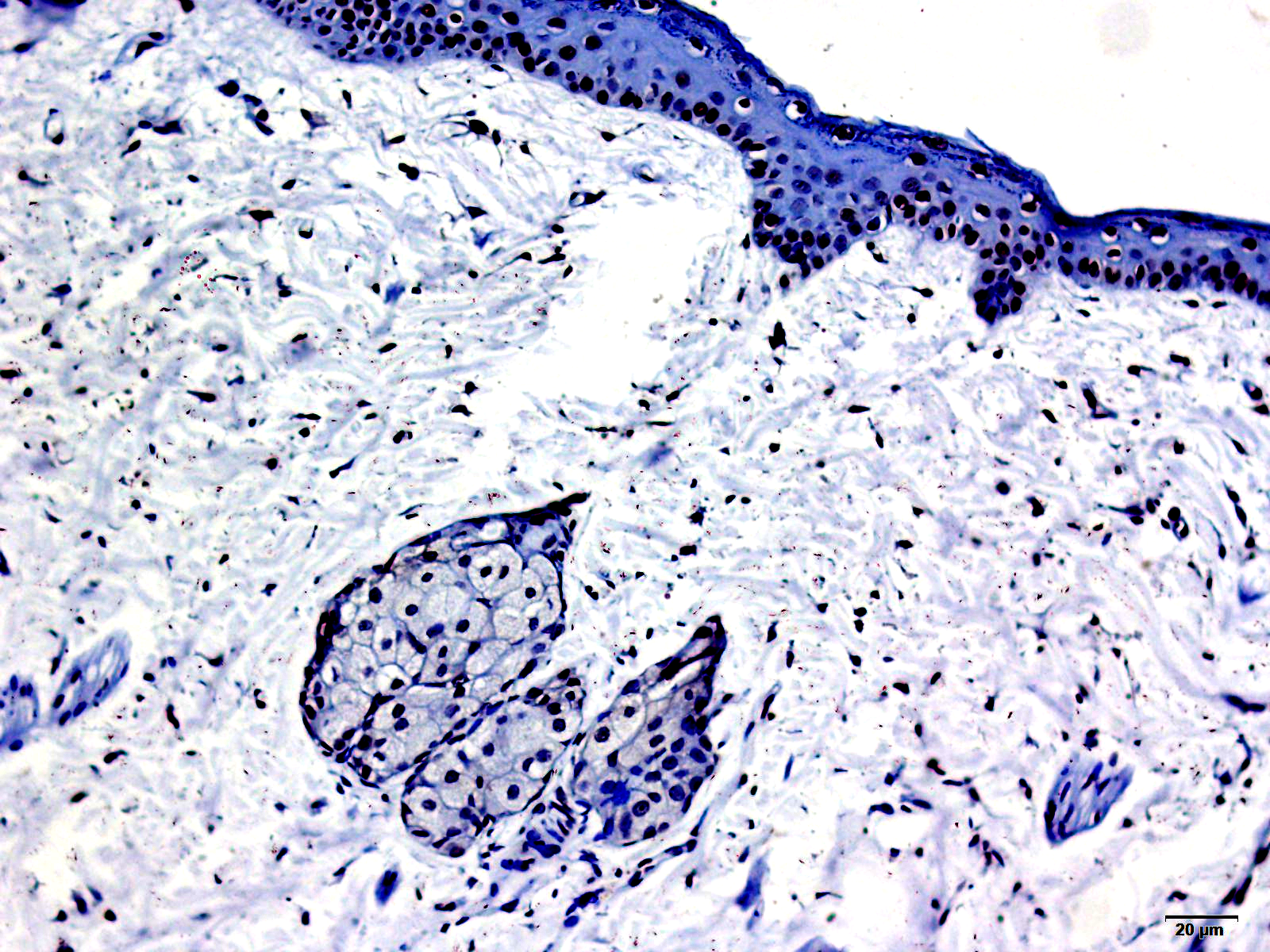

Supplement: S5 File — (ZIP) [file pone.0330078.s005.zip › Tunel/7D/CGF+HAMCC/CGF+HAMCC 1.tif]

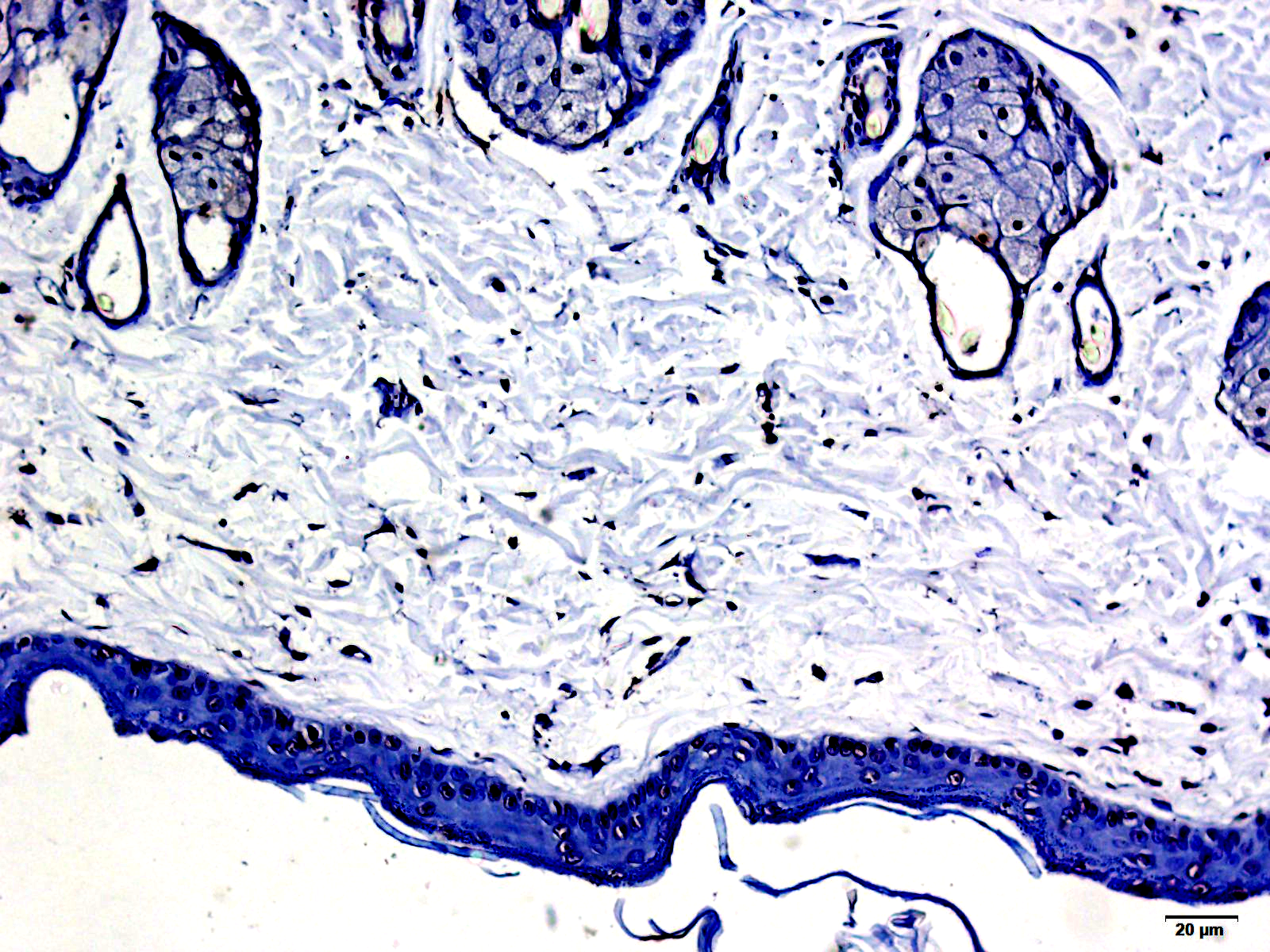

Supplement: S5 File — (ZIP) [file pone.0330078.s005.zip › Tunel/7D/CGF+HAMCC/CGF+HAMCC 2.tif]

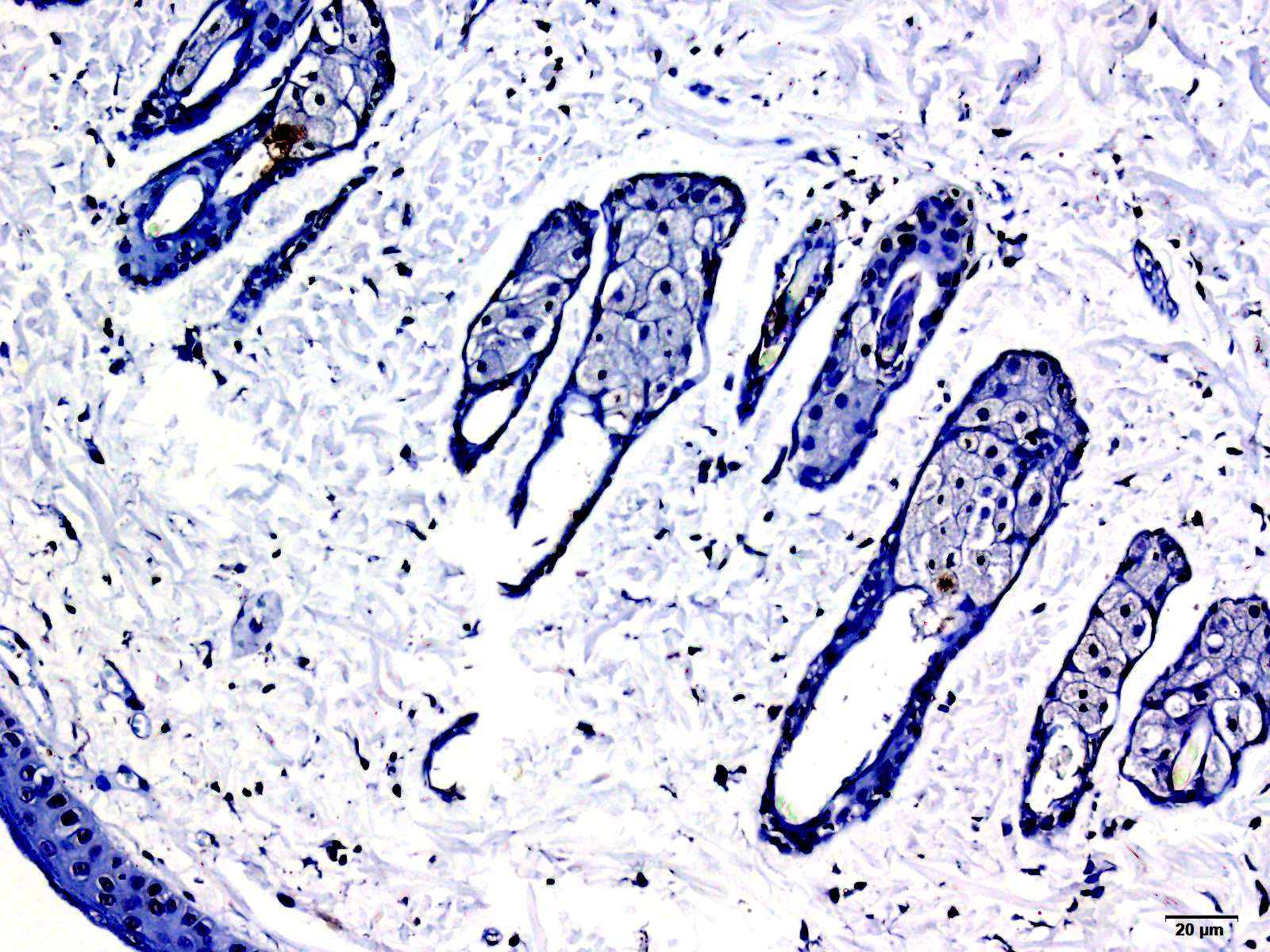

Supplement: S5 File — (ZIP) [file pone.0330078.s005.zip › Tunel/7D/CGF+HAMCC/CGF+HAMCC 3.tif]

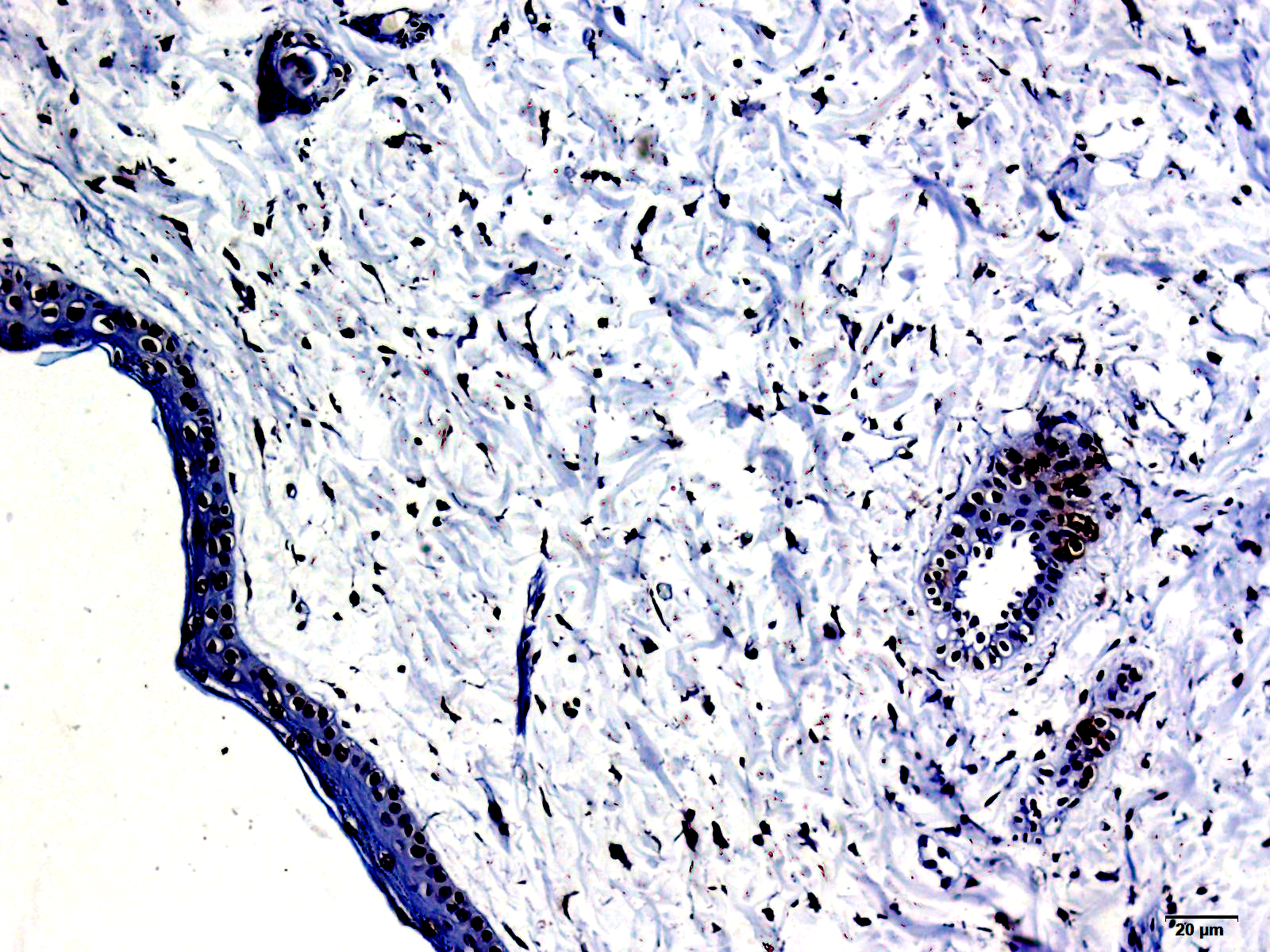

Supplement: S5 File — (ZIP) [file pone.0330078.s005.zip › Tunel/7D/Control/Control 1.tif]

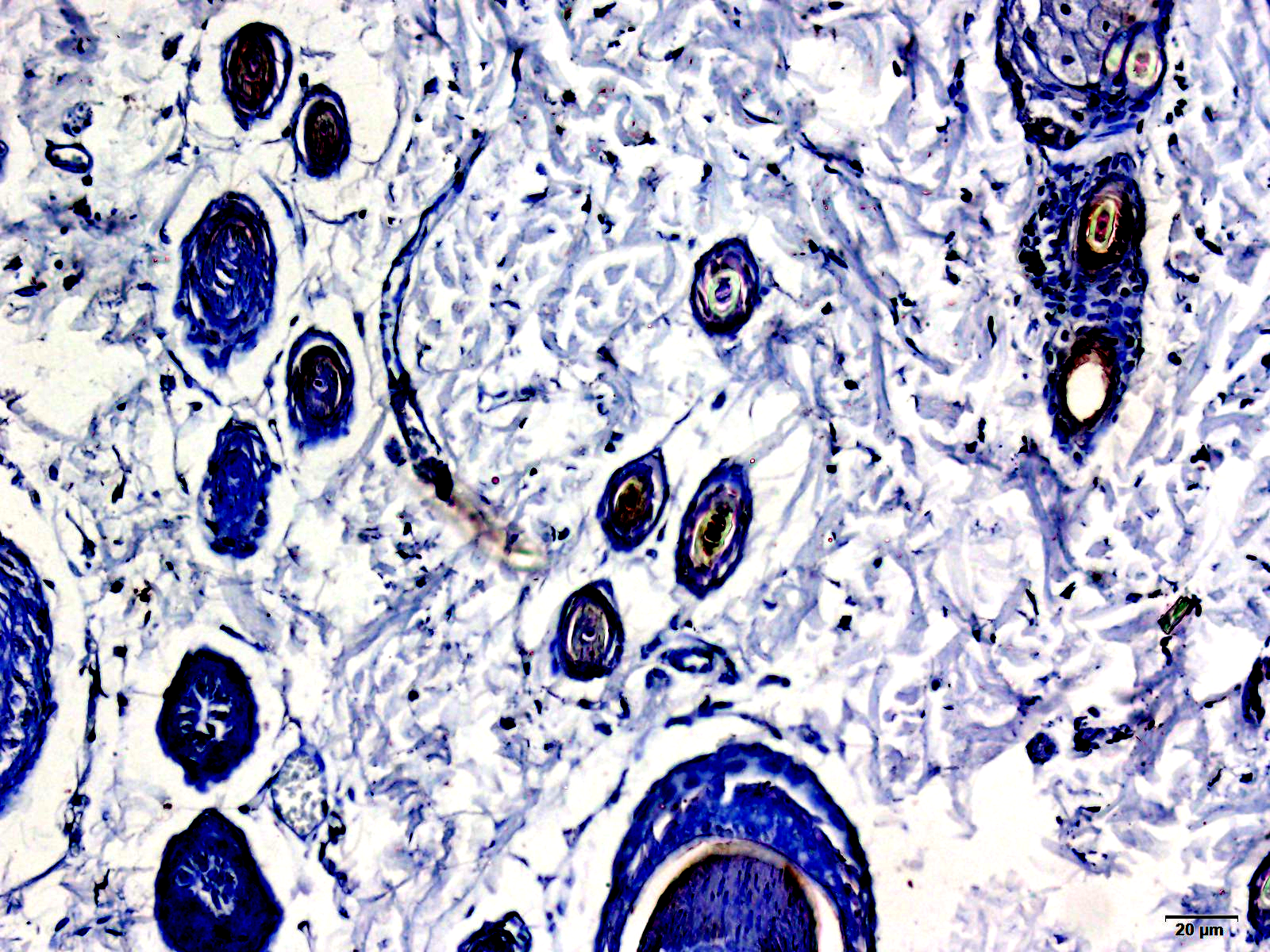

Supplement: S5 File — (ZIP) [file pone.0330078.s005.zip › Tunel/7D/Control/Control 2.tif]

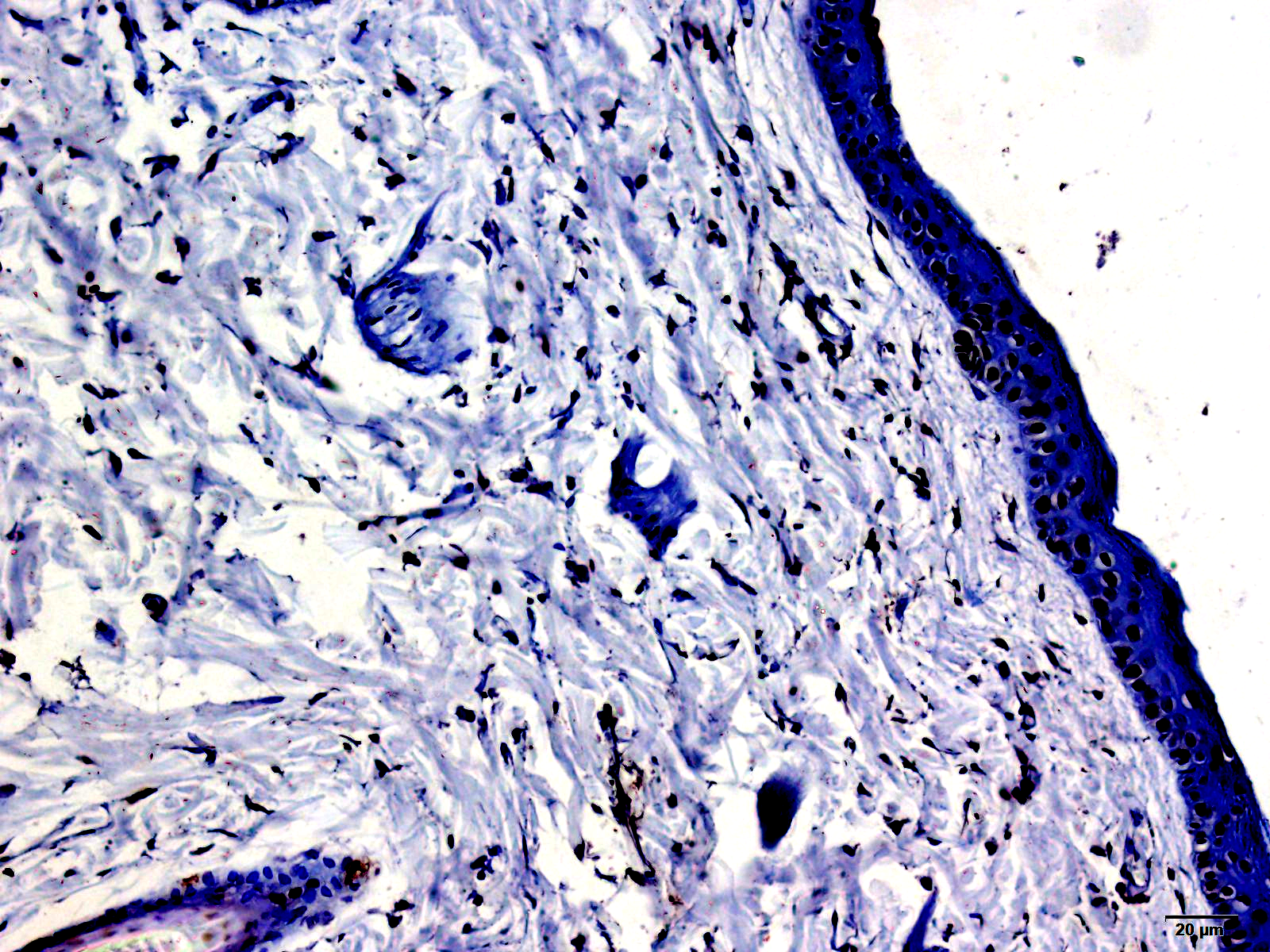

Supplement: S5 File — (ZIP) [file pone.0330078.s005.zip › Tunel/7D/Control/Control 3.tif]

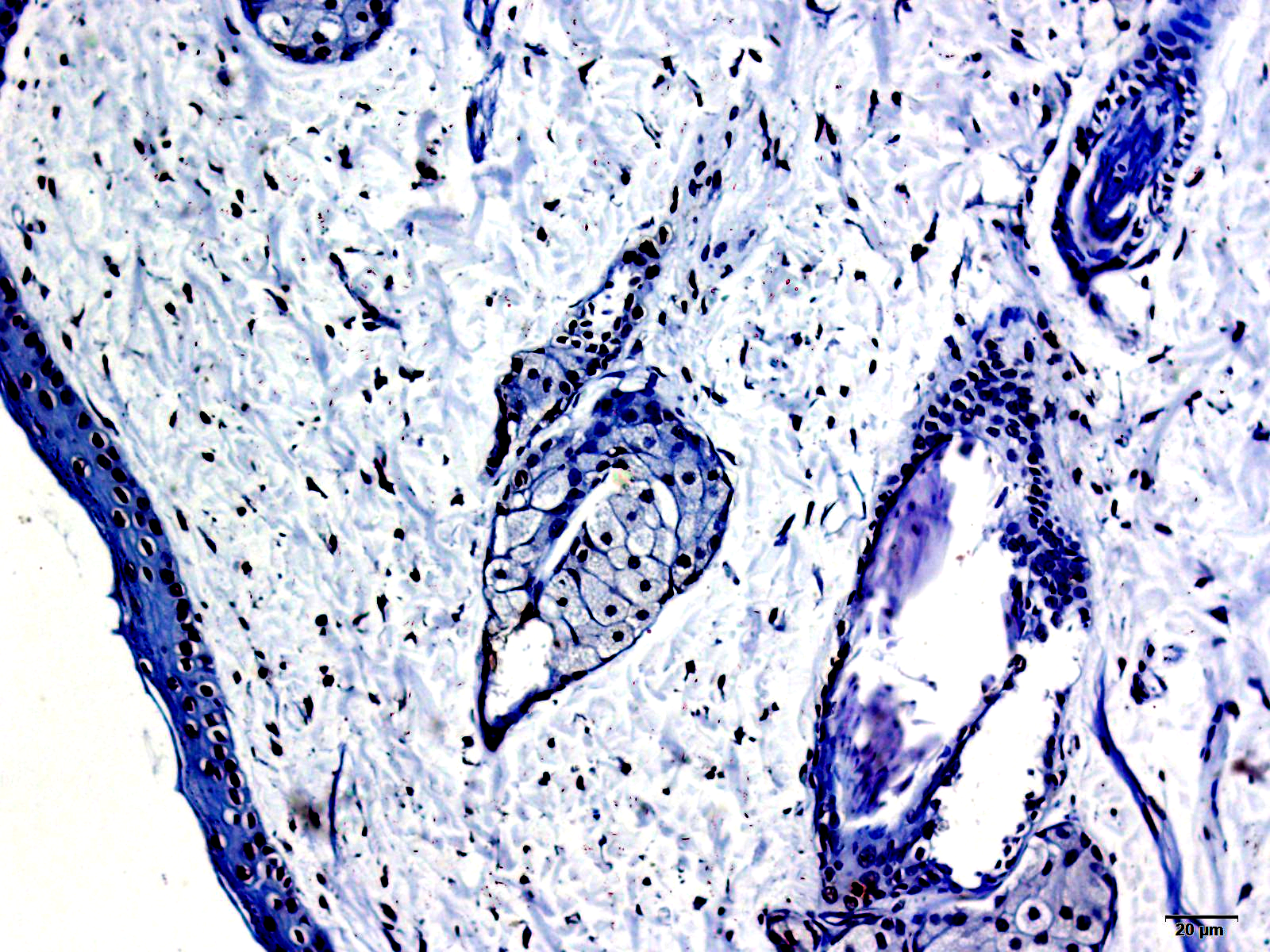

Supplement: S5 File — (ZIP) [file pone.0330078.s005.zip › Tunel/7D/HAMCC/HAMCC 1.tif]

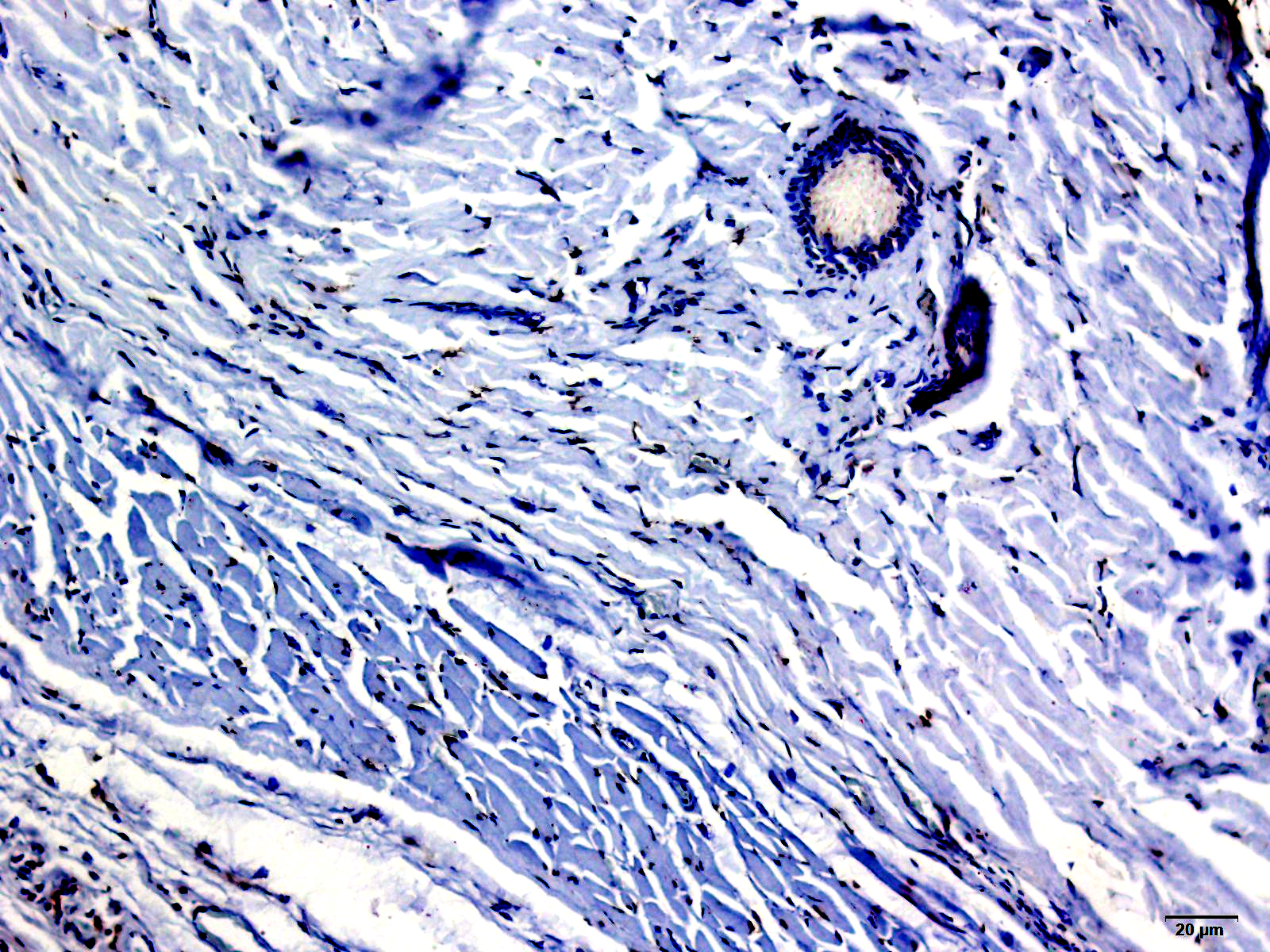

Supplement: S5 File — (ZIP) [file pone.0330078.s005.zip › Tunel/7D/HAMCC/HAMCC 2.tif]

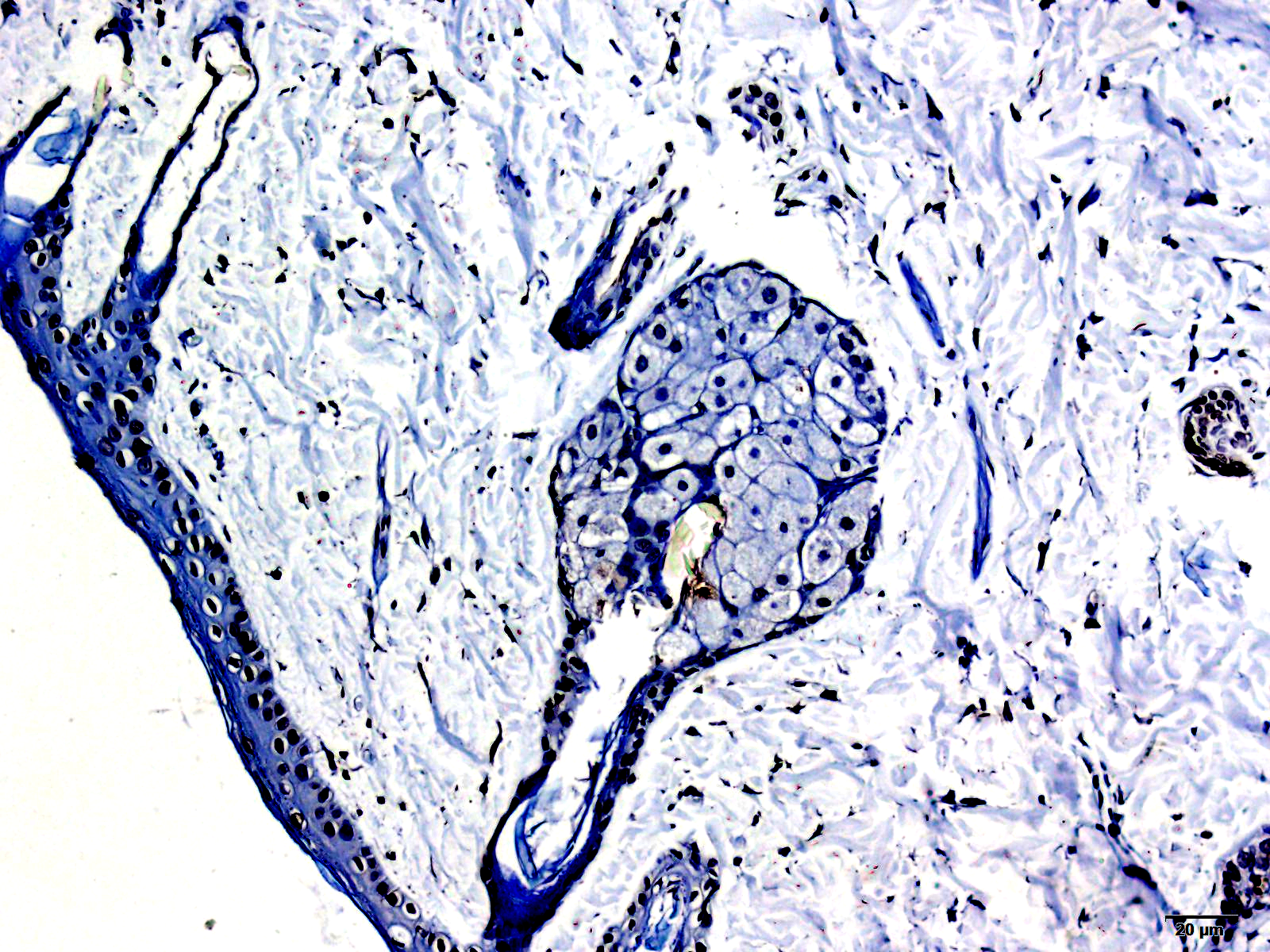

Supplement: S5 File — (ZIP) [file pone.0330078.s005.zip › Tunel/7D/HAMCC/HAMCC 3.tif]

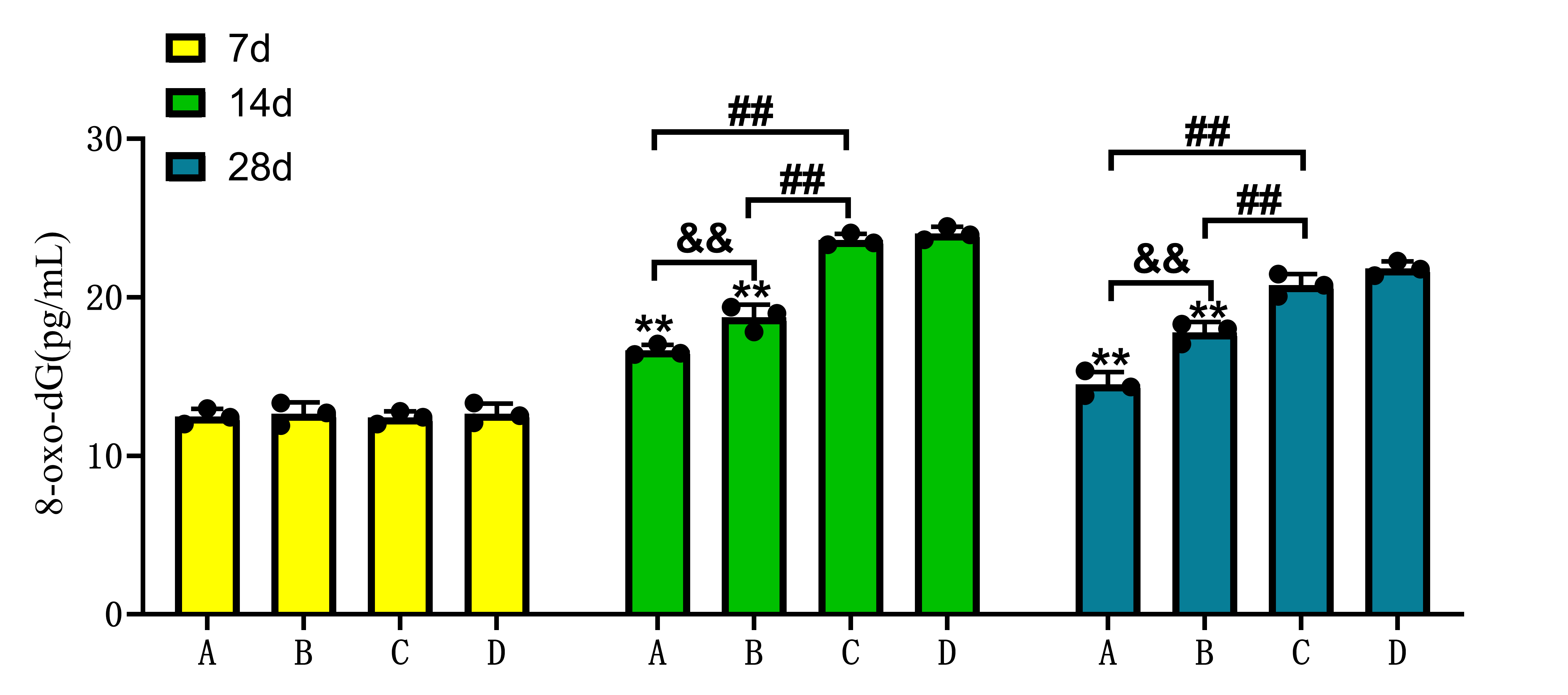

Supplement: S6 File — (ZIP) [file pone.0330078.s006.zip › ELISA/Figure/8-oxo-dG .tif]

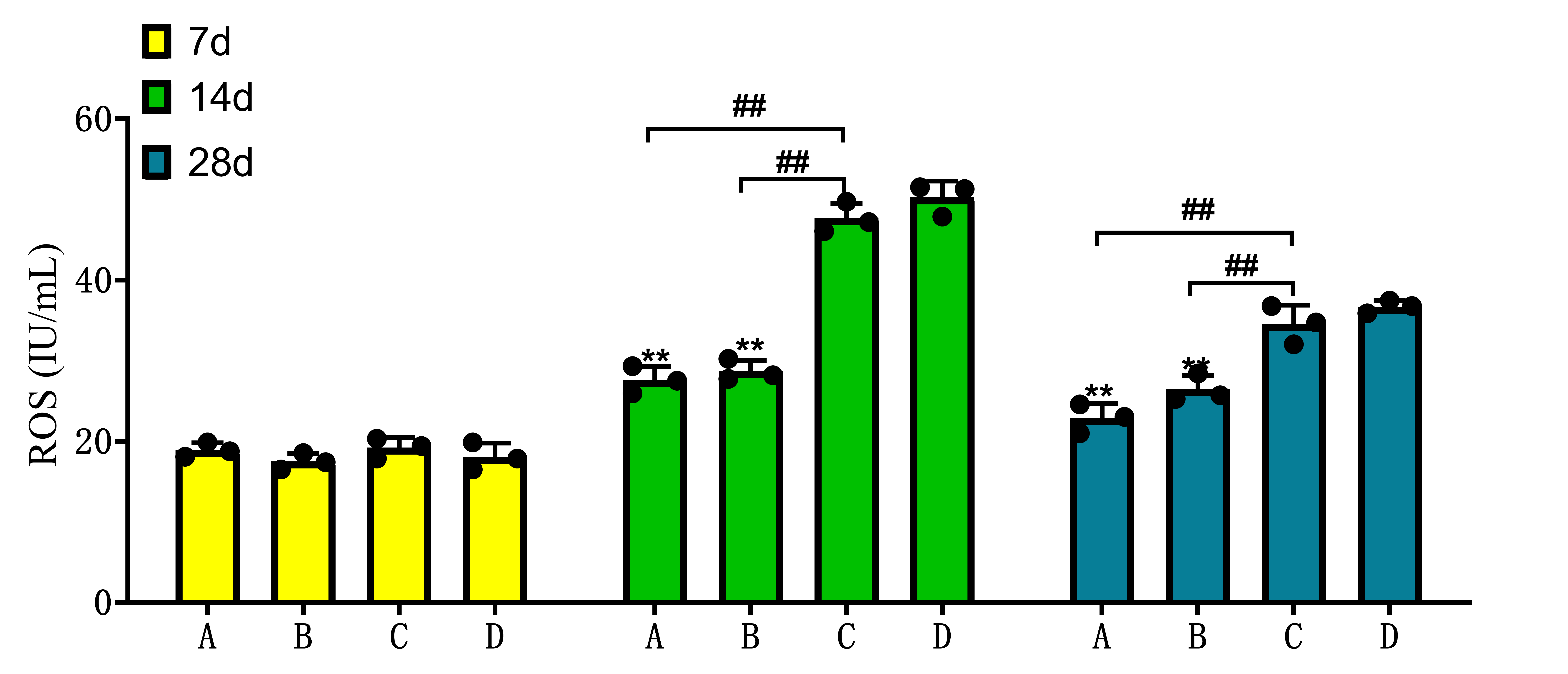

Supplement: S6 File — (ZIP) [file pone.0330078.s006.zip › ELISA/Figure/ROS.tif]

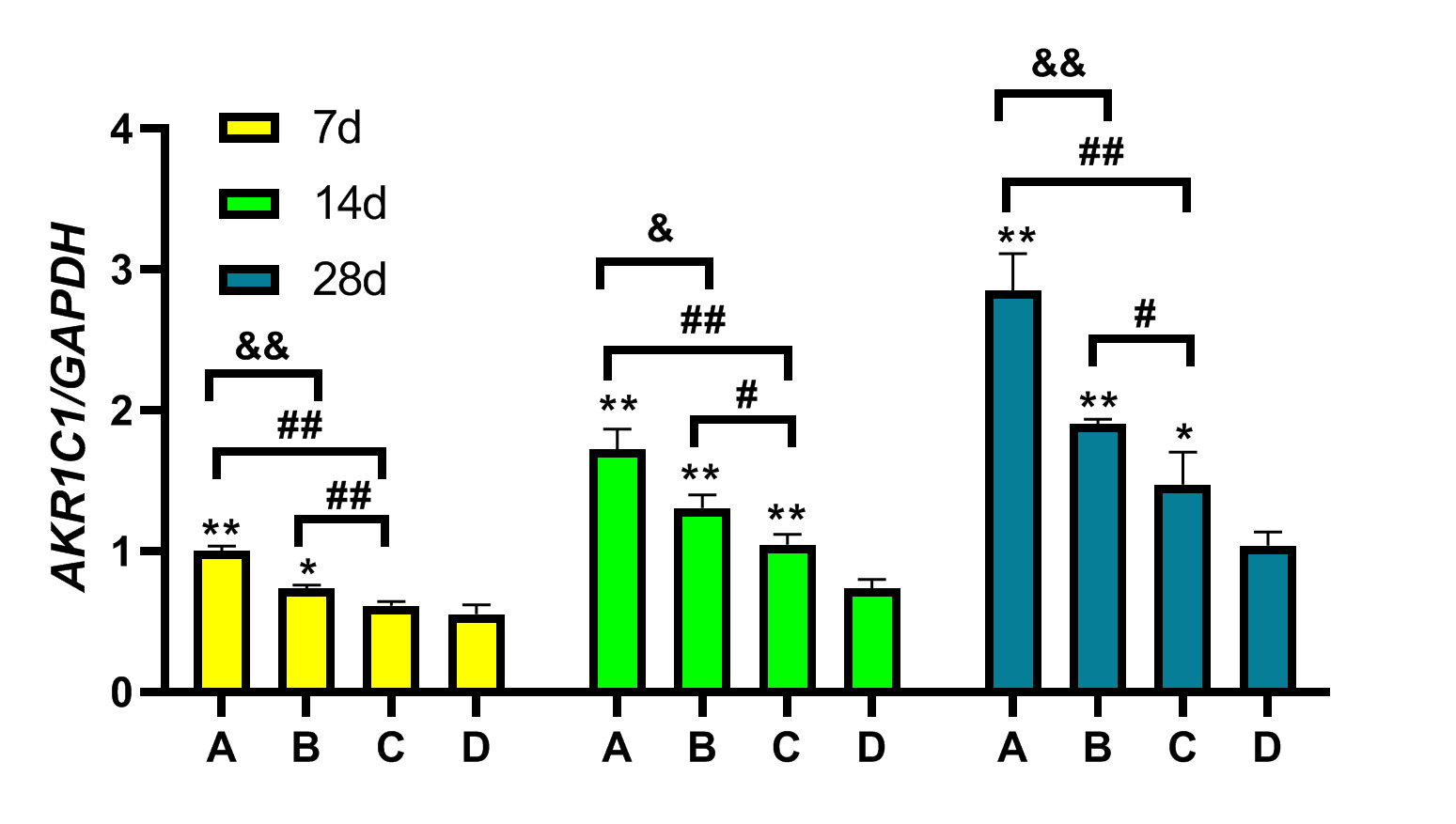

Supplement: S7 File — (ZIP) [file pone.0330078.s007.zip › QPCR/Figure/AKR1C1.bmp]

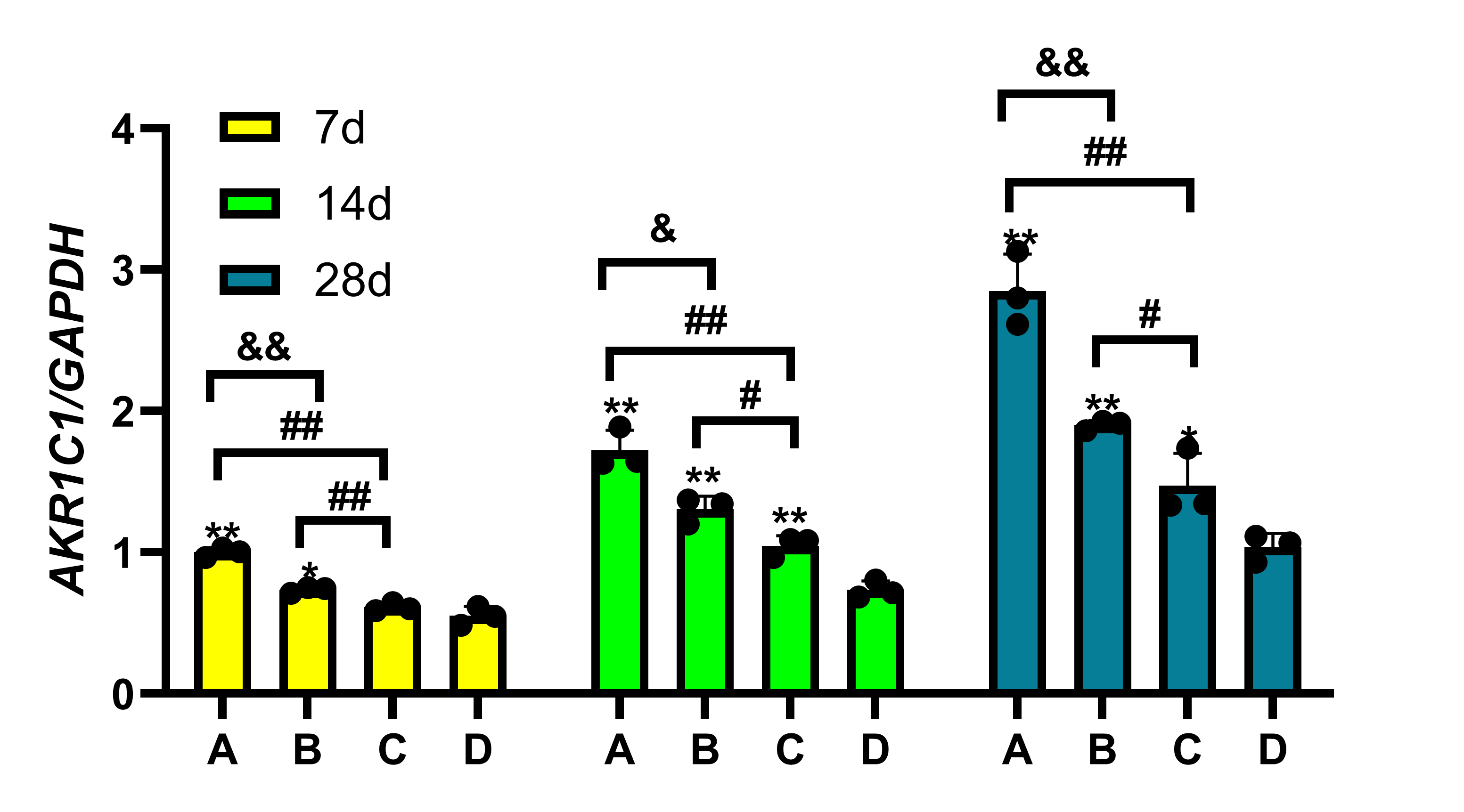

Supplement: S7 File — (ZIP) [file pone.0330078.s007.zip › QPCR/Figure/Copy of AKR1C1.tif]

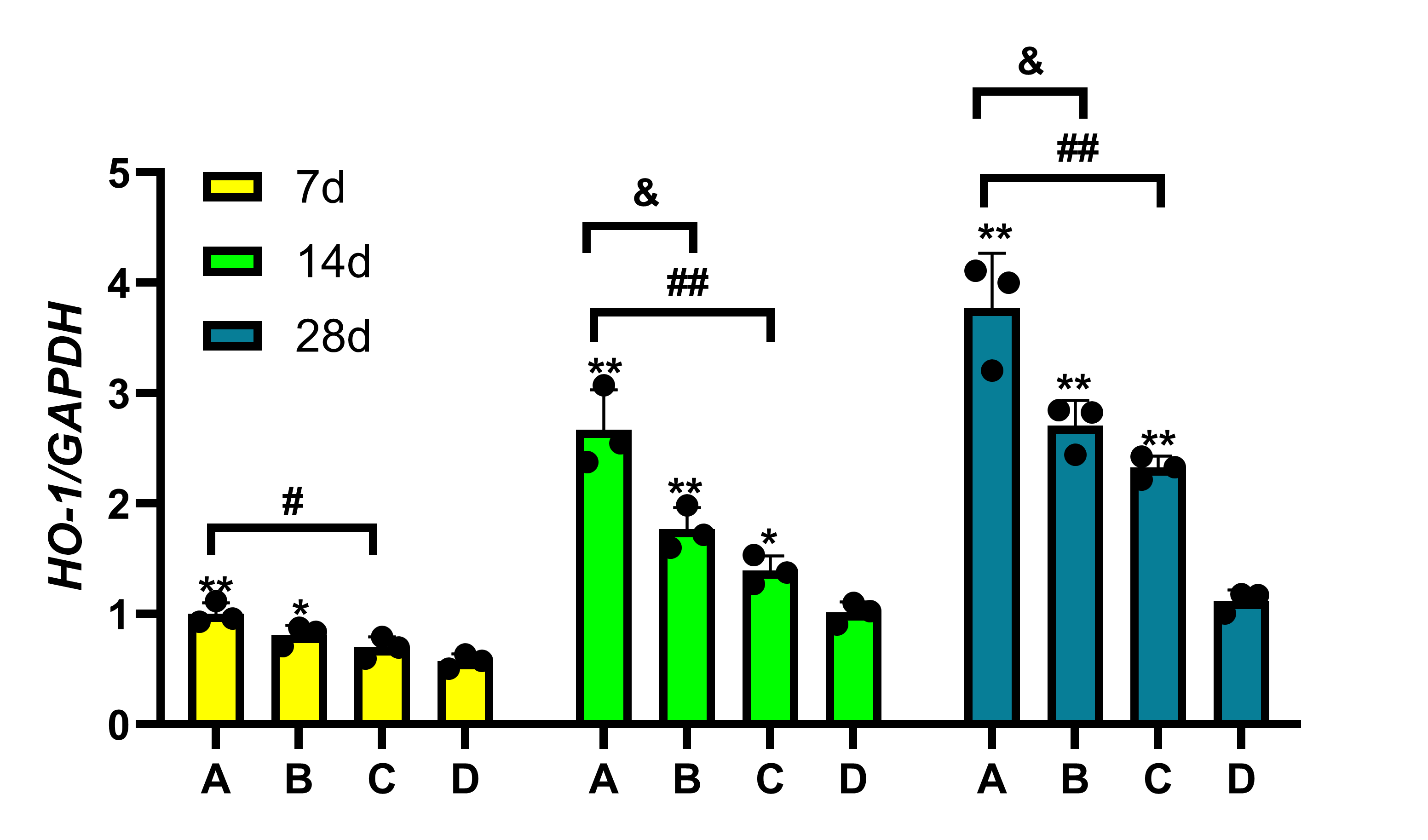

Supplement: S7 File — (ZIP) [file pone.0330078.s007.zip › QPCR/Figure/Copy of HO-1.tif]

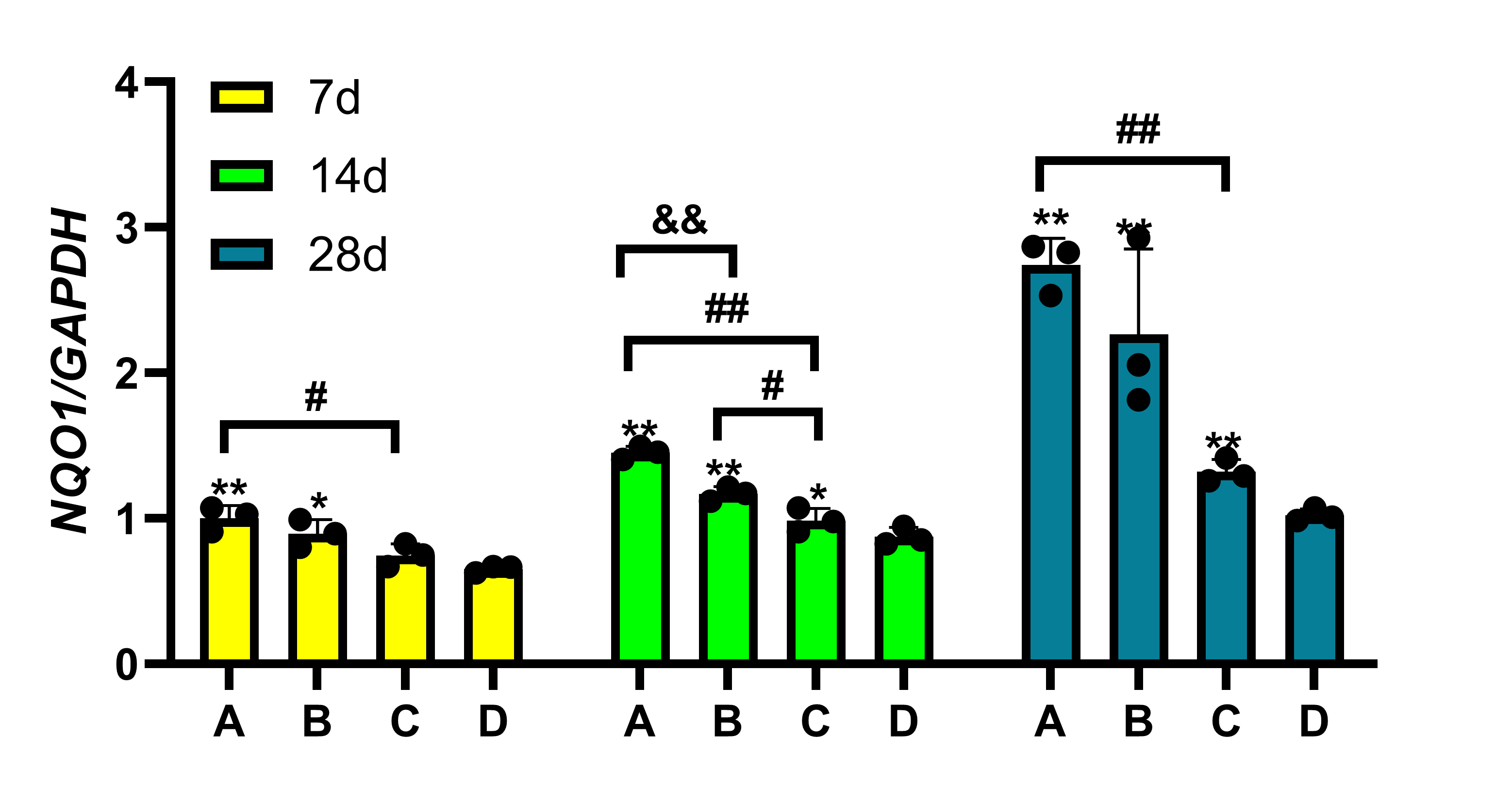

Supplement: S7 File — (ZIP) [file pone.0330078.s007.zip › QPCR/Figure/Copy of NQO1.tif]

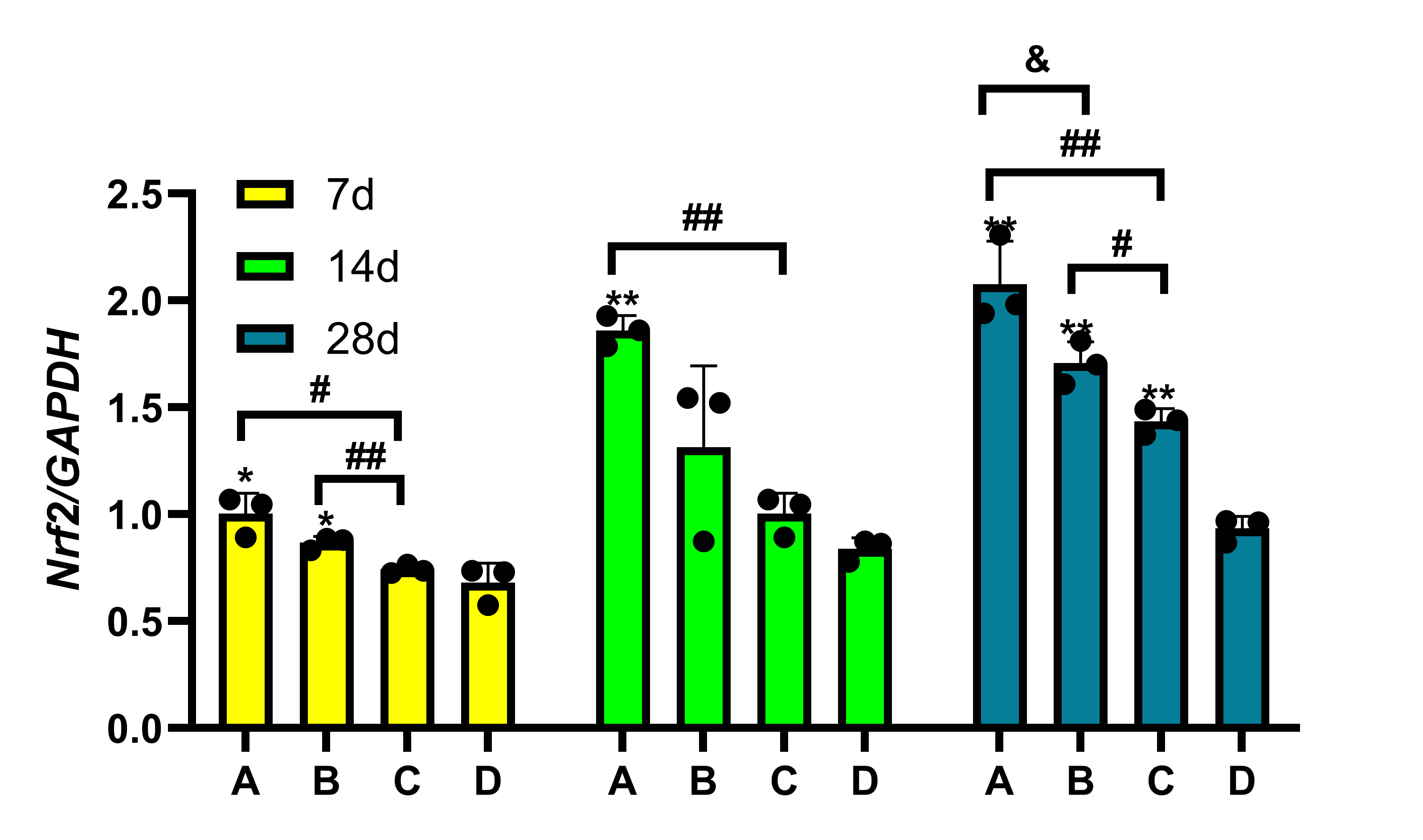

Supplement: S7 File — (ZIP) [file pone.0330078.s007.zip › QPCR/Figure/Copy of Nrf2.tif]

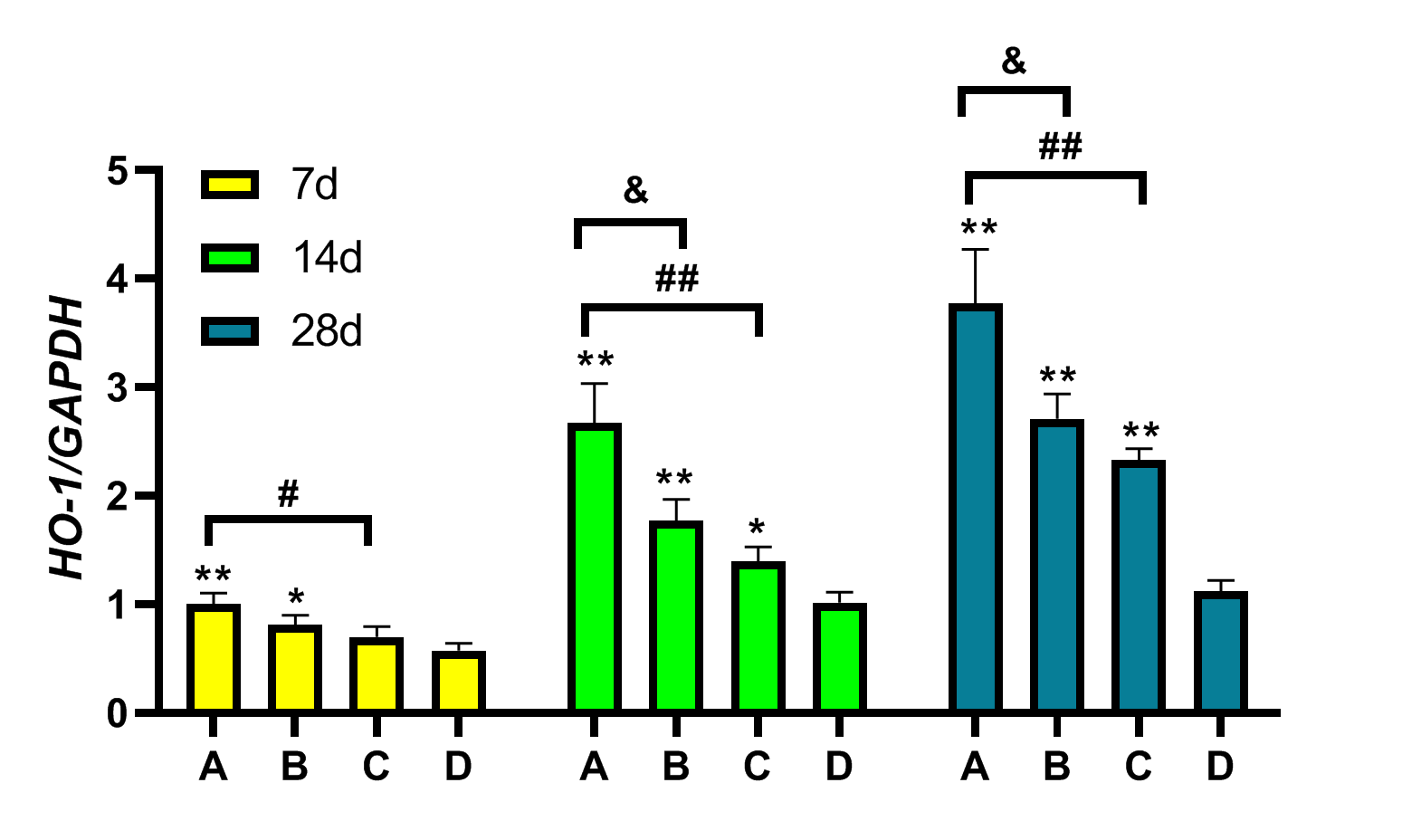

Supplement: S7 File — (ZIP) [file pone.0330078.s007.zip › QPCR/Figure/HO-1.bmp]

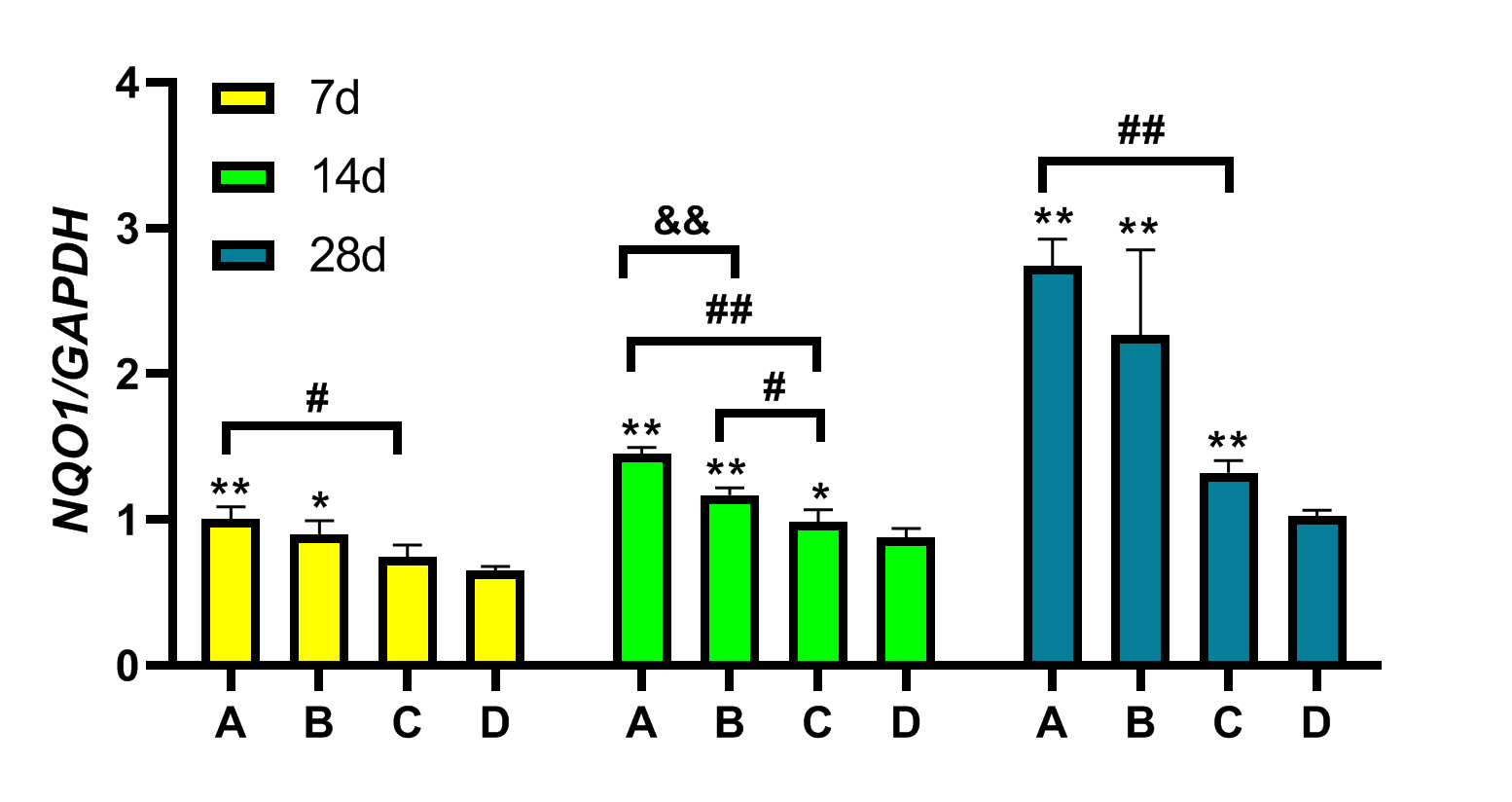

Supplement: S7 File — (ZIP) [file pone.0330078.s007.zip › QPCR/Figure/NQO1.bmp]

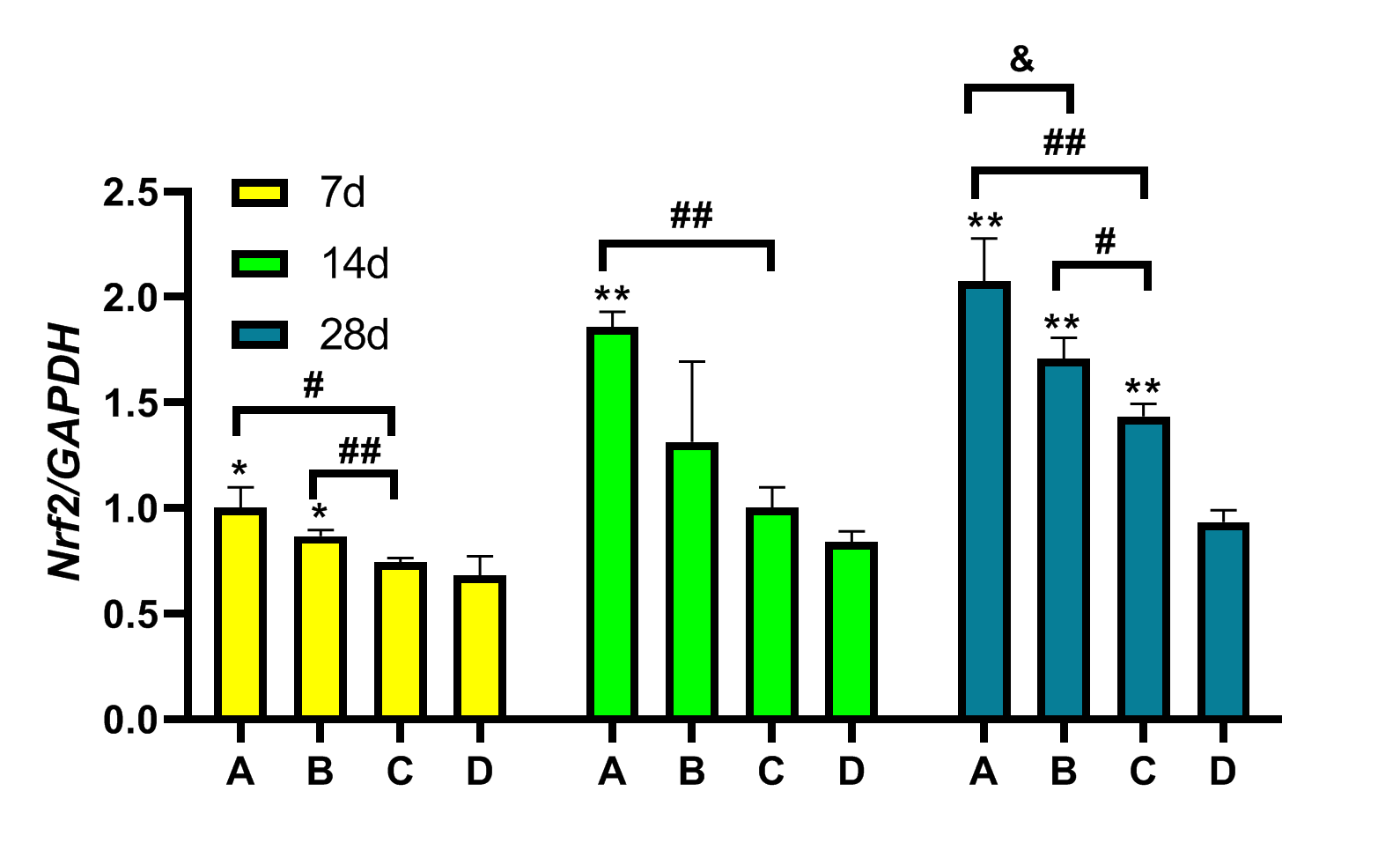

Supplement: S7 File — (ZIP) [file pone.0330078.s007.zip › QPCR/Figure/Nrf2.bmp]

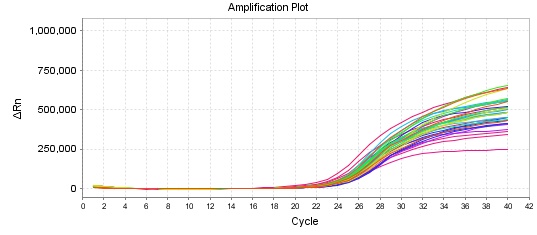

Supplement: S7 File — (ZIP) [file pone.0330078.s007.zip › QPCR/Result/Dissolution and amplification curve/Amplification Plot-AKR1C1.jpg]

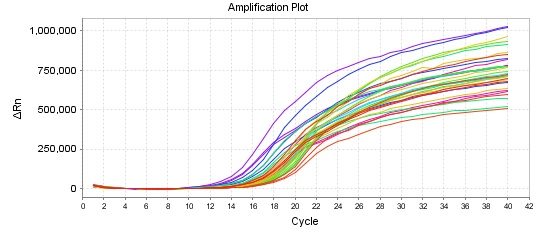

Supplement: S7 File — (ZIP) [file pone.0330078.s007.zip › QPCR/Result/Dissolution and amplification curve/Amplification Plot-GAPDH(AKR1C1).jpg]

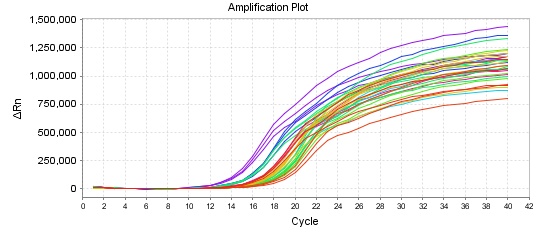

Supplement: S7 File — (ZIP) [file pone.0330078.s007.zip › QPCR/Result/Dissolution and amplification curve/Amplification Plot-GAPDH(HO-1).jpg]

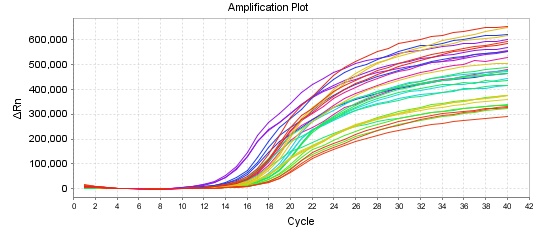

Supplement: S7 File — (ZIP) [file pone.0330078.s007.zip › QPCR/Result/Dissolution and amplification curve/Amplification Plot-GAPDH(NQO1).jpg]

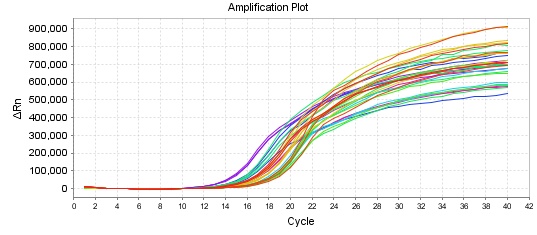

Supplement: S7 File — (ZIP) [file pone.0330078.s007.zip › QPCR/Result/Dissolution and amplification curve/Amplification Plot-GAPDH(Nrf2).jpg]

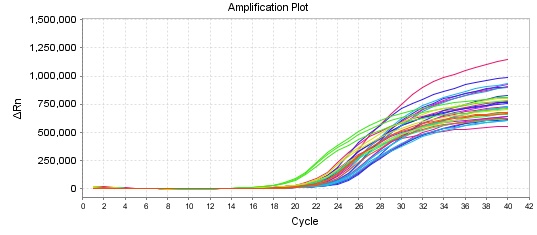

Supplement: S7 File — (ZIP) [file pone.0330078.s007.zip › QPCR/Result/Dissolution and amplification curve/Amplification Plot-HO-1.jpg]

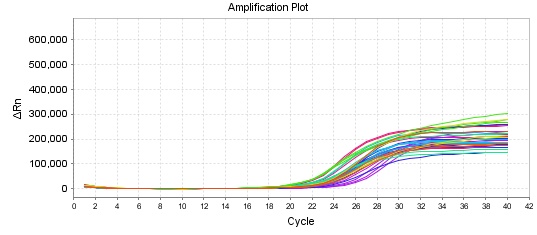

Supplement: S7 File — (ZIP) [file pone.0330078.s007.zip › QPCR/Result/Dissolution and amplification curve/Amplification Plot-NQO1.jpg]

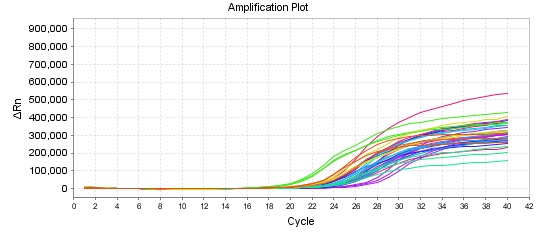

Supplement: S7 File — (ZIP) [file pone.0330078.s007.zip › QPCR/Result/Dissolution and amplification curve/Amplification Plot-Nrf2.jpg]

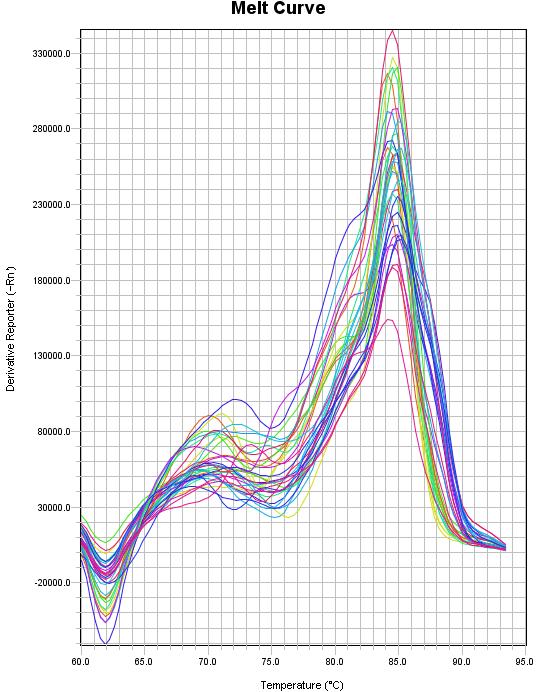

Supplement: S7 File — (ZIP) [file pone.0330078.s007.zip › QPCR/Result/Dissolution and amplification curve/Melt Curve-AKR1C1.jpg]

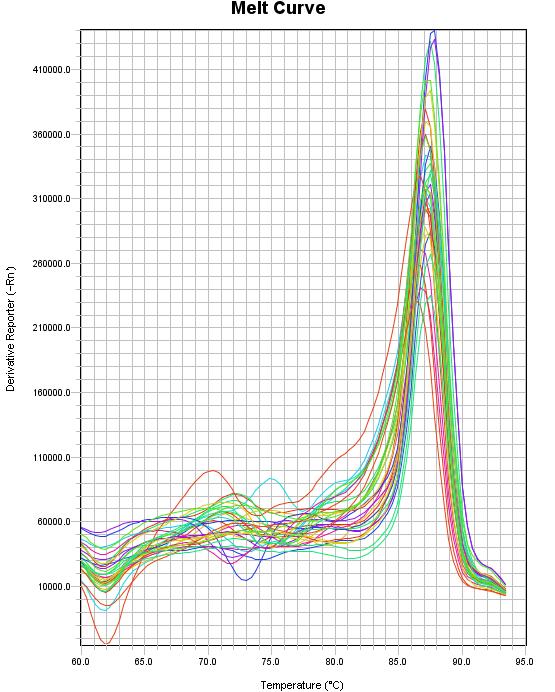

Supplement: S7 File — (ZIP) [file pone.0330078.s007.zip › QPCR/Result/Dissolution and amplification curve/Melt Curve-GAPDH(AKR1C1).jpg]

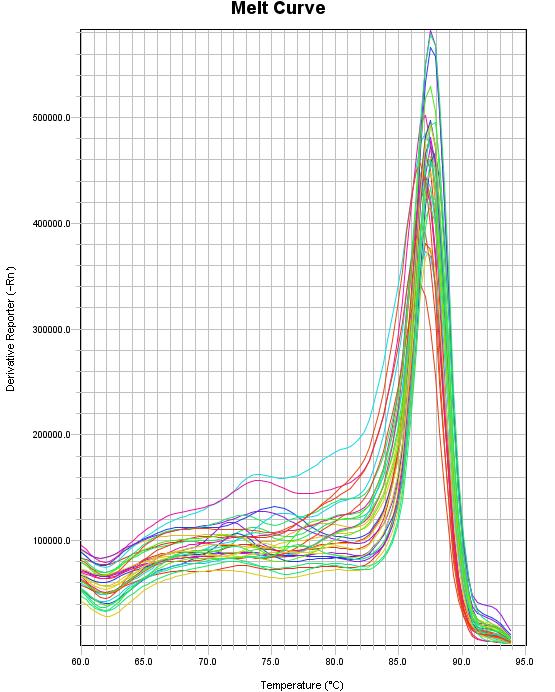

Supplement: S7 File — (ZIP) [file pone.0330078.s007.zip › QPCR/Result/Dissolution and amplification curve/Melt Curve-GAPDH(HO-1).jpg]

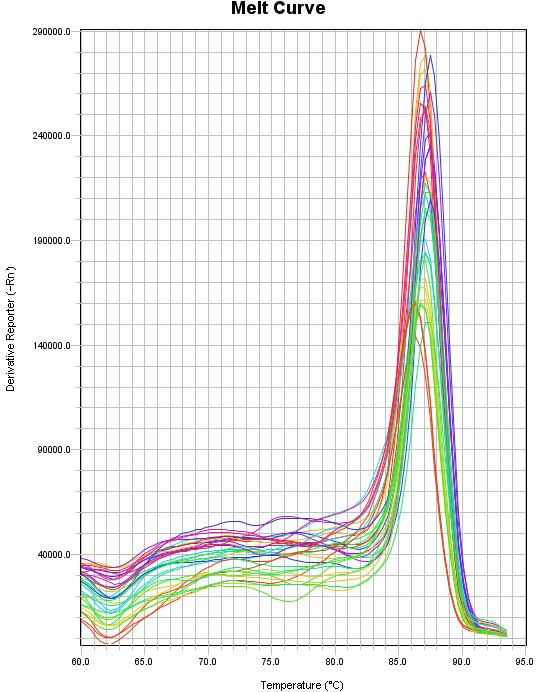

Supplement: S7 File — (ZIP) [file pone.0330078.s007.zip › QPCR/Result/Dissolution and amplification curve/Melt Curve-GAPDH(NQO1).jpg]

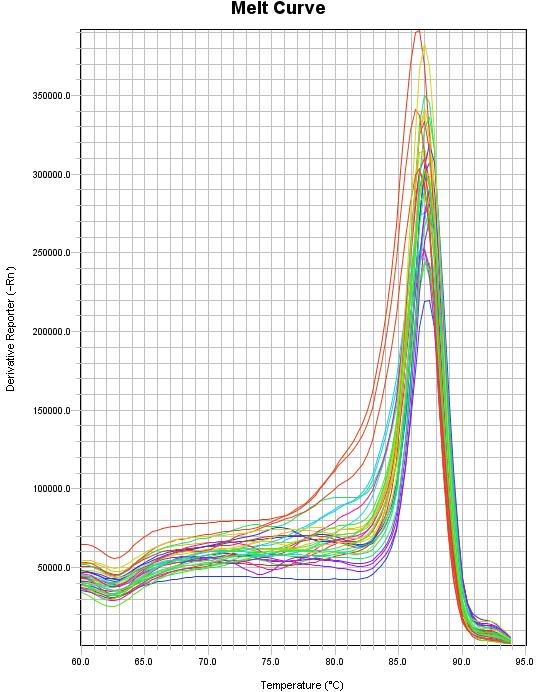

Supplement: S7 File — (ZIP) [file pone.0330078.s007.zip › QPCR/Result/Dissolution and amplification curve/Melt Curve-GAPDH(Nrf2).jpg]

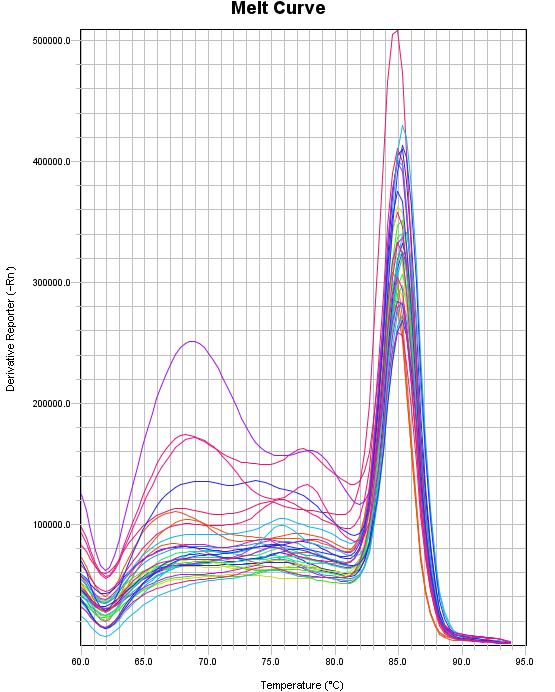

Supplement: S7 File — (ZIP) [file pone.0330078.s007.zip › QPCR/Result/Dissolution and amplification curve/Melt Curve-HO-1.jpg]

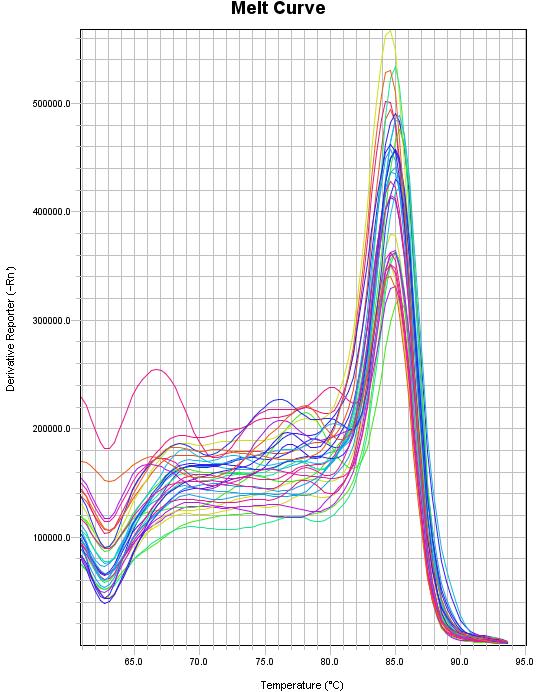

Supplement: S7 File — (ZIP) [file pone.0330078.s007.zip › QPCR/Result/Dissolution and amplification curve/Melt Curve-NQO1.jpg]

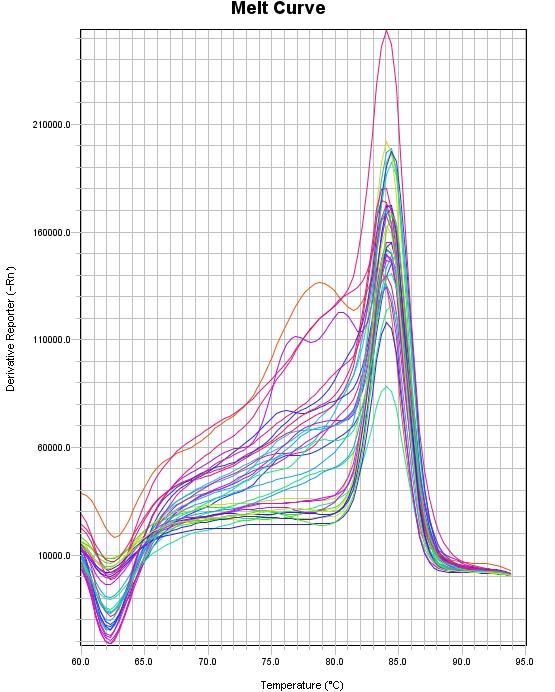

Supplement: S7 File — (ZIP) [file pone.0330078.s007.zip › QPCR/Result/Dissolution and amplification curve/Melt Curve-Nrf2.jpg]

**Nrf2**

CGF+HAMCC

HAMCC

CGF

Control

250kD

150kD

100kD

70kD

50kD

40kD

35kD

25kD

20kD

15kD

10kD

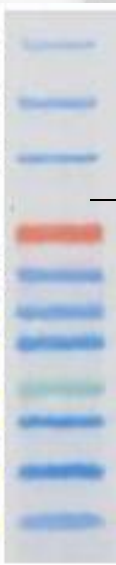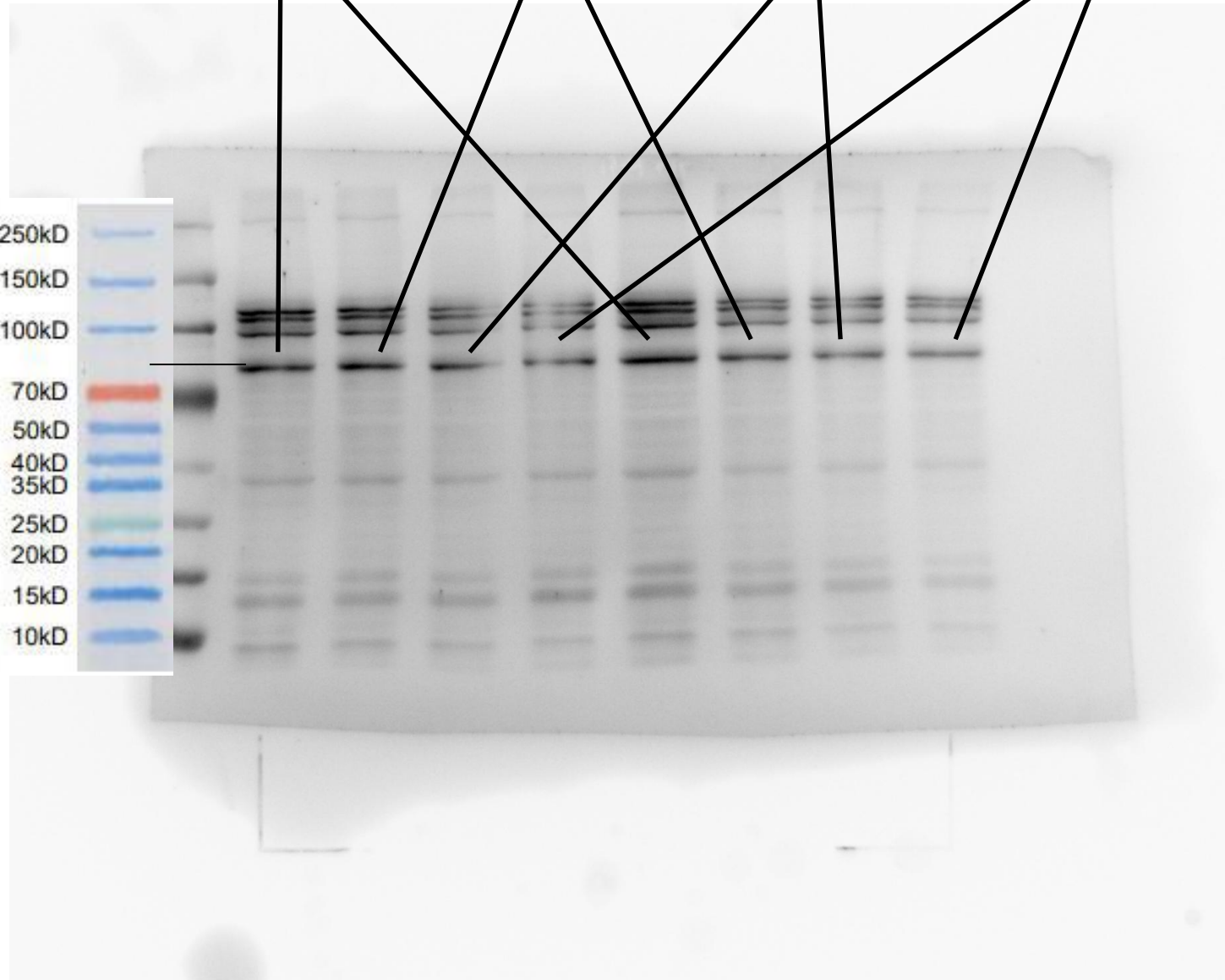

## Nrf2

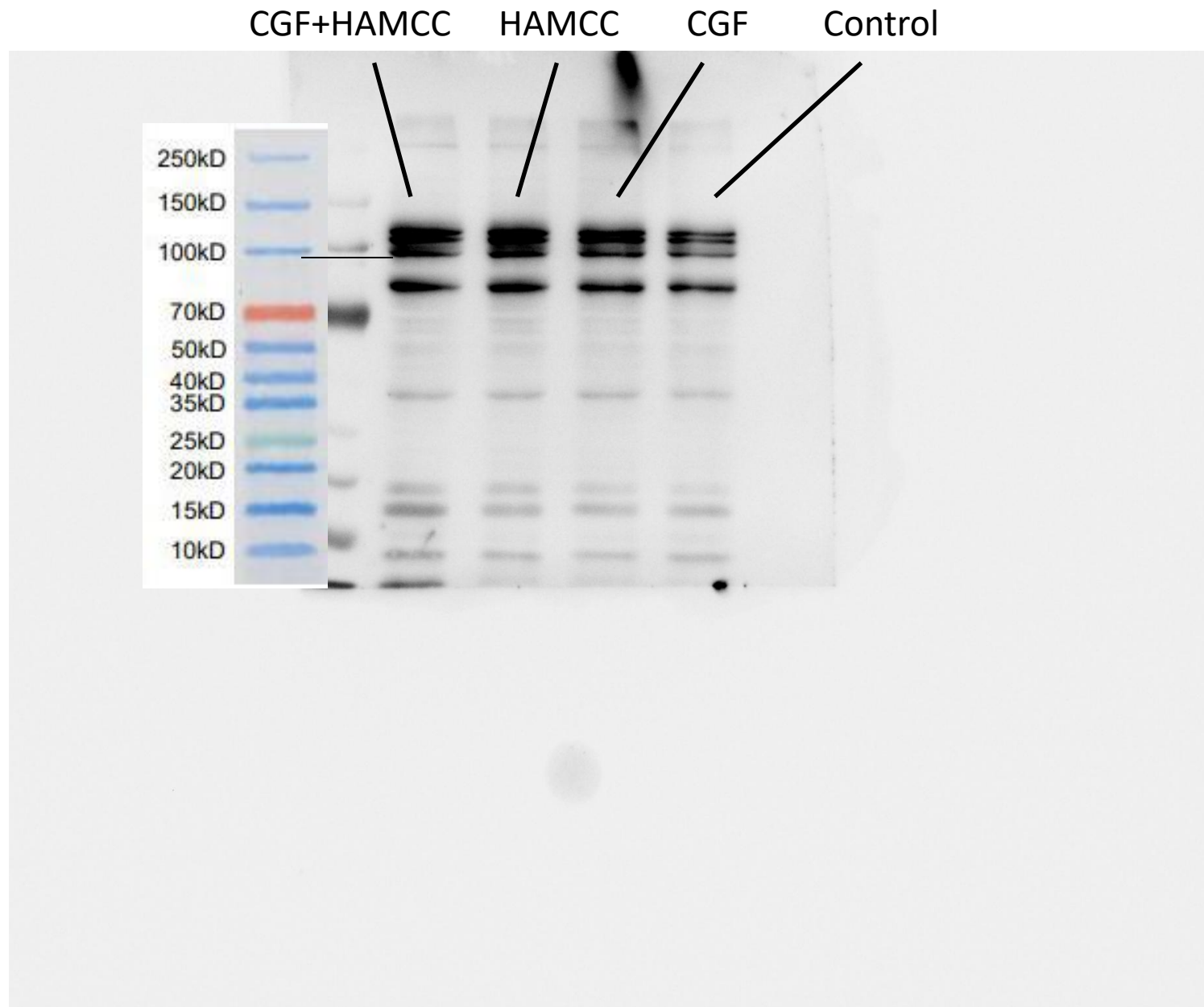

# HO-1

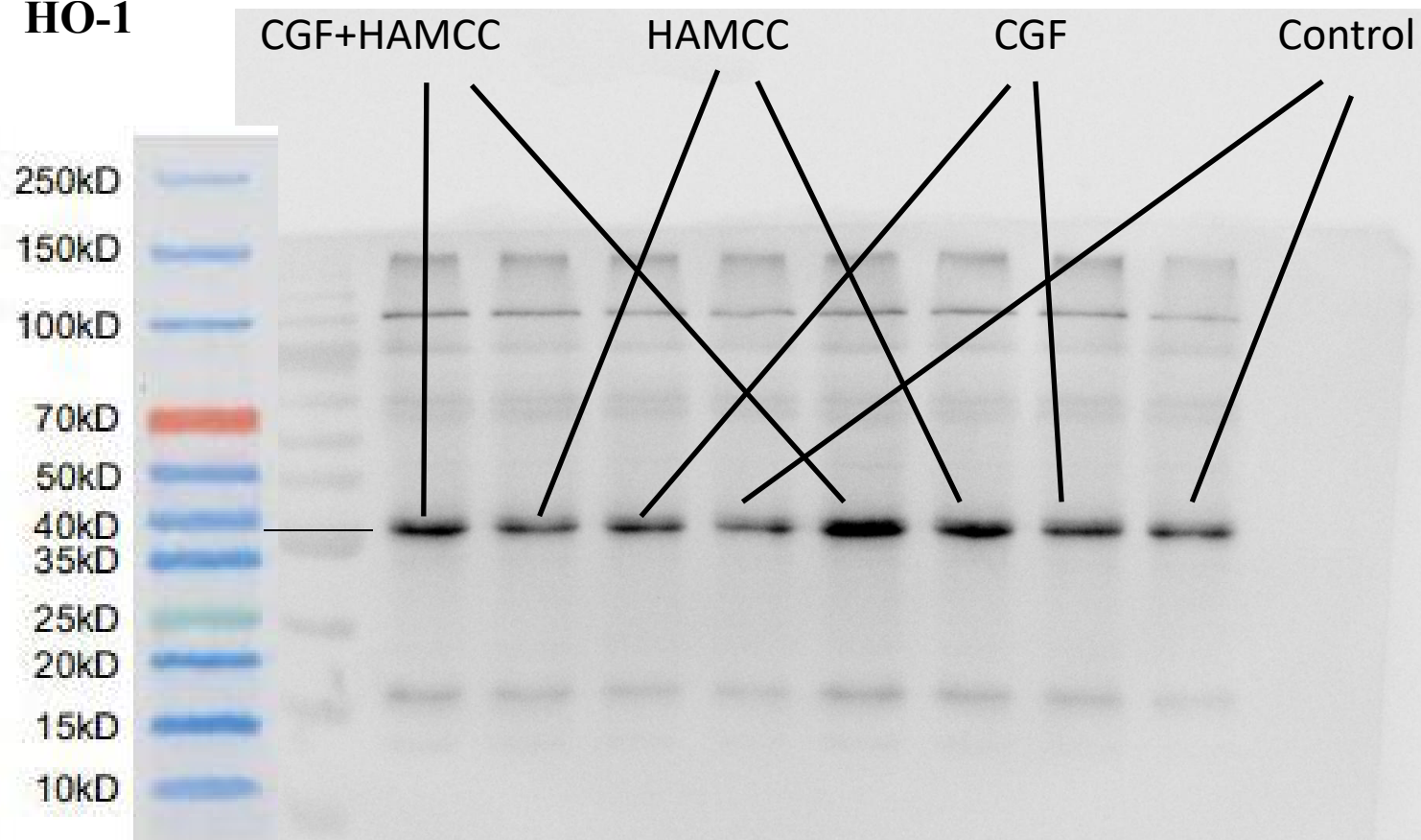

# HO-1

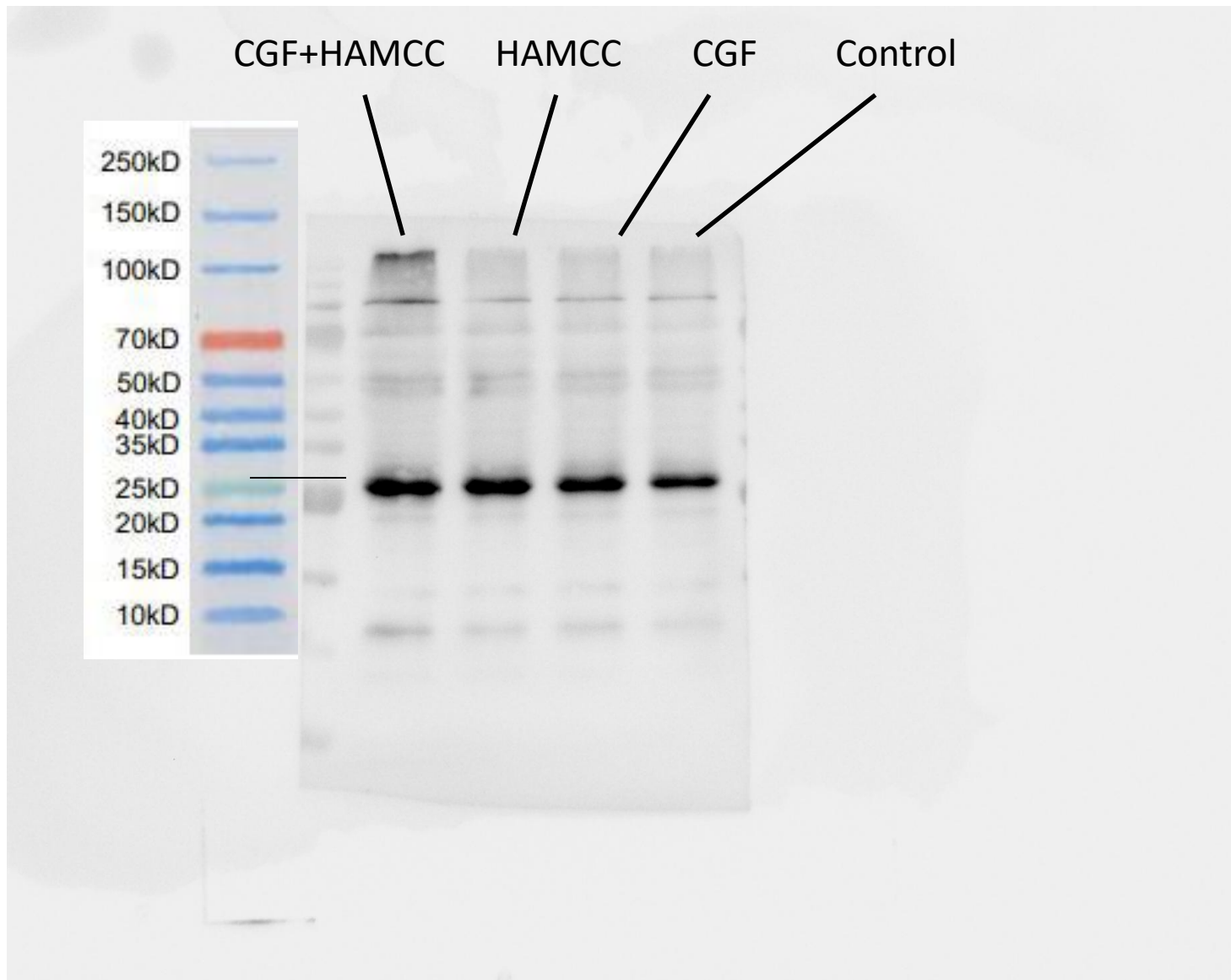

# NQO1

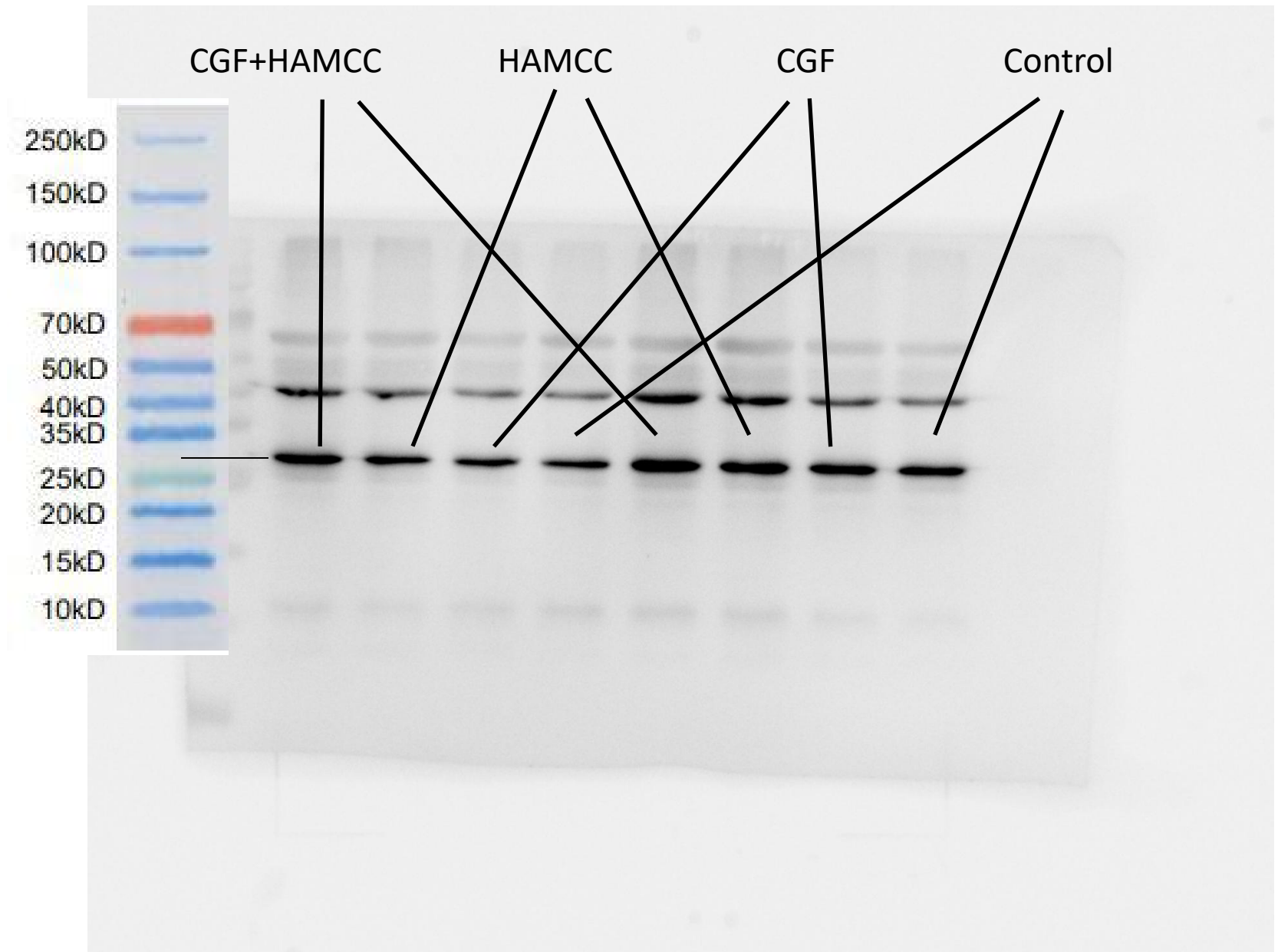

# NQO1

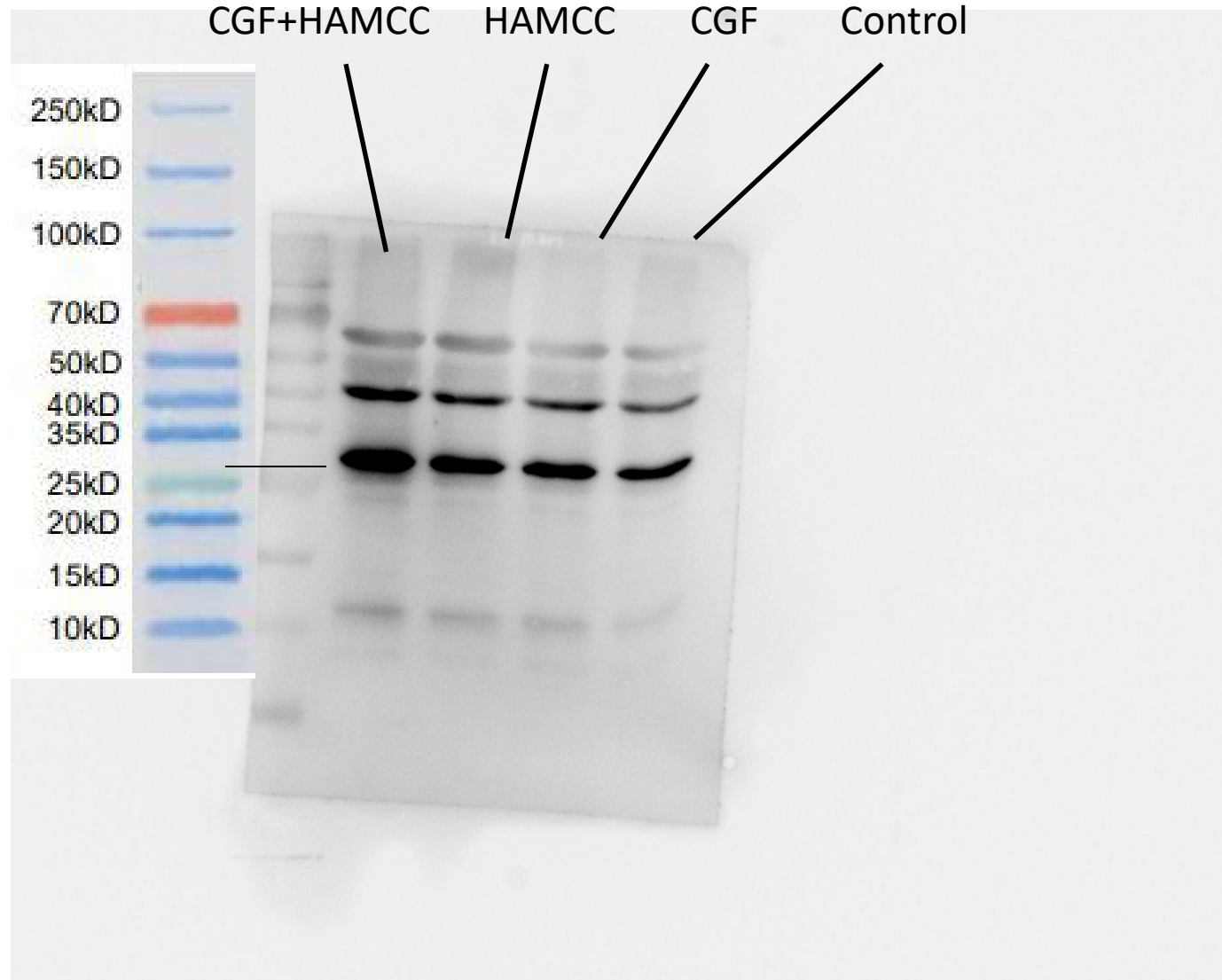

# AKR1C1

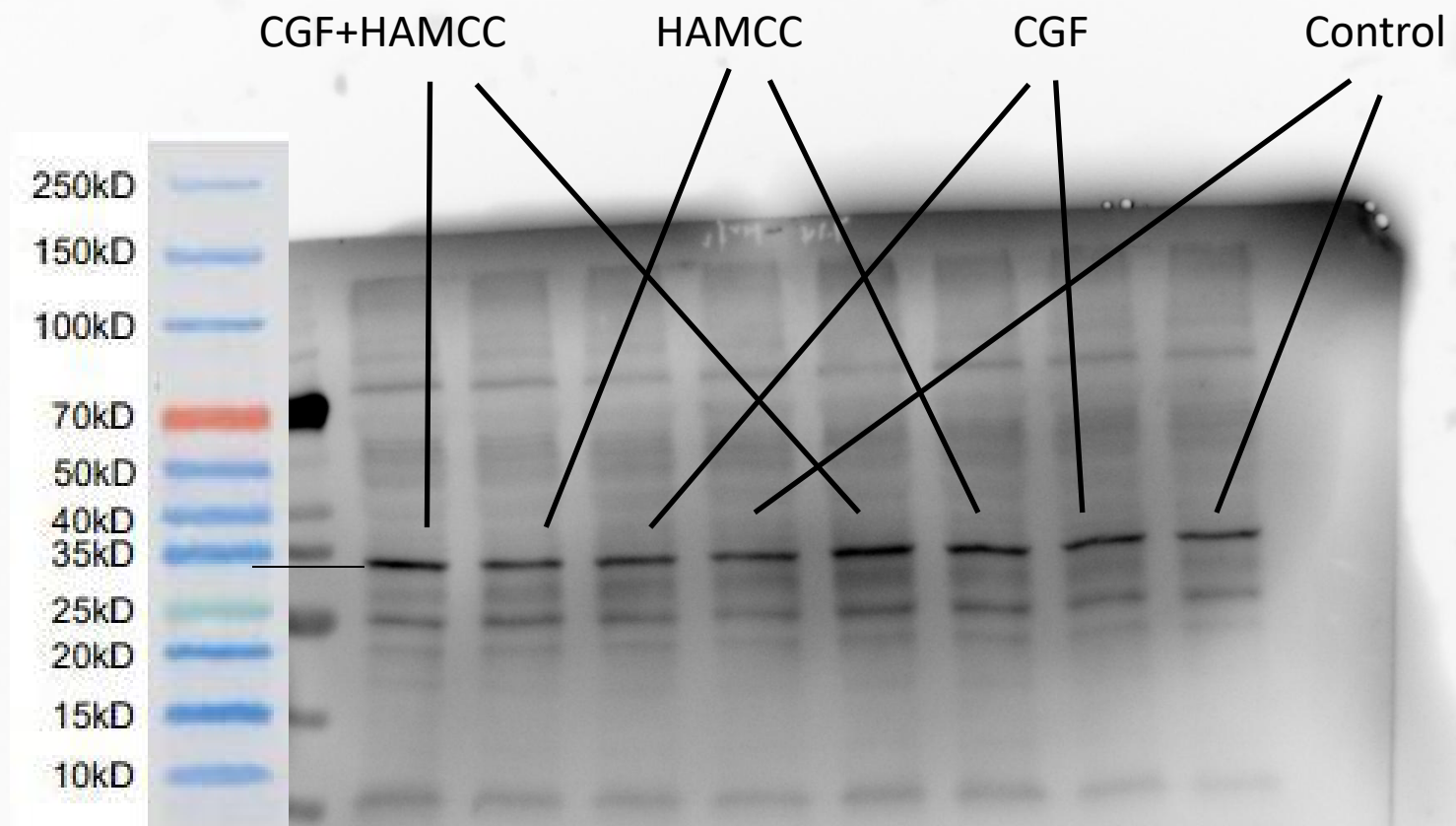

# AKR1C1

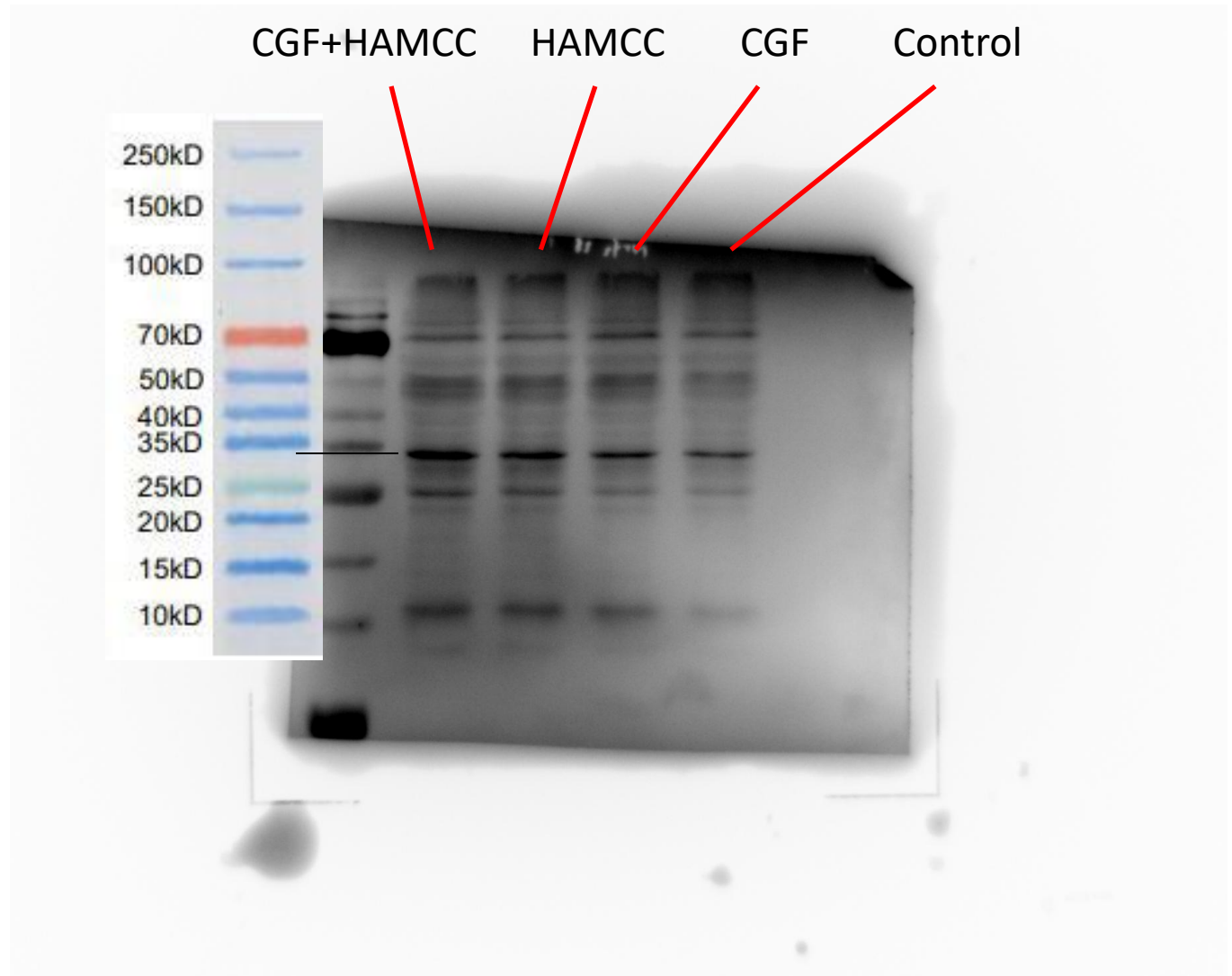

# GAPDH

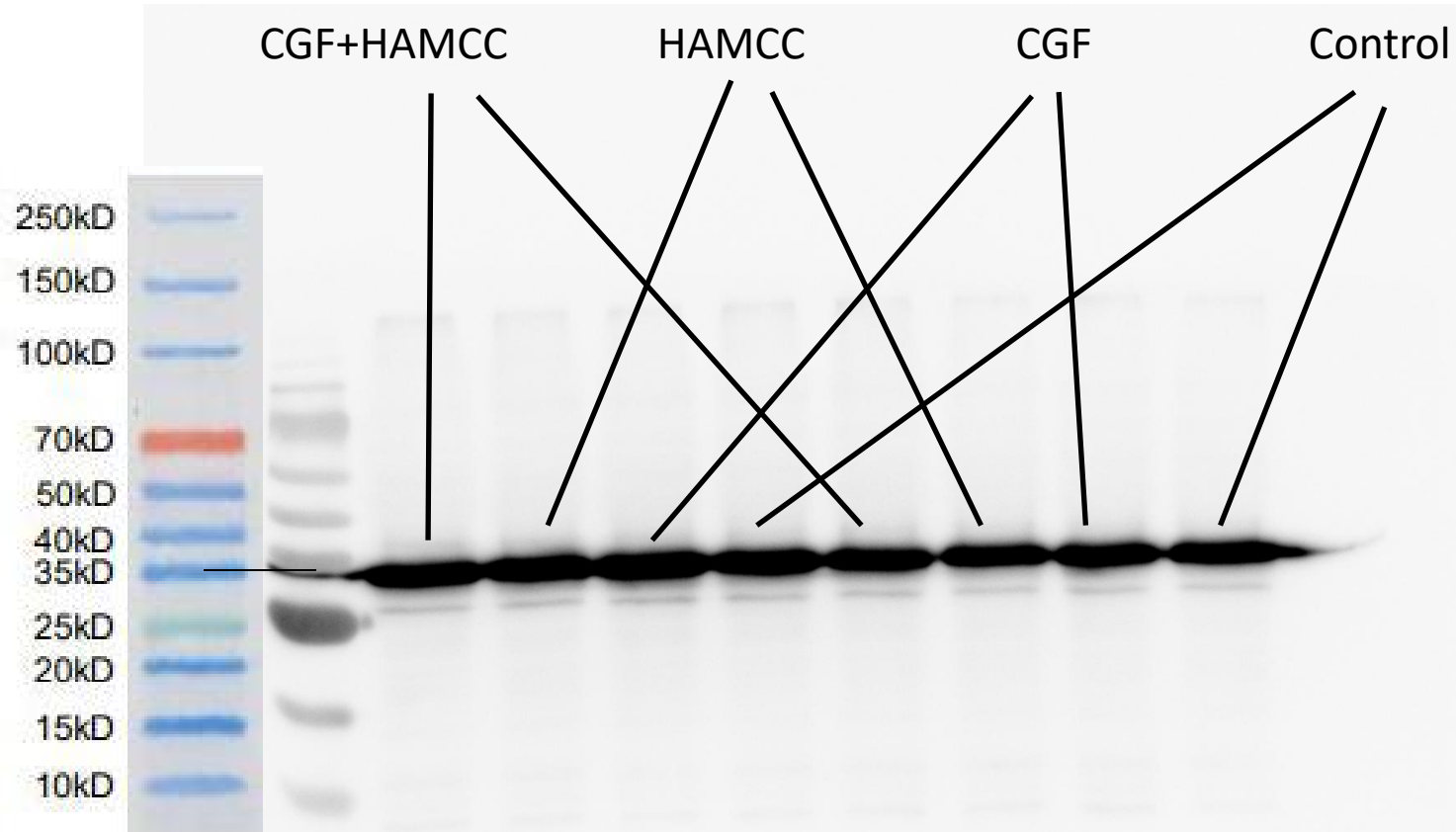

# GAPDH

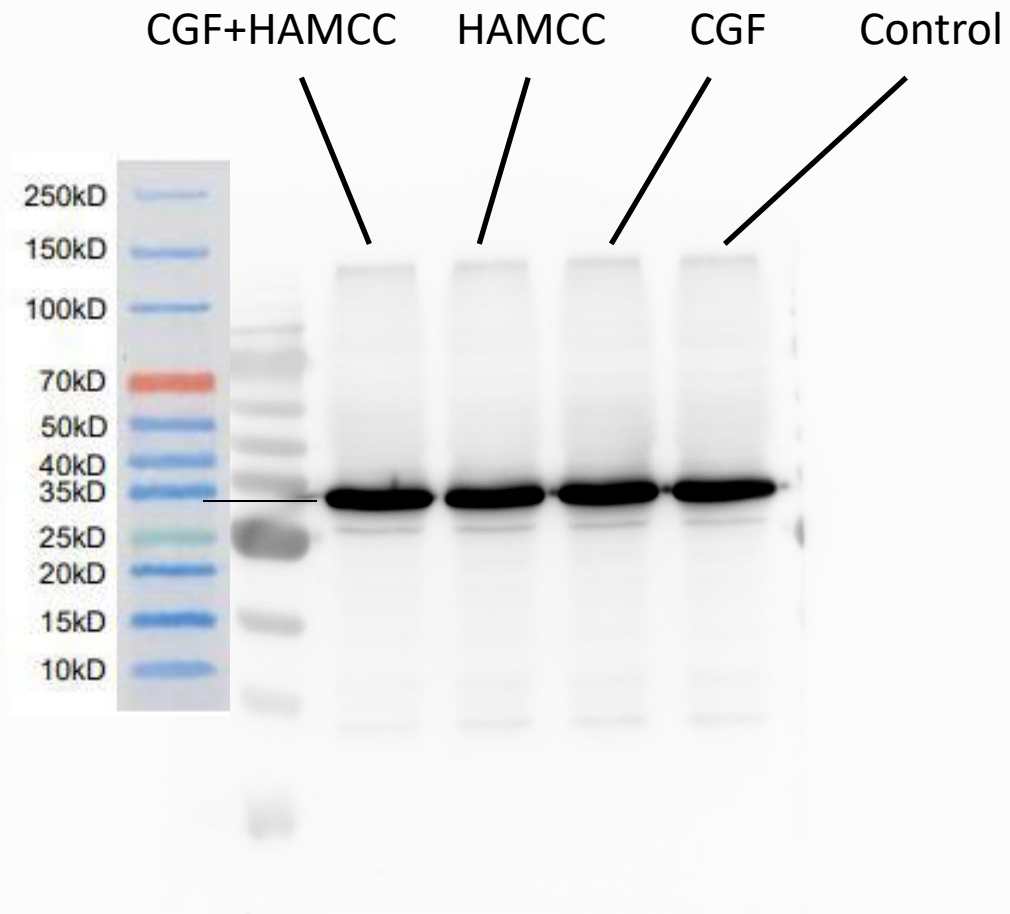

Supplement: S8 File — (ZIP) [file pone.0330078.s008.zip › WB/S1_raw_images.pdf]

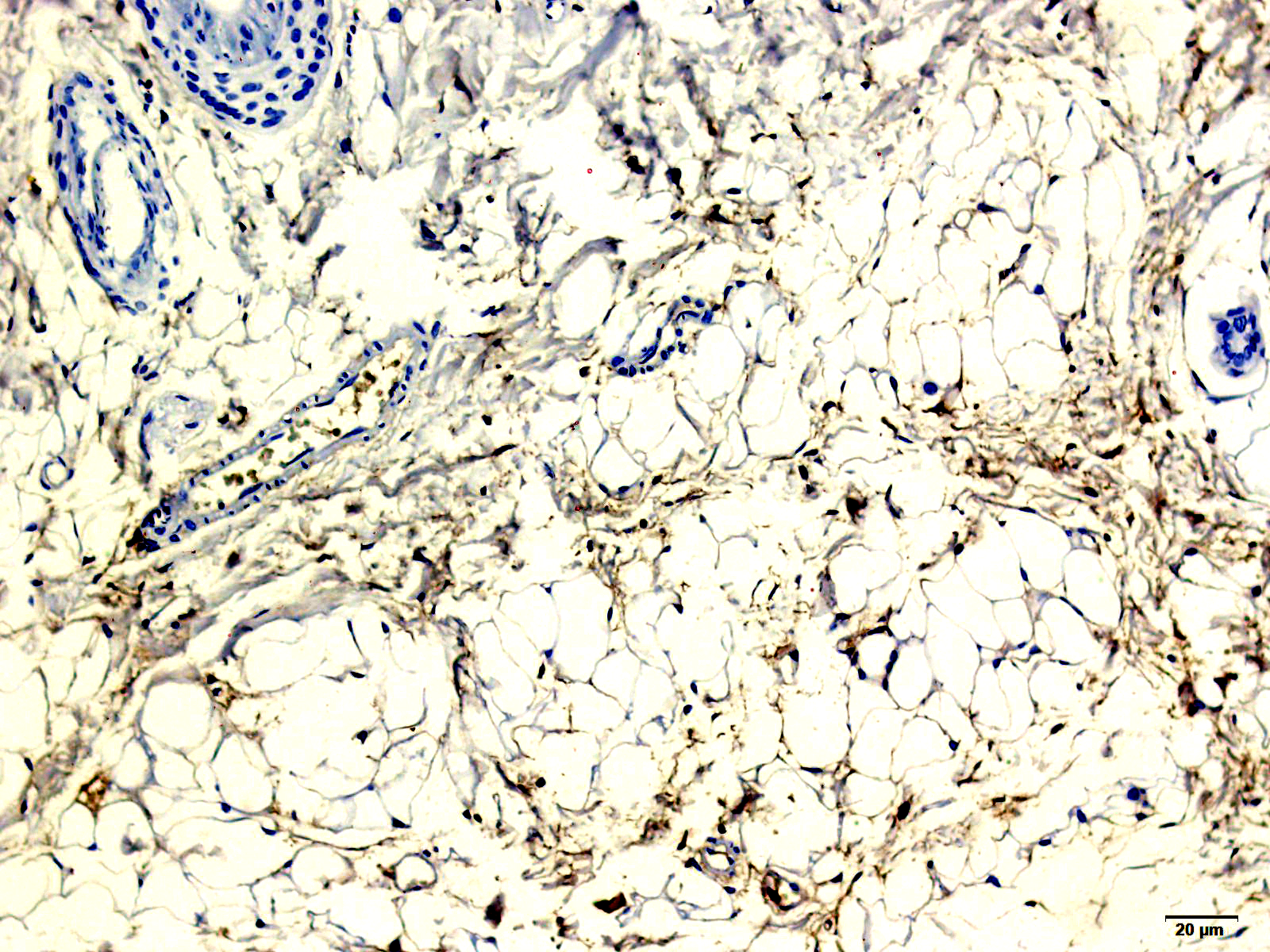

Supplement: S9 File — (ZIP) [file pone.0330078.s009.zip › immunohistochemistry/14D/CGF/14D CGF 1.tif]

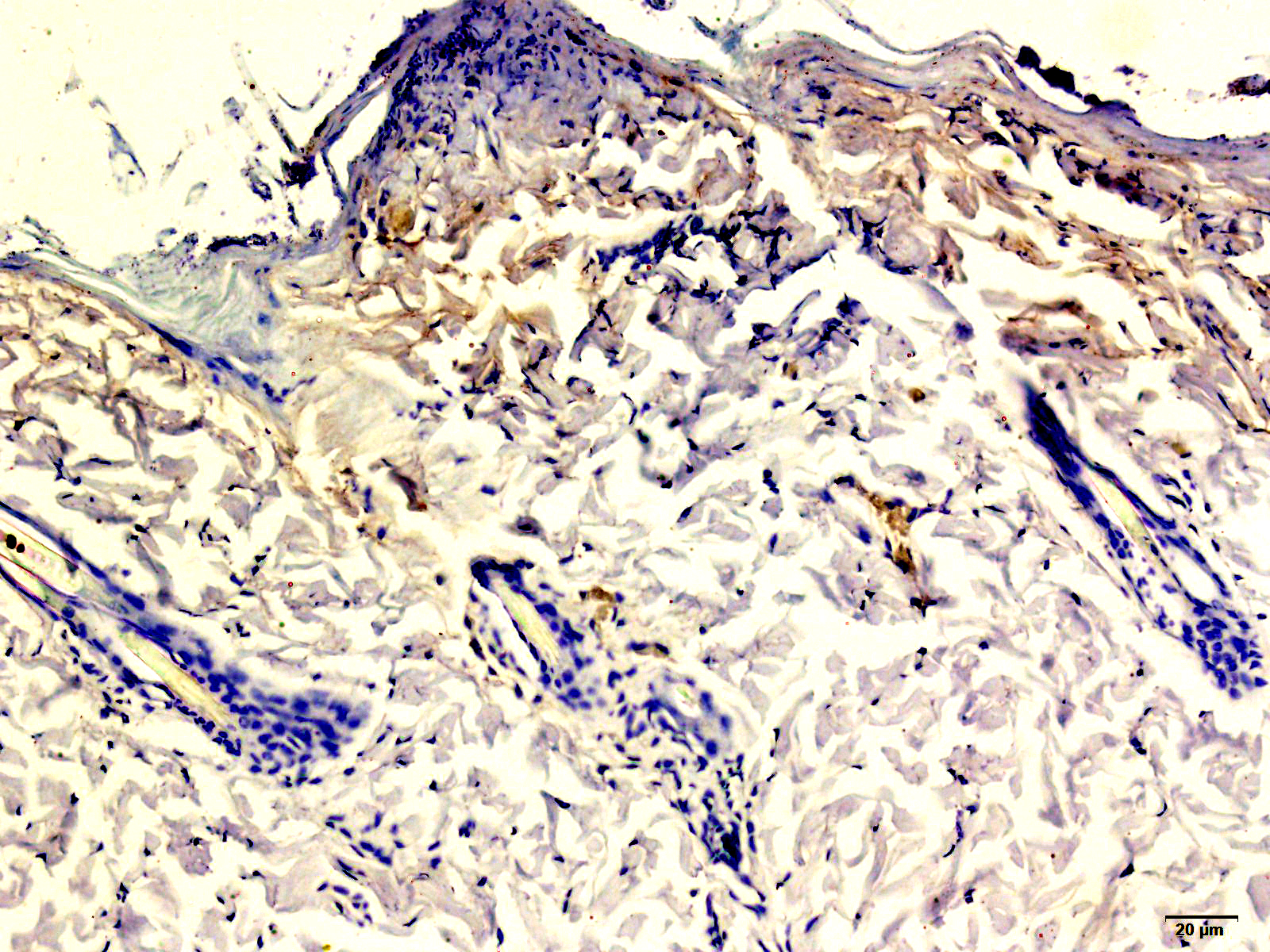

Supplement: S9 File — (ZIP) [file pone.0330078.s009.zip › immunohistochemistry/14D/CGF/14D CGF 2.tif]

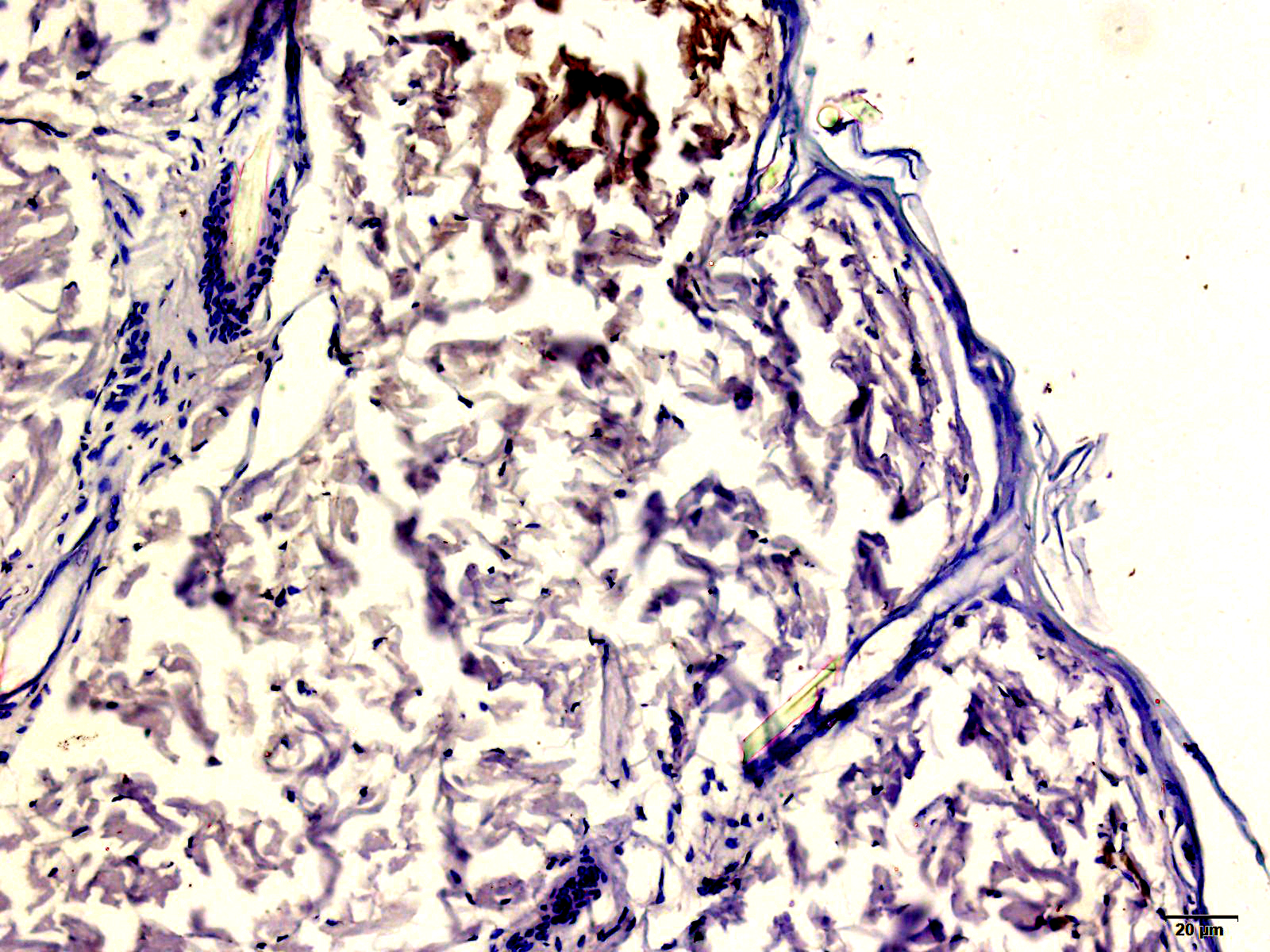

Supplement: S9 File — (ZIP) [file pone.0330078.s009.zip › immunohistochemistry/14D/CGF/14D CGF 3.tif]

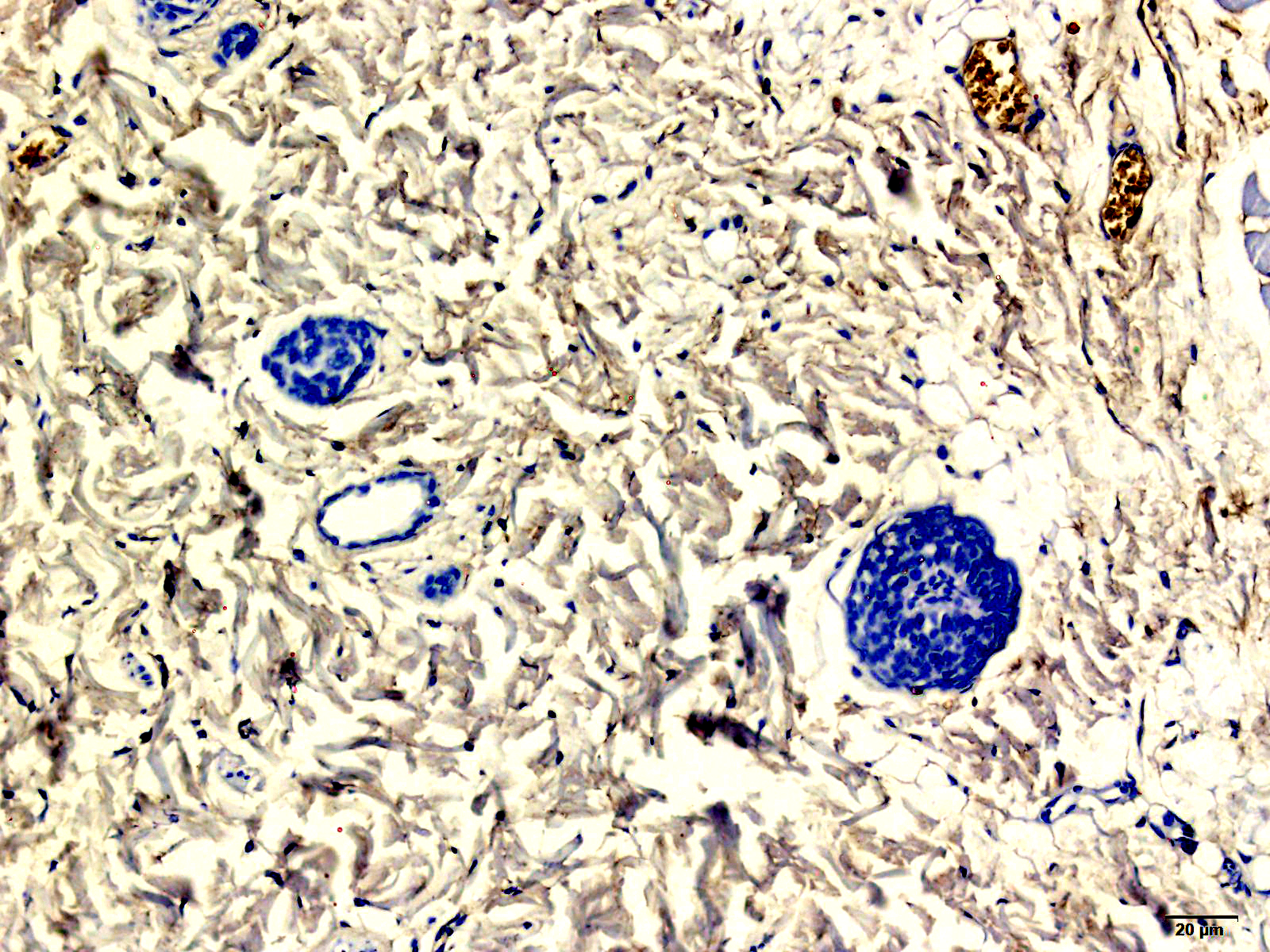

Supplement: S9 File — (ZIP) [file pone.0330078.s009.zip › immunohistochemistry/14D/CGF+HAMCC/14D CGF+HAMCC 1.tif]

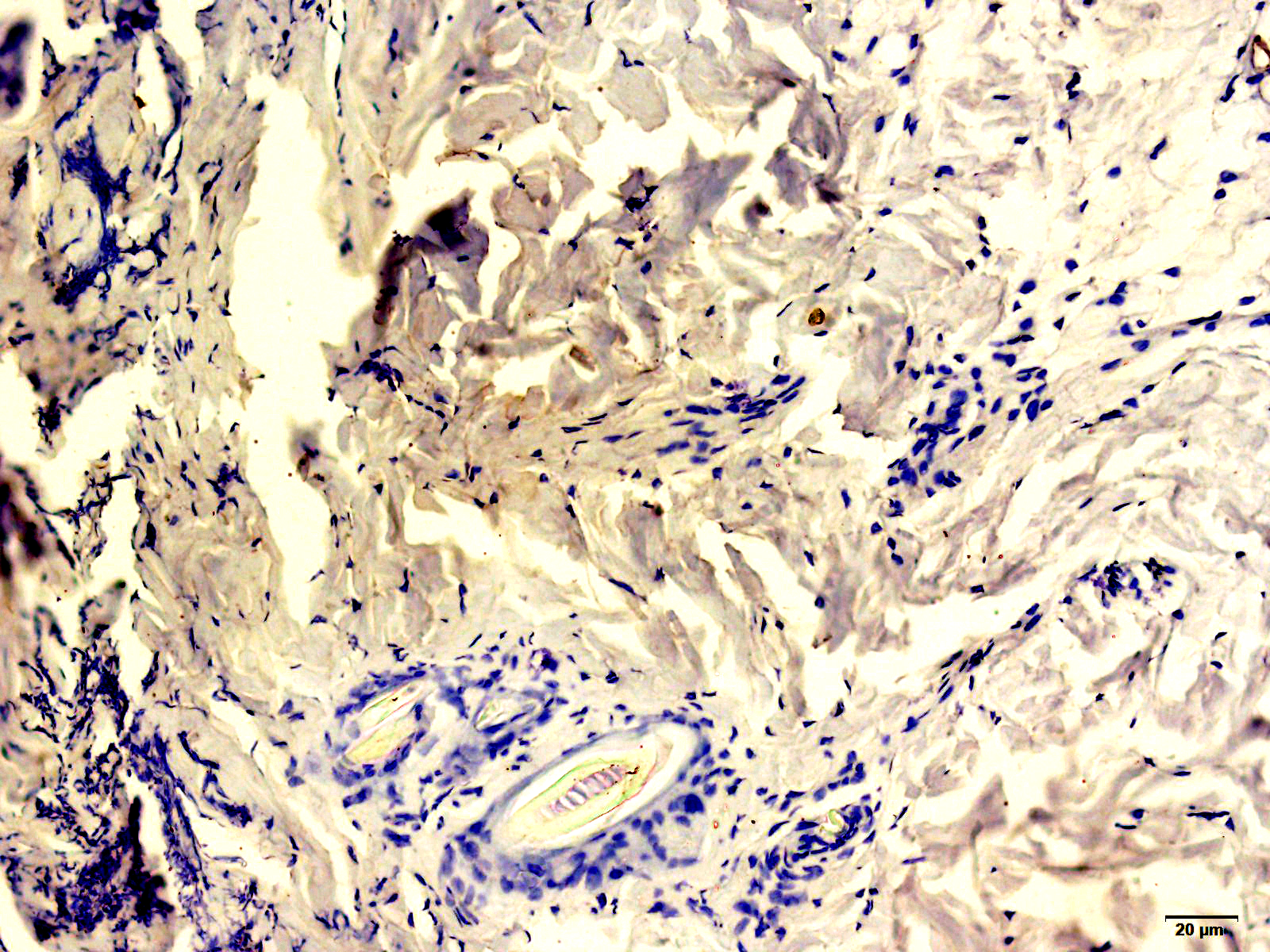

Supplement: S9 File — (ZIP) [file pone.0330078.s009.zip › immunohistochemistry/14D/CGF+HAMCC/14D CGF+HAMCC 2.tif]

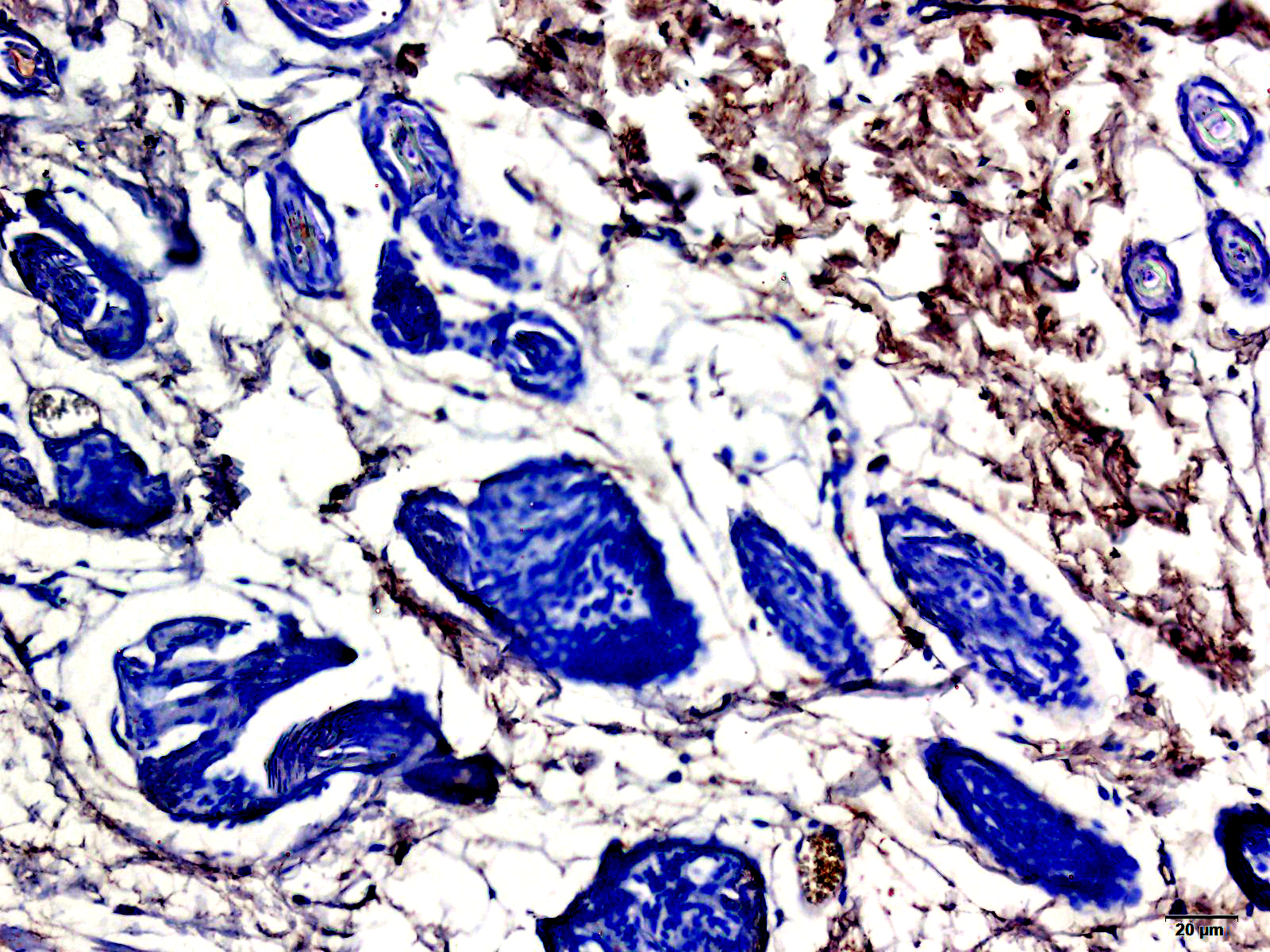

Supplement: S9 File — (ZIP) [file pone.0330078.s009.zip › immunohistochemistry/14D/CGF+HAMCC/14D CGF+HAMCC 3.tif]

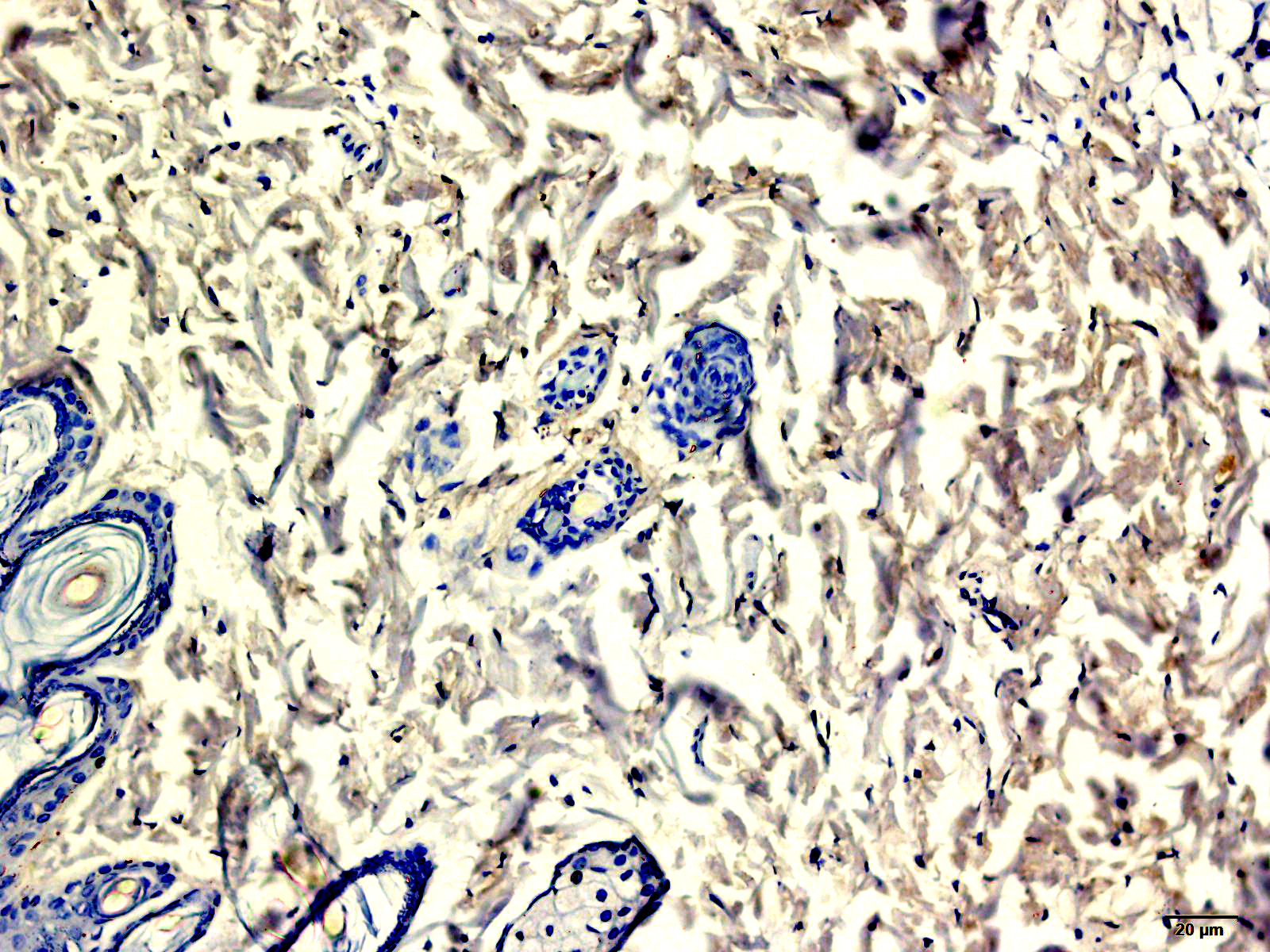

Supplement: S9 File — (ZIP) [file pone.0330078.s009.zip › immunohistochemistry/14D/Control/14D Control 1.tif]

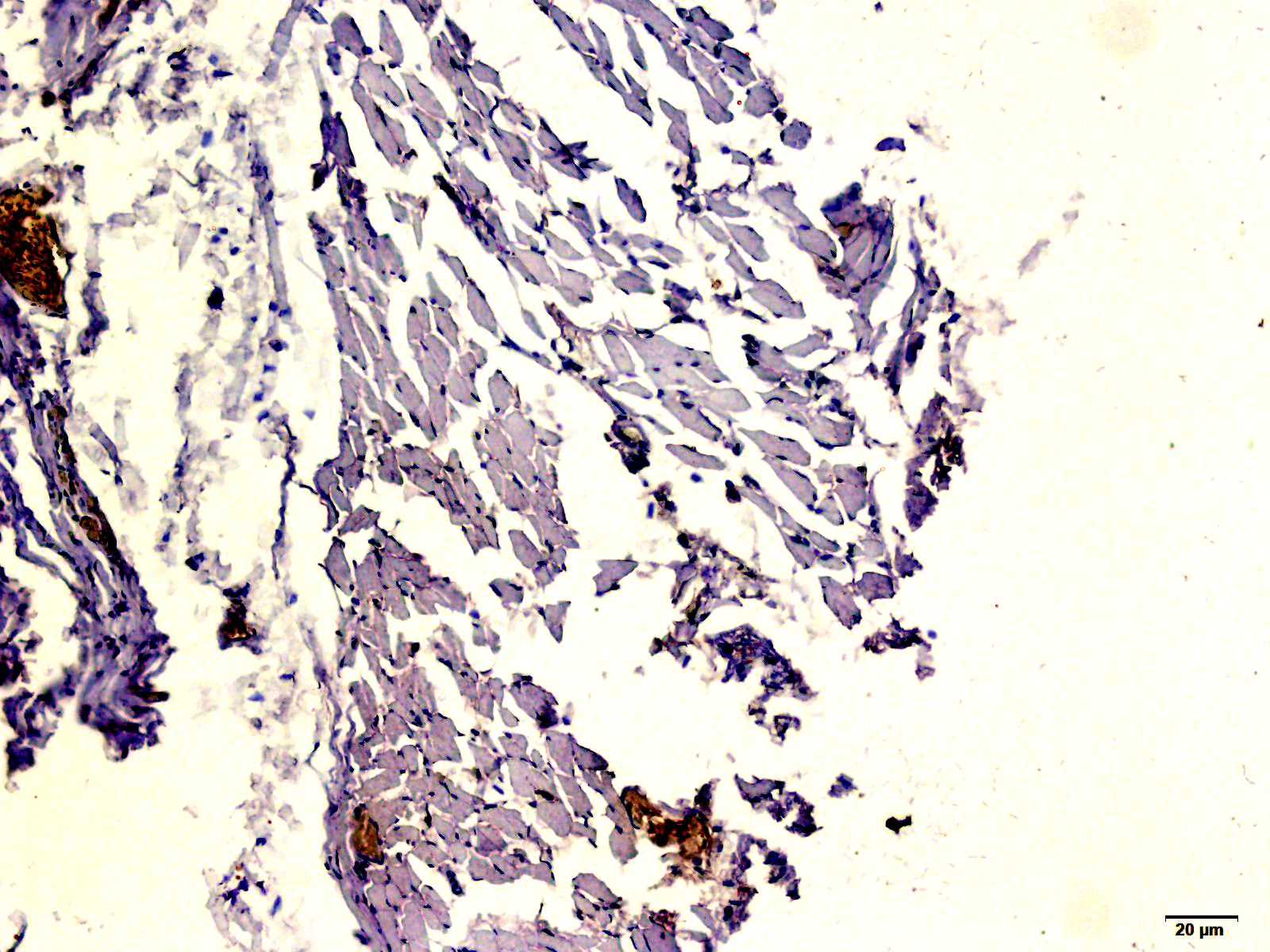

Supplement: S9 File — (ZIP) [file pone.0330078.s009.zip › immunohistochemistry/14D/Control/14D Control 2.tif]

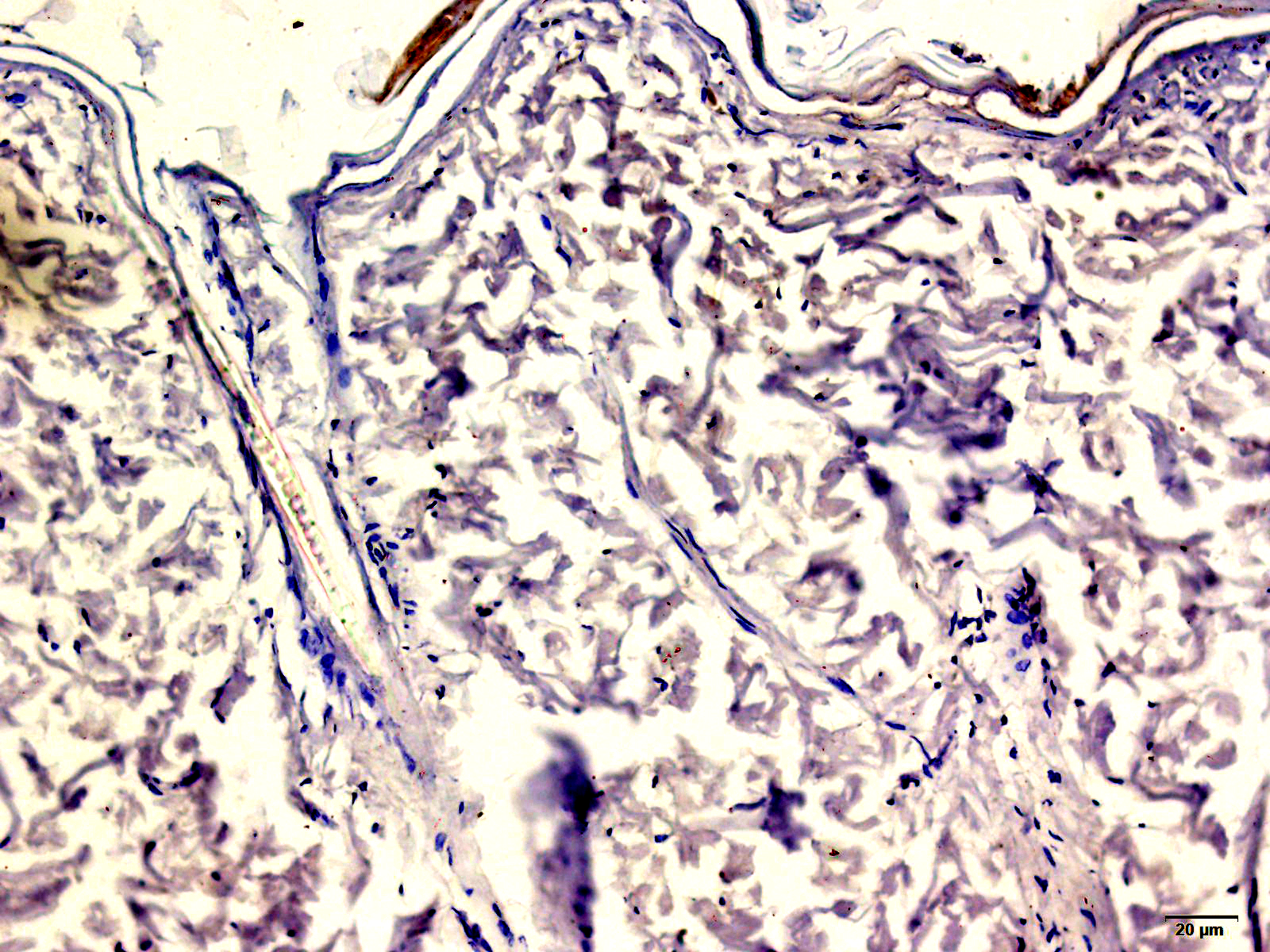

Supplement: S9 File — (ZIP) [file pone.0330078.s009.zip › immunohistochemistry/14D/Control/14D Control 3.tif]
