# Supplementary figures and images for: Efficacy and mechanism of high-purity HAMCC combined with CGF in promoting the repair of radiation-induced skin and soft tissue damage (part 3 of 3)
Source: PLoS One. 2025 Sep 9;20(9):e0330078. doi: 10.1371/journal.pone.0330078 (PMC12419615; doi:10.1371/journal.pone.0330078)

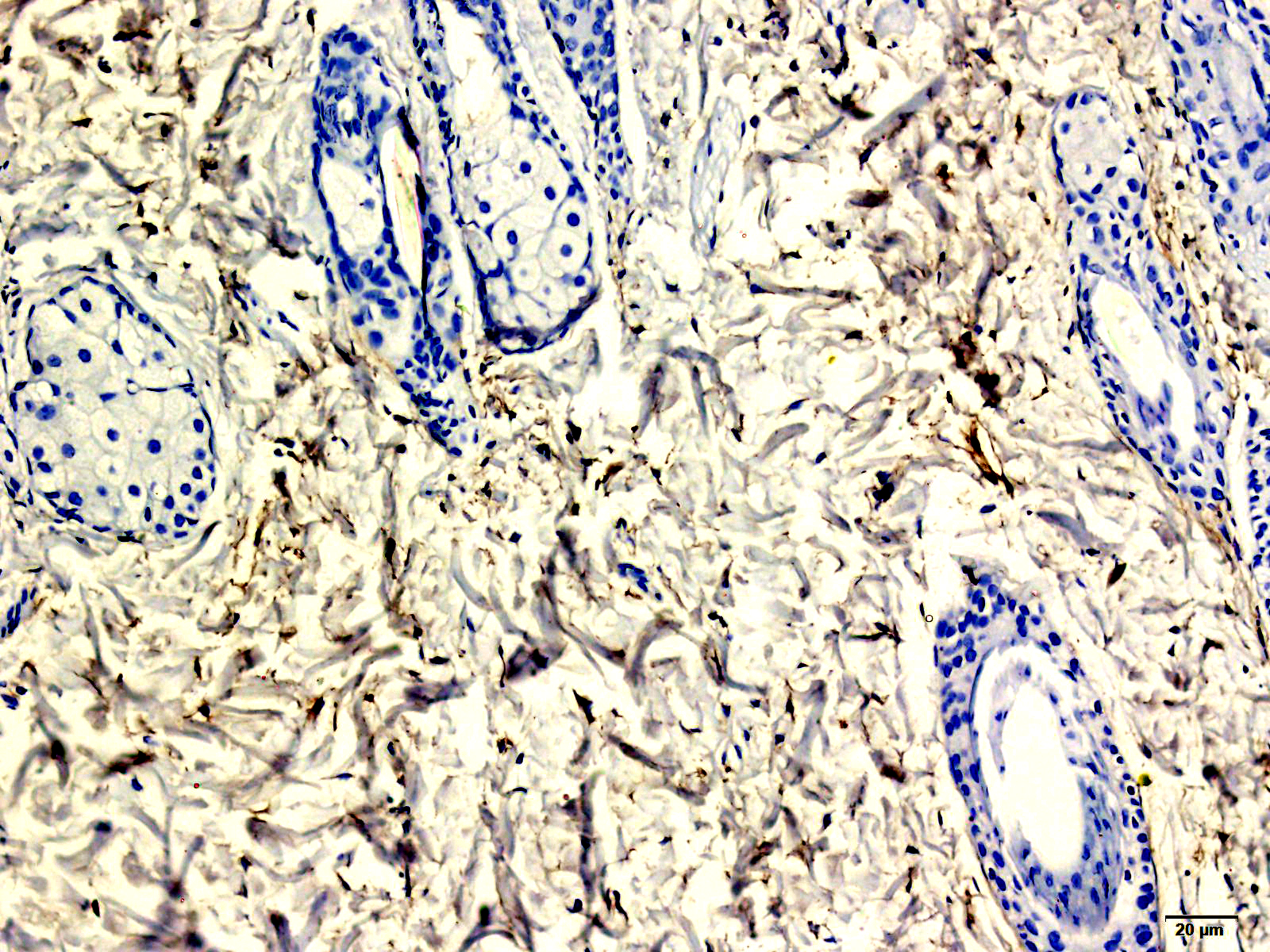

Supplement: S9 File — (ZIP) [file pone.0330078.s009.zip › immunohistochemistry/14D/HAMCC/14D HAMCC 1.tif]

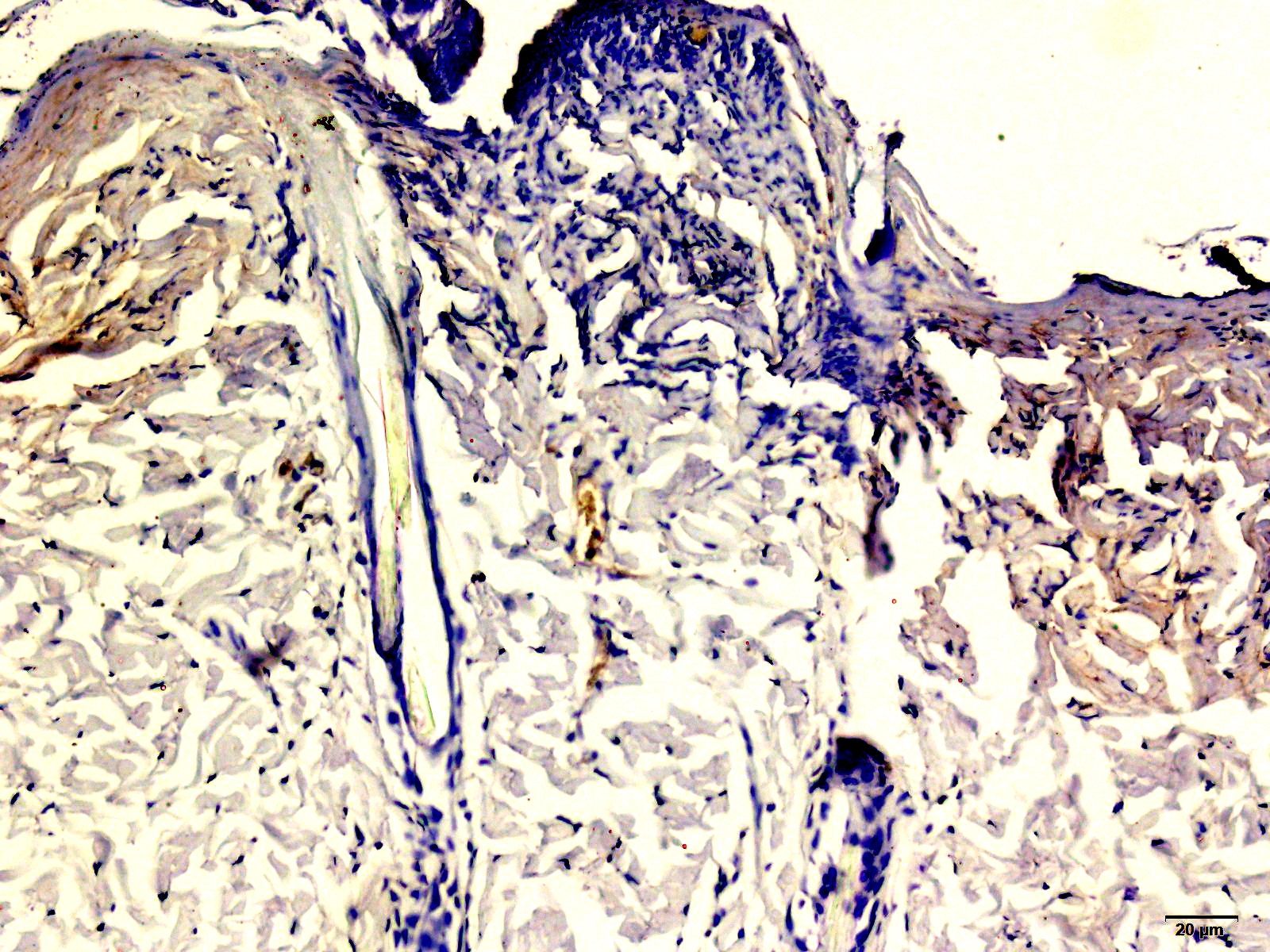

Supplement: S9 File — (ZIP) [file pone.0330078.s009.zip › immunohistochemistry/14D/HAMCC/14D HAMCC 2.tif]

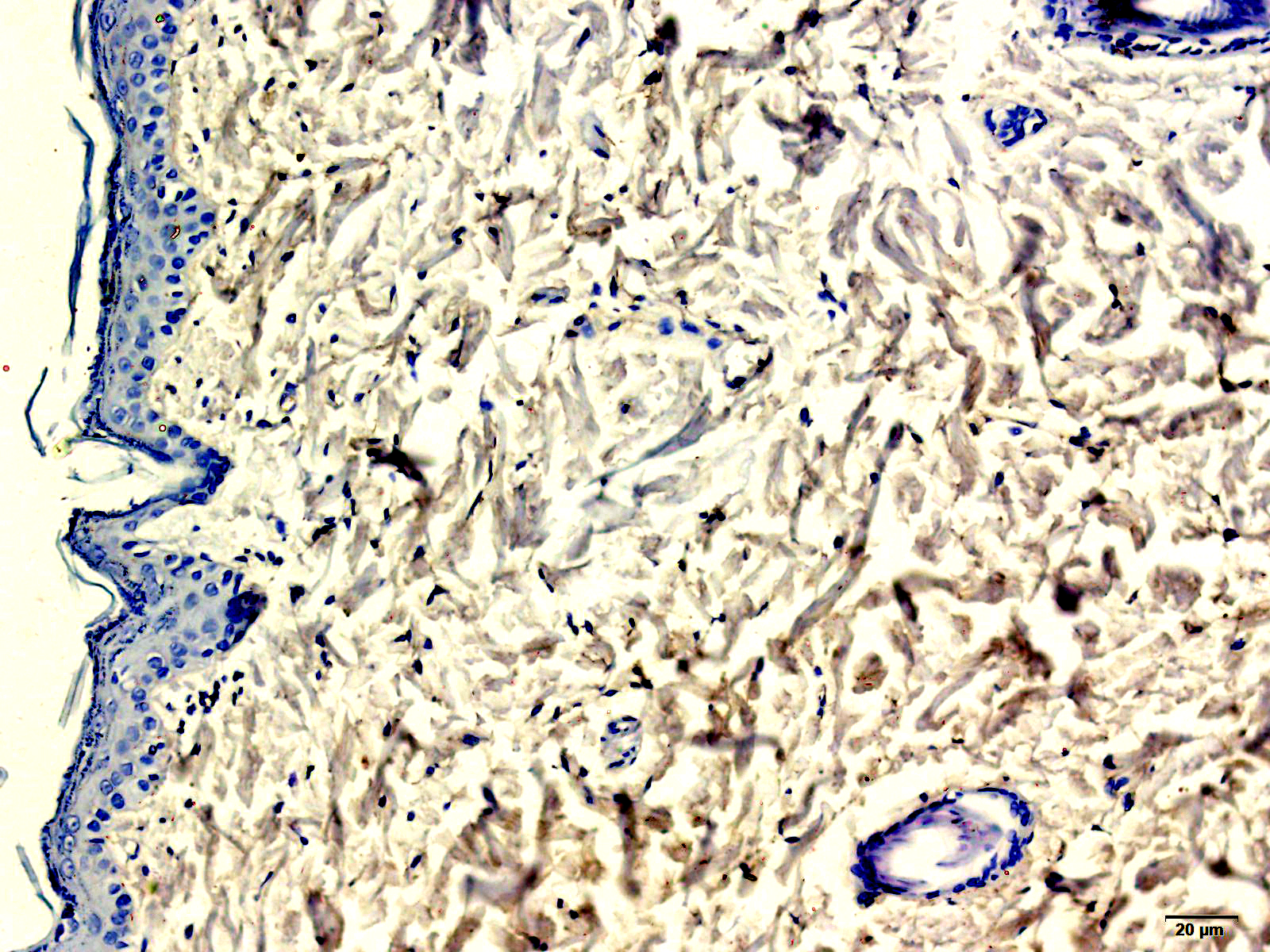

Supplement: S9 File — (ZIP) [file pone.0330078.s009.zip › immunohistochemistry/14D/HAMCC/14D HAMCC 3.tif]

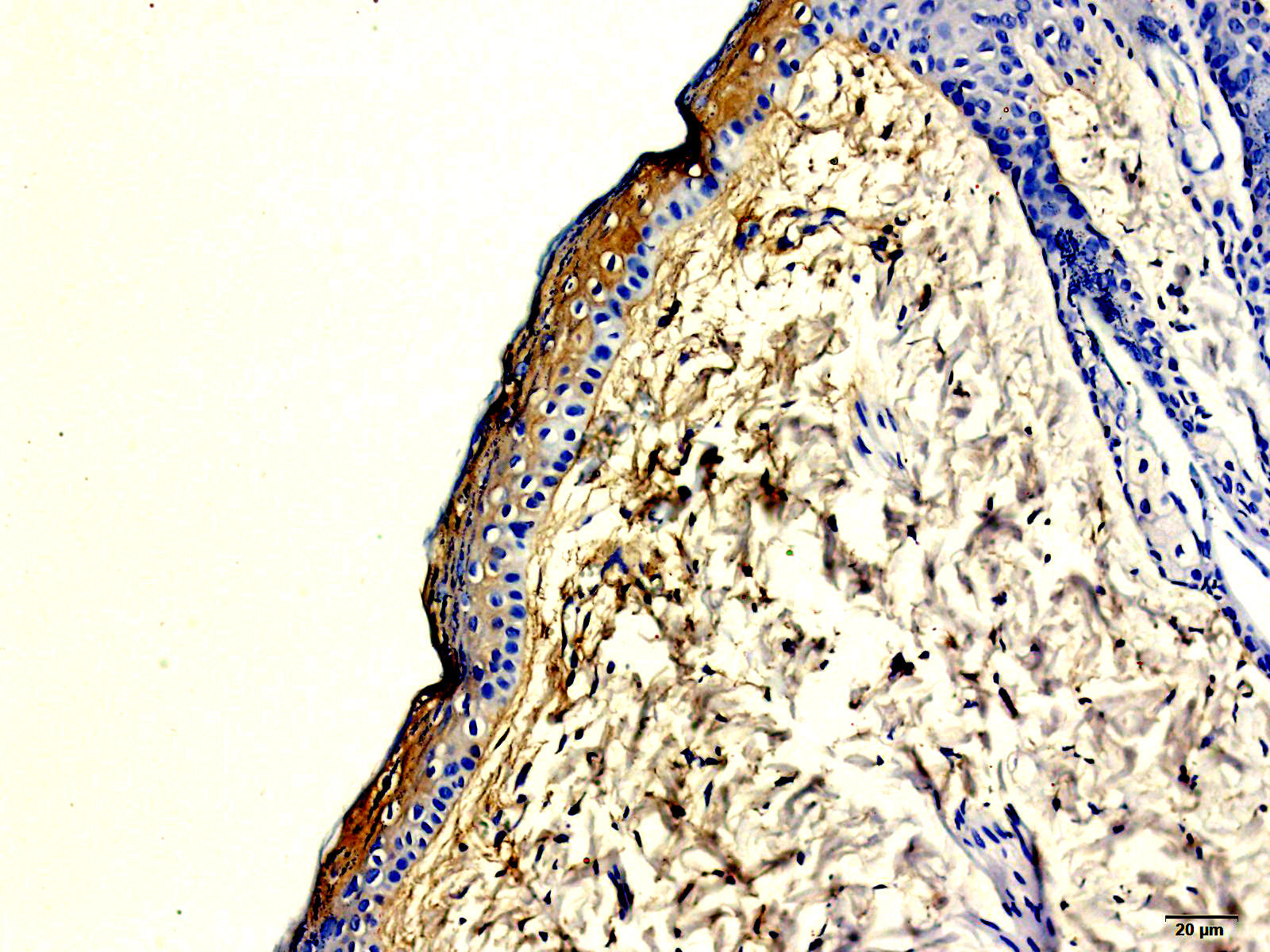

Supplement: S9 File — (ZIP) [file pone.0330078.s009.zip › immunohistochemistry/28D/CGF/28D CGF 1.tif]

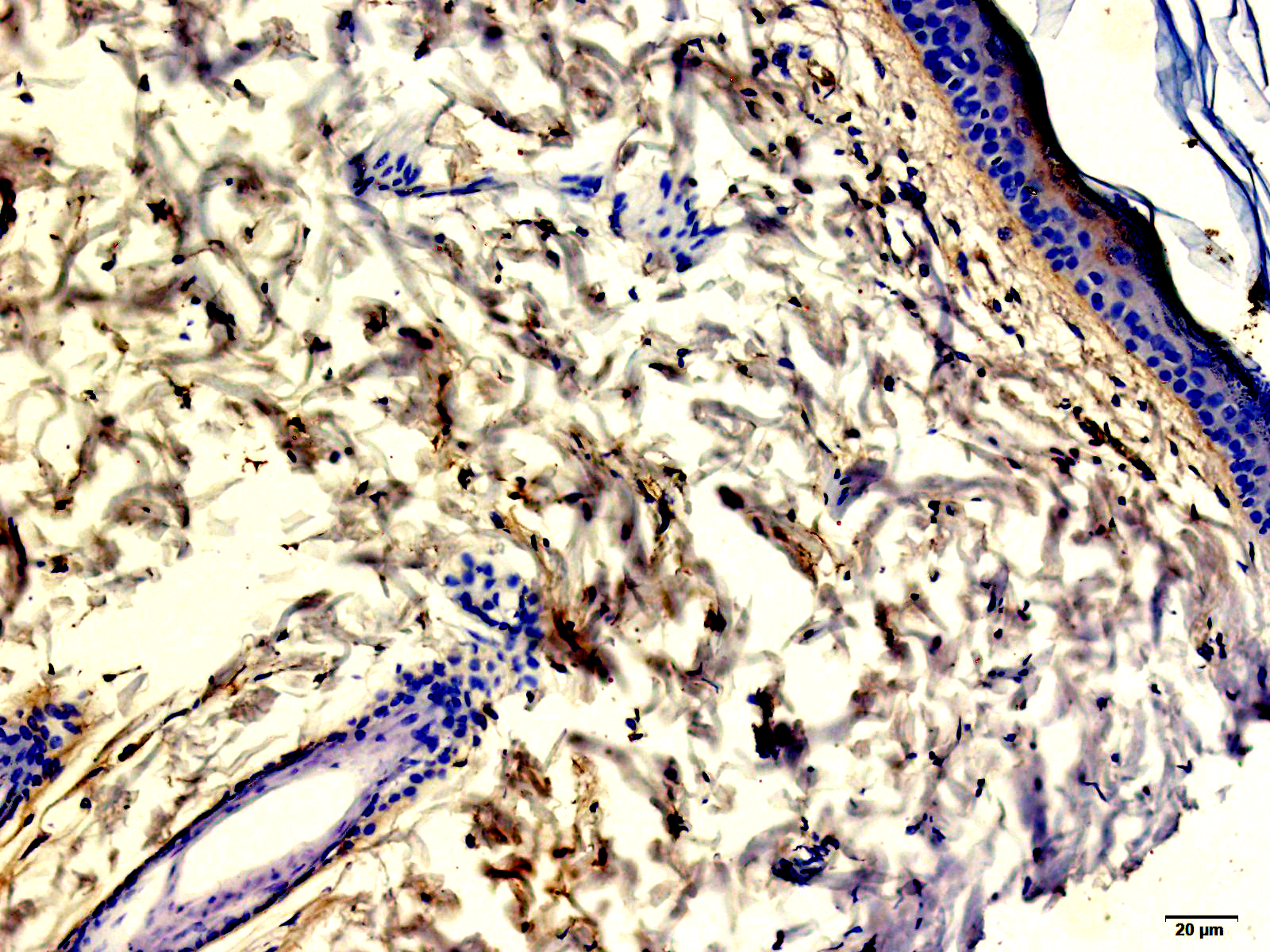

Supplement: S9 File — (ZIP) [file pone.0330078.s009.zip › immunohistochemistry/28D/CGF/28D CGF 2.tif]

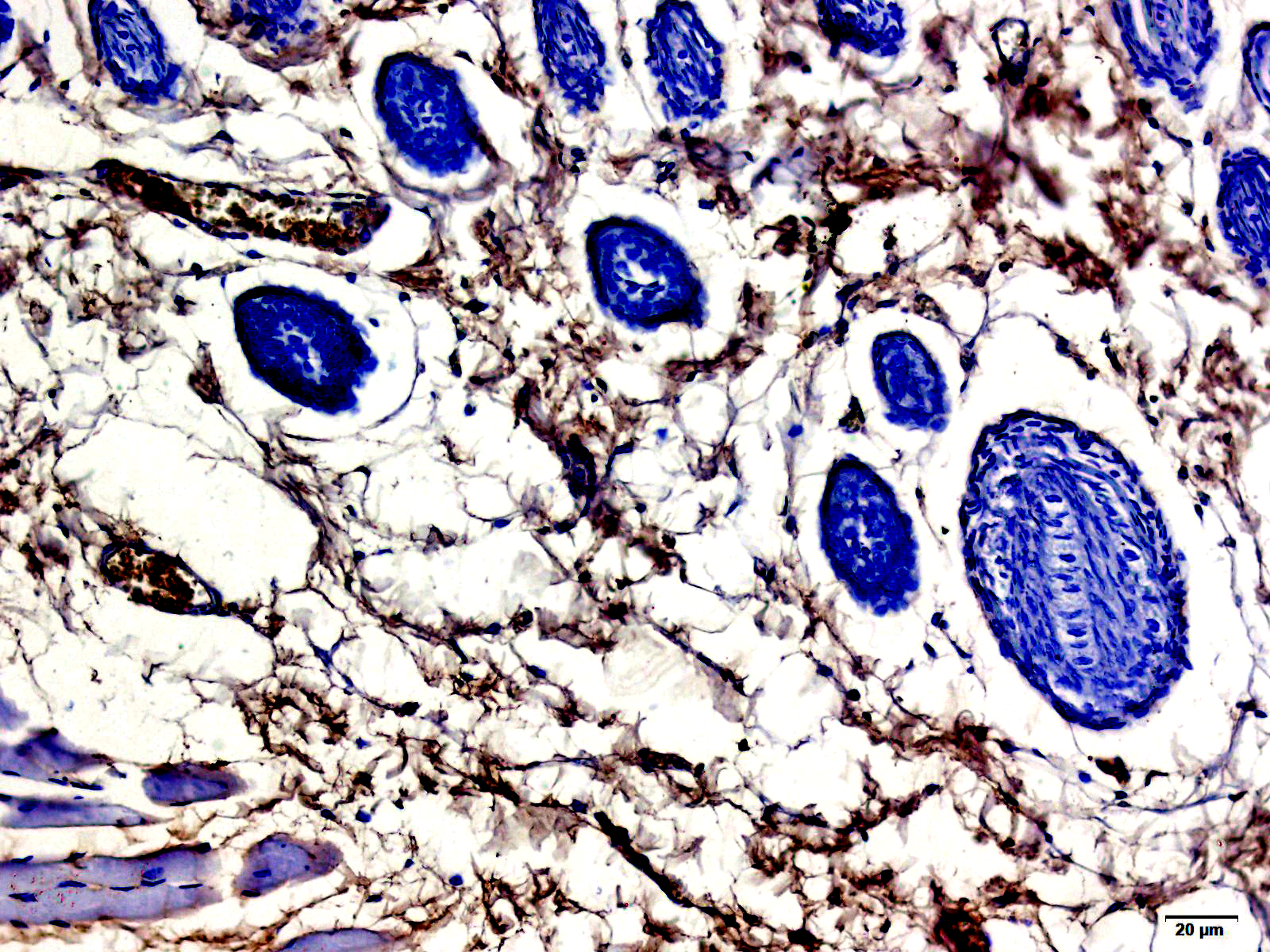

Supplement: S9 File — (ZIP) [file pone.0330078.s009.zip › immunohistochemistry/28D/CGF/28D CGF 3.tif]

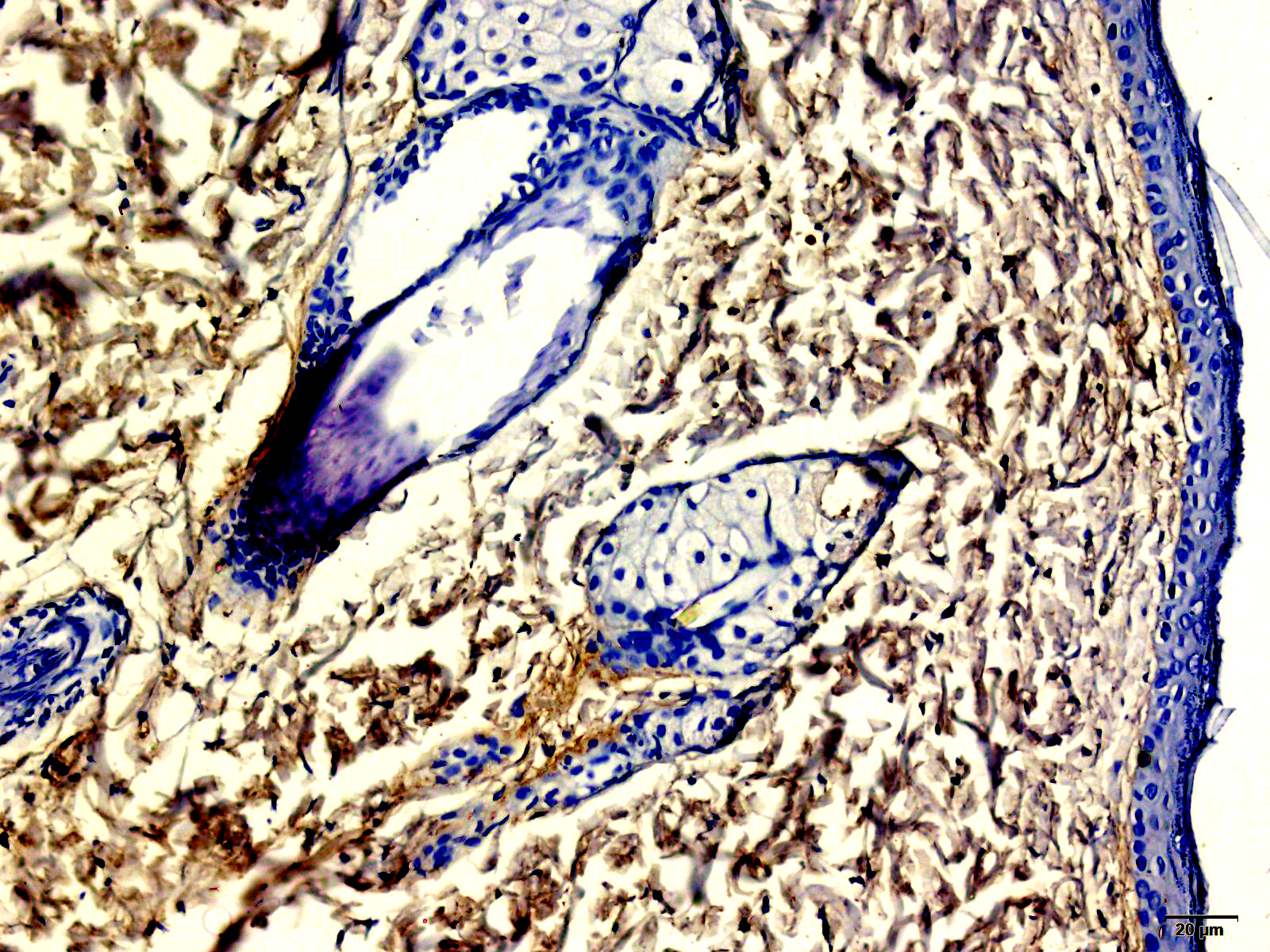

Supplement: S9 File — (ZIP) [file pone.0330078.s009.zip › immunohistochemistry/28D/CGF+HAMCC/28D CGF+HAMCC 1.tif]

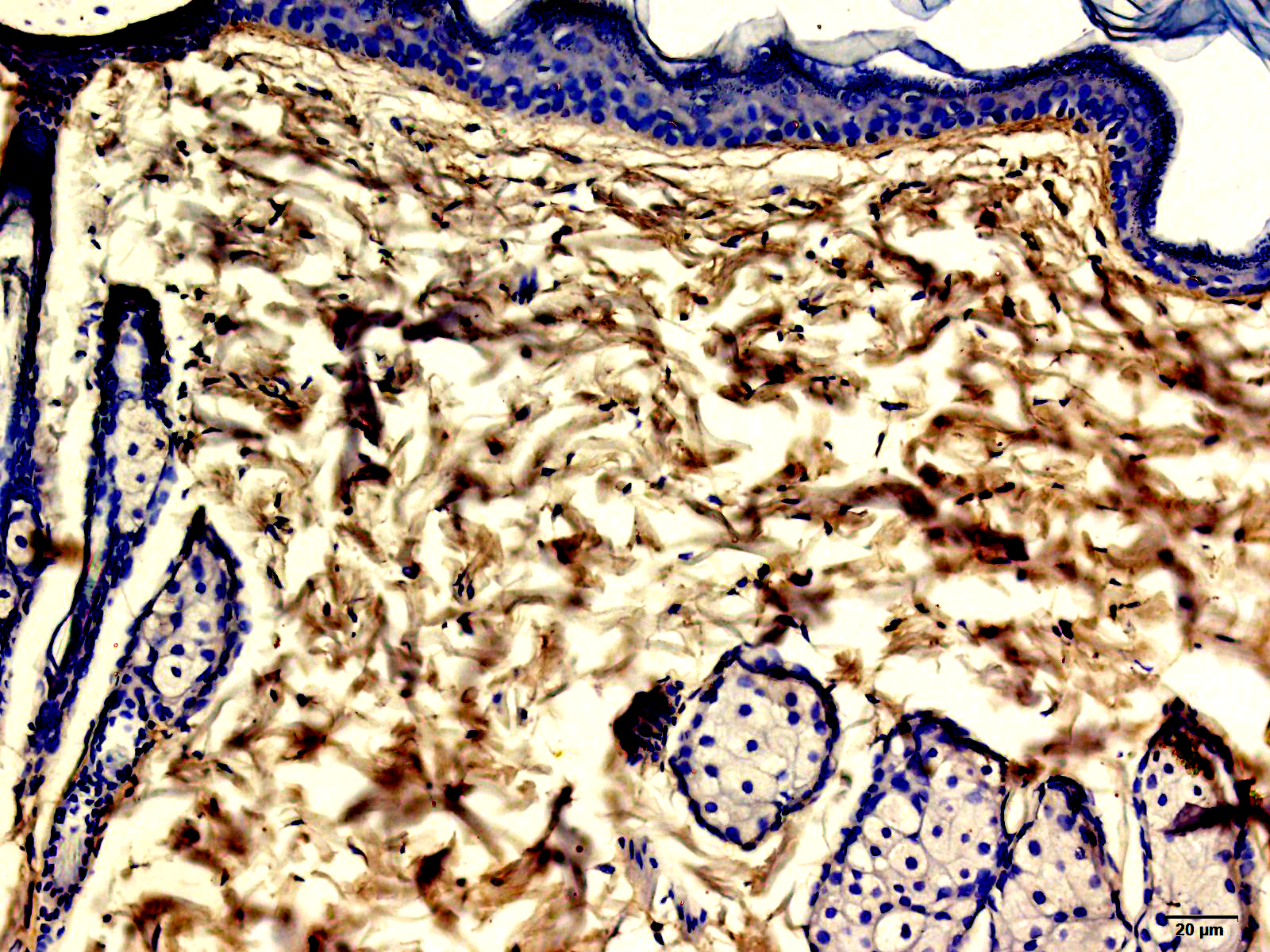

Supplement: S9 File — (ZIP) [file pone.0330078.s009.zip › immunohistochemistry/28D/CGF+HAMCC/28D CGF+HAMCC 2.tif]

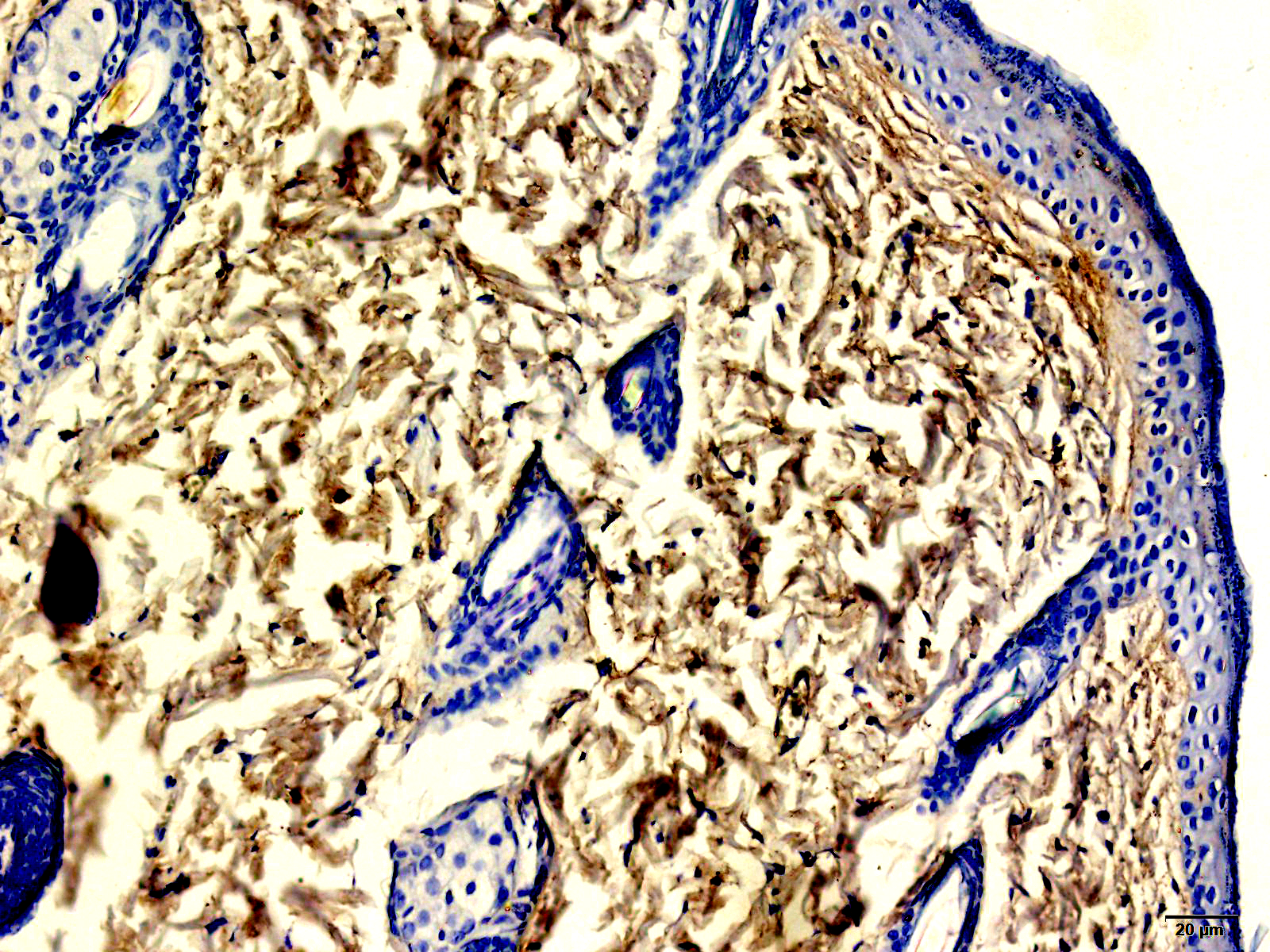

Supplement: S9 File — (ZIP) [file pone.0330078.s009.zip › immunohistochemistry/28D/CGF+HAMCC/28D CGF+HAMCC 3.tif]

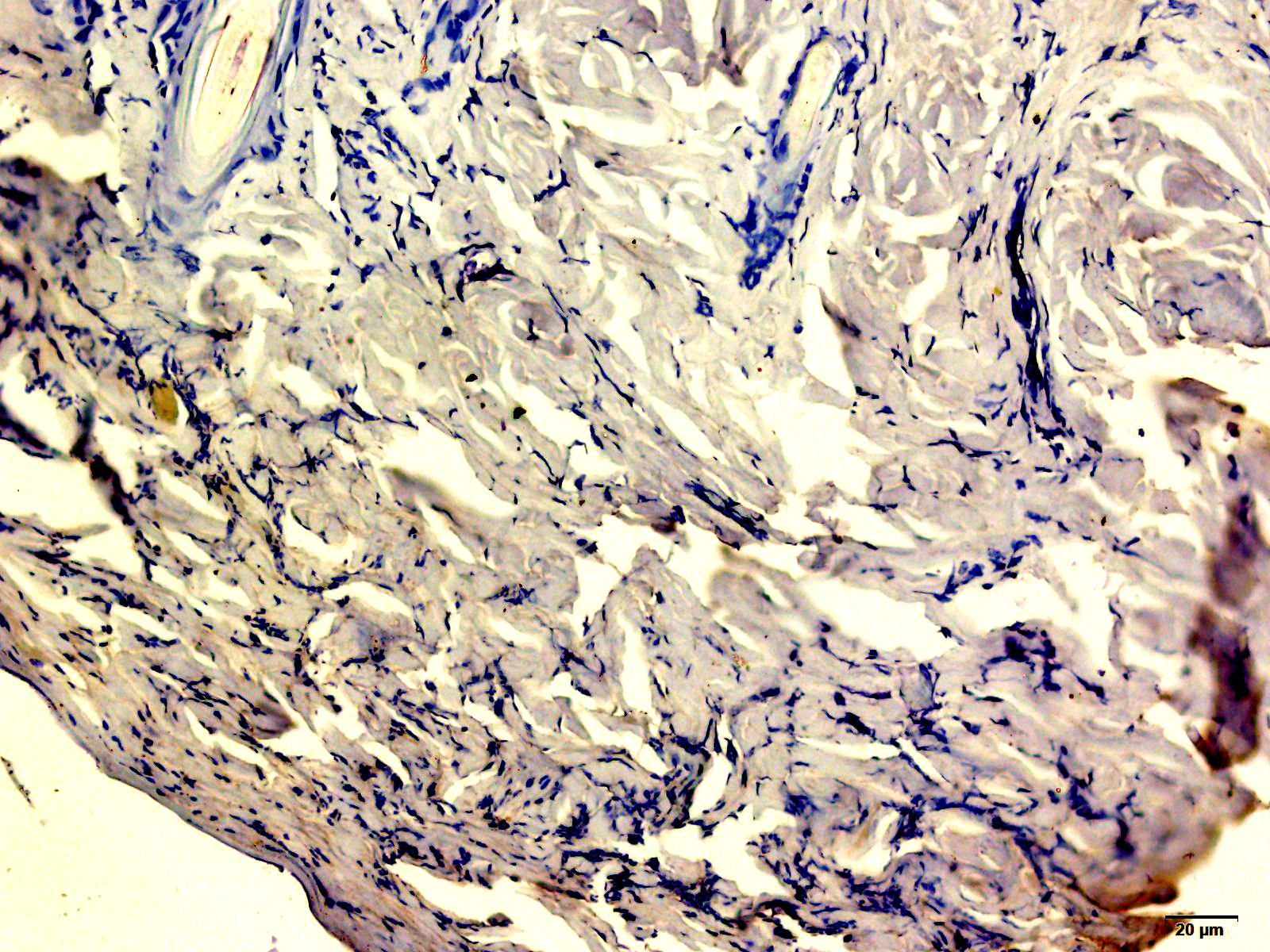

Supplement: S9 File — (ZIP) [file pone.0330078.s009.zip › immunohistochemistry/28D/Control/28D Control 1.tif]

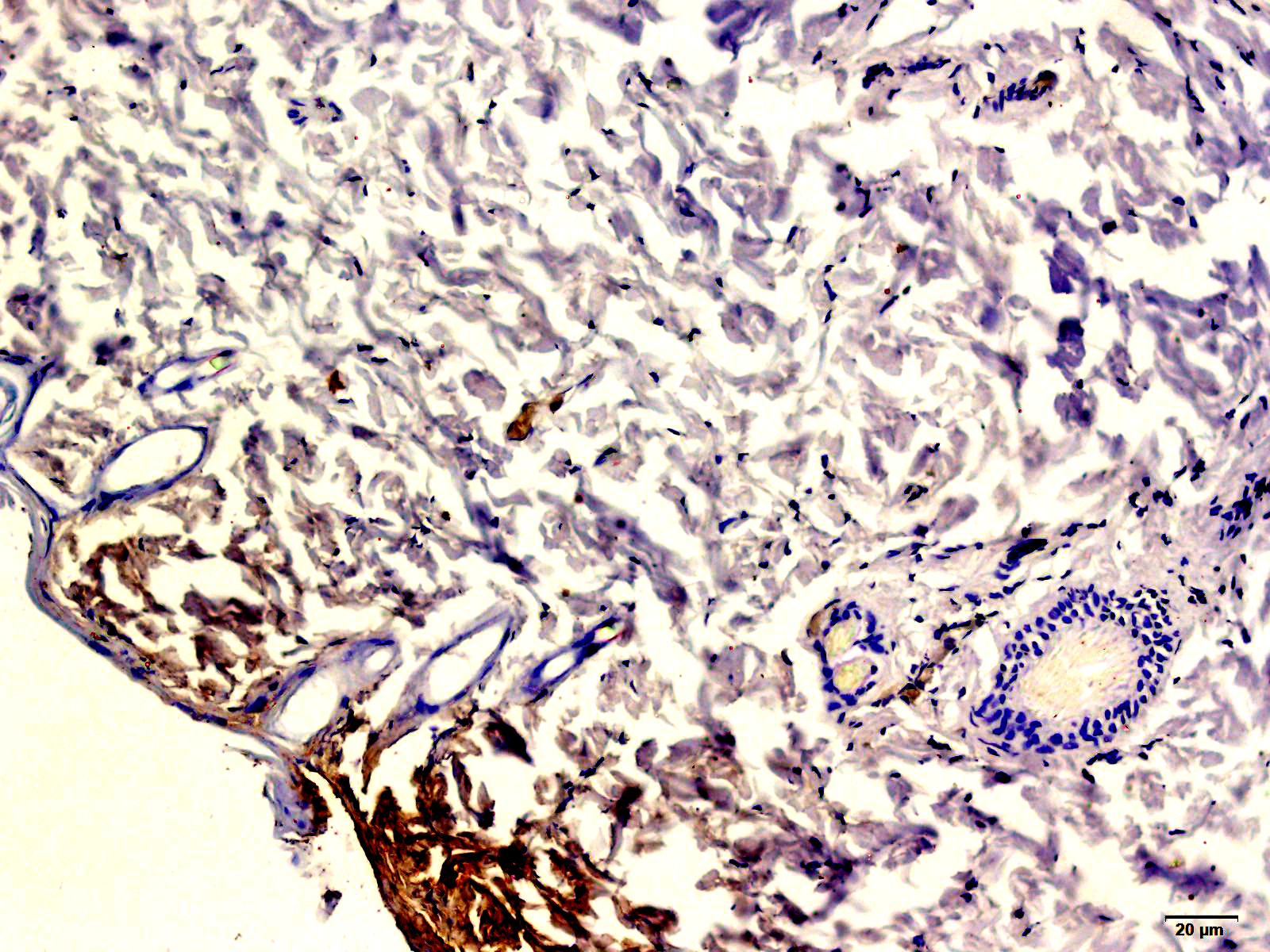

Supplement: S9 File — (ZIP) [file pone.0330078.s009.zip › immunohistochemistry/28D/Control/28D Control 2.tif]

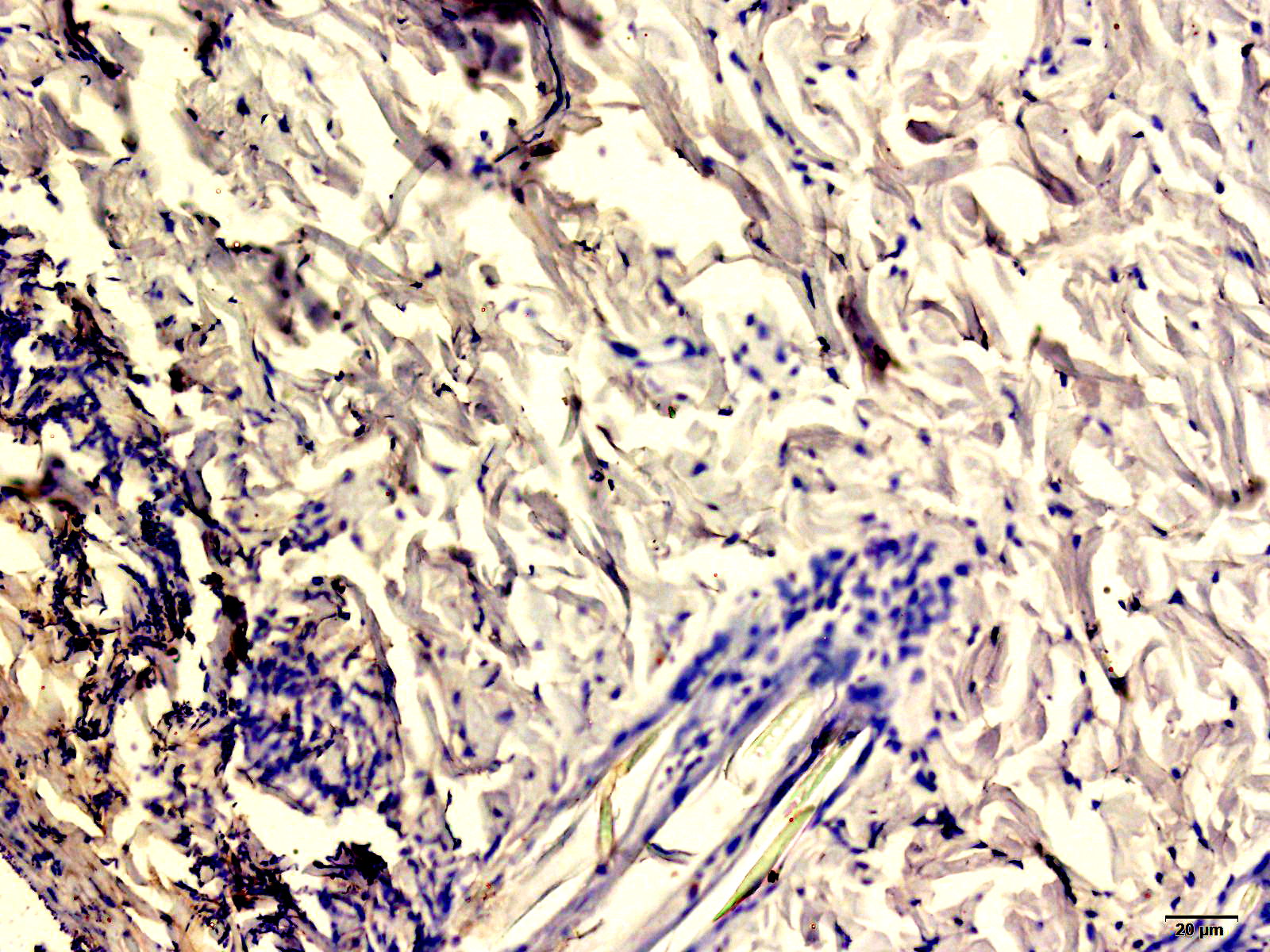

Supplement: S9 File — (ZIP) [file pone.0330078.s009.zip › immunohistochemistry/28D/Control/28D Control 3.tif]

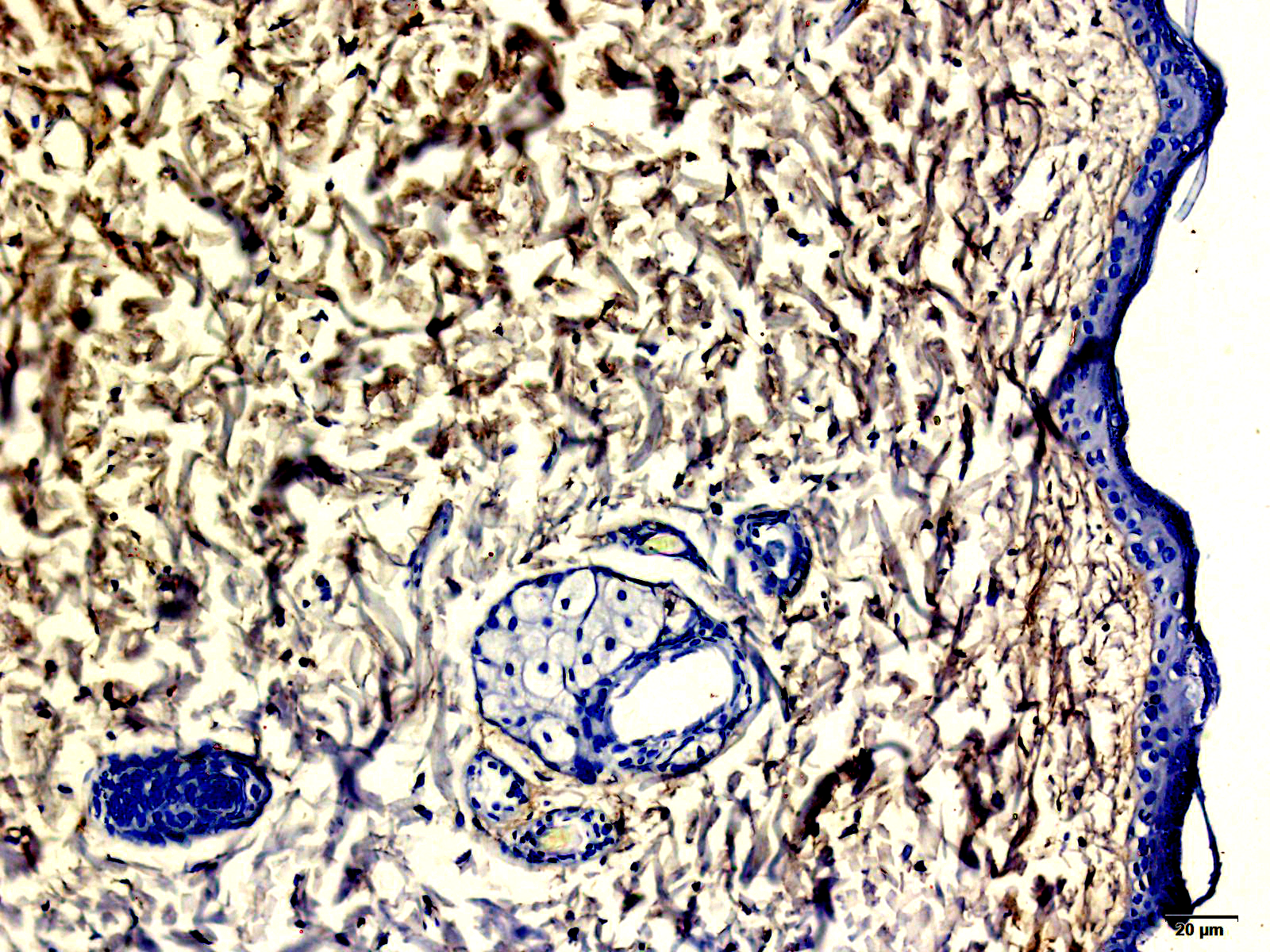

Supplement: S9 File — (ZIP) [file pone.0330078.s009.zip › immunohistochemistry/28D/HAMCC/28D HAMCC 1.tif]

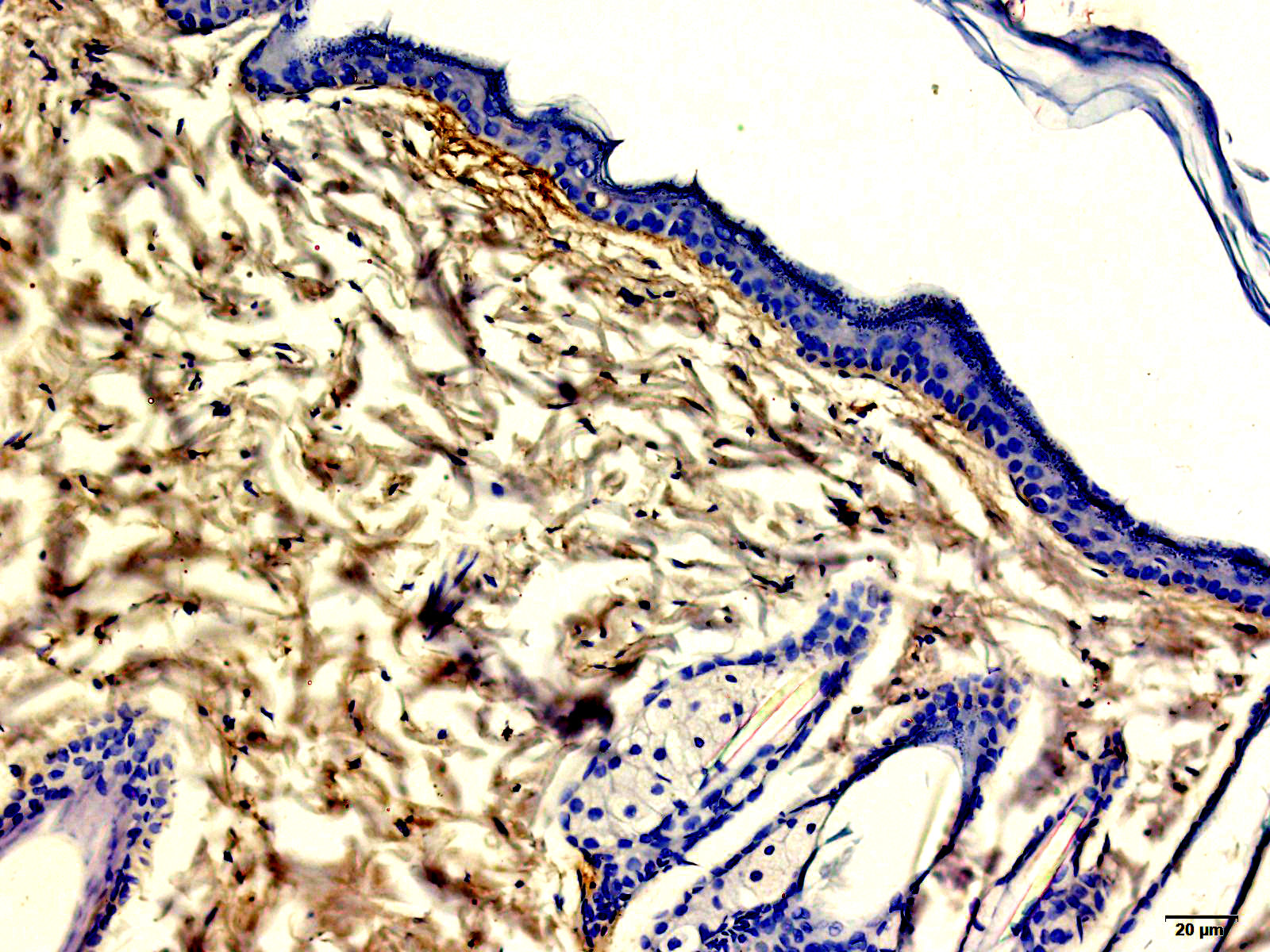

Supplement: S9 File — (ZIP) [file pone.0330078.s009.zip › immunohistochemistry/28D/HAMCC/28D HAMCC 2.tif]

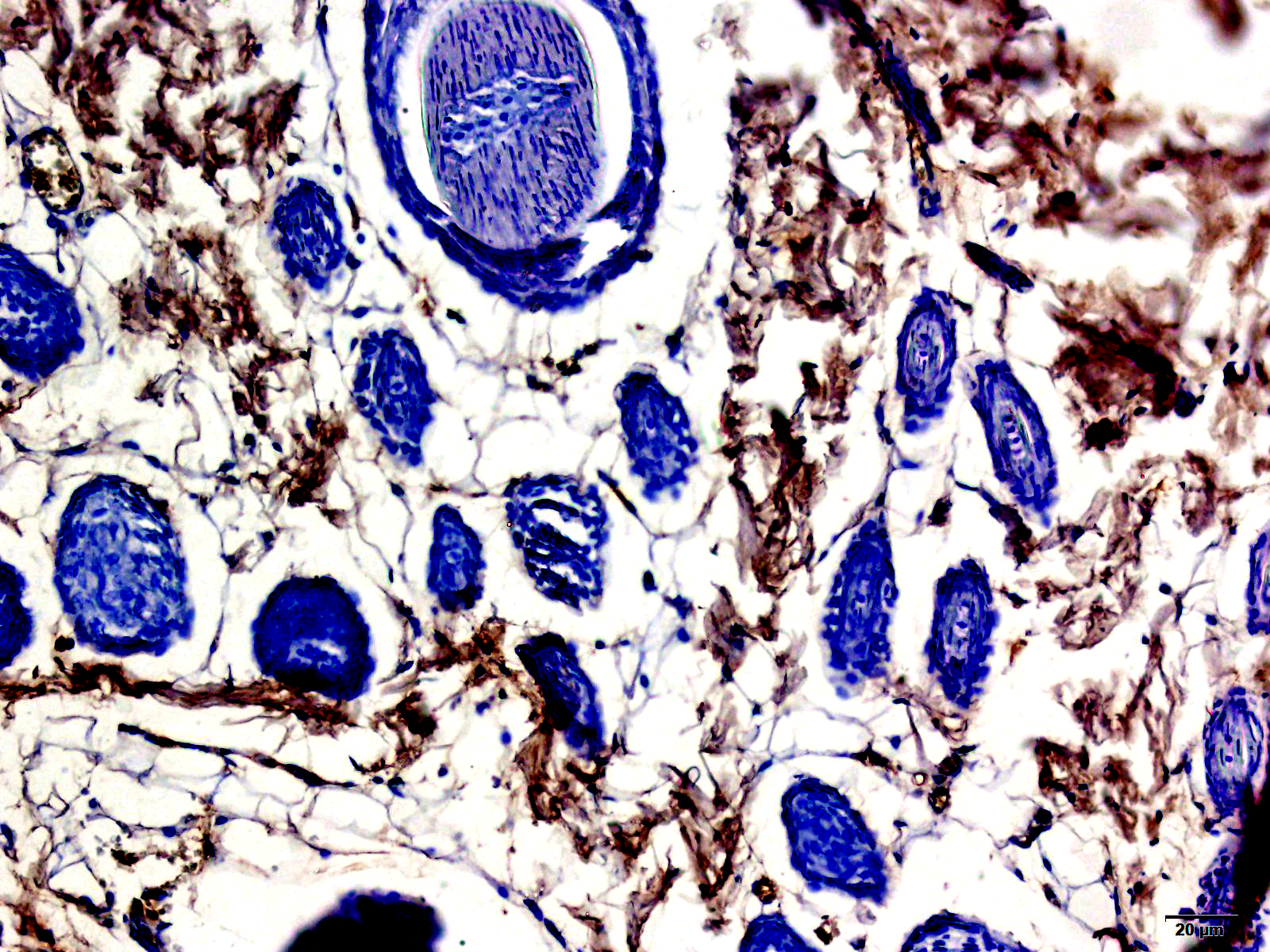

Supplement: S9 File — (ZIP) [file pone.0330078.s009.zip › immunohistochemistry/28D/HAMCC/28D HAMCC 3.tif]

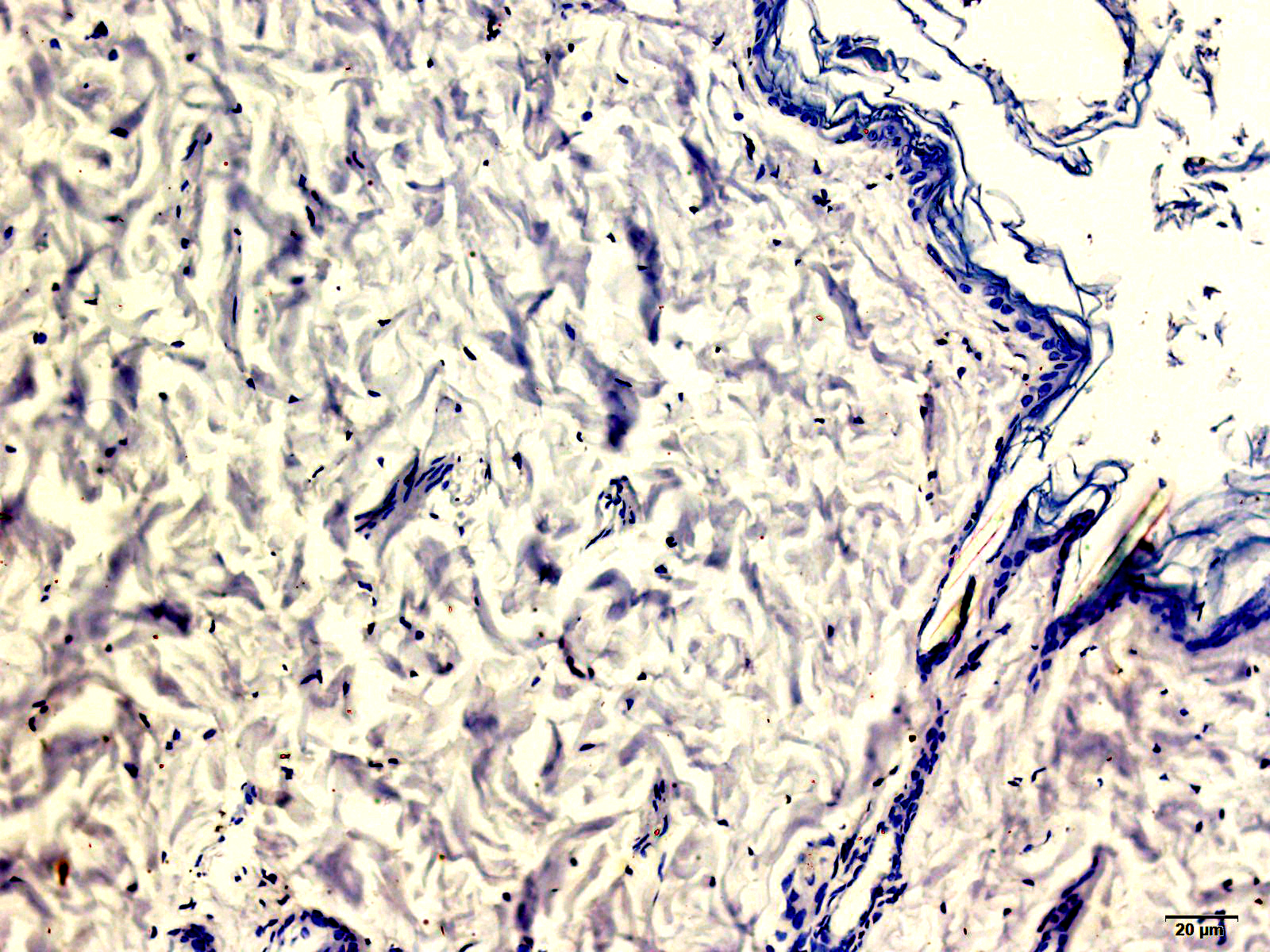

Supplement: S9 File — (ZIP) [file pone.0330078.s009.zip › immunohistochemistry/7D/CGF/7D CGF 1.tif]

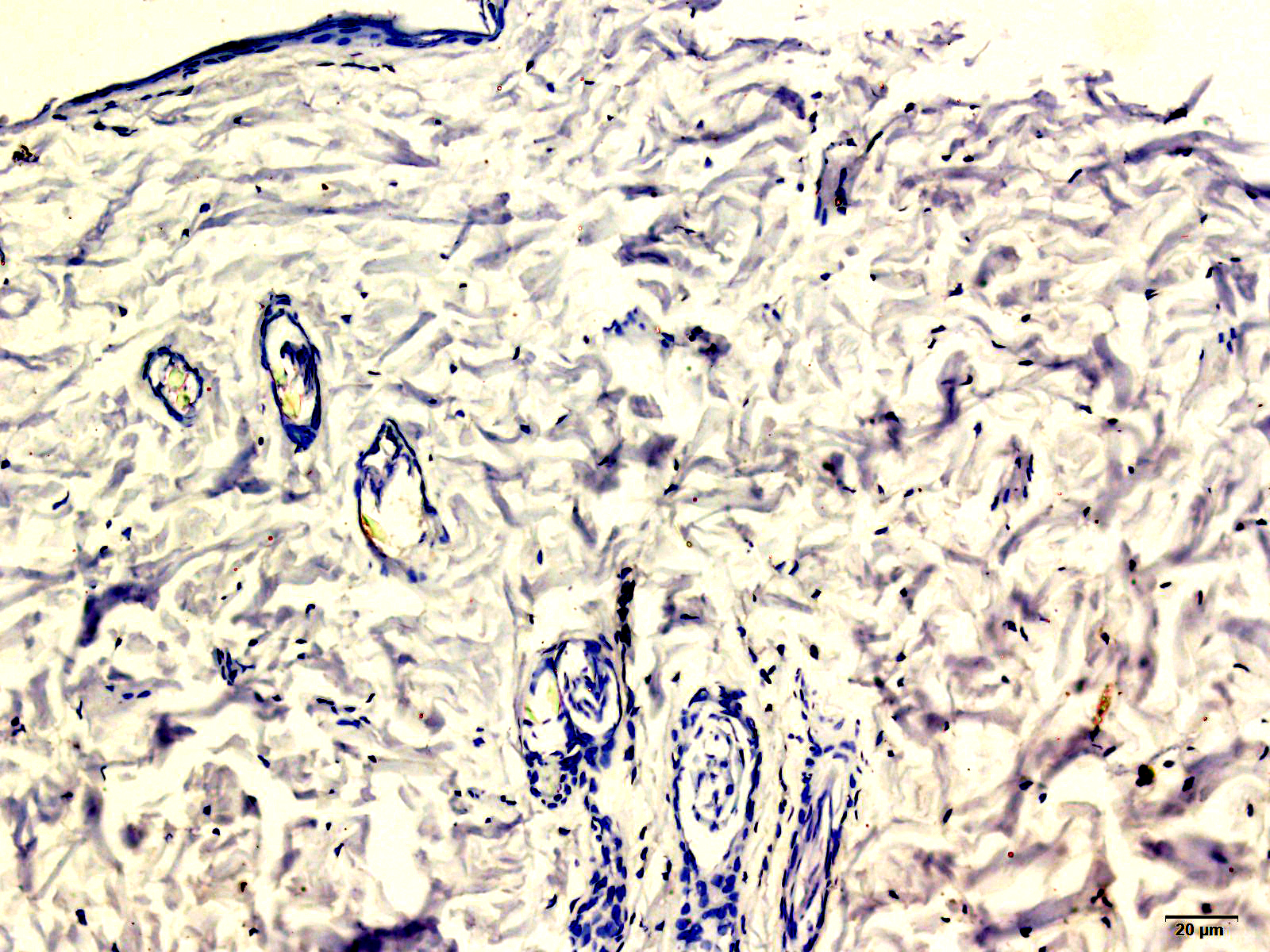

Supplement: S9 File — (ZIP) [file pone.0330078.s009.zip › immunohistochemistry/7D/CGF/7D CGF 2.tif]

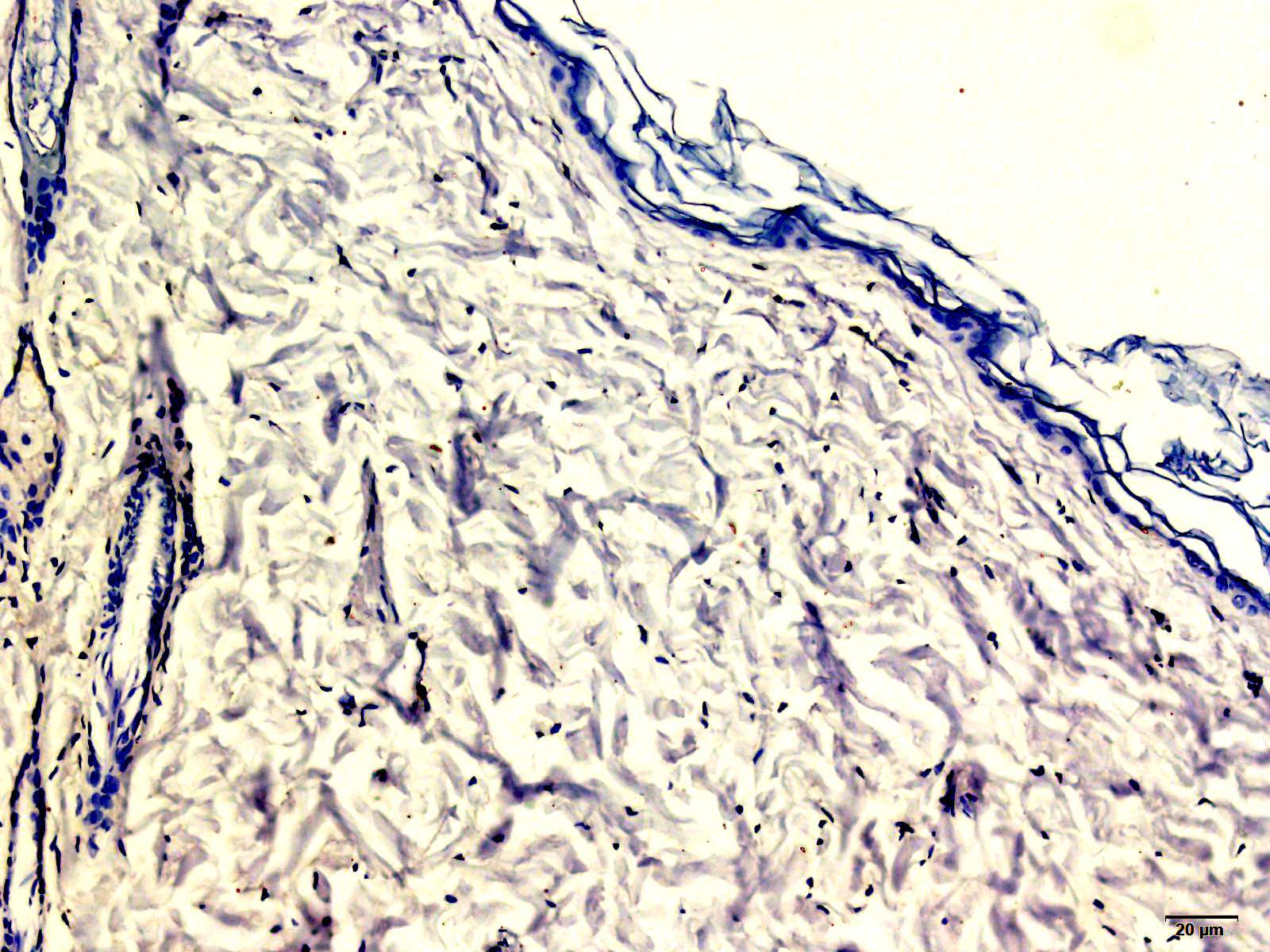

Supplement: S9 File — (ZIP) [file pone.0330078.s009.zip › immunohistochemistry/7D/CGF/7D CGF 3.tif]

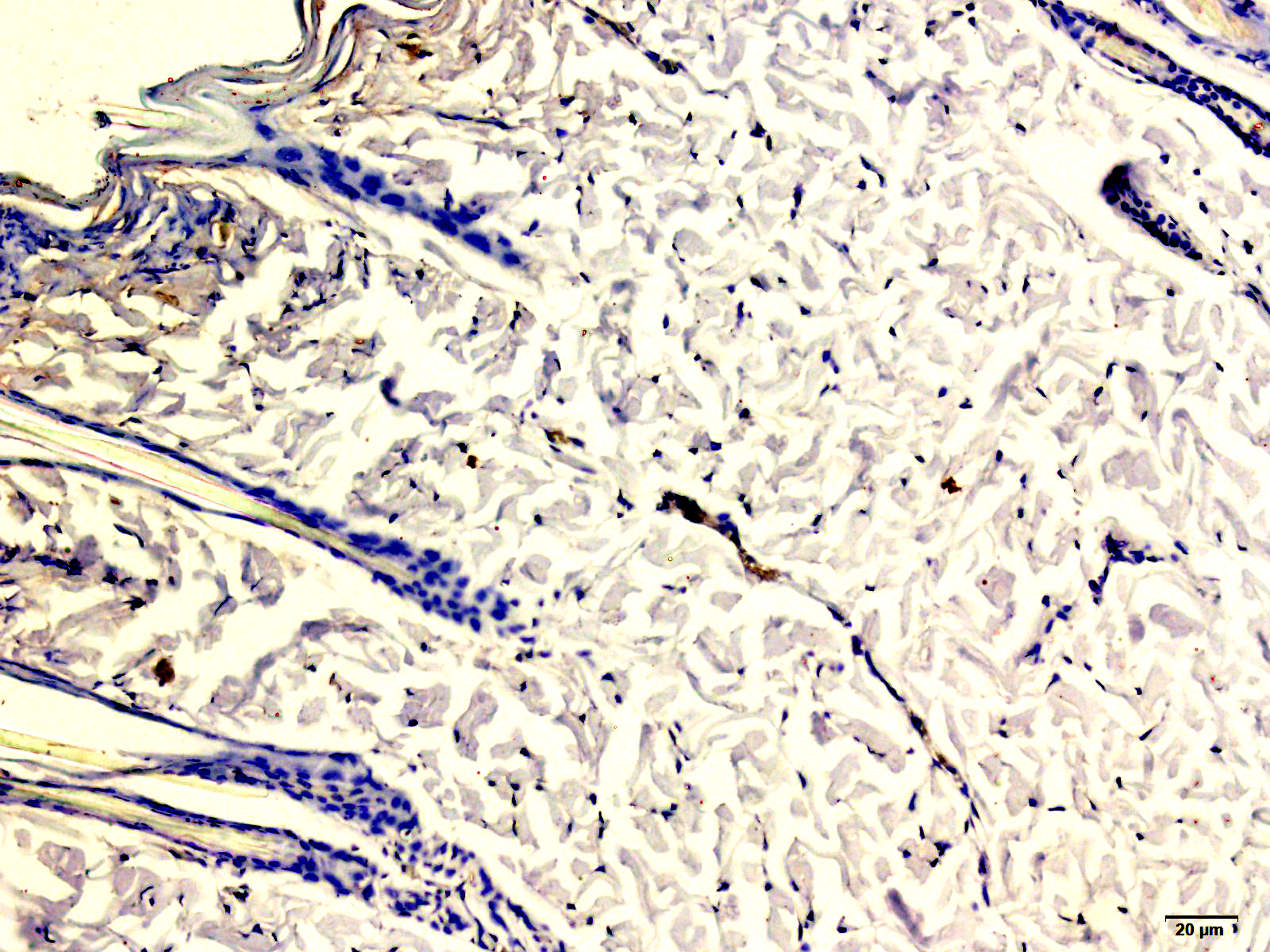

Supplement: S9 File — (ZIP) [file pone.0330078.s009.zip › immunohistochemistry/7D/CGF+HAMCC/7D CGF+HAMCC 1.tif]

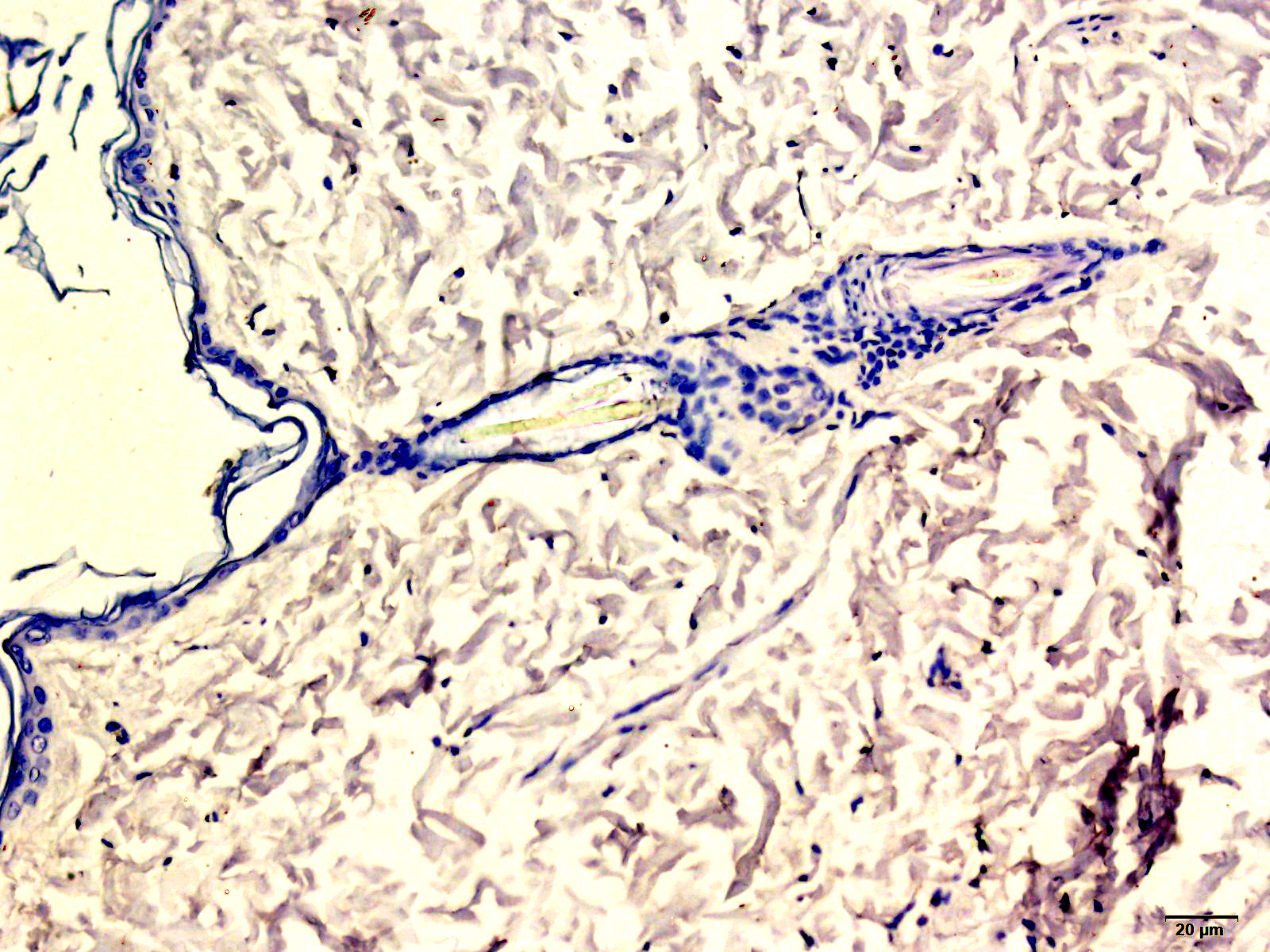

Supplement: S9 File — (ZIP) [file pone.0330078.s009.zip › immunohistochemistry/7D/CGF+HAMCC/7D CGF+HAMCC 2.tif]

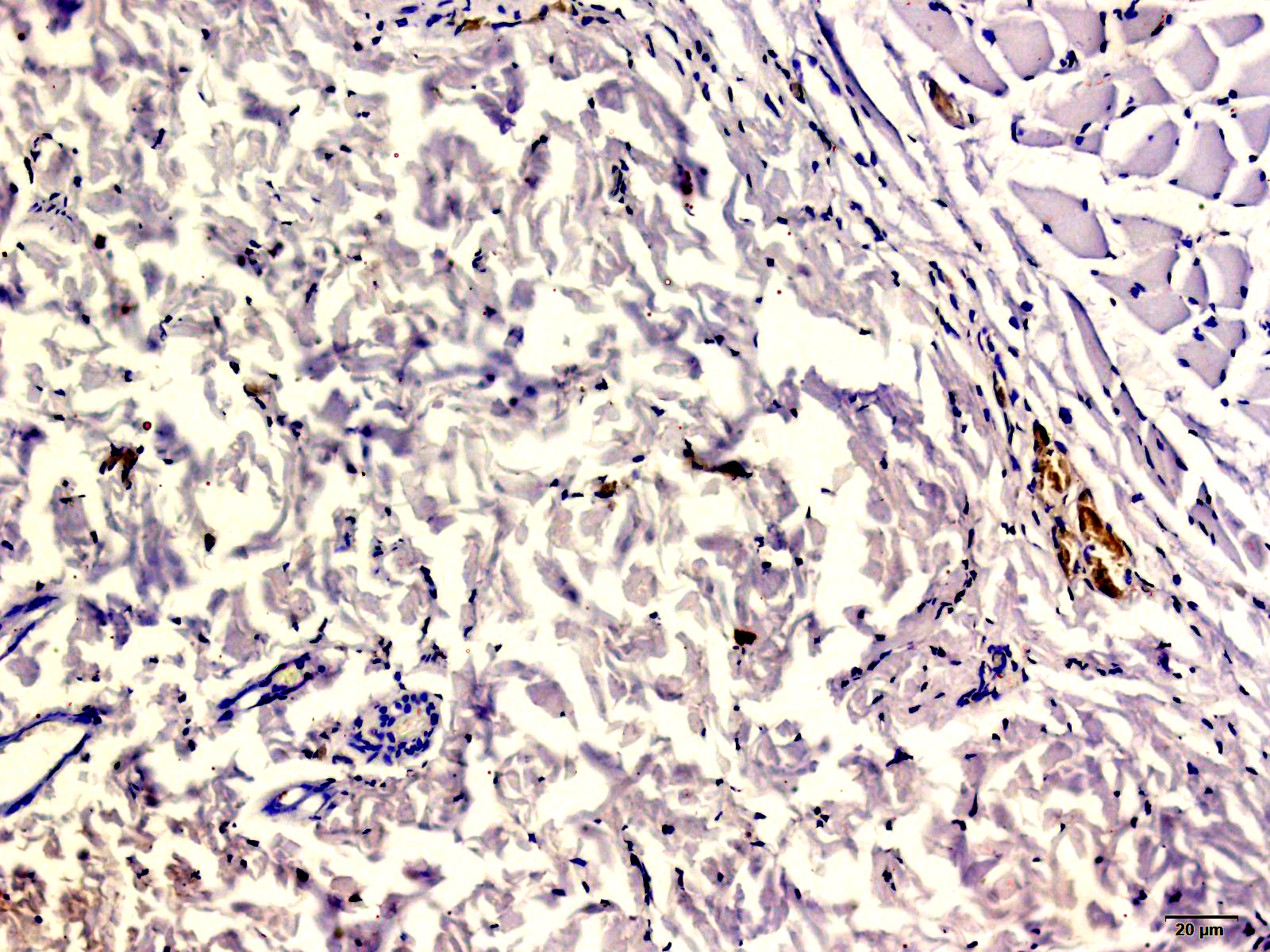

Supplement: S9 File — (ZIP) [file pone.0330078.s009.zip › immunohistochemistry/7D/CGF+HAMCC/7D CGF+HAMCC 3.tif]

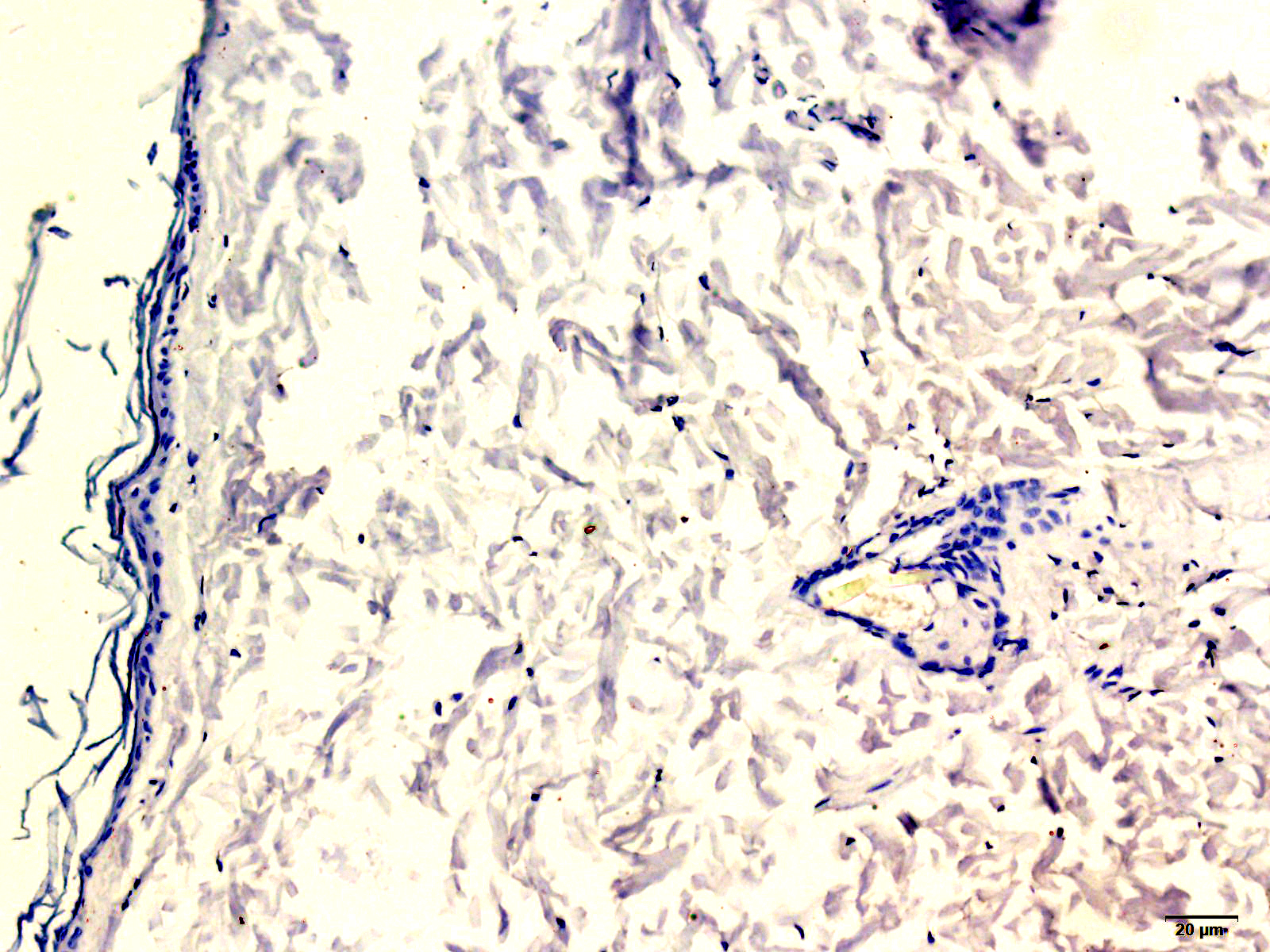

Supplement: S9 File — (ZIP) [file pone.0330078.s009.zip › immunohistochemistry/7D/Control/7D Control 1.tif]

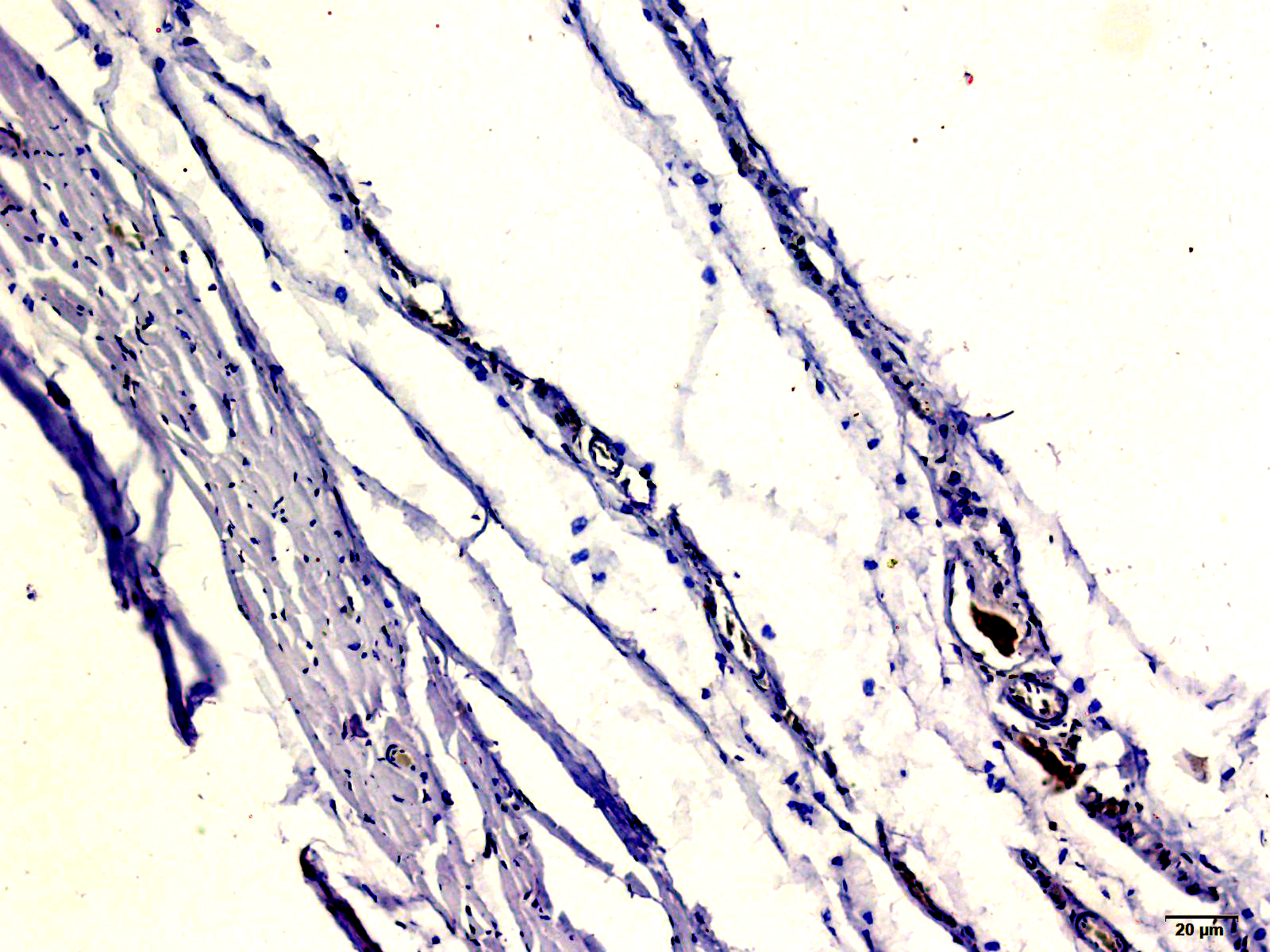

Supplement: S9 File — (ZIP) [file pone.0330078.s009.zip › immunohistochemistry/7D/Control/7D Control 2.tif]

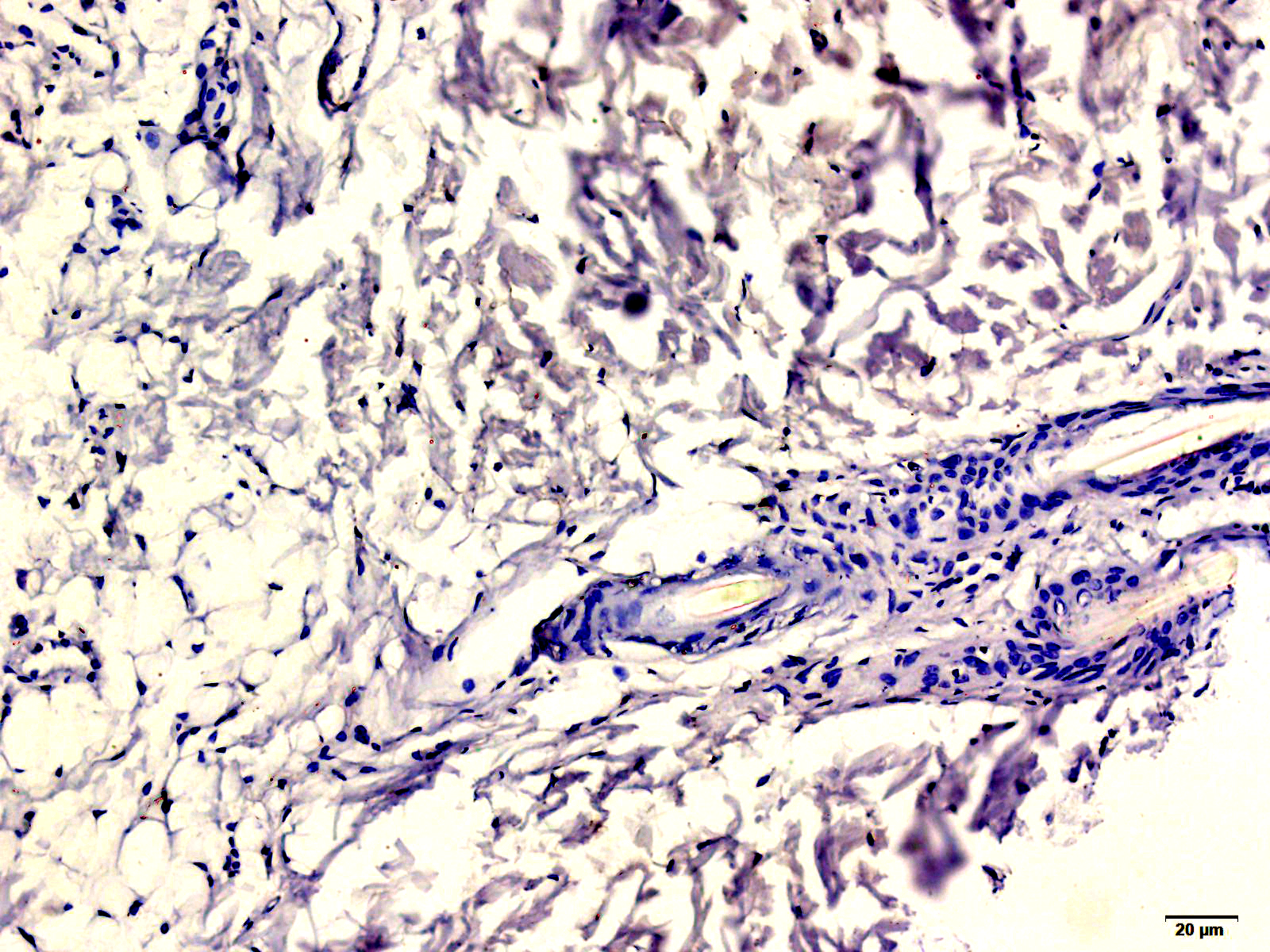

Supplement: S9 File — (ZIP) [file pone.0330078.s009.zip › immunohistochemistry/7D/Control/7D Control 3.tif]

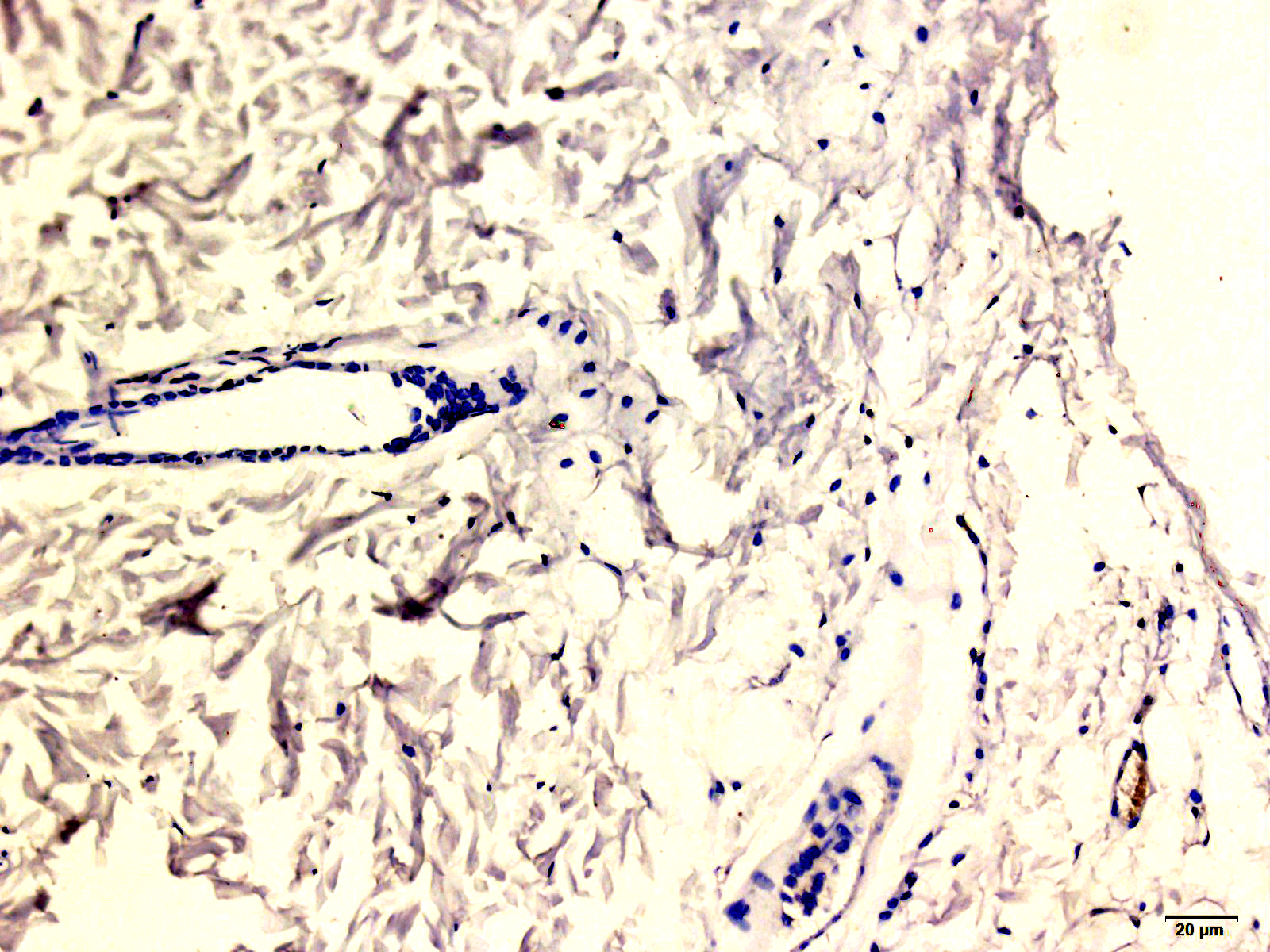

Supplement: S9 File — (ZIP) [file pone.0330078.s009.zip › immunohistochemistry/7D/HAMCC/7D HAMCC 1.tif]

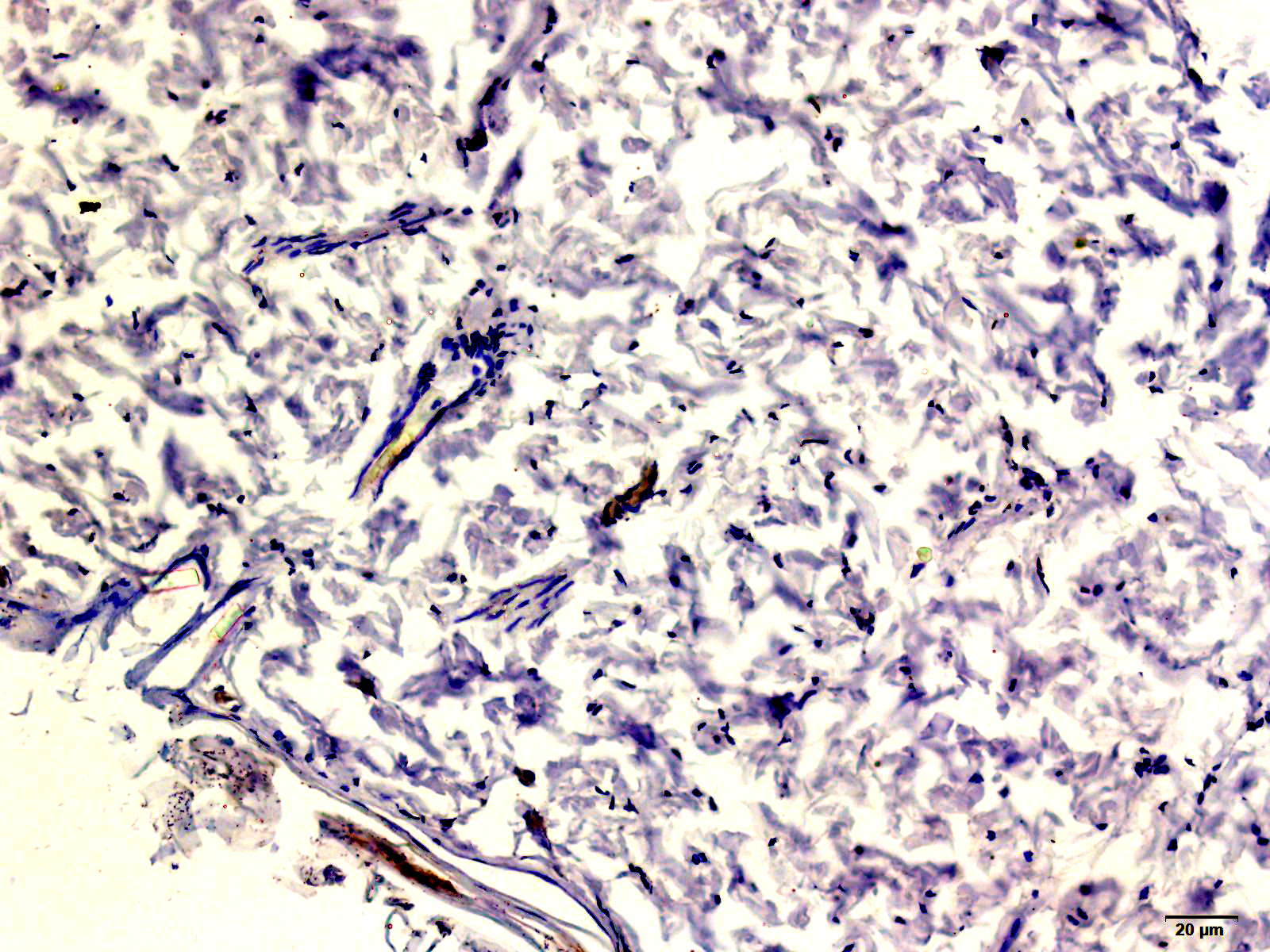

Supplement: S9 File — (ZIP) [file pone.0330078.s009.zip › immunohistochemistry/7D/HAMCC/7D HAMCC 2.tif]

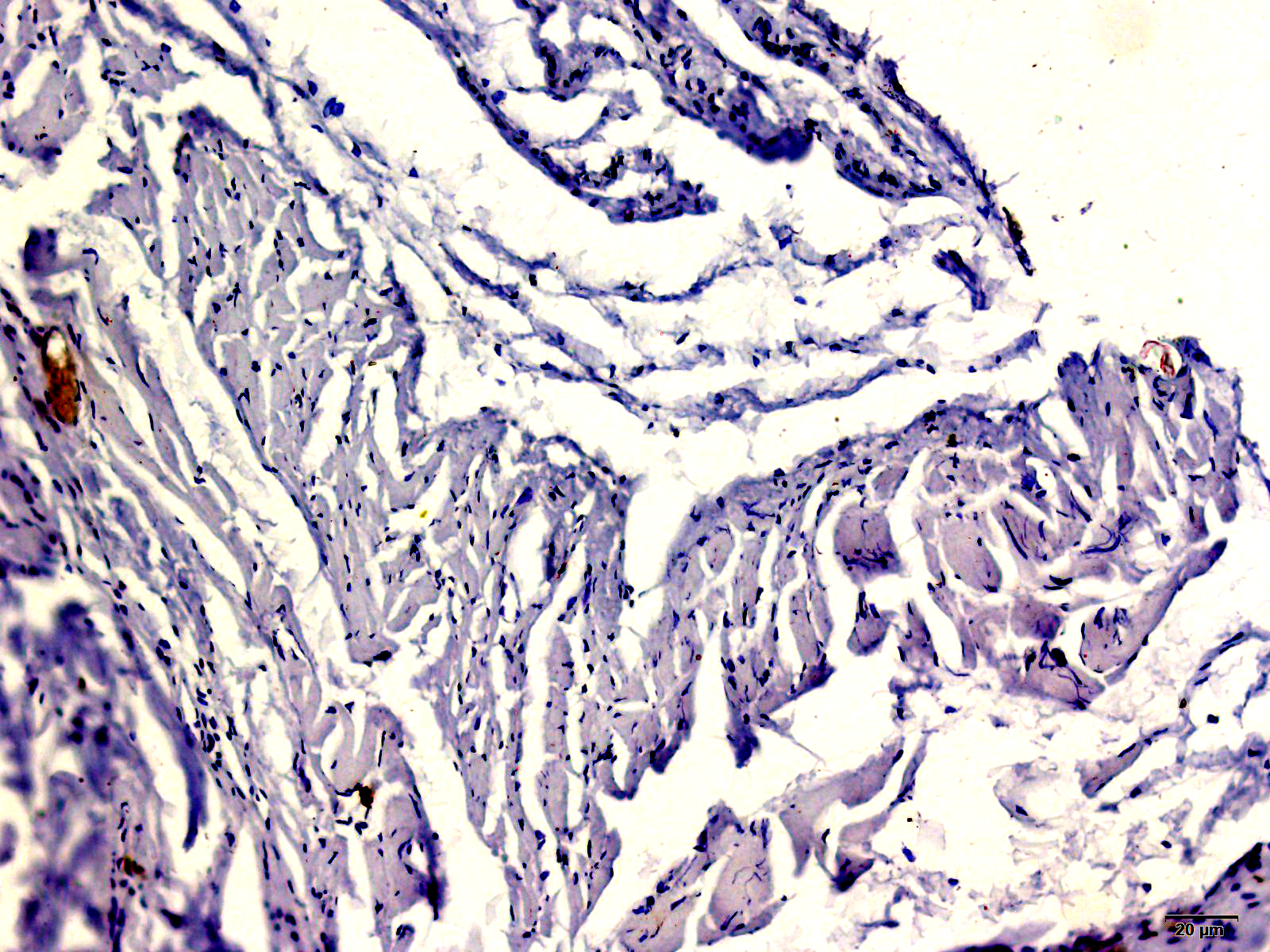

Supplement: S9 File — (ZIP) [file pone.0330078.s009.zip › immunohistochemistry/7D/HAMCC/7D HAMCC 3.tif]

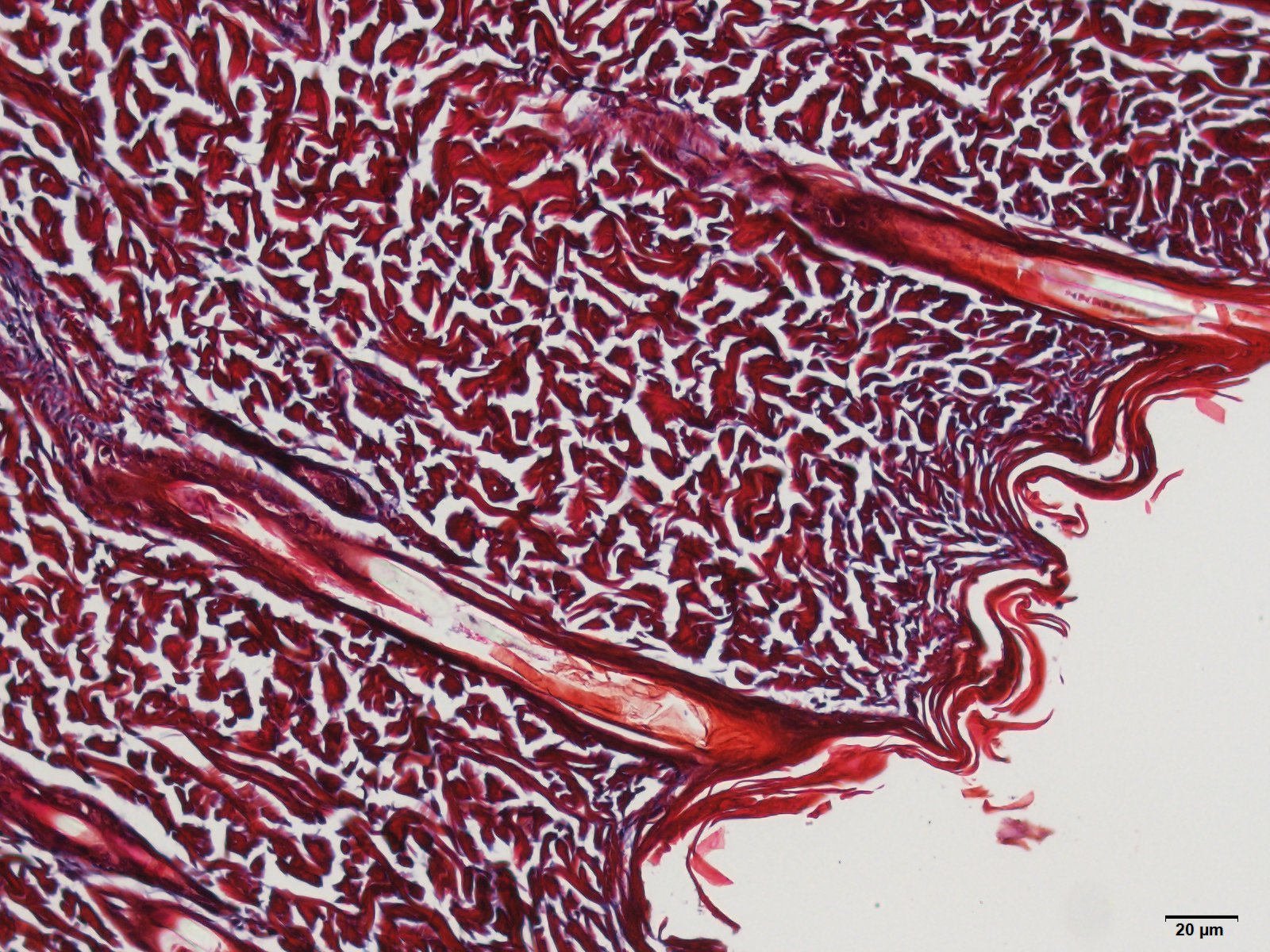

Supplement: S10 File — (ZIP) [file pone.0330078.s010.zip › Masson staning/14d CGF 1.jpg]

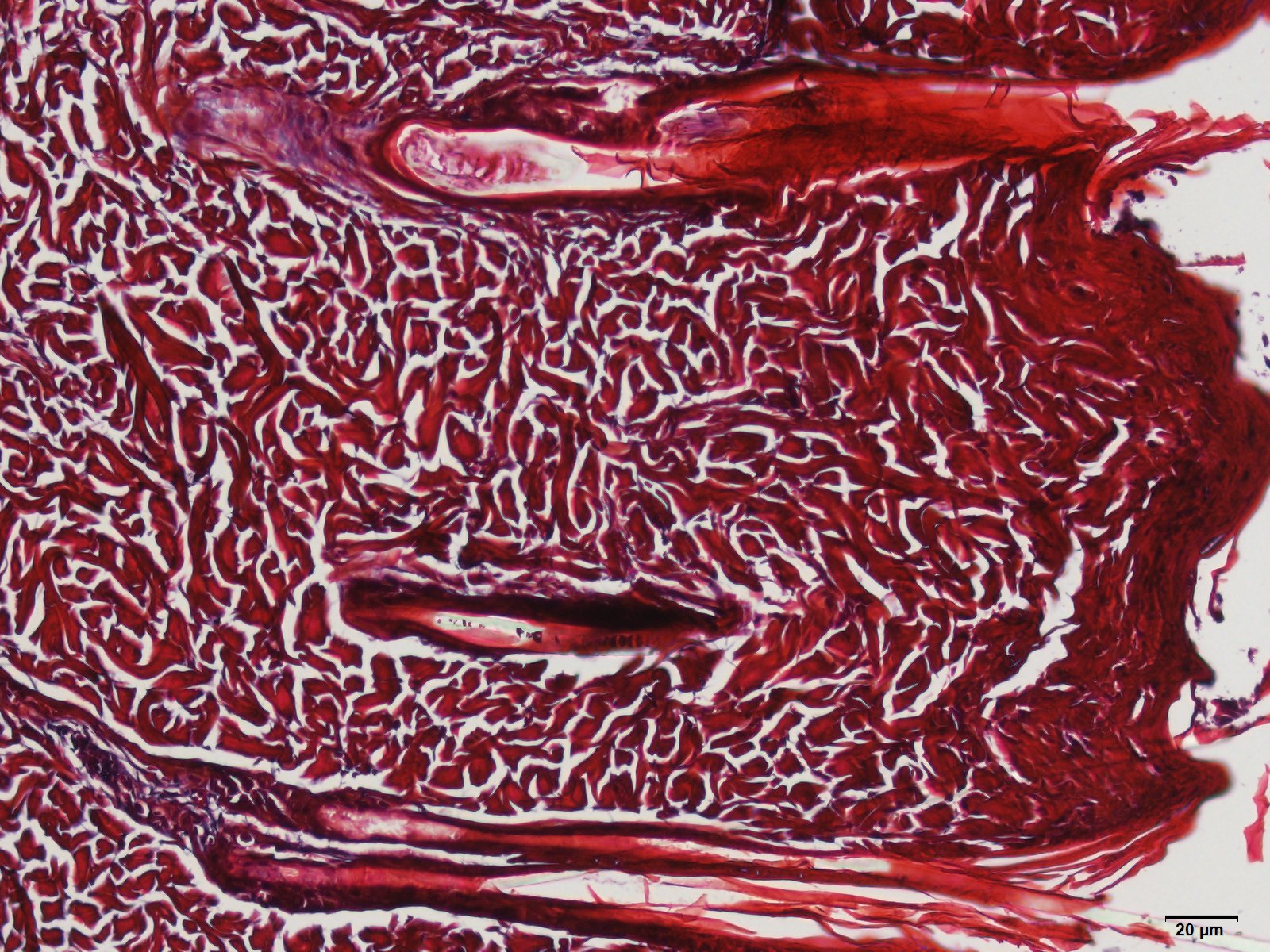

Supplement: S10 File — (ZIP) [file pone.0330078.s010.zip › Masson staning/14d CGF 2.jpg]

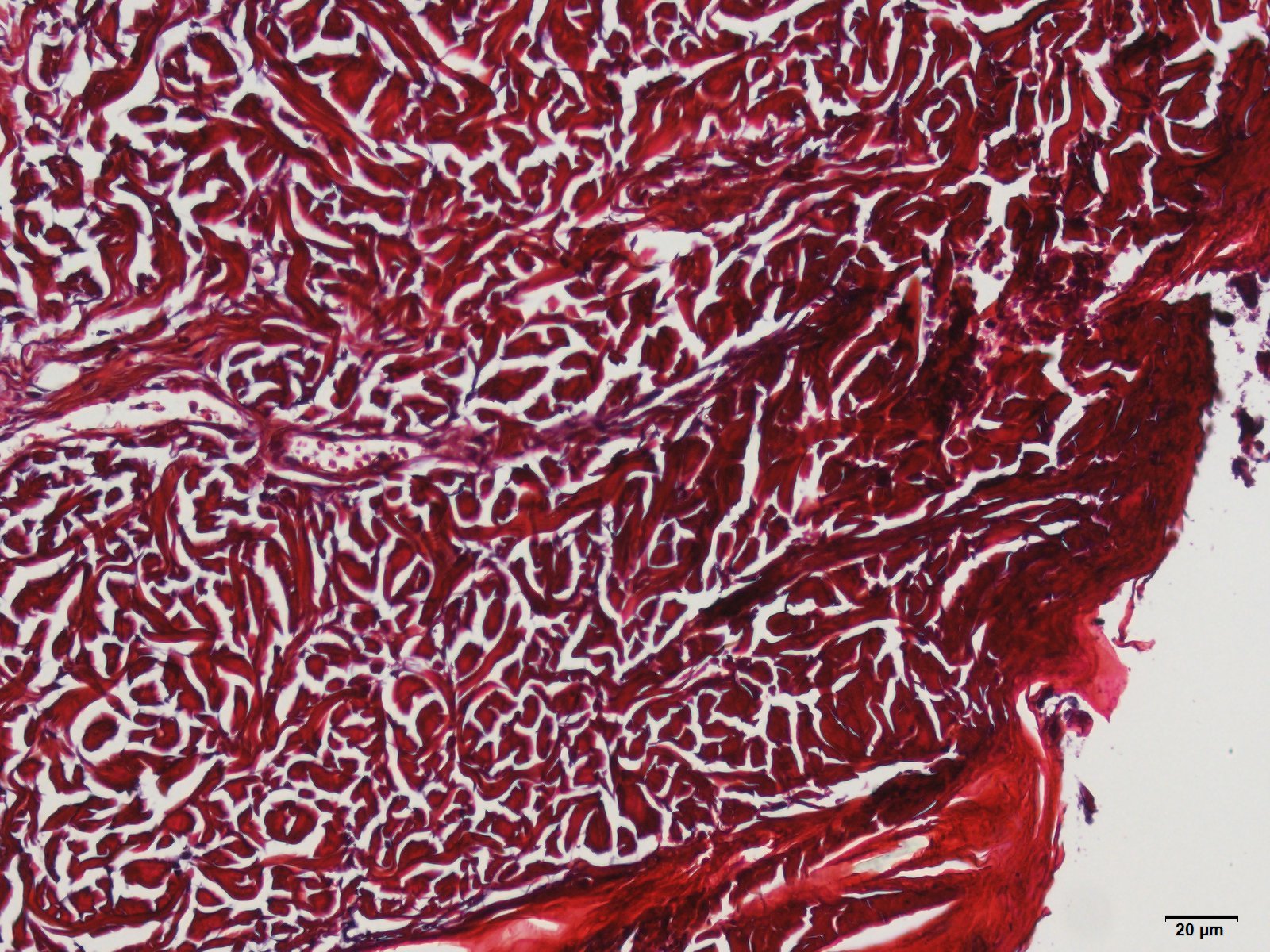

Supplement: S10 File — (ZIP) [file pone.0330078.s010.zip › Masson staning/14d CGF 3.jpg]

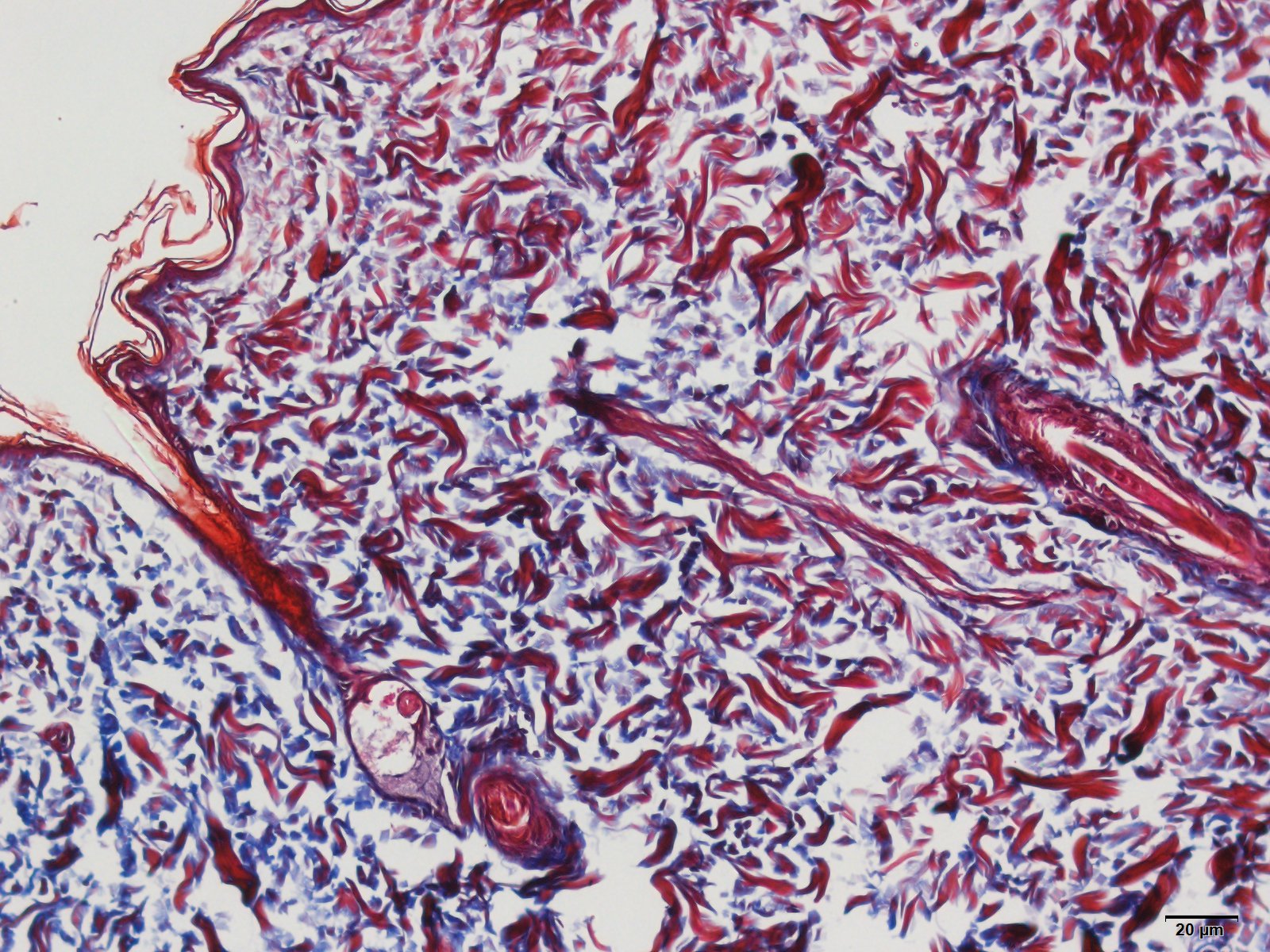

Supplement: S10 File — (ZIP) [file pone.0330078.s010.zip › Masson staning/14d CGF+HAMCC 1.jpg]

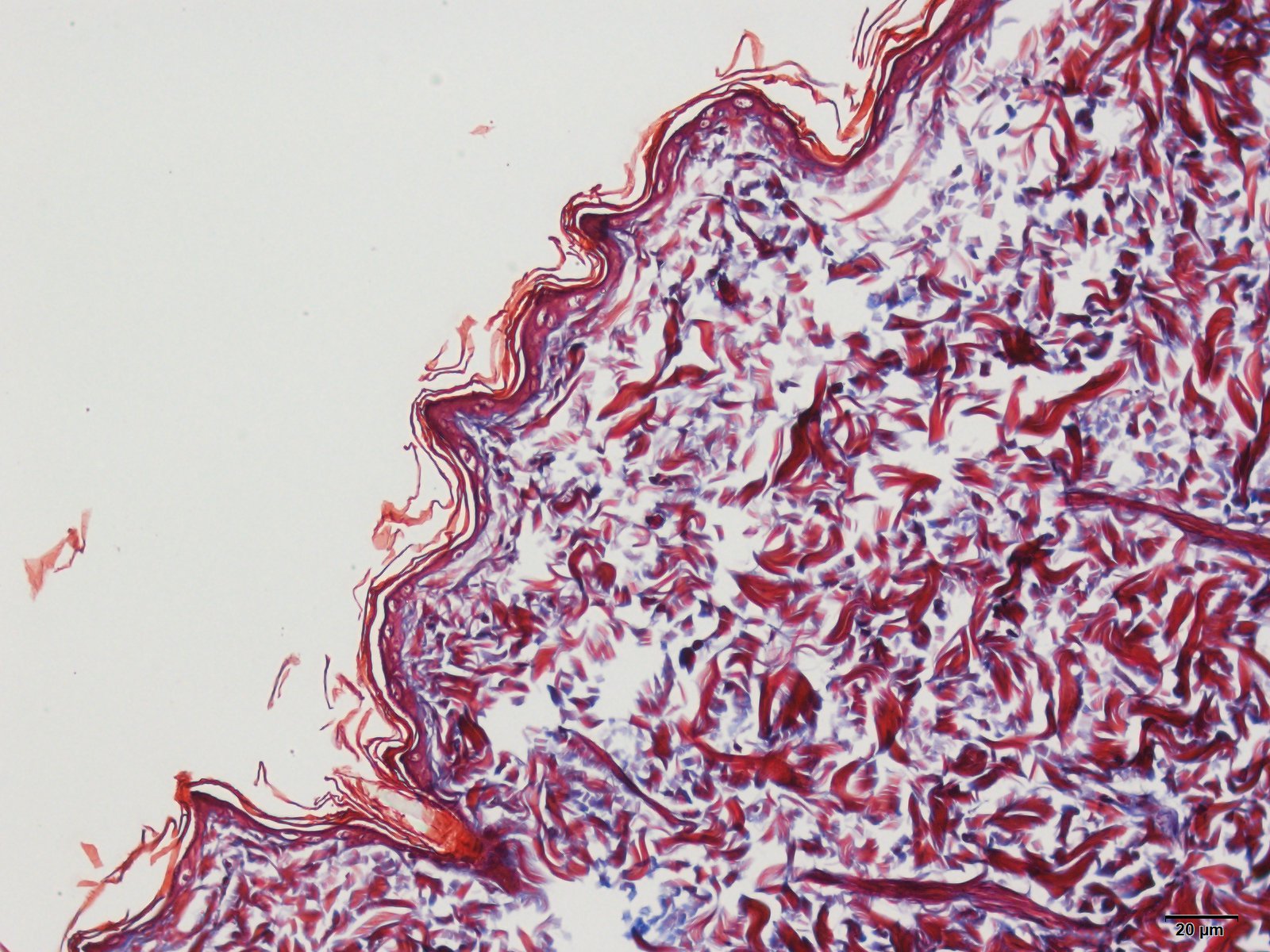

Supplement: S10 File — (ZIP) [file pone.0330078.s010.zip › Masson staning/14d CGF+HAMCC 2.jpg]

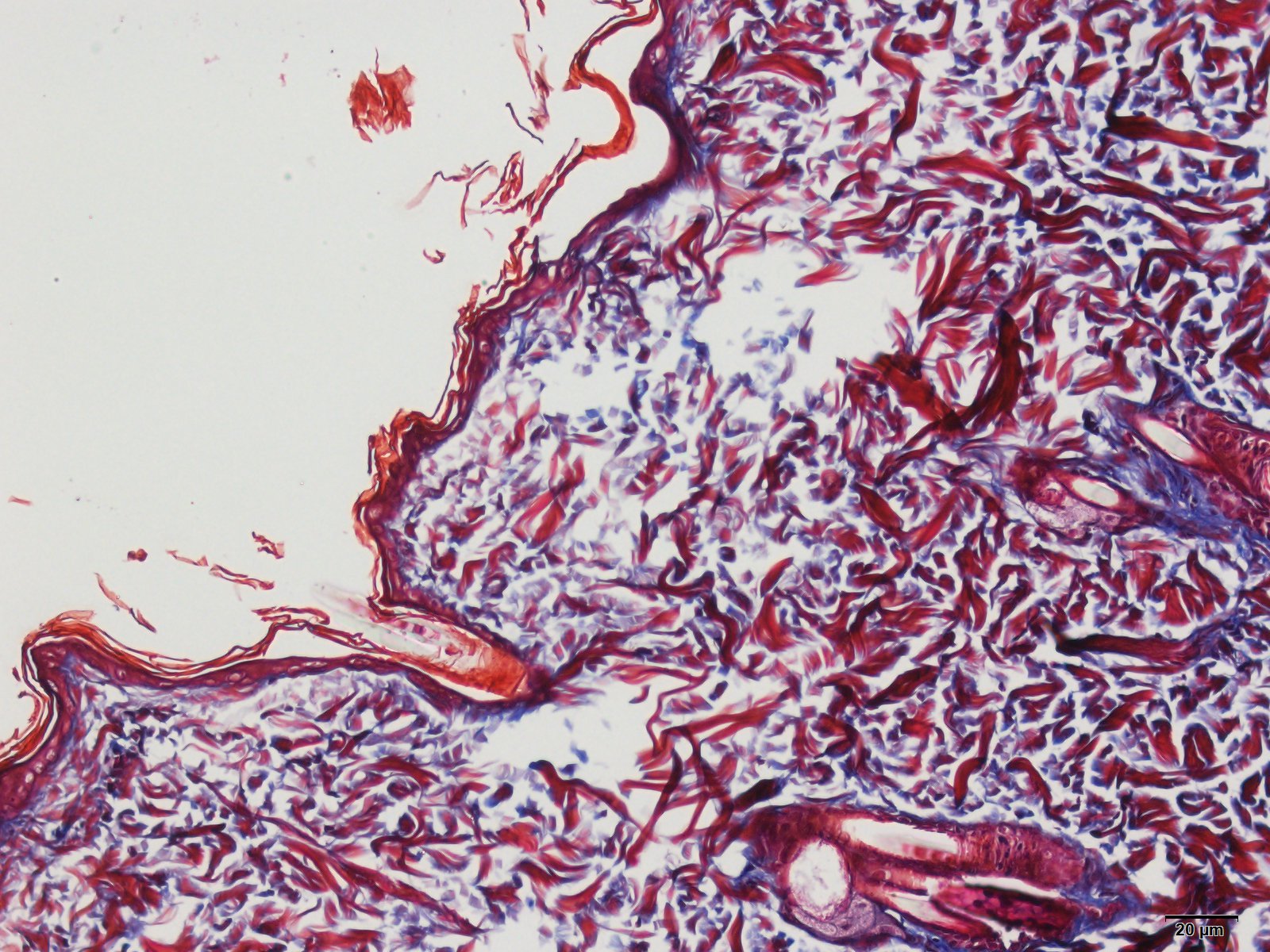

Supplement: S10 File — (ZIP) [file pone.0330078.s010.zip › Masson staning/14d CGF+HAMCC 3.jpg]

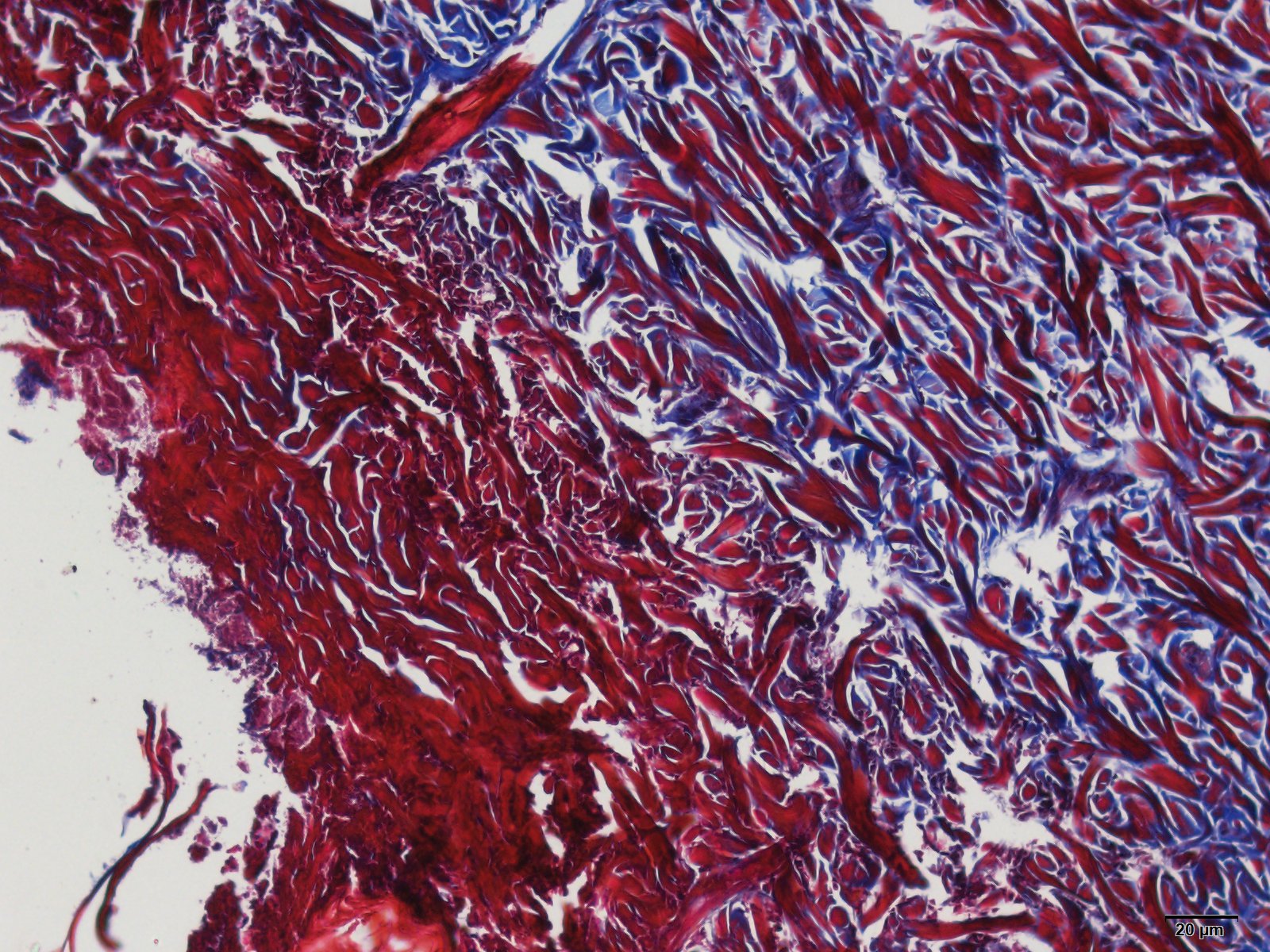

Supplement: S10 File — (ZIP) [file pone.0330078.s010.zip › Masson staning/14d Control 1.jpg]

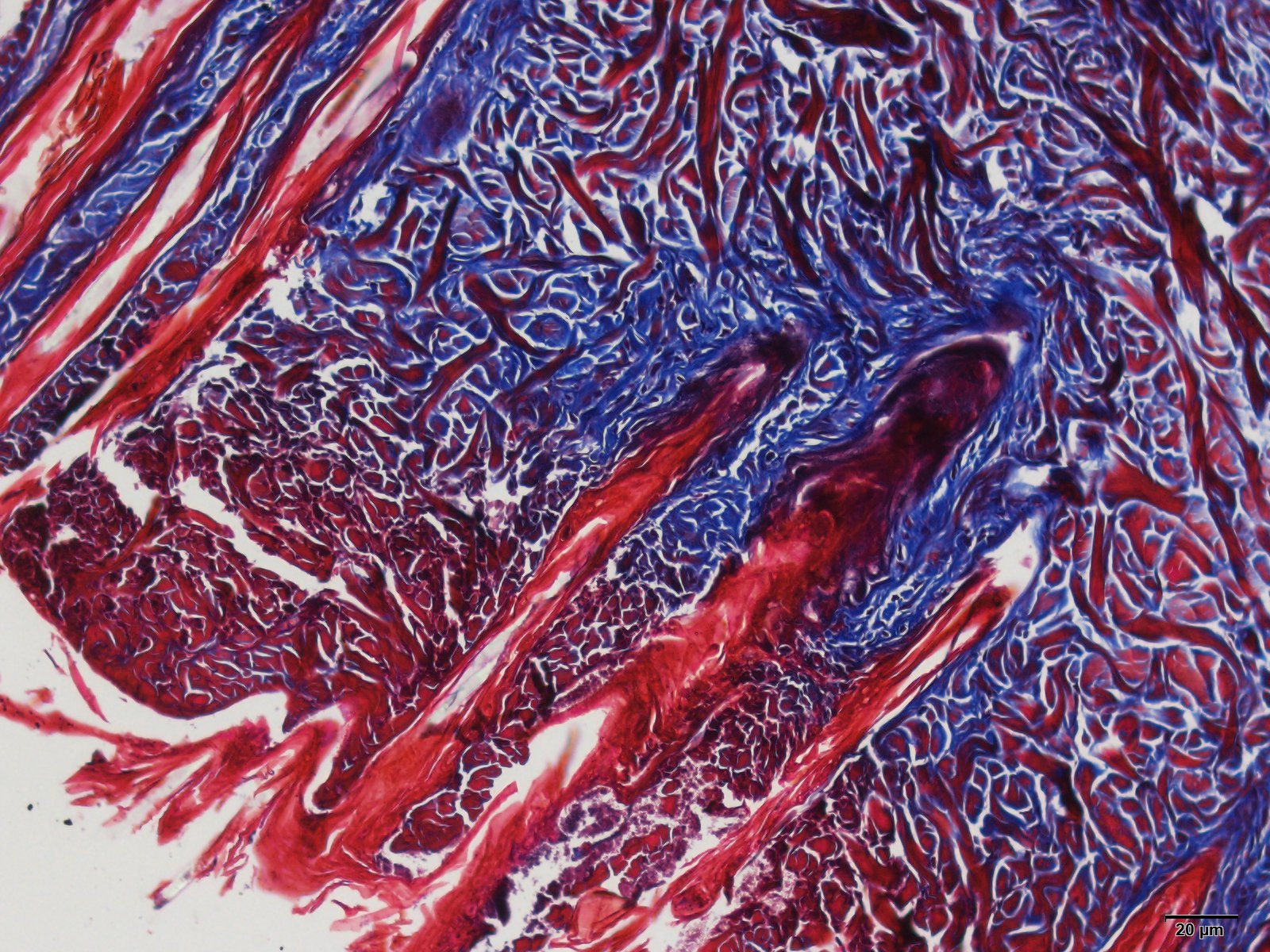

Supplement: S10 File — (ZIP) [file pone.0330078.s010.zip › Masson staning/14d Control 2.jpg]

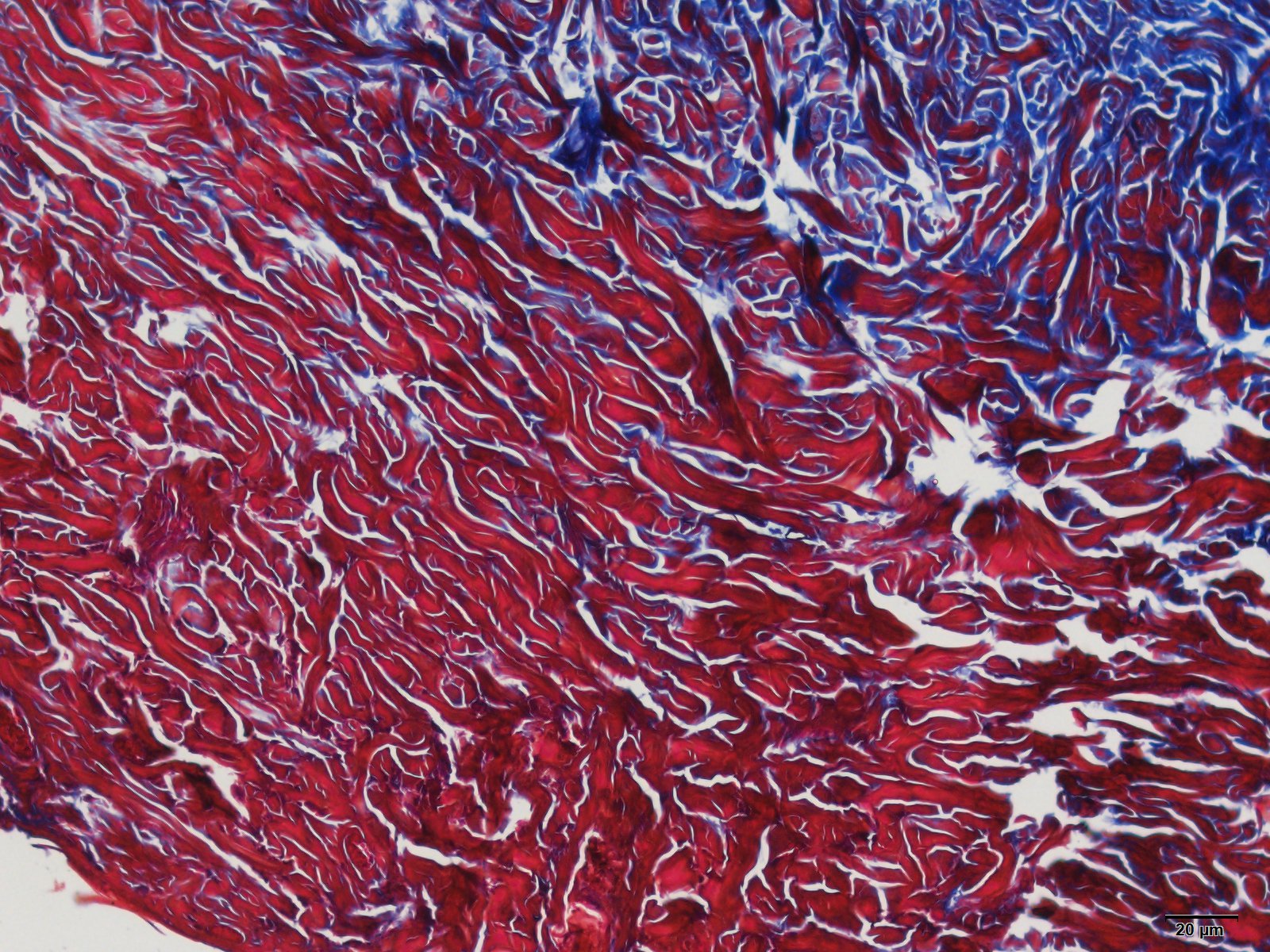

Supplement: S10 File — (ZIP) [file pone.0330078.s010.zip › Masson staning/14d Control 3.jpg]

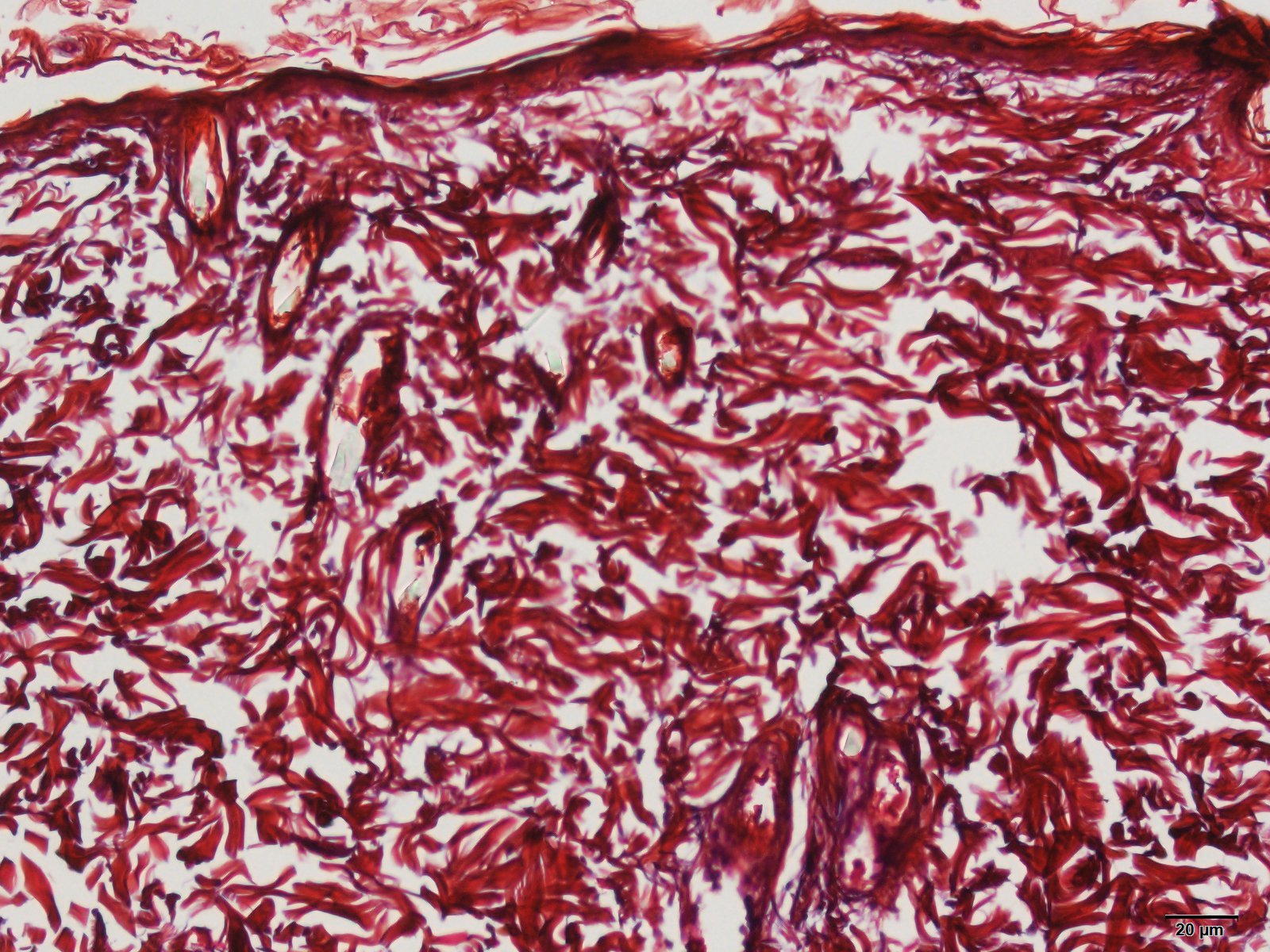

Supplement: S10 File — (ZIP) [file pone.0330078.s010.zip › Masson staning/14d HAMCC 1.jpg]

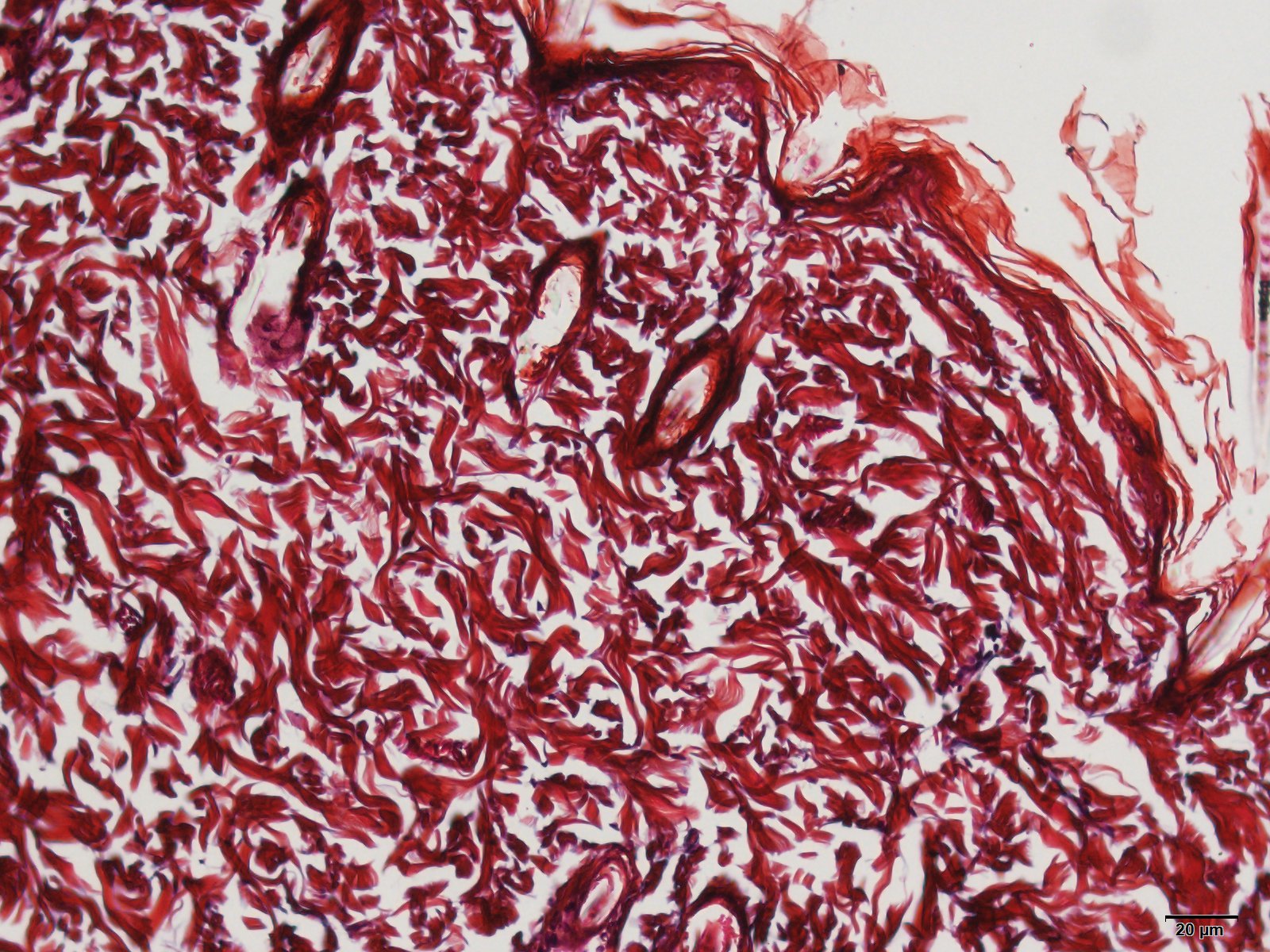

Supplement: S10 File — (ZIP) [file pone.0330078.s010.zip › Masson staning/14d HAMCC 2.jpg]

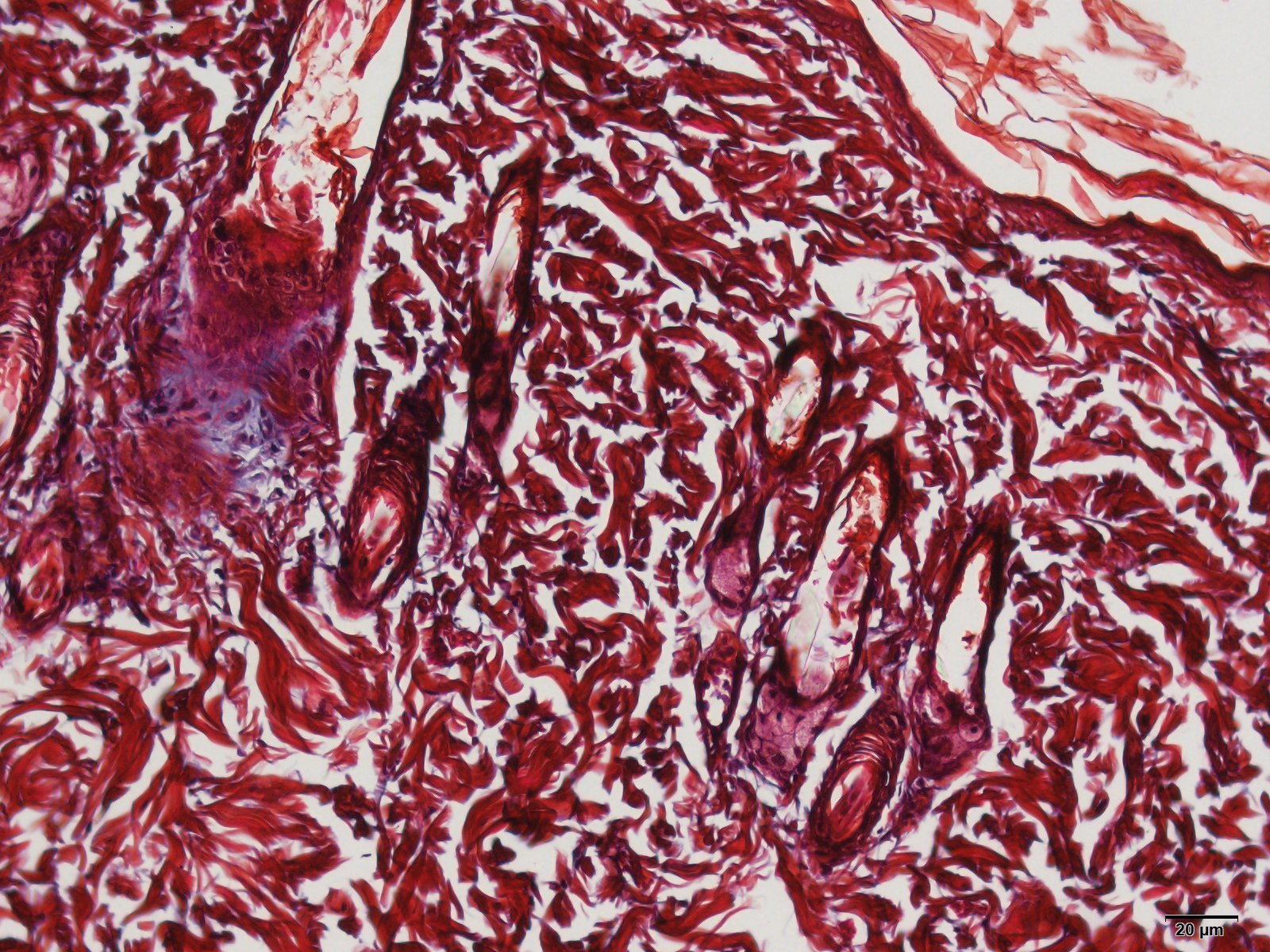

Supplement: S10 File — (ZIP) [file pone.0330078.s010.zip › Masson staning/14d HAMCC 3.jpg]

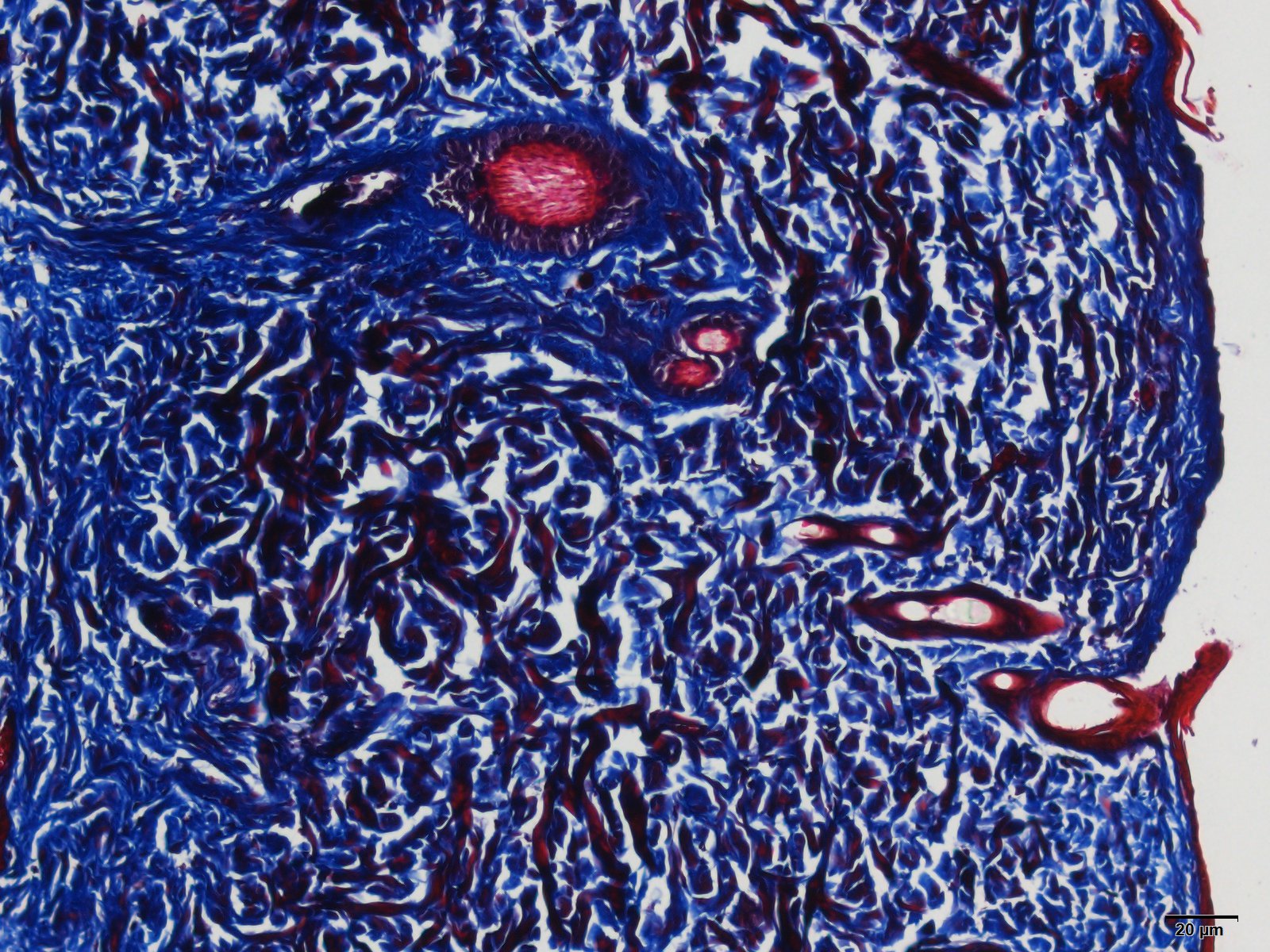

Supplement: S10 File — (ZIP) [file pone.0330078.s010.zip › Masson staning/28d CGF 1.jpg]

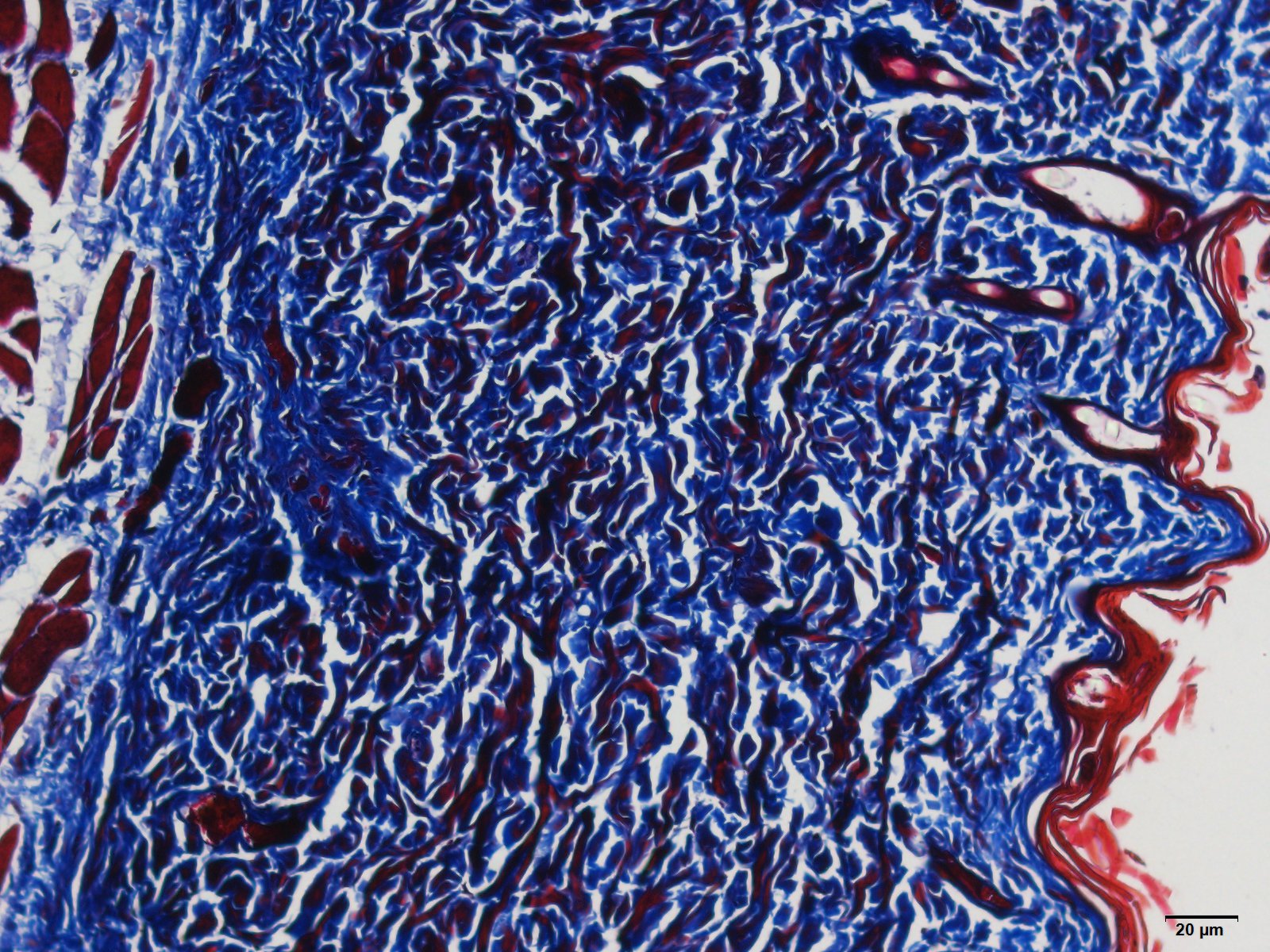

Supplement: S10 File — (ZIP) [file pone.0330078.s010.zip › Masson staning/28d CGF 2.jpg]

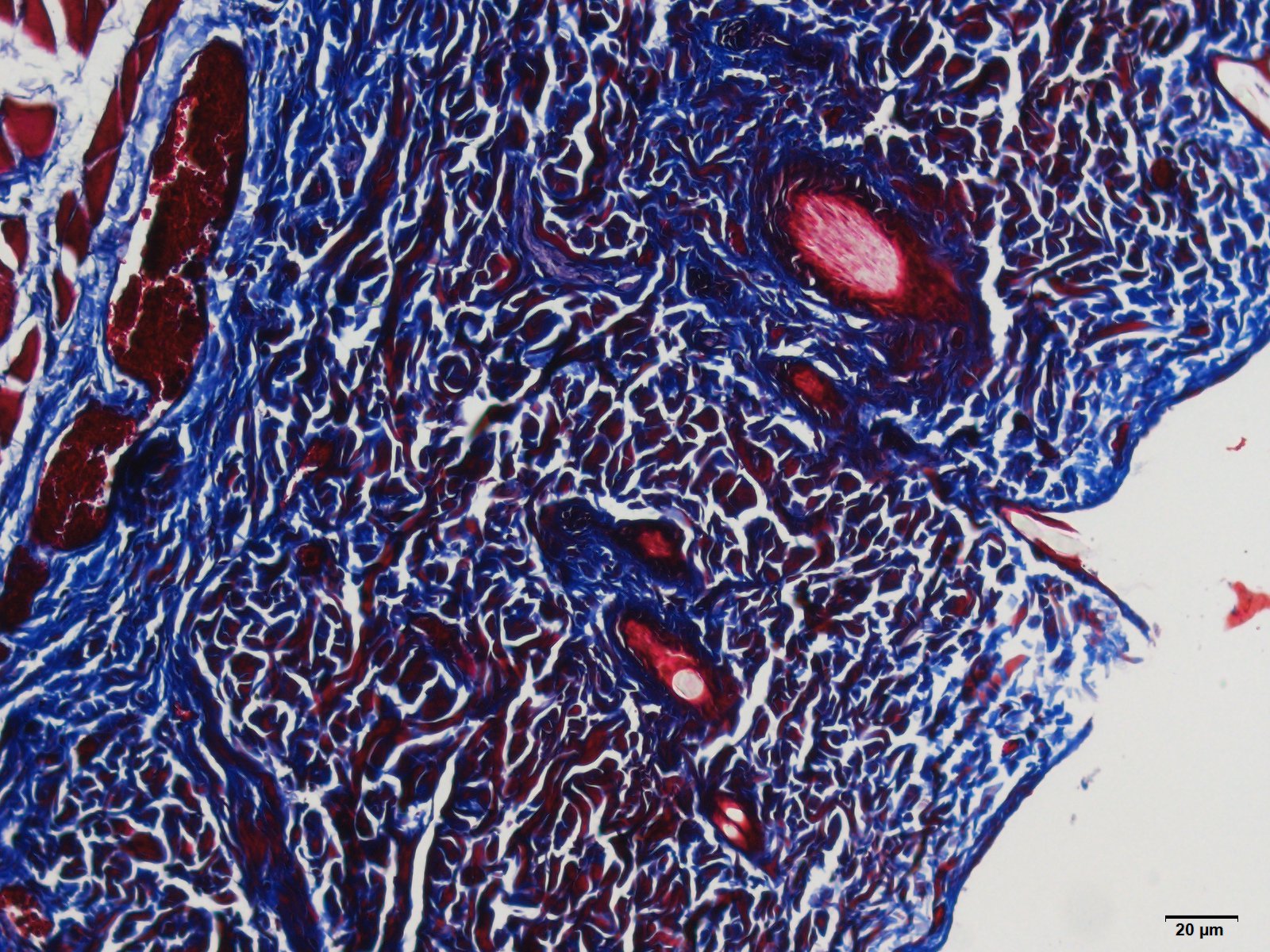

Supplement: S10 File — (ZIP) [file pone.0330078.s010.zip › Masson staning/28d CGF 3.jpg]

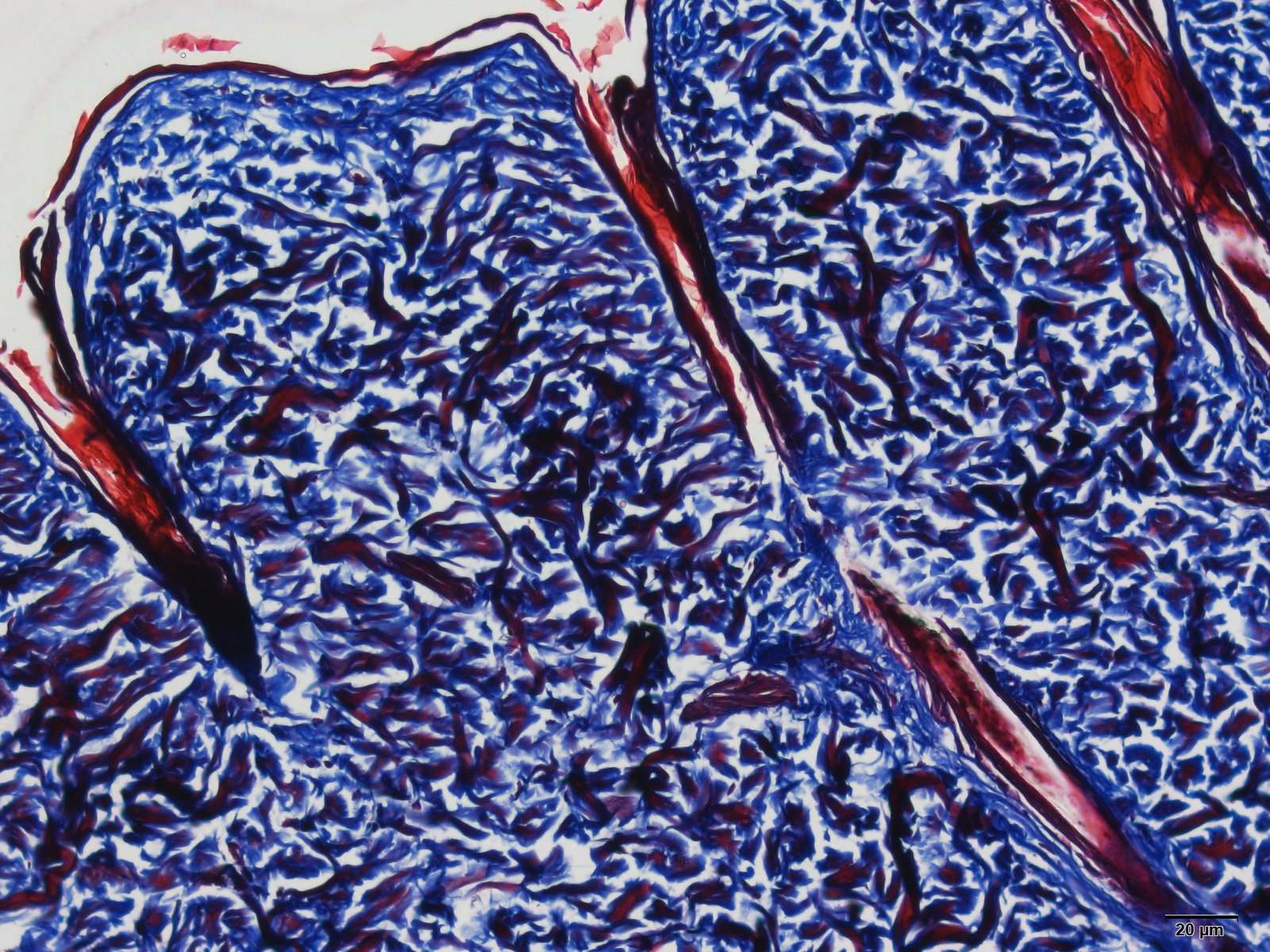

Supplement: S10 File — (ZIP) [file pone.0330078.s010.zip › Masson staning/28d CGF+HAMCC 1.jpg]

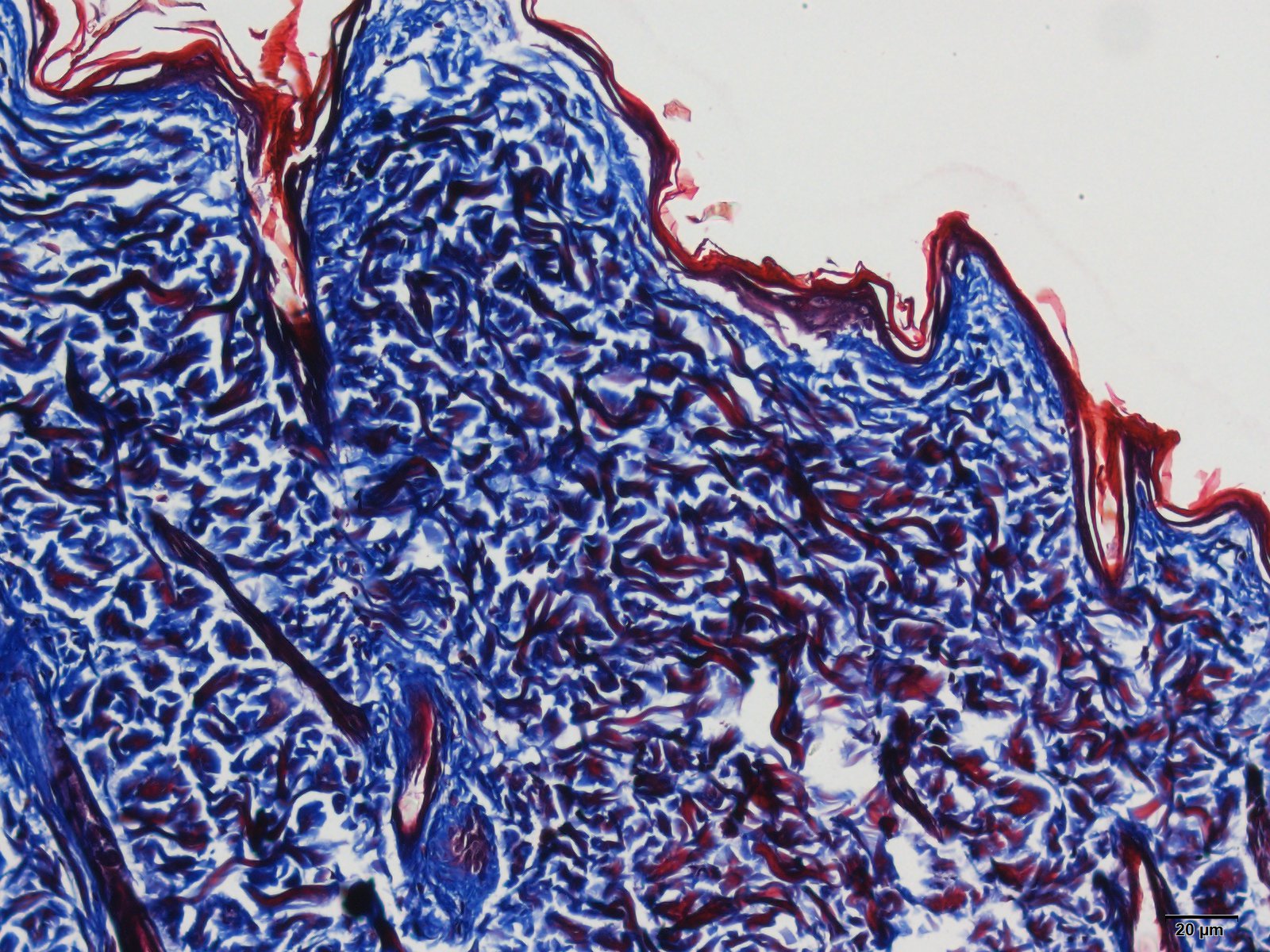

Supplement: S10 File — (ZIP) [file pone.0330078.s010.zip › Masson staning/28d CGF+HAMCC 2.jpg]

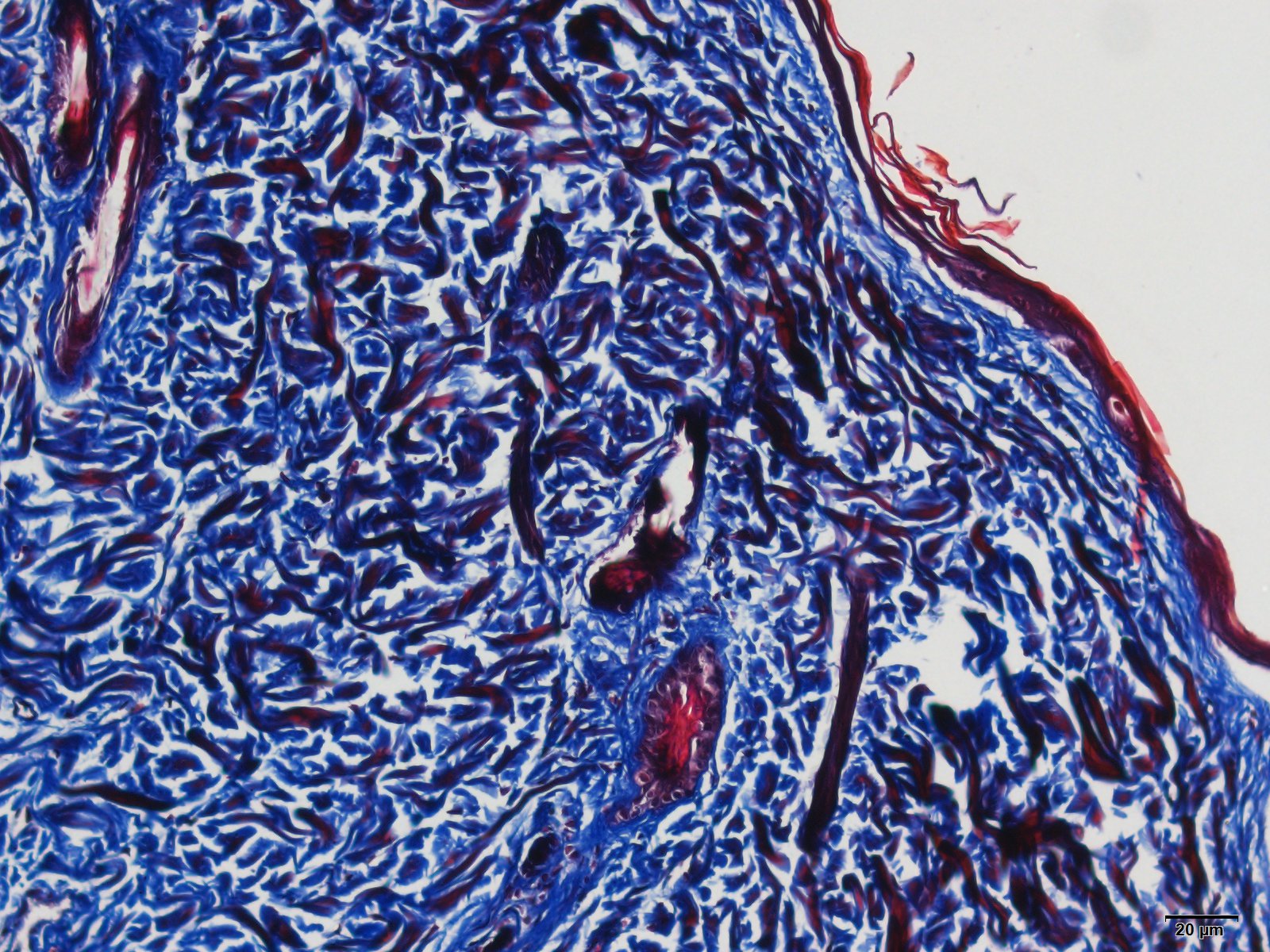

Supplement: S10 File — (ZIP) [file pone.0330078.s010.zip › Masson staning/28d CGF+HAMCC 3.jpg]

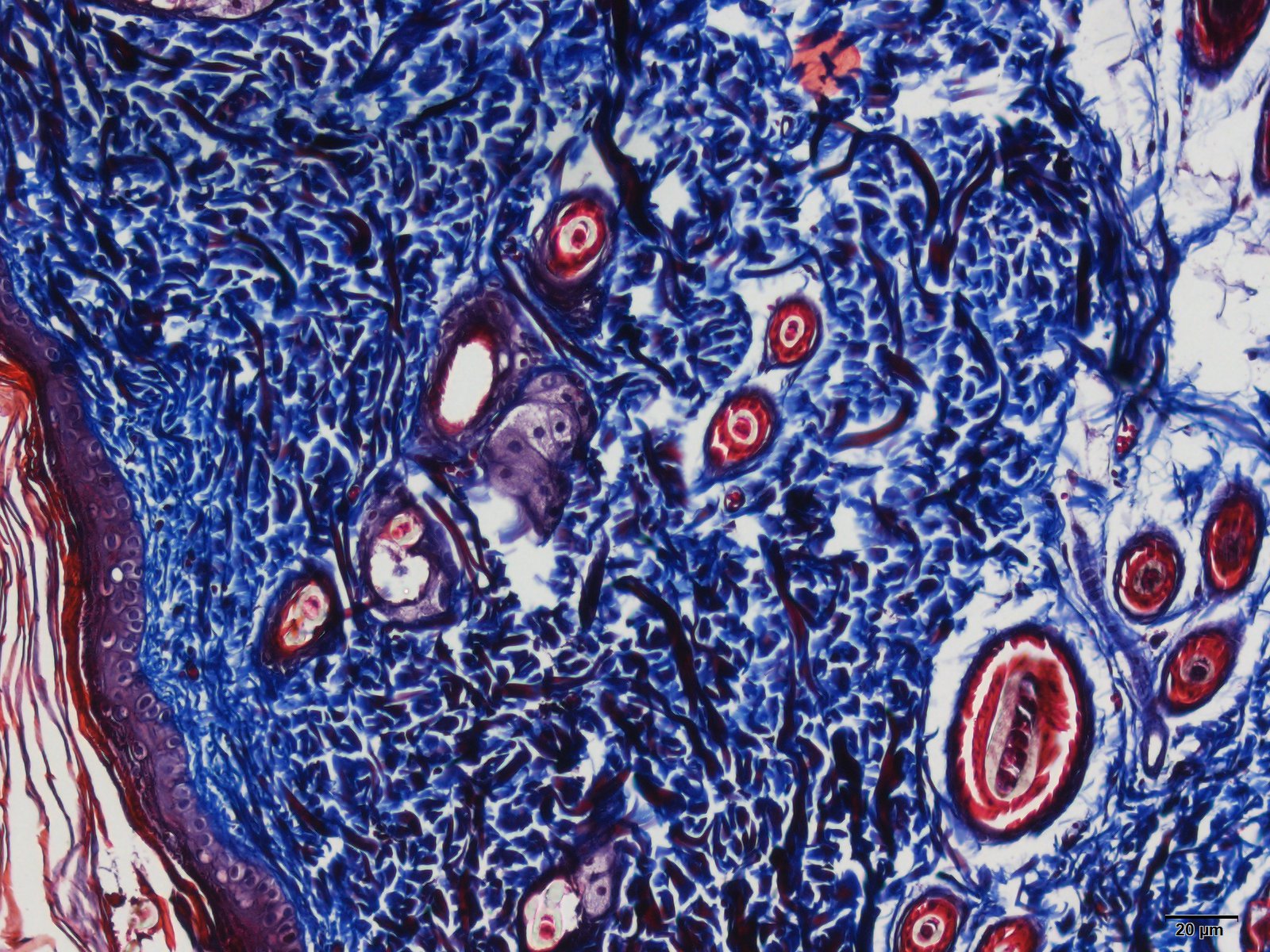

Supplement: S10 File — (ZIP) [file pone.0330078.s010.zip › Masson staning/28d Control 1.jpg]

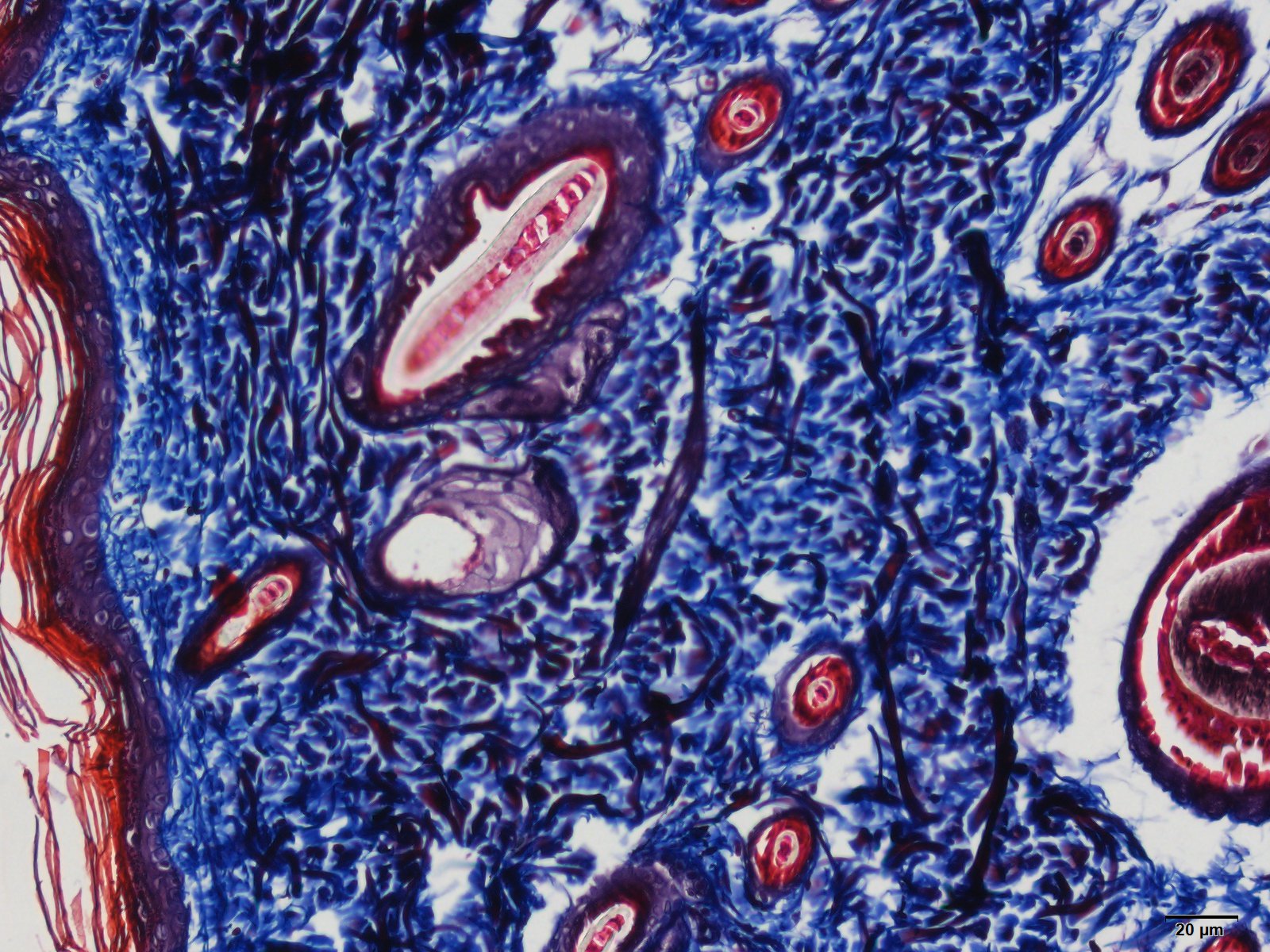

Supplement: S10 File — (ZIP) [file pone.0330078.s010.zip › Masson staning/28d Control 2.jpg]

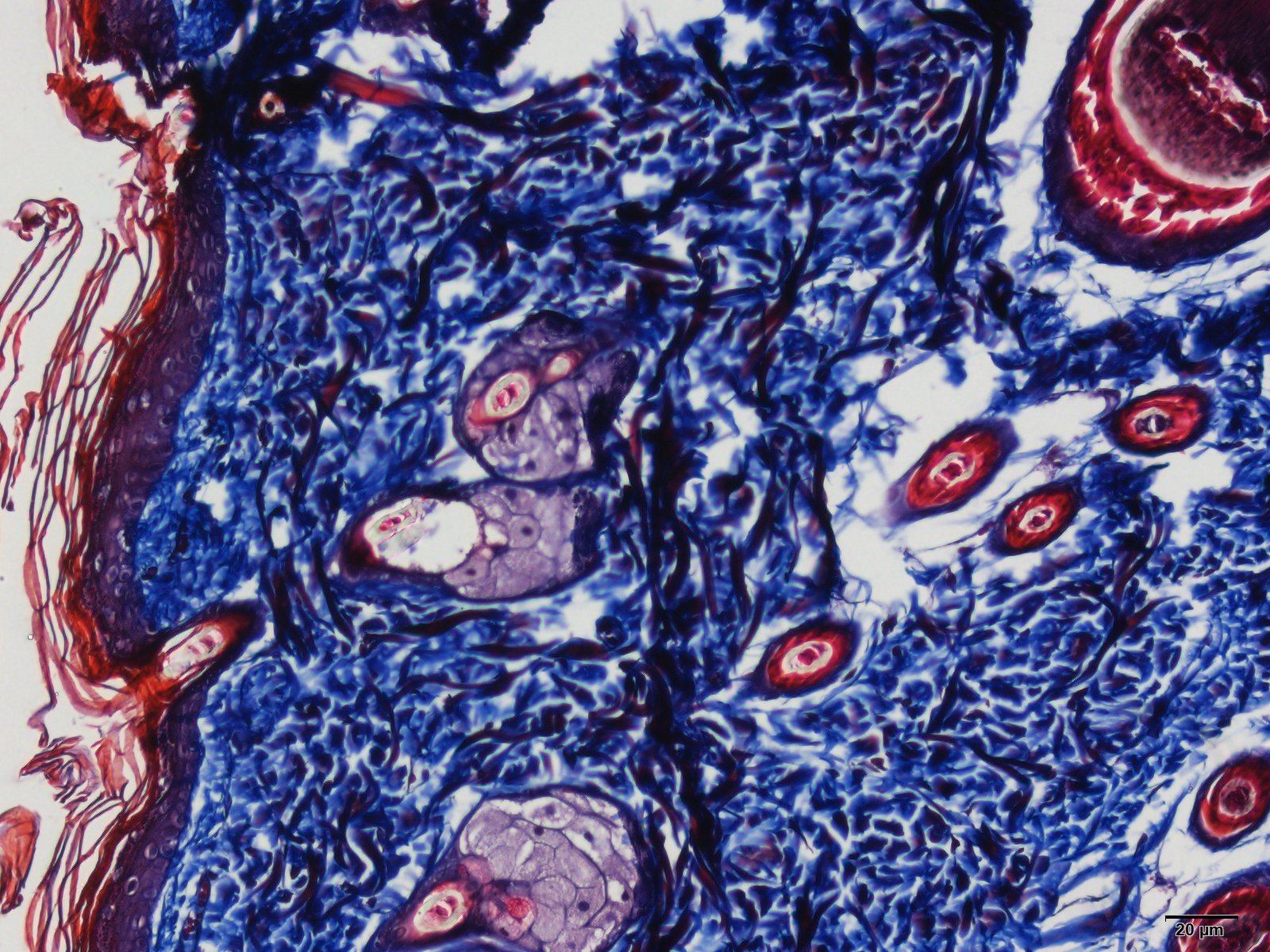

Supplement: S10 File — (ZIP) [file pone.0330078.s010.zip › Masson staning/28d Control 3.jpg]

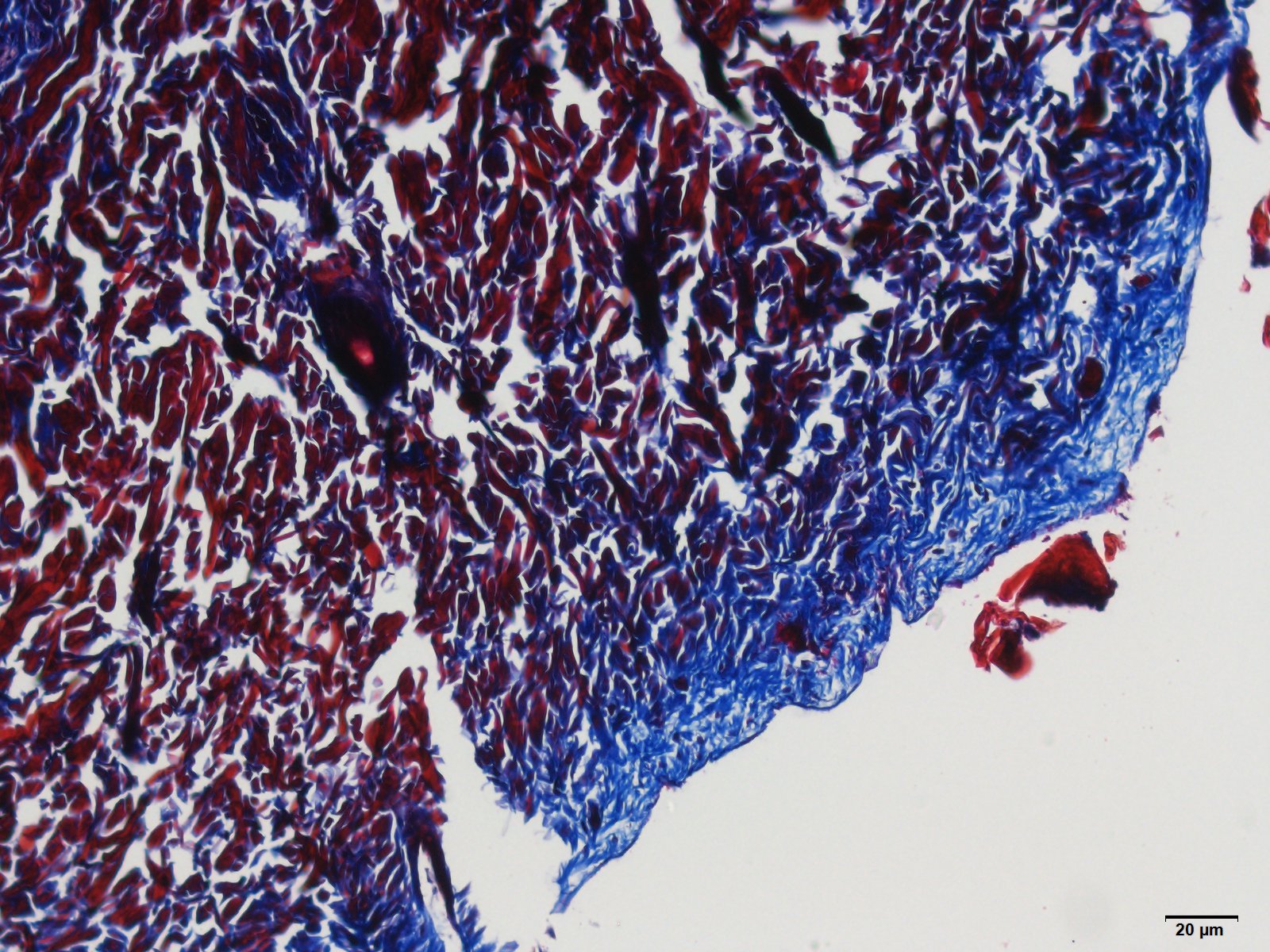

Supplement: S10 File — (ZIP) [file pone.0330078.s010.zip › Masson staning/28d HAMCC 1.jpg]

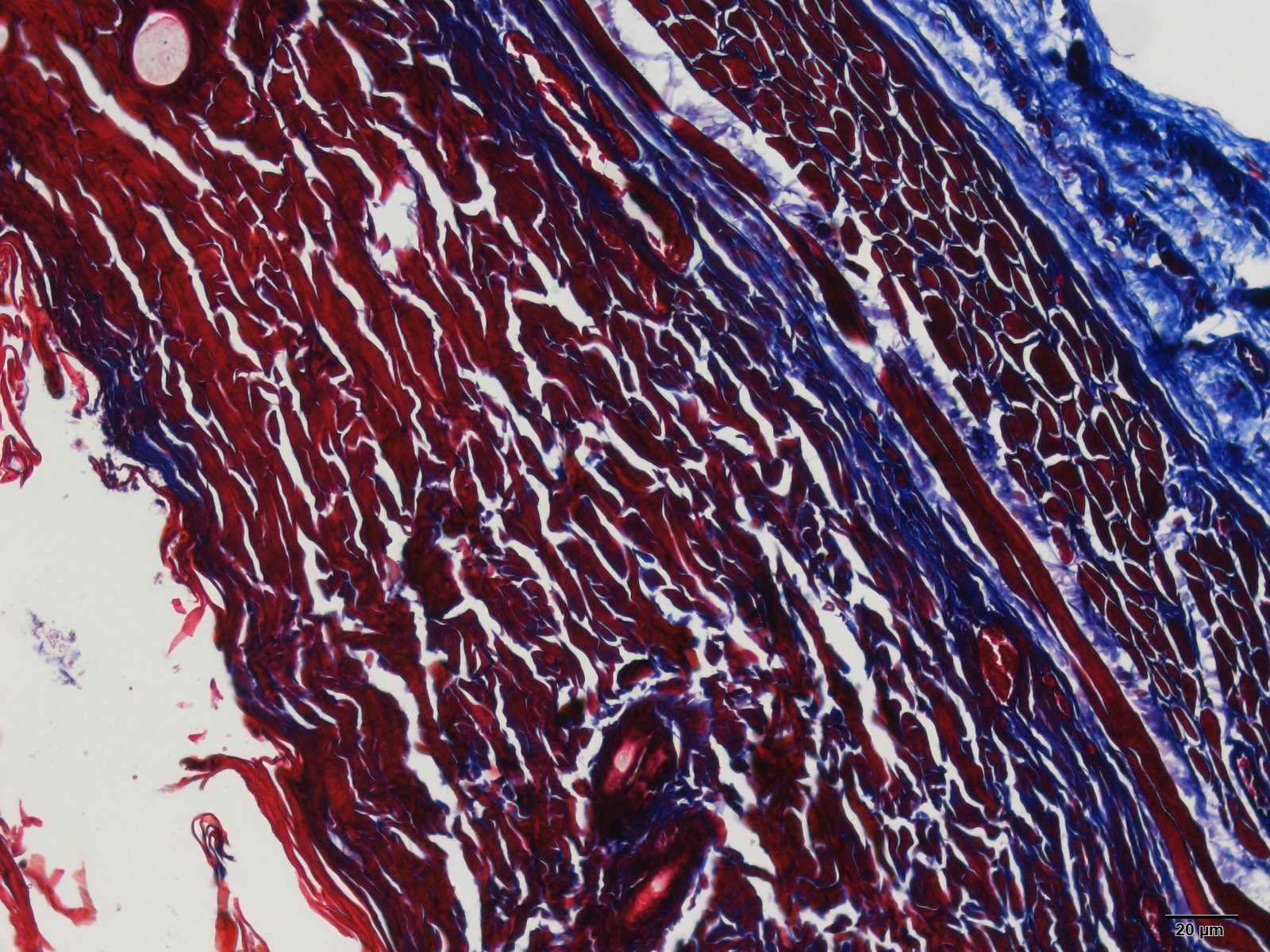

Supplement: S10 File — (ZIP) [file pone.0330078.s010.zip › Masson staning/28d HAMCC 2.jpg]

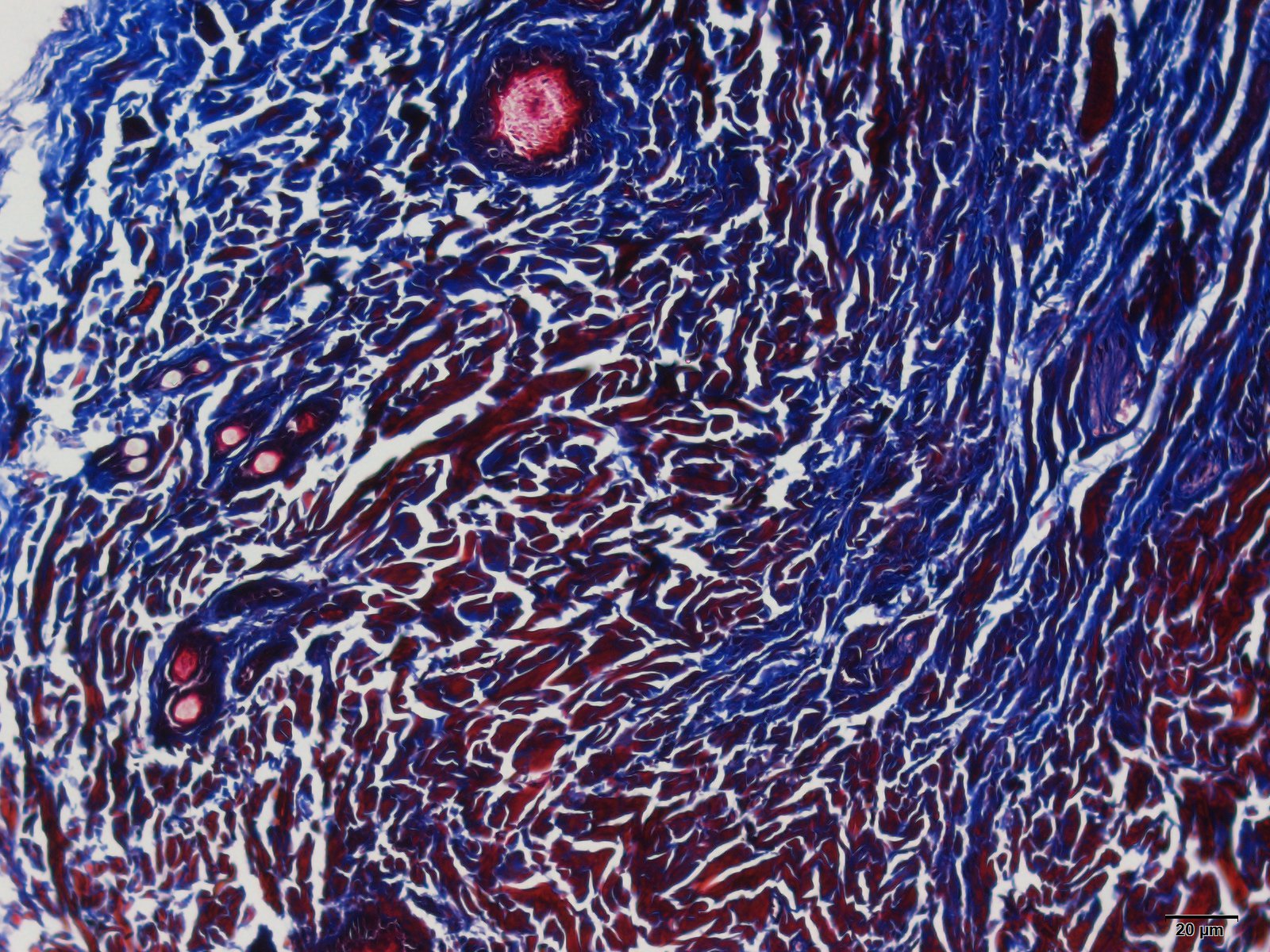

Supplement: S10 File — (ZIP) [file pone.0330078.s010.zip › Masson staning/28d HAMCC 3.jpg]

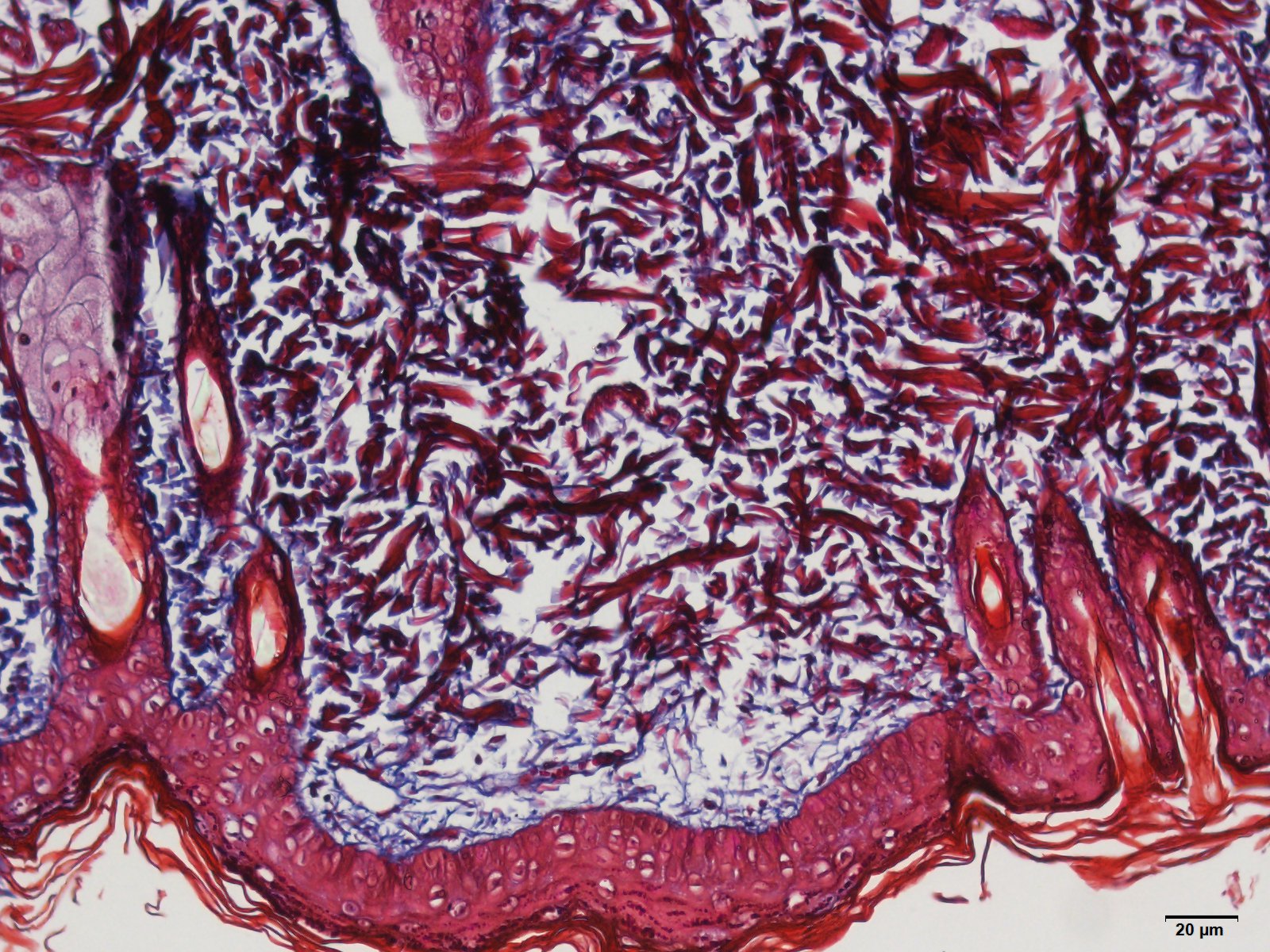

Supplement: S10 File — (ZIP) [file pone.0330078.s010.zip › Masson staning/7d CGF 1.jpg]

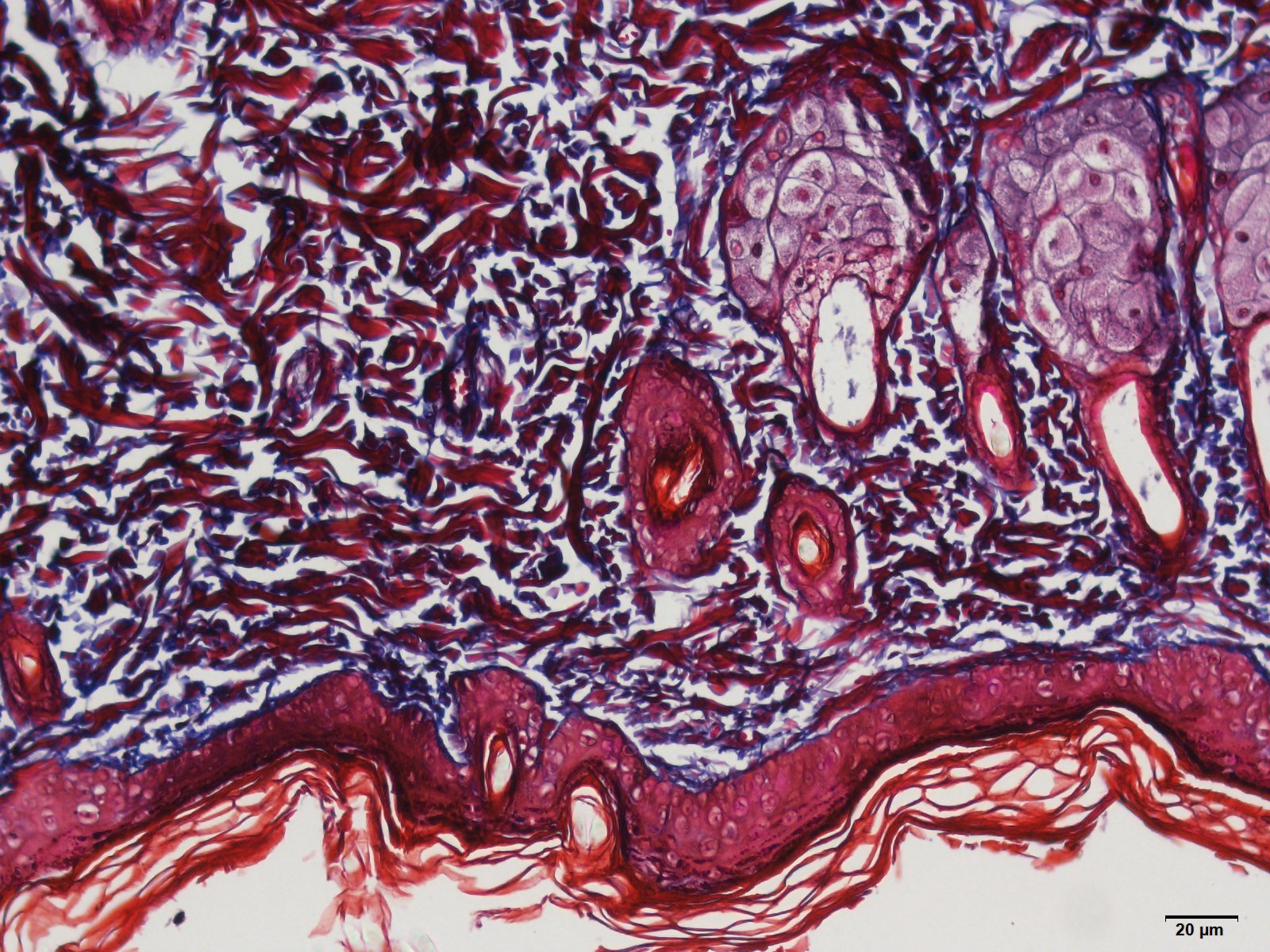

Supplement: S10 File — (ZIP) [file pone.0330078.s010.zip › Masson staning/7d CGF 2.jpg]

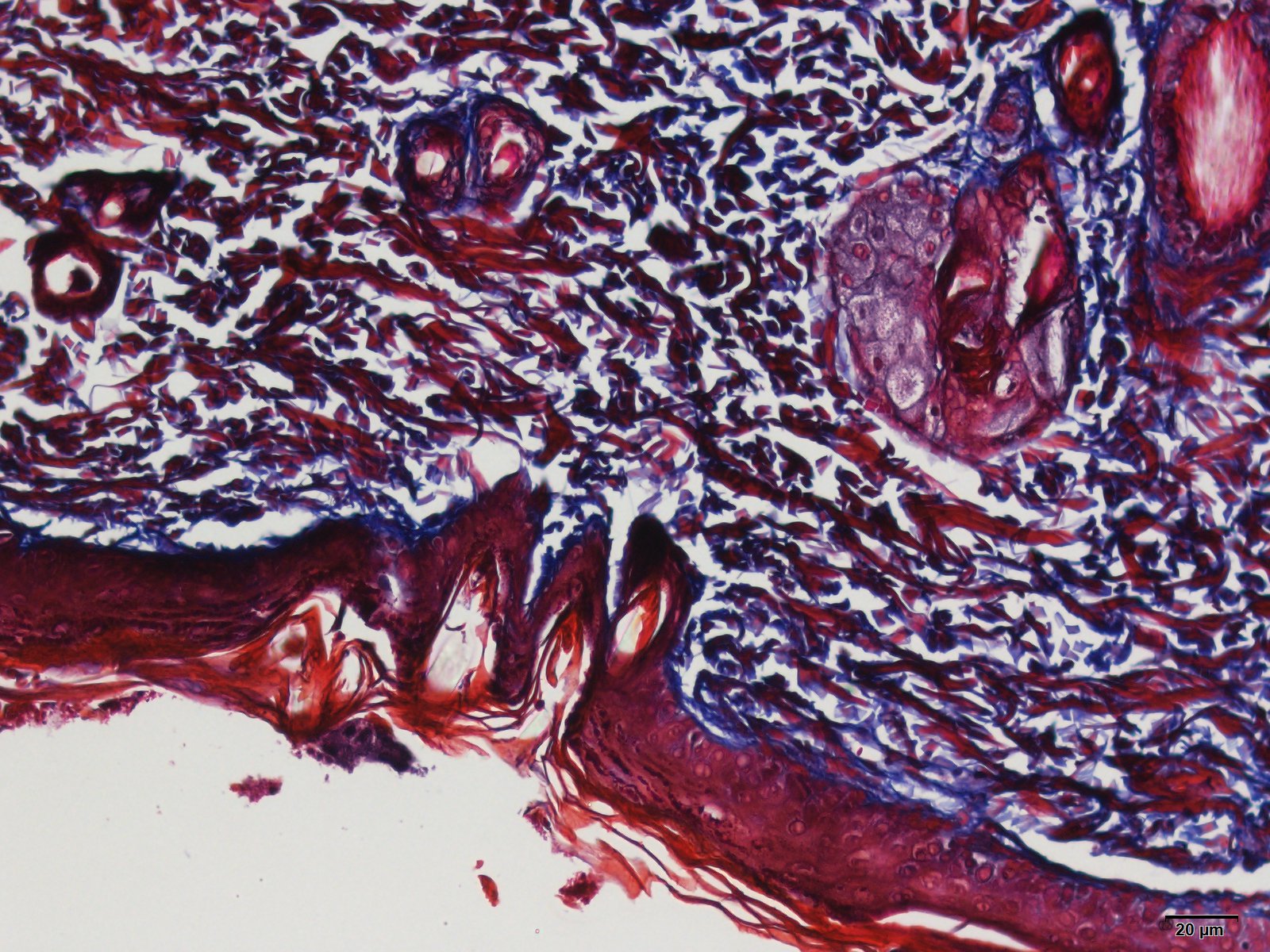

Supplement: S10 File — (ZIP) [file pone.0330078.s010.zip › Masson staning/7d CGF 3.jpg]

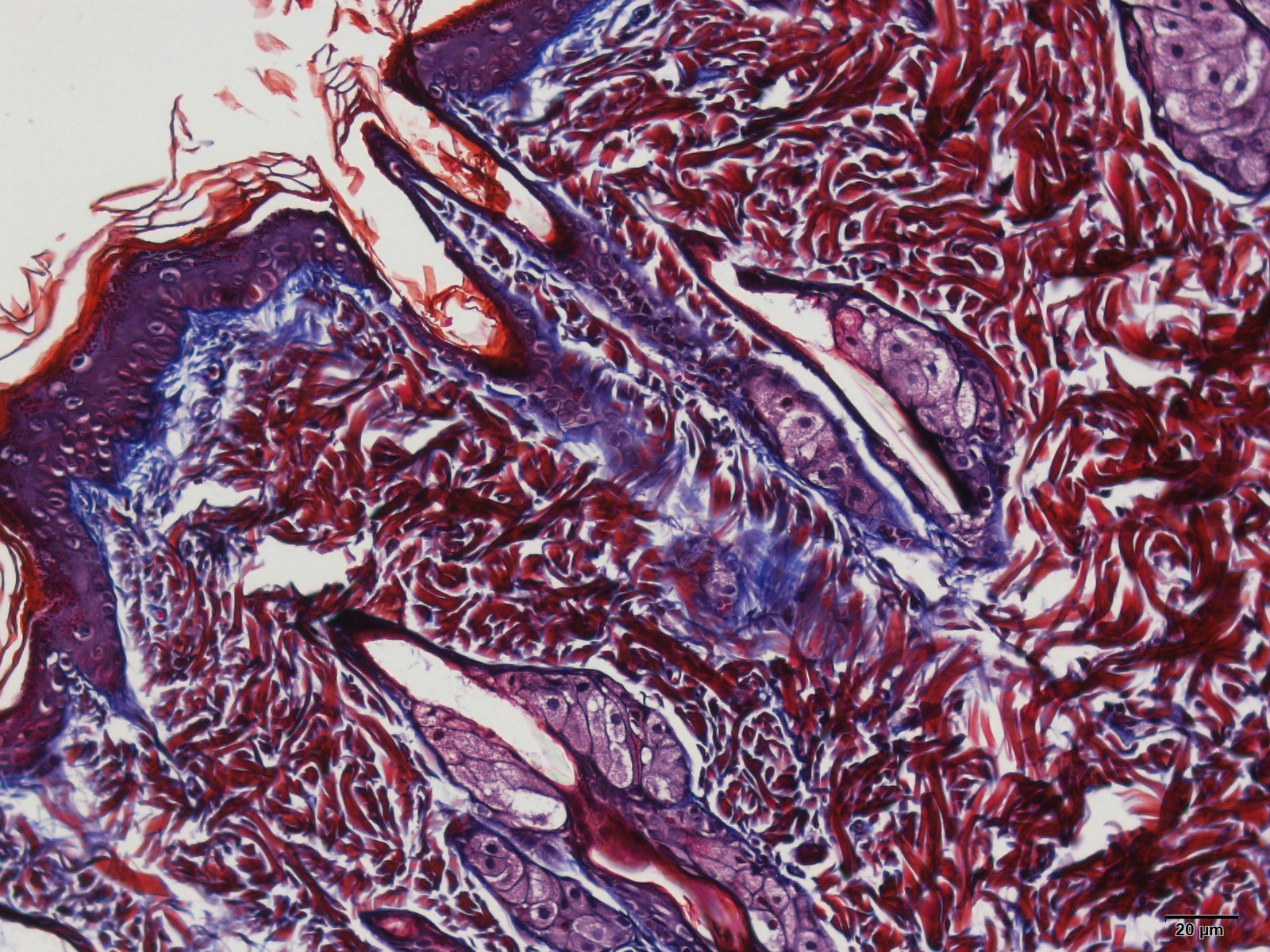

Supplement: S10 File — (ZIP) [file pone.0330078.s010.zip › Masson staning/7d CGF+HAMCC 1.jpg]

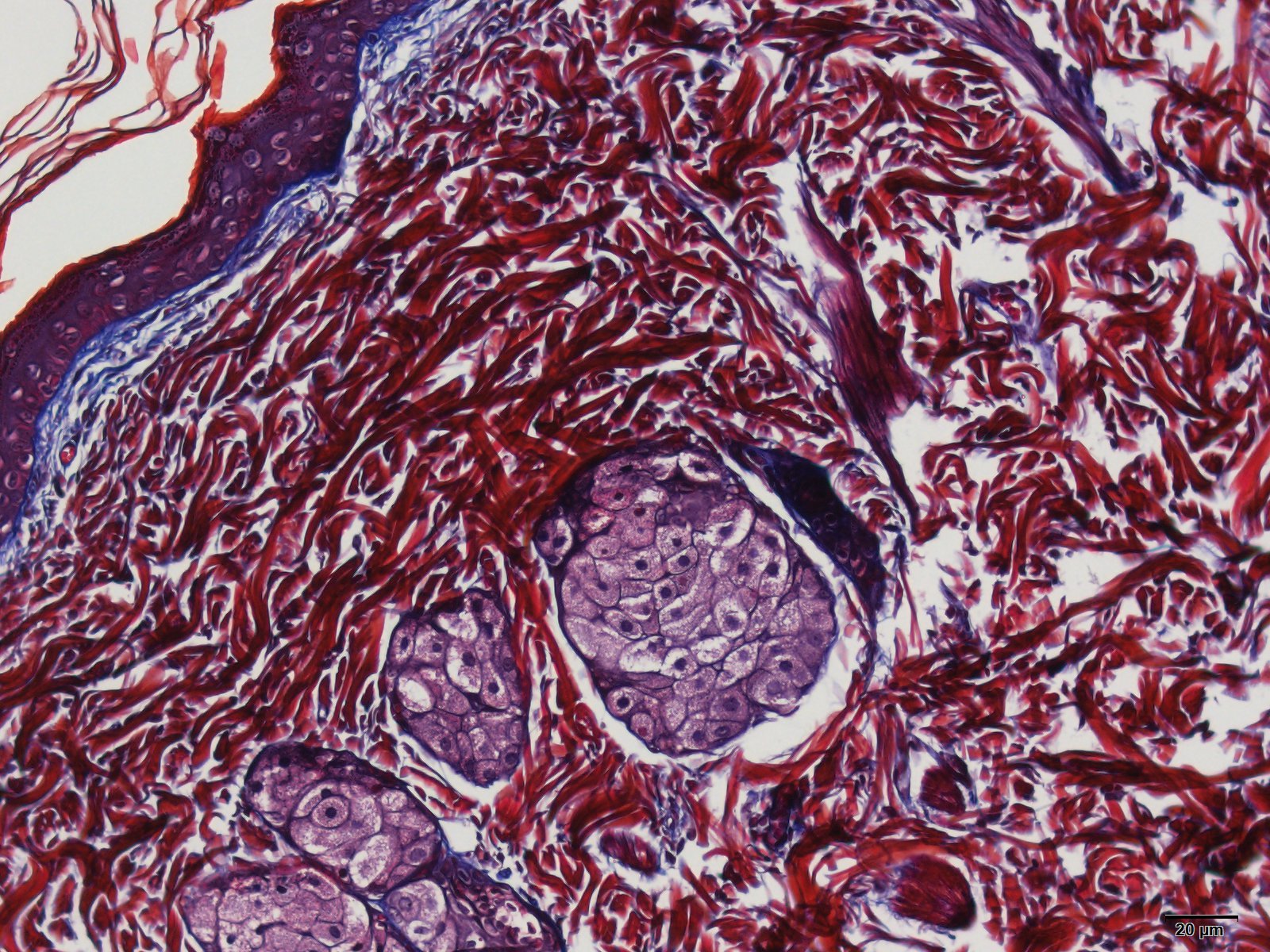

Supplement: S10 File — (ZIP) [file pone.0330078.s010.zip › Masson staning/7d CGF+HAMCC 2.jpg]

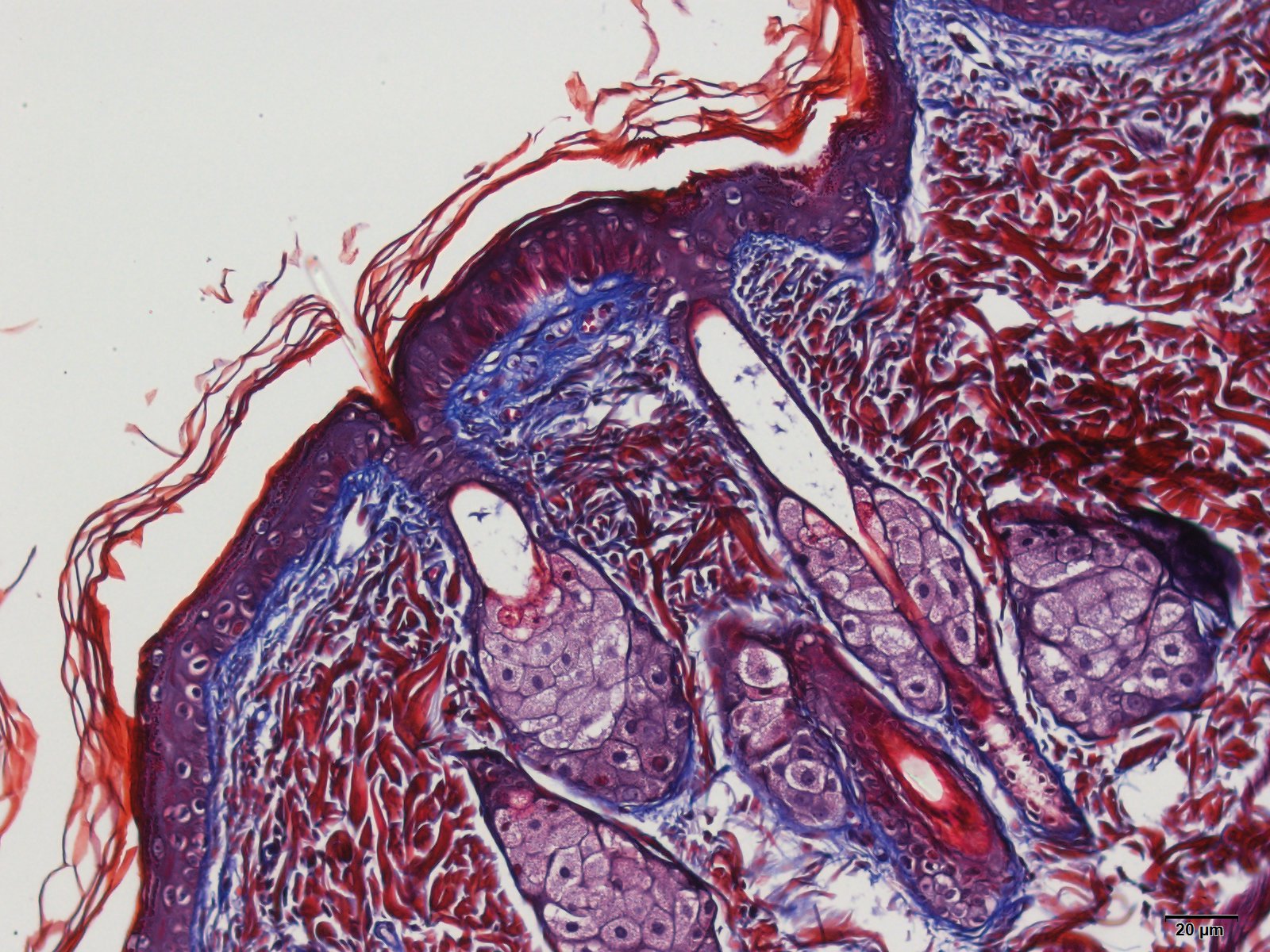

Supplement: S10 File — (ZIP) [file pone.0330078.s010.zip › Masson staning/7d CGF+HAMCC 3.jpg]

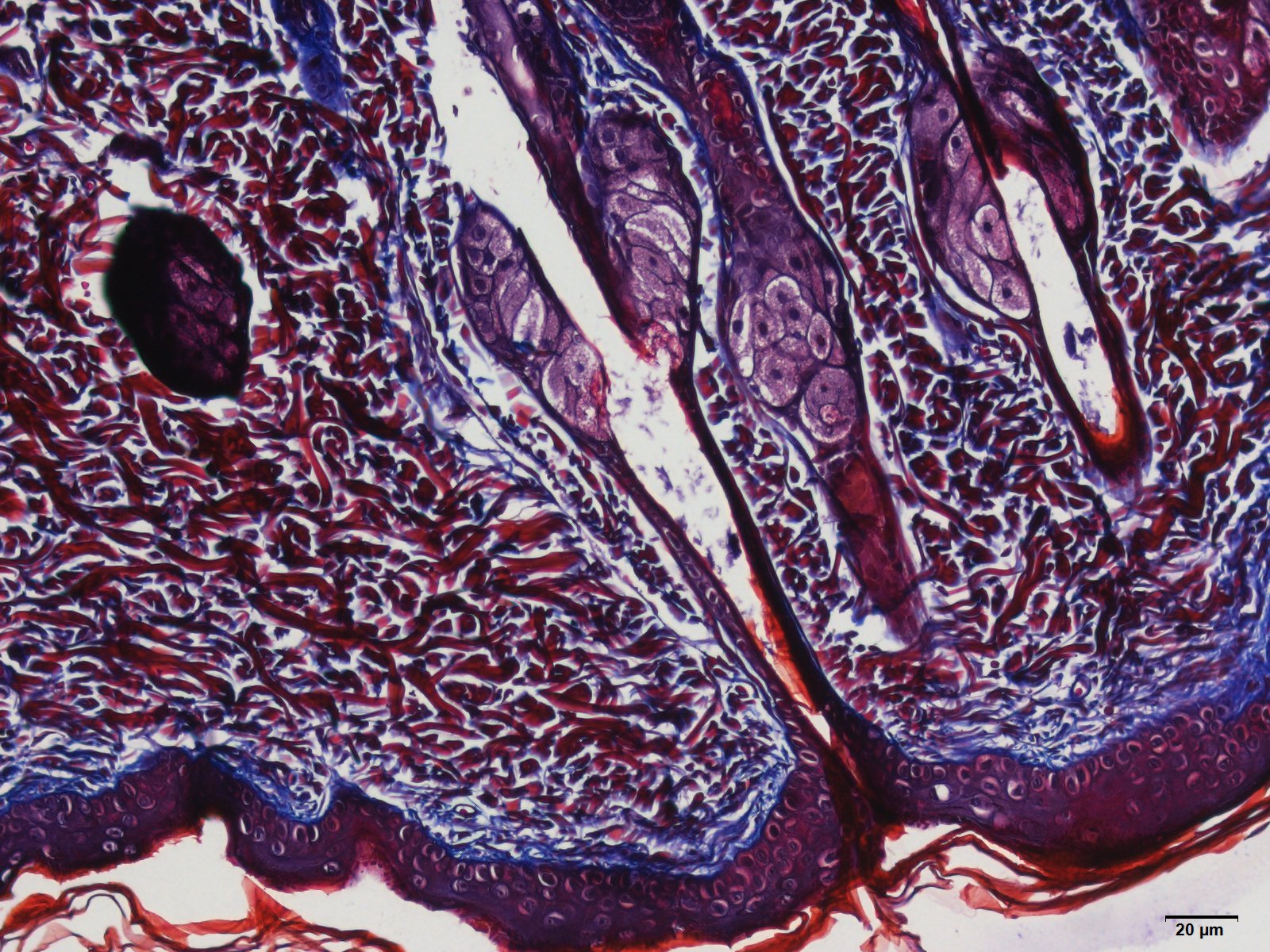

Supplement: S10 File — (ZIP) [file pone.0330078.s010.zip › Masson staning/7d Control 1.jpg]

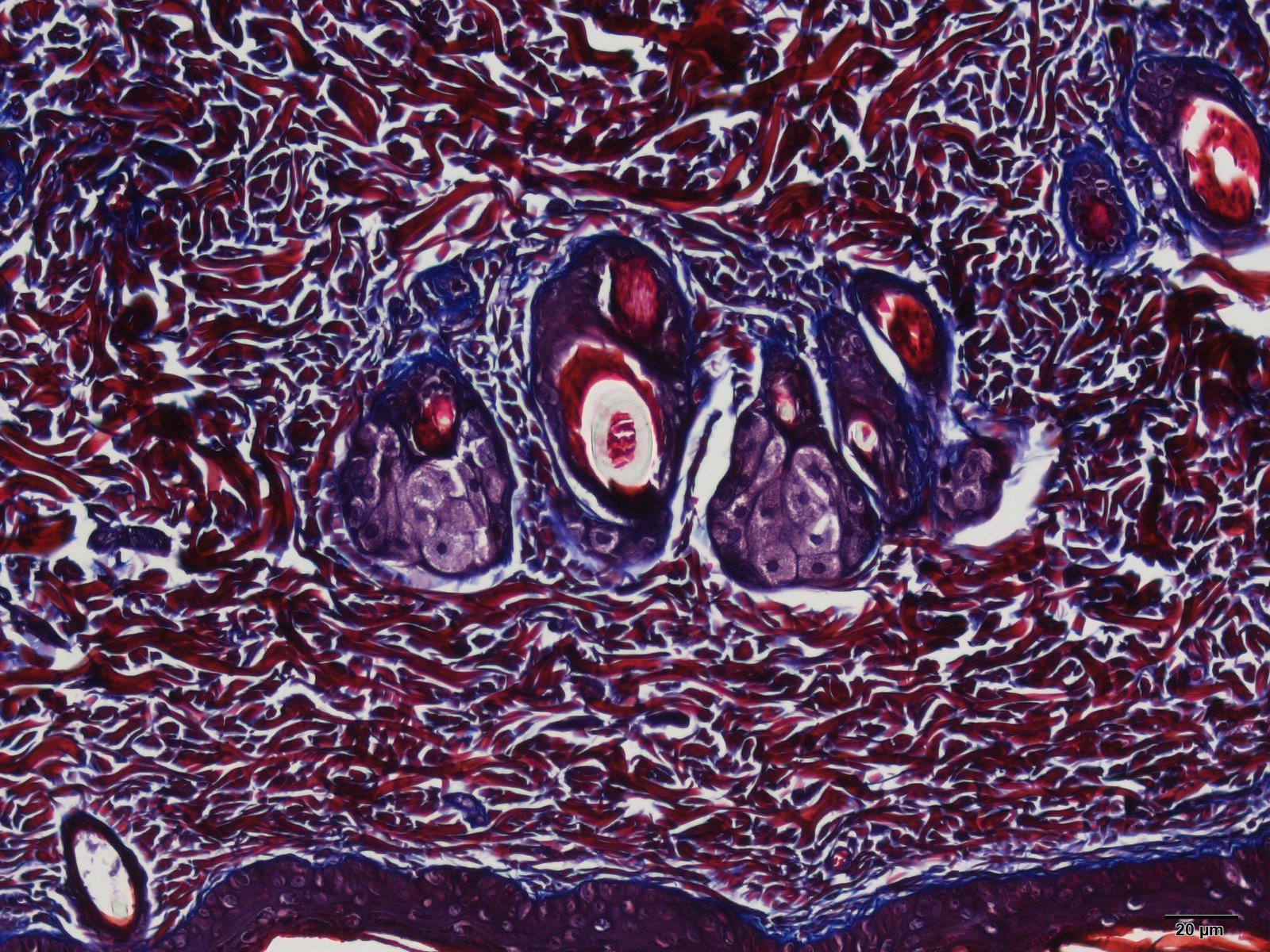

Supplement: S10 File — (ZIP) [file pone.0330078.s010.zip › Masson staning/7d Control 2.jpg]

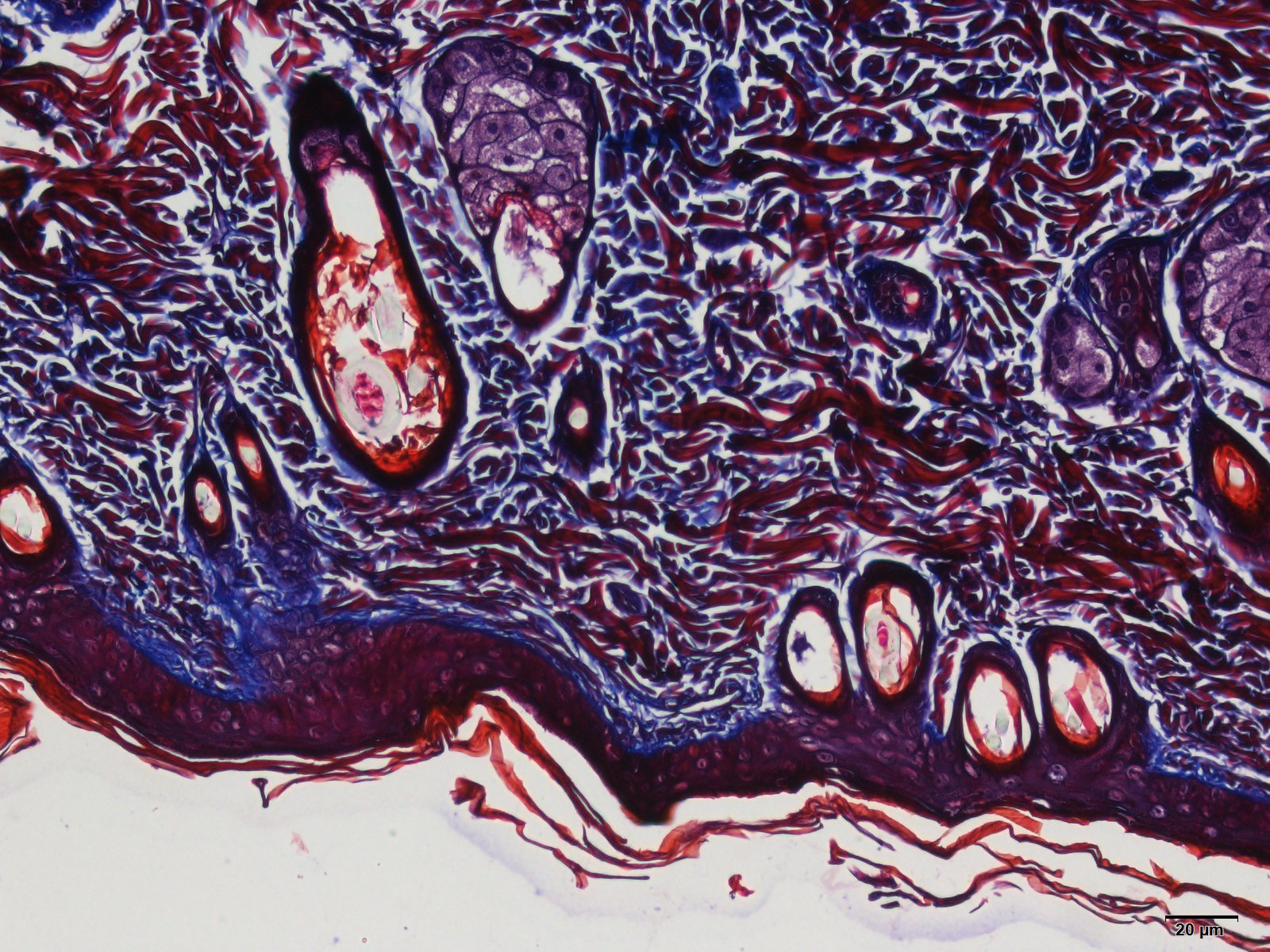

Supplement: S10 File — (ZIP) [file pone.0330078.s010.zip › Masson staning/7d Control 3.jpg]

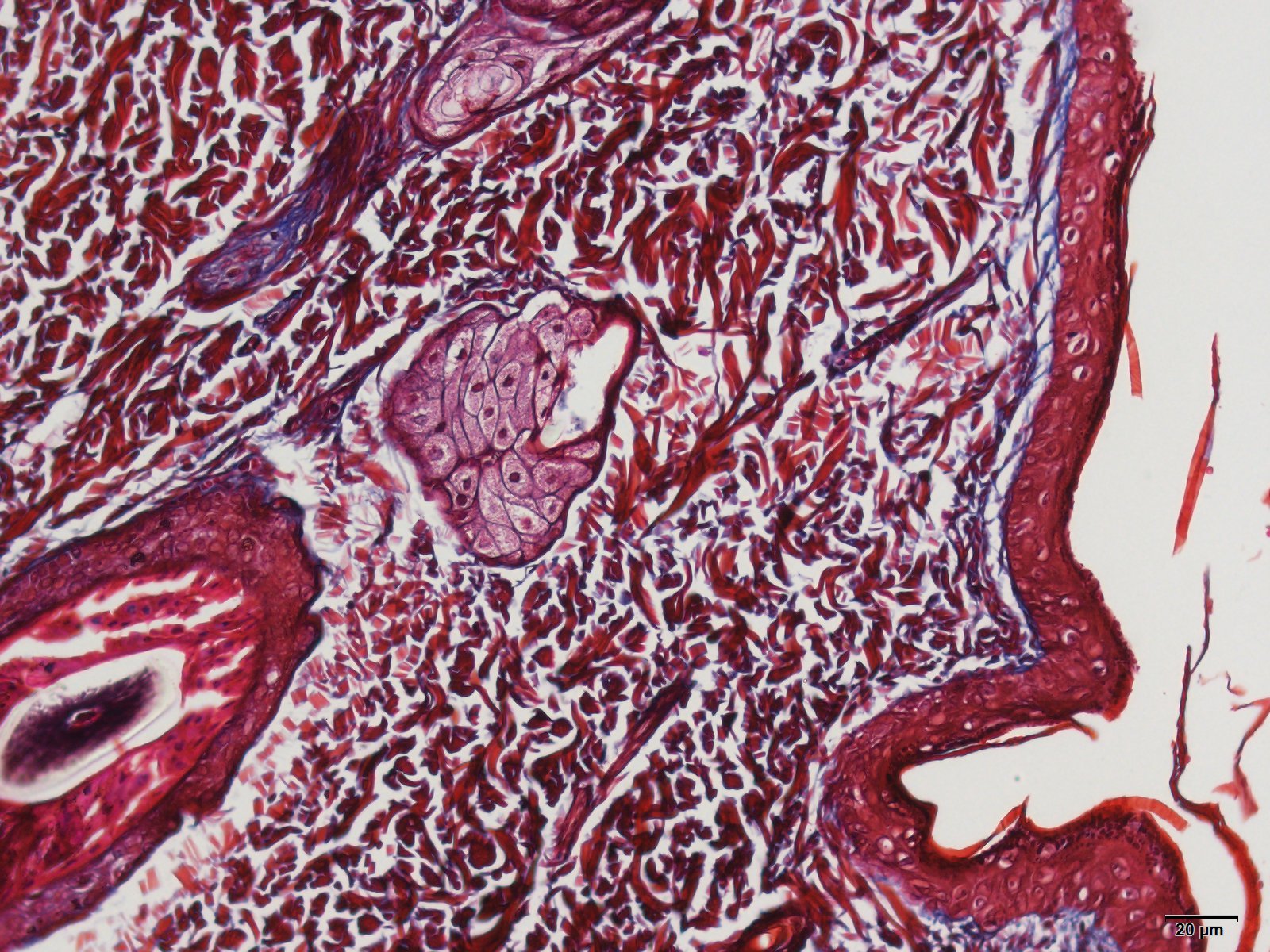

Supplement: S10 File — (ZIP) [file pone.0330078.s010.zip › Masson staning/7d HAMCC 1.jpg]

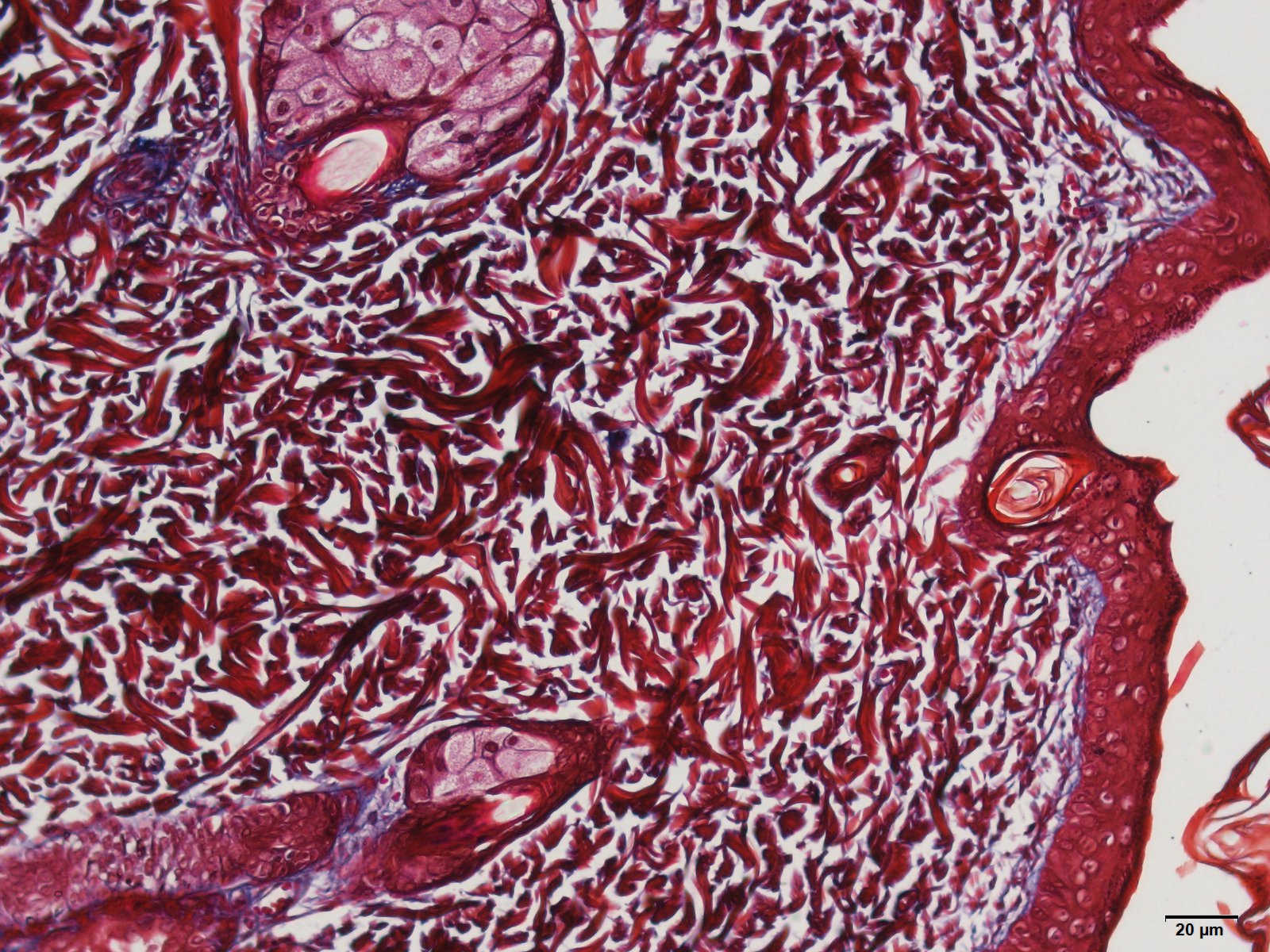

Supplement: S10 File — (ZIP) [file pone.0330078.s010.zip › Masson staning/7d HAMCC 2.jpg]

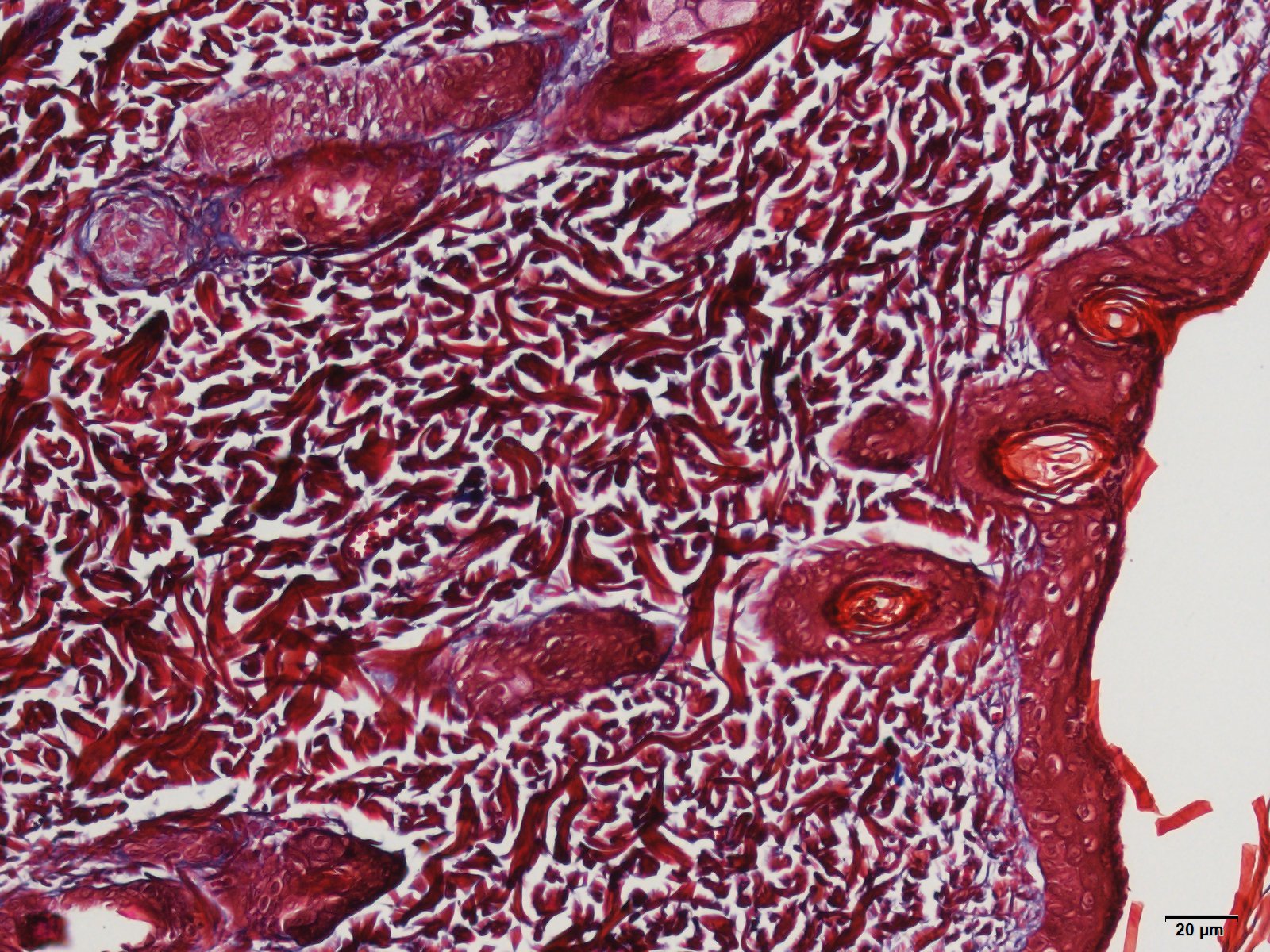

Supplement: S10 File — (ZIP) [file pone.0330078.s010.zip › Masson staning/7d HAMCC 3.jpg]
